# Supplementary material for: The Government Finance Database: A Common Resource for Quantitative Research in Public Financial Analysis
Source: PLoS One. 2015 Jun 24;10(6):e0130119. doi: 10.1371/journal.pone.0130119 (PMC4479543; doi:10.1371/journal.pone.0130119)
Supplement: S1 File — This file contains detailed information showing how to replicate our work creating the database described in the paper. The three appendices include step by step instructions, a mapping of our variable names to the census data codes, and the SAS code we used to consolidate the data files we received from the Census into the government finance database. (DOC) [file pone.0130119.s001.doc]

Appendix A: Replication of Data Consolidation

Instructions for replicating the the process of data organization we followed.

- Obtain the relevant files from the census: four (4) “fin_data” files from 2008 – 2011, and one (1) “IndFin_1967-2007.zip” file.
  - (Contact information is available from: http://www.census.gov/govs/local/)
- Extract these files to a directory on your computer
- Make sure that you have at least 20GB of space available on the drive where your SAS work directory is located, since the data files stored in memory get very large during this process.
- Paste the code from appendix C into a SAS program file.
- Change the directories referenced in the code so that they will work on your machine. There are several locations for these:
  - Line 4
    - Make sure to include the final “/IndFin0” after the file path pointing to the 1967-2007 folder.
  - Line 46
    - Make sure to include the final “/IndFin” (different from above) after the file path pointing to the 1967-2007 folder.
  - Line 114
    - This should point to the data file containing the 2011 data, the text “2011FinEstDAT_ALL5modp_pu.txt” fits the current census data, but these naming conventions change regularly.
  - Line 142
    - This should point to the file containing the 2011 government ID information
  - Line 168
    - Points to the 2010 financial data text file.
  - Line 196
    - Points to the 2010 government ID file.
  - Line 222
    - Points to the 2009 financial data text file.
  - Line 250
    - Points to the 2009 government ID file.
  - Line 276
    - Points to the 2008 financial data text file.
  - Line 304
    - Points to the 2008 government ID file.
  - Line 1189
    - This points to the location and file name where the final comma separated data file should be exported and saved.
- Run the SAS program, it should output a csv file with the consolidated data to the location you indicated on line 1189.

Appendix B: Mapping Natural Language Variables to Census Data Codes

In table A1 we display the census codes used to create each of the database variables, or present a formula in cases where the variable is computed from other database variables. Variables listed as “.” Do not have a data code in the newest census data and will not have complete coverage in the database as a result. IGR stands for “intergovernmental revenue”, NEC stands for “not elsewhere classified”, LTD stands for “long term debt”, FFC stands for “full faith and credit”, and NG stands for “not guaranteed”.

**TABLE A1**

**Corresponding Census Codes or Formulas for Each Database Variable**

| Database Variable Name | Census Data Codes or Formula |
| --- | --- |
| Total_Revenue | B01, B21, B22, B30, B42, B46, B50, B59, B79, B80, B89, B91, B92, B93, B94, C21, C30, C42, C46, C50, C79, C80, C89, C91, C92, C93, C94, D21, D30, D42, D46, D50, D79, D80, D89, D91, D92, D93, D94, T01, T09, T10, T11, T12, T13, T14, T15, T16, T19, T20, T21, T22, T23, T24, T25, T27, T28, T29, T40, T41, T50, T51, T53, T99, A01, A03, A09, A10, A12, A16, A18, A21, A36, A44, A45, A50, A56, A59, A60, A61, A80, A81, A87, A89, U01, U11, U20, U21, U30, U40, U41, U50, U95, U99, A90, A91, A92, A93, A94, X01, X02, X05, X08, Y01, Y02, Y04, Y11, Y12, Y51, Y52 |
| Total_Rev_Own_Sources | T01, T09, T10, T11, T12, T13, T14, T15, T16, T19, T20, T21, T22, T23, T24, T25, T27, T28, T29, T40, T41, T50, T51, T53, T99, A01, A03, A09, A10, A12, A16, A18, A21, A36, A44, A45, A50, A56, A59, A60, A61, A80, A81, A87, A89, U01, U11, U20, U21, U30, U40, U41, U50, U95, U99, A90, A91, A92, A93, A94, X01, X02, X05, X08, Y01, Y02, Y04, Y11, Y12, Y51, Y52 |
| General_Revenue | B01, B21, B22, B30, B42, B46, B50, B59, B79, B80, B89, B91, B92, B93, B94, C21, C30, C42, C46, C50, C79, C80, C89, C91, C92, C93, C94, D21, D30, D42, D46, D50, D79, D80, D89, D91, D92, D93, D94, T01, T09, T10, T11, T12, T13, T14, T15, T16, T19, T20, T21, T22, T23, T24, T25, T27, T28, T29, T40, T41, T50, T51, T53, T99, A01, A03, A09, A10, A12, A16, A18, A21, A36, A44, A45, A50, A56, A59, A60, A61, A80, A81, A87, A89, U01, U11, U20, U21, U30, U40, U41, U50, U95, U99 |
| Gen_Rev_Own_Sources | A01, A03, A09, A10, A12, A16, A18, A21, A36, A44, A45, A50, A56, A59, A60, A61, A80, A81, A87, A89, T01, T09, T10, T11, T12, T13, T14, T15, T16, T19, T20, T21, T22, T23, T24, T25, T27, T28, T29, T40, T41, T50, T51, T53, T99, U01, U11, U20, U21, U30, U40, U41, U50, U95, U99 |
| Total_Taxes | T01, T09, T10, T11, T12, T13, T14, T15, T16, T19, T20, T21, T22, T23, T24, T25, T27, T28, T29, T40, T41, T50, T51, T53, T99 |
| Property_Tax | T01 |
| Tot_Sales___Gr_Rec_Tax | T09, T10, T11, T12, T13, T14, T15, T16, T19 |
| Total_Gen_Sales_Tax | T09 |
| Total_Select_Sales_Tax | T10, T11, T12, T13, T14, T15, T16, T19 |
| Alcoholic_Beverage_Tax | T10 |
| Amusement_Tax | T11 |
| Insurance_Premium_Tax | T12 |
| Motor_Fuels_Tax | T13 |
| Pari_mutuels_Tax | T14 |
| Public_Utility_Tax | T15 |
| Tobacco_Tax | T16 |
| Other_Select_Sales_Tax | T19 |
| Total_License_Taxes | T20 , T21 , T22 , T23 , T24 , T25 , T27 , T28 , T29 |
| Alcoholic_Beverage_Lic | T20 |
| Amusement_License | T21 |
| Corporation_License | T22 |
| Hunting___Fishing_License | T23 |
| Motor_Vehicle_License | T24 |
| Motor_Veh_Oper_License | T25 |
| Motor_Vehicle_License_Total | T24, T25 |
| Public_Utility_License | T27 |
| Occup_and_Bus_Lic_NEC | T28 |
| Other_License_Taxes | T29 |
| Total_Income_Taxes | T40 , T41 , T50 , T51 , T53 , T99 |
| Individual_Income_Tax | T40 |
| Corp_Net_Income_Tax | T41 |
| Death_and_Gift_Tax | T50 |
| Docum_and_Stock_Tr_Tax | T51 |
| Severance_Tax | T53 |
| Taxes_NEC | T99 |
| Total_IG_Revenue | B01, B21, B22, B30, B42, B46, B50, B59, B79, B80, B89, B91, B92, B93, B94, C21, C30, C42, C46, C50, C79, C80, C89, C91, C92, C93, C94, D21, D30, D42, D46, D50, D79, D80, D89, D91, D92, D93, D94 |
| Total_Fed_IG_Revenue | B01, B21, B22, B30, B42, B46, B50, B59, B79, B80, B89, B91, B92, B93, B94 |
| Fed_IGR_Air_Transport | B01 |
| Fed_IGR_Education | B21 |
| Fed_IGR_Emp_Sec_Adm | B22 |
| Fed_IGR_Gen_Rev_Shar | . |
| Fed_IGR_Gen_Support | B30 |
| Fed_IGR_Health___Hos | B42 |
| Fed_IGR_Highways | B46 |
| Fed_IGR_Transit_Sub | B94 |
| Fed_IGR_Hous_Com_Dev | B50 |
| Fed_IGR_Natural_Res | B59 |
| Fed_IGR_Public_Welf | B79 |
| Fed_IGR_Sewerage | B80 |
| Fed_IGR_Other | B89 |
| Total_State_IG_Revenue | C21, C30, C42, C46, C50, C79, C80, C89, C91, C92, C93, C94 |
| State_IGR_Education | C21 |
| State_IGR_Tax_Relief | . |
| State_IGR_Oth_Gen_Sup | C30 |
| State_IGR_Gen_Sup | State_IGR_Oth_Gen_Sup, State_IGR_Tax_Relief |
| State_IGR_Health___Hos | C42 |
| State_IGR_Highways | C46 |
| State_IGR_Transit_Sub | C94 |
| State_IGR_Hous_Com_Dev | C50 |
| State_IGR_Public_Welf | C79 |
| State_IGR_Sewerage | C80 |
| State_IGR_Other | C89 |
| Tot_Local_IG_Rev | D21, D30, D42, D46, D50, D79, D80, D89, D91, D92, D93, D94 |
| Local_IGR_InterSchool_Aid | D11 |
| Local_IGR_Other_Education | D21 |
| Local_IGR_Oth_Gen_Sup | D30 |
| Local_IGR_Health___Hos | D42 |
| Local_IGR_Highways | D46 |
| Local_IGR_Transit_Sub | D94 |
| Local_IGR_Hous_Com_Dev | D50 |
| Local_IGR_Public_Welf | D79 |
| Local_IGR_Sewerage | D80 |
| Local_IGR_Other | D89 |
| Tot_Chgs_and_Misc_Rev | A01, A03, A09, A10, A12, A16, A18, A21, A36, A44, A45, A50, A56, A59, A60, A61, A80, A81, A87, A89, U01, U11, U20, U21, U30, U40, U41, U50, U95, U99 |
| Total_General_Charges | A01, A03, A09, A10, A12, A16, A18, A21, A36, A44, A45, A50, A56, A59, A60, A61, A80, A81, A87, A89 |
| Chg_Air_Transportation | A01 |
| Chg_Misc_Com_Activ | A03 |
| Chg_Total_Education | A09, A10, A12, A16, A18, A21 |
| Chg_Total_Elem_Education | Chg_Elem_Ed_Sch_Lunch, Chg_Elem_Ed_Tuition, Chg_Elem_Ed_NEC |
| Chg_Elem_Ed_Sch_Lunch | A09 |
| Chg_Elem_Ed_Tuition | A10 |
| Chg_Elem_Ed_NEC | A12 |
| Chg_Total_High_Ed | A16, A18 |
| Chg_Hospitals | A36 |
| Chg_Highways | Chg_Regular_Highways, Chg_Toll_Highways |
| Chg_Regular_Highways | A44 |
| Chg_Toll_Highways | A45 |
| Chg_Housing___Comm_Dev | A50 |
| Chg_Total_Nat_Res | A56 , A59 |
| Chg_Parking | A60 |
| Chg_Parks___Recreation | A61 |
| Chg_Sewerage | A80 |
| Chg_Solid_Waste_Mgmt | A81 |
| Chg_Water_Transport | A87 |
| Chg_All_Other_NEC | A89 |
| Misc_General_Revenue | U01, U11, U20, U21, U30, U40, U41, U50, U95, U99 |
| Special_Assessments | U01 |
| Prop_Sale_Total | Prop_Sale_Hous_Com_Dev, Prop_Sale_Other |
| Prop_Sale_Hous_Com_Dev | . |
| Prop_Sale_Other | U11 |
| Interest_Revenue | U20 |
| Fines_and_Forfeits | U30 |
| Rents_and_Royalties | U40 , U41 |
| Net_Lottery_Revenue | U95 |
| Misc_General_Rev_NEC | U99 |
| Liquor_Stores_Revenue | A90 |
| Total_Utility_Revenue | A91, A92, A93, A94 |
| Water_Utility_Revenue | A91 |
| Electric_Utility_Rev | A92 |
| Gas_Utility_Rev | A93 |
| Transit_Utility_Rev | A94 |
| Total_Insur_Trust_Rev | X01, X02, X05, X08, Y01, Y02, Y04, Y11, Y12, Y51, Y52 |
| Total_Insur_Trust_Ctrb | X01, X02, X05, Y01 |
| Tot_Ins_Trust_Inv_Rev | X08 , Y02 |
| Total_Emp_Ret_Rev | X01, X02, X05, X08 |
| Emp_Ret_Total_Ctrib | X01, X02, X05 |
| Emp_Ret_Loc_Emp_Ctrib | X01 |
| Emp_Ret_Loc_To_Loc_Sys | X04 |
| Emp_Ret_From_Other_Gov | X05 |
| Emp_Ret_Sta_To_Sta_Ctr | X06 |
| Emp_Ret_Int_Rev | X08 |
| Emp_Ret_Other_Earnings | . |
| Total_Unemp_Rev | Y01, Y02, Y04 |
| Unemp_Payroll_Tax | Y01 |
| Unemp_Int_Revenue | Y02 |
| Unemp_Federal_Advances | Y04 |
| Total_Expenditure | E01, E03, E04, E05, E12, E16, E18, E21, E22, E23, E24, E25, E26, E29, E31, E32, E36, E44, E44, E45, E50, E52, E55, E56, E59, E60, E61, E62, E66, E74, E75, E77, E79, E80, E81, E85, E87, E89, E90, E91, E92, E93, E94, I89, I91, I92, I93, I94, J19, J67, J68, J85, X11, X12, Y05, Y06, Y14, Y53, F01, F03, F04, F05, F12, F16, F18, F21, F22, F23, F24, F25, F26, F29, F31, F32, F36, F44, F45, F50, F52, F55, F56, F59, F60, F61, F62, F66, F77, F79, F80, F81, F85, F87, F89, F90, F91, F92, F93, F94, G01, G03, G04, G05, G12, G16, G18, G21, G22, G23, G24, G25, G26, G29, G31, G32, G36, G44, G45, G50, G52, G55, G56, G59, G60, G61, G62, G66, G77, G79, G80, G81, G85, G87, G89, G90, G91, G92, G93, G94, L01, L04, L05, L12, L18, L23, L25, L29, L32, L36, L44, L52, L59, L60, L61, L62, L66, L67, L79, L80, L81, L87, L89, L91, L92, L93, L94, M01, M04, M05, M12, M18, M21, M23, M24, M25, M29, M30, M32, M36, M44, M50, M52, M55, M56, M59, M60, M61, M62, M66, M67, M68, M79, M80, M81, M87, M89, M91, M92, M93, M94, Q12, Q18, S67, S74, S89 |
| Total_IG_Expenditure | L01, L04, L05, L12, L18, L23, L25, L29, L32, L36, L44, L50, L52, L59, L60, L61, L62, L66, L67, L79, L80, L81, L87, L89, L91, L92, L93, L94, M01, M04, M05, M12, M18, M21, M23, M24, M25, M29, M30, M32, M36, M44, M50, M52, M52, M55, M56, M59, M60, M61, M62, M66, M67, M68, M79, M80, M81, M87, M89, M91, M92, M93, M94, Q12, Q18, S67, S89 |
| Direct_Expenditure | E01, E03, E04, E05, E12, E16, E18, E21, E22, E23, E24, E25, E26, E29, E31, E32, E36, E44, E45, E50, E52, E55, E56, E59, E60, E61, E62, E66, E74, E75, E77, E79, E80, E81, E85, E87, E89, E90, E91, E92, E93, E94, F01, F03, F04, F05, F12, F16, F18, F21, F22, F23, F24, F25, F26, F29, F31, F32, F36, F44, F45, F50, F52, F55, F56, F59, F60, F61, F62, F66, F77, F79, F80, F81, F85, F87, F89, F90, F91, F92, F93, F94, G01, G03, G04, G05, G12, G16, G18, G21, G22, G23, G24, G25, G26, G29, G31, G32, G36, G44, G45, G50, G52, G55, G56, G59, G60, G61, G62, G66, G77, G79, G80, G81, G85, G87, G89, G90, G91, G92, G93, G94, X11, X12, Y05, Y06, Y14, Y53, J19, J67, J68, J85, I89, I91, I92, I93, I94 |
| Total_Current_Expend | Total_Expenditure, - Total_Capital_Outlays |
| Total_Current_Oper | E01, E03, E04, E05, E12, E16, E18, E21, E22, E23, E24, E25, E26, E29, E31, E32, E36, E44, E45, E50, E52, E55, E56, E59, E60, E61, E62, E66, E74, E75, E77, E79, E80, E81, E85, E87, E89, E90, E91, E92, E93, E94 |
| Total_Capital_Outlays | F01, F03, F04, F05, F12, F16, F18, F21, F22, F23, F24, F25, F26, F29, F31, F32, F36, F44, F45, F50, F52, F55, F56, F59, F60, F61, F62, F66, F77, F79, F80, F81, F85, F87, F89, F90, F91, F92, F93, F94, G01, G03, G04, G05, G12, G16, G18, G21, G22, G23, G24, G25, G26, G29, G31, G32, G36, G44, G45, G50, G52, G55, G56, G59, G60, G61, G62, G66, G77, G79, G80, G81, G85, G87, G89, G90, G91, G92, G93, G94 |
| Total_Construction | F01, F03, F04, F05, F12, F16, F18, F21, F22, F23, F24, F25, F26, F29, F31, F32, F36, F44, F45, F50, F52, F55, F56, F59, F60, F61, F62, F66, F77, F79, F80, F81, F85, F87, F89, F90, F91, F92, F93, F94 |
| Total_Other_Capital_Outlays | Total_Capital_Outlays, -Total_Construction |
| Tot_Assist___Subsidies | J19, J67, J68, J85 |
| Total_Interest_on_Debt | I89, I91, I92, I93, I94 |
| Total_Insur_Trust_Ben | X11, X12, Y05, Y06, Y14, Y53 |
| Total_Salaries___Wages | Z00 |
| General_Expenditure | E01, E03, E04, E05, E12, E16, E18, E21, E22, E23, E24, E25, E26, E29, E31, E32, E36, E44, E45, E50, E52, E55, E56, E59, E60, E61, E62, E66, E74, E75, E77, E79, E80, E81, E85, E87, E89, E90, E91, E92, E93, E94, F01, F03, F04, F05, F12, F16, F18, F21, F22, F23, F24, F25, F26, F29, F31, F32, F36, F44, F45, F50, F52, F55, F56, F59, F60, F61, F62, F66, F77, F79, F80, F81, F85, F87, F89, F90, F91, F92, F93, F94, G01, G03, G04, G05, G12, G16, G18, G21, G22, G23, G24, G25, G26, G29, G31, G32, G36, G44, G45, G50, G52, G55, G56, G59, G60, G61, G62, G66, G77, G79, G80, G81, G85, G87, G89, G90, G91, G92, G93, G94, X11, X12, Y05, Y06, Y14, Y53, J19, J67, J68, J85, I89, I91, I92, I93, I94 |
| IG_Exp_To_State_Govt | L01, L04, L05, L12, L18, L23, L25, L29, L32, L36, L44, L50, L52, L59, L60, L61, L62, L66, L67, L79, L80, L81, L87, L89, L91, L92, L93, L94 |
| IG_Exp_To_Local_Govts | M01, M04, M05, M12, M18, M21, M23, M24, M25, M29, M30, M32, M36, M44, M50, M52, M52, M55, M56, M59, M60, M61, M62, M66, M67, M68, M79, M80, M81, M87, M89, M91, M92, M93, M94 |
| IG_Exp_To_Federal_Govt | S67, S74, S89 |
| Direct_General_Expend | E01, E03, E04, E05, E12, E16, E18, E21, E22, E23, E24, E25, E26, E29, E31, E32, E36, E44, E45, E50, E52, E55, E56, E59, E60, E61, E62, E66, E74, E75, E77, E79, E80, E81, E85, E87, E89, E90, E91, E92, E93, E94, F01, F03, F04, F05, F12, F16, F18, F21, F22, F23, F24, F25, F26, F29, F31, F32, F36, F44, F45, F50, F52, F55, F56, F59, F60, F61, F62, F66, F77, F79, F80, F81, F85, F87, F89, F90, F91, F92, F93, F94, G01, G03, G04, G05, G12, G16, G18, G21, G22, G23, G24, G25, G26, G29, G31, G32, G36, G44, G45, G50, G52, G55, G56, G59, G60, G61, G62, G66, G77, G79, G80, G81, G85, G87, G89, G90, G91, G92, G93, G94, X11, X12, Y05, Y06, Y14, Y53, J19, J67, J68, J85, I89, I91, I92, I93, I94 |
| General_Current_Expend | General_Expenditure, - General_Capital_Outlay |
| General_Current_Oper | E01, E03, E04, E05, E12, E16, E18, E21, E22, E23, E24, E25, E26, E29, E31, E32, E36, E44, E45, E50, E52, E55, E56, E59, E60, E61, E62, E66, E74, E75, E77, E79, E80, E81, E85, E87, E89, E90, E91, E92, E93, E94 |
| General_Capital_Outlay | F01, F03, F04, F05, F12, F16, F18, F21, F22, F23, F24, F25, F26, F29, F31, F32, F36, F44, F45, F50, F52, F55, F56, F59, F60, F61, F62, F66, F77, F79, F80, F81, F85, F87, F89, G01, G03, G04, G05, G12, G16, G18, G21, G22, G23, G24, G25, G26, G29, G31, G32, G36, G44, G45, G50, G52, G55, G56, G59, G60, G61, G62, G66, G77, G79, G80, G81, G85, G87, G89 |
| General_Construction | F01, F03, F04, F05, F12, F16, F18, F21, F22, F23, F24, F25, F26, F29, F31, F32, F36, F44, F45, F50, F52, F55, F56, F59, F60, F61, F62, F66, F77, F79, F80, F81, F85, F87, F89, F90, F91, F92, F93, F94 |
| General_Capital_Outlay_Other | General_Capital_Outlay, -General_Construction |
| General_Assist___Sub | Tot_Assist___Subsidies |
| General_Debt_Interest | I89 |
| Air_Trans_Total_Expend | E01 , F01 , G01 , L01 , M01 |
| Air_Trans_Direct_Expend | E01 , F01 , G01 |
| Air_Trans_Cap_Outlay | F01 , G01 |
| Air_Trans_Current_Exp | Air_Trans_Direct_Expend, -Air_Trans_Cap_Outlay |
| Air_Trans_Construction | F01 |
| Air_Trans_IG_To_State | L01 |
| Air_Trans_IG_Local_Govts | M01 |
| Misc_Com_Activ_Tot_Exp | E03 , F03 , G03 |
| Misc_Com_Activ_Cap_Out | F03 , G03 |
| Misc_Com_Activ_Current_Exp | Misc_Com_Activ_Tot_Exp, -Misc_Com_Activ_Cap_Out |
| Misc_Com_Activ_Constr | F03 |
| Correct_Total_Exp | E04 , F04 , G04 , E05 , F05 , G05 , L04 , L05 , M04 , M05 |
| Correct_Direct_Exp | E04 , E05 , F04 , G04 , F05 , G05 |
| Correct_Cap_Outlay | F04 , G04 , F05 , G05 |
| Correct_Current_Exp | Correct_Direct_Exp, -Correct_Cap_Outlay |
| Correct_Construct | F04 , F05 |
| Correct_IG_To_St | L04 , L05 |
| Correct_IG_Loc_Govts | M04 , M05 |
| Total_Educ_Total_Exp | E12, F12, G12, E16, F16, G16, E18, F18, G18, J19, E21, F21, G21, L12, M12, Q12, L18, M18, L21, M21 |
| Total_Educ_Direct_Exp | E12, F12, G12, E16, F16, G16, E18, F18, G18, J19, E21, F21, G21 |
| Total_Educ_Assist___Sub | J19 |
| Total_Educ_Cap_Outlay | F12, F16, F18, F21, G12, G16, G18, G21 |
| Total_Educ_Current_Exp | Total_Educ_Direct_Exp, -Total_Educ_Cap_Outlay |
| Total_Educ_Construct | F12 , F16 , F18 , F21 |
| Elem_Educ_Total_Exp | E12, F12, G12, L12, M12, Q12 |
| Elem_Educ_Direct_Exp | E12, F12, G12 |
| Elem_Educ_Cap_Outlay | F12, G12 |
| Elem_Educ_Current_Exp | Elem_Educ_Direct_Exp, -Elem_Educ_Cap_Outlay |
| Elem_Educ_Construction | F12 |
| Elem_Educ_IG_To_State | L12 |
| Elem_Educ_IG_Local_Govts | M12 |
| Elem_Educ_IG_Sch_to_Sch | Q12 |
| Higher_Ed_Total_Exp | E16, E18, F16, F18, G16, G18, L18, M18 |
| Higher_Ed_Direct_Exp | E16, E18, F16, F18, G16, G18 |
| Higher_Ed_Cap_Outlay | F16, F18, G16, G18 |
| Higher_Ed_Current_Exp | Higher_Ed_Direct_Exp, -Higher_Ed_Cap_Outlay |
| Higher_Ed_Construct | F16, F18 |
| Higher_Ed_IG_To_St | L18 |
| Higher_Ed_IG_Loc_Govts | M18 |
| Educ_NEC_Total_Expend | E21, F21, G21, L21, M21 |
| Educ_NEC_Direct_Expend | E21, F21, G21 |
| Educ_NEC_Assistance | . |
| Educ_NEC_Cap_Outlay | F21, G21 |
| Educ_NEC_Current_Exp | Educ_NEC_Direct_Expend, -Educ_NEC_Cap_Outlay |
| Educ_NEC_Construction | F21 |
| Educ_NEC_IG_To_State | L21 |
| Educ_NEC_IG_Local_Govts | M21 |
| Emp_Sec_Adm_Direct_Exp | E22 , F22 , G22 |
| Emp_Sec_Adm_Cap_Outlay | F22 , G22 |
| Emp_Sec_Adm_Current_Exp | Emp_Sec_Adm_Direct_Exp, -Emp_Sec_Adm_Cap_Outlay |
| Emp_Sec_Adm_Construct | F22 |
| Fin_Admin_Total_Exp | E23, F23, G23, L23, M23 |
| Fin_Admin_Direct_Exp | E23, F23, G23 |
| Fin_Admin_Cap_Outlay | F23, G23 |
| Fin_Admin_Current_Exp | Fin_Admin_Direct_Exp, -Fin_Admin_Cap_Outlay |
| Fin_Admin_Construction | F23 |
| Fin_Admin_IG_To_State | L23 |
| Fin_Admin_IG_Local_Govts | M23 |
| Fire_Prot_Total_Expend | E24, F24, G24, L24, M24 |
| Fire_Prot_Direct_Exp | E24, F24, G24 |
| Fire_Prot_Cap_Outlay | F24, G24 |
| Fire_Prot_Current_Exp | Fire_Prot_Direct_Exp, -Fire_Prot_Cap_Outlay |
| Fire_Prot_Construction | F24 |
| Fire_Prot_IG_To_State | L24 |
| Fire_Prot_IG_Local_Govts | M24 |
| Judicial_Total_Expend | E25, F25, G25, L25, M25 |
| Judicial_Direct_Expend | E25, F25, G25 |
| Judicial_Cap_Outlay | F25, G25 |
| Judicial_Current_Exp | Judicial_Direct_Expend, -Judicial_Cap_Outlay |
| Judicial_Construction | F25 |
| Judicial_IG_To_State | L25 |
| Judicial_IG_Local_Govts | M25 |
| Cen_Staff_Total_Expend | E29, F29, G29, L29, M29 |
| Cen_Staff_Direct_Exp | E29, F29, G29 |
| Cen_Staff_Cap_Outlay | F29, G29 |
| Cen_Staff_Current_Exp | Cen_Staff_Direct_Exp, -Cen_Staff_Cap_Outlay |
| Cen_Staff_Construction | F29 |
| Cen_Staff_IG_To_State | L29 |
| Cen_Staff_IG_Local_Govts | M29 |
| Gen_Pub_Bldg_Total_Exp | E31, F31, G31 |
| Gen_Pub_Bldg_Cap_Out | F31, G31 |
| Gen_Pub_Bldg_Current_Exp | Gen_Pub_Bldg_Total_Exp, -Gen_Pub_Bldg_Cap_Out |
| Gen_Pub_Bldg_Construct | F31 |
| Health_Total_Expend | E32, F32, G32, L32, M32 |
| Health_Direct_Expend | E32, F32, G32 |
| Health_Capital_Outlay | F32, G32 |
| Health_Current_Exp | Health_Direct_Expend, -Health_Capital_Outlay |
| Health_Construction | F32 |
| Health_IG_To_State | L32 |
| Health_IG_Local_Govts | M32 |
| Total_Hospital_Total_Exp | E36, F36, G36, L36, M36 |
| Total_Hospital_Dir_Exp | E36, F36, G36 |
| Total_Hospital_Cap_Out | F36, G36 |
| Total_Hospital_Current_Exp | Total_Hospital_Dir_Exp, -Total_Hospital_Cap_Out |
| Total_Hospital_Construct | F36 |
| Total_Hospital_IG_To_State | L36 |
| Total_Hospital_IG_Loc_Govts | M36 |
| Own_Hospital_Total_Exp | E37, F37, G37 |
| Own_Hospital_Cap_Out | F37, G37 |
| Own_Hospital_Current_Exp | Own_Hospital_Total_Exp, -Own_Hospital_Cap_Out |
| Own_Hospital_Construct | F37 |
| Hosp_Other_Total_Exp | E39, F39, G39, L39, M39 |
| Hosp_Other_Direct_Exp | E39, F39, G39 |
| Hosp_Other_Cap_Outlay | F39, G39 |
| Hosp_Other_Current_Exp | Hosp_Other_Direct_Exp, -Hosp_Other_Cap_Outlay |
| Hosp_Other_Construct | F39 |
| Hosp_Other_IG_To_State | L39 |
| Hosp_Other_IG_Loc_Govts | M39 |
| Total_Highways_Tot_Exp | E44, F44, G44, E45, F45, G45, L44, M44 |
| Total_Highways_Dir_Exp | E44, F44, G44, E45, F45, G45 |
| Total_Highways_Cap_Out | F44, G44, F45, G45 |
| Total_Highways_Current_Exp | Total_Highways_Dir_Exp, -Total_Highways_Cap_Out |
| Total_Highways_Construct | F44, F45 |
| Regular_Hwy_Total_Exp | E44, F44, G44, L44, M44 |
| Regular_Hwy_Direct_Exp | E44, F44, G44 |
| Regular_Hwy_Cap_Outlay | F44, G44 |
| Regular_Hwy_Current_Exp | Regular_Hwy_Direct_Exp, -Regular_Hwy_Cap_Outlay |
| Regular_Hwy_Construct | F44 |
| Regular_Hwy_IG_To_Sta | L44 |
| Regular_Hwy_IG_Loc_Govts | M44 |
| Toll_Hwy_Total_Expend | E45, F45, G45 |
| Toll_Hwy_Cap_Outlay | F45, G45 |
| Toll_Hwy_Current_Exp | Toll_Hwy_Total_Expend, -Toll_Hwy_Cap_Outlay |
| Toll_Hwy_Construction | F45 |
| Transit_Sub_Total_Exp | . |
| Transit_Sub_Direct_Sub | . |
| Transit_Sub_IG_To_Sta | . |
| Transit_Sub_IG_Loc_Govts | . |
| Transit_Sub_To_Own_Sys | . |
| Hous___Com_Total_Exp | E50, F50, G50, L50, M50 |
| Hous___Com_Direct_Exp | E50, F50, G50 |
| Hous___Com_Cap_Outlay | F50, G50 |
| Hous___Com_Current_Exp | Hous___Com_Direct_Exp, -Hous___Com_Cap_Outlay |
| Hous___Com_Construct | F50 |
| Hous___Com_IG_To_State | L50 |
| Hous___Com_IG_Loc_Govts | M50 |
| Libraries_Total_Expend | E52, F52, G52, L52, M52 |
| Libraries_Direct_Exp | E52, F52, G52 |
| Libraries_Cap_Outlay | F52, G52 |
| Libraries_Current_Exp | Libraries_Direct_Exp, -Libraries_Cap_Outlay |
| Libraries_Construction | F52 |
| Libraries_IG_To_State | L52 |
| Libraries_IG_Local_Govts | M52 |
| Natural_Res_Total_Exp | E55, F55, G55, M55, E56, F56, G56, M56, E59, F59, G59, L59, M59 |
| Natural_Res_Direct_Exp | E55, F55, G55, E56, F56, G56, E59, F59, G59 |
| Natural_Res_Cap_Outlay | F55, G55, F56, G56, F59, G59 |
| Natural_Res_Current_Exp | Natural_Res_Direct_Exp, -Natural_Res_Cap_Outlay |
| Natural_Res_Construct | F55, F56, F59 |
| Natural_Res_IG_To_Sta | L59 |
| Natural_Res_IG_Loc_Govts | M55, M56 , M59 |
| Parking_Total_Expend | E60, F60, G60, L60, M60 |
| Parking_Direct_Expend | E60, F60, G60 |
| Parking_Capital_Outlay | F60, G60 |
| Parking_Current_Exp | Parking_Direct_Expend, -Parking_Capital_Outlay |
| Parking_Construction | F60 |
| Parking_IG_To_State | L60 |
| Parking_IG_Local_Govts | M60 |
| Parks___Rec_Total_Exp | E61, F61, G61, L61, M61 |
| Parks___Rec_Direct_Exp | E61, F61, G61 |
| Parks___Rec_Cap_Outlay | F61, G61 |
| Parks___Rec_Current_Exp | Parks___Rec_Direct_Exp, -Parks___Rec_Cap_Outlay |
| Parks___Rec_Construct | F61 |
| Parks___Rec_IG_To_Sta | L61 |
| Parks___Rec_IG_Loc_Govts | M61 |
| Police_Prot_Total_Exp | E62, F62, G62, L62, M62 |
| Police_Prot_Direct_Exp | E62, F62, G62 |
| Police_Prot_Cap_Outlay | F62, G62 |
| Police_Prot_Current_Exp | Police_Prot_Direct_Exp, -Police_Prot_Cap_Outlay |
| Police_Prot_Construct | F62 |
| Police_Prot_IG_To_Sta | L62 |
| Police_Prot_IG_Loc_Govts | M62 |
| Prot_Insp_Total_Exp | E66, F66, G66, L66, M66 |
| Prot_Insp_Direct_Exp | E66, F66, G66 |
| Prot_Insp_Cap_Outlay | F66, G66 |
| Prot_Insp_Current_Exp | Prot_Insp_Direct_Exp, -Prot_Insp_Cap_Outlay |
| Prot_Insp_Construction | F66 |
| Prot_Insp_IG_To_State | L66 |
| Prot_Insp_IG_Local_Govts | M66 |
| Public_Welf_Total_Exp | J67, L67, M67, J68 , M68, E74, E75, S74, E77, F77, G77, E79, F79, G79, L79, M79 |
| Public_Welf_Direct_Exp | J67, J68 , E74, E75, E77, F77, G77, E79, F79, G79 |
| Public_Welf_Cash_Asst | J67, J68, M67, M68 |
| Public_Welf_Cap_Outlay | F77, G77, F79, G79 |
| Public_Welf_Current_Exp | Public_Welf_Direct_Exp, -Public_Welf_Cap_Outlay, -Public_Welf_Cash_Asst |
| Public_Welf_Construct | F77, F79 |
| Welf_Categ_Total_Exp | J67, L67, M67 |
| Welf_Categ_Cash_Assist | J67 |
| Welf_Categ_IG_To_State | L67 |
| Welf_Categ_IG_Loc_Govts | M67 |
| Welf_Cash_Total_Exp | J68 , M68 |
| Welf_Cash_Cash_Assist | J68 |
| Welf_Cash_IG_Local_Govts | M68 |
| Welf_Vend_Pmts_Medical | E74 |
| Welf_Vend_Pmts_NEC | E75 |
| Welf_State_Share_Part_D | S74 |
| Welf_Ins_Total_Exp | E77, F77, G77 |
| Welf_Ins_Cap_Outlay | F77, G77 |
| Welf_Ins_Current_Exp |  |
| Welf_Ins_Construction | F77 |
| Welf_NEC_Total_Expend | E79, F79, G79, L79, M79 |
| Welf_NEC_Direct_Expend | E79, F79, G79 |
| Welf_NEC_Cap_Outlay | F79, G79 |
| Welf_NEC_Current_Exp | Welf_NEC_Direct_Expend, -Welf_NEC_Cap_Outlay |
| Welf_NEC_Construction | F79 |
| Welf_NEC_IG_To_State | L79 |
| Welf_NEC_IG_Local_Govts | M79 |
| Sewerage_Total_Expend | E80, F80, G80, L80, M80 |
| Sewerage_Direct_Expend | E80, F80, G80 |
| Sewerage_Cap_Outlay | F80, G80 |
| Sewerage_Current_Exp | Sewerage_Direct_Expend, -Sewerage_Cap_Outlay |
| Sewerage_Construction | F80 |
| Sewerage_IG_To_State | L80 |
| Sewerage_IG_Local_Govts | M80 |
| SW_Mgmt_Total_Expend | E81, F81, G81, L81, M81 |
| SW_Mgmt_Direct_Expend | E81, F81, G81 |
| SW_Mgmt_Capital_Outlay | F81, G81 |
| SW_Mgmt_Current_Exp | SW_Mgmt_Direct_Expend, -SW_Mgmt_Capital_Outlay |
| SW_Mgmt_Construction | F81 |
| SW_Mgmt_IG_To_State | L81 |
| SW_Mgmt_IG_Local_Govts | M81 |
| Water_Trans_Total_Exp | E87, F87, G87, L87, M87 |
| Water_Trans_Direct_Exp | E87, F87, G87 |
| Water_Trans_Cap_Outlay | F87, G87 |
| Water_Trans_Current_Exp | Water_Trans_Direct_Exp, -Water_Trans_Cap_Outlay |
| Water_Trans_Construct | F87 |
| Water_Trans_IG_To_Sta | L87 |
| Water_Trans_IG_Loc_Govts | M87 |
| Interest_on_Gen_Debt | I89 |
| General_NEC_Total_Exp | E89, F89, G89, L89, M89, S89, J89 |
| General_NEC_Direct_Exp | E89, F89, G89, J89 |
| VetBonus | J89 |
| General_NEC_Cap_Outlay | F89, G89 |
| General_NEC_Current_Exp | General_NEC_Direct_Exp, -General_NEC_Cap_Outlay, -VetBonus |
| General_NEC_Construct | F89 |
| General_NEC_IG_To_St | L89 |
| General_NEC_IG_Loc_Govts | M89 |
| General_NEC_IG_To_Fed | S89 |
| Liquor_Stores_Tot_Exp | E90, F90, G90 |
| Liquor_Stores_Cap_Out | F90, G90 |
| Liquor_Stores_Current_Exp | Liquor_Stores_Tot_Exp, -Liquor_Stores_Cap_Out |
| Liquor_Stores_Constr | F90 |
| Total_Util_Total_Exp | E91, I91, F91, G91, L91, M91, E92, I92, F92, G92, L92, M92, E93, I93, F93, G93, L93, M93, E94, I94, F94, G94, L94, M94 |
| Total_Util_Inter_Exp | I91, I92, I93, I94 |
| Total_Util_Cap_Outlay | F91, F92, F93, F94, G91, G92, G93, G94 |
| Total_Util_Current_Exp | Total_Util_Total_Exp, -Total_Util_Inter_Exp, -Total_Util_Cap_Outlay |
| Total_Util_Construct | F91, F92, F93, F94 |
| Water_Util_Total_Exp | E91, I91, F91, G91, L91, M91 |
| Water_Util_Inter_Exp | I91 |
| Water_Util_Cap_Outlay | F91, G91 |
| Water_Util_Current_Exp | Water_Util_Total_Exp, -Water_Util_Inter_Exp, -Water_Util_Cap_Outlay |
| Water_Util_Construct | F91 |
| Elec_Util_Total_Exp | E92, I92, F92, G92, L92, M92 |
| Elec_Util_Inter_Exp | I92 |
| Elec_Util_Cap_Outlay | F92, G92 |
| Elec_Util_Current_Exp | Elec_Util_Total_Exp, -Elec_Util_Inter_Exp, -Elec_Util_Cap_Outlay |
| Elec_Util_Construct | F92 |
| Gas_Util_Total_Exp | E93, I93, F93, G93, L93, M93 |
| Gas_Util_Inter_Exp | I93 |
| Gas_Util_Cap_Outlay | F93, G93 |
| Gas_Util_Current_Exp | Gas_Util_Total_Exp, -Gas_Util_Inter_Exp, -Gas_Util_Cap_Outlay |
| Gas_Util_Construct | F93 |
| Trans_Util_Total_Exp | E94, I94, F94, G94, L94, M94 |
| Trans_Util_Inter_Exp | I94 |
| Trans_Util_Cap_Outlay | F94, G94 |
| Trans_Util_Current_Exp Trans_Util_Total_Exp, -Trans_Util_Inter_Exp, -Trans_Util_Cap_Outlay | Trans_Util_Total_Exp, -Trans_Util_Inter_Exp, -Trans_Util_Cap_Outlay |
| Trans_Util_Construct | F94 |
| Emp_Ret_Total_Expend | X11, X12 |
| Emp_Ret_Benefit_Paymts | X11 |
| Emp_Ret_Withdrawals | X12 |
| Emp_Ret_Other_Paymts | . |
| Unemp_Comp_Total_Exp | Y05, Y06 |
| Unemp_Comp_Ben_Paymts | Y05 |
| Unemp_Ext___Spec_Pmts | Y06 |
| Total_Debt_Outstanding | _44T, _49U, _64V |
| Total_Long_Term_Debt_Out | _44T, _49U |
| ST_Debt_End_of_Year | _64V |
| Total_Beg_LTD_Out | _19T, _19U |
| Beg_LTD_Out_Private_Purp | _19T |
| Beg_LTD_Out_All_Other | _19U |
| Beg_LTD_Out_Utility | . *All detailed debt codes were discontinued in 2005 |
| Beg_LTD_Out_Water_Util | . |
| Beg_LTD_Out_Elec_Util | . |
| Beg_LTD_Out_Gas_Util | . |
| Beg_LTD_Out_Trans_Util | . |
| Beg_LTD_Out_General | . |
| Beg_LTD_Out_Education | . |
| Beg_LTD_Out_Priv_Purp | . |
| Beg_LTD_Out_Other_NEC | . |
| Total_LTD_Issued | _24T, _29U |
| LTD_Iss_Private_Purp | _24T |
| LTD_Iss_All_Other | _29U |
| LTD_Iss_Utility | . |
| LTD_Iss_Util_Water | . |
| LTD_Iss_Util_Electric | . |
| LTD_Iss_Util_Gas_Supply | . |
| LTD_Iss_Util_Transit | . |
| LTD_Iss_General | . |
| LTD_Iss_Gen_Elem_Educ | . |
| LTD_Iss_Gen_Other_Educ | . |
| LTD_Iss_Gen_Other_NEC | . |
| Total_LTD_Iss_FFC | . |
| LTD_Iss_FFC_Utility | . |
| LTD_Iss_FFC_Water_Util | . |
| LTD_Iss_FFC_Elec_Util | . |
| LTD_Iss_FFC_Gas_Util | . |
| LTD_Iss_FFC_Trans_Util | . |
| LTD_Iss_FFC_General | . |
| LTD_Iss_FFC_Elem_Educ | . |
| LTD_Iss_FFC_Other_Educ | . |
| LTD_Iss_FFC_Other_NEC | . |
| Total_LTD_Iss_NG | . |
| LTD_Iss_NG_Utility | . |
| LTD_Iss_NG_Water_Util | . |
| LTD_Iss_NG_Elec_Util | . |
| LTD_Iss_NG_Gas_Util | . |
| LTD_Iss_NG_Trans_Util | . |
| LTD_Iss_NG_General | . |
| LTD_Iss_NG_Elem_Educ | . |
| LTD_Iss_NG_Other_Educ | . |
| LTD_Iss_NG_Private_Purp | . |
| LTD_Iss_NG_Other_NEC | . |
| Total_LTD_Iss_Unsp | . |
| LTD_Iss_Unsp_Utility | . |
| LTD_Iss_Unsp_Water_Util | . |
| LTD_Iss_Unsp_Elec_Util | . |
| LTD_Iss_Unsp_Gas_Util | . |
| LTD_Iss_Unsp_Trans_Util | . |
| LTD_Iss_Unsp_General | . |
| LTD_Iss_Unsp_Elem_Educ | . |
| LTD_Iss_Unsp_Other_Educ | . |
| LTD_Iss_Unsp_Other_NEC | . |
| Total_LTD_Retired | _34T, _39U |
| LTD_Ret_Private_Purp | _34T |
| LTD_Ret_All_Other | _39U |
| LTD_Ret_Utility | . |
| LTD_Ret_Util_Water | . |
| LTD_Ret_Util_Electric | . |
| LTD_Ret_Util_Gas_Supply | . |
| LTD_Ret_Util_Transit | . |
| LTD_Ret_General | . |
| LTD_Ret_Gen_Elem_Educ | . |
| LTD_Ret_Gen_Other_Educ | . |
| LTD_Ret_Gen_Other_NEC | . |
| Total_LTD_Ret_FFC | . |
| LTD_Ret_FFC_Utility | . |
| LTD_Ret_FFC_Water_Util | . |
| LTD_Ret_FFC_Elec_Util | . |
| LTD_Ret_FFC_Gas_Util | . |
| LTD_Ret_FFC_Trans_Util | . |
| LTD_Ret_FFC_General | . |
| LTD_Ret_FFC_Elem_Educ | . |
| LTD_Ret_FFC_Other_Educ | . |
| LTD_Ret_FFC_Other_NEC | . |
| Total_LTD_Ret_NG | . |
| LTD_Ret_NG_Utility | . |
| LTD_Ret_NG_Water_Util | . |
| LTD_Ret_NG_Elec_Util | . |
| LTD_Ret_NG_Gas_Util | . |
| LTD_Ret_NG_Trans_Util | . |
| LTD_Ret_NG_General | . |
| LTD_Ret_NG_Elem_Educ | . |
| LTD_Ret_NG_Other_Educ | . |
| LTD_Ret_NG_Private_Purp | . |
| LTD_Ret_NG_Other_NEC | . |
| Total_LTD_Ret_Unsp | . |
| LTD_Ret_Unsp_Utility | . |
| LTD_Ret_Unsp_Water_Util | . |
| LTD_Ret_Unsp_Elec_Utili | . |
| LTD_Ret_Unsp_Gas_Util | . |
| LTD_Ret_Unsp_Trans_Util | . |
| LTD_Ret_Unsp_General | . |
| LTD_Ret_Unsp_Elem_Educ | . |
| LTD_Ret_Unsp_Other_Educ | . |
| LTD_Ret_Unsp_Other_NEC | . |
| Total_LTD_Out | _44T, _49U |
| LTD_Out_Private_Purp | _44T |
| LTD_Out_All_Other | _49U |
| Total_LTD_Out_Utility | . |
| LTD_Out_Util_Water | . |
| LTD_Out_Util_Electric | . |
| LTD_Out_Util_Gas_Supply | . |
| LTD_Out_Util_Transit | . |
| LTD_Out_General | . |
| LTD_Out_Gen_Elem_Educ | . |
| LTD_Out_Gen_Other_Educ | . |
| LTD_Out_Gen_Other_NEC | . |
| Total_LTD_Out_FFC | . |
| LTD_Out_FFC_Utility | . |
| LTD_Out_FFC_Water_Util | . |
| LTD_Out_FFC_Elec_Util | . |
| LTD_Out_FFC_Gas_Util | . |
| LTD_Out_FFC_Trans_Util | . |
| LTD_Out_FFC_General | . |
| LTD_Out_FFC_Elem_Educ | . |
| LTD_Out_FFC_Other_Educ | . |
| LTD_Out_FFC_Other_NEC | . |
| Tot_LTD_Out_NG | . |
| LTD_Out_NG_Utility | . |
| LTD_Out_NG_Water_Util | . |
| LTD_Out_NG_Elec_Util | . |
| LTD_Out_NG_Gas_Util | . |
| LTD_Out_NG_Trans_Util | . |
| LTD_Out_NG_General | . |
| LTD_Out_NG_Elem_Educ | . |
| LTD_Out_NG_Other_Educ | . |
| LTD_Out_NG_Private_Purp | . |
| LTD_Out_NG_Other_NEC | . |
| Total_Cash___Securities | W01, W31, W61, X21, X30, Z77, Z78, X42, X44, X47, Y07, Y08, Y21, Y61 |
| Insur_Trust_Cash___Sec | X21, X30, Z77, Z78, X42, X44, X47, Y07, Y08, Y21, Y61 |
| Emp_Retire_Cash___Sec | X21, X30, X35, Z77, Z78, X42, X47, X44 |
| Emp_Retire_Cash___Dep | X21 |
| Emp_Retire_Total_Sec | X30, X35, Z77, Z78, X42, X47, X44 |
| Emp_Retire_Sec_Tot_Fed | X30 |
| Emp_Retire_Sec_S_L_Secur | X35 |
| Emp_Retire_Sec_Tot_Nong | Z77, Z78, X42, X47, X44 |
| Emp_Retire_Sec_Corp_Bds | Z77 |
| Emp_Retire_Sec_Corp_Stk | Z78 |
| Emp_Retire_Sec_Mortgages | X42 |
| Emp_Retire_Sec_Misc_Inv | X47 |
| Emp_Retire_Sec_Oth_Nong | X44 |
| Unemp_Comp_Cash___Sec | Y07, Y08 |
| Unemp_Comp_Bal_In_US_Trs | Y07 |
| Unemp_Comp_Other_Balance | Y08 |
| Nonin_Trust_Cash___Sec | W01, W31, W61 |
| Sinking_Fd_Cash___Sec | W01 |
| Bond_Fd_Cash___Sec | W31 |
| Oth_Nonin_Fd_Cash___Sec | W61 |

Appendix C: Sas Code for Data Consolidation

The following is the complete SAS code we used to consolidate all of the files we received from the census. Please refer to appendix A for step by step instructions for using this SAS code.

* This sas macro merges the historical data files that the census provides;

**%macro** ***census***;

* Defines the text that is repeated in each census file. Base text should be changed to your correct file path (it is declared twice in the code, so change both);

%LET BaseText = E:/CensusData/IndFin/IndFin0;

%LET EndTextA = a.Txt;

%LET EndTextB = b.Txt;

%LET EndTextC = c.Txt;

%do i = **7** %to **0** %by -**1**;

* Import the data from each of the three physical files in the current year;

%Let FileName = &BaseText&i&EndTextA; *Creates concatenated file names following the census naming conventions;

proc import datafile="&FileName" out=DataA dbms=dlm replace;

delimiter=",";

getnames=yes;

GUESSINGROWS=**700**; *This takes longer to run than is optimal, but will help to ensure that the data types created by proc import are correct.;

run;

%Let FileName = &BaseText&i&EndTextB;

proc import datafile="&FileName" out=DataB dbms=dlm replace;

delimiter=",";

getnames=yes;

run;

%Let FileName = &BaseText&i&EndTextC;

proc import datafile="&FileName" out=DataC dbms=dlm replace;

delimiter=",";

getnames=yes;

run;

* Merge the three data items into one;

Data LatestData;

Merge DataA DataB DataC;

by ID;

run;

* The if then else structure allows the larger data set to be created for the first set of files;

%if &i = **7** %then

%do;

* Add trailing zeros to the ID number to match the format in more recent data;

Data LargeData;

set LatestData;

run;

%end;

%else

%do;

* Append the most recently merged data to the larger data file;

PROC APPEND BASE= LargeData DATA= LatestData force;

RUN;

%end;

%end;

%LET BaseText = E:/CensusData/IndFin/IndFin; * Make sure to change this destination to match your file system;

%do i = **70** %to **99**;

* Import the data from each of the three physical files in the current year;

%Let FileName = &BaseText&i&EndTextA; *Creates concatenated file names following the census naming conventions;

proc import datafile="&FileName" out=DataA dbms=dlm replace;

delimiter=",";

getnames=yes;

GUESSINGROWS=**700**;

run;

%Let FileName = &BaseText&i&EndTextB;

proc import datafile="&FileName" out=DataB dbms=dlm replace;

delimiter=",";

getnames=yes;

run;

%Let FileName = &BaseText&i&EndTextC;

proc import datafile="&FileName" out=DataC dbms=dlm replace;

delimiter=",";

getnames=yes;

run;

* Merge the three data items into one;

Data LatestData;

Merge DataA DataB DataC;

by ID;

run;

* Append the most recently merged data to the larger data file;

PROC APPEND BASE= LargeData DATA= LatestData force;

RUN;

%end;

%do i = **67** %to **67**;

* Import the data from each of the three physical files in the current year;

%Let FileName = &BaseText&i&EndTextA; *Creates concatenated file names following the census naming conventions;

proc import datafile="&FileName" out=DataA dbms=dlm replace;

delimiter=",";

getnames=yes;

GUESSINGROWS=**700**;

run;

%Let FileName = &BaseText&i&EndTextB;

proc import datafile="&FileName" out=DataB dbms=dlm replace;

delimiter=",";

getnames=yes;

run;

%Let FileName = &BaseText&i&EndTextC;

proc import datafile="&FileName" out=DataC dbms=dlm replace;

delimiter=",";

getnames=yes;

run;

* Merge the three data items into one;

Data LatestData;

Merge DataA DataB DataC;

by ID;

run;

* Append the most recently merged data to the larger data file;

PROC APPEND BASE= LargeData DATA= LatestData force;

RUN;

%end;

**%MEND** Census;

* This line runs the macro shown above;

%***Census***;

*Create variables not existing in earlier data that can be calculated from it;

**Data** LargeData;

Set LargeData;

*Manipulate certain identification variables that can be improved in the overall database;

FunctionCode = **.**;

Enrollment = **.**;

if Type_Code = '4' then

do;

FunctionCode = Population;

Population = **.**;

Enrollment = **.**;

end;

if Type_Code = '5' then

do;

FunctionCode = **.**;

Enrollment = Population;

Population = **.**;

end;

**run**;

* We now need to format the newer census data to roughly match the format of the earlier data. This process is;

* accomplished without a macro because there are only a few years of newer data, and the file names thend to be non-standard;

* Make sure that you change the "Inflie" line on each of the data steps to the path for the files on your system.

* Read the financial data file in fixed width format, dollar signs indicate values which are stored as characters;

**DATA** FinancialData;

INFILE 'E:/CensusData/2011/2011FinEstDAT_ALL5modp_pu.txt'

MISSOVER;

INPUT id_state $ **1**-**2** id_type $ **3** id_county $ **4**-**6** id_unit $ **7**-**9** id_add $ **10**-**14** itemcode $ **15**-**17** data **18**-**29** year **30**-**33** code $ **34**;

id = cats(of id_state id_type id_county id_unit);

**RUN**;

* Transpose the financial information file so that every government ID number corresponds to one row and every data item has its own column;

**PROC** **TRANSPOSE** data=work.FinancialData

out=work.TransposedData

name=Year;

var data;

by id;

id itemcode;

**run**;

* Change the "year" variable in the finacnial data matrix to be the year we are currently working with;

**Data** TransposedDates;

Set work.TransposedData;

Year = **2011**;

**run**;

* Sort each of the matricies by ID code in preparation for merging them.;

**proc** **sort** data=TransposedDates;

by id;

**run**;

* Read the government information file as a fixed width text file. Dollar signs indicate fields that are coded as text.;

**DATA** Identification;

INFILE 'E:/CensusData/2011/fin_gid_2011.txt'

MISSOVER;

INPUT id_state $ **1**-**2** id_type $ **3** id_county $ **4**-**6** id_unit $ **7**-**9** id_add $ **10**-**14** Name $ **15**-**78** CountyName $ **79**-**113** FIPSstate $ **114**-**115** FIPScounty $ **116**-**118** FIPSplace $ **119**-**123** Population **124**-**132** PopYear $ **133**-**134** Enrollment **135**-**141** EnrollYear $ **142**-**143** FunctionCode **144**-**145** SchoolLevel $ **146**-**147** FYEnd $ **148**-**151** SurveyYear $ **152**-**153**;

id = cats(of id_state id_type id_county id_unit);

**RUN**;

* Merge the government identification file with the transposed, dated, finacial information file;

**Data** LatestData;

Merge Identification TransposedDates;

By id;

**run**;

*Concatenate the Latest data to the larger data file preserving every data element for later reorganization;

**Data** NewData;

Set LatestData;

**Run**;

*Begin the same process for the 2010 data;

*

*

*

*;

* Read the financial data file in fixed width format, dollar signs indicate values which are stored as characters;

**DATA** FinancialData;

INFILE 'E:/CensusData/2010/2010FinEstDAT_ALL12modp_pu.txt'

MISSOVER;

INPUT id_state $ **1**-**2** id_type $ **3** id_county $ **4**-**6** id_unit $ **7**-**9** id_add $ **10**-**14** itemcode $ **15**-**17** data **18**-**29** year **30**-**33** code $ **34**;

id = cats(of id_state id_type id_county id_unit);

**RUN**;

* Transpose the financial information file so that every government ID number corresponds to one row and every data item has its own column;

**PROC** **TRANSPOSE** data=work.FinancialData

out=work.TransposedData

name=Year;

var data;

by id;

id itemcode;

**run**;

* Change the "year" variable in the finacnial data matrix to be the year we are currently working with;

**Data** TransposedDates;

Set work.TransposedData;

Year = **2010**;

**run**;

* Sort each of the matricies by ID code in preparation for merging them.;

**proc** **sort** data=TransposedDates;

by id;

**run**;

* Read the government information file as a fixed width text file. Dollar signs indicate fields that are coded as text.;

**DATA** Identification;

INFILE 'E:/CensusData/2010/fin_gid_2010.txt'

MISSOVER;

INPUT id_state $ **1**-**2** id_type $ **3** id_county $ **4**-**6** id_unit $ **7**-**9** id_add $ **10**-**14** Name $ **15**-**78** CountyName $ **79**-**113** FIPSstate $ **114**-**115** FIPScounty $ **116**-**118** FIPSplace $ **119**-**123** Population **124**-**132** PopYear $ **133**-**134** Enrollment **135**-**141** EnrollYear $ **142**-**143** FunctionCode **144**-**145** SchoolLevel $ **146**-**147** FYEnd $ **148**-**151** SurveyYear $ **152**-**153**;

id = cats(of id_state id_type id_county id_unit);

**RUN**;

* Merge the government identification file with the transposed, dated, finacial information file;

**Data** LatestData;

Merge Identification TransposedDates;

By id;

**run**;

*Concatenate the Latest data to the larger data file preserving every data element for later reorganization;

**Data** NewData;

Set NewData LatestData;

**Run**;

*Begin the same process for the 2009 data;

*

*

*

*;

* Read the financial data file in fixed width format, dollar signs indicate values which are stored as characters;

**DATA** FinancialData;

INFILE 'E:/CensusData/2009/2009FinEstDAT_ALL16modp_pu.txt'

MISSOVER;

INPUT id_state $ **1**-**2** id_type $ **3** id_county $ **4**-**6** id_unit $ **7**-**9** id_add $ **10**-**14** itemcode $ **15**-**17** data **18**-**29** year **30**-**33** code $ **34**;

id = cats(of id_state id_type id_county id_unit);

**RUN**;

* Transpose the financial information file so that every government ID number corresponds to one row and every data item has its own column;

**PROC** **TRANSPOSE** data=work.FinancialData

out=work.TransposedData

name=Year;

var data;

by id;

id itemcode;

**run**;

* Change the "year" variable in the finacnial data matrix to be the year we are currently working with;

**Data** TransposedDates;

Set work.TransposedData;

Year = **2009**;

**run**;

* Sort each of the matricies by ID code in preparation for merging them.;

**proc** **sort** data=TransposedDates;

by id;

**run**;

* Read the government information file as a fixed width text file. Dollar signs indicate fields that are coded as text.;

**DATA** Identification;

INFILE 'E:/CensusData/2009/fin_gid_2009.txt'

MISSOVER;

INPUT id_state $ **1**-**2** id_type $ **3** id_county $ **4**-**6** id_unit $ **7**-**9** id_add $ **10**-**14** Name $ **15**-**78** CountyName $ **79**-**113** FIPSstate $ **114**-**115** FIPScounty $ **116**-**118** FIPSplace $ **119**-**123** Population **124**-**132** PopYear $ **133**-**134** Enrollment **135**-**141** EnrollYear $ **142**-**143** FunctionCode **144**-**145** SchoolLevel $ **146**-**147** FYEnd $ **148**-**151** SurveyYear $ **152**-**153**;

id = cats(of id_state id_type id_county id_unit);

**RUN**;

* Merge the government identification file with the transposed, dated, finacial information file;

**Data** LatestData;

Merge Identification TransposedDates;

By id;

**run**;

*Concatenate the Latest data to the larger data file preserving every data element for later reorganization;

**Data** NewData;

Set NewData LatestData;

**Run**;

*Begin the same process for the 2008 data;

*

*

*

*;

* Read the financial data file in fixed width format, dollar signs indicate values which are stored as characters;

**DATA** FinancialData;

INFILE 'E:/CensusData/2008/2008FinInddiv15_modp3.txt'

MISSOVER;

INPUT id_state $ **1**-**2** id_type $ **3** id_county $ **4**-**6** id_unit $ **7**-**9** id_add $ **10**-**14** itemcode $ **15**-**17** data **18**-**29** year **30**-**33** code $ **34**;

id = cats(of id_state id_type id_county id_unit);

**RUN**;

* Transpose the financial information file so that every government ID number corresponds to one row and every data item has its own column;

**PROC** **TRANSPOSE** data=work.FinancialData LET

out=work.TransposedData

name=Year;

var data;

by id;

id itemcode;

**run**;

* Change the "year" variable in the finacnial data matrix to be the year we are currently working with;

**Data** TransposedDates;

Set work.TransposedData;

Year = **2008**;

**run**;

* Sort each of the matricies by ID code in preparation for merging them.;

**proc** **sort** data=TransposedDates;

by id;

**run**;

* Read the government information file as a fixed width text file. Dollar signs indicate fields that are coded as text.;

**DATA** Identification;

INFILE 'E:/CensusData/2008/fin_gid_2008.txt'

MISSOVER;

INPUT id_state $ **1**-**2** id_type $ **3** id_county $ **4**-**6** id_unit $ **7**-**9** id_add $ **10**-**14** Name $ **15**-**78** CountyName $ **79**-**113** FIPSstate $ **114**-**115** FIPScounty $ **116**-**118** FIPSplace $ **119**-**123** Population **124**-**132** PopYear $ **133**-**134** Enrollment **135**-**141** EnrollYear $ **142**-**143** FunctionCode **144**-**145** SchoolLevel $ **146**-**147** FYEnd $ **148**-**151** SurveyYear $ **152**-**153**;

id = cats(of id_state id_type id_county id_unit);

**RUN**;

* Merge the government identification file with the transposed, dated, finacial information file;

**Data** LatestData;

Merge Identification TransposedDates;

By id;

**run**;

*Concatenate the Latest data to the larger data file preserving every data element for later reorganization;

**Data** NewData;

Set NewData LatestData;

**Run**;

*

*

*

*

*Manually convert the newer data to the format of the older data and keep only variables that exist in the final data set;

**Data** ConvertedNewData (keep= SurveyYr Year4 ID State_Code Type_Code County Name FIPS_Code_State FYEndDate YearPop SchLevCode Population Total_Revenue Total_Rev_Own_Sources General_Revenue Gen_Rev_Own_Sources Total_Taxes Property_Tax Tot_Sales___Gr_Rec_Tax Total_Gen_Sales_Tax Total_Select_Sales_Tax Alcoholic_Beverage_Tax Amusement_Tax Insurance_Premium_Tax Motor_Fuels_Tax Pari_mutuels_Tax Public_Utility_Tax Tobacco_Tax Other_Select_Sales_Tax Total_License_Taxes Alcoholic_Beverage_Lic

Amusement_License Corporation_License Hunting___Fishing_License Motor_Vehicle_License Motor_Veh_Oper_License Public_Utility_License Occup_and_Bus_Lic_NEC Other_License_Taxes Total_Income_Taxes Individual_Income_Tax Corp_Net_Income_Tax Death_and_Gift_Tax Docum_and_Stock_Tr_Tax Severance_Tax Taxes_NEC Total_IG_Revenue Total_Fed_IG_Revenue Fed_IGR_Air_Transport Fed_IGR_Education Fed_IGR_Emp_Sec_Adm Fed_IGR_Gen_Rev_Shar Fed_IGR_Gen_Support Fed_IGR_Health___Hos Fed_IGR_Highways

Fed_IGR_Transit_Sub Fed_IGR_Hous_Com_Dev Fed_IGR_Natural_Res Fed_IGR_Public_Welf Fed_IGR_Sewerage Fed_IGR_Other Total_State_IG_Revenue State_IGR_Education State_IGR_Tax_Relief State_IGR_Oth_Gen_Sup State_IGR_Health___Hos State_IGR_Highways State_IGR_Transit_Sub State_IGR_Hous_Com_Dev State_IGR_Public_Welf State_IGR_Sewerage State_IGR_Other Tot_Local_IG_Rev Local_IGR_InterSchool_Aid Local_IGR_Other_Education Local_IGR_Oth_Gen_Sup Local_IGR_Health___Hos Local_IGR_Highways

Local_IGR_Transit_Sub Local_IGR_Hous_Com_Dev Local_IGR_Public_Welf Local_IGR_Sewerage Local_IGR_Other Tot_Chgs_and_Misc_Rev Total_General_Charges Chg_Air_Transportation Chg_Misc_Com_Activ Chg_Total_Education Chg_Elem_Ed_Sch_Lunch Chg_Elem_Ed_Tuition Chg_Elem_Ed_NEC Chg_Total_High_Ed Chg_Hospitals Chg_Regular_Highways Chg_Toll_Highways Chg_Housing___Comm_Dev Chg_Total_Nat_Res Chg_Parking Chg_Parks___Recreation Chg_Sewerage Chg_Solid_Waste_Mgmt

Chg_Water_Transport Chg_All_Other_NEC Misc_General_Revenue Special_Assessments Prop_Sale_Hous_Com_Dev Prop_Sale_Other Interest_Revenue Fines_and_Forfeits Rents_and_Royalties Net_Lottery_Revenue Misc_General_Rev_NEC Liquor_Stores_Revenue Total_Utility_Revenue Water_Utility_Revenue Electric_Utility_Rev Gas_Utility_Rev Transit_Utility_Rev Total_Insur_Trust_Rev Total_Insur_Trust_Ctrb Tot_Ins_Trust_Inv_Rev Total_Emp_Ret_Rev

Emp_Ret_Total_Ctrib Emp_Ret_Loc_Emp_Ctrib Emp_Ret_Loc_To_Loc_Sys Emp_Ret_From_Other_Gov Emp_Ret_Sta_To_Sta_Ctr Emp_Ret_Int_Rev Emp_Ret_Other_Earnings Total_Unemp_Rev Unemp_Payroll_Tax Unemp_Int_Revenue Unemp_Federal_Advances Total_Expenditure Total_IG_Expenditure Direct_Expenditure Total_Current_Expend Total_Current_Oper Total_Capital_Outlays Total_Construction Tot_Assist___Subsidies Total_Interest_on_Debt Total_Insur_Trust_Ben Total_Salaries___Wages

General_Expenditure IG_Exp_To_State_Govt IG_Exp_To_Local_Govts IG_Exp_To_Federal_Govt Direct_General_Expend General_Current_Expend General_Current_Oper General_Capital_Outlay General_Construction General_Assist___Sub General_Debt_Interest Air_Trans_Total_Expend Air_Trans_Direct_Expend Air_Trans_Cap_Outlay Air_Trans_Construction Air_Trans_IG_To_State Air_Trans_IG_Local_Govts Misc_Com_Activ_Tot_Exp Misc_Com_Activ_Cap_Out Misc_Com_Activ_Constr

Correct_Total_Exp Correct_Direct_Exp Correct_Cap_Outlay Correct_Construct Correct_IG_To_St Correct_IG_Loc_Govts Total_Educ_Total_Exp Total_Educ_Direct_Exp Total_Educ_Assist___Sub Total_Educ_Cap_Outlay Total_Educ_Construct Elem_Educ_Total_Exp Elem_Educ_Direct_Exp Elem_Educ_Cap_Outlay Elem_Educ_Construction Elem_Educ_IG_To_State Elem_Educ_IG_Local_Govts Elem_Educ_IG_Sch_to_Sch Higher_Ed_Total_Exp Higher_Ed_Direct_Exp Higher_Ed_Cap_Outlay

Higher_Ed_Construct Higher_Ed_IG_To_St Higher_Ed_IG_Loc_Govts Educ_NEC_Total_Expend Educ_NEC_Direct_Expend Educ_NEC_Assistance Educ_NEC_Cap_Outlay Educ_NEC_Construction Educ_NEC_IG_To_State Educ_NEC_IG_Local_Govts Emp_Sec_Adm_Direct_Exp Emp_Sec_Adm_Cap_Outlay Emp_Sec_Adm_Construct Fin_Admin_Total_Exp Fin_Admin_Direct_Exp Fin_Admin_Cap_Outlay Fin_Admin_Construction Fin_Admin_IG_To_State Fin_Admin_IG_Local_Govts Fire_Prot_Total_Expend Fire_Prot_Direct_Exp

Fire_Prot_Cap_Outlay Fire_Prot_Construction Fire_Prot_IG_To_State Fire_Prot_IG_Local_Govts Judicial_Total_Expend Judicial_Direct_Expend Judicial_Cap_Outlay Judicial_Construction Judicial_IG_To_State Judicial_IG_Local_Govts Cen_Staff_Total_Expend Cen_Staff_Direct_Exp Cen_Staff_Cap_Outlay Cen_Staff_Construction Cen_Staff_IG_To_State Cen_Staff_IG_Local_Govts Gen_Pub_Bldg_Total_Exp Gen_Pub_Bldg_Cap_Out Gen_Pub_Bldg_Construct

Health_Total_Expend Health_Direct_Expend Health_Capital_Outlay Health_Construction Health_IG_To_State Health_IG_Local_Govts Total_Hospital_Total_Exp Total_Hospital_Dir_Exp Total_Hospital_Cap_Out Total_Hospital_Construct Total_Hospital_IG_To_State Total_Hospital_IG_Loc_Govts Own_Hospital_Total_Exp Own_Hospital_Cap_Out Own_Hospital_Construct Hosp_Other_Total_Exp Hosp_Other_Direct_Exp Hosp_Other_Cap_Outlay Hosp_Other_Construct Hosp_Other_IG_To_State

Hosp_Other_IG_Loc_Govts Total_Highways_Tot_Exp Total_Highways_Dir_Exp Total_Highways_Cap_Out Total_Highways_Construct Regular_Hwy_Total_Exp Regular_Hwy_Direct_Exp Regular_Hwy_Cap_Outlay Regular_Hwy_Construct Regular_Hwy_IG_To_Sta Regular_Hwy_IG_Loc_Govts Toll_Hwy_Total_Expend Toll_Hwy_Cap_Outlay Toll_Hwy_Construction Transit_Sub_Total_Exp Transit_Sub_Direct_Sub Transit_Sub_IG_To_Sta Transit_Sub_IG_Loc_Govts Transit_Sub_To_Own_Sys Hous___Com_Total_Exp

Hous___Com_Direct_Exp Hous___Com_Cap_Outlay Hous___Com_Construct Hous___Com_IG_To_State Hous___Com_IG_Loc_Govts Libraries_Total_Expend Libraries_Direct_Exp Libraries_Cap_Outlay Libraries_Construction Libraries_IG_To_State Libraries_IG_Local_Govts Natural_Res_Total_Exp Natural_Res_Direct_Exp Natural_Res_Cap_Outlay Natural_Res_Construct Natural_Res_IG_To_Sta Natural_Res_IG_Loc_Govts Parking_Total_Expend Parking_Direct_Expend Parking_Capital_Outlay

Parking_Construction Parking_IG_To_State Parking_IG_Local_Govts Parks___Rec_Total_Exp Parks___Rec_Direct_Exp Parks___Rec_Cap_Outlay Parks___Rec_Construct Parks___Rec_IG_To_Sta Parks___Rec_IG_Loc_Govts Police_Prot_Total_Exp Police_Prot_Direct_Exp Police_Prot_Cap_Outlay Police_Prot_Construct Police_Prot_IG_To_Sta Police_Prot_IG_Loc_Govts Prot_Insp_Total_Exp Prot_Insp_Direct_Exp Prot_Insp_Cap_Outlay Prot_Insp_Construction Prot_Insp_IG_To_State

Prot_Insp_IG_Local_Govts Public_Welf_Total_Exp Public_Welf_Direct_Exp Public_Welf_Cash_Asst Public_Welf_Cap_Outlay Public_Welf_Construct Welf_Categ_Total_Exp Welf_Categ_Cash_Assist Welf_Categ_IG_To_State Welf_Categ_IG_Loc_Govts Welf_Cash_Total_Exp Welf_Cash_Cash_Assist Welf_Cash_IG_Local_Govts Welf_Vend_Pmts_Medical Welf_Vend_Pmts_NEC Welf_State_Share_Part_D Welf_Ins_Total_Exp Welf_Ins_Cap_Outlay Welf_Ins_Construction Welf_NEC_Total_Expend

Welf_NEC_Direct_Expend Welf_NEC_Cap_Outlay Welf_NEC_Construction Welf_NEC_IG_To_State Welf_NEC_IG_Local_Govts Sewerage_Total_Expend Sewerage_Direct_Expend Sewerage_Cap_Outlay Sewerage_Construction Sewerage_IG_To_State Sewerage_IG_Local_Govts SW_Mgmt_Total_Expend SW_Mgmt_Direct_Expend SW_Mgmt_Capital_Outlay SW_Mgmt_Construction SW_Mgmt_IG_To_State SW_Mgmt_IG_Local_Govts Water_Trans_Total_Exp Water_Trans_Direct_Exp Water_Trans_Cap_Outlay Water_Trans_Construct

Water_Trans_IG_To_Sta Water_Trans_IG_Loc_Govts Interest_on_Gen_Debt General_NEC_Total_Exp General_NEC_Direct_Exp VetBonus General_NEC_Cap_Outlay General_NEC_Construct General_NEC_IG_To_St General_NEC_IG_Loc_Govts General_NEC_IG_To_Fed Liquor_Stores_Tot_Exp Liquor_Stores_Cap_Out Liquor_Stores_Constr Total_Util_Total_Exp Total_Util_Inter_Exp Total_Util_Cap_Outlay Total_Util_Construct Water_Util_Total_Exp Water_Util_Inter_Exp

Water_Util_Cap_Outlay Water_Util_Construct Elec_Util_Total_Exp Elec_Util_Inter_Exp Elec_Util_Cap_Outlay Elec_Util_Construct Gas_Util_Total_Exp Gas_Util_Inter_Exp Gas_Util_Cap_Outlay Gas_Util_Construct Trans_Util_Total_Exp Trans_Util_Inter_Exp Trans_Util_Cap_Outlay Trans_Util_Construct Emp_Ret_Total_Expend Emp_Ret_Benefit_Paymts Emp_Ret_Withdrawals Emp_Ret_Other_Paymts Unemp_Comp_Total_Exp Unemp_Comp_Ben_Paymts Unemp_Ext___Spec_Pmts

Total_Debt_Outstanding Total_Long_Term_Debt_Out ST_Debt_End_of_Year Total_Beg_LTD_Out Beg_LTD_Out_Private_Purp Beg_LTD_Out_All_Other Beg_LTD_Out_Utility Beg_LTD_Out_Water_Util Beg_LTD_Out_Elec_Util Beg_LTD_Out_Gas_Util Beg_LTD_Out_Trans_Util Beg_LTD_Out_General Beg_LTD_Out_Education Beg_LTD_Out_Priv_Purp Beg_LTD_Out_Other_NEC Total_LTD_Issued LTD_Iss_Private_Purp LTD_Iss_All_Other LTD_Iss_Utility LTD_Iss_Util_Water LTD_Iss_Util_Electric

LTD_Iss_Util_Gas_Supply LTD_Iss_Util_Transit LTD_Iss_General LTD_Iss_Gen_Elem_Educ LTD_Iss_Gen_Other_Educ LTD_Iss_Gen_Other_NEC Total_LTD_Iss_FFC LTD_Iss_FFC_Utility LTD_Iss_FFC_Water_Util LTD_Iss_FFC_Elec_Util LTD_Iss_FFC_Gas_Util LTD_Iss_FFC_Trans_Util LTD_Iss_FFC_General LTD_Iss_FFC_Elem_Educ LTD_Iss_FFC_Other_Educ LTD_Iss_FFC_Other_NEC Total_LTD_Iss_NG LTD_Iss_NG_Utility LTD_Iss_NG_Water_Util LTD_Iss_NG_Elec_Util LTD_Iss_NG_Gas_Util

LTD_Iss_NG_Trans_Util LTD_Iss_NG_General LTD_Iss_NG_Elem_Educ LTD_Iss_NG_Other_Educ LTD_Iss_NG_Private_Purp LTD_Iss_NG_Other_NEC Total_LTD_Iss_Unsp LTD_Iss_Unsp_Utility LTD_Iss_Unsp_Water_Util LTD_Iss_Unsp_Elec_Util LTD_Iss_Unsp_Gas_Util LTD_Iss_Unsp_Trans_Util LTD_Iss_Unsp_General LTD_Iss_Unsp_Elem_Educ LTD_Iss_Unsp_Other_Educ LTD_Iss_Unsp_Other_NEC Total_LTD_Retired LTD_Ret_Private_Purp LTD_Ret_All_Other LTD_Ret_Utility LTD_Ret_Util_Water

LTD_Ret_Util_Electric LTD_Ret_Util_Gas_Supply LTD_Ret_Util_Transit LTD_Ret_General LTD_Ret_Gen_Elem_Educ LTD_Ret_Gen_Other_Educ LTD_Ret_Gen_Other_NEC Total_LTD_Ret_FFC LTD_Ret_FFC_Utility LTD_Ret_FFC_Water_Util LTD_Ret_FFC_Elec_Util LTD_Ret_FFC_Gas_Util LTD_Ret_FFC_Trans_Util LTD_Ret_FFC_General LTD_Ret_FFC_Elem_Educ LTD_Ret_FFC_Other_Educ LTD_Ret_FFC_Other_NEC Total_LTD_Ret_NG LTD_Ret_NG_Utility LTD_Ret_NG_Water_Util LTD_Ret_NG_Elec_Util

LTD_Ret_NG_Gas_Util LTD_Ret_NG_Trans_Util LTD_Ret_NG_General LTD_Ret_NG_Elem_Educ LTD_Ret_NG_Other_Educ LTD_Ret_NG_Private_Purp LTD_Ret_NG_Other_NEC Total_LTD_Ret_Unsp LTD_Ret_Unsp_Utility LTD_Ret_Unsp_Water_Util LTD_Ret_Unsp_Elec_Utili LTD_Ret_Unsp_Gas_Util LTD_Ret_Unsp_Trans_Util LTD_Ret_Unsp_General LTD_Ret_Unsp_Elem_Educ LTD_Ret_Unsp_Other_Educ LTD_Ret_Unsp_Other_NEC Total_LTD_Out LTD_Out_Private_Purp LTD_Out_All_Other Total_LTD_Out_Utility

LTD_Out_Util_Water LTD_Out_Util_Electric LTD_Out_Util_Gas_Supply LTD_Out_Util_Transit LTD_Out_General LTD_Out_Gen_Elem_Educ LTD_Out_Gen_Other_Educ LTD_Out_Gen_Other_NEC Total_LTD_Out_FFC LTD_Out_FFC_Utility LTD_Out_FFC_Water_Util LTD_Out_FFC_Elec_Util LTD_Out_FFC_Gas_Util LTD_Out_FFC_Trans_Util LTD_Out_FFC_General LTD_Out_FFC_Elem_Educ LTD_Out_FFC_Other_Educ LTD_Out_FFC_Other_NEC Tot_LTD_Out_NG LTD_Out_NG_Utility LTD_Out_NG_Water_Util

LTD_Out_NG_Elec_Util LTD_Out_NG_Gas_Util LTD_Out_NG_Trans_Util LTD_Out_NG_General LTD_Out_NG_Elem_Educ LTD_Out_NG_Other_Educ LTD_Out_NG_Private_Purp LTD_Out_NG_Other_NEC Total_Cash___Securities Insur_Trust_Cash___Sec Emp_Retire_Cash___Sec Emp_Retire_Cash___Dep Emp_Retire_Total_Sec Emp_Retire_Sec_Tot_Fed Emp_Retire_Sec_S_L_Secur Emp_Retire_Sec_Tot_Nong Emp_Retire_Sec_Corp_Bds Emp_Retire_Sec_Corp_Stk Emp_Retire_Sec_Mortgages

Emp_Retire_Sec_Misc_Inv Emp_Retire_Sec_Oth_Nong Unemp_Comp_Cash___Sec Unemp_Comp_Bal_In_US_Trs Unemp_Comp_Other_Balance Nonin_Trust_Cash___Sec Sinking_Fd_Cash___Sec Bond_Fd_Cash___Sec Oth_Nonin_Fd_Cash___Sec FunctionCode Enrollment);

set NewData;

* Convert variables to ones that exist in the earlier dataset. Multiplying by 1 converts character variables to numeric formats;

SurveyYr = SurveyYear***1**;

Year4 = Year***1**;

State_Code = id_state;

Type_Code = id_type;

County = id_County;

FIPS_Code_State = FIPSstate;

FYEndDate = FYEnd;

YearPop = PopYear;

SchLevCode = SchoolLevel;

*Consolidate and name variables;

*Revenue;

Total_Revenue = sum(B01, B21, B22, B30, B42, B46, B50, B59, B79, B80, B89, B91, B92, B93, B94, C21, C30, C42, C46, C50, C79, C80, C89, C91, C92, C93, C94, D21, D30, D42, D46, D50, D79, D80, D89, D91, D92, D93, D94, T01, T09, T10, T11, T12, T13, T14, T15, T16, T19, T20, T21, T22, T23, T24, T25, T27, T28, T29, T40, T41, T50, T51, T53, T99, A01, A03, A09, A10, A12, A16, A18, A21, A36, A44, A45, A50, A56, A59, A60, A61, A80, A81, A87, A89, U01, U11, U20, U21, U30, U40, U41, U50, U95, U99, A90, A91, A92, A93, A94, X01, X02, X05, X08, Y01, Y02, Y04, Y11, Y12, Y51, Y52);

General_Revenue = sum(B01, B21, B22, B30, B42, B46, B50, B59, B79, B80, B89, B91, B92, B93, B94, C21, C30, C42, C46, C50, C79, C80, C89, C91, C92, C93, C94, D21, D30, D42, D46, D50, D79, D80, D89, D91, D92, D93, D94, T01, T09, T10, T11, T12, T13, T14, T15, T16, T19, T20, T21, T22, T23, T24, T25, T27, T28, T29, T40, T41, T50, T51, T53, T99, A01, A03, A09, A10, A12, A16, A18, A21, A36, A44, A45, A50, A56, A59, A60, A61, A80, A81, A87, A89, U01, U11, U20, U21, U30, U40, U41, U50, U95, U99);

Gen_Rev_Own_Sources = sum(A01, A03, A09, A10, A12, A16, A18, A21, A36, A44, A45, A50, A56, A59, A60, A61, A80, A81, A87, A89, T01, T09, T10, T11, T12, T13, T14, T15, T16, T19, T20, T21, T22, T23, T24, T25, T27, T28, T29, T40, T41, T50, T51, T53, T99, U01, U11, U20, U21, U30, U40, U41, U50, U95, U99);

Total_Rev_Own_Sources = sum(T01, T09, T10, T11, T12, T13, T14, T15, T16, T19, T20, T21, T22, T23, T24, T25, T27, T28, T29, T40, T41, T50, T51, T53, T99, A01, A03, A09, A10, A12, A16, A18, A21, A36, A44, A45, A50, A56, A59, A60, A61, A80, A81, A87, A89, U01, U11, U20, U21, U30, U40, U41, U50, U95, U99, A90, A91, A92, A93, A94, X01, X02, X05, X08, Y01, Y02, Y04, Y11, Y12, Y51, Y52);

*Taxes;

Total_Taxes = sum(T01, T09, T10, T11, T12, T13, T14, T15, T16, T19, T20, T21, T22, T23, T24, T25, T27, T28, T29, T40, T41, T50, T51, T53, T99);

Property_Tax = T01;

Tot_Sales___Gr_Rec_Tax = sum(T09, T10, T11, T12, T13, T14, T15, T16, T19);

Total_Gen_Sales_Tax = T09;

Total_Select_Sales_Tax = sum(T10, T11, T12, T13, T14, T15, T16, T19);

Alcoholic_Beverage_Tax = T10;

Amusement_Tax = T11;

Insurance_Premium_Tax = T12;

Motor_Fuels_Tax = T13;

Pari_mutuels_Tax = T14;

Public_Utility_Tax = T15;

Tobacco_Tax = T16;

Other_Select_Sales_Tax = T19;

*Licenses;

Total_License_Taxes = sum(T20 , T21 , T22 , T23 , T24 , T25 , T27 , T28 , T29);

Alcoholic_Beverage_Lic = T20;

Amusement_License = T21;

Corporation_License = T22;

Hunting___Fishing_License = T23;

Motor_Vehicle_License = T24;

Motor_Veh_Oper_License = T25;

Public_Utility_License = T27;

Occup_and_Bus_Lic_NEC = T28;

Other_License_Taxes = T29;

*Income Taxes;

Total_Income_Taxes = sum(T40 , T41 , T50 , T51 , T53 , T99);

Individual_Income_Tax = T40;

Corp_Net_Income_Tax = T41;

Death_and_Gift_Tax = T50;

Docum_and_Stock_Tr_Tax = T51;

Severance_Tax = T53;

Taxes_NEC = T99;

* Intergovernmental Revenue;

Total_IG_Revenue = sum(B01, B21, B22, B30, B42, B46, B50, B59, B79, B80, B89, B91, B92, B93, B94, C21, C30, C42, C46, C50, C79, C80, C89, C91, C92, C93, C94, D21, D30, D42, D46, D50, D79, D80, D89, D91, D92, D93, D94);

* Federal;

Total_Fed_IG_Revenue = sum(B01, B21, B22, B30, B42, B46, B50, B59, B79, B80, B89, B91, B92, B93, B94);

Fed_IGR_Air_Transport = B01;

Fed_IGR_Education = B21;

Fed_IGR_Emp_Sec_Adm = B22;

Fed_IGR_Gen_Rev_Shar = **.**; *Obsolete after 1987;

Fed_IGR_Gen_Support = B30;

Fed_IGR_Health___Hos = B42;

Fed_IGR_Highways = B46;

Fed_IGR_Transit_Sub = B94;

Fed_IGR_Hous_Com_Dev = B50;

Fed_IGR_Natural_Res = B59;

Fed_IGR_Public_Welf = B79;

Fed_IGR_Sewerage = B80;

Fed_IGR_Other = B89;

* State;

Total_State_IG_Revenue = sum(C21, C30, C42, C46, C50, C79, C80, C89, C91, C92, C93, C94);

State_IGR_Education = C21;

State_IGR_Tax_Relief = **.**; *Obsolete after 1987;

State_IGR_Oth_Gen_Sup = C30;

State_IGR_Health___Hos = C42;

State_IGR_Highways = C46;

State_IGR_Transit_Sub = C94;

State_IGR_Hous_Com_Dev = C50;

State_IGR_Public_Welf = C79;

State_IGR_Sewerage = C80;

State_IGR_Other = C89;

* Local;

Tot_Local_IG_Rev = sum(D21, D30, D42, D46, D50, D79, D80, D89, D91, D92, D93, D94);

Local_IGR_InterSchool_Aid = D11;

Local_IGR_Other_Education = D21;

Local_IGR_Oth_Gen_Sup = D30;

Local_IGR_Health___Hos = D42;

Local_IGR_Highways = D46;

Local_IGR_Transit_Sub = D94;

Local_IGR_Hous_Com_Dev = D50;

Local_IGR_Public_Welf = D79;

Local_IGR_Sewerage = D80;

Local_IGR_Other = D89;

* Charges and Misc Revenue;

Tot_Chgs_and_Misc_Rev = sum(A01, A03, A09, A10, A12, A16, A18, A21, A36, A44, A45, A50, A56, A59, A60, A61, A80, A81, A87, A89, U01, U11, U20, U21, U30, U40, U41, U50, U95, U99);

* Charges;

Total_General_Charges = sum(A01, A03, A09, A10, A12, A16, A18, A21, A36, A44, A45, A50, A56, A59, A60, A61, A80, A81, A87, A89);

Chg_Air_Transportation = A01;

Chg_Misc_Com_Activ = A03;

Chg_Total_Education = sum(A09, A10, A12, A16, A18, A21);

Chg_Elem_Ed_Sch_Lunch = A09;

Chg_Elem_Ed_Tuition = A10;

Chg_Elem_Ed_NEC = A12;

Chg_Total_High_Ed = sum(A16, A18);

Chg_Hospitals = A36;

Chg_Regular_Highways = A44;

Chg_Toll_Highways = A45;

Chg_Housing___Comm_Dev = A50;

Chg_Total_Nat_Res = sum(A56 , A59);

Chg_Parking = A60;

Chg_Parks___Recreation = A61;

Chg_Sewerage = A80;

Chg_Solid_Waste_Mgmt = A81;

Chg_Water_Transport = A87;

Chg_All_Other_NEC = A89;

* Misc Revenue;

Misc_General_Revenue = sum(U01, U11, U20, U21, U30, U40, U41, U50, U95, U99);

Special_Assessments = U01;

Prop_Sale_Hous_Com_Dev = **.**; *Deleted as of 2005 and reported in U11 Prop_Sale_Other;

Prop_Sale_Other = U11;

Interest_Revenue = U20;

Fines_and_Forfeits = U30;

Rents_and_Royalties = sum(U40 , U41);

Net_Lottery_Revenue = U95;

Misc_General_Rev_NEC = U99;

* Liquor and Utilities;

Liquor_Stores_Revenue = A90;

Total_Utility_Revenue = sum(A91, A92, A93, A94);

Water_Utility_Revenue = A91;

Electric_Utility_Rev = A92;

Gas_Utility_Rev = A93;

Transit_Utility_Rev = A94;

* Insurance Trusts;

Total_Insur_Trust_Rev = sum(X01, X02, X05, X08, Y01, Y02, Y04, Y11, Y12, Y51, Y52);

Total_Insur_Trust_Ctrb = sum(X01, X02, X05, Y01);

Tot_Ins_Trust_Inv_Rev = sum(X08 , Y02);

*Retirement Plan Data;

Total_Emp_Ret_Rev = sum(X01, X02, X05, X08);

Emp_Ret_Total_Ctrib = sum(X01, X02, X05);

Emp_Ret_Loc_Emp_Ctrib = X01;

Emp_Ret_Loc_To_Loc_Sys = X04;

Emp_Ret_From_Other_Gov = X05;

Emp_Ret_Sta_To_Sta_Ctr = X06;

Emp_Ret_Int_Rev = X08;

Emp_Ret_Other_Earnings = **.**; *Consolidated with X08 Emp_Ret_Int_Rev in 1990;

* Old data does not have Worker's Comp information;

* Unemployment Revenue;

Total_Unemp_Rev = sum(Y01, Y02, Y04);

Unemp_Payroll_Tax = Y01;

Unemp_Int_Revenue = Y02;

Unemp_Federal_Advances = Y04;

*Expenses;

Total_Expenditure = sum(E01, E03, E04, E05, E12, E16, E18, E21, E22, E23, E24, E25, E26, E29, E31, E32, E36, E44, E44, E45, E50, E52, E55, E56, E59, E60, E61, E62, E66, E74, E75, E77, E79, E80, E81, E85, E87, E89, E90, E91, E92, E93, E94, I89, I91, I92, I93, I94, J19, J67, J68, J85, X11, X12, Y05, Y06, Y14, Y53, F01, F03, F04, F05, F12, F16, F18, F21, F22, F23, F24, F25, F26, F29, F31, F32, F36, F44, F45, F50, F52, F55, F56, F59, F60, F61, F62, F66, F77, F79, F80, F81, F85, F87, F89, F90, F91, F92, F93, F94, G01, G03, G04, G05, G12, G16, G18, G21, G22, G23, G24, G25, G26, G29, G31, G32, G36, G44, G45, G50, G52, G55, G56, G59, G60, G61, G62, G66, G77, G79, G80, G81, G85, G87, G89, G90, G91, G92, G93, G94, L01, L04, L05, L12, L18, L23, L25, L29, L32, L36, L44, L52, L59, L60, L61, L62, L66, L67, L79, L80, L81, L87, L89, L91, L92, L93, L94, M01, M04, M05, M12, M18, M21, M23, M24, M25, M29, M30, M32, M36, M44, M50, M52, M55, M56, M59, M60, M61, M62, M66, M67, M68, M79, M80, M81, M87, M89, M91, M92, M93, M94, Q12, Q18, S67, S74, S89);

*Totals;

Total_IG_Expenditure = sum(L01, L04, L05, L12, L18, L23, L25, L29, L32, L36, L44, L50, L52, L59, L60, L61, L62, L66, L67, L79, L80, L81, L87, L89, L91, L92, L93, L94, M01, M04, M05, M12, M18, M21, M23, M24, M25, M29, M30, M32, M36, M44, M50, M52, M52, M55, M56, M59, M60, M61, M62, M66, M67, M68, M79, M80, M81, M87, M89, M91, M92, M93, M94, Q12, Q18, S67, S89);

Direct_Expenditure = sum(E01, E03, E04, E05, E12, E16, E18, E21, E22, E23, E24, E25, E26, E29, E31, E32, E36, E44, E45, E50, E52, E55, E56, E59, E60, E61, E62, E66, E74, E75, E77, E79, E80, E81, E85, E87, E89, E90, E91, E92, E93, E94, F01, F03, F04, F05, F12, F16, F18, F21, F22, F23, F24, F25, F26, F29, F31, F32, F36, F44, F45, F50, F52, F55, F56, F59, F60, F61, F62, F66, F77, F79, F80, F81, F85, F87, F89, F90, F91, F92, F93, F94, G01, G03, G04, G05, G12, G16, G18, G21, G22, G23, G24, G25, G26, G29, G31, G32, G36, G44, G45, G50, G52, G55, G56, G59, G60, G61, G62, G66, G77, G79, G80, G81, G85, G87, G89, G90, G91, G92, G93, G94, X11, X12, Y05, Y06, Y14, Y53, J19, J67, J68, J85, I89, I91, I92, I93, I94);

Total_Current_Oper = sum(E01, E03, E04, E05, E12, E16, E18, E21, E22, E23, E24, E25, E26, E29, E31, E32, E36, E44, E45, E50, E52, E55, E56, E59, E60, E61, E62, E66, E74, E75, E77, E79, E80, E81, E85, E87, E89, E90, E91, E92, E93, E94);

Total_Capital_Outlays = sum(F01, F03, F04, F05, F12, F16, F18, F21, F22, F23, F24, F25, F26, F29, F31, F32, F36, F44, F45, F50, F52, F55, F56, F59, F60, F61, F62, F66, F77, F79, F80, F81, F85, F87, F89, F90, F91, F92, F93, F94, G01, G03, G04, G05, G12, G16, G18, G21, G22, G23, G24, G25, G26, G29, G31, G32, G36, G44, G45, G50, G52, G55, G56, G59, G60, G61, G62, G66, G77, G79, G80, G81, G85, G87, G89, G90, G91, G92, G93, G94);

Total_Construction = sum(F01, F03, F04, F05, F12, F16, F18, F21, F22, F23, F24, F25, F26, F29, F31, F32, F36, F44, F45, F50, F52, F55, F56, F59, F60, F61, F62, F66, F77, F79, F80, F81, F85, F87, F89, F90, F91, F92, F93, F94);

Tot_Assist___Subsidies = sum(J19, J67, J68, J85);

Total_Interest_on_Debt = sum(I89, I91, I92, I93, I94);

Total_Insur_Trust_Ben = sum(X11, X12, Y05, Y06, Y14, Y53);

Total_Salaries___Wages = Z00;

Total_Current_Expend = sum(Total_Expenditure, - Total_Capital_Outlays);

* General Expenses;

General_Expenditure = sum(E01, E03, E04, E05, E12, E16, E18, E21, E22, E23, E24, E25, E26, E29, E31, E32, E36, E44, E45, E50, E52, E55, E56, E59, E60, E61, E62, E66, E74, E75, E77, E79, E80, E81, E85, E87, E89, E90, E91, E92, E93, E94, F01, F03, F04, F05, F12, F16, F18, F21, F22, F23, F24, F25, F26, F29, F31, F32, F36, F44, F45, F50, F52, F55, F56, F59, F60, F61, F62, F66, F77, F79, F80, F81, F85, F87, F89, F90, F91, F92, F93, F94, G01, G03, G04, G05, G12, G16, G18, G21, G22, G23, G24, G25, G26, G29, G31, G32, G36, G44, G45, G50, G52, G55, G56, G59, G60, G61, G62, G66, G77, G79, G80, G81, G85, G87, G89, G90, G91, G92, G93, G94, X11, X12, Y05, Y06, Y14, Y53, J19, J67, J68, J85, I89, I91, I92, I93, I94);

IG_Exp_To_State_Govt = sum(L01, L04, L05, L12, L18, L23, L25, L29, L32, L36, L44, L50, L52, L59, L60, L61, L62, L66, L67, L79, L80, L81, L87, L89, L91, L92, L93, L94);

IG_Exp_To_Local_Govts = sum(M01, M04, M05, M12, M18, M21, M23, M24, M25, M29, M30, M32, M36, M44, M50, M52, M52, M55, M56, M59, M60, M61, M62, M66, M67, M68, M79, M80, M81, M87, M89, M91, M92, M93, M94);

IG_Exp_To_Federal_Govt = sum(S67, S74, S89);

Direct_General_Expend = sum(E01, E03, E04, E05, E12, E16, E18, E21, E22, E23, E24, E25, E26, E29, E31, E32, E36, E44, E45, E50, E52, E55, E56, E59, E60, E61, E62, E66, E74, E75, E77, E79, E80, E81, E85, E87, E89, E90, E91, E92, E93, E94, F01, F03, F04, F05, F12, F16, F18, F21, F22, F23, F24, F25, F26, F29, F31, F32, F36, F44, F45, F50, F52, F55, F56, F59, F60, F61, F62, F66, F77, F79, F80, F81, F85, F87, F89, F90, F91, F92, F93, F94, G01, G03, G04, G05, G12, G16, G18, G21, G22, G23, G24, G25, G26, G29, G31, G32, G36, G44, G45, G50, G52, G55, G56, G59, G60, G61, G62, G66, G77, G79, G80, G81, G85, G87, G89, G90, G91, G92, G93, G94, X11, X12, Y05, Y06, Y14, Y53, J19, J67, J68, J85, I89, I91, I92, I93, I94);

General_Current_Oper = sum(E01, E03, E04, E05, E12, E16, E18, E21, E22, E23, E24, E25, E26, E29, E31, E32, E36, E44, E45, E50, E52, E55, E56, E59, E60, E61, E62, E66, E74, E75, E77, E79, E80, E81, E85, E87, E89, E90, E91, E92, E93, E94);

General_Capital_Outlay = sum(F01, F03, F04, F05, F12, F16, F18, F21, F22, F23, F24, F25, F26, F29, F31, F32, F36, F44, F45, F50, F52, F55, F56, F59, F60, F61, F62, F66, F77, F79, F80, F81, F85, F87, F89, G01, G03, G04, G05, G12, G16, G18, G21, G22, G23, G24, G25, G26, G29, G31, G32, G36, G44, G45, G50, G52, G55, G56, G59, G60, G61, G62, G66, G77, G79, G80, G81, G85, G87, G89);

General_Construction = sum(F01, F03, F04, F05, F12, F16, F18, F21, F22, F23, F24, F25, F26, F29, F31, F32, F36, F44, F45, F50, F52, F55, F56, F59, F60, F61, F62, F66, F77, F79, F80, F81, F85, F87, F89, F90, F91, F92, F93, F94);

General_Assist___Sub = Tot_Assist___Subsidies;

General_Debt_Interest = I89;

General_Current_Expend = sum(General_Expenditure, - General_Capital_Outlay);

* Air Transport;

Air_Trans_Total_Expend = sum(E01 , F01 , G01 , L01 , M01);

Air_Trans_Direct_Expend = sum(E01 , F01 , G01);

Air_Trans_Cap_Outlay = sum(F01 , G01);

Air_Trans_Construction = F01;

Air_Trans_IG_To_State = L01;

Air_Trans_IG_Local_Govts = M01;

* Misc Commercial Activities;

Misc_Com_Activ_Tot_Exp = sum(E03 , F03 , G03);

Misc_Com_Activ_Cap_Out = sum(F03 , G03);

Misc_Com_Activ_Constr = F03;

* Correctional Institutions;

Correct_Total_Exp = sum(E04 , F04 , G04 , E05 , F05 , G05 , L04 , L05 , M04 , M05);

Correct_Direct_Exp = sum(E04 , E05 , F04 , G04 , F05 , G05);

Correct_Cap_Outlay = sum(F04 , G04 , F05 , G05);

Correct_Construct = sum(F04 , F05);

Correct_IG_To_St = sum(L04 , L05);

Correct_IG_Loc_Govts = sum(M04 , M05);

* Missing national defense code 06;

* Education;

Total_Educ_Total_Exp = sum(E12, F12, G12, E16, F16, G16, E18, F18, G18, J19, E21, F21, G21, L12, M12, Q12, L18, M18, L21, M21);

Total_Educ_Direct_Exp = sum(E12, F12, G12, E16, F16, G16, E18, F18, G18, J19, E21, F21, G21);

Total_Educ_Assist___Sub = J19;

Total_Educ_Cap_Outlay = sum(F12, F16, F18, F21, G12, G16, G18, G21);

Total_Educ_Construct = sum(F12 , F16 , F18 , F21);

* Elementary and Secondary Education;

Elem_Educ_Total_Exp = sum(E12, F12, G12, L12, M12, Q12);

Elem_Educ_Direct_Exp = sum(E12, F12, G12);

Elem_Educ_Cap_Outlay = sum(F12, G12);

Elem_Educ_Construction = F12;

Elem_Educ_IG_To_State = L12;

Elem_Educ_IG_Local_Govts = M12;

Elem_Educ_IG_Sch_to_Sch = Q12;

* Higher Education;

Higher_Ed_Total_Exp = sum(E16, E18, F16, F18, G16, G18, L18, M18);

Higher_Ed_Direct_Exp = sum(E16, E18, F16, F18, G16, G18);

Higher_Ed_Cap_Outlay = sum(F16, F18, G16, G18);

Higher_Ed_Construct = sum(F16, F18);

Higher_Ed_IG_To_St = L18;

Higher_Ed_IG_Loc_Govts = M18;

* Education not otherwise classified;

Educ_NEC_Total_Expend = sum(E21, F21, G21, L21, M21);

Educ_NEC_Direct_Expend = sum(E21, F21, G21);

Educ_NEC_Assistance = **.**; *The prior code, E19 no longer exists in the data;

Educ_NEC_Cap_Outlay = sum(F21, G21);

Educ_NEC_Construction = F21;

Educ_NEC_IG_To_State = L21;

Educ_NEC_IG_Local_Govts = M21;

* Missing post offices code 14;

*Employment Security Administration;

Emp_Sec_Adm_Direct_Exp = sum(E22 , F22 , G22);

Emp_Sec_Adm_Cap_Outlay = sum(F22 , G22);

Emp_Sec_Adm_Construct = F22;

*Financial Administration;

Fin_Admin_Total_Exp = sum(E23, F23, G23, L23, M23);

Fin_Admin_Direct_Exp = sum(E23, F23, G23);

Fin_Admin_Cap_Outlay = sum(F23, G23);

Fin_Admin_Construction = F23;

Fin_Admin_IG_To_State = L23;

Fin_Admin_IG_Local_Govts = M23;

*Fire protection;

Fire_Prot_Total_Expend = sum(E24, F24, G24, L24, M24);

Fire_Prot_Direct_Exp = sum(E24, F24, G24);

Fire_Prot_Cap_Outlay = sum(F24, G24);

Fire_Prot_Construction = F24;

Fire_Prot_IG_To_State = L24;

Fire_Prot_IG_Local_Govts = M24;

*Judicial Expenditures;

Judicial_Total_Expend = sum(E25, F25, G25, L25, M25);

Judicial_Direct_Expend = sum(E25, F25, G25);

Judicial_Cap_Outlay = sum(F25, G25);

Judicial_Construction = F25;

Judicial_IG_To_State = L25;

Judicial_IG_Local_Govts = M25;

*Central Staff Services;

Cen_Staff_Total_Expend = sum(E29, F29, G29, L29, M29);

Cen_Staff_Direct_Exp = sum(E29, F29, G29);

Cen_Staff_Cap_Outlay = sum(F29, G29);

Cen_Staff_Construction = F29;

Cen_Staff_IG_To_State = L29;

Cen_Staff_IG_Local_Govts = M29;

* General Public Buildings;

Gen_Pub_Bldg_Total_Exp = sum(E31, F31, G31);

Gen_Pub_Bldg_Cap_Out = sum(F31, G31);

Gen_Pub_Bldg_Construct = F31;

* Health;

Health_Total_Expend = sum(E32, F32, G32, L32, M32);

Health_Direct_Expend = sum(E32, F32, G32);

Health_Capital_Outlay = sum(F32, G32);

Health_Construction = F32;

Health_IG_To_State = L32;

Health_IG_Local_Govts = M32;

*Hospitals;

Total_Hospital_Total_Exp = sum(E36, F36, G36, L36, M36);

Total_Hospital_Dir_Exp = sum(E36, F36, G36);

Total_Hospital_Cap_Out = sum(F36, G36);

Total_Hospital_Construct = F36;

Total_Hospital_IG_To_State = L36;

Total_Hospital_IG_Loc_Govts = M36;

*Federal Owned Hospitals - Veterans;

Own_Hospital_Total_Exp = sum(E37, F37, G37);

Own_Hospital_Cap_Out = sum(F37, G37);

Own_Hospital_Construct = F37;

* Other Hospital Expenses - Federal but not veterans;

Hosp_Other_Total_Exp = sum(E39, F39, G39, L39, M39);

Hosp_Other_Direct_Exp = sum(E39, F39, G39);

Hosp_Other_Cap_Outlay = sum(F39, G39);

Hosp_Other_Construct = F39;

Hosp_Other_IG_To_State = L39;

Hosp_Other_IG_Loc_Govts = M39;

* Highways;

Total_Highways_Tot_Exp = sum(E44, F44, G44, E45, F45, G45, L44, M44);

Total_Highways_Dir_Exp = sum(E44, F44, G44, E45, F45, G45);

Total_Highways_Cap_Out = sum(F44, G44, F45, G45);

Total_Highways_Construct = sum(F44, F45);

* Non-Toll Highways;

Regular_Hwy_Total_Exp = sum(E44, F44, G44, L44, M44);

Regular_Hwy_Direct_Exp = sum(E44, F44, G44);

Regular_Hwy_Cap_Outlay = sum(F44, G44);

Regular_Hwy_Construct = F44;

Regular_Hwy_IG_To_Sta = L44;

Regular_Hwy_IG_Loc_Govts = M44;

* Toll Highways;

Toll_Hwy_Total_Expend = sum(E45, F45, G45);

Toll_Hwy_Cap_Outlay = sum(F45, G45);

Toll_Hwy_Construction = F45;

* Transit Subsidies - These data were eliminated in the 2005 data;

Transit_Sub_Total_Exp = **.**;

Transit_Sub_Direct_Sub = **.**;

Transit_Sub_IG_To_Sta = **.**;

Transit_Sub_IG_Loc_Govts = **.**;

Transit_Sub_To_Own_Sys = **.**;

*Housing and Community Development;

Hous___Com_Total_Exp = sum(E50, F50, G50, L50, M50);

Hous___Com_Direct_Exp = sum(E50, F50, G50);

Hous___Com_Cap_Outlay = sum(F50, G50);

Hous___Com_Construct = F50;

Hous___Com_IG_To_State = L50;

Hous___Com_IG_Loc_Govts = M50;

* Libraries;

Libraries_Total_Expend = sum(E52, F52, G52, L52, M52);

Libraries_Direct_Exp = sum(E52, F52, G52);

Libraries_Cap_Outlay = sum(F52, G52);

Libraries_Construction = F52;

Libraries_IG_To_State = L52;

Libraries_IG_Local_Govts = M52;

*Natural Resources;

Natural_Res_Total_Exp = sum(E55, F55, G55, M55, E56, F56, G56, M56, E59, F59, G59, L59, M59);

Natural_Res_Direct_Exp = sum(E55, F55, G55, E56, F56, G56, E59, F59, G59);

Natural_Res_Cap_Outlay = sum(F55, G55, F56, G56, F59, G59);

Natural_Res_Construct = sum(F55, F56, F59);

Natural_Res_IG_To_Sta = L59;

Natural_Res_IG_Loc_Govts = sum(M55, M56 , M59);

* Parking Facilities;

Parking_Total_Expend = sum(E60, F60, G60, L60, M60);

Parking_Direct_Expend = sum(E60, F60, G60);

Parking_Capital_Outlay = sum(F60, G60);

Parking_Construction = F60;

Parking_IG_To_State = L60;

Parking_IG_Local_Govts = M60;

* Parks and Recreation;

Parks___Rec_Total_Exp = sum(E61, F61, G61, L61, M61);

Parks___Rec_Direct_Exp = sum(E61, F61, G61);

Parks___Rec_Cap_Outlay = sum(F61, G61);

Parks___Rec_Construct = F61;

Parks___Rec_IG_To_Sta = L61;

Parks___Rec_IG_Loc_Govts = M61;

* Police Protection;

Police_Prot_Total_Exp = sum(E62, F62, G62, L62, M62);

Police_Prot_Direct_Exp = sum(E62, F62, G62);

Police_Prot_Cap_Outlay = sum(F62, G62);

Police_Prot_Construct = F62;

Police_Prot_IG_To_Sta = L62;

Police_Prot_IG_Loc_Govts = M62;

* Protective Inspection and Regulation;

Prot_Insp_Total_Exp = sum(E66, F66, G66, L66, M66);

Prot_Insp_Direct_Exp = sum(E66, F66, G66);

Prot_Insp_Cap_Outlay = sum(F66, G66);

Prot_Insp_Construction = F66;

Prot_Insp_IG_To_State = L66;

Prot_Insp_IG_Local_Govts = M66;

* Public Welfare;

Public_Welf_Total_Exp = sum(J67, L67, M67, J68 , M68, E74, E75, S74, E77, F77, G77, E79, F79, G79, L79, M79);

Public_Welf_Direct_Exp = sum(J67, J68 , E74, E75, E77, F77, G77, E79, F79, G79);

Public_Welf_Cash_Asst = sum(J67, J68, M67, M68);

Public_Welf_Cap_Outlay = sum(F77, G77, F79, G79);

Public_Welf_Construct = sum(F77, F79);

* Public Welfare-Categorical Assistance Programs;

Welf_Categ_Total_Exp = sum(J67, L67, M67);

Welf_Categ_Cash_Assist = J67;

Welf_Categ_IG_To_State = L67;

Welf_Categ_IG_Loc_Govts = M67;

* Public Welfare-Cash assistance payments;

Welf_Cash_Total_Exp = sum(J68 , M68);

Welf_Cash_Cash_Assist = J68;

Welf_Cash_IG_Local_Govts = M68;

* Public Welfare-Vendor Payments;

Welf_Vend_Pmts_Medical = E74;

Welf_Vend_Pmts_NEC = E75;

* State Share of Medicare Part D;

Welf_State_Share_Part_D = S74;

* Public Welfare-Institutions;

Welf_Ins_Total_Exp = sum(E77, F77, G77);

Welf_Ins_Cap_Outlay = sum(F77, G77);

Welf_Ins_Construction = F77;

*Public Welfare not elsewhere classified;

Welf_NEC_Total_Expend = sum(E79, F79, G79, L79, M79);

Welf_NEC_Direct_Expend = sum(E79, F79, G79);

Welf_NEC_Cap_Outlay = sum(F79, G79);

Welf_NEC_Construction = F79;

Welf_NEC_IG_To_State = L79;

Welf_NEC_IG_Local_Govts = M79;

*Sewerage;

Sewerage_Total_Expend = sum(E80, F80, G80, L80, M80);

Sewerage_Direct_Expend = sum(E80, F80, G80);

Sewerage_Cap_Outlay = sum(F80, G80);

Sewerage_Construction = F80;

Sewerage_IG_To_State = L80;

Sewerage_IG_Local_Govts = M80;

*Solid Waste Management;

SW_Mgmt_Total_Expend = sum(E81, F81, G81, L81, M81);

SW_Mgmt_Direct_Expend = sum(E81, F81, G81);

SW_Mgmt_Capital_Outlay = sum(F81, G81);

SW_Mgmt_Construction = F81;

SW_Mgmt_IG_To_State = L81;

SW_Mgmt_IG_Local_Govts = M81;

*Sea and Inland Port Facilities;

Water_Trans_Total_Exp = sum(E87, F87, G87, L87, M87);

Water_Trans_Direct_Exp = sum(E87, F87, G87);

Water_Trans_Cap_Outlay = sum(F87, G87);

Water_Trans_Construct = F87;

Water_Trans_IG_To_Sta = L87;

Water_Trans_IG_Loc_Govts = M87;

*Interest on General Debt;

Interest_on_Gen_Debt = I89;

* General Expenditure not elsewhere classified;

General_NEC_Total_Exp = sum(E89, F89, G89, L89, M89, S89, J89);

General_NEC_Direct_Exp = sum(E89, F89, G89, J89);

VetBonus = J89;

General_NEC_Cap_Outlay = sum(F89, G89);

General_NEC_Construct = F89;

General_NEC_IG_To_St = L89;

General_NEC_IG_Loc_Govts = M89;

General_NEC_IG_To_Fed = S89;

* Liquor Stores;

Liquor_Stores_Tot_Exp = sum(E90, F90, G90);

Liquor_Stores_Cap_Out = sum(F90, G90);

Liquor_Stores_Constr = F90;

* Total Utilities;

Total_Util_Total_Exp = sum(E91, I91, F91, G91, L91, M91, E92, I92, F92, G92, L92, M92, E93, I93, F93, G93, L93, M93, E94, I94, F94, G94, L94, M94);

Total_Util_Inter_Exp = sum(I91, I92, I93, I94);

Total_Util_Cap_Outlay = sum(F91, F92, F93, F94, G91, G92, G93, G94);

Total_Util_Construct = sum(F91, F92, F93, F94);

* Water Supply Utilities;

Water_Util_Total_Exp = sum(E91, I91, F91, G91, L91, M91);

Water_Util_Inter_Exp = I91;

Water_Util_Cap_Outlay = sum(F91, G91);

Water_Util_Construct = F91;

* Electric Power Utilities;

Elec_Util_Total_Exp = sum(E92, I92, F92, G92, L92, M92);

Elec_Util_Inter_Exp = I92;

Elec_Util_Cap_Outlay = sum(F92, G92);

Elec_Util_Construct = F92;

* Gas Supply Utilities;

Gas_Util_Total_Exp = sum(E93, I93, F93, G93, L93, M93);

Gas_Util_Inter_Exp = I93;

Gas_Util_Cap_Outlay = sum(F93, G93);

Gas_Util_Construct = F93;

* Transit System Utilities;

Trans_Util_Total_Exp = sum(E94, I94, F94, G94, L94, M94);

Trans_Util_Inter_Exp = I94;

Trans_Util_Cap_Outlay = sum(F94, G94);

Trans_Util_Construct = F94;

* Employee Retirement;

Emp_Ret_Total_Expend = sum(X11, X12);

Emp_Ret_Benefit_Paymts = X11;

Emp_Ret_Withdrawals = X12;

Emp_Ret_Other_Paymts = **.**; *This code used to be X14 is now obsolete since 2002 represented realized losses and is coded elsewhere;

* Unemployment Compensation;

Unemp_Comp_Total_Exp = sum(Y05, Y06);

Unemp_Comp_Ben_Paymts = Y05;

Unemp_Ext___Spec_Pmts = Y06;

* Debt totals;

Total_Debt_Outstanding = sum(_44T, _49U, _64V);

Total_Long_Term_Debt_Out = sum(_44T, _49U);

ST_Debt_End_of_Year = _64V;

* Beginning Long Term Debt Outstanding;

Total_Beg_LTD_Out = sum(_19T, _19U);

Beg_LTD_Out_Private_Purp = _19T;

Beg_LTD_Out_All_Other = _19U;

Beg_LTD_Out_Utility = **.**; *All detailed debt codes were discontinued in 2005;

Beg_LTD_Out_Water_Util = **.**;

Beg_LTD_Out_Elec_Util = **.**;

Beg_LTD_Out_Gas_Util = **.**;

Beg_LTD_Out_Trans_Util = **.**;

Beg_LTD_Out_General = **.**;

Beg_LTD_Out_Education = **.**;

Beg_LTD_Out_Priv_Purp = **.**;

Beg_LTD_Out_Other_NEC = **.**;

* Long Term Debt Issued;

Total_LTD_Issued = sum(_24T, _29U);

LTD_Iss_Private_Purp = _24T;

LTD_Iss_All_Other = _29U;

LTD_Iss_Utility = **.**;

LTD_Iss_Util_Water = **.**;

LTD_Iss_Util_Electric = **.**;

LTD_Iss_Util_Gas_Supply = **.**;

LTD_Iss_Util_Transit = **.**;

LTD_Iss_General = **.**;

LTD_Iss_Gen_Elem_Educ = **.**;

LTD_Iss_Gen_Other_Educ = **.**;

LTD_Iss_Gen_Other_NEC = **.**;

* Long-Term Debt Issued, Full-Faith and Credit; *Codes discontinued in 2005;

Total_LTD_Iss_FFC = **.**;

LTD_Iss_FFC_Utility = **.**;

LTD_Iss_FFC_Water_Util = **.**;

LTD_Iss_FFC_Elec_Util = **.**;

LTD_Iss_FFC_Gas_Util = **.**;

LTD_Iss_FFC_Trans_Util = **.**;

LTD_Iss_FFC_General = **.**;

LTD_Iss_FFC_Elem_Educ = **.**;

LTD_Iss_FFC_Other_Educ = **.**;

LTD_Iss_FFC_Other_NEC = **.**;

* Long-Term Debt Issued, Nonguaranteed;

Total_LTD_Iss_NG = **.**;

LTD_Iss_NG_Utility = **.**;

LTD_Iss_NG_Water_Util = **.**;

LTD_Iss_NG_Elec_Util = **.**;

LTD_Iss_NG_Gas_Util = **.**;

LTD_Iss_NG_Trans_Util = **.**;

LTD_Iss_NG_General = **.**;

LTD_Iss_NG_Elem_Educ = **.**;

LTD_Iss_NG_Other_Educ = **.**;

LTD_Iss_NG_Private_Purp = **.**;

LTD_Iss_NG_Other_NEC = **.**;

* Long-Term Debt Issued, Unspecified Issue;

Total_LTD_Iss_Unsp = **.**;

LTD_Iss_Unsp_Utility = **.**;

LTD_Iss_Unsp_Water_Util = **.**;

LTD_Iss_Unsp_Elec_Util = **.**;

LTD_Iss_Unsp_Gas_Util = **.**;

LTD_Iss_Unsp_Trans_Util = **.**;

LTD_Iss_Unsp_General = **.**;

LTD_Iss_Unsp_Elem_Educ = **.**;

LTD_Iss_Unsp_Other_Educ = **.**;

LTD_Iss_Unsp_Other_NEC = **.**;

*Long-Term Debt Retired During Fiscal Year;

Total_LTD_Retired = sum(_34T, _39U);

LTD_Ret_Private_Purp = _34T;

LTD_Ret_All_Other = _39U;

LTD_Ret_Utility = **.**;

LTD_Ret_Util_Water = **.**;

LTD_Ret_Util_Electric = **.**;

LTD_Ret_Util_Gas_Supply = **.**;

LTD_Ret_Util_Transit = **.**;

LTD_Ret_General = **.**;

LTD_Ret_Gen_Elem_Educ = **.**;

LTD_Ret_Gen_Other_Educ = **.**;

LTD_Ret_Gen_Other_NEC = **.**;

* Long-Term Debt Retired, Full-Faith and Credit;

Total_LTD_Ret_FFC = **.**;

LTD_Ret_FFC_Utility = **.**;

LTD_Ret_FFC_Water_Util = **.**;

LTD_Ret_FFC_Elec_Util = **.**;

LTD_Ret_FFC_Gas_Util = **.**;

LTD_Ret_FFC_Trans_Util = **.**;

LTD_Ret_FFC_General = **.**;

LTD_Ret_FFC_Elem_Educ = **.**;

LTD_Ret_FFC_Other_Educ = **.**;

LTD_Ret_FFC_Other_NEC = **.**;

* Long-Term Debt Retired, Nonguaranteed;

Total_LTD_Ret_NG = **.**;

LTD_Ret_NG_Utility = **.**;

LTD_Ret_NG_Water_Util = **.**;

LTD_Ret_NG_Elec_Util = **.**;

LTD_Ret_NG_Gas_Util = **.**;

LTD_Ret_NG_Trans_Util = **.**;

LTD_Ret_NG_General = **.**;

LTD_Ret_NG_Elem_Educ = **.**;

LTD_Ret_NG_Other_Educ = **.**;

LTD_Ret_NG_Private_Purp = **.**;

LTD_Ret_NG_Other_NEC = **.**;

* Long-Term Debt Retired, Unspecified;

Total_LTD_Ret_Unsp = **.**;

LTD_Ret_Unsp_Utility = **.**;

LTD_Ret_Unsp_Water_Util = **.**;

LTD_Ret_Unsp_Elec_Utili = **.**;

LTD_Ret_Unsp_Gas_Util = **.**;

LTD_Ret_Unsp_Trans_Util = **.**;

LTD_Ret_Unsp_General = **.**;

LTD_Ret_Unsp_Elem_Educ = **.**;

LTD_Ret_Unsp_Other_Educ = **.**;

LTD_Ret_Unsp_Other_NEC = **.**;

* Long-Term Debt Outstanding;

Total_LTD_Out = sum(_44T, _49U);

LTD_Out_Private_Purp = _44T;

LTD_Out_All_Other = _49U;

Total_LTD_Out_Utility = **.**;

LTD_Out_Util_Water = **.**;

LTD_Out_Util_Electric = **.**;

LTD_Out_Util_Gas_Supply = **.**;

LTD_Out_Util_Transit = **.**;

LTD_Out_General = **.**;

LTD_Out_Gen_Elem_Educ = **.**;

LTD_Out_Gen_Other_Educ = **.**;

LTD_Out_Gen_Other_NEC = **.**;

* Long-Term Debt Outstanding, Full-Faith and Credit;

Total_LTD_Out_FFC = **.**;

LTD_Out_FFC_Utility = **.**;

LTD_Out_FFC_Water_Util = **.**;

LTD_Out_FFC_Elec_Util = **.**;

LTD_Out_FFC_Gas_Util = **.**;

LTD_Out_FFC_Trans_Util = **.**;

LTD_Out_FFC_General = **.**;

LTD_Out_FFC_Elem_Educ = **.**;

LTD_Out_FFC_Other_Educ = **.**;

LTD_Out_FFC_Other_NEC = **.**;

* Long-Term Debt Outstanding, Nonguaranteed;

Tot_LTD_Out_NG = **.**;

LTD_Out_NG_Utility = **.**;

LTD_Out_NG_Water_Util = **.**;

LTD_Out_NG_Elec_Util = **.**;

LTD_Out_NG_Gas_Util = **.**;

LTD_Out_NG_Trans_Util = **.**;

LTD_Out_NG_General = **.**;

LTD_Out_NG_Elem_Educ = **.**;

LTD_Out_NG_Other_Educ = **.**;

LTD_Out_NG_Private_Purp = **.**;

LTD_Out_NG_Other_NEC = **.**;

* Cash and Securities;

Total_Cash___Securities = sum(W01, W31, W61, X21, X30, Z77, Z78, X42, X44, X47, Y07, Y08, Y21, Y61);

* Insurance Trust Funds Only Cash and Securities;

Insur_Trust_Cash___Sec = sum(X21, X30, Z77, Z78, X42, X44, X47, Y07, Y08, Y21, Y61);

* Employee Retirement Systems Cash and Securities;

Emp_Retire_Cash___Sec = sum(X21, X30, X35, Z77, Z78, X42, X47, X44);

Emp_Retire_Cash___Dep = X21;

Emp_Retire_Total_Sec = sum(X30, X35, Z77, Z78, X42, X47, X44);

Emp_Retire_Sec_Tot_Fed = X30;

Emp_Retire_Sec_S_L_Secur = X35;

Emp_Retire_Sec_Tot_Nong = sum(Z77, Z78, X42, X47, X44);

Emp_Retire_Sec_Corp_Bds = Z77;

Emp_Retire_Sec_Corp_Stk = Z78;

Emp_Retire_Sec_Mortgages = X42;

Emp_Retire_Sec_Misc_Inv = X47;

Emp_Retire_Sec_Oth_Nong = X44;

* Unemployment Compensation Funds Cash and Securities;

Unemp_Comp_Cash___Sec = sum(Y07, Y08);

Unemp_Comp_Bal_In_US_Trs = Y07;

Unemp_Comp_Other_Balance = Y08;

* Other Insurance Trusts Holdings Cash and Securities;

Nonin_Trust_Cash___Sec = sum(W01, W31, W61);

* Sinking Funds (debt service funds) Cash and Securities;

Sinking_Fd_Cash___Sec = W01;

* Bond Funds Cash and Securities;

Bond_Fd_Cash___Sec = W31;

* All Other Noninsurance Funds Cash and Securities;

Oth_Nonin_Fd_Cash___Sec = W61;

**run**;

*Merge the older data with the newer data that has had its format converted;

**Proc** **append** base=LargeData data=ConvertedNewData force;

**run**;

**Data** LargestData;

Set LargeData;

*Calculate several totals from revenue data and to correct for differences between old and new data;

Motor_Vehicle_License_Total = sum(Motor_Vehicle_License, Motor_Veh_Oper_License);

Fed_IGR_Gen_Support = sum(Fed_IGR_Gen_Support, Fed_IGR_Gen_Rev_Shar);

State_IGR_Gen_Sup = sum(State_IGR_Oth_Gen_Sup, State_IGR_Tax_Relief);

Chg_Total_Elem_Education = sum(Chg_Elem_Ed_Sch_Lunch, Chg_Elem_Ed_Tuition, Chg_Elem_Ed_NEC);

Chg_Highways = sum(Chg_Regular_Highways, Chg_Toll_Highways);

Prop_Sale_Total = sum(Prop_Sale_Hous_Com_Dev, Prop_Sale_Other);

Total_Other_Capital_Outlays = sum(Total_Capital_Outlays, -Total_Construction);

General_Capital_Outlay_Other = sum(General_Capital_Outlay, -General_Construction);

*Calculate current expenditure data for every function where it is missing;

Air_Trans_Current_Exp = sum(Air_Trans_Direct_Expend, -Air_Trans_Cap_Outlay);

Misc_Com_Activ_Current_Exp = sum(Misc_Com_Activ_Tot_Exp, -Misc_Com_Activ_Cap_Out);

Correct_Current_Exp = sum(Correct_Direct_Exp, -Correct_Cap_Outlay);

Total_Educ_Current_Exp = sum(Total_Educ_Direct_Exp, -Total_Educ_Cap_Outlay);

Elem_Educ_Current_Exp = sum(Elem_Educ_Direct_Exp, -Elem_Educ_Cap_Outlay);

Higher_Ed_Current_Exp = sum(Higher_Ed_Direct_Exp, -Higher_Ed_Cap_Outlay);

Educ_NEC_Current_Exp = sum(Educ_NEC_Direct_Expend, -Educ_NEC_Cap_Outlay);

Emp_Sec_Adm_Current_Exp = sum(Emp_Sec_Adm_Direct_Exp, -Emp_Sec_Adm_Cap_Outlay);

Fin_Admin_Current_Exp = sum(Fin_Admin_Direct_Exp, -Fin_Admin_Cap_Outlay);

Fire_Prot_Current_Exp = sum(Fire_Prot_Direct_Exp, -Fire_Prot_Cap_Outlay);

Judicial_Current_Exp = sum(Judicial_Direct_Expend, -Judicial_Cap_Outlay);

Cen_Staff_Current_Exp = sum(Cen_Staff_Direct_Exp, -Cen_Staff_Cap_Outlay);

Gen_Pub_Bldg_Current_Exp = sum(Gen_Pub_Bldg_Total_Exp, -Gen_Pub_Bldg_Cap_Out);

Health_Current_Exp = sum(Health_Direct_Expend, -Health_Capital_Outlay);

Total_Hospital_Current_Exp = sum(Total_Hospital_Dir_Exp, -Total_Hospital_Cap_Out);

Own_Hospital_Current_Exp = sum(Own_Hospital_Total_Exp, -Own_Hospital_Cap_Out);

Hosp_Other_Current_Exp = sum(Hosp_Other_Direct_Exp, -Hosp_Other_Cap_Outlay);

Total_Highways_Current_Exp = sum(Total_Highways_Dir_Exp, -Total_Highways_Cap_Out);

Regular_Hwy_Current_Exp = sum(Regular_Hwy_Direct_Exp, -Regular_Hwy_Cap_Outlay);

Toll_Hwy_Current_Exp = sum(Toll_Hwy_Total_Expend, -Toll_Hwy_Cap_Outlay);

Hous___Com_Current_Exp = sum(Hous___Com_Direct_Exp, -Hous___Com_Cap_Outlay);

Libraries_Current_Exp = sum(Libraries_Direct_Exp, -Libraries_Cap_Outlay);

Natural_Res_Current_Exp = sum(Natural_Res_Direct_Exp, -Natural_Res_Cap_Outlay);

Parking_Current_Exp = sum(Parking_Direct_Expend, -Parking_Capital_Outlay);

Parks___Rec_Current_Exp = sum(Parks___Rec_Direct_Exp, -Parks___Rec_Cap_Outlay);

Police_Prot_Current_Exp = sum(Police_Prot_Direct_Exp, -Police_Prot_Cap_Outlay);

Prot_Insp_Current_Exp = sum(Prot_Insp_Direct_Exp, -Prot_Insp_Cap_Outlay);

Public_Welf_Current_Exp = sum(Public_Welf_Direct_Exp, -Public_Welf_Cap_Outlay, -Public_Welf_Cash_Asst);

Welf_Ins_Current_Exp = sum(Welf_Ins_Total_Exp, -Welf_Ins_Cap_Outlay);

Welf_NEC_Current_Exp = sum(Welf_NEC_Direct_Expend, -Welf_NEC_Cap_Outlay);

Sewerage_Current_Exp = sum(Sewerage_Direct_Expend, -Sewerage_Cap_Outlay);

SW_Mgmt_Current_Exp = sum(SW_Mgmt_Direct_Expend, -SW_Mgmt_Capital_Outlay);

Water_Trans_Current_Exp = sum(Water_Trans_Direct_Exp, -Water_Trans_Cap_Outlay);

General_NEC_Current_Exp = sum(General_NEC_Direct_Exp, -General_NEC_Cap_Outlay, -VetBonus);

Liquor_Stores_Current_Exp = sum(Liquor_Stores_Tot_Exp, -Liquor_Stores_Cap_Out);

Total_Util_Current_Exp = sum(Total_Util_Total_Exp, -Total_Util_Inter_Exp, -Total_Util_Cap_Outlay);

Water_Util_Current_Exp = sum(Water_Util_Total_Exp, -Water_Util_Inter_Exp, -Water_Util_Cap_Outlay);

Elec_Util_Current_Exp = sum(Elec_Util_Total_Exp, -Elec_Util_Inter_Exp, -Elec_Util_Cap_Outlay);

Gas_Util_Current_Exp = sum(Gas_Util_Total_Exp, -Gas_Util_Inter_Exp, -Gas_Util_Cap_Outlay);

Trans_Util_Current_Exp = sum(Trans_Util_Total_Exp, -Trans_Util_Inter_Exp, -Trans_Util_Cap_Outlay);

**run**;

*Set the order of the variables in the largest data set. Only the variables to be placed at the front of the data set need to be listed in the retain statement.;

*Also drop any variables that are unreliable according to the census, or that are both unneeded and unavailable in the newest data.;

**Data** LargestData (Drop= SortCode Census_Region Weight YearDepSch YearRetire Version ReviseDate Data_Flag JacketUnit ZeroData Imputed_Record);

Retain SurveyYr Year4 YearofData ID IDChanged State_Code Type_Code County Name FIPS_Code_State FYEndDate YearPop SchLevCode Population FunctionCode Enrollment SurveyYr Year4 ID State_Code Type_Code County Name FIPS_Code_State FYEndDate YearPop SchLevCode Population Total_Revenue Total_Rev_Own_Sources General_Revenue Gen_Rev_Own_Sources Total_Taxes Property_Tax Tot_Sales___Gr_Rec_Tax Total_Gen_Sales_Tax Total_Select_Sales_Tax Alcoholic_Beverage_Tax Amusement_Tax Insurance_Premium_Tax Motor_Fuels_Tax Pari_mutuels_Tax Public_Utility_Tax Tobacco_Tax Other_Select_Sales_Tax Total_License_Taxes Alcoholic_Beverage_Lic

Amusement_License Corporation_License Hunting___Fishing_License Motor_Vehicle_License Motor_Veh_Oper_License Motor_Vehicle_License_Total Public_Utility_License Occup_and_Bus_Lic_NEC Other_License_Taxes Total_Income_Taxes Individual_Income_Tax Corp_Net_Income_Tax Death_and_Gift_Tax Docum_and_Stock_Tr_Tax Severance_Tax Taxes_NEC Total_IG_Revenue Total_Fed_IG_Revenue Fed_IGR_Air_Transport Fed_IGR_Education Fed_IGR_Emp_Sec_Adm Fed_IGR_Gen_Rev_Shar Fed_IGR_Gen_Support Fed_IGR_Health___Hos Fed_IGR_Highways

Fed_IGR_Transit_Sub Fed_IGR_Hous_Com_Dev Fed_IGR_Natural_Res Fed_IGR_Public_Welf Fed_IGR_Sewerage Fed_IGR_Other Total_State_IG_Revenue State_IGR_Education State_IGR_Tax_Relief State_IGR_Oth_Gen_Sup State_IGR_Gen_Sup State_IGR_Health___Hos State_IGR_Highways State_IGR_Transit_Sub State_IGR_Hous_Com_Dev State_IGR_Public_Welf State_IGR_Sewerage State_IGR_Other Tot_Local_IG_Rev Local_IGR_InterSchool_Aid Local_IGR_Other_Education Local_IGR_Oth_Gen_Sup Local_IGR_Health___Hos Local_IGR_Highways

Local_IGR_Transit_Sub Local_IGR_Hous_Com_Dev Local_IGR_Public_Welf Local_IGR_Sewerage Local_IGR_Other Tot_Chgs_and_Misc_Rev Total_General_Charges Chg_Air_Transportation Chg_Misc_Com_Activ Chg_Total_Education Chg_Total_Elem_Education Chg_Elem_Ed_Sch_Lunch Chg_Elem_Ed_Tuition Chg_Elem_Ed_NEC Chg_Total_High_Ed Chg_Hospitals Chg_Highways Chg_Regular_Highways Chg_Toll_Highways Chg_Housing___Comm_Dev Chg_Total_Nat_Res Chg_Parking Chg_Parks___Recreation Chg_Sewerage Chg_Solid_Waste_Mgmt

Chg_Water_Transport Chg_All_Other_NEC Misc_General_Revenue Special_Assessments Prop_Sale_Total Prop_Sale_Hous_Com_Dev Prop_Sale_Other Interest_Revenue Fines_and_Forfeits Rents_and_Royalties Net_Lottery_Revenue Misc_General_Rev_NEC Liquor_Stores_Revenue Total_Utility_Revenue Water_Utility_Revenue Electric_Utility_Rev Gas_Utility_Rev Transit_Utility_Rev Total_Insur_Trust_Rev Total_Insur_Trust_Ctrb Tot_Ins_Trust_Inv_Rev Total_Emp_Ret_Rev

Emp_Ret_Total_Ctrib Emp_Ret_Loc_Emp_Ctrib Emp_Ret_Loc_To_Loc_Sys Emp_Ret_From_Other_Gov Emp_Ret_Sta_To_Sta_Ctr Emp_Ret_Int_Rev Emp_Ret_Other_Earnings Total_Unemp_Rev Unemp_Payroll_Tax Unemp_Int_Revenue Unemp_Federal_Advances Total_Expenditure Total_IG_Expenditure Direct_Expenditure Total_Current_Expend Total_Current_Oper Total_Capital_Outlays Total_Construction Total_Other_Capital_Outlays Tot_Assist___Subsidies Total_Interest_on_Debt Total_Insur_Trust_Ben Total_Salaries___Wages

General_Expenditure IG_Exp_To_State_Govt IG_Exp_To_Local_Govts IG_Exp_To_Federal_Govt Direct_General_Expend General_Current_Expend General_Current_Oper General_Capital_Outlay General_Construction General_Capital_Outlay_Other General_Assist___Sub General_Debt_Interest Air_Trans_Total_Expend Air_Trans_Direct_Expend Air_Trans_Cap_Outlay Air_Trans_Current_Exp Air_Trans_Construction Air_Trans_IG_To_State Air_Trans_IG_Local_Govts Misc_Com_Activ_Tot_Exp Misc_Com_Activ_Cap_Out Misc_Com_Activ_Current_Exp

Misc_Com_Activ_Constr Correct_Total_Exp Correct_Direct_Exp Correct_Cap_Outlay Correct_Current_Exp Correct_Construct Correct_IG_To_St Correct_IG_Loc_Govts Total_Educ_Total_Exp Total_Educ_Direct_Exp Total_Educ_Assist___Sub Total_Educ_Cap_Outlay Total_Educ_Current_Exp Total_Educ_Construct Elem_Educ_Total_Exp Elem_Educ_Direct_Exp Elem_Educ_Cap_Outlay Elem_Educ_Current_Exp Elem_Educ_Construction Elem_Educ_IG_To_State Elem_Educ_IG_Local_Govts Elem_Educ_IG_Sch_to_Sch Higher_Ed_Total_Exp Higher_Ed_Direct_Exp Higher_Ed_Cap_Outlay

Higher_Ed_Current_Exp Higher_Ed_Construct Higher_Ed_IG_To_St Higher_Ed_IG_Loc_Govts Educ_NEC_Total_Expend Educ_NEC_Direct_Expend Educ_NEC_Assistance Educ_NEC_Cap_Outlay Educ_NEC_Current_Exp Educ_NEC_Construction Educ_NEC_IG_To_State Educ_NEC_IG_Local_Govts Emp_Sec_Adm_Direct_Exp Emp_Sec_Adm_Cap_Outlay Emp_Sec_Adm_Current_Exp Emp_Sec_Adm_Construct Fin_Admin_Total_Exp Fin_Admin_Direct_Exp Fin_Admin_Cap_Outlay Fin_Admin_Current_Exp Fin_Admin_Construction Fin_Admin_IG_To_State Fin_Admin_IG_Local_Govts Fire_Prot_Total_Expend Fire_Prot_Direct_Exp

Fire_Prot_Cap_Outlay Fire_Prot_Current_Exp Fire_Prot_Construction Fire_Prot_IG_To_State Fire_Prot_IG_Local_Govts Judicial_Total_Expend Judicial_Direct_Expend Judicial_Cap_Outlay Judicial_Current_Exp Judicial_Construction Judicial_IG_To_State Judicial_IG_Local_Govts Cen_Staff_Total_Expend Cen_Staff_Direct_Exp Cen_Staff_Cap_Outlay Cen_Staff_Current_Exp Cen_Staff_Construction Cen_Staff_IG_To_State Cen_Staff_IG_Local_Govts Gen_Pub_Bldg_Total_Exp Gen_Pub_Bldg_Cap_Out Gen_Pub_Bldg_Current_Exp Gen_Pub_Bldg_Construct

Health_Total_Expend Health_Direct_Expend Health_Capital_Outlay Health_Current_Exp Health_Construction Health_IG_To_State Health_IG_Local_Govts Total_Hospital_Total_Exp Total_Hospital_Dir_Exp Total_Hospital_Cap_Out Total_Hospital_Current_Exp Total_Hospital_Construct Total_Hospital_IG_To_State Total_Hospital_IG_Loc_Govts Own_Hospital_Total_Exp Own_Hospital_Cap_Out Own_Hospital_Current_Exp Own_Hospital_Construct Hosp_Other_Total_Exp Hosp_Other_Direct_Exp Hosp_Other_Cap_Outlay Hosp_Other_Current_Exp Hosp_Other_Construct Hosp_Other_IG_To_State

Hosp_Other_IG_Loc_Govts Total_Highways_Tot_Exp Total_Highways_Dir_Exp Total_Highways_Cap_Out Total_Highways_Current_Exp Total_Highways_Construct Regular_Hwy_Total_Exp Regular_Hwy_Direct_Exp Regular_Hwy_Cap_Outlay Regular_Hwy_Current_Exp Regular_Hwy_Construct Regular_Hwy_IG_To_Sta Regular_Hwy_IG_Loc_Govts Toll_Hwy_Total_Expend Toll_Hwy_Cap_Outlay Toll_Hwy_Current_Exp Toll_Hwy_Construction Transit_Sub_Total_Exp Transit_Sub_Direct_Sub Transit_Sub_IG_To_Sta Transit_Sub_IG_Loc_Govts Transit_Sub_To_Own_Sys Hous___Com_Total_Exp

Hous___Com_Direct_Exp Hous___Com_Cap_Outlay Hous___Com_Current_Exp Hous___Com_Construct Hous___Com_IG_To_State Hous___Com_IG_Loc_Govts Libraries_Total_Expend Libraries_Direct_Exp Libraries_Cap_Outlay Libraries_Current_Exp Libraries_Construction Libraries_IG_To_State Libraries_IG_Local_Govts Natural_Res_Total_Exp Natural_Res_Direct_Exp Natural_Res_Cap_Outlay Natural_Res_Current_Exp Natural_Res_Construct Natural_Res_IG_To_Sta Natural_Res_IG_Loc_Govts Parking_Total_Expend Parking_Direct_Expend Parking_Capital_Outlay

Parking_Current_Exp Parking_Construction Parking_IG_To_State Parking_IG_Local_Govts Parks___Rec_Total_Exp Parks___Rec_Direct_Exp Parks___Rec_Cap_Outlay Parks___Rec_Current_Exp Parks___Rec_Construct Parks___Rec_IG_To_Sta Parks___Rec_IG_Loc_Govts Police_Prot_Total_Exp Police_Prot_Direct_Exp Police_Prot_Cap_Outlay Police_Prot_Current_Exp Police_Prot_Construct Police_Prot_IG_To_Sta Police_Prot_IG_Loc_Govts Prot_Insp_Total_Exp Prot_Insp_Direct_Exp Prot_Insp_Cap_Outlay Prot_Insp_Current_Exp Prot_Insp_Construction Prot_Insp_IG_To_State

Prot_Insp_IG_Local_Govts Public_Welf_Total_Exp Public_Welf_Direct_Exp Public_Welf_Cash_Asst Public_Welf_Cap_Outlay Public_Welf_Current_Exp Public_Welf_Construct Welf_Categ_Total_Exp Welf_Categ_Cash_Assist Welf_Categ_IG_To_State Welf_Categ_IG_Loc_Govts Welf_Cash_Total_Exp Welf_Cash_Cash_Assist Welf_Cash_IG_Local_Govts Welf_Vend_Pmts_Medical Welf_Vend_Pmts_NEC Welf_State_Share_Part_D Welf_Ins_Total_Exp Welf_Ins_Cap_Outlay Welf_Ins_Current_Exp Welf_Ins_Construction Welf_NEC_Total_Expend

Welf_NEC_Direct_Expend Welf_NEC_Cap_Outlay Welf_NEC_Current_Exp Welf_NEC_Construction Welf_NEC_IG_To_State Welf_NEC_IG_Local_Govts Sewerage_Total_Expend Sewerage_Direct_Expend Sewerage_Cap_Outlay Sewerage_Current_Exp Sewerage_Construction Sewerage_IG_To_State Sewerage_IG_Local_Govts SW_Mgmt_Total_Expend SW_Mgmt_Direct_Expend SW_Mgmt_Capital_Outlay SW_Mgmt_Current_Exp SW_Mgmt_Construction SW_Mgmt_IG_To_State SW_Mgmt_IG_Local_Govts Water_Trans_Total_Exp Water_Trans_Direct_Exp Water_Trans_Cap_Outlay Water_Trans_Current_Exp Water_Trans_Construct

Water_Trans_IG_To_Sta Water_Trans_IG_Loc_Govts Interest_on_Gen_Debt General_NEC_Total_Exp General_NEC_Direct_Exp VetBonus General_NEC_Cap_Outlay General_NEC_Current_Exp General_NEC_Construct General_NEC_IG_To_St General_NEC_IG_Loc_Govts General_NEC_IG_To_Fed Liquor_Stores_Tot_Exp Liquor_Stores_Cap_Out Liquor_Stores_Current_Exp Liquor_Stores_Constr Total_Util_Total_Exp Total_Util_Inter_Exp Total_Util_Cap_Outlay Total_Util_Current_Exp Total_Util_Construct Water_Util_Total_Exp Water_Util_Inter_Exp

Water_Util_Cap_Outlay Water_Util_Current_Exp Water_Util_Construct Elec_Util_Total_Exp Elec_Util_Inter_Exp Elec_Util_Cap_Outlay Elec_Util_Current_Exp Elec_Util_Construct Gas_Util_Total_Exp Gas_Util_Inter_Exp Gas_Util_Cap_Outlay Gas_Util_Current_Exp Gas_Util_Construct Trans_Util_Total_Exp Trans_Util_Inter_Exp Trans_Util_Cap_Outlay Trans_Util_Current_Exp Trans_Util_Construct Emp_Ret_Total_Expend Emp_Ret_Benefit_Paymts Emp_Ret_Withdrawals Emp_Ret_Other_Paymts Unemp_Comp_Total_Exp Unemp_Comp_Ben_Paymts Unemp_Ext___Spec_Pmts

Total_Debt_Outstanding Total_Long_Term_Debt_Out ST_Debt_End_of_Year Total_Beg_LTD_Out Beg_LTD_Out_Private_Purp Beg_LTD_Out_All_Other Beg_LTD_Out_Utility Beg_LTD_Out_Water_Util Beg_LTD_Out_Elec_Util Beg_LTD_Out_Gas_Util Beg_LTD_Out_Trans_Util Beg_LTD_Out_General Beg_LTD_Out_Education Beg_LTD_Out_Priv_Purp Beg_LTD_Out_Other_NEC Total_LTD_Issued LTD_Iss_Private_Purp LTD_Iss_All_Other LTD_Iss_Utility LTD_Iss_Util_Water LTD_Iss_Util_Electric

LTD_Iss_Util_Gas_Supply LTD_Iss_Util_Transit LTD_Iss_General LTD_Iss_Gen_Elem_Educ LTD_Iss_Gen_Other_Educ LTD_Iss_Gen_Other_NEC Total_LTD_Iss_FFC LTD_Iss_FFC_Utility LTD_Iss_FFC_Water_Util LTD_Iss_FFC_Elec_Util LTD_Iss_FFC_Gas_Util LTD_Iss_FFC_Trans_Util LTD_Iss_FFC_General LTD_Iss_FFC_Elem_Educ LTD_Iss_FFC_Other_Educ LTD_Iss_FFC_Other_NEC Total_LTD_Iss_NG LTD_Iss_NG_Utility LTD_Iss_NG_Water_Util LTD_Iss_NG_Elec_Util LTD_Iss_NG_Gas_Util

LTD_Iss_NG_Trans_Util LTD_Iss_NG_General LTD_Iss_NG_Elem_Educ LTD_Iss_NG_Other_Educ LTD_Iss_NG_Private_Purp LTD_Iss_NG_Other_NEC Total_LTD_Iss_Unsp LTD_Iss_Unsp_Utility LTD_Iss_Unsp_Water_Util LTD_Iss_Unsp_Elec_Util LTD_Iss_Unsp_Gas_Util LTD_Iss_Unsp_Trans_Util LTD_Iss_Unsp_General LTD_Iss_Unsp_Elem_Educ LTD_Iss_Unsp_Other_Educ LTD_Iss_Unsp_Other_NEC Total_LTD_Retired LTD_Ret_Private_Purp LTD_Ret_All_Other LTD_Ret_Utility LTD_Ret_Util_Water

LTD_Ret_Util_Electric LTD_Ret_Util_Gas_Supply LTD_Ret_Util_Transit LTD_Ret_General LTD_Ret_Gen_Elem_Educ LTD_Ret_Gen_Other_Educ LTD_Ret_Gen_Other_NEC Total_LTD_Ret_FFC LTD_Ret_FFC_Utility LTD_Ret_FFC_Water_Util LTD_Ret_FFC_Elec_Util LTD_Ret_FFC_Gas_Util LTD_Ret_FFC_Trans_Util LTD_Ret_FFC_General LTD_Ret_FFC_Elem_Educ LTD_Ret_FFC_Other_Educ LTD_Ret_FFC_Other_NEC Total_LTD_Ret_NG LTD_Ret_NG_Utility LTD_Ret_NG_Water_Util LTD_Ret_NG_Elec_Util

LTD_Ret_NG_Gas_Util LTD_Ret_NG_Trans_Util LTD_Ret_NG_General LTD_Ret_NG_Elem_Educ LTD_Ret_NG_Other_Educ LTD_Ret_NG_Private_Purp LTD_Ret_NG_Other_NEC Total_LTD_Ret_Unsp LTD_Ret_Unsp_Utility LTD_Ret_Unsp_Water_Util LTD_Ret_Unsp_Elec_Utili LTD_Ret_Unsp_Gas_Util LTD_Ret_Unsp_Trans_Util LTD_Ret_Unsp_General LTD_Ret_Unsp_Elem_Educ LTD_Ret_Unsp_Other_Educ LTD_Ret_Unsp_Other_NEC Total_LTD_Out LTD_Out_Private_Purp LTD_Out_All_Other Total_LTD_Out_Utility

LTD_Out_Util_Water LTD_Out_Util_Electric LTD_Out_Util_Gas_Supply LTD_Out_Util_Transit LTD_Out_General LTD_Out_Gen_Elem_Educ LTD_Out_Gen_Other_Educ LTD_Out_Gen_Other_NEC Total_LTD_Out_FFC LTD_Out_FFC_Utility LTD_Out_FFC_Water_Util LTD_Out_FFC_Elec_Util LTD_Out_FFC_Gas_Util LTD_Out_FFC_Trans_Util LTD_Out_FFC_General LTD_Out_FFC_Elem_Educ LTD_Out_FFC_Other_Educ LTD_Out_FFC_Other_NEC Tot_LTD_Out_NG LTD_Out_NG_Utility LTD_Out_NG_Water_Util

LTD_Out_NG_Elec_Util LTD_Out_NG_Gas_Util LTD_Out_NG_Trans_Util LTD_Out_NG_General LTD_Out_NG_Elem_Educ LTD_Out_NG_Other_Educ LTD_Out_NG_Private_Purp LTD_Out_NG_Other_NEC Total_Cash___Securities Insur_Trust_Cash___Sec Emp_Retire_Cash___Sec Emp_Retire_Cash___Dep Emp_Retire_Total_Sec Emp_Retire_Sec_Tot_Fed Emp_Retire_Sec_S_L_Secur Emp_Retire_Sec_Tot_Nong Emp_Retire_Sec_Corp_Bds Emp_Retire_Sec_Corp_Stk Emp_Retire_Sec_Mortgages

Emp_Retire_Sec_Misc_Inv Emp_Retire_Sec_Oth_Nong Unemp_Comp_Cash___Sec Unemp_Comp_Bal_In_US_Trs Unemp_Comp_Other_Balance Nonin_Trust_Cash___Sec Sinking_Fd_Cash___Sec Bond_Fd_Cash___Sec Oth_Nonin_Fd_Cash___Sec FunctionCode Enrollment;

Set LargestData;

**run**;

* Write the final file to a delinimated text file, change this path to define a valid location for the file on your system;

**Proc** **Export** Data=work.LargestData

outfile='E:/CensusData/AllCensusData.csv'

dbms=CSV

replace;

**run**;
