# Supplementary material for: The Government Finance Database: A Common Resource for Quantitative Research in Public Financial Analysis
Source: PLoS One. 2015 Jun 24;10(6):e0130119. doi: 10.1371/journal.pone.0130119 (PMC4479543; doi:10.1371/journal.pone.0130119)
Supplement: S2 File — This file includes the state data from the Government Finance Database at the time of publication, the appendix for the Government Finance Database, and the U.S. Census 2006 Classification manual. For more up to date versions of the data please visit http://www.willamette.edu/mba/research_impact/public_datasets/. (ZIP) [file pone.0130119.s002.zip › 2006_classification_manual.pdf]

U.S. Bureau of the Census

Government Finance and Employment

Classification Manual

Covering the activities of the Federal, state, and local governments.

Effective with the fiscal year 2005 finance and 2006 employment surveys.

October 2006

PREFACE

This is the 2006 edition of the U. S. Census Bureau's *Government Finance and Employment Classification Manual.* It contains the statistical classification categories and definitions that are applicable to the Census Bureau's statistical programs covering government finances and public employment. These include the census of governments, as well as other annual and quarterly surveys of government finance and employment. These statistical programs are conducted by the Census Bureau's Governments Division.

The census of governments is one component of the nation's economic census and is required by the United States Codes, Title 13, Section 161. The annual and quarterly surveys are authorized by Title 13, Section 182 of the U.S. Code. The U.S. Census Bureau, which conducts the economic census every five years, is an agency of the Department of Commerce.

This edition of the *Classification Manual* reflects revisions to classification categories that were the result of two major initiatives. First, the Governments Division revised several major concepts and categories about public employee retirement systems, effective with the 2002 Census of Governments. These revisions were approved by the U.S. Office of Management and Budget, as required. Second, the Governments Division, in conjunction with the Economic Statistical Methods and Programming Division (also a major division of the U.S. Census Bureau) initiated a redesign of the government finance program in 2004, to be effective with the statistical surveys for survey year 2005. The objective was to ensure that the government finance programs met new statistical standards for census and sample surveys, that were being adopted by the Office of Management and Budget. This initiative led to a significant number of changes in the classification system.

This Classification Manual is produced in limited volume in print (hard copy) format. It is being maintained in its full format on the Census Bureau's Internet site. It will be updated regularly, especially the tables pertaining to statistical code applicability, by survey. Users should refer to the Internet site at www.census.gov for information on this and other Census Bureau programs, as well a for information on how to contact the Governments Division to obtain other information about the census of governments and related statistical programs.

October, 2006

Summarized Table of Contents

Introduction

Part 1. The Framework of Census Bureau Statistics on Governments

Chapter 1. The Basic Unit of Reporting – The Government Entity
Introduction
1.1 Types of Government Entities
1.2 Definition of a Government Entity
1.3 The Parent Government Concept
1.4 Dependent Agencies
1.5 Joint Activities of Governments
1.6 Government Organization – The Government Sector
1.7 The Governments Integrated Directory (GID)
1.8 Government Organization Reference Codes
1.9 Tables

Chapter 2. Classification System Overview – Government Sectors and Types of Statistics
Introduction
2.1 The Four Sectors of Government Activity
2.2 The Four Types of Census Bureau Statistics
2.3 Special Topics: How Census Bureau Statistics on Governments are Developed

Part 2. Government Finance Statistics

Chapter 3. The Foundation for Census Bureau Financial Statistics on Governments
Introduction
3.1 Primary Basis of Government Finance Statistics
3.2 Fiscal Years as Statistical Reporting Periods
3.3 Current Dollars
3.4 Accounting Basis
3.5 Four Sectors of Government from a Finance Viewpoint
3.6 Noncash Transactions
3.7 Intergovernmental Transactions
3.8 Netting Out Duplicative Intergovernmental Transactions
3.9 Internal Transfers
3.10 Agency, Private Trust, Investment, and Other Transactions
3.11 Miscellaneous Topics
3.12 Special Topics: Statistical Nature of Data
3.13 Special Topics: Classification Issues for Washington, DC
3.14 Joint Financial Activities of Governments
3.15 Tables

Chapter 4. Revenue
Introduction
4.1 Revenue Definition
4.2 The Four Sectors of Government – Revenue Issues
4.3 General Revenue

4.4 Liquor Store Revenue
4.5 Utility Revenue
4.6 Social Insurance Trust Revenue
4.7 Exhibit Statistics for Revenue – Revenue versus Receipts
4.8 Explanation of Statistical Codes Used to Identify Revenue Categories
4.9 Description Pages for Revenue Categories
4.10 Tables

Chapter 5. Expenditure
Introduction
5.1 Expenditure Definition
5.2 Cross Classification of Expenditure – Function, Character, and Object Definitions
5.3 The Four Sectors of Government – Expenditure Issues
5.4 Measurement Issues for Expenditure
5.5 Types of Expenditure Statistics – Regular, Exhibit, and Derived
5.6 Functional Categories for Regular Finance Expenditure Statistics
5.7 Tables

Chapter 6. Indebtedness
Introduction
6.1 Concepts and Definitions
6.2 Measurement Issues for Debt Statistics
6.3 Statistical Classification Categories for Debt
6.4 Topical Issues Related to Statistics on Government Debt
6.5 Tables

Chapter 7. Cash and Security Holdings
Introduction
7.1 Cash and Security Holdings Definition
7.2 Measurement Issues
7.3 Special Topics: Assets of State Lottery Systems
7.4 Special Topics: Federal Government Cash and Security Holding Statistics
7.5 Special Topics: Liquor Stores Cash and Security Holdings
7.6 Statistical Classification Categories for Cash and Security Holdings
7.7 Description of Cash and Security Holdings Categories
7.8 Tables

Chapter 8. Public Employee Retirement Systems
Introduction
8.1 Public Employee Retirement Systems Definition
8.2 Regular Finance Statistics, Exhibit Finance Statistics, and Descriptive Statistics – Explanations
8.3 Measurement Issues for Public Employee Retirement System Statistics
8.4 Public Employee Retirement System Finances – Basic Concepts
8.5 Federal Government Public Employee Retirement System Statistics
8.6 Description Pages for Public Employee Retirement System Statistics
8.7 Tables

Chapter 9. Federal and State Social Insurance Trust Systems, Other Than Public Employee Retirement
Introduction
9.1 Social Insurance Trust System Definition
9.2 Types of Insurance Trust Systems
9.3 Regular Finance Statistics and Exhibit Finance Statistics – Explanations
9.4 Measurement Issues for Social Insurance Trust System Statistics
9.5 Federal and State Social Insurance Trust System Finances – Basic Concepts
9.6 Special Topics
9.7 Description Pages for Federal and State Social Insurance Trust System Statistics
9.8 Tables

Chapter 10. Liquor Stores and Lotteries – All Statistics
Introduction
10.1 Liquor Stores Statistics – Overview
10.2 Liquor Stores Definition
10.3 How Liquor Stores are Treated in Regular Finance Statistics
10.4 Liquor Stores Exhibit Statistics and Derived Statistics
10.5 Description Pages for Liquor Stores
10.6 Lottery System Statistics – Overview
10.7 Lottery System Definitions
10.8 How Lotteries are Treated in Regular Finance Statistics
10.9 Exhibit Statistics for Lotteries – Receipts and Expenses
10.10 Description Pages for Lottery Systems
10.11 Applicability of Liquor Stores and Lottery Codes by Level and Type of Government
10.12 Tables

Part 3. Public Employment and Payroll Statistics

Chapter 11. Overview of the Public Employment Statistics
11.1 Survey Coverage and Definitions
11.2 Employment Statistics for the Federal Government
11.3 Reporting Periods
11.4 Joint Activities of Governments
11.5 Special Topics: Classification Issues for Washington, DC
11.6 Special Topics: Additional Statistics During Census of Governments

Chapter 12. Functional Categories for Employment Statistics
Introduction
12.1 Overview
12.2 Key to the Employment Function Pages
12.3 Description Pages for Employment Functions
12.4 Tables

Part 4. Appendices

Appendix 1. Modifications Effective with this Edition of the Classification Manual
Appendix 1.1 Revisions to Presentation and Format
Appendix 1.2 Revisions to Public Employment Statistical Classifications
Appendix 1.3 Summary of Revisions to Government Finance Classifications
Appendix 1.4 Revisions to Definitions

Appendix 1.5 Revisions to Classification Categories and Codes for Statistics on Public Employee Retirement Systems
Appendix 1.6 Revisions from the 2005 Redesign of the Government Finance Program
Appendix 1.7 Coverage Changes for Washington, DC

Appendix 2. Valid Codes Tables

Detailed Table of Contents

Preface
Summarized Table of Contents
Detailed Table of Contents
List of Charts and Tables
Guide to Symbols

Introduction

Part 1. The Framework of Census Bureau Statistics on Governments

Chapter 1. The Basic Unit of Reporting – The Government Entity
Introduction
1.1 Types of Government Entities
1.2 Definition of a Government Entity
1.2.1 Other Factors Used in Defining Governments
1.2.2 Noncritical Characteristics Used in Defining Governments
1.3 The Parent Government Concept
1.4 Dependent Agencies
1.5 Joint Activities of Governments
1.6 Government Organization – The Government Sector
1.6.1 Statistical Aggregation and Tabulation
1.7 The Governments Integrated Directory (GID)
1.8 Government Organization Reference Codes
1.8.1 Government Unit Codes
1.8.1.1 Special Topics: Government Unit Code Historical Assignments
1.8.2 Government Organization Function Codes
1.8.3 Government Organization Activity Codes
1.8.4 Government Organization School Level Codes
1.8.5 ID Codes for Files of Aggregated Individual Government Records
1.9 Tables
Table 1.1 Government Organization Function and Activity Codes
Table 1.2 Government Organization Function Codes and Their Associated Finance Expenditure Codes

Chapter 2. Classification System Overview – Government Sectors and Types of Statistics
Introduction
2.1 The Four Sectors of Government Activity
2.1.1 General Government Sector
2.1.2 Utilities Sector
2.1.3 Liquor Stores Sector
2.1.4 Social Insurance Trust Sector
2.1.5 Summary Chart: The Four Sectors of Government Activity
2.2 The Four Types of Census Bureau Statistics
2.2.1 Regular Statistics
2.2.2 Exhibit Statistics
2.2.3 Derived Statistics
2.2.4 Descriptive Statistics
2.3 Special Topics: How Census Bureau Statistics on Governments are Developed

2.3.1 Mail Canvass
2.3.2 Compilation
2.3.3 Central Collection
2.3.4 Calculated Statistics
2.3.5 Imputation

Part 2. Government Finance Statistics

Chapter 3. The Foundation for Census Bureau Financial Statistics on Governments
Introduction
3.1 Primary Basis of Government Finance Statistics
3.2 Fiscal Years as Statistical Reporting Periods
3.3 Current Dollars
3.4 Accounting Basis
3.4.1 Accounting Funds (or Entities)
3.4.2 Cash versus Accrual Basis of Accounting
3.4.3 Special Topics: Governmental Accounting Standards Board (GASB)
3.5 Four Sectors of Government from a Finance Viewpoint
3.6 Noncash Transactions
3.7 Intergovernmental Transactions
3.7.1 Intergovernmental Fiscal Relations (Type 1)
3.7.2 Fiscal Agents for Other Governments (Type 2)
3.7.3 Purchases from Other Governments (Type 3)
3.8 Netting Out Duplicative Intergovernmental Transactions
3.8.1 Special Topics: Payments to Other Government Social Insurance Trust Systems
3.9 Internal Transfers
3.9.1 Interfund Transactions
3.9.2 Intragovernmental Service Funds (Revolving Funds)
3.9.3 Interdepartmental Charges
3.10 Agency, Private Trust, Investment, and Other Transactions
3.10.1 Agency Transactions
3.10.2 Private Trust Transactions
3.10.3 Investment Transactions
3.10.3.1 Special Topics: Investment Transactions for Public Employee Retirement Systems.
3.10.4 Adjustment and Correction Transactions
3.11 Miscellaneous Topics
3.11.1 Suspense Transactions
3.11.2 Capital and Operating Leases
3.11.3 Depreciation
3.11.4 Anomalies of Government Finance Statistics
3.12 Special Topics: Statistical Nature of Data
3.13 Special Topics: Classification Issues for Washington, DC
3.14 Joint Financial Activities of Governments
3.15 Tables
Table 3.1 Summary of Government Fiscal Year Ending Dates
Table 3.2 Examples of Accounting Funds and Their Treatment in Census Bureau Statistics on Government Finances

Chapter 4. Revenue
Introduction
4.1 Revenue Definition
4.1.1 Coverage Issues: Revenue
4.1.2 Measurement Issues: Revenue
4.1.2.1 Measurement Issues: Refunds and Correcting Transactions
4.1.2.2 Measurement Issues: Timing
4.1.2.3 Measurement Issues: Aggregation and Tabulation
4.1.2.4 Measurement Issues: Government Enterprises Activities
4.1.3 Exclusions from the Revenue Definition
4.2 The Four Sectors of Government – Revenue Issues
4.3 General Revenue
4.3.1 Taxes
4.3.1.1 Special Topics: Assignment of Tax Revenue
4.3.1.2 Refunds of Taxes
4.3.1.3 Taxes on Government Utilities
4.3.2 Intergovernmental Revenue
4.3.3 Current Charges
4.3.4 Miscellaneous General Revenue
4.4 Liquor Store Revenue
4.5 Utility Revenue
4.5.1 Special Topics: Intergovernmental Revenue Codes for Utilities
4.6 Social Insurance Trust Revenue
4.7 Exhibit Statistics for Revenue – Revenue versus Receipts
4.8 Explanation of Statistical Codes Used to Identify Revenue Categories
4.9 Description Pages for Revenue Categories
4.10 Tables
Table 4.1 Revenue Code Validity by Level and Type of Government: General Revenue
Table 4.2 Revenue Code Validity by Level and Type of Government: Liquor Stores and Utilities Sectors
Table 4.3 Revenue Code Validity by Level and Type of Government: Social Insurance Trust Sector

Chapter 5. Expenditure
Introduction
5.1 Expenditure Definition
5.1.1 Financial Transactions Excluded From the Definition of Expenditure
5.1.2 Refunds and Correcting Transactions
5.2 Cross Classification of Expenditure – Function, Character, and Object Definitions
5.2.1 Expenditure Function Definition
5.2.2 Expenditure Character and Object Definitions
5.2.2.1 Direct Expenditures Defined
5.2.2.2 Intergovernmental Expenditures Defined
5.3 The Four Sectors of Government – Expenditure Issues
5.3.1 General Expenditure
5.3.2 Utility Expenditure
5.3.2.1 Special Topics: Intergovernmental Expenditure Codes for Utilities
5.3.2.2 Special Topics: Joint Utility Projects (Public and Private)
5.3.3 Liquor Stores Expenditure
5.3.4 Social Insurance Trust Expenditure

5.4 Measurement Issues for Expenditure
5.4.1 Measurement Issues: Timing
5.4.2 Measurement Issues: Valuation
5.4.3 Measurement Issues: Aggregation and Tabulation
5.5 Types of Expenditure Statistics – Regular, Exhibit, and Derived
5.6 Functional Categories for Regular Finance Expenditure Statistics
5.6.1 Overview
5.6.2 Special Topics: Federal Government Expenditure Statistics
5.6.3 Key to the Expenditure Function Pages
5.7 Tables
Table 5.1 Description of Character and Object Categories
Table 5.2 Function and Object Code Validity, by Level and Type of Government

Chapter 6. Indebtedness
Introduction
6.1 Concepts and Definitions
6.1.1 Definitions – Debt Instruments
6.1.2 Liabilities Outside Census Bureau Definition
6.1.3 Definitions – Reporting Entity
6.1.4 Census Bureau Reporting and the Governmental Accounting Standards Board
6.1.5 Three Way Classification of Public Debt
6.1.6 Classification Changes Effective With 2005 Survey Year
6.2 Measurement Issues for Debt Statistics
6.2.1 Measurement Issues: Timing
6.2.2 Measurement Issues: Valuation
6.2.3 Measurement Issues: Aggregation and Tabulation
6.3 Statistical Classification Categories for Debt
6.4 Topical Issues Related to Statistics on Government Debt
6.4.1 Public Debt for Private Purposes
6.4.2 Refunding of Long-Term Debt
6.4.2.1 Regular Refunding
6.4.2.2 Advance Refunding
6.4.3 Zero Coupon and Other Deep Discount Bonds
6.4.4 Bond Banks and Pooled Debt
6.4.5 Leases and Lease-Purchase Agreements
6.4.6 Taxable Public Debt
6.4.7 Special Topics: Debt Statistics and the Four Sectors of Government
6.5 Tables
Table 6.1 Applicability of Debt Categories and Codes
Table 6.2 Historical Crosswalk: State and Local Debt Classification Categories

Chapter 7. Cash and Security Holdings
Introduction
7.1 Cash and Security Holdings Definition
7.1.1 Assets Outside of Census Bureau Definition
7.2 Measurement Issues
7.2.1 Measurement Issues: Timing
7.2.2 Measurement Issues: Valuation
7.3 Special Topics: Assets of State Lottery Systems
7.4 Special Topics: Federal Government Cash and Security Holding Statistics

7.5 Special Topics: Liquor Stores Cash and Security Holdings
7.6 Statistical Classification Categories for Cash and Security Holdings
7.7 Description of Cash and Securities Categories
7.8 Tables
Table 7.1 Examples of Assets Included and Excluded from Census Bureau Definition of Cash and Securities
Table 7.2 Applicability of Cash and Security Codes, by Level of Government

Chapter 8. Public Employee Retirement Systems
Introduction
8.1 Public Employee Retirement Systems Definition
8.1.1 Examples of Public Employee Retirement Systems Included by this Definition
8.1.2 Examples of Public Employee Retirement Systems Excluded by this Definition
8.2 Regular Finance Statistics, Exhibit Finance Statistics, and Descriptive Statistics – Explanations
8.2.1 Exhibit Statistics – Additional Detail
8.2.2 Exhibit Statistics – Enterprise Perspective
8.3 Measurement Issues for Public Employee Retirement System Statistics
8.3.1 Measurement Issues: Timing
8.3.2 Measurement Issues: Aggregation and Tabulation
8.3.3 Measurement Issues: Valuation
8.4 Public Employee Retirement System Finances – Basic Concepts
8.4.1 Public Employee Retirement Systems Revenue and Receipts
8.4.1.1 Unrealized Gains or Losses as Revenue
8.4.2 Public Employee Retirement Systems Expenditure and Expenses
8.4.3 Public Employee Retirement Systems Cash and Security Holdings
8.4.4 Special Topics: Purchase and Sale of Investments
8.4.5 Special Topics: “Internal” Investment Transactions
8.4.6 Special Topics: Loans and Borrowing Associated with Employee Retirement System Finances
8.4.6.1 Special Topics: Loans to Members
8.4.6.2 Special Topics: Loans to Parent Government
8.4.7 Special Topics: Fiscal Conditions of Retirement Systems
8.4.8 Special Topics: Retirement Revenue Gains and Losses
8.5 Federal Government Public Employee Retirement System Statistics
8.6 Description Pages for Public Employee Retirement System Statistics
8.7 Tables
Table 8.1 Applicability of Public Employee Retirement System Codes, by Level of Government
Table 8.2 Summary of Public Employee Retirement System Codes

Chapter 9. Federal and State Social Insurance Trust Systems, Other Than Public Employee Retirement
Introduction
9.1 Social Insurance Trust System Definition
9.1.1 Examples of Social Insurance Trust Systems Included by this Definition
9.1.2 Examples of Social Insurance Trust Systems Excluded by this Definition
9.2 Types of Insurance Trust Systems
9.2.1 Public Employee Retirement Systems
9.2.2 Unemployment Compensation Systems
9.2.3 Workers’ Compensation Systems
9.2.4 Other Federal and State Social Insurance Trust Systems
9.3 Regular Finance Statistics and Exhibit Finance Statistics – Explanations

9.3.1 Exhibit Statistics – Additional Detail
9.3.2 Exhibit Statistics – Enterprise Perspective
9.3.3 Other Federal and State Social Insurance Trust Systems – Exhibit Codes
9.4 Measurement Issues for Social Insurance Trust System Statistics
9.4.1 Measurement Issues: Timing
9.4.2 Measurement Issues: Aggregation and Tabulation
9.4.3 Measurement Issues: Valuation
9.5 Federal and State Social Insurance Trust System Finances – Basic Concepts
9.5.1 Social Insurance Trust Systems: Revenue and Receipts
9.5.2 Social Insurance Trust Systems: Expenditure and Expenses
9.5.3 Social Insurance Trust Systems: Cash and Security Holdings
9.6 Special Topics
9.6.1 Special Topics: Classifying Debt for Unemployment Compensation Funds
9.6.2 Special Topics: Loans to Parent Government by Social Insurance Trust Systems
9.6.3 Special Topics: "Internal" Investment Transactions
9.7 Description Pages for Federal and State Social Insurance Trust System Statistics
9.8 Tables
Table 9.1 Applicability of Other Social Insurance Trust System Codes, by Level of Government
Table 9.2 Summary of Federal and State Social Insurance Trust System Codes

Chapter 10. Liquor Stores and Lotteries – All Statistics
Introduction
10.1 Liquor Stores Statistics – Overview
10.2 Liquor Stores Definition
10.3 How Liquor Stores are Treated in Regular Finance Statistics
10.4 Liquor Stores Exhibit Statistics and Derived Statistics
10.5 Description Pages for Liquor Stores
10.6 Lottery System Statistics – Overview
10.7 Lottery System Definitions
10.8 How Lotteries are Treated in Regular Finance Statistics
10.8.1 Special Topics: How Lottery System Cash and Security Holdings are Reported in Regular Finance Statistics
10.9 Exhibit Statistics for Lotteries – Receipts and Expenses
10.10 Description Pages for Lottery Systems
10.11 Applicability of Liquor Stores and Lottery Codes by Level and Type of Government
10.12 Tables
Table 10.1 Liquor Stores and Lottery System Code Validity, by Level and Type of Government
Table 10.2 Summary of Liquor Stores and Lottery System Finance Codes and Statistics

Part 3. Public Employment and Payroll Statistics

Chapter 11. Overview of the Public Employment Statistics
Introduction
11.1 Survey Coverage and Definitions
11.1.1 Employment Statistics on State and Local Governments
11.1.2 Employees/Employment Definition
11.1.3 Full-Time and Part-Time Employees
11.1.4 Payrolls
11.1.5 Hours Worked by Part-Time Workers
11.1.6 Full-Time Equivalent Employment

11.1.6.1 Special Topics: Note Regarding the October 1986 Revision to Full-Time Equivalent Calculation Method
11.1.7 Average Monthly Earnings – Full-Time Employees
11.1.8 Standard Hours Worked
11.1.9 Pay Interval
11.2 Employment Statistics for the Federal Government
11.2.1 Federal Government – Employment By Function
11.2.2 Federal Government – Employment By State Area
11.3 Reporting Periods
11.4 Joint Activities of Governments
11.5 Special Topics: Classification Issues for Washington, DC
11.6 Special Topics: Additional Statistics During Census of Governments

Chapter 12. Functional Categories for Employment Statistics
Introduction
12.1 Overview
12.2 Key to the Employment Function Pages
12.3 Description Pages for Employment Functions
12.4 Tables
Table 12.1 Functional Categories Applicable to Employment Data
Table 12.2 Applicability of Employment Function Codes, by Level and Type of Government

Part 4. Appendices

Appendix 1. Modifications Effective with this Edition of the Classification Manual
Appendix 1.1 Revisions to Presentation and Format
Appendix 1.1.1 Separate Explanations of Government Finance and Public Employment
Appendix 1.1.2 Separate Presentation of Classification Categories for Public Employee Retirement Systems
Appendix 1.1.3 Introduction of Government Organization Topics
Appendix 1.1.4 Addition of Valid Code Tables
Appendix 1.2 Revisions to Public Employment Statistical Classifications
Appendix 1.3 Summary of Revisions to Government Finance Classifications
Appendix 1.4 Revisions to Definitions
Appendix 1.5 Revisions to Classification Categories and Codes for Statistics on Public Employee Retirement Systems
Appendix 1.6 Revisions from the 2005 Redesign of the Government Finance Program
Appendix 1.6.1 Changes to Object Codes for Expenditure Statistics
Appendix 1.6.1.1 Assistance and Subsidies
Appendix 1.6.1.2 Elimination of Selected Intergovernmental Object Codes
Appendix 1.6.1.3 Revisions to Object Code K
Appendix 1.6.2 Changes for Liquor and Lotteries
Appendix 1.6.3 Revenue Classification Category Revisions
Appendix 1.6.4 Expenditure Classification Category Revisions
Appendix 1.6.5 Debt Classification Category Revisions
Appendix 1.6.6 Cash and Security Classification Changes – Market Value
Appendix 1.7 Coverage Changes for Washington, DC
Appendix Table 1.1 Pre 2005 Basis of Measurement for Cash and Security Holdings

Appendix Table 1.2 State and Local Government Public Employee Retirement System Regular and

Exhibit Variables, Current and Historical

Appendix 2. Valid Finance Code Reference Tables
Appendix Table 2.1 Code Validity, by Level and Type of Government
Appendix Table 2.2 Cross-Classification of Expenditure Function and Object Codes
Appendix Table 2.3 Employment Code Validity, by Level and Type of Government

Topical Index

List of Charts and Tables

Chapter 1. The Basic Unit of Reporting – The Government Entity
- Chart 1.A Government Unit Code Assignments by Year
- Chart 1.B School Level Codes
- Table 1.1 Government Organization Function and Activity Codes
- Table 1.2 Government Organization Function Codes and Their Associated Finance Expenditure Codes

Chapter 2. The Foundation for Census Bureau Financial Statistics on Governments
- Chart 2.A The Four Sectors of Government Activity

Chapter 3. Overview of Government Finance Statistics
- Chart 3.A Rules for Reporting Refund and Correction Adjustments
- Table 3.1 Summary of Government Fiscal Year Ending Dates
- Table 3.2 Examples of Accounting Funds and Their Treatment in Census Bureau Statistics on Government Finances

Chapter 4. Revenue
- Table 4.1 Revenue Code Validity by Level and Type of Government: General Revenue
- Table 4.2 Revenue Code Validity by Level and Type of Government: Liquor Stores and Utilities Sectors
- Table 4.3 Revenue Code Validity by Level and Type of Government: Social Insurance Trust Sector

Chapter 5. Expenditure
- Chart 5.A Character and Object Categories
- Table 5.1 Description of Character and Object Categories
- Table 5.2 Function and Object Code Validity, by Level and Type of Government

Chapter 6. Indebtedness
- Chart 6.A Examples of Liabilities Included and Excluded from Census Bureau Definition of Public Debt
- Table 6.1 Applicability of Debt Categories and Codes
- Table 6.2 Historical Crosswalk: State and Local Debt Classification Categories

Chapter 7. Cash and Security Holdings
- Chart 7.A Statistical Classification Categories for Cash and Security Holdings
- Table 7.1 Examples of Assets Included and Excluded from Census Bureau Definition of Cash and Securities
- Table 7.2 Applicability of Cash and Security Codes, by Level and Type of Government

Chapter 8. Public Employee Retirement Systems
- Table 8.1 Applicability of Public Employee Retirement System Codes, By Level of Government
- Table 8.2 Summary of Public Employee Retirement System Codes

Chapter 9. Federal and State Social Insurance Trust Systems, Other Than Public Employee Retirement
- Table 9.1 Applicability of Other Social Insurance Trust System Codes, by Level of Government
- Table 9.2 Summary of Federal and State Social Insurance Trust System Codes

Chapter 10. Liquor Stores and Lotteries – All Statistics
Table 10.1 Liquor Stores and Lottery System Code Validity, by Level and Type of Government
Table 10.2 Summary of Liquor Stores and Lottery System Finance Codes and Statistics

Chapter 11. Overview of the Public Employment and Payrolls Statistics
Chart 11.A Sources of the Eight Employment Statistics
Chart 11.B Conversion Factors for Monthly Pay

Chapter 12. Functional Categories for Employment Statistics
Table 12.1 Functional Categories Applicable to Employment Data
Table 12.2 Applicability of Employment Function Codes, by Level and Type of Government

Appendix 1. Modifications Effective with this Edition of the Classification Manual
Appendix Table 1.1 Pre 2005 Basis of Measurement for Cash and Security Holdings
Appendix Table 1.2 State and Local Government Public Employee Retirement System Regular and Exhibit Variables, Current and Historical

Appendix 2. Valid Finance Code Reference Tables
Appendix Table 2.1 Code Validity, by Level and Type of Government
Appendix Table 2.2 Cross-Classification of Expenditure Function and Object Codes
Appendix Table 2.3 Employment Code Validity, by Level and Type of Government

GUIDE TO SYMBOLS

Every effort has been made to keep symbols, abbreviations, and acronyms to a minimum. Where used throughout this *Classification Manual*, they are defined generally at their instance of first use. Several commonly used references are noted and explained here:

NEC Not Elsewhere Classified

This phrase is used in context with the overall classification system, which contains hundreds of well defined finance and employment categories, functions, or groupings. NEC, in this context, refers to a general activity undertaken by governments that cannot fit into a category, function, or statistical group that is defined in this *Classification Manual*. In effect, it a miscellaneous or catch-all grouping.

X Not Applicable

This symbol always is used in conjunction with a category, function, or statistical grouping, and implies that the category does not apply where noted. If used in a table, for example, X would mean that the statistical code represented by a table cell does not exist (there is no such valid statistical classification for that combination of coding).

-- Indicates not applicable for type of statistic (used primarily in tables).

* Wildcard Code

This symbol is found frequently in combination with the three-character classification codes that are used to label the many categories in the statistical classification system. It refers to any code that could be inserted into the three-character combination. It takes a number of forms, such as:
*01 (meaning any object code that is valid for expenditure function 01),
X** (meaning any function code that is valid for the object code applicable to employee retirement statistics)
... and so forth.

... Does Not Exist

This symbol indicates that there is no code or content for the table cell in which it is found. It is commonly used in tables to indicate that there is no official statistical code for the type of financial category. The most common use is for descriptive statistical categories.

Valid This term is commonly used throughout the tables in this *Classification Manual*. It has a very specific meaning, namely that the table cell in which it is located contains a three-character statistical code combination for which statistics are collected and reported.

Pagination in the body of this *Classification Manual* is by chapter. Each page is numbered as 1-1, 1-2, 1-3, and so forth, until the final page of the chapter. Tables are included in the numbering scheme. The Internet version of this *Classification Manual* contains additional information showing the date of the last update or revision to a chapter.

INTRODUCTION

This edition of the *Classification Manual* retains the overall classification system that has been is use since 1951, but with modifications necessitated by the following events:

- Changes in accounting and reporting practices being utilized by state and local governments.

- Revisions to definitions used in the surveys covering public employee retirement systems, largely effective with the 2002 Census of Governments.

- Classification system changes, reflecting expansion of some categories and collapsing of others, that came about as a result of the Governments Division 2005 redesign initiative for the government finance statistical program. The most extensive of these involved the expansion of many tax categories and the collapse of most debt categories.

- A major change in the time period for coverage in the public employment surveys (explained in Chapter 11).

Appendix 1 summarizes the changes to the classification system that have occurred since the last edition of this *Classification Manual* was released in 1992.

The format of the *Classification Manual* itself has undergone a major modification. Specifically, this edition contains separate chapters covering the public employment surveys and the public employee retirement systems surveys, in their entirety. General information about the classifications used in both of those surveys was contained in earlier editions of this manual, but not as stand-alone chapters in their entirety.

For the public employment survey, this edition of this *Classification Manual* contains detail on all of the functional categories used for statistical classification. This is important because the functional categories used for government finance expenditures statistics and public employment survey statistics differ in many instances, in terms of their number, their applicability by level and type of government, and their definitions in a few, selected instances.

The separate chapter on public employee retirement systems in this edition of the *Classification Manual* attests to the growing importance of that group of surveys, as well as to the increasing complexity of trying to obtain accurate measurements about financial transactions of these systems.

PART 1. THE FRAMEWORK OF CENSUS BUREAU STATISTICS ON GOVERNMENTS

The U. S. Census Bureau conducts the nation's Economic Census every five years, in years ending in "2" and "7." The census of governments is one component of the economic census and is required by the United States Codes, Title 13, Section 161. The Census Bureau also conducts a number of recurrent annual and quarterly surveys related to the census of governments, authorized by Title 13, Section 182 of the U.S. Code.   The census of governments and its associated recurrent surveys cover statistics on government finance and public employment.

As with all census and other types of statistical surveys, one of the first tasks is to define the scope of coverage. For the census of governments and its related surveys, this task involves defining the government sector of the nation's economy.

This part contains two chapters that provide an overview of the classification system used by the Census Bureau for its statistical programs on government finance and employment. Chapter 1 covers the government sector as defined by the Census Bureau. Chapter 2 covers the basic classification system used, including the four sectors of government and the types of statistics. Users also can refer to the 2002 Census of Governments, Volume 1, *Government Organization*, for additional detail about how the Census Bureau defines the government sector.

Chapter 1. The Basic Unit of Reporting – The Government Entity

Introduction

Individual governments are the basic reporting units for Census Bureau statistics on government finance and employment. The government entity includes the parent government and all agencies, institutions, and authorities connected to it. To identify the latter, the Census Bureau uses an established set of criteria to determine which of the government organizations with governmental or quasi-governmental character should be included in the government sector. For statistical surveys, the central or primary government is often referred to as the parent government. The associated agencies, institutions, and authorities are generally referred to as dependent agencies of the parent government.

1.1 Types of Government Entities

The Census Bureau identified 87,525 governments during the 2002 Census of Governments. In addition to the Federal Government and the 50 state governments, the Census Bureau recognizes five basic types of local governments.

> *County Governments* (3,034). Organized county governments are found throughout the nation, except in Connecticut, Rhode Island, the District of Columbia, and limited portions of other states where county areas lack a distinct county government. They are created to provide general government activities in specified geographic areas. In Census Bureau statistics, counties include those entities called boroughs in Alaska and parishes in Louisiana.
>
> *Municipal Governments* (19,429). Municipalities are sub-county general purpose governments established to provide general services for a specific population concentration in a defined area. Municipal governments include cities, boroughs (except in Alaska), villages, and towns (except in the six New England states, Minnesota, New York, and Wisconsin). Consolidated city-county governments are treated as municipal governments for Census Bureau statistics.
>
> *Township Governments* (16,504). Townships are sub-county general purpose governments established to provide general services for areas without regard to population concentrations. They include towns in the six New England states, Minnesota, New York, and Wisconsin, and townships in eleven other states.
>
> *Special District Governments* (35,052). These are established to provide only one or a limited number of designated services (functions) and have sufficient administrative and fiscal autonomy to qualify as independent governments.
>
> *School District Governments* (13,506). These are created to provide public elementary, secondary and/or higher education services and have sufficient administrative and fiscal autonomy to qualify as independent governments. They exclude school systems that are "dependent" on a county, municipal, township, or state government.

County, municipal, and township governments are referred to as "general purpose" local governments in Census Bureau statistics on governments. Special district and school district governments are referred to as special purpose governments.

1.2 Definition of a Government Entity

A government is defined as follows, for Census Bureau reporting:

> *A government is an organized entity which, in addition to having governmental character, has sufficient discretion in the management of its own affairs to distinguish it as separate from the administrative structure of any other governmental unit.*

An entity must possess all three of the following critical attributes to be regarded as a government:

Existence as an Organized Entity:

Evidence of this attribute is provided by the presence of some form of organization and the possession of some corporate powers, such as perpetual succession, the right to sue and be sued, have a name, make contracts, acquire and dispose of property, and the like.

Designation of a class of governments in law as “municipal corporations,” “public corporations,” “bodies corporate and public,” and the like indicates that such units are organized entities. On the other hand, some entities not stated specifically by law as corporations do have sufficient powers to be recognized as governments.

The right to exist is not sufficient. Where a former government has ceased to operate – i.e., receives no revenue, conducts no activities, and has no current officers – it is not recognized as an active government.

Governmental Character:

This characteristic is indicated where officers of the entity are popularly elected or are appointed by public officials. A high degree of responsibility to the public, demonstrated by requirements for public reporting or for accessibility of records to public inspection, is also taken as critical evidence of governmental character.

Governmental character is attributed to any entities having power to levy property taxes, power to issue debt paying interest exempt from Federal taxation, or responsibility for performing a function commonly regarded as governmental in nature. A lack of these attributes or of evidence about them, however, does not preclude a class of units from being recognized as having governmental character, if it meets the indicated requirements as to officers or public accountability. Thus, some special district governments with no taxing powers provide electric power or other public utility services that are also widely rendered privately; they may be recognized as local governments because of provisions as to their administration and public accountability.

Substantial Autonomy:

This requirement is met where, subject to statutory limitations and any supervision of local governments by the state, an entity has considerable fiscal and administrative independence. Fiscal independence generally derives from power of the entity to determine its budget without review and detailed modification by other local officials or governments, to determine taxes to be levied for its support, to fix and collect charges for its services, or to issue debt without review by another local government.

Administrative independence is related closely to the basis for selecting the governing body of the entity. Accordingly, a public agency is recognized as an independent government if it has independent fiscal powers and in addition (1) has a popularly elected governing body; (2) has a governing body representing two or more state or local governments; or (3) even in the event its governing body is appointed, performs functions that are essentially different from those of, and are not subject to specification by, its creating government.

1.2.1 Other Factors Used in Defining Governments

Applying the above criteria usually involves little difficulty in most instances. However, the determination of autonomy, as well as other factors, sometimes leaves the classification of entities subject to considerable judgment. In such cases, the Census Bureau has taken account of (a) local attitudes as to whether the type of unit involved is independent or not, and (b) the effect of the decision upon collection and presentation of statistics on government finances and employment.

1.2.2 Noncritical Characteristics Used in Defining Governments

In addition to the essential characteristics above, there are other common attributes that are not essential for the identification of governments. Among them are geographic area, population, taxing power, and internal uniformity of taxation and services.

Most, but not all, governments serve and operate primarily within a specific geographic area for which a population can be determined. Some entities having all essential characteristics of local governments, however, do not possess this attribute, but at best can be associated only with an area unrelated to specific population concentration (e.g., a special district for toll road and bridge facilities).

Although most governments have the authority to levy taxes, this power is not an essential attribute. Even those governments that have property taxing powers and serve a precisely determined area do not always provide a single level of taxation or services throughout the area concerned. Differential taxation often occurs legally where annexation or other boundary changes place a burden of debt service on part but not all the territory. Moreover, subordinate "taxing districts" are sometimes provided for, with regard to particular types of improvements or government services. The existence of Business Improvement Districts is an example of how this taxation could take place.

1.3 The Parent Government Concept

One by-product of this broad definition of a government entity is the concept of the parent government. Governments operate using a wide variety of organizational units to fulfill their duties. The core of the government to which these organizations are attached (as defined above) is often referred to as the "parent" government. A dependent agency or school system often is described in terms of the relationship to its parent government.

1.4 Dependent Agencies

Within the definition of "government," separate existence is not attributed to entities lacking either fiscal or administrative independence. Some local government agencies having either considerable fiscal autonomy or administrative autonomy are classified as dependent agencies of another government, rather than as governments, because one or more of the following characteristics is present:

1. Control of the agency by a board composed wholly or mainly of parent government officials.

2. Control by the agency over facilities that supplement, serve, or take the place of facilities ordinarily provided by the creating government.

3. Provision that agency properties and responsibilities revert to the creating government after agency debt has been repaid.

4. Requirements for approval of agency plans by the creating government.

5. Legislative or executive specification by the parent government as to the location and type of facilities the agency is to construct and maintain.

6. Dependence of an agency for all or a substantial part of its revenues on appropriations or allocations made at the discretion of another state or local government.

7. Provision for the review and the detailed modification of agency budgets by another local government. County review of agency budgets in connection with statutory limitations on tax rates, however, is not, by itself, sufficient to establish lack of fiscal autonomy.

The Census Bureau's definition of a government entity includes the parent government and also agencies that are "dependent" on the government, using the criteria above.

The determination of an agency's dependence or independence for Census Bureau purposes is not made on an ad hoc basis. The Census Bureau's Governments Division identifies and classifies the agencies that are dependent on parent governments. The findings are reported in a "Database of State Dependent Agencies" (formerly the "Checklists of Dependent Agencies for Government Divisions Surveys"). Dependent agencies also are identified in the Governments Integrated Directory, especially for local governments (see Section 1.7). Census Bureau statisticians use both of these references to assign agencies to their proper parent government.

1.5 Joint Activities of Governments

Governments sometimes cooperate to carry out specific projects or activities – e.g., city-county health departments or hospitals, joint bridge authorities, joint interstate agencies, consortia, and councils of government.

The participating units may carry out this joint activity in various ways, each of which affects the reporting of finance and employment statistics for the participating governments:

1. They may establish an independent special district to carry out the activity, in which case the related finances and employment are assigned to that government unit. Their financial transactions with the special district are treated as intergovernmental revenue and expenditure.

2. One of the governments might be solely responsible for administering the activity but the other governments share its financial support. In such situations, all its activities are assigned to the administering government with the other governments' shares being reported as intergovernmental transactions.

3. The participating governments might create a separate organizational body that is neither

independent nor dependent on any one member but is jointly administered by all of them. These types of joint activities provide special problems in a classification system whose focus is the individual government entity. Such joint activities, therefore, are classified on the basis of the circumstances involved in each case. The Census Bureau also considers the magnitude of financial or employment activity involved in deciding on to classify these activities.

See Section 3.14 for an explanation of how these are classified in statistics on government finances, and Section 11.4 for an explanation of how these are classified for statistics on public employment.

1.6 Government Organization – The Government Sector

The government sector is comprised of all the individual units of government and their respective dependent agencies and activities. For Census Bureau statistical surveys, it consists of three levels – the Federal Government, state governments, and all local governments. The local level in turn consists of the totality of each type of local government, as noted above.

1.6.1 Statistical Aggregation and Tabulation

Census Bureau statistical surveys covering government finance and employment build upon the individual unit of government. All applicable statistics for the individual unit are aggregated, including the statistics for the parent government and all dependent agencies. Individual government statistics in turn can be tabulated to develop statistical measures in a number of ways. Among the tabulations frequently used in Census Bureau statistical reports are the following:

- National totals – consisting of all three levels of government (such as the national total of full-time public employees).
- State area totals – consisting of the state government plus all local governments within the state.
- County area totals – consisting of the county government plus all local governments within the county.
- Level of government totals (such as total state government tax collections).
- Type of government totals, which can be tabulated at the national level or within a state area (such as total township tax collections within a state).

1.7 The Governments Integrated Directory (GID)

The Census Bureau maintains a database of all governments in the United States, along with their dependent agencies. This database is the Governments Integrated Directory, or GID. The GID is updated continuously, although a formal and comprehensive update occurs every five years during the Census of Governments. Information about the GID and access to it can be found on the Census Bureau's Internet website.

The GID contains a significant amount of identifying information about each government unit or dependent agency. In addition to names and address, such information might include the type of government, the services provided by the government, the school levels (such as K –12) supported by the government or dependent school system, school enrollment, demographic information such as population, and geographic information such as related geographic codes. The most important piece of information in

the GID is the government unit code. This code, along with three other types of codes useful to the government finance and employment statistics program, are described in Section 1.8.

1.8 Government Organization Reference Codes

The Census Bureau uses a variety of codes for purposes of identifying governments and classifying their activity. Four are described here, as they are important for purposes of classifying and processing statistics on finance and employment. Users interested in more detailed information should refer to Volume 1, Number 1, of the Census of Governments (*Government Organization*), or to the Census Bureaus website.

1.8.1 Government Unit Codes

The government unit code is the basic identification system used by the Census Bureau to keep track of all governments and their dependent agencies. The code is a 14 digit, numeric identifier. The format is:

SS T CCC UUU SUP SB

where:

SS = state
T = type of government
CCC = county area within the state
UUU = unit code within the county area
SUP = supplemental unit code, for dependent agencies of the parent unit
SB = subsidiary code for dependent agencies that require separate data collection / canvassing

The following parameters and rules apply to the code structure:

- SS is a numeric sequence assigned to the states alphabetically – Alabama is 01, Alaska 02, and so forth, until Wyoming at 51. Note the District of Columbia is assigned code 09 and the U.S. Federal Government is assigned code 00.

- T represents type of government, as defined by the Census Bureau. Government type codes are
  0 = state governments
  1 = county governments
  2 = municipal governments
  3 = township governments
  4 = special district governments
  5 = school district governments
  6 = Federal Government

- CCC, the county area codes, are assigned alphabetically within each state area, although there are some exceptions where county consolidations have taken place or new counties have been created.

- The unit code UUU identifies a specific government within the state, county, and type of government sequence. They, too, are generally alphabetical within the county area and type of government combination, but exceptions exist (especially for special districts). The unit code

for a county government is usually the same as its county area code.

- The supplemental code SUP identifies different dependent agencies of the same parent state or local government. Every supplement must have a parent government. The first nine digits of a supplement and parent code always are the same.

- SB is the subsidiary code is used for component units of dependent agencies that require a separate data collection. A good example is found in state dependent university systems, where separate campuses exist for a dependent university, each campus having its own subsidiary code.

For state governments, the type, county area, and unit codes are 0. Thus the code for the New York state government is: 33 0 000 000 000 00.

For dependent agencies, the full code (all 14 digits) is used. The following example shows the code for New York City and some of its dependent agencies.

33 2 031 001 000 00 = New York City
33 2 031 001 301 00 = New York City public school system (dependent on the city government)
33 2 031 001 302 00 = Fashion Institute (dependent post-secondary education agency)
33 2 031 001 303 00 = CUNY, City University of New York (dependent on the city government)
33 2 031 001 303 01 = Manhattan Community College (one campus of CUNY)

The structure of the government unit codes makes them useful for data processing purposes. Individual unit financial aggregates would consist of the sum of all applicable variables for any unit having the common nine digit codes of SS T CCC UUU, for example, regardless of the SUP and SB codes.

1.8.1.1 Special Topics: Government Unit Code Historical Assignments

The Census Bureau used a general historical pattern to assign the unit code (UUU) sequence of government unit codes for each census of governments. The pattern applied to new governments (or newly identified governments) only, and not to existing governments. The pattern was "general" because, in some instances, the number of local governments exceeded the available codes, and the pattern could not be followed strictly. Nevertheless, the pattern is useful for analysts (see table on the following page).

Chart 1.A Government Unit Code Assignments by Year	
Unit Code Range:	Applicable Census of Governments Year:
000 - 099	1957 Census of Governments
100 - 199	2007 Census of Governments *
200 - 299	1992 and 1997 Censuses of Governments
300 - 399	2002 Census of Governments
400 - 499	1987 Census of Governments
500 - 599	1962 Census of Governments
600 - 699	1982 Census of Governments
700 -799	1967 Census of Governments
800 - 899	1972 Census of Governments
900 - 999	1977 Census of Governments

*This range also was used in the 1957 Census of Governments for local governments in three county areas – Allegheny County, Pennsylvania, Cook County, Illinois, and Harris County, Texas.

1.8.2 Government Organization Function Codes

Government organization function codes are used primarily to classify the activities of special district government units. They are important in the Government Organization Phase of the Census of Governments, wherein the Census Bureau develops information on the structure and organization of government units, and the government sector generally.

Government organization function codes are closely aligned with the function codes used to classify government expenditure and public employment. However, there are some differences between the three sets of codes. The most notable difference is that there are fewer government organization function codes than there are finance expenditure functions or employment functions. The reason there are fewer government organization function codes is that many types of special districts exist in large numbers, but represent extremely small aggregates of financial or employment activity. Cemetery districts are an example. Thousands of these exist, but their financial and employment levels are so small as to preclude separate expenditure and employment measures.

Another difference is that some government organization function codes represent multiple activities, rather than single purpose activities. Table 1.1 shows the government function codes used in the Organization Phase of the Census of Governments. Chapter 5 contains the detailed codes used to classify government expenditure statistics, and Chapter 12 contains the detailed function codes used to classify public employment statistics.

Special district governments are assigned a government organization function code. Their expenditure activities are then classified in accordance with that code. Table 1.2 contains a crosswalk used for this conversion process.

1.8.3 Government Organization Activity Codes

Government activity codes are similar in purpose to government function codes. However, instead of applying to special district governments, they are used to classify the purpose for which dependent agencies exist (the services such agencies provide). Like government function codes, they are two digit numeric codes. These are shown in Table 1.1, along with the government function codes.

1.8.4 Government Organization School Level Codes

School level codes are contained in the GID for school district governments and dependent school systems. They indicate the level of educational instruction provided by each school system. College and other postsecondary institutions are included. The codes are noted on the next page.

Chart 1.B School Level Codes

Code	Description
01	Elementary only
02	Secondary only
03	Elementary and Secondary
04	Postsecondary
05	Special / Vocational education
06	Nonoperating school system
07	Education Service Agency only

Elementary and secondary = Pre-kindergarten through grade 12.
Postsecondary = 2 year, 4 year and post graduate college/universities, plus other training beyond the high school level.
Nonoperating = A school system that exists for administrative purposes only and does not operate its own schools
Education service agency = A dependent agency that provides a specialized service for one or more school systems, such as a special education cooperative.

1.8.5 ID Codes for Files of Aggregated Individual Government Records

As noted in Section 1.6.1, individual government records are the building blocks for producing higher-level statistics on government finances and employment. Records in the files produced by this process require their own, unique identification codes. The ID coding schemes for the two most common types of aggregated data files are presented below:

State-by-type-of-government aggregates:

In these files, individual state and local government records are summed by (a) state and (b) within state by level of government (state versus local) and (c) by type of local government. Aggregated records representing the United States as a whole are also produced. The ID code assigned to these records is a 3 digit, numeric identifier whose format is:

SS T

where:

SS = state code
T = type of government

The state code is the same as the one assigned to the individual unit code described in Section 1.8.1. Records representing the Federal Government as well as the United States total are assigned a state code of “00”.

The type code, however, consists of one of the following:

- 0 = Federal Government
- 1 = total of state and local governments
  - 2 = state government(s)
  - 3 = total of local governments
    - 5 = county governments
    - 6 = municipal governments
    - 7 = town or township governments
    - 8 = special district governments
    - 9 = school district governments

Note that data for dependent school systems are included with those of their parent governments.

County area aggregates:

In these files, individual local government records are summed by (a) state and (b) within state by county area. The ID code assigned to these records is a 5 digit, numeric identifier whose format is:

SS CCC

where:

SS = state
CCC = county area within the state

Both the state code and county area code are the same as the ones assigned to the individual unit code described in Section 1.8.1.

The following chart summarizes the differences in the type code schemes between files of individual government records and those aggregated by state and type of government (see chart on following page).

Chart 1.C Government Type of Government Codes		
Level or type of government	Type code for individual government records	Type code for state-by-type-of-government records
Federal Government	6	0
State and local government total	-	1
State government(s)	0	2
Local government total	-	3
County governments	1	5
Municipal governments	2	6
Town or township governments	3	7
Special district governments	4	8
School district governments	5	9

Key:
"-" Indicates that no such code applies to the individual government records.

1.9 Tables

Table 1.1 contains the detailed government organization function codes and the government organization activity codes currently in use by the Census Bureau. Table 1.2 contains the full set of government organization function codes and their associated government expenditure functions.

Table 1.1 Government Organization Function and Activity Codes

Function Code	Activity Code	Title	Function Code	Activity Code	Title
01	01	Air Transportation	60	60	Parking Facilities
02	...	Cemeteries	61	61	Parks and Recreation
03	03	Miscellaneous Commercial Activities	62	62	Police Protection
04	04	Correctional Institutions	63	...	Flood Control
05	05	Other Corrections	64	...	Irrigation
09	...	Education (School Building Authorities)	77	77	Public Welfare Institutions
...	12	Elementary and Secondary Education	79	79	Public Welfare
...	18	Other Higher Education	80	80	Sewerage
...	21	Other Education	81	81	Solid Waste Management
...	22	Social Insurance Administration	86	...	Reclamation
...	23	Financial Administration	87	87	Sea and Inland Port Facilities
24	24	Local Fire Protection	88	...	Soil and Water Conservation
...	25	Judicial and Legal	89	...	Other Single Function Districts
...	26	Legislative	...	89	Other and Unallocable
...	29	Central Staff Services	...	90	Liquor Stores
...	31	General Public Buildings	91	91	Water Supply Utility
32	32	Health	92	92	Electric Power Utility
40	36	Hospitals	93	93	Gas Supply Utility
41	...	Industrial Development	94	94	Public Mass Transit Utility
42	...	Mortgage Credit	96	24/91	Fire Protection and Water Supply
44	44	Regular Highways	97	59/91	Natural Resources and Water Supply
45	45	Toll Highways	98	80/91	Sewerage and Water Supply
50	50	Housing and Community Development	99	Various	Other Multi-function Districts
51	...	Drainage	...	X1	Public Employee Retirement Systems
52	52	Libraries	...	Y1	Workers' Compensation Systems
59	59	Other Natural Resources			

Key:
... Indicates that the code does not apply for either the function or activity category.

**Table 1.2 Government Organization Function Codes and Their Associated Finance Expenditure Codes –** page 1 of 2		
**Organization Function Code**	**Name**	**Expenditure Function Code**
Single-Function Districts:		
01	Air Transportation (Airports)	01
02*	Cemeteries	03
03	Miscellaneous Commercial Activities	03
04	Correctional Institutions	04
05	Other Corrections.	05
09*	Education (School Building Authorities)	12
24	Local Fire Protection	24
32	Health	32
40*	Hospitals	36
41*	Industrial Development	89
42*	Mortgage Credit	89
44	Regular Highways	44
45	Toll Highways	45
50	Housing and Community Development	50
51*	Drainage	59
52	Libraries	52
59	Other Natural Resources	59
60	Parking Facilities	60
61	Parks and Recreation	61
62	Police Protection	62
63*	Flood Control	59
64*	Irrigation	59
77	Public Welfare Institutions	77
79	Other Public Welfare	79
80	Sewerage	80
81	Solid Waste Management	81
86*	Reclamation	59
87	Sea and Inland Port Facilities	87
88*	Soil and Water Conservation	59
89	Other Single-Function Districts	89
91	Water Supply Utility	91
92	Electric Power Utility	92
93	Gas Supply Utility	93
94	Mass Transit System Utility	94
* Code is unique to the Organization Survey. Government Finance data are subsumed into regular expenditure function code as shown in table above.		

**Table 1.2 Government Organization Function Codes and Their Associated Finance Expenditure Codes** – page 2 of 2		
**Organization Function Code**	**Name**	**Expenditure Function Code**
Multiple-Function Districts:		
96*	Fire Protection and Water Supply	24/91
97*	Natural Resources and Water Supply	59/91
98*	Sewerage and Water Supply	80/91
99*	Other Multi-Function Districts	Various
* Code is unique to the Organization Survey. Government Finance data are subsumed into regular expenditure function code as shown in table above.		

Chapter 2. Classification System Overview – Government Sectors and Types of Statistics

Introduction

Although the Census Bureau began regular collection of government finance data in the early 1900's, the current classification system is based on a redesign that was implemented with data for 1951. Like its predecessor, this classification system makes use of the concept of government sectors and employs four types of statistics. This chapter explains these classification system issues. It also contains a summary description of data collection methodologies used by the Census Bureau for its surveys on government finance and public employment.

2.1 The Four Sectors of Government Activity

The Census Bureau categorizes all government activities into one of four mutually exclusive sectors: general government, utilities, liquor stores, and social insurance trust systems. Each sector is described below.

2.1.1 General Government Sector

The general government sector includes all activities not defined in the utilities, liquor stores, or social insurance trust sectors. For Census Bureau purposes, government operated lotteries are included in this sector.

2.1.2 Utilities Sector

This sector is comprised of four types of governmental activities:

Water supply	Gas supply
Electric power	Public mass transit

Utility systems must be either owned and operated by a government, or owned and operated under contract by a private firm where the government maintains day-to-day financial oversight.

Except for these four types of utilities and the liquor stores sector below, all commercial-type activities of a government (airports, toll roads and bridges, housing projects, parking facilities, port facilities, lotteries, Federal Government corporations, and the like) are classified in the general government sector.

2.1.3 Liquor Stores Sector

This sector covers only liquor store systems owned and operated by many state governments, and local governments in some states. The Census Bureau definition limits this sector to government operated retail or wholesale liquor monopolies. Any associated government activities, such as licensing and enforcement of liquor laws and collection of liquor taxes, are classified in the general government category. As of 2005, this sector applies to seventeen state governments, and to local governments in seven states.

2.1.4 Social Insurance Trust Sector

The social insurance trust sector represents government operated social insurance trust systems. Explained in detail in Chapters 8 and 9, this sector consists of social insurance operations for which a government maintains accounting trust funds separate from other funds or financial activities of the government. The system must also provide a social purpose.

To be categorized as an insurance trust for purposes of Census Bureau statistics, a system must meet all five characteristics of a social insurance trust described in Sections 8.1 or 9.1.

The social insurance trust sector is comprised of four sub-sectors, based on the type of system:

1. Public employee retirement systems, embracing both contributory and noncontributory systems administered by a government for public employees (including employees of other governments). This sub-sector is limited to defined benefit plans (see Chapter 8).

2. The Federal-state cooperative unemployment compensation system, which is based on Federal law but administered by state governments (and the city of Washington, DC) under state law. For the Census Bureau's government statistics program, the latter activities are included in the financial statistics of each state government and Washington, DC.

3. State government workers' compensation systems, where they exist. Not all states administer a workers compensations system.

4. Other state government social insurance trusts, such as those for uninsured motorists or disability insurance. These types of social insurance systems are not defined as applicable to local government finances.

Note that the insurance trust category applies only to the government actually administering the system. A government's participation in an insurance trust administered either privately or by another government is not treated as an insurance trust activity of the participating government. Generally, it would be treated as a current operations expenditure for the paying activity in the general government, utilities, or liquor stores sector.

No employment statistics are classified within the insurance trust sector. Employment related to administering insurance trust systems is classified in the general government sector.

2.1.5 Summary Chart: The Four Sectors of Government Activity

Not all of these four sectors apply to every type of finance or employment statistic. Chart 2.A on the following page indicates whether statistics apply (Yes) or do not apply (No) to each sector.

In all cases where data do not apply to the particular sector, they are included instead in the "general" sector measures. Thus if a state government issues debt to construct a building to house its liquor distribution warehouse, such debt is included in the general government sector. The same reasoning holds true for counts of employees, as noted above. For example, employees of a government administered retirement system are included in the general sector counts of employees (at *Financial Administration*, code *23).

Chart 2.A The Four Sectors of Government Activity				
**Type of Data**	**General**	**Utilities**	**Liquor Stores**	**Social Insurance Trust**
FINANCE:				
Revenue	Yes	Yes	Yes	Yes
Expenditure	Yes	Yes	Yes	Yes
Debt	Yes	No[1]	No[1]	No[1]
Cash and Securities	Yes	No[1]	No[1]	Yes
EMPLOYMENT	Yes	Yes	Yes	No

[1]Any such amounts are reported under the general government sector

There are two general reasons for excluding selected finance or employment statistics from a particular sector. The first is definitional – the conceptual make-up of the sectors. The social insurance trust sector was conceived to measure the financial activity of these special government services. Historically, many employees involved in administering these systems also were involved in administering other government financial activities. This concept has prevailed over the years, even where such systems have grown, or become highly specialized with separate administrative units. Employees of social insurance trust systems are thus defined as serving in the "financial administration" functional area within the general government sector.

A second reason generally has to do with the magnitudes of activity. Where magnitudes of a particular type of government activity are minimal, the resources used to keep track of that activity separately are considered excessive for this statistical program. Traditionally, there are very few instances of debt activity for liquor stores, for example, so debt applicability to that sector has been excluded as impractical.

Effective in 2005, debt statistics are no longer collected separately in the utilities sector. Instead, they are classified along with all other government debt and reported in the general sector statistics. Separate data are available in historical reports, tables and electronic files for years prior to 2005. This revision is explained more fully in Appendix 1.

2.2 The Four Types of Census Bureau Statistics

The Census Bureau uses four types (categories) of statistics in its programs on government finance and public employment. These are regular statistics, exhibit statistics, derived statistics, and descriptive statistics. Each type contributes to the overall statistical program, but to different degrees. Each is explained in the sections that follow.

Note that all four types are used in the statistical programs on government finance (including the programs on public employee retirement system finances described in Chapter 8). The statistical program on public employment comprises primarily regular type statistics, however.

2.2.1 Regular Statistics

The regular statistics are the foundation of the Census Bureau's program to measure government finances and empoyment. The critical perspective in the regular statistics program is to measure the financial activity for each government as a whole. For regular statistics, all components of a single government entity, including the parent government and all its dependent agencies and activities, can be aggregated to obtain total financial activities or employment for an individual government unit.

For finance data, a key characteristic of the regular statistics is that they generally exclude a government's internal transactions. For example, the financial transactions between a parent government and any of its dependent agencies or activities are not counted in regular Census Bureau statistics on government finance. Likewise, transfers or contributions between sectors of governments are excluded from Census Bureau regular statistics.

2.2.2 Exhibit Statistics

Exhibit statistics expand the statistical coverage for selected government sectors or for selected types of activities. They do so in two ways. First, they provide additional detail to the regular statistics, showing financial and employment activities outside the scope of regular statistics. Second, some exhibit statistics are designed to account for internal financial transactions excluded from regular statistics. For example, exhibit statistics allow transactions between a social insurance trust system and its parent government to be reported, while regular statistics exclude them, by definition.

Examples of current exhibit statistics include the following:

- Total salaries and wages for the fiscal year (all functions and sectors combined).
- Transactions of state liquor control systems, including financial relationships with the parent government.
- Selected operations of state lottery systems.
- Data about receipts from, or expenditures of, proceeds from the Tobacco Lawsuit Settlement between the state governments and the tobacco industry.
- More complete detail on the operation of social insurance trusts (especially public employee retirement systems), including transactions with other government sectors and the parent government.
- Special codes applicable only to public school system data.
- Full and part-time pay codes, used in the employment survey to derive full-time equivalent employment.

The additional detail provided by exhibit statistics can be best examined in the social insurance trust sector. Regular statistics can capture only certain aspects of these complex financial transactions. In the case of public employee retirement systems, the additional detail provided by exhibit statistics consists primarily of additional data on revenue sources and cash and security holdings. This added detail is important because at the state and local government level, such holdings of public employee

retirement systems totaled $2.2 trillion in the last Census of Governments (2002), representing sixty percent of total government cash and security holdings.

The Census Bureau does not collect this same level of detail for all sectors of government for a number of reasons, including the fact that other areas do not have the extensive cash and security holdings found in public employee retirement systems.

Exhibit statistics can also provide additional detail to meet the needs of special users of Census Bureau data. For public employee retirement system statistics, the major user is the Federal Reserve Board, which uses these additional exhibit statistics in its own statistical programs (such as the Flow of Funds Accounts).

2.2.3 Derived Statistics

In addition to those collected as part of the basic government finance and public employment surveys, the Census Bureau produces other statistics that are derived from the regular and exhibit statistics. Unlike the latter, derived statistics usually do not have a standard variable code assigned to them.

For financial data, derived statistics are limited largely to two areas – four involving indebtedness (detailed in Section 6.3) and four involving government-operated liquor stores (detailed in Section 10.15).

For employment data, the main derived statistics are: the conversion of reported payrolls to monthly figures, full-time equivalent employment, and average monthly pay for full-time employees.

Derived statistics should not be confused with other calculated statistics. Calculated statistics can be found among the regular and exhibit statistical type categories. In addition, all derived statistics are "calculated" from other statistical measures. The term "calculated" refers to the methodology – how the statistics are developed, rather than to a type of statistic category.

2.2.4 Descriptive Statistics

Descriptive statistics are a fourth type used in the Census Bureau's government statistics program, but actually serve a dual purpose. They are mostly used by the Census Bureau to collect information on the characteristics of a particular type of government activity. In this way, they are used to explain the structure and organization of government units. Currently, there are twenty descriptive statistic codes in the Census Bureau's classification system. All twenty apply to public employee retirement systems and are described in detail in Section 8.6. Descriptive statistics have a standard three character code, as do regular and exhibit statistics.

For public employee retirement systems, descriptive statistics represent information on system characteristics. These descriptive statistics also are developed for specific user needs to describe or characterize the important role of public employee retirement systems. Descriptive statistics include data on membership, the type of system, numbers of annuitants, and so forth. Some of these descriptive statistics are collected only during a census of governments.

2.3 Special Topics: How Census Bureau Statistics on Governments are Developed

The Census Bureau uses four basic methods to develop it statistics on government finances and employment. These are described briefly here, for reference purposes. Regardless of the methodology,

the categories contained in this Classification Manual serve as the basis for developing the statistics. More detail about methodology is available from the Census Bureau's website or by contacting the Census Bureau directly.

2.3.1 Mail Canvass

This methodology employs a survey questionnaire that is transmitted to respondents, with instructions and guidelines. The respondent completes the questionnaire and returns it directly to the Census Bureau for editing and processing. The term "respondent" generally refers to government units, although dependent agencies of governments are canvassed separately to collect missing data.

For state government employment statistics collected using this method the Census Bureau canvasses individual state agencies, rather than sending a single questionnaire to a central payroll agency.

Much data collection activity (requests and responses) now involves electronic reporting via the Internet, rather than actual paper questionnaires transmitted via the regular mail.

2.3.2 Compilation

For state governments and many large county and city governments, the Census Bureau compiles government finance statistics directly from government reports and records. In many cases, this is performed electronically, using data files provided by the government.

The reasons have to do with the complexity of the accounting records of the large governments, the corresponding complexity of the Census Bureau government finance classification system, and the potential reporting burden to the government if it were to complete a questionnaire.

For surveys covering public employment, compilation is used less frequently.

Compilation is also employed under other circumstances. Governments that are initially sent a questionnaire sometimes do not respond, or return the questionnaire along with an official financial report. In these cases, Census Bureau analysts can compile the statistics they need from the official records provided.

2.3.3 Central Collection

The Census Bureau also engages in cooperative arrangements to collect data. Some of these efforts involve joint data collection with a state government agency, while others involve states sharing data from their own collection systems. Regardless, these cooperative arrangements yield very high quality data while minimizing reporting burden on governments.

In a typical central collection arrangement, the Census Bureau works with a state agency responsible for monitoring either local government finances or for handling central personnel records. The Census Bureau and the state work closely together in order to minimize overall reporting burden by designing the joint data collection to meet the needs of both the Census Bureau and the state. Classification and definitional differences are taken into account and managed.

Central collection is used for both the government finance and the public employments statistics programs. Notably, the Census Bureau collects nearly all of the finance statistics covering dependent and independent elementary-secondary school systems via central collection arrangements with state

government education agencies. For public employment, most state government statistics are collected from central payroll agencies. Employment data for some local elementary and secondary school districts are collected in coordination with state education agencies.

2.3.4 Calculated Statistics

In addition to the above statistics, the Census Bureau defines other variables that calculated from other statistical codes. These calculations are performed by the Census Bureau rather than by the respondent.

This methodology is used for several reasons. In some cases, the calculated statistic would add unnecessarily to the reporting burden of the respondent. In other cases, the use of a calculated statistical code ensures consistency in measurement, where complex methodology is involved. Such is the case with the public employment statistics for converting reported payrolls to a monthly amount (explained in Chapter 11).

Note that calculated statistics are found among three of the four basic types of statistics in the Census Bureau's program. Selected regular and exhibit codes are calculated, and all derived statistical codes are calculated, by definition. These calculated statistical codes are clearly noted throughout this *Classification Manual.*

2.3.5 Imputation

Imputation refers to the process of estimating what would have been reported by a government unit that did not respond to a request for information. For government finance and employment, imputation is less of a problem than for other types of surveys (such as demographic surveys), because most state and local financial and employment activity are a matter of public record. Consequently, the Census Bureau can resort to the compilation methodology described above to obtain data from publicly available records, when a government cannot respond to information requests.

Even so, some imputation to account for unit nonresponse is used in Census Bureau surveys on government finance and public employment. It generally represents a relatively small share of the total financial or employment levels being measured, however, since large governments dominate the activity levels within the government sector overall.

The topic of imputation is a complex one that involves many statistical and methodological issues. More information about imputation methodologies used can be obtained from the Census Bureau.

PART 2. GOVERNMENT FINANCE STATISTICS

This part of the *Government Finance and Employment Classification Manual* covers the Census Bureau's statistical program on government finances. This part is organized into eight chapters. Chapter 3 provides an overview of the classification system for government finance statistics, explaining basic concepts and coverage. Chapters 4 through 7 contain the definitions and explanations about categories used to classify the four types of government finance statistics: revenue, expenditure, indebtedness, and cash and security holdings. Chapters 8 and 9 contain more detailed definitions and explanations of the financial categories that apply to social insurance trust systems. Chapter 10 is dedicated to the finances of government operated liquor stores and lottery operations.

Chapter 3. The Foundation for Census Bureau Financial Statistics On Governments

Introduction

Governments maintain their financial records using similar practices and procedures that help shape the Census Bureau’s classification system for its government finance statistics program. This chapter provides a general overview of government finances, the accounting practices of governments, and how the Census Bureau's finance statistics relate to the individual government accounting systems from which they originate.

Sections 3.1 through 3.5 explain a number of coverage and measurement issues, with a focus on the parent government accounting and record-keeping practices and how they are converted for use in the Census Bureau’s statistical program.

Sections 3.6 through 3.14 contain explanations of various common types of government financial transactions, as defined for purposes of Census Bureau statistics. Some of these have different meanings when used in other accounting contexts or scenarios. Nevertheless, proper collection and use of Census Bureau finance statistics requires an understanding of these major concepts.

3.1 Primary Basis of Government Finance Statistics

The primary basis of the government finance statistics reported by the Census Bureau is to measure all the external transactions of a government, exclusive of agency, private trust, and investment transactions. The government unit is treated as an economic entity and the Census Bureau classification system is designed to report on all revenue from external sources, all expenditure flowing externally, and all debt owed to outside interests. Cash and security holdings of the government also are included.

The income concept of “revenue,” the outgo concept of “expenditure,” and the concept of government indebtedness pertain to nearly all accounting funds and accounts, as well as to all boards, commissions, and other agencies of a government (see Chapter 2). The Census Bureau statistics are designed to be net of any duplicating amounts resulting from transactions between funds or agencies of the same government.

3.2 Fiscal Years as Statistical Reporting Periods

Governments keep their financial accounts on a fiscal year basis, almost always a 12 month period. For this Census Bureau program, statistics are collected so that they cover each government’s own fiscal year period, rather than a standard calendar-based reporting period that cuts across government fiscal years. This methodology is used because it links directly to the manner in which governments maintain their financial records. Any attempt to standardize the time frame for 80,000 plus governments would create an insurmountable data collection challenge and would be cost prohibitive.

As a result, the Census Bureau survey of government finance (including each census of governments) is based on the following definition of a fiscal year:

A survey year includes each individual government fiscal year that ended between July 1 of the previous year and June 30 of the survey year. Hence, survey year 2002 (the last census of governments prior to this edition of the *Classification Manual*), covers individual government fiscal years that ended from

July 1, 2001 through June 30, 2002.[1] The resulting Census Bureau statistics for that survey year are referred to as covering fiscal year 2002.

This current definition has been used since 1964. Prior to that time, a calendar basis was used – i.e., local government fiscal years used were those that fell within a particular calendar year.

For Census Bureau purposes, every government canvassed reports its financial data for the fiscal year that fits this current definition. The methodology for tabulating statistics for multiple governments involves summing the individual government amounts to arrive at totals, just as if all governments were on an identical fiscal year cycle. While this methodology results in an imperfect "time frame" for statistical purposes, it has been used consistently for the entire time series for which government financial statistics exist (whether based on the current definition of fiscal year or any prior definition).

Another aspect of the fiscal year topic is that it applies to both parent governments and their dependent agencies, which sometimes have fiscal years that differ. In most cases, the July 1 through June 30 rule still applies. The finance statistics from the appropriate fiscal years for the parent government and the dependent agency are summed, just as if they both used the same fiscal year, to arrive at totals for the overall government unit.

It is useful to have a sense of how actual fiscal years fall. While Table 3.1 contains a breakdown of fiscal year frequencies, it is noted that the Federal Government fiscal year extends from October 1 through the following September 30. Most state governments have a fiscal year that runs from July 1 to the following June 30, although Alabama (September 30) Michigan (September 30), New York (March 31), and Texas (August 31) are exceptions. Local government fiscal years vary. Users interested in exact counts of government fiscal years can make use of the GID database.

In terms of fiscal year reporting, Census Bureau financial statistics employ two types of measures: flow of funds during the fiscal year and measurement at a specific point in time, generally the last day of the fiscal year. The following rules apply to the four basic types of statistics:

Revenue – measured over the entire fiscal year of the government.
Expenditure – measured over the entire fiscal year of the government.
Debt – measured as of the end date of the fiscal year (for debt outstanding) and over the course of the fiscal year (for debt issued and retired).
Cash and Security Holdings – measured as of the end date of the government fiscal year.

Additional detail about how the fiscal year impacts particular statistics covered in this *Classification Manual* appears within each chapter.

This aspect of the Census Bureau statistical program on government finances is quite different from most statistical censuses and surveys, which usually cover a specific time frame or fixed point in time. The Census Bureau statistics on public employment cover the levels of government employment and payrolls for the month of March, annually, regardless of fiscal year of the government or its dependent agencies.

---

[1] For a variety of methodological, practical, and historical reasons, there are three general exceptions to the fiscal year definition. They involve the Federal Government, three of the state governments noted (AL, MI, and TX), and local school systems in Alabama, Nebraska and Texas (where the fiscal year coincides with the start of the traditional school year). For these governments only, the fiscal year moves beyond June 30 cutoff, so that fiscal year 2002 statistics cover the years that ended on August 31 2002 (TX) and September 30, 2002 (Federal Government, AL, and MI).

3.3 Current Dollars

Government finance statistics published by the Census Bureau are in terms of current dollar amounts – i.e., they have not be adjusted for price and wage changes that occur. The reason for using only current dollar measures has to do with the how the Census Bureau statistics are used. All or portions of the statistics are used by other government agencies, economists, or statisticians to analyze various topics pertaining to the government sector. Such analyses are many and varied, and each might have a different requirement for converting the basic financial statistics to account for historical price level changes. The Census Bureau program thus provides the raw data for these subsequent analytical uses of the data.

3.4 Accounting Basis

The major source of these finance statistics is the governments' own accounting systems, either directly from a government's own records or through intermediate reporting systems (like central state collections described in Section 2.3.3).

3.4.1 Accounting Funds (or Entities)

Governments administer their finances through accounting devices called funds (not to be confused with the use of the term "funds" for monies). Funds are established to support specific activities or to attain certain objectives, the most important of which is accountability of public monies. The focus of the government's own reporting system, therefore, is on the transactions and condition of these funds.

In contrast, the Census Bureau public finance statistics are organized to show a government's finances in their entirety, with emphasis on the general government sector and without distinguishing the various accounting funds. In other words, the fund accounting nature of government reports disappears in Census Bureau statistics.

Table 3.2 at the end of this chapter contains a listing of common fund types found in standard financial reporting statements, along with their treatment in Census Bureau statistics on government finance. (Section 3.4.4 provides information on these accounting fund terms, which are based primarily on Governmental Accounting Standards Board terminology.)

3.4.2 Cash versus Accrual Basis of Accounting

State and local government accounting reports and records may provide data on a cash, accrual, or modified accrual basis. For a particular government, the basis of reporting may differ among funds. Census Bureau statistics generally adopt whatever basis the government itself uses so long as that basis (1) conforms to generally accepted accounting procedures and (2) is applied consistently from year-to-year. Because of the growing standardization of accounting methods by governments (see below), this means that most finance statistics pertaining to governmental fund types are based on the modified accrual basis; proprietary (enterprise) fund types are based on the full accrual basis; and fiduciary fund types are recognized on the basis consistent with the fund's accounting measurement objective.

It should be noted that some governments may still use cash basis accounting because of

constitutional provisions or other state law.[2]

For statistics on Federal Government expenditure, the Census Bureau includes actual outlays rather than obligations or amounts authorized.

3.4.3 Special Topics: Governmental Accounting Standards Board (GASB)

The Census Bureau's government finance statistics have been increasingly influenced by the adoption by governments of generally accepted accounting procedures issued under the aegis of the Governmental Accounting Standards Board (GASB). Until the 1990s, the accounting rules for governments were broad enough that each could develop their own unique form of accounting for their finances. Each financial report, for instance, looked differently and reported varying degrees of detail.

In recent years, more governments have adopted standard accounting procedures for their reporting systems, which has had a profound effect on the Bureau's data collection efforts. On the one hand, finance data are being recorded by governments in a more consistent fashion. On the other hand, the products of this system result in a lower level of detail than in the past, forcing the Census Bureau to rely less on published financial reports and more on internal accounting records of governments. This has both complicated the work involved and resulted in the modification of selected categories of data and the basis by which some categories are measured.

The most profound effects have been to statistics on government indebtedness and cash and security holdings. See Chapters 6 and 7 for a discussion of these two topics.

3.5 Four Sectors of Government from a Finance Viewpoint

As described in Section 2.1, the activities of governments are divided into four sectors for Census Bureau purposes. The explanations below provide additional information about these sectors in terms of how they apply to government finance statistics.

General Government Sector: Within the totals of government revenue and expenditure, internal transfers (e.g., interfund transactions) are "netted out." Therefore, "general revenue" and "general expenditure" represent only revenue from external sources and expenditures to individuals or agencies outside the government, and do not directly reflect any "transfer" or "contributions" to or from the utilities, liquor stores, or insurance trust sectors. See Section 3.9 for more information on internal transactions.

Utilities Sector: In the primary classification of government revenue and expenditure, the term "utility" is used to identify certain types of revenue and expenditure categories. Utility revenue relates only to the revenue from sales of goods or services and by-products to consumers outside the government. Revenue arising from outside other aspects of utility operations is classified as general revenue (e.g., interest earnings). Utility expenditure applies to all expenditures for financing utility facilities, for interest on utility debt, and for operation, maintenance, and other costs involved in producing and selling utility commodities and services to the public (other than noncash transactions like depreciation of assets).

[2] The "cash" basis of accounting, now rare in its pure form, reflects actual cash receipts and outlays during the year. The "accrual" basis reports revenue when it is first earned and measurable (e.g., tax bills are sent) and expenditures when they are incurred. The "modified accrual" basis, on the other hand, recognizes revenues when they become available and measurable while expenditures are recognized when their fund liability is incurred.

Utility revenue and expenditure are reported on a gross basis – i.e., without offsetting revenue with its related expenditure or expenditure with its related revenue.

Liquor Stores Sector: Liquor stores revenue relates only to amounts received from sale of goods and associated services or products. Liquor store expenditure relates only to amounts for purchase of goods for resale and for provision, operation, and maintenance of the stores. Any associated government activity, such as licensing and enforcement of liquor laws or collection of liquor taxes, are classified under the general government sector.

Social Insurance Trust Sector: Insurance trust revenue comprises only (1) retirement and social insurance contributions, including unemployment compensation "taxes" received from employees and other government or private employers, and (2) net earnings on investments set aside to provide income for insurance trusts.[3] Transfers or contributions from other funds of the same government are not classified as insurance trust revenue but rather are reported under special exhibit categories (see Chapters 8 and 9). Insurance trust expenditure comprises only benefit payments and withdrawals of contributions made from retirement and social insurance trust funds. Costs for administering insurance trust systems are classified under the general government sector.

3.6 Noncash Transactions

These finance statistics exclude noncash transactions, such as technical aid, depreciation of fixed assets, payments-in-kind, loan guarantees, and the like.

3.7 Intergovernmental Transactions

A major share of both revenue and expenditure involves intergovernmental transactions. Governments have important fiscal relations among themselves, generally of three types. First, they transfer monies to other governments to enable the receiving government to perform specific public functions or to provide for its general financial support (Type 1). Second, they act as agents for other governments in financial matters (Type 2). And, third, they purchase commodities, property, and services from other governments much as they do from private suppliers (Type 3). Only the first type of transactions is classified by the Census Bureau as intergovernmental revenue and expenditure. How each of these three types of intergovernmental transactions is handled is described below.

3.7.1 Intergovernmental Fiscal Relations (Type 1)

The Census Bureau definition of intergovernmental transactions is limited to monies paid to or received from other governments for performing specific governmental functions or for general financial support, whether the activity is undertaken on behalf of the paying government or whether such funds are regarded as assistance for the support of activities of the receiving government. They are classified in the general government sector no matter their purpose (including utilities). Examples of Type 1 intergovernmental transactions include the reimbursement of one government by another for tuition costs, hospital care, boarding prisoners, construction of public improvements, etc.; grants

[3]The substantial amount of interest paid by the U.S. Treasury to Federal social insurance trust systems (which have all their reserves invested in Federal securities) is excluded from Federal insurance trust revenue since it is an intragovernmental transfer. The principle of eliminating these interfund transactions, however, is not followed in the case of interest paid by a state or local government on any of its own securities held as investments by insurance trust funds it administers – mainly because of the difficulty of identifying such transactions.

in aid; payments-in-lieu-of-taxes, and the like.

Another form of intergovernmental transaction involves the sharing of tax proceeds with other governments, typically by the state government. A portion of the sales taxes, for instance, collected by the state often may be redistributed to the local governments where they were collected. Whenever a government distributes some share of a tax it imposes and collects, its payments are reported as intergovernmental expenditure and the receiving governments' receipts are reported as intergovernmental revenue. As described below in Section 3.7.2, the retention of taxes collected by a government acting as an agent for another is not reported as an intergovernmental transfer.

Generally, loans and advances to other governments are not treated as intergovernmental transactions except for one unique type: "contingent" loans or advances. Similar to grants-in-aid, contingent loans are made by a government to another without any fixed provisions about its repayment. Designed to assist governments in financing public projects, they are used for such purposes as public works, planning advances, housing development, etc. In all these cases, repayment of money advanced depends on certain contingencies or conditions at a later date. Thus, to ensure that these transactions are reported somewhere in the classification system, they are reported as intergovernmental revenue and expenditure rather than as a debt transaction.[4]

Finally, the Census Bureau definition of intergovernmental transactions excludes the sale of marketable securities to other governments (interest bearing or non-interest bearing, if the latter have fixed repayment conditions). Instead, they are treated as investment or debt transactions – i.e., as neither revenue nor expenditure.

3.7.2 Fiscal Agents for Other Governments (Type 2)

Type 2 intergovernmental transactions rarely are classified as intergovernmental revenue or expenditure for Census Bureau purposes.

Governments sometimes act as fiscal agents for other governments. Such relations are excluded from the Bureau's definition of intergovernmental transactions. One common example is the collection and retention of withholdings from employee pay for taxes owed to other governments (such as Federal social security taxes). These are treated as an agency transaction and are excluded entirely from the Bureau's statistics.

Governments also act as fiscal agents to collect taxes that are imposed or authorized by another government. These are reported as taxes by the authorizing government, while for the collecting agent they are excluded except for one circumstance. Specifically, if the collecting government is allowed to retain part of the tax proceeds (e.g., as reimbursement), then that amount is reported as tax revenue by the collecting government. In neither case is the money treated as an intergovernmental transaction.

Thus, if a state government imposes, authorizes, or requires local governments to collect a specific tax and permits the local government to retain all or part of its collections, then this local share is classified as tax revenue of the local government, not as intergovernmental revenue. This same treatment applies where a state government retains part of a locally-imposed tax or where a local government retains a portion of tax proceeds collected for another local government.

[4]Unlike "regular" loans to other governments, contingent loans are not reported as debt transactions for Census Bureau purposes.

3.7.3 Purchases from Other Governments (Type 3)

Type 3 intergovernmental services are not classified as intergovernmental revenue or expenditure unless they involve government services (other than utilities).

Governments may purchase property, commodities, and utility services from other governments much as they do from private vendors. Similarly, governments may levy taxes on facilities of other governments in the same manner as on privately-owned facilities. Because a government's records ordinarily do not distinguish between private and governmental purchasers or taxpayers, these types of transactions are not reported as intergovernmental revenue and expenditure in Census Bureau statistics. Instead, they are treated in the same manner as those involving the private sector – e.g., as tax or charge revenue by the receiving government and as direct expenditure by the paying one.

On the other hand, purchases of governmental services (e.g., police and fire protection) from other governments usually are identified as such in a government's records. Thus, reimbursement of one government by another for such services (other than utilities) is reported as an intergovernmental transaction, to the extent such items are identifiable in the governmental records used by the Bureau.

3.8 Netting Out Duplicative Intergovernmental Transactions

Intergovernmental transactions receive special treatment whenever data for individual governments are grouped, or aggregated, for publication purposes. Transactions among governments within the same group are "netted out." For instance, data for all local governments within a state are net of revenue and expenditure between them (i.e., only amounts to and from the Federal and state governments are included). This procedure avoids the duplication that would result if the published data included both the intergovernmental payment made by the one government and the direct expenditure from that money by the receiving government.

3.8.1 Special Topics: Payments to Other Government Social Insurance Trust Systems

An important exception to the above involves payment by one government to an insurance trust system administered by another, most commonly public employee retirement systems. The payment by one government, either on behalf of its employees who are members of the plan or for general financial support, to another government's insurance trust is treated as a current operation expenditure of the paying government (for the function involved) and as an insurance trust revenue of the receiving government, not as intergovernmental transactions.

The purpose of this treatment is to avoid an imbalance between intergovernmental revenue and expenditure. Since intergovernmental revenue and expenditure are "two sides of the same coin," in theory the two should always equal. Also, contributions for an insurance trust system are insurance trust revenue so long as they come from outside the administering government. To avoid the imbalance between intergovernmental revenue and expenditure that would result if the payment of the contribution were treated as an intergovernmental expenditure and the receipt were treated as an insurance trust revenue, neither of these transactions is treated as intergovernmental.

3.9 Internal Transfers

In general, the Census Bureau reports revenue and expenditure that originate from or are directed outside the government. Internal transactions within the government are excluded. There are so many variations

to this general rule, however, that the topic warrants special consideration.

3.9.1 Interfund Transactions

As noted above, governments record their financial transactions through accounting devices called "funds." The different funds that account for a government's finances have fiscal transactions with each other as well. These are reported in accounting reports just like those made outside the government. Take, for instance, funds established to record the provision of services or commodities to other agencies of the same government, such as motor pools, central computer centers, central stores, garages, asphalt plants, and the like. In other cases, the general fund may transfer money to a construction (bond) fund or loan money to special revenue fund. The first types of interfund transactions, commonly called intragovernmental service funds, are included in Bureau statistics. The second type is excluded from Census Bureau statistics.

Transfers to Own Utility: A government utility often provides services or commodities to other agencies of the same government. To the extent they are identifiable, these transactions are reported as an expenditure of the activity consuming the utility services (rather than as a utility expenditure) and any corresponding revenue is excluded from utility revenue.

Other Interfund Transactions: Accounting procedures focus on the activities of funds as self-contained entities. Since the Census Bureau treats the entire government as a single financial entity, its statistics represent a consolidation of accounting funds. To avoid duplicating revenues and expenditures among these funds, the Census Bureau "nets out" such interfund transactions. Thus, revenues are reported when they "enter" the government for the first time and expenditures are reported when they "leave" the government.

3.9.2 Intragovernmental Service Funds (Revolving Funds)

Interfund transfers also arise when one department or agency furnishes services or commodities to others on a reimbursable basis. Such services often are reported through intragovernmental service (revolving) funds. These types of internal transfers are included in Census Bureau finance statistics by reporting the payments to intragovernmental service funds as an expenditure of the activity benefited – i.e., of the agency using the service or commodity. Since the finances of the intragovernmental service funds themselves are not compiled (with the exceptions cited in the next paragraph), then there is no duplication of data. Rather, the financial transactions of the intragovernmental service funds are "functionalized" according to the activities using its services or commodities. Unallocable amounts are reported as expenditures "not elsewhere classified" (NEC).

As a general rule, the financial activities of intragovernmental service funds themselves are excluded from Census Bureau statistics since they are, in effect, reported with the activity paying for the service or commodity. However, certain financial transactions of such funds, primarily those that occur with those outside the government, are included in Census Bureau data on revenue and expenditure. These consist of:

- The sale of surplus vehicles or other items to outsiders are reported as revenues.

- Intergovernmental grants directly from another government – i.e., those not passing through another fund of the government – also are reported as revenue.

- Major capital outlays of intragovernmental service funds should be double-coded as a capital

outlay expenditure and as an offset to current operations expenditure. If known, they are recorded under the applicable function. (For instance, major purchase of police cars by a local government motor pool fund should be double-coded as *Police Protection – Capital Outlay Other Than Construction*, code G62, and as a deduction from *Police Protection - Current Operations*, code E62). If the function is unknown, they are reported under *Other and Unallocable*, code *89. The purpose of this practice is to avoid understating a government's capital outlays.

• Total salaries and wages paid during the fiscal year are included in the exhibit code, ZOO.

3.9.3 Interdepartmental Charges

As government financial management systems have become more sophisticated so have they grown more complicated. Certain internal transfers involving services provided to other departments no longer occur in special funds but are built into the regular accounts through a method of additions and subtractions.

Agencies often provide services to other departments on a reimbursable basis. Traditional ones, like motor pools or computer services, generally operate though intragovernmental service funds. Less obvious are legal offices, personnel and administrative departments, or even staff in functional agencies like highways.

Some governments' accounting systems report the finances for these services as expenditure of the activity receiving the service and as deductions to expenditures of the agency providing the service. The Bureau reports expenditures as shown in these reports without modification. It should be noted that such systems may understate activities, such as government administration, that are service providers to other departments.

3.10 Agency, Private Trust, Investment, and Other Transactions

Government accounting records from which the Census Bureau derives statistics contain a wide variety of fiscal transactions, not all of which fall within the scope of the finance survey. This section discusses those types of transactions that are excluded.

3.10.1 Agency Transactions

Agency transactions are excluded from financial statistics produced by the Census Bureau. Agency transactions represent financial activities involving the receipt, holding, and disbursement of monies which a government undertakes for other governments in the capacity of an agent – e.g., transactions undertaken without discretion on the part of the agent government. Generally, monies received from other governments or individuals for transmittal to other governments or individuals are classified as agency receipts, and the corresponding payments as agency disbursements, if the intermediate government has no discretion in determining either the amounts of such payments or the recipients.[5]

The most common agency transaction involves the collection of taxes for other governments. Often,

[5] The "pass-through" of Federal aid through the state governments to local governments, however, is treated as regular finance activities since the state usually has discretionary authority over distribution of the funds.

for example, a county government will collect general property taxes on behalf of all governments levying such taxes within its area. These collections are classified as tax revenue of the final recipient governments (i.e., the ones levying the tax) and are omitted from the finance statistics of the county government (i.e., the collecting agent). Another typical example is the withholding of Federal Social Security taxes from public employees salaries and their disbursement to the Federal Government. Still another type is where the county government acts as an agent of the state government in distributing aid to other local governments within its boundaries.

Census Bureau finance statistics also exclude transactions arising from agreements between the Federal Government and state or local governments whereby the latter agree to serve as agents in transmitting Federal payments to individuals (e.g., various Federal benefit payments to veterans). These are reported as Federal Government direct expenditure and as agency transactions of the state or local government.

3.10.2 Private Trust Transactions

Private trust transactions are excluded from financial statistics produced by the Census Bureau.

Private trust transactions comprise accounting funds that receive and disburse assets held in trust for a particular individual or corporation, such as when a government assumes the obligation of applying money to a specified use benefiting the private party for whom the fund was established, or when the government holds deposit or other monies pending fulfillment of certain conditions or pending determination of ownership. Essentially, they are funds through which the government acts as a trustee or agent on behalf of private individuals or corporations.

3.10.3 Investment Transactions

With one major exception, described below, the receipts and payments arising from investment transactions are excluded from Census finance statistics except for any recorded profit or loss when they are sold.

Investment transactions involve the purchase and sale of securities for investment purposes, extension of loans to individuals, and receipts resulting from repayment of such loans. Their exclusion does not apply to purchase and sale of realty or tangible personal property not for investment nor to the extension and repayment of contingent loans and advances to another government.

3.10.3.1 Special Topics: Investment Transactions for Public Employee Retirement Systems

The investment activity engaged in by public employee retirement systems warrants special handling in Census Bureau statistics on government finance. Public employee retirement systems (described in detail in Chapter 8) carry the largest amounts of cash and security holdings of any government entities (parent governments or dependent agencies). The methodology used to report on investment transactions includes measuring both realized and unrealized gains (or losses) on investments. Realized gains or losses are reported as noted above – at the time of sale, as a recorded profit or loss. However, unrealized gains or losses also are included in Census Bureau statistics for these systems, following the accounting standards as promulgated by GASB (see Section 3.4.3). These unrealized gains or losses are treated as revenue in the Census Bureau classification system. Sections 8.4.1 and 8.4.1.1 describe this methodology in more detail. Another feature of the statistics for public employee retirement systems is that the value of real property is included in Census Bureau exhibit statistics on cash and security holdings. This

special case is described in Section 8.4.3.

3.10.4 Adjustment and Correction Transactions

Census Bureau finance statistics represent transactions that are net of refunds or other correcting adjustments. Adjustment transactions are not reported separately in Census statistics; instead, they reduce or increase amounts reported as revenue and expenditure. The exact treatment of such transactions depends on when the refund or correction occurred – i.e., whether it was in the same fiscal year or a prior fiscal year. Chart 3.A summarizes the rules for reporting refund and correction adjustments.

Chart 3.A Rules for Reporting Refund and Correction Adjustments

**Type of data**	**Refund or Correction In Same Fiscal Year**	**Refund or Correction for Prior Fiscal Year(s)**	
		**Taxes**	**Other**
Revenue	Deduct from gross receipts.	Deduct from gross taxes.	Report as expenditure (E89).
Expenditure	Deduct from gross disbursements.	(X)	Report as revenue (U99).

(X) means not applicable.

Same fiscal year adjustments: Revenue amounts represent gross collections less any refunds paid out during the same fiscal year. Expenditure amounts represent gross disbursements less any amounts refunded for payments made in the same fiscal year. Also, interest earnings on investments and interest payments on debt are adjusted for accrued interest on securities purchased and on debt obligations issued, respectively.

Prior fiscal year adjustments: The refund of an expenditure made in a prior fiscal year is treated as *Miscellaneous General Revenue* (code U99). The refund of a revenue other than taxes is treated as *Other and Unallocable* (code E89). Refunds of prior year tax collections are treated as offsets to current year tax collections.

3.11 Miscellaneous Topics

This Section covers miscellaneous financial transactions and how they are reported in Census Bureau statistics.

3.11.1 Suspense Transactions

Suspended receipts and expenditures are not classified as either revenue or expenditure. Their treatment depends on the resolution of the dispute creating the suspense transaction.

Suspense transactions arise when a government's receipts or expenditures are held in "suspense" until the resolution of the condition or event that halted the transaction. For instance, taxes paid under protest are a common type of suspense transaction. If the situation is resolved in the government's favor, then the receipt or expenditure is recorded in that period. If the situation is not resolved in the government's favor, then the transaction is never reported for Census Bureau purposes.

3.11.2 Capital and Operating Leases

An increasingly popular finance mechanism, especially for capital outlays, is the use of operating and capital leases. The latter often replace the more traditional method of funding capital projects by issuing bonded debt.

Leases are treated in the Census Bureau classification system as follows:

- Effective with fiscal year 1988 data, payments on capital leases are treated as capital outlay expenditure, either for the purchase of land and existing structures ("G" code) or for purchase of equipment ("K" code for states only) depending on the situation. These include payments representing both principal and interest, which may be separate in a government's fiscal report. Examples include lease-purchase agreements, installment purchase contracts, and capitalized leases. Prior to fiscal year 1988, these payments were classed as current operations. The present treatment recognizes capital leases as a type of "pay-as-you-go" funding technique.

- Payments on operating leases are treated as current operations expenditure ("E" code). This represents no change in classification.

- Capital and operating leases are not treated as debt for Census Bureau purposes even if shown as liabilities in a government's fiscal report or debt schedule.

3.11.3 Depreciation

Depreciation is excluded from Census Bureau statistics on government finance. This treatment differs from the standard accounting guidelines found in GASB. However, it is internally consistent for this Census Bureau classification system. Similarly, Census Bureau statistics on cash and security holdings exclude the value of land, buildings, roads and other capital assets.[6]

3.11.4 Anomalies of Government Finance Statistics

The classification system for government finances described above has resulted in several anomalies that deserve mentioning.

- All intergovernmental revenue is, by definition, included in the general government sector, including the utility intergovernmental codes first used in the fiscal year 1988 finance survey. Utility disbursements from these monies, however, are reported in the utilities sector.

[6] One exception exists for statistics on public employee retirement systems, however. The value of real estate is included as an exhibit statistic for these systems. See Chapter 8.

- Insurance trust systems often hold for investment purposes securities issued by their own government. Since these types of transactions are generally not identified in a government's records, the interest paid by the government for such securities is reported as interest expenditure (e.g., at *Interest on General Debt*, code I89) and the interest received by the insurance trust system is reported as earnings on investments. (The exception is the interest earnings of Federal Government insurance trusts since all their investments are in U.S. securities.)

3.12 Special Topics: Statistical Nature of Data

Although the original sources for finance statistics are accounting records of governments, the data derived from them are purely statistical in nature. Consequently, the Census Bureau statistics on government finance cannot be used as financial statements, or to measure a government's fiscal condition. For instance, the difference between a government's total revenue and total expenditure cannot be construed to be a "surplus" or "deficit."

There are several reasons why these survey data are not suitable for measuring the financial condition of a government, any of its sectors, or any of its dependent agencies:

- The Census Bureau intentionally excludes several important accounting measures from its statistics. One example involves public employee retirement systems, which exclude measures of future liability, future revenue streams, and all related measures of future solvency (such as the potential amount of unfunded liabilities). These cannot be calculated from Census Bureau statistics.

- The Census Bureau program develops these data to measure the economic activity of state and local governments in general. The definitions used in Census Bureau statistics about governments can vary considerably from definitions applied in standard accounting reports.

- Definitional differences can include those of coverage (what constitutes a government entity), functional activity, financial transaction (revenue, expenditure, indebtedness, and asset), or measurement (cash versus accrual accounting, or asset valuation procedures).

- Census Bureau data include the operations of dependent agencies whose finances are reported outside those of the parent government.

Sections 8.4.7 and 8.4.8 contain additional information on this topic.

3.13 Special Topics: Classification Issues for Washington, DC

The District of Columbia, within which the city of Washington is located, is classified as a separate county area and as a separate state area in the Census Bureau's population statistics programs. The government entity of Washington, DC, however, is classified as a municipal government for statistics on government finance and employment. It is a unique government entity in many respects, having characteristics of both state and local governments. It also has a special fiscal relationship with the Federal Government.

As a result, there are several finance classification categories that apply solely to Washington, DC and to no other local governments. These are detailed throughout this *Classification Manual* and should be understood thoroughly:

1. Revenue object code “C” (intergovernmental revenue from the state government) does not apply for Washington, DC, since it exists outside the domain of any state government.

2. Expenditure object code “L” (payments to a state government) similarly does not apply.

3. Like state governments, Washington, DC participates in two programs within the public welfare functional expenditure category that involve making intergovernmental payments to the Federal Government. Thus, codes S67 and S74 apply to Washington, DC – the only local government for which that applicability is true. See Chapter 5 for additional information.

4. Washington, DC also administers a Federal-state cooperative unemployment compensation insurance program within its borders. Hence, Washington, DC has financial statistics pertaining to *Social Insurance Administration*, code *22, and to the Unemployment Insurance sub-sector of Social Insurance Trust Systems. The following state government finance codes also apply to Washington, DC:

    • General sector intergovernmental revenue code B22 and expenditure codes E22, F22, and G22 (See Chapters 4 and 5 for additional detail).

    • Social insurance trust codes for the Unemployment Compensation system: revenue (Y01, Y02, Y04), expenditure (Y05, Y06), and cash and security holdings (Y07, Y08). See Chapter 9 for details.

5. Washington, DC does not operate a workers’ compensation system that meets the Census Bureau definition of a social insurance trust system. The DC system, like that of the Federal Government, consists of “pay-as-you-go” financial transactions. See Chapter 9 for details.

6. In the general government sector, revenue exhibit code CGR and expenditure exhibit code CGE apply to Washington, DC as well as to state governments. These codes pertain to the tobacco lawsuit financial settlement transactions between the major tobacco companies and the state governments.

7. While lottery system revenue applies to all governments within the Census Bureau’s classification system, as of Fiscal Year 2005 Washington, DC is the only local government that operates a lottery. Hence, Washington, DC is the only local government that reports regular revenue code U95. In addition, lottery system exhibit statistics apply only to state governments and to Washington, DC, by definition. Net Lottery Revenue, and all finance exhibit codes associated with lotteries, are reported for Washington, DC only. See Chapter 10 for details.

3.14 Joint Financial Activities of Governments

As noted in Section 1.5, government units often cooperate to provide a specific service or activity. Section 1.5 explained three methods for the classifying joint financial activity. The first two methods, summarized below, are straightforward. This section further explains the third methodology.

1. Governments might establish an independent special district to carry out the activity, in which case the related finances and employment are assigned to that government unit. Their financial transactions with the special district are treated as intergovernmental revenue and expenditure.

2. One of the governments might be solely responsible for administering the activity, but the other governments share its financial support. In such situations, all its activities are assigned to the administering government with the other governments' shares being reported as intergovernmental transactions.

3. The participating governments might create a separate organizational body that is neither independent nor dependent on any one member, but is jointly administered by all of them. These types of joint activities provide special problems in a classification system whose focus is the individual government entity. Such joint activities, therefore, are classified on the basis of the circumstances involved in each case. The Census Bureau also considers the magnitude of financial activity involved in deciding on to classify these activities.

For this third type of situation, the Census Bureau has three options for classifying financial activity. As a general rule, the first two options (3a and 3b, below) are used for large joint agencies, while the third option (3c below) is usually applied for small joint agencies. Finances of a joint inter-local agency having substantial financial activity or which issues substantial debt that is not in the name of any of the participating governments might be classified as one of the following:

3a. The joint agency is assigned as a dependent agency of one of the participating governments. Its finances are classified in the same manner as the second case cited above.

3b. The joint agency can be treated (classified) as if it were an independent special district government.

3c. A joint inter-local agency not having substantial financial activity or employment is allocated to each of the participating governments to the extent of their respective participation, as follows:

- Revenue of each parent government should include any taxes or other receipts it collects to turn over to the joint agency or activity.

- Borrowing and debt of each parent government should include any debt it issues or guarantees on behalf of the joint agency or activity.

- Direct expenditure of each parent should include any amount it pays (e.g., for salaries and other expenses) on account of, and any contributions it makes directly to, a joint agency or activity. Such amounts are classified according to the function involved and as appropriately as possible by character and object.

- Parent government data should exclude any revenue or expenditure of the joint agency or activity, as such, and any joint agency debt not issued in the name of, or guaranteed by, the parent government.

3.15 Tables

Table 3.1, Summary of Government Fiscal Year Ending Dates, shows the fiscal year ending dates for the Federal and state governments, as well as a frequency distribution for local government fiscal year ending dates, by month. Table 3.2, Examples of Accounting Funds and Their Treatment in Census Bureau Statistics on Government Finances, is a useful reference for identifying the most common types of accounting funds used by government units and determining whether they are in-scope or out-of-scope

for Census Bureau statistics on government finances.

Table 3.1 Summary of Government Fiscal Year Ending Dates		
**Level of Government**	**3.1 Fiscal Year Ending Date**	**Number of Governments**
Federal Government	September 30	1
State Governments (unless noted below)	June 30	46
Alabama	September 30	1
Michigan	September 30	1
New York	March 31	1
Texas	August 31	1
**Local Governments**	**Month of Fiscal Year Ending Date (any day):**	**Approximate Percentage of Total Number of Governments**
Fiscal years ending in -	January	under 1%
Fiscal years ending in -	February	2%
Fiscal years ending in -	March	5%
Fiscal years ending in -	April	3%
Fiscal years ending in -	May	1%
Fiscal years ending in -	June	37%
Fiscal years ending in -	July	1%
Fiscal years ending in -	August	3%
Fiscal years ending in -	September	6%
Fiscal years ending in -	October	under 1%
Fiscal years ending in -	November	under 1%
Fiscal years ending in -	December	40%

Source: Governments Integrated Directory, 2002 Census of Governments

Table 3.2
Examples of Accounting Funds and Their Treatment in Census Bureau Statistics on Government Finances

This chart lists the most common types of accounting funds found in GASB financial reports and describes whether their finances are reported for Census Bureau purposes.

Type of GASB Accounting Fund:	Classify its finances for census statistics?
Agency	Varies according to exact fund involved
Bond	Yes
Capital Projects	Yes
Debt service	Yes
Enterprise	Yes
Expendable Trust	Yes
Fiduciary	Varies according to exact fund involved
General	Yes
General Fixed Assets Accounts	No*
General long-term debt Accounts	No*
Internal Service	No
Intragovernmental Service	No
Investment Trust	No
Nonexpendable Trust	Varies according to exact fund involved
Pension Trust	Yes**
Permanent Funds	Yes
Private Purpose Trust	Varies according to exact fund involved
Self-insurance	Yes
Sinking	Yes
Special Revenue	Yes
Trust and Agency	Varies according to exact fund involved

*These are not true accounting funds; rather, they are special displays that may contain data that are useful for Census Bureau purposes (especially the general long-term debt accounts).

** However, the treatment will vary depending on whether the pension trust represents a public employee retirement system as defined for Census Bureau purposes, whether the government has a '"pay-as-you-go" pension plan, or whether there is some other pension arrangement in place. See Chapter 8 for additional information.

Chapter 4. Revenue

Introduction

This chapter explains the concept of revenue as it is used in the government finance statistics. This includes the concepts of governmental revenue, revenue transactions between governments, and funds of the same government. This chapter also contains the complete definition of revenue categories used in the classification system. Chapters 8 and 9 contain topics related to the structure and purpose of “receipts” for retirement and social insurance trust systems.

4.1 Revenue Definition

Revenue is defined as all amounts of money received by a government from external sources (i.e., those originating from “outside the government”), net of refunds and other correcting transactions, proceeds from issuance of debt, the sale of investments, agency or private trust transactions, and intragovernmental transfers.

4.1.1 Coverage Issues: Revenue

Revenue comprises amounts received by all agencies, boards, commissions, or other organizations categorized as dependent on the government concerned (see Chapter 1). Stated in terms of the accounting procedures from which these data originate, revenue covers receipts from all accounting funds of a government, other than intragovernmental service (revolving), agency, and private trust funds.

4.1.2 Measurement Issues: Revenue

The methodology used to measure revenue involves addressing four issues: refunds and correcting transactions; timing; aggregation and tabulation; and government enterprise activities. Each is explained below.

4.1.2.1 Measurement Issues: Refunds and Correcting Transactions

Revenue data are adjusted for refunds and other correcting transactions. However, the rules for refunds of taxes differ from those for other revenues. See Section 4.3.1.2 for details.

4.1.2.2 Measurement Issues: Timing

Revenue is measured over the full fiscal year of the government (see Section 3.2). Revenue received at any time during the fiscal year is included in the measurable amounts reported. Thus total property tax revenue reflects such tax collections received by the government over the full twelve months of its fiscal year. As discussed in Chapter 3, governments often report revenue, and keep their official accounting records, in terms of a modified accrual form of accounting. Where this happens, Census Bureau statistics reflect this accounting approach, even though it does not correspond exactly to the concept of cash received during the fiscal year.

4.1.2.3 Measurement Issues: Aggregation and Tabulation

Aggregate statistics for an individual government reflect the revenue of the parent government and all of its dependent agencies. However, flows of funds between these entities are considered

internal transfers and are excluded, by definition, from revenue totals. These are treated as intragovernmental revenue and are excluded in much the same way as most intragovernmental service (revolving) funds.

Tabulated statistics on revenue for multiple governments reflect the fiscal years of the governments being summed. Since these fiscal years differ, total statistics (such as for all local government in a state, or all townships nationally) reflect a mix of fiscal periods. For the annual surveys of government finance, the Census Bureau makes no effort to adjust aggregates so that they represent a standard time period. The Quarterly Summary of State and Local Tax revenue is an exception, however. For this survey, statistics represent calendar year quarters and are also aggregated for twelve month periods, rather than government fiscal years.

4.1.2.4 Measurement Issues: Government Enterprises Activities

Revenue of business-type activities of governments (utilities and other commercial or auxiliary enterprises) is reported on a gross basis. That is, related expenditures are not deducted from their revenues to derive net revenue amounts.[1] In this regard, the Census Bureau uses a methodology that differs from that used by some other statistical agencies. The most notable of these is the Bureau of Economic Analysis (BEA) and its treatment of government enterprise activities for purposes of the National Income and Product Accounts (NIPAs). The BEA essentially reports government enterprise activities on a net basis.

4.1.3 Exclusions from the Revenue Definition

The definition used for revenue, in combination with coverage and measurement issues, leaves certain types of government revenue to be out-of-scope for Census Bureau surveys. The following types of receipts are excluded from revenue:

- Taxes and other amounts paid under protest and held in suspense accounts subject to possible refund. Such amounts are not reported as revenue unless and until the protest is decided in the government's favor (see Section 3.11.1).

- Proceeds from borrowing, whether short- or long-term, except contingent loans and advances which are reported as intergovernmental revenues (see Section 3.7.1).

- Recoveries or refunds of amounts spent in the same fiscal year, which are deducted from expenditures (see Section 3.10.4).

- Proceeds from the sale of investments and the repayment of loans, except for contingent loans as mentioned above. Any recorded profit or loss from the sale of investments, however, is reported as revenue or expenditure, respectively.

- Transfers from agencies or funds of the same government (see Section 3.9).

- Agency or private trust transactions, where the government is acting on behalf of others (see Section 3.10).

[1] An exception exists for *Net Lottery Revenue*, code U95, for which the costs of prizes are deducted from gross receipts. See Section 4.9 and the explanation of code U95 for details.

- Noncash transactions, such as receipt of technical services, commodities, property, noncash gifts or bequests, and other “receipts-in-kind.”

4.2 The Four Sectors of Government – Revenue Issues

All government finance statistics, including revenue, are categorized within the four basic sectors of government, as explained in Chapter 2. As a result, there are four basic categories of revenue, as shown below with their subcategories (referred to as type of revenue in Census Bureau statistics):

- General Revenue – pertains to the general government sector and is classified by Type
  - Taxes
  - Intergovernmental Revenue
  - Current Charges
  - Miscellaneous General Revenue

- Utility Revenue – pertains to the utility sector and is classified by utility (Type = Current Charges, by definition)

- Liquor Stores Revenue – pertains to the liquor stores sector (Type = Current Charges, by definition, except for exhibit codes)

- Social Insurance Trust Revenue – pertains to social insurance trust systems and is classified by system and by Type.

Sections 4.3 through 4.6 contain explanations of how revenue statistics are categorized in accordance with these four sectors, by type of revenue.

4.3 General Revenue

General revenue comprises all revenue except that classified as liquor store, utility, or insurance trust revenue. The basis for this distinction is the nature of the revenue source involved, not the fund or administrative unit established to account for and control a particular activity.

There are four types of revenue within general revenue sector: taxes, intergovernmental revenue, current charges, and miscellaneous general revenue.

4.3.1 Taxes

Taxes are compulsory contributions exacted by a government for public purposes, other than for employee and employer assessments and contributions to finance retirement and social insurance trust systems and for special assessments to pay capital improvements. Tax revenue comprises gross amounts collected (including interest and penalties) minus amounts paid under protest and amounts refunded during the same period. It consists of all taxes imposed by a government whether the government collects the taxes itself or relies on another government to act as its collection agent.

4.3.1.1 Special Topics: Assignment of Tax Revenue

For classification decisions involving the assignment of taxes, the Census Bureau typically

examines three factors – imposition, collection, and retention (or distribution) of tax proceeds. The general rule is that tax collection amounts are assigned to the government controlling two of the three factors. In determining the assignment of taxes, the Census Bureau gives primary consideration to the government actually imposing the tax and usually credits that government with the tax collection. The government imposing a tax is the jurisdiction whose governing body adopts the legislation or ordinance specifying the type of tax, scope, and rate and requiring its payment. Generally, if another government collects a tax for the levying unit, then that government is considered to be acting as a collecting agent and is credited only with any amount it retains as reimbursement for administration or other costs. These guidelines apply to all taxes, whether levied under general municipal powers, charter powers, or specific state legislative authority.

The following examples are relevant.

- A locally-imposed and collected tax whose ordinance or statutory authorization specifies a distribution of funds to other jurisdictions (either mandatory or optional) is credited to the imposing government. In such cases, payments to the other units are treated as intergovernmental transfers.

- Taxes adopted by a government in response to requests from other jurisdictions who may then share in the proceeds also are credited to the imposing government, the distribution being treated as intergovernmental transfers.

- A state-mandated tax required to be levied by a local government and collected by that government is credited to the local government imposing the tax.

- Similarly, that portion of a state-enacted tax which is locally collected and retained is credited as a tax of the collecting agency. This is true even if there is a voluntary sharing of the tax collections, and these transactions are classified as intergovernmental transfers. State or local government legislation which provides that the imposing government waive credit for part or all of the amounts transferred to other jurisdictions does not alter these guidelines.

- A state tax collected locally, and redistributed in accordance with state statute or administrative directive, is the most complex of taxes to assign. In recent years, several states have used their authority to redistribute or redirect property taxes designated for educational purposes. If the state imposes the tax, such as establishing a base millage for a property tax dedicated to public schools, AND there is a mandatory redistribution to other local governments of the taxes collected based on a state-controlled formula, the Census Bureau assigns the tax to the state government. In this example, the state controls two of the three factors used by the Census Bureau to determine tax assignment – imposition and distribution. The local collection of the tax is merely an agency transaction.

The examples below illustrate the various types of arrangements and how they are handled in this classification scheme:

- For a state government, local collection of state-imposed taxes is classified as state tax revenue.

- State government distribution of its tax proceeds to local governments (e.g., on a formula basis) is treated as intergovernmental expenditure of the state and as intergovernmental revenue of the local governments. This is true even for amounts designated as the "local share" of state-imposed taxes so long as the tax proceeds are collected by the state or transferred to the state by local government collection agents before their distribution.

- On the other hand, if the state collects a tax imposed by local governments, the collection and distribution to the imposing local governments is treated as an agency transaction; that is, the receipts are reported entirely as tax revenue of the local governments and not as either a state tax or state intergovernmental expenditure. These situations occur where a local government might impose a "piggyback" tax (always with state approval) onto a state tax of same type. Common examples are local option sales taxes and local option income taxes.

- Proceeds from taxes imposed by one local government but collected for it by another are reported as tax revenue of the imposing government, not the collecting one. Monies retained as a collection fee, however, are reported as tax revenue of the collecting government.

4.3.1.2 Refunds of Taxes

Refunds for taxes originally paid in either the current or prior fiscal years are deducted from gross collections in the same year refunded. Discounts to taxpayers for prompt payment or for collecting consumer taxes also are deducted from gross tax revenue. The cost of collecting and administering taxes, however, is reported as an expenditure, not as an offset to taxes.

4.3.1.3 Taxes on Government Utilities

Taxes usually are imposed on publicly-owned utilities, as well as on private ones. These amounts are reported as tax revenue for Census Bureau purposes. Payments-in-lieu-of-taxes from a utility operated by another government, however, are treated as intergovernmental revenue. (Payments-in-lieu-of-taxes from a private utility are reported under *Miscellaneous General Revenue, NEC*, code U99.) Both taxes and payments-in-lieu-of-taxes received by a government from a utility it operates are treated as an interfund transfer and are not reported as either revenue or utility expenditure.

4.3.2 Intergovernmental Revenue

Intergovernmental revenue comprises monies from other governments, including grants, shared taxes, and contingent loans and advances for support of particular functions or for general financial support; any significant and identifiable amounts received as reimbursement for performance of governmental services for other governments; and any other form of revenue representing the sharing by other governments in the financing of activities administered by the receiving government. All intergovernmental revenue is reported in the general government sector, even if it is used to support activities in other sectors (such as utilities).

Intergovernmental revenue excludes amounts received from the sale of property, commodities, and utility services to other governments (which are reported in different revenue categories). It also excludes amounts received from other governments as the employer share or for support of public employee retirement or other insurance trust funds of the recipient government, which are treated as

insurance trust revenue (see Chapter 8).

Intergovernmental revenue is classified by function and by the level of government where it originated (i.e., Federal, state, or local). The transfer of Federal aid through the state government to local governments is reported as state intergovernmental revenue at the local level.

4.3.3 Current Charges

This category comprises charges imposed for providing current services or for the sale of products in connection with general government activities. Amounts designated as current charges are reported on a gross basis without offsetting the cost to produce or buy the commodities or services sold. Utility service charges are excluded here and reported under *Utility Revenue*.

For the Federal Government, this category includes revenue from premiums related to non-social insurance programs such as crop and farm mortgage insurance, home mortgage insurance, and the like.

4.3.4 Miscellaneous General Revenue

This category comprises all other general revenue of governments from their own sources (i.e., other than liquor stores, utilities, and social insurance trust revenue).

Effective with the 1988 survey, interest revenue necessary to pay the interest expenditure on all public debt for private purposes is now reported under *Interest Earnings*, code U20. Previously, this treatment was limited to mortgage revenue bonded debt and was classified under *Rents,* code U40.

4.4 Liquor Stores Revenue

Liquor store revenue comprises only receipts from sales and associated services or products of liquor stores owned and operated by state and local governments. It excludes any application of general revenue for liquor store operations as well as receipts from licenses or other liquor taxes collected by liquor stores or systems (including general sales tax collections). All taxes collected through liquor store operations are classified as tax revenue.

4.5 Utility Revenue

Utility revenue comprises receipts from sales and directly related services and by-products of the four types of state and local government utilities recognized by the Census Bureau: water supply, electric power, gas supply, and public mass transit systems. Utility revenue is reported on a gross amount without deducting its related expenditures. Utility revenue is categorized according to the type of utility involved – water supply, electric power, gas supply, and public mass transit systems.

Utility revenue includes contributions from other governments for construction of a joint utility project (“payments-in-aid-of-construction”) or for debt service of a utility consortium IF the contributions are treated as part of the utility's basic rate structure. (These situations generally arise when a governments electric power utility is a major provider of electricity to other utilities who redistribute it to the ultimate consumers.) If not, then the revenue is reported under intergovernmental revenue.

Utility revenue excludes any identifiable amounts received from sales to the parent government. Assessments or contributions of utility employees that are received by public employee retirement

systems are classified as social insurance trust revenue.

Utility revenue also does not reflect any application of general revenue to utility purposes nor does it include any of the following receipts even when received by utility agencies or funds: interest on investments; rents from leases and other earnings from nonoperating property; grants, shared taxes, or any other form of intergovernmental aid (not to be confused with sales to other governments as customers); taxes imposed by public utilities; and special assessments for utility capital improvements. These are classified in other revenue categories.

4.5.1 Special Topics: Intergovernmental Revenue Codes for Utilities

Effective with fiscal year 1988 data, the Census Bureau created intergovernmental revenue codes for utilities. Despite the fact that these categories possess utility function codes (*91, *92, *93, *94), they are classified as general revenues. Note that these categories are used to record intergovernmental transactions that were being reported previously under other intergovernmental revenue codes (i.e., B47, B89, C47, C89, D47, or D89). Sale of utility services or commodities to other governments continues to be recorded as utility revenue (i.e., A91, A92, A93, or A94).

4.6 Social Insurance Trust Revenue

Social insurance trust revenue consists of two major types: a) contributions distinctively imposed for the support of public employee retirement and social insurance systems, and b) net earnings on investments

Insurance trust revenue excludes contributions from the government which administers the system, whether they are paid on behalf of its employees covered by the plan or for supplemental support.[2] Also excluded from insurance trust revenue and classified as general revenue are tax receipts credited directly to insurance trust funds and intergovernmental aid, such as grants and shared taxes for support of insurance trust activities.[3] Excluded entirely as revenue (insurance trust or general) are proceeds from borrowing for insurance trust purposes.

Social insurance trust revenue is classified according to the major types of insurance trust systems recognized by the Census Bureau and by type of receipt (contribution or investment earnings).

4.7 Exhibit Statistics for Revenue – Revenue versus Receipts

As noted in Chapter 2, the Census Bureau uses special exhibit codes and derived statistics in its classification system. These provide additional detail about financial transactions that are used to develop special tabulations of government activities.

These special codes are labeled “receipts” to distinguish them from the “revenue” categories used in the Census Bureau’s classification system. These receipt codes are explained briefly in this chapter under their related revenue category. Additional information is contained in Sections 8.2, 9.3, and 10.4.

---

[2]Such contributions by the administering government, however, are recorded under special exhibit codes and included in insurance trust revenue when data are published solely for insurance trust systems.

[3]On the other hand, funds from other governments which represent the latter’s employer share of contributions to an insurance trust system to which their employees are members are classified as insurance trust revenue.

The exhibit codes and derived statistics related to revenue are noted in the description sheets for revenue categories (Section 4.9), as well as in the tables that accompany this chapter.

4.8 Explanation of Statistical Codes Used to Identify Revenue Categories

A three character code is used to identify revenue codes. The first character is alphabetic and designates the sector and type of revenue. The second two characters are generally numeric and designate the specific revenue item within the sector and type category. In the key below, "nn" refers to the numeric code assigned to each type of revenue.

- General revenue
  - Taxes = Tnn
  - Intergovernmental Revenue (includes such revenue for utility purposes)
    - From Federal Government = Bnn
    - From state governments = Cnn
    - From local governments = Dnn
  - Current Charges = Ann
  - Miscellaneous General Revenue = Unn (for regular statistics)
Znn or Cnn (for exhibit statistics)

- Utility Revenue = Ann (since all utility revenue represents charges for services rendered, by definition)

- Liquor Stores Revenue = Ann (for regular statistics)
Znn (for exhibit statistics)

- Social Insurance Trust Systems Revenue
  - Federal Systems = Xnn or Ynn
  - Employee Retirement = Xnn or Znn
  - Unemployment Compensation = Ynn
  - Workers' Compensation = Ynn
  - Other Federal and State Social Insurance Trust = Ynn

4.9 Description Pages for Revenue Categories

The following pages contain the detailed revenue code categories used in the Census Bureau's statistical program on government finances. They include definitions, references, and explanations, plus examples where appropriate. Codes representing exhibit statistics or derived statistics are labeled appropriately. Otherwise, all items listed are regular codes.

In Census Bureau statistics on government finances, taxes are compulsory contributions exacted by a government for public purposes, other than from special assessments for capital improvements and from employee and employer contributions or “taxes” for retirement and social insurance systems.

Comprises amounts received (including interest and penalties) from taxes (1) imposed by a government and collected by that government or (2) collected on its behalf by another government serving as its agent. See Section 4.3.1 for additional explanations.

Excludes protested amounts and discounts; special assessments for property improvements (use *Special Assessments*, code U01); compulsory contributions to social insurance systems even if labeled a tax – e.g., Federal Social Security tax, unemployment insurance and workers’ compensation payroll taxes, etc. (report at appropriate *Insurance Trust Revenue* code); taxes collected by a government as an agent for another government which actually imposed the tax (agency transaction) except amounts retained as a fee or shared tax.

Refunds of taxes paid are deducted from gross tax receipts even if they were reported as tax revenue in a prior fiscal year.

Taxes are classified according to the type of tax imposed. Unlike most other Census Bureau finance statistics, they are not categorized along any functional lines.

CATEGORY **PROPERTY TAXES**

Code T01 Property Taxes

Taxes imposed on ownership of property and measured by its value.

Definition: Three types of property taxes, all having in common the use of value as a basis for the tax:

- General property taxes, relating to property as a whole, taxed at a single rate or at classified rates according to the class of property. Property refers to real property (e.g., land and structures) as well as personal property; personal property can be either tangible (e.g., automobiles and boats) or intangible (e.g., bank accounts and stocks and bonds).
- Special property taxes, levied on selected types of property (e.g., oil and gas properties, house trailers, motor vehicles, and intangibles) and subject to rates not directly related to general property tax rates.
- Taxes based on income produced by property as a measure of its value on the assessment date.

Includes: Penalties and interest on delinquent property taxes; proceeds of tax sales and tax redemptions, up to the amount of taxes due plus penalties and interest (report any excess receipts as follows: report amounts retained by the taxing government at *Miscellaneous General Revenue, NEC*, code U99, and exclude any amounts held for or returned to original property owner(s)). For governments collecting taxes as agents for another, includes any commissions, fees, or other items representing collection expenses retained from tax proceeds.

Code T01 Property Taxes - continued

Excludes:

- Discounts to taxpayers for prompt payment of their tax bill.
- Taxes or other charges on property measured by any basis other than its value, such as area, front footage, or other "special assessments" (use *Special Assessments*, code U01) as well as such measures as corporate stock, bank deposits, or "per head" taxes (see description under *License Taxes*, codes T20 – T29).
- Taxes measured by taxpayer's income from <u>intangible</u> property (report at *Income Taxes*, codes T40 and T41*)*.
- Taxes paid in protest and held by government in a suspense fund (report as property tax revenue if dispute is settled in government's favor; do <u>not</u> report as tax revenue any amounts returned to taxpayer). See Section 3.11.1.
- Taxes from utility owned by the taxing government (nonrevenues).
- Payments-in-lieu-of-taxes (if paid by another government, report at *Intergovernmental Revenue*; if paid by a private organization, use *Miscellaneous General Revenue, NEC*, code U99; and if paid by another agency or utility of the same government, exclude entirely from revenue).

CATEGORY SALES AND GROSS RECEIPTS TAXES

Taxes on goods and services, measured on the basis of the volume or value of their transfer, upon gross receipts or gross income therefrom, or as an amount per unit sold (gallon, package, etc.); and related taxes based upon use, storage, production, importation, or consumption of goods and services. Includes licenses levied at more than minor rates.

Excludes dealer discounts or "commissions" allowed to merchants for collecting taxes; taxes on the severance of natural resources, measured by value or quantity severed (report at *Severance Taxes*, code T53), except when imposed as a part of and at the same rate as general sales or gross receipts taxes.

Code T08 Federal Customs Duties

Definition: Taxes levied on the importation or exportation of goods and services.

Special Consideration:
This tax category applies only to the Federal Government. State and local governments are excluded by definition.

Code T09 General Sales and Gross Receipts Taxes

Definition: Taxes applicable with only specified exceptions (e.g., food and prescribed medicines) to sales of all types of goods and services or to all gross receipts, whether at a single rate or at classified rates; and sales use taxes.

Code T09 General Sales and Gross Receipts Taxes - continued

Includes: This includes sales or gross receipts taxes on the purchase or lease of motor vehicles, if there is no specific and separate tax law covering this activity.

Excludes: Taxes imposed distinctively on sales of or gross receipts from selected commodities, services, or businesses (report at appropriate *Selective Sales and Gross Receipts Taxes*, codes T10 - T19). If a sales tax on vehicles is authorized by a law distinctly separate from a general sales tax law, use *Other Selective Sales and Gross Receipts*, code T19.

SUB-CATEGORY SELECTIVE SALES AND GROSS RECEIPTS TAXES

Taxes imposed on the sale of particular commodities or services or on gross receipts of particular businesses separately and apart from General Sales or Gross Receipts Taxes; and licenses measured by sales or gross receipts and producing more than minor amounts of revenue.

Excludes license fees for commodity inspections at a rate per unit of commodity inspected that produces only minor revenue (report at appropriate *License Taxes*, codes T20 – T29).

Code T10 Alcoholic Beverages Sales Tax

Definition: Taxes on sale of alcoholic beverages, whether collected through government-operated liquor stores or through private outlets.

Code T11 Amusements Sales Tax

Definition: Taxes on admission tickets or admission charges and on gross receipts of all or specified types of amusement businesses.

Code T12 Insurance Premiums Sales Tax

Definition: Taxes imposed distinctively on insurance companies and measured by gross premiums or adjusted gross premiums.

Code T13 Motor Fuels Sales Tax

Definition: Taxes on gasoline, diesel oil, aviation fuel, “gasohol,” “ethanol,” and any other fuels used in motor vehicles or aircraft.

Excludes: Taxes on sale of fuels other than motor fuels – e.g., for heating, lighting, cooking, etc. (report at *Other Selective Sales Taxes*, code T19); and refunds of fuel taxes (deduct from gross tax proceeds).

Code T14 Pari-mutuels Sales Tax

Definition: Taxes measured by amounts wagered or bet on horse-racing, dog racing, jai-lai, etc., including "breakage" collected by the government.

Excludes: Race track licenses not based on amounts wagered (report at *Amusement License Taxes*, code T21); sales taxes on race track admissions (use *Amusements Sales Tax*, code T11).

Code T15 Public Utilities Sales Tax

Definition: Taxes imposed distinctively on public utilities, and measured by gross receipts, gross earnings, or units of service sold, either as a direct tax on consumers or as a percentage of gross receipts of utility.

Includes: Public utilities include passenger and freight transportation companies; telephone (land based and mobile), telegraph, cable television providers, and Internet service providers, in addition to the electric power, gas, mass transit, and water supply utilities defined separately for Census Bureau statistics on government-operated utilities.

Excludes: Payments-in-lieu-of-taxes from utilities operated by other governments (report at *Intergovernmental Revenue*); any tax or payment-in-lieu-of-taxes imposed on a government's own utility gross receipts (interfund transfer); taxes levied on such companies on any other basis (report at appropriate tax related to the type of measurement concerned).

Special Considerations:

1. Report here taxes imposed by a government on its own utility if they are billed directly to consumers on a percentage or flat rate base and are clearly visible to the consumer as a tax separate from the utility charges. Do not include taxes on the gross receipts of its own utilities.
2. The term "public utility" is applied broadly within this category and is not limited to the four public utility types used in the Census Bureau classification system to define government-operated utilities.

Code T16 Tobacco Products Sales Tax

Definition: Taxes on tobacco products and synthetic cigars and cigarettes, including related products like cigarette tubes and paper.

Code T19 Other Selective Sales and Gross Receipts Taxes

Definition: Taxes on specific commodities, businesses, or services not reported separately above (e.g., on contractors, hotel/motel, lubricating oil, fuels other than motor fuel, motor vehicles, meals, soft drinks, margarine, etc.). For state governments, includes sales or use taxes based on sales price, where the authorizing legislation is separate from the state's general sales and use tax law.

Code T19 Other Selective Sales and Gross Receipts Taxes – continued

Examples: For state governments, includes the following:
• Special taxes on car rentals, such as Florida's Motor Vehicle Car Rental Surcharge.

• State medical provider taxes, such as New Hampshire's Medicaid Assessment Program and West Virginia's Health Care Provider Tax.

• If a motor vehicle sales tax is based on the sales price of the vehicle and the authorizing legislation is separate from the general sales tax law, it is coded at T19.

CATEGORY LICENSE TAXES

Taxes exacted (either for revenue raising or for regulation) as a condition to the exercise of a business or nonbusiness privilege. Can be levied at a flat rate or by such bases as capital stock or surplus, number of business units, or capacity. Generally, includes taxes on property levied on some basis other than assessed value (e.g., on corporate stock or bank deposits). Also includes "fees" related to licensing activities as well as license taxes producing substantial revenues.

Excludes taxes measured directly by transactions, by gross or net income and receipts, or by value of property, except those with only minor rates (report at appropriate *Tax* code related to the type of measurement concerned).

Code T20 Alcoholic Beverages License

Definition: Licenses for manufacturing, importing, wholesaling, and retailing of alcoholic beverages.

Excludes: Taxes based on volume or value of transactions (report at *Alcoholic Beverage Sales Taxes*, code T10) or on assessed value of property (report at *Property Taxes*, code T01); and Federal customs duties on alcoholic beverage imports (use *Federal Customs Duties*, code T08).

Code T21 Amusements License

Definition: Licenses on amusement businesses generally and on specific types of amusement enterprises or devices (e.g., race tracks, movie theaters, athletic events, pinball and video game machines, etc.).

Excludes: "Licenses" based on value or number of admissions (report at *Amusements Sales Taxes*, code T11), on amount of wagers (report at *Pari-mutuels Sales Taxes*, code T14), or on gross or net income and receipts (use code T11).

Code T22 Corporations in General License

Definition: Franchise license taxes; organization, filing and entrance fees; taxes on property measured by amount of corporate stock, debt, or other basis besides assessed value of property; and other licenses applicable with few, specified exceptions to all corporations.

Excludes: Taxes on corporations based on value of property (use *Property Taxes*, code T01), on net income (report at *Corporation Net Income Taxes,* code T41), or on gross receipts from sales, other than at minor rates (use *General Sales and Gross Receipt Taxes*, code T09); and taxes distinctively imposed on particular kinds of businesses, such as public utilities, insurance companies, etc. (report at appropriate *Selective Sales Tax*, codes T10 – T19).

Code T23 Hunting and Fishing License

Definition: Licenses for commercial and noncommercial hunting and fishing permits.

Code: T24 Motor Vehicles License

Definition: Licenses imposed on owners or operators of motor vehicles for the right to use public highways, such as fees for title registration, license plates, vehicle inspection, vehicle mileage and weight taxes on motor carriers, highway use taxes, and off-highway fees.

Excludes: Personal property taxes on motor vehicles (use *Property Taxes*, code T01); sales or gross receipts taxes on the sale of motor vehicles (use *General Sales and Gross Receipts Taxes,* code T09); taxes on motor carriers based on assessed value of property (use code T01), gross receipts (use code T09), net income (use *Corporation Net Income Taxes*, code T41); and other taxes on the business of motor transport (use *Occupation and Business Licenses, NEC*, code T28).

Code T25 Motor Vehicle Operators License

Definition: Licenses for the privilege of driving motor vehicles, both commercial and private.

Code T27 Public Utilities License

Definition: Licenses distinctively imposed on public utilities, whether privately- or publicly-owned.

Includes: Public utilities include passenger and freight transportation companies; telephone (land based and mobile), telegraph, cable television providers, and Internet service providers, in addition to the electric power, gas, mass transit, and water supply utilities defined separately for Census Bureau statistics on government-operated utilities.

Code T27 Public Utilities License - continued

Excludes: Taxes measured by gross or net income, units of service sold, value of property (report previous items at appropriate *Tax* code related to the type of measurement concerned); and payments-in-lieu-of-taxes received from other government-owned utilities (report at *Intergovernmental Revenue*).

Code T28 Occupation and Businesses License, NEC

Definition: Licenses (including examination and inspection fees) required of persons engaged in particular professions, trades, or occupations; taxes on insurance companies based on value of their policies; such taxes on businesses not elsewhere classified; and charges or fees relating to the inspection and marketing of commodities (e.g., seed, feed, fertilizer, gasoline, oil, citrus fruit, etc.).

Code T29 Other License Taxes

Definition: Licenses not listed separately above (e.g., animal licenses including "per head" levies on livestock, health permits, marriage licenses, building and equipment permits, individual permits to purchase liquor, registration fees on aircraft and pleasure boats, impact fees paid in connection with issuance of building permits, and other nonbusiness privileges).

CATEGORY **INCOME TAXES**

Taxes levied on the gross income of individuals or on the net income of corporations and businesses (i.e., after allowable deductions).

Deduct refunds of taxes from gross collections even if they were recorded as tax revenue in a previous fiscal year.

Code T40 Individual Income Taxes

Definition: Taxes on individuals measured by net income and taxes on special types of income (e.g., interest, dividends, income from intangible property, etc.).

Includes: For local governments, includes wages, salaries, and other compensation earned by both residents and nonresidents, that are subject to tax collections by the reporting government.

Excludes: Taxes using income from intangible property as a measure of its value as of assessment date (report at *Property Taxes*, code T01); income taxes on unincorporated businesses (report at *Corporation Net Income*, code T41); payroll taxes to finance insurance trusts programs, such as Social Security taxes (report at appropriate *Social Insurance Trust Revenue* code); and city gross earnings taxes (report at *Taxes, NEC,* code T99).

Code T40 Individual Income Taxes - continued

Special Consideration:
This category includes combined individual and corporation income taxes where proceeds could not be separated in older, historical data.

Code T41 Corporation Net Income Taxes

Definitions: Taxes on corporations and unincorporated businesses (when taxed separately from individual income), measured by net income, whether on corporations in general or on specific kinds of corporations, such as financial institutions.

Excludes: Income taxes on gross income or receipts of corporations (report at *Sales and Gross Receipts Taxes*, codes T09 – T19) and combined corporation and individual income taxes not separable by type in older, historical data (report at *Individual Income Taxes*, code T40).

Examples: State governments – Michigan's VAT tax (Single Business Tax) is classified here.

Special Consideration:
Although such taxes may be called "license" or "franchise taxes," they are classified here if measured by net income.

CATEGORY **OTHER TAXES**

Code T50 Death and Gift Taxes

Definition: Taxes imposed on the transfer of property at death, in contemplation of death, or as a gift (e.g., inheritance and estate taxes).

Code T51 Documentary and Stock Transfer Taxes

Definition: Taxes on the recording, registration, and transfer of documents, such as mortgages, deeds, and securities.

Excludes: Taxes on recording or transfer of motor vehicle titles (report at *Motor Vehicle Licenses*, code T24).

Code T53 Severance Taxes

Definition: Taxes imposed distinctively on removal (severance) of natural resources (e.g., oil, gas, coal, other minerals, timber, fish, etc.) from land or water and measured by the value or quantity of products removed or sold.

GENERAL REVENUE BY TYPE: TAXES

Code T99 Taxes, NEC

Definition: Taxes not listed separately or provided for in categories above, such as taxes on land at a specified rate per acre (rather than on assessed value).

Examples: Local governments – Pennsylvania townships commonly impose a "Per Capita Tax" that is based strictly on residency within a community

Special Consideration:
For local governments, this category includes local "gross earnings" taxes, which are paid by employers' based on their total payroll (not to be confused with individual income taxes imposed on employees and reported at *Individual Income Taxes*, code T40).

GENERAL REVENUE BY TYPE: INTERGOVERNMENTAL REVENUE

The intergovernmental revenue category consists of amounts received from other governments, whether for use in performing specific activities, for general financial assistance, or as a share of tax proceeds.

This category includes the following types of transactions:

- Grants-in-aid
- Shared taxes
- Payments-in-lieu-of-taxes on public property exempt from taxation
- Contingent loans and advances (see Section 3.7.1)
- Significant and identifiable reimbursements from other governments for performing governmental functions
- Aid from other governments representing their share of the costs to finance activities administered by the receiving government

The intergovernmental revenue category can be further characterized by the types of transactions that are excluded. Two general types of financial transactions between governments are excluded from intergovernmental revenue:

1) Transactions that represent money exchanged in business-type activities. Type 1 transactions are amounts received from sale of buildings, property or commodities (report at appropriate *Miscellaneous General Revenue* code) and charges for utility services to other governments (report at appropriate *Utility Revenue* code).

2) Transactions involving the receipt of funds in an agency capacity. Type 2 transactions are the retention of taxes collected on behalf of another government which actually imposed the tax (report at appropriate *Tax* code), transactions between governments involving marketable securities or debt instruments (nonrevenues except for earnings, reported at *Interest Earnings*, code U20), and contributions received from other governments for employee retirement and other insurance trust funds (report at appropriate *Social Insurance Trust Revenue* code).

All intergovernmental revenue, regardless of its use, is classified as general revenue. Thus, the intergovernmental revenue codes for utilities that were added to the classification system effective with fiscal year 1988 data are treated as general revenue.

Intergovernmental revenue is classified two ways: (1) by the type of government from which the money is received, and (2) by its purpose or function.

GENERAL REVENUE BY TYPE: INTERGOVERNMENTAL REVENUE

CATEGORY INTERGOVERNMENTAL REVENUE FROM FEDERAL GOVERNMENT

Amounts received directly from the Federal Government. For states, this includes Federal grants and aid, payments-in-lieu-of-taxes on Federal property, reimbursements for state activities, and revenue received but later transmitted through the state to local governments. For local governments, includes only direct aid from the Federal Government. Report Federal grants "passed-through" the states as *Intergovernmental Revenue from State Governments* at the local government level.

Excludes amounts received by state governments that are distributed to individuals without discretion as to how they are disbursed (e.g., certain veterans benefits); rental or sale of buildings, property, or commodities to the Federal Government (report at appropriate *General Charges* or *Miscellaneous General Revenue* code); taxes on Federal property (report at *Property Taxes*, code T01); and proceeds from sale, liquidation, or earnings of Federal securities (nonrevenues except for earnings, reported at *Interest Earnings*, code U20).

Also excludes charges for utility services to Federal Government (report at appropriate *Utility Revenue* code); payments-in-lieu-of-taxes from independent special district housing authorities even though they may be designated locally as "Federal" agencies (report at *Intergovernmental Revenue from Local Governments*); and value of noncash assistance (e.g., gifts of food, property, land, etc.).

Code B01 Air Transportation

Definition: Federal aid for construction, operation, and support of public airports; and other distributions from the Federal Airport and Airway Trust Fund.

Code B21 Education

Definition: Federal aid for Federally-impacted areas; migrant and bilingual education; Indian education; Head Start program; Federal grants for school nutrition and milk programs (use B21 for state and *Intergovernmental Revenue From State Government*, code C21, for local and special districts); and grants and contractual amounts received by institutions of higher education for education or for research and development programs.

Excludes: Grants for agricultural experiment stations and extensions (use *Natural Resources*, code B59) and aid for library services (use *All Other*, code B89).

Code B22 Employment Security Administration

Definition: Federal aid for administration of the cooperative Federal-state unemployment compensation system, public employment offices and related services, and veterans' readjustment allowances; and determination of eligibility for Social Security disability benefits.

Code B22 Employment Security Administration – continued

Excludes: Workforce Investment Act - WIA (formerly the Job Training Partnership Act - JTPA) and Federal grants for occupational health and safety (use *All Other*, code B89, for both).

Special Consideration:
Valid for statistics on state governments and Washington, DC, only.

Code B30 General Local Government Support

Definition: Federal aid which may be applied at the discretion of the receiving government to any of its basic functions or purposes; payments-in-lieu-of-taxes; and aid received distinctively as reimbursement for Federal programs or facilities which have resulted in loss of state or local tax revenue.

Excludes: School aid in Federally-affected areas (use *Education*, code B21) and shared revenues from national forests, grazing lands, mineral leases, etc. (use *All Other*, code B89).

Special Consideration:
Effective with fiscal year 1988 data, includes amounts for Federal tax relief programs previously reported at code B28.

Code B42 Health and Hospitals

Definition: Federal aid for alcohol, drug abuse, and mental health; communicable disease control; maternal and child health; special supplemental food program (WIC); environmental health; and care of veterans in state hospitals, including construction of facilities.

Excludes: Federal aid for medical care under public assistance programs such as Medicaid even if received by a public hospital, for care in nursing homes not associated with hospitals, or for payments to vendors for medical care in public assistance cases (use *Public Welfare*, code B79); and hospital charges received on behalf of individuals under Medicare or other Federal insurance-type arrangements (report at *Public Hospital*, code A36).

Examples: Federal EPA Superfund grants.

Code B46 Highways (including roads and streets)

Definition: Federal aid distributed from the Federal Highway Trust or other funds for approved projects and for highway safety.

Excludes: Federal grants for urban mass transit (use *Public Mass Transit Systems*, code B94).

GENERAL REVENUE BY TYPE: INTERGOVERNMENTAL REVENUE

Code B50 Housing and Community Development

Definition: Federal aid for construction or operation of public housing; rent subsidy programs (e.g., “Section 8” funds); and rural, urban, and community development.

Code B59 Natural Resources

Definition: Federal aid for forests and grasslands; soil, water, and energy conservation; flood prevention and drainage; fish and wildlife management; and mine reclamation and safety.

Includes: Federal aid for agricultural experiment stations and extension services; inspection of meat, poultry, and other agricultural products; and agricultural research.

Excludes: Federal aid for parks and recreation and shared revenue from national forests, grazing lands, mineral leasing, flood control lands, etc. (use *All Other*, code B89 for both). Also excludes Federal grants for school nutrition and milk programs (use *Education*, code B21, for states and *Education*, code C21, for local and special district governments); special supplemental food program, or WIC (use *Health and Hospitals*, code B42); food stamp program (use *Public Welfare*, code B79); and value of food commodities distributed to states.

Special Consideration:
Effective 2005, this category includes transactions formerly reported under code B54, Agriculture.

Code B79 Public Welfare

Definition: Federal aid for categorical programs – Temporary Assistance for Needy Families - TANF (formerly Aid to Families with Dependent Children - AFDC); medical assistance programs (Medicaid) even if received by a public hospital; care in nursing homes not associated with hospitals; food stamp administration; child welfare services; low-income energy assistance; social and community services block grants; refugee assistance; work incentives program (WIN); and related administration.

Excludes: Federal aid for school nutrition and milk programs (use *Education*, code B21, for states and *Education*, code C21, for local and special district governments) and special supplemental food program, or WIC (use *Health and Hospitals*, code B42).

Code B80 Sewerage

Definition: Federal aid for construction, operation, and maintenance of sanitary and storm water sewer systems, and sewage disposal and treatment facilities.

Code B89 All Other

Definition: Federal aid for specified purposes other than categories shown above (e.g., economic development, libraries, civil defense and militias, disaster assistance, public broadcasting, parks and recreation, water transportation) and for multipurpose activities.

Examples:

- Workforce Investment Act (formerly the Job Training Partnership Act - JTPA).
- Shared revenue from national forests, grazing lands, mineral leasing, and flood control lands, even though such funds must be spent on specified types of activities, such as schools and roads.
- Disaster Assistance (FEMA).

SUB-CATEGORY UTILITIES

Federal aid received in support of utility projects, other than receipt of charges for utility services and commodities provided to the Federal Government, which are classified as *Utility Charges* (A91 - A94).

These codes were added to the *Classification Manual* effective with fiscal year 1988 data, as a service to users who wanted to track the fiscal activities of utilities. They represent items previously classed at other codes under this same category (i.e., *All Other*, B89 and Public Transportation, code B47). Despite their utility suffixes, however, these codes continue to be classified as general revenue.

Code B91 Water Supply Systems

Definition: Federal aid for maintenance, operation, and construction of public water supply systems, whether for distribution of water to the general public or to other public or private utilities.

Excludes: Reimbursements for sale of water to Federal Government as a regular utility customer (use *Water Supply Systems*, code A91) and grants related to water irrigation, conservation, and management even if project is designed for future water supply uses (use *Natural Resources*, code B59).

Code B92 Electric Power Systems

Definition: Federal aid for maintenance, operation, and construction of public electric power generating plants and related transmission systems.

Excludes: Reimbursements for sale of electric power to Federal Government as a regular utility customer (use *Electric Power Systems*, code A92) and grants for water development projects which have an electricity generating component (use *Natural Resources*, code B59).

Code B93 Gas Supply Systems

Definition: Federal aid for maintenance, operation, and construction of public natural gas supply and distribution systems.

Excludes: Reimbursements for sale of natural gas to Federal Government as a regular utility customer (use *Gas Supply Systems,* code A93).

Code B94 Public Mass Transit Systems

Definition: Federal aid for maintenance, operation, and construction of public mass transit systems (buses, subways, commuter railroads, urban mass transit, and the like).

Excludes: Grants for other modes of transportation, such as airports (use *Airports*, code B01) and highways (use *Highways*, code B46).

Special Consideration:
Prior to fiscal year 1988, these revenues were classified at Public Transportation, code B47.

GENERAL REVENUE BY TYPE: INTERGOVERNMENTAL REVENUE

CATEGORY INTERGOVERNMENTAL REVENUE FROM STATE GOVERNMENTS

Amounts received directly from the state government, including Federal aid passed through the state government and state aid channeled through intermediate local governments (e.g., counties), which have no discretion as to its distribution.

Includes state grants-in-aid, regardless of basis of distribution; local share of state-collected taxes (see Section 4.3.1.1); payments-in-lieu-of-taxes on state property; and reimbursement for services performed for state government (e.g., care of state prisoners in local jails, construction or maintenance of state highway facilities, etc.).

Excludes proceeds from sale of buildings, property, or commodities (report at appropriate *Miscellaneous General Revenue* code); charges for utility services to state government (report at appropriate *Utility Revenue* code); taxes on state government facilities (use *Property Taxes*, code T01); contributions from state for locally-administered employee retirement systems (use *Contributions From Other Governments*, code X05); and retained share or collection “fee” on state-imposed taxes collected by local governments (report at appropriate *Tax* code).

Also excludes proceeds from the sale, liquidation, or earnings of state government securities held by local governments (nonrevenues except for earnings, reported at *Interest Earnings*, code U20); receipt of locally-imposed taxes collected by the state acting as an agent for local governments (report at appropriate *Tax* code); agency receipts (i.e., amounts received for transmittal to other governments or individuals without discretion as to how they are disbursed by the agent government); and value of noncash assistance (e.g., gifts of property, commodities, etc.).

This category applies only to Federal and local government finances. The Census Bureau’s classification system does not recognize state-to-state payments as intergovernmental transactions.

Code C21 Education

Definition: State aid for support of local schools; redistribution of Federal aid for education; handicapped, special, and vocational education and rehabilitation; student transportation; equalization aid; school health; local community colleges; adult education; school buildings; and property tax relief related strictly to school funding.

Excludes: State grants for libraries (use *All Other*, code C89); state expenditures on behalf of local schools for textbooks, buses, school buildings, etc. (report as state direct expenditure for education); and value of donated food commodities (nonrevenue).

Examples: Federal pass-through programs – Chapter 1, Individuals with Disabilities Education Act, Eisenhower Math and Science, Safe and Drug Free Schools, Chapter 2 Block Grants, and Child Nutrition Act funds.

GENERAL REVENUE BY TYPE: INTERGOVERNMENTAL REVENUE

Code C30 General Local Government Support

Definition: State aid which may be applied at the discretion of the receiving government to any of its basic functions or purposes; local share of state-imposed taxes or of profits from alcoholic beverage monopolies; per capita aid; state revenue sharing; payments-in-lieu-of-taxes; and aid received distinctively as reimbursement for state-mandated tax relief programs or for facilities which have resulted in loss of local tax revenue (e.g., property tax relief, homestead exemptions, etc.).

Excludes: Amounts received as reimbursement for services performed by local governments for the state government (report at appropriate *Intergovernmental Revenue from State Government* code); shared state taxes or property tax relief related strictly to school funding (use *Education*, code C21); shared state taxes with specific uses, such as gasoline taxes designated for highway purposes (report those with single uses at appropriate *Intergovernmental Revenue from State Government* code and those with multipurpose uses at *All Other,* code C89); and share of state taxes expressly for local employee retirement systems even if they are first passed through the parent government (report at *Contributions from Other Governments,* code X05).

Special Consideration:
Effective with fiscal 1988 data, includes amounts for state tax relief programs previously reported at code C28.

Code C42 Health and Hospitals

Definition: State aid for local health programs; maternal and child health; alcohol, drug abuse, and mental health; environmental health; superfunds; nursing aid; hospital financing (including construction); and hospitalization of patients in local government hospitals.

Excludes: State aid for medical care under public assistance programs such as Medicaid even if received by a public hospital, for care in nursing homes not associated with hospitals, and for payments to vendors for medical care in public assistance cases (use *Public Welfare*, code C79); and hospital charges received on behalf of individuals under state insurance-type arrangements (use *Public Hospitals*, code A36).

Code C46 Highways

Definition: State aid for construction, improvement, or maintenance of streets, highways, bridges, tunnels, etc.; distribution of state fuel taxes; and aid for debt service on local highway debt.

Excludes: State grants for urban mass transit (use *Public Mass Transit Systems*, code C94).

GENERAL REVENUE BY TYPE: INTERGOVERNMENTAL REVENUE

Code C50 Housing and Community Development

Definition: State aid for construction or operation of public housing; rent subsidy programs; repair and renovation of existing houses; and rural, urban, and community development.

Code C79 Public Welfare

Definition: State aid for public welfare purposes; medical care and related administration under public assistance programs (including Medicaid) even if received by a public hospital; care in nursing homes not associated with hospitals; Federal categorical assistance; noncategorical assistance (e.g., home relief, emergency assistance); and administration of local welfare programs. For Federal Government (intergovernmental revenue from the states), this category includes revenue from state governments for increased benefits under the Supplemental Security Income program (SSI).

Examples: Federal pass-through categorical assistance programs, such as Temporary Assistance to Needy Families **-** TANF (formerly Aid to Families with Dependent Children, AFDC).

Code C80 Sewerage

Definition: State aid for construction, operation, and maintenance of sanitary and storm water sewer systems, and sewage disposal and treatment facilities.

Code C89 All Other

Definition: State aid for specific purposes other than categories shown above and for multipurpose activities, such as public works in general.

Excludes: State aid (other than shared taxes reported at *General Local Government Support*, code C30) expressly for local employee retirement systems even if they are first passed through the parent government (report at *Employee Retirement Contributions from Other Governments*, code X05).

SUB-CATEGORY   UTILITIES

State aid received in support of utility projects other than receipt of charges for utility services and commodities provided to the state government.

Excludes contributions from parent government (interfund transfers) and contributions from other participating public utilities for the construction of a joint utility project or for debt service on such a project IF the monies are treated as part of the recipient utility's basic rate structure (report at appropriate *Utility Revenue* code).

GENERAL REVENUE BY TYPE: INTERGOVERNMENTAL REVENUE

These codes were added to the Classification Manual, effective with fiscal year 1988 data, as a service to users who wanted to track the fiscal activities of utilities. They represent items previously classed at other codes under this same category (i.e., *All Other*, code C89, and Public Transportation, code C47). Despite their utility suffixes, however, these codes continue to be classified as general revenue.

Code C91 Water Supply Systems

Definition: State aid for maintenance, operation, and construction of public water supply systems, water purification plants, etc.

Excludes: Reimbursements for sale of water to state government as a regular utility customer (use *Water Supply Systems*, code A91) and water conservation unrelated to utility operations (use *All Other*, code C89).

Code C92 Electric Power Systems

Definition: State aid for maintenance, operation, and construction of public electric power generating plants and related transmission systems.

Excludes: Reimbursements for sale of electric power to state government as a regular utility customer (use *Electric Power Systems*, code A92).

Code C93 Gas Supply Systems

Definition: State aid for maintenance, operation, and construction of public natural gas supply and distribution systems.

Excludes: Reimbursements for sale of natural gas to state government as a regular utility customer (use *Gas Supply Systems*, code A93).

Code C94 Public Mass Transit Systems

Includes: State aid for maintenance, operation, and construction of public mass transit systems (buses, subways, commuter railroads, urban mass transit, and the like).

Excludes: Grants for other modes of transportation, such as airports (use *All Other*, code C89) or highways and ferries (use *Highways*, code C46); and for pupil transportation (use *Education*, code C21).

Special Consideration:
Prior to fiscal year 1988, these revenues were classified at Public Transportation, code C47.

GENERAL REVENUE BY TYPE: INTERGOVERNMENTAL REVENUE

CATEGORY INTERGOVERNMENTAL REVENUE FROM LOCAL GOVERNMENTS

Amounts received from local governments for use in performing specific functions, for general financial assistance, or as share of tax proceeds.

For state governments, includes local government share of state-administered programs requiring local financial participation; reimbursements from locals for services provided to them by the state, such as auditing local accounts; monies from localities to pay debt service on state debt issued for the benefit of local governments; and repayments of contingent loans and advances previously extended to local governments.

For state governments, excludes monies received from local governments and held in an agency capacity on their behalf (e.g., to repay interest and principal on local debt); receipt of state-imposed taxes or other state revenues collected by local governments acting as agents for state (report at appropriate *Tax* code); taxes on local government facilities (report at appropriate *Tax* code); proceeds from the sale, liquidation, or earnings of local government securities held by the state (nonrevenues except for earnings, reported at *Interest Earnings*, code U20); proceeds from sale of buildings, property, or commodities (report at appropriate *Miscellaneous General Revenue*, code U99); charges for utility services to local governments (report at appropriate *Utility Revenue* code); and contributions from local governments for employee retirement or other insurance trust systems (report at appropriate *Social Insurance Trust Revenue* code).

For local governments, includes financial support from other local governments for activities administered by recipient locality, including its dependent agencies; state aid channeled through other local governments which have some discretion as to its distribution; reimbursements for services provided to other local governments (other than for utility services); and payments-in-lieu-of-taxes on other local governments' property.

For local governments, excludes state grants or shared taxes to be transmitted through government with no discretion as to their distribution (report at *Intergovernmental Revenue From State Governments* for final recipient local government); receipt of taxes or other charges collected by another local government acting as the receiving government's agent (report at appropriate *Tax* code); proceeds from sale, liquidation, or earnings of local government securities held by other localities (nonrevenues except for earnings, reported at *Interest Earnings*, code U20); taxes on facilities of other local governments (use *Property Tax*, code T01); proceeds from sale of buildings, property, or commodities (report at appropriate *Miscellaneous General Revenue* code); charges for utility services to other local governments (report at appropriate *Utility Revenue* code); and payments-in-lieu-of-taxes from private organizations like private colleges (use *Miscellaneous General Revenue, NEC*, code U99).

Code D11 Interschool System Revenue

Definition: Local aid received by a school district from other school districts.

Special Considerations:

1. This category applies only to the finances of individual independent school districts. It is not used for other types of governments, but is necessary for accurate measurement of totals for individual units.

Code D11 Interschool System Revenue - continued

Special Considerations: (continued)

2. Similarly, this category is not included in any Census Bureau statistics that present totals for more than a single government, such as state area or national totals (tabulated or estimated). The exclusion is conceptual, reflecting the fact that this is an intergovernmental code.

Code D21 Education

Definition: For state governments, rental on school buildings constructed by state school building authorities and leased to local governments; and local share of school building construction projects.

Excludes: Local aid received by an independent school district from other school systems (use *Interschool System Revenue*, code D11) and contributions from parent government of a dependent school system (nonrevenues).

Code D30 General Local Government Support

Definition: Local aid that may be applied at the discretion of the receiving government to any of its basic functions or purposes, including payments-in-lieu-of-taxes.

Code D42 Health and Hospitals

Definition: Local aid for hospitalization of patients in other government hospitals and local share of state or other local government-administered programs.

Excludes: Care in nursing homes not associated with hospitals (use *Public Welfare*, code D79) and payments from parent government of a dependent hospital (nonrevenues).

Code D46 Highways

Definition: Local aid as reimbursement for signals and traffic lights maintenance and for share of costs of joint highway projects.

Code D50 Housing and Community Development

Definition: Local aid as share of joint housing projects, and intergovernmental payments for community development projects.

Special Consideration:

This code is valid for state governments in fiscal year 2006.

Code D79 Public Welfare

Definition: Local aid as share of state-administered welfare programs.

Code D80 Sewerage

Definition: Local aid as contributions or share of joint sewer projects; and charges for sewer services provided to local governments.

Code D89 All Other

Definition: Local aid received for specific purposes other than categories shown above and for multipurpose activities, such as public works in general and share of costs for courts, central computer services, etc.

Excludes: Aid received for public utility purposes, use *Utility*, codes D91 – D94.

SUB-CATEGORY UTILITIES

Local aid received in support of utility projects other than receipt of charges for utility services and commodities provided to local governments.

Excludes contributions from parent government (interfund transfers) and contributions from other participating public utilities for the construction of a joint utility project or for debt service on such a project IF the monies are treated as part of the recipient utility's basic rate structure (report at appropriate *Utility Revenue* code).

These codes were added to the Classification Manual, effective with fiscal year 1988 data, as a service to users who wanted to track the fiscal activities of utilities. They represent items previously classed at other codes under this same category (i.e., *All Other*, code D89, and Public Transportation, code D47). Despite their utility suffixes, however, these codes continue to be classified as general revenue.

Code D91 Water Supply Systems

Definition: Local aid for maintenance, operation, and construction of public water supply systems, water purification plants, etc.

Excludes: Reimbursements for sale of water to local governments as regular utility customers (use *Water Supply Systems*, code A91).

Code D92 Electric Power Systems

Definition: Local aid for maintenance, operation, and construction of public electric power generating plants and related transmission systems.

Excludes: Reimbursements for sale of electric power to local governments as regular utility customers (use *Electric Power Systems*, code A92).

GENERAL REVENUE BY TYPE: INTERGOVERNMENTAL REVENUE

Code D93 Gas Supply Systems

Definition: Local aid for maintenance, operation, and construction of public natural gas supply and distribution systems.

Excludes: Reimbursements for sale of natural gas to local governments as regular utility customers (use *Gas Supply Systems*, code A93).

Code D94 Public Mass Transit Systems

Definition: Local aid for maintenance, operation, and construction of public mass transit systems (buses, subways, commuter railroads, urban mass transit, and the like).

Excludes: Grants for other modes of transportation, such as airports (use *All Other*, code D89) and highways (use *Highways*, code D46).

Special Consideration:
Prior to fiscal year 1988, these revenues were classified at Public Transportation, code D47.

GENERAL REVENUE BY TYPE: CURRENT CHARGES

The concept of current charges covers amounts received from the public for performance of specific services which benefit the person charged and from the sale of commodities or services other than utilities and liquor stores. Includes fees, maintenance assessments, and other reimbursements for current services; rents and sales derived from commodities or services furnished incident to the performance of particular functions; gross income of commercial enterprises; and the like. Charges are distinguished from license taxes, which are privileges granted by a government or fees collected to finance regulatory activities.

The category of current charges excludes special assessments (use *Special Assessments*, code U01), amounts received from other governments in return for services provided (report at appropriate *Intergovernmental Revenue* code), and interdepartmental charges and intragovernmental transfers within the same government (see Section 3.9).

Report current charges on a gross basis without deducting the cost of providing related services.

Code A01 Air Transportation (Airports)

Definition: Hangar rentals, landing fees, terminal and concession rents, sale of aircraft fuel and oil, parking fees at airport lots, and other charges for use of airport facilities or for services associated with their use.

Code A03 Miscellaneous Commercial Activities

Definition: Charges of publicly-owned commercial enterprises not classified elsewhere, such as markets, cement plants, cemeteries, etc.

Excludes: Charge revenues from utilities (use *Utility Revenue*, codes A91 – A94); liquor stores (use *Liquor Store Revenue*, code A90); sewerage facilities (use *Sewerage*, code A80); and landfills, garbage collection, and other solid waste management activities (use *Solid Waste Management*, code A81).

Code A06 Federal National Defense and International Relations

Definition: Proceeds from the sale of defense materials and stocks to non-Federal sources.

Code A09 Elementary-Secondary Education School Lunch

Definition: Gross receipts from sale of milk and school lunches.

Code A10 Elementary-Secondary Education School Tuition

Definition: Amounts from pupils and parents for tuition and transportation.

Excludes: Tuition received from other governments (report at appropriate *Education* code, B21, C21, or D21).

GENERAL REVENUE BY TYPE: CURRENT CHARGES

Code A12 Other Elementary-Secondary Education

Definition: Revenues from athletic contests, sale or rental of textbooks, student activity funds, and the like.

Code A14 Federal Postal Service

Definition: Sale of postage and commemorative stamps, postal insurance fees, fees for certified and registered mail, and other charges by the U.S. Postal Service.

Code A16 Higher Education Auxiliary Enterprises

Includes: Gross receipts from sales and charges by dormitories, cafeterias, athletic contests, lunchrooms, student activities, bookstores, and similar commercial activities financed wholly or largely through these charges.

Excludes: Receipts from sale of products incidental to operation of a higher education program (use *Other Higher Education Charges*, code A18).

Code A18 Other Higher Education Charges

Includes: Tuition, laboratory fees, and other charges of higher education facilities other than auxiliary enterprises.

Excludes: Charges of hospitals for general public operated by universities (use *Public Hospitals*, code A36).

Code A21 Federal and State Education Charges, NEC

Definition: Fees of state schools for blind, deaf, and handicapped; and tuition and fees of state vocational-technical schools which award certificates equal to less than two years of college.

Excludes: Library charges and fees (use *All Other*, code A89); payments from local governments for state school building authorities (use *Education*, code D21); and charges of university-operated hospitals and of handicapped institutions primarily for physical rehabilitation or care (use *Public Hospitals*, code A36).

Special Considerations:
This code applies to Federal and state governments. Allocate all local government education charges among *Elementary-Secondary School Lunch*, code A09, *Elementary-Secondary School Tuition*, code A10, *Other Elementary-Secondary Education*, code A12, and *Other Higher Education*, code A18.

GENERAL REVENUE BY TYPE: CURRENT CHARGES

Code A36 Public Hospitals

Definition: Charges from patients, private insurance companies, and public insurance programs (such as Medicare) of public hospitals and of institutions for care and treatment of the handicapped; and receipts of hospital canteens, cafeterias, gift shops, etc.

Excludes: Revenues received from other governments for care of patients (report at health and hospitals *Health and Hospitals*, codes B42, C42, or D42); fees of state schools for blind, deaf, or handicapped (use *Federal and State Education Charges, NEC,* code A21); and health clinics operating on outpatient basis only (use *All Other,* code A89).

Special Consideration:
As of fiscal year 1974 data, intergovernmental aid for hospital medical care under public assistance programs (such as Medicaid) has been reported at *Public Welfare,* code B79 or C79, rather than code A36; all other intergovernmental aid for hospital medical care should be reported at *Health and Hospitals,* code B42, C42, or D42. Other than insurance-type programs cited above, do not report payments from other governments at this code.

Code A44 Regular Highways

Definition: Reimbursements for street construction and repairs; fees for street cuts and special traffic signs; and maintenance assessments for street lighting, snow plowing, and other highway or street services unrelated to toll facilities.

Excludes: Excludes special assessments levied on homeowners who benefit from specific street or sidewalk improvements and impact fees to build roads in new developments (use *Special Assessments,* code U01).

Code A45 Toll Highways

Definition: Fees from turnpikes, toll roads, bridges, ferries, and tunnels; rents and other revenue from concessions (service stations, restaurants, etc.); and other charges for use of toll facilities.

Includes: Lease of toll roads.

Code A50 Housing and Community Development Charges

Definition: Gross rentals, tenant charges, and other revenue from operation of public housing projects; and fees for housing mortgage insurance (e.g., FHA-insured loans).

Excludes: Receipts from sale of property (use *Sale of Property*, code U11) and payments-in-lieu-of-taxes from housing projects operated by other governments, whether independent housing authorities (special districts) or dependent housing agencies of general purpose governments (report at *Housing and Community Development*, codes B50, C50, or D50).

GENERAL REVENUE BY TYPE: CURRENT CHARGES

Code A56 Federal and State Forestry

Definition: Sale of timber, nursery stock, and other products from Federal and state forests; and reimbursements for forest fire suppression services.

Code A59 Other Natural Resources

Definition: Sale of minerals and other natural products (other than forestry products) from public lands. For Federal Government also includes fees and rentals from public grazing and grasslands, fees for uranium enrichment, and sale of electric power (e.g., by Bonneville Power Administration and Tennessee Valley Authority).

Includes: Receipts from sale of products from agricultural experiment station farms; revenue from agricultural fairs and shows; agricultural laboratory charges (such as milk testing fees); Federal Government sale of surplus crops and commodities; and other related agricultural charges.

Excludes: Royalties for privilege of developing public land (use *Royalties*, code U41); and taxes on the removal (severance) of natural resources measured by value or quantity (use *Severance Taxes*, code T53). Revenue from the sale of forestry products (report at *Federal and State Forestry*, code A56). Also excludes fees related to regulation or inspection activities (report at *Occupation and Businesses License Taxes, NEC*, code T28); and special assessments for capital outlay improvements benefiting specific agricultural land owners (use *Special Assessments*, code U01).

Code A60 Parking Facilities

Definition: Revenue from on-street and off-street parking meters and charges and rentals from government-owned parking lots or public garages.

Special Consideration:
Effective 2005, this code is valid for state governments.

Code A61 Parks and Recreation

Definition: Gross revenue of facilities operated by a government (swimming pools, recreational marinas and piers, golf courses, skating rinks, museums, zoos, etc.); auxiliary facilities in public recreation areas (camping areas, refreshment stands, gift shops, etc.); lease or use fees from stadiums, auditoriums, and community and convention centers; and rentals from concessions at such facilities.

GENERAL REVENUE BY TYPE: CURRENT CHARGES

Code A80 Sewerage

Definition: Charges for sewage collection and disposal, including sewer connection fees.

Excludes: Impact fees to build sewers in new developments (use *Special Assessments*, code U01).

Special Considerations:
For combined water-sewer system, include segregable amounts derived from sewerage activities.

Code A81 Solid Waste Management

Definition: Fees for garbage collection and disposal; operation of landfills; sale of recyclable materials; cleanup of hazardous wastes; and sale of electricity, gas, steam, or other by-products of waste resource recovery or cogeneration facilities.

Excludes: Fees related to regulation or inspection activities (report at *Occupation and Businesses License Taxes, NEC*, code T28).

Special Consideration:
Effective with 1988 data, this category was expanded to include the Federal and state governments, formerly classed at *All Other*, code A89, and renamed from "Other Sanitation Charges."

Code A87 Sea and Inland Port Facilities

Definition: Canal tolls, rents from leases, concession rents, and other charges for use of commercial or industrial water transport and port terminal facilities and related services.

Excludes: Fees and rents related to water facilities provided for recreational purposes, such as marinas, public docks, etc. (use *Parks and Recreation*, code A61); and toll ferries (use *Toll Highways*, code A45).

Special Consideration:
Effective 2005, the title of this category was changed from Water Transport and Terminals.

Code A89 All Other General Current Charges

Definition: Charges not covered by any of the above categories, such as those derived from court and recording fees, police, fire, correction, defense, public welfare, public nursing homes, public libraries, and health activities. For Federal Government also includes fees on utilities for nuclear waste disposal and assessments on banks and savings and loans institutions for insurance on savers' deposits.

Excludes: Reimbursements and special assessments for capital outlay improvements which benefit specific property owners (use *Special Assessments*, code U01) and sale of used vehicles, surplus equipment, scrap materials, etc., which do not relate to any specific function or service (report at *Sale of Property*, code U11, or *Miscellaneous General Revenue*, code U99).

GENERAL REVENUE BY TYPE: MISCELLANEOUS GENERAL REVENUE

Miscellaneous General Revenue is comprised of general revenue that does not fall into one of the above *Tax, Intergovernmental Revenue,* or *Current Charges* categories.

Code U01 Special Assessments

Definition: Compulsory contributions and reimbursements from owners of property who benefit from specific public improvements; and impact fees to fund extension of water, sewer, roads, and other infrastructure facilities in new developments. These contributions and reimbursements are designed to defray all or part of the cost of such improvements, either directly or through payment of principal and interest on debt issued to finance them. Generally, special assessments are apportioned according to assumed benefits to the property affected by the improvements. They cover not only general improvements – such as street paving, sidewalks, highway construction, sewer lines, drainage and irrigation projects – but also utility improvements, such as water lines.

Excludes: Proceeds from sale of special assessment bonds (report at appropriate *Debt* code); maintenance assessments for recurrent services such as street lighting, leaf removal, and weed control (report at appropriate *Current Charges* code); and reimbursements or contributions for capital outlays incurred under voluntary agreements, such as cooperative state government construction projects (use *Miscellaneous General Revenue, NEC,* code U99) and joint state-local or local-local construction projects (report at appropriate *Intergovernmental Revenue* code).

Special Considerations:

1. Special assessments and property taxes are distinguished from each other by these features: taxes are levied on all property owners on the basis of the property's assessed value and are generally used to support a wide range of public activities. In contrast, special assessments are levied on just certain property owners who have benefited from specific public improvements and are used to fund or repay their costs.

2. Special assessments and current charges are distinguished from each other by these features: special assessments are generally for one-time capital improvements, such as paving a street, installing street lights or sidewalks, etc. Current charges, on the other hand, are for ongoing, recurrent services such as street cleaning, street lighting, leaf removal, etc., even though they may be labeled as "maintenance assessments" or even just "assessments."

3. This code does not apply to Federal Government financial statistics.

Code U11 Sale of Property

Definition: Amounts received from sale of real property, buildings, improvements to them, land easements, rights-of-way, and other capital assets (buses, automobiles, etc.), including proceeds from sale of operating and nonoperating property of utilities.

Includes: Proceeds from sale of land and improvements associated with housing and community development programs, proceeds from sale of toll roads. Includes sale of property to other governments.

Code U11 Sale of Property - continued

Excludes: Excludes proceeds from sale of securities (nonrevenues); sale of property to meet delinquent taxes or tax liens (report at *Property Tax*, code T01); and sale of commodities, unclaimed or confiscated property, and minor personal property (use *Miscellaneous General Revenue, NEC*, U99).

Special Consideration:
Prior to the 2005 fiscal year, the Census Bureau classification system included two categories for sale of property. Code U10 was Housing and Community Development and represented revenue from the sale of property associated with housing and community development programs only. Code U11 was All Other and represented revenue from the sale of all other types of government owned land or property. These two categories were merged, effective with fiscal year 2005.

Code U20 Interest Earnings

Definition: Amounts from interest on all interest-bearing deposits and accounts; accrued interest on investment securities sold; interest on funds held for construction; and interest related to public debt for private purposes.

Excludes: Interest on deposits and investments of employee retirement and other insurance trust funds (report at appropriate *Social Insurance Trust Revenue* code); dividends from investments (use *State Government Other Dividends*, code U21, for state governments and *Miscellaneous General Revenue, NEC*, code U99 for local governments); accrued interest on bonds issued by the government (deduct from *Interest on Debt*, codes I89 or I91 - I94); recorded profits on sale of investments (use code U99 except for profits from sale of investments by Social Insurance Trust Systems); and accrued interest on the purchase of investments (deduct from *Interest Earnings* reported for those investments).

Special Consideration:
Effective with fiscal year 1988 data, the treatment of interest revenue related to public debt for private purposes was changed. Now, report interest revenue in an amount equal to the interest payments (*Interest on Debt*, code I89) on this type of debt. Previously, this treatment was limited to mortgage revenue bonds and was classified at *Rents*, code U40.

Code U21 State Government – Other Dividends

Definition: Revenue from the distribution of earnings by a corporation for which the government has an ownership claim, based on the purchase of investments (such as stocks) in that corporation.

Excludes: Interest earnings (report at U20, *Interest earnings*).

Code U21 State Government – Other Dividends - continued

Special Considerations:

1. This classification category was introduced effective with fiscal year 2005. Previously, dividends were reported at *Miscellaneous General Revenue NEC,* code U99.
2. This code applies only to state governments.
3. This code applies to general sector dividends only. For Public Employee Retirement Systems, dividends are reported at code Z72.

Code U30 Fines and Forfeits

Definition: Revenue from penalties imposed for violations of law; civil penalties (e.g., for violating court orders); court fees if levied upon conviction of a crime or violation; court-ordered restitutions to crime victims where government actually collects the monies; and forfeits of deposits held for performance guarantees or against loss or damage (such as forfeited bail and collateral).

Excludes: Penalties relating to tax delinquency (report at appropriate *Tax* code); library fines (report at *All Other General Current Charges*, code A89); and sale of confiscated property (use *Miscellaneous General Revenue, NEC,* code U99).

Code U40 Rents

Definition: Revenue from allowing temporary possession of government-owned buildings, land, or other fixed properties, such as from grazing fees, timberland leases, rental of unused land or property (including nonoperating property of a government utility), and revenue from leases (or lease bonus payments) of land relating to natural resource exploration and production.

Excludes: Rental revenue from functionally classified facilities, such as rent of airport hangars, or where the government provides services in return, such as public housing rents (report at appropriate *Current Charge* code); and rental revenue from other governments (report at appropriate *Intergovernmental Revenue* code).

Special Considerations:

1. Prior to FY 1988 data, this code included interest on mortgage revenue bonds equal to the amount reported for interest payments on this type of debt. (See note under *Interest Earnings,* code U20.)
2. Rents reported here are revenues for which there are little or no related services provided by the government.
3. This code does not apply to Federal Government financial statistics, nor to special district or school district governments.

Code U41 Royalties

Definition: Compensation or portion of proceeds received by a state or local government for granting the privilege of using or developing property or operating under a right, primarily those related to natural resources, such as oil, gas, and mineral rights.

Excludes: Sale of timber and other forestry products from state forests (report at *Federal and State Forestry Charges*, code A56).

Special Considerations:

1. Prior to 2005, this code applied only to state governments. Local government royalties were reported at *Rents*, code U40.
2. Rents and royalties are distinguished from each other by these features: rents are compensation for the temporary possession of a building, land, or some other fixed or immovable object, usually with specific payments over a specified period of time. Royalties are compensation for the use or development of land and property or for operating under a right (such as a mineral right) and are usually based on a share of the proceeds the payer earns from these.
3. This code does not apply to Federal Government financial statistics.

Code U50 Donations from Private Sources

Definition: Gifts of cash or securities from private individuals or corporations.

Excludes: Noncash gifts and donations, such as food, property, buildings, land, commodities, etc. (nonrevenues).

Special Considerations:

1. Report the entire receipt of nonexpendable bequests even if only the interest income from it can be spent at the government's discretion.
2. This code does not apply to Federal Government financial statistics.

Code U95 Net Lottery Revenue

Definition: Proceeds from the operation of government-sponsored lotteries after deducting the cost of prizes.

Special Considerations:

1. In terms of lottery exhibit codes, this item is computed by subtracting *Prizes Awarded*, exhibit code Z52 from *Total Ticket Sales*, exhibit code Z51. Thus, it includes amounts used for administration that are reported at *Administrative Expenses,* exhibit code Z53.

2. This code does not apply to Federal Government financial statistics.

Code U95 Net Lottery Revenue - continued

Special Considerations: (continued)

3. Note that the list of exhibit codes below includes those related to receipts and to expenses. The latter are included here because there is no equivalent lottery expenditure code, since U95 is a net amount that already takes into account the lottery costs and expenditures.

Exhibit Code Statistics Related to Lottery Revenue
(Summarized – see Chapter 10 for detail)

Code and Description		Formula, If Calculated
Code Z51	Total Ticket Sales	
Code Z52	Prizes Awarded	
Code Z53	Administrative Expenses	
Code Z54	Proceeds Available	+Z51 - (Z52 + Z53)

Special Considerations:

1. Codes Z51 and Z54 pertains to "receipts," while Z52 and Z53 pertain to "expenses."
2. These exhibit codes are valid for state governments and Washington, DC only.
3. See Chapter 10 for full definitions and additional explanations of these lottery exhibit codes.

Code U99 Miscellaneous General Revenue, NEC

Definition: Recovery of losses charged off in a prior fiscal year; insurance adjustments; payments-in-lieu-of-taxes from private sources; voluntary contributions or reimbursements to defray construction costs of capital improvement projects such as in joint state construction projects; premiums on bonds issued; revenues from sponsorship agreements; unemployment compensation contributions from employers for servicing debt issued to cover deficits in these trust funds; recoveries of expenditures made in a prior fiscal year; receipts from escheats and other unclaimed monies; recorded profits from sale of investments; and any other receipts within the definition of general revenue but not classifiable as a *Tax, Intergovernmental Revenue,* or *Current Charge*.

Includes: Tobacco Lawsuit Settlement revenue or proceeds of bond sales made by a private corporation, IF the government has entered into a Tobacco Securitization Agreement with the private corporation (see also *Tobacco Lawsuit Settlements - Receipts,* exhibit code CGR); dividends for local governments.

Excludes: Proceeds from borrowing (report at appropriate *Debt* code); sale of investment securities (nonrevenue); interfund transfers or loans (nonrevenue); transfers from other agencies of same government (nonrevenue); and discounts on bonds issued (noncash transaction).

Code U99 Miscellaneous General Revenue, NEC - continued

Special Consideration:
Prior to fiscal year 2005, this category included dividends earned by a state government (except for dividends earned by a public employee retirement system). Effective 2005, dividends of state governments are reported at *State Government – Other Dividends*, code U21.

Exhibit Codes Related to Miscellaneous General Revenue

<u>Code and Description</u>

CGR Tobacco Lawsuit Settlement – Receipts

Definition: Total amount received during the fiscal year, from proceeds of master settlement agreement between the state governments' and the major tobacco manufacturers, pursuant to Federal judicial proceedings.

Includes: Total amount received, regardless of whether such receipts were expended during the fiscal year.

Special Considerations:

1. All amounts reported here also are reported at *Miscellaneous General Revenue, NEC,* code U99, except annual payments for securitization through a private corporation.
2. This exhibit code applies only to state governments and to Washington, DC, by definition. The Census Bureau does not track the total amount of lawsuit proceeds expended at the local government level, but rather reports such expenditure by function only.
3. Excludes the lump sum proceeds from tobacco securitization IF handled via a private corporation. Continue to report at code U99.
4. The related expenditure exhibit code for these receipts is *Tobacco Lawsuit Settlement – Expenditure of Proceeds*, code CGE. See Chapter 5 for details.

LIQUOR STORES REVENUE BY TYPE: REGULAR AND EXHIBIT

CATEGORY **LIQUOR STORES**

Liquor stores revenue comprises only receipts from sales and associated services or products of liquor stores owned and operated by state and local governments. It excludes any application of general revenue for liquor store operations as well as receipts from licenses or other liquor taxes collected by liquor stores or systems (including general sales tax collections). All taxes collected through liquor store operations are classified as *Tax Revenue*.

Code A90 Liquor Stores Revenue - Current Charges

Definition: Gross receipts (less discounts and any sales taxes included in receipts) from sale of alcoholic beverages and related operations revenue of publicly-owned and operated liquor stores, Alcoholic Beverage Control (ABC) stores, and their variously-named equivalents.

Excludes: Sales and license taxes on alcoholic beverages collected through these stores (report at *Alcoholic Beverages Sales Tax*, code T10, or at *Alcoholic Beverages License Tax*, code T20); contributions from parent government (nonrevenues); receipt of state liquor store profits distributed to local governments (if unrestricted in use, report at *General Local Government Support*, code C30).

Examples: State governments – West Virginia Alcohol Beverage Control Administration, which controls the wholesale distribution of alcoholic beverages in that state. Pennsylvania Liquor Control Board, which controls the sale of alcoholic beverages in that state.

Special Consideration:
The exhibit codes and derived statistics shown below apply only to state government liquor stores. There are no such codes reported for local governments.

Exhibit Codes and Derived Statistics Related to Liquor Stores Revenue
(Summarized – see Chapter 10 for detail)

Code and Description		Formula, If Calculated
Z41	Net Sale of Goods	
Z44	Other Income	
Z48	Receipts from Sales Taxes, Licenses, and Permits	
….	Gross Profit on Sales	+ Z41 - Z42
….	Net Operating Revenue	+ Z41 - (Z42 + Z43)
….	Net Income	+ Z41 - (Z42 + Z43) + Z44 - Z45

UTILITY REVENUE BY TYPE: REGULAR AND EXHIBIT

CATEGORY **UTILITY REVENUE**

Gross receipts from sale of utility commodities or services to the public or other governments by publicly-owned and controlled utilities.

Includes receipts from direct sales of commodities and services; rentals from operating property; customers' forfeitures and penalties; and charges for installing and servicing connections and meters. Excludes identifiable amounts for commodities or services furnished to the parent government, its agencies, or other utilities of the same government (intragovernmental transfers); revenue from investments or other capital transactions (report *Interest Earnings*, at code U20 and recorded profits on their sale at *Miscellaneous General Revenue, NEC*, code U99); and lease rentals or other earnings from nonoperating utility property (use *Rents*, code U40).

Also excludes special assessments for utility improvements, including water impact fees (report at *Special Assessments*, code U01); contributions from parent government (another intragovernmental transfer); financial aid from other governments not representing sale of utility good or services (report at appropriate *Intergovernmental Revenue* code); taxes imposed on sale of utility commodities and services (report at *Public Utilities Tax*, code T15); and contributions from utility employees for retirement system administered by same government (report at *Employee Contributions*, code X01 or X02).

Code A91 Water Supply Systems

Definition: Revenue from operations of public water supply systems, such as sale of water to residential, industrial, and commercial customers (including bulk water for resale by other private or public water utilities); connection and "tap" fees; sprinkler fees; meter inspection fees; late payment penalties; and other operations revenues.

Excludes: Water impact fees (use *Special Assessments,* code U01), contributions-in-aid-of-construction (use code U01), and other special assessments (use code U01); sale of water to parent government or its agencies, including fire hydrants (nonrevenues); and rent or other income from nonoperating property (use *Rent*, code U40).

Special Consideration:
For combined water-sewer systems include here segregable amounts related to water supply and report sewer portion at *Sewerage*, code A80.

Code A92 Electric Power Systems

Definition: Revenue from operations of public electric power supply systems, such as sale of electricity to residential, commercial, and industrial customers (including electricity for resale by other private or public electric utilities); and other operations revenues.

Excludes: Sale of electricity to parent government or its agencies, including street lights and traffic signals (nonrevenues); rent or other income from nonoperating property (use *Rents*, code U40); and sale of electricity produced as a by-product by resource recovery facilities (report at *Solid Waste Management Charges*, code A81).

Code A92 Electric Power Systems - continued

Special Consideration:

Contributions from other governmental electric utilities for construction of joint utility projects or for debt service on them may be classified as either *Intergovernmental Revenue* or *Utility Revenue* depending on the nature of the contributions. If they are treated as part of the utility's basic rate structure, then report them as a utility revenue, *Electric Power Systems*, code A92. Otherwise, report them as *Electric Power Systems*, codes B92, C92, or D92.

Code A93 Gas Supply Systems

Definition: Revenue from operations of public gas supply systems, such as sale of natural gas to residential, commercial, and industrial customers (including natural gas for resale by other private or public gas supply utilities); connection fees; and other operations revenues.

Excludes: Sale of natural gas to parent government or its agencies (nonrevenues); rent or other income from nonoperating property (use *Rents*, code U40); and sale of natural gas produced as a by-product by resource recovery facilities (use *Solid Waste Management*, code A81).

Code A94 Public Mass Transit Systems

Includes: Revenue from operations of public mass transportation systems (rapid transit, subway, bus, street railway, and commuter rail services), such as fares, charter fees, advertising income, and other operations revenues.

Excludes: Toll ferries, turnpikes, toll roads and bridges, and other fees on personal highway transportation (report at *Toll Highways*, code A45) and subsidies from other governments to support either operations or capital projects (report at *Public Mass Transit Systems*, codes B94, C94, or D94).

SOCIAL INSURANCE TRUST REVENUE BY TYPE: REGULAR AND EXHIBIT

Revenue of social insurance trust systems includes amounts derived from contributions, assessments, premiums, or payroll taxes required of employers, employees, and others to finance compulsory or voluntary social insurance programs operated by the public sector; and any earnings on assets held or invested by such funds. In the case of public employee retirement systems, revenue also includes unrealized gains (or losses, which are deducted from revenue), based on the change in value of their investment holdings annually. (The methodology for measuring unrealized gains or losses is described more completely in Section 8.4.1.1)

Chapters 8 and 9 contain separate discussions of insurance trust systems, including a formal definition of a social insurance trust and the full definitions of the various exhibit codes for these activities.

Some of the contributions allocated to these categories are considered to be taxes (e.g., unemployment compensation payroll taxes, Social Security taxes) by many economists. However, for Census Bureau purposes these are treated as insurance trust revenues, not tax revenues.

Excluded from social insurance trust revenue are general tax proceeds, donations, and other revenue except that enumerated below even if such amounts are levied specifically for insurance trust purposes (report at appropriate *General Revenue* code); grants and shared taxes for support of insurance trusts other than employee retirement systems, whether received directly or through the parent government (report at appropriate *Intergovernmental Revenue* code); borrowing of funds (non-revenues); transfers or contributions from parent government which administers the social insurance system (either as employer contributions or for general financial support); rentals from real property leased to other agencies of the parent government (latter two are intragovernmental transfers).

These categories also exclude interest earnings on Federal securities held by the three Federal-only insurance trusts enumerated below, since these are intragovernmental transfers. In the case of state and local government insurance trusts, however, interest earnings on their own or other governments' securities held for investment are included because of the difficulty in identifying such transactions.

Moreover, not all revenues received by an insurance trust fund are classified as insurance trust revenues for Census Bureau purposes. Such receipts as grants-in-aid, charges for miscellaneous services, fines and penalties, and other miscellaneous revenues are treated as general revenues.

Statistics for social insurance trusts are comprised of regular and exhibit codes. Both types of statistics are used to measure the full set of financial transactions (revenues for this chapter) undertaken by these systems. However, only the "regular" finance codes are used for Census Bureau reports on the overall finances of a government (e.g., to derive total revenue). The exhibit codes represent financial receipts not included with revenues in the regular finances (e.g., transfers or contributions from parent government), but which are needed if users want to analyze the insurance trust independently. While both regular and exhibit statistics are included in this chapter, the latter are covered only via reference. See Chapters 8 and 9.

SOCIAL INSURANCE TRUST REVENUE BY TYPE: REGULAR AND EXHIBIT

CATEGORY **FEDERAL-ONLY SOCIAL INSURANCE TRUST SYSTEMS**

Contributions for social insurance programs administered by the Federal Government. These systems are described in more detail in Chapters 8 and 9. Note that these Federal-only systems exclude "earnings on investments" from revenue, since the assets of these trust funds are invested solely in Federal securities (thereby making them intragovernmental transfers).

Code X03 Contributions From Federal Government Employees

Definition: Amounts contributed by, or withheld from, salaries and wages of employees, and voluntary employee contributions, to provide for retirement or survivors' benefits. Includes reimbursements for disability payments, interest on employee loans, and interest on buyback contributions.

Includes: Employee contributions under the Federal supplemental retirement portion of the Federal Employee Retirement System (FERS).

Excludes: Voluntary contributions made by Federal employees to the Thrift Savings Plan portion of the FERS.

Code Y20 Federal Social Security and Medicare

Definition: Contributions (employee, employer, and self-employed payroll taxes) to provide for benefits under the Federal Old-Age, Survivors, and Health and Disability Insurance Programs, commonly referred to as Social Security and Medicare.

Code Y31 Federal Veterans' Life Insurance

Definition: Insurance premiums from veterans for purchase of annuities in life insurance programs administered by the U.S. Department of Veterans Affairs (formerly, U.S. Veterans Administration).

Excludes: Appropriations from general and special revenue funds (intragovernmental transfers).

Code Y41 Federal Railroad Retirement

Definition: Contributions (employee and employer) to provide for benefits under the Federal Railroad Retirement and Disability System.

Excludes: Contributions from Social Security trusts, tax subsidies, and any other Federal receipts representing interfund transfers; and contributions for Federal railroad unemployment compensation (use *Unemployment Compensation - Contributions*, code Y01).

SOCIAL INSURANCE TRUST REVENUE: BY TYPE

Exhibit Code Statistics Related to Federal-Only Social Insurance Trust Revenues
(Summarized – see Chapters 8 and 9 for detail)

<u>Code and Description</u>

Code X99	Interest on U.S. Securities Held By Federal Retirement Systems
Code Y22	Interest on U.S. Securities Held By Federal Social Security and Medicare Accounts
Code Y32	Interest on U.S. Securities Held By Federal Veterans' Life Insurance Funds
Code Y42	Interest on U.S. Securities Held By Railroad Retirement Fund Accounts
Code Y99	Interest on U.S. Securities Held By Federal Government Unemployment Compensation Accounts

CATEGORY PUBLIC EMPLOYEE RETIREMENT SYSTEMS

Contributions from employees and other governments (as employers or as grants-in-aid and shared taxes); and interest earnings on investments of retirement systems administered by governments for public employees.

These data are collected by a separate mail canvass survey of state and local government-administered public employee retirement systems.

See Chapter 8 for a full discussion of exhibit codes and calculated statistics for employee retirement systems.

SUB-CATEGORY: Employee Contributions

Amounts contributed or withheld from salaries and wages of employees, and voluntary employee contributions, to provide for retirement or survivors' benefits. Includes reimbursements for disability payments, interest on employee loans, and interest on buyback contributions.

Also includes contributions from general employees as well as those of public colleges and other institutions or dependent agencies.

Code X01 Contributions From Local Government Employees

Code X02 Contributions From State Government Employees

Special Consideration:
This code applies to state governments only. No locally-administered employee retirement systems cover state government employees.

SOCIAL INSURANCE TRUST REVENUE: BY TYPE

SUB-CATEGORY: Employer (Government) Contributions

Code X05 Contributions from Other Governments

Definition: For local governments, amounts received either from the state or from other local governments as employer contributions for their own employees. Contributions from state governments to local retirement systems includes not only employer contributions but also state aid (e.g., contributions on behalf of local teachers) and shared taxes (e.g., portion of taxes imposed on insurance companies), even if such amounts are received through the parent government. For state governments, includes amounts received from local governments as employer contributions for their own employees.

Excludes: Contributions from parent government that administers the employee retirement system, including its share as an employer (use exhibit codes *From Parent Local Government*, code X04, or *State Contributions To Own System, Total*, code X06 – see Chapter 8 for details) and repayment of loans made to members (nonrevenue).

SUB-CATEGORY: Other Revenue (Other Receipts)

Code X08 Total Earnings on Investments

Definition: Interest earnings on investment securities, deposits, and other interest-bearing accounts, including accrued interest on investment securities sold; dividends; recorded profits on investment transactions (minus any recorded losses); rentals; and other earnings on investments. Includes unrealized gains (and losses), as reported for accounting purposes.

Excludes: Rental of property owned by a retirement system and leased to its parent government, and amortization of discounts (nonrevenues).

Special Considerations:

1. This is a calculated statistic only. It is the sum of the following five exhibit codes (see Chapter 8 for additional definitions).
   + Z71 Interest Earnings
   + Z72 Dividend Earnings
   + Z73 Other Investment Earnings
   + Z96 Gains on Investments, (Realized and Unrealized)
   - Z91 Losses on Investments (Realized and Unrealized)

2. For Retirement Survey purposes, X08 includes another exhibit code in the calculation as well, *Rentals from State Government*, code Z98. However, such receipts reflect an intragovernmental transaction and thus cannot be included as revenue for a government. See Chapter 8.

Code X08 Total Earnings on Investments - continued

Special Considerations: (continued)

3. Previous versions of the *Classification Manual* separated earnings on investments between interest income (code X08) and other types of investment earnings (X09). Since this distinction was not made on the survey schedules themselves, these two codes were consolidated effective with fiscal year 1990.

Exhibit Codes Related to Public Employee Retirement Systems Receipts (Summarized – see Chapter 8 for detail)

Code and Description

Code X04	From Parent Local Government
Code Z71	Interest Earnings
Code Z72	Dividend Earnings
Code Z73	Other Investment Earnings
Code Z91	Losses on Investments (Realized and Unrealized)
Code Z95	Other Receipts, NEC
Code Z96	Gains on Investments, (Realized and Unrealized)
Code Z98	Rentals from State Government
Code X06	State Contributions To Own System, Total
Code V87	State Contributions to Own System, on Behalf of Local Employees
Code Z99	State Contributions to Own System, on Behalf of State Employees

SOCIAL INSURANCE TRUST REVENUE BY TYPE: REGULAR AND EXHIBIT

CATEGORY **UNEMPLOYMENT COMPENSATION SYSTEMS**

Contributions from employees and governments (as employers) and interest earnings on deposits in the U.S. Treasury for the cooperative Federal-state unemployment compensation insurance system and for Federal railroad unemployment compensation. This category applies only to the Federal Government, state governments, and the District of Columbia. For a description of how this system operates, see Chapter 9.

Note that these data are obtained largely from the Employment and Training Administration of the U.S. Department of Labor.

Code Y01 Contributions

Definition: Payroll levies (or "taxes") collected from private and public employers (and in some cases employees) to provide for unemployment compensation benefits.

Excludes: Borrowing of funds (nonrevenues) and contributions (including special payroll levies) to pay debt service on loans issued to cover fund deficits (report at *Miscellaneous General Revenue, NEC*, code U99).

Code Y02 Interest Credited by U.S. Government

Definition: Interests earned on deposits held in the Unemployment Trust Fund of the U.S. Treasury and credited to individual state governments and to the District of Columbia.

Code Y04 Federal Advances and Contributions

Definition: Net amounts credited to the accounts of the individual state governments and District of Columbia during periods where contributions and interest are insufficient to pay benefits due. Report repayment of advances as <u>deductions</u> to this code, not as unemployment compensation expenditures.

Exhibit Codes Related to Unemployment Compensation System Receipts (Summarized – see Chapter 9 for detail)

<u>Code and Description</u>

Code Y99 Interest on U.S. Securities Held by Federal portion of the Unemployment Compensation Trust Funds.

SOCIAL INSURANCE TRUST REVENUE BY TYPE: REGULAR AND EXHIBIT

CATEGORY **WORKERS' COMPENSATION SYSTEMS**

Contributions and interest earnings of state government-operated insurance trust funds to provide accident, disability, and death benefits for private and public employees. This category applies solely to state governments.

These data are collected largely by a separate mail canvass survey of state workers' compensation systems. See Chapter 9 for a detailed discussion of exhibit codes for workers' compensation systems.

Code Y11 Contributions

Definition: Premiums, assessments, payroll "taxes" or other contributions collected from employers and employees to provide for workers' compensation benefits, including contributions from local governments as employers on behalf of their own employees. Also includes amounts received from employees of either state or local governments.

Excludes: Contributions from state government administering insurance trust system (use *Own Contributions from Administering Government*, code Y10); policy dividends or return of excess premiums (report as a deduction from revenues); penalties and fines (use *Fines and Forfeits*, code U30); charges for such services as copying documents (report at *All Other General Charges*, code A89); recovery of benefits and other miscellaneous revenues (use *Miscellaneous General Revenue, NEC*, code U99); and Federal grants (report at appropriate *Intergovernmental Revenue from the Federal Government* code).

Code Y12 Earnings on Investments

Definition: Interest earnings on investment securities, deposits, and other interest-bearing accounts, including accrued interest on investment securities sold; dividends; recorded profits on investment transactions (minus any recorded losses); rentals; and other earnings on investments.

Special Considerations:

1. Report losses on investments at expenditure exhibit *Administrative Expenses*, code Y15 (see Chapter 5).
2. Previous manuals separated earnings on investments between interest income (code Y12) and other types of investment earnings (code Y13). Since this distinction was not made on the survey schedules themselves, these two codes were consolidated effective with fiscal year 1990.

Exhibit Codes Related to Workers' Compensation Systems Revenue (Summarized – see Chapter 9 for detail)

<u>Code and Description</u>

Code Y10 Own Contribution from Administering Government

SOCIAL INSURANCE TRUST REVENUE BY TYPE: REGULAR AND EXHIBIT

CATEGORY **OTHER STATE SOCIAL INSURANCE TRUST SYSTEMS**

Contributions and earnings on investments for state-administered social insurance systems for health benefits, disability benefits, and other purposes. This category applies solely to state governments.

These data are collected largely by a separate mail canvass survey of state miscellaneous insurance trust systems. See Chapter 9 for additional detail on exhibit codes for other Federal and state social insurance trust systems.

Code Y51 Contributions

Definition: Premiums, assessments, or contributions collected from members to provide for benefits, including contributions from local governments as employers on behalf of their own employees. Also includes amounts received from employees of either state or local governments.

Excludes: Contributions from state government administering insurance trust system (use *Contributions to Own System*, code Y50).

Code Y52 Earnings on Investments

Definition: Interest earnings on investment securities, deposits, and other interest-bearing accounts, including accrued interest on investment securities sold; dividends; recorded profits on investment transactions (minus any recorded losses); rentals; and other earnings on investments.

Special Consideration:

1. Report losses on investments at exhibit *Other Payments*, code Y54 (see Chapter 5).
2. Previous manuals separated earnings on investments between interest income (code Y52) and other types of investment earnings (code Y55). Since this distinction was not made on the survey schedules themselves, these two codes were consolidated effective with fiscal year 1990.

Exhibit Codes Related to Other State Social Insurance Trust Systems Revenue (Summarized – see Chapter 9 for detail)

<u>Code and Description</u>

Code Y50 Contributions from Administering Government

4.10 Tables

These tables show how the revenue codes used in the Census Bureau's government finance statistical program apply to the three levels and all types of governments. The tables are shown here and repeated in Appendix 2. The revenue codes are split into the three tables for ease of reference. Table 4.1 covers the general government sector, Table 4.2 the utilities and liquor stores sectors, and Table 4.3 the social insurance trust sector (and the sub-sectors by type of social insurance trust system).

Table 4.1
Revenue Code Validity by Level and Type of Government: General Revenue – page 1 of 3

Revenue Type	Level and Type of Government						
**Taxes**	**Federal**	**State**	**County**	**Municipal**	**Township**	**Special Districts**	**School Districts**
Regular statistics:							
T01	X	Valid	Valid	Valid	Valid	Valid	Valid
T08	Valid	X	X	X	X	X	X
T09	X	Valid	Valid	Valid	Valid	Valid	Valid
T10	Valid	Valid	Valid	Valid	Valid	Valid	Valid
T11	X	Valid	Valid	Valid	Valid	Valid	Valid
T12	X	Valid	Valid	Valid	Valid	Valid	Valid
T13	Valid	Valid	Valid	Valid	Valid	Valid	Valid
T14	X	Valid	Valid	Valid	Valid	Valid	Valid
T15	Valid	Valid	Valid	Valid	Valid	Valid	Valid
T16	Valid	Valid	Valid	Valid	Valid	Valid	Valid
T19	Valid	Valid	Valid	Valid	Valid	Valid	Valid
T20	X	Valid	Valid	Valid	Valid	Valid	Valid
T21	X	Valid	Valid	Valid	Valid	Valid	Valid
T22	X	Valid	Valid	Valid	Valid	Valid	Valid
T23	X	Valid	Valid	Valid	Valid	Valid	Valid
T24	X	Valid	Valid	Valid	Valid	Valid	Valid
T25	X	Valid	Valid	Valid	Valid	Valid	Valid
T27	X	Valid	Valid	Valid	Valid	Valid	Valid
T28	X	Valid	Valid	Valid	Valid	Valid	Valid
T29	X	Valid	Valid	Valid	Valid	Valid	Valid
T40	Valid	Valid	Valid	Valid	Valid	Valid	Valid
T41	Valid	Valid	Valid	Valid	Valid	Valid	Valid
T50	Valid	Valid	Valid	Valid	Valid	Valid	Valid
T51	X	Valid	Valid	Valid	Valid	Valid	Valid
T53	X	Valid	Valid	Valid	Valid	Valid	Valid
T99	Valid	Valid	Valid	Valid	Valid	Valid	Valid
Exhibit statistics: None							
**Intergovernmental From Federal**	**Federal**	**State**	**County**	**Municipal**	**Township**	**Special Districts**	**School Districts**
Regular statistics:							
B01	X	Valid	Valid	Valid	Valid	Valid	X
B21	X	Valid	Valid	Valid	Valid	Valid	Valid
B22[1]	X	Valid	X	X[1]	X	X	X
B30	X	Valid	Valid	Valid	Valid	X	X
B42	X	Valid	Valid	Valid	Valid	Valid	X
B46	X	Valid	Valid	Valid	Valid	Valid	X
B50	X	Valid	Valid	Valid	Valid	Valid	X
B59	X	Valid	Valid	Valid	Valid	Valid	X
B79	X	Valid	Valid	Valid	Valid	Valid	X
B80	X	Valid	Valid	Valid	Valid	Valid	X
B89	X	Valid	Valid	Valid	Valid	Valid	X
B91	X	Valid	Valid	Valid	Valid	Valid	X
B92	X	Valid	Valid	Valid	Valid	Valid	X
B93	X	Valid	Valid	Valid	Valid	Valid	X
B94	X	Valid	Valid	Valid	Valid	Valid	X
Exhibit statistics: None							

Notes:
[1]B22 is a valid code at the local government level for Washington DC, only.

Table 4.1
Revenue Code Validity by Level and Type of Government: General Revenue
page 2 of 3

Revenue Type	Level and Type of Government						
**Intergovernmental From State**	**Federal**	**State**	**County**	**Municipal**	**Township**	**Special District**	**School District**
Regular statistics:							
C21[2]	X	X	Valid	Valid[2]	Valid	Valid	Valid
C30[2]	X	X	Valid	Valid[2]	Valid	X	X
C42[2]	X	X	Valid	Valid[2]	Valid	Valid	X
C46[2]	X	X	Valid	Valid[2]	Valid	Valid	X
C50[2]	X	X	Valid	Valid[2]	Valid	Valid	X
C79[2]	Valid	X	Valid	Valid[2]	Valid	Valid	X
C80[2]	X	X	Valid	Valid[2]	Valid	Valid	X
C89[2]	Valid	X	Valid	Valid[2]	Valid	Valid	X
C91[2]	X	X	Valid	Valid[2]	Valid	Valid	X
C92[2]	X	X	Valid	Valid[2]	Valid	Valid	X
C93[2]	X	X	Valid	Valid[2]	Valid	Valid	X
C94[2]	X	X	Valid	Valid[2]	Valid	Valid	X
Exhibit statistics: None							
**Intergovernmental From Local**	**Federal**	**State**	**County**	**Municipal**	**Township**	**Special District**	**School District**
Regular statistics:							
D11[3]	X	X	X	X	X	X	Valid[3]
D21	X	Valid	Valid	Valid	Valid	Valid	Valid
D30	X	Valid	Valid	Valid	Valid	X	X
D42	X	Valid	Valid	Valid	Valid	Valid	X
D46	X	Valid	Valid	Valid	Valid	Valid	X
D50[4]	X	Valid[4]	Valid	Valid	Valid	Valid	X
D79	X	Valid	Valid	Valid	Valid	Valid	X
D80	X	Valid	Valid	Valid	Valid	Valid	X
D89	X	Valid	Valid	Valid	Valid	Valid	X
D91	X	Valid	Valid	Valid	Valid	Valid	X
D92	X	Valid	Valid	Valid	Valid	Valid	X
D93	X	Valid	Valid	Valid	Valid	Valid	X
D94	X	Valid	Valid	Valid	Valid	Valid	X
Exhibit statistics: None							

Notes:
[2]Although valid for municipal governments, this code is not valid for Washington, DC, by definition.
[3]This code is valid only for school districts. It is not used in aggregate statistics by level or type of government.
[4]This code becomes valid for state government effective with 2006 statistics.

Table 4.1
Revenue Code Validity by Level and Type of Government: General Revenue
page 3 of 3

**Revenue Type**	**Level and Type of Government**						
**Current Charges**	**Federal**	**State**	**County**	**Municipal**	**Township**	**Special District**	**School District**
Regular statistics:							
A01	Valid	Valid	Valid	Valid	Valid	Valid	X
A03	X	Valid	Valid	Valid	Valid	Valid	X
A06	Valid	X	X	X	X	X	X
A09	X	Valid	Valid	Valid	Valid	X	Valid
A10	X	Valid	Valid	Valid	Valid	X	Valid
A12	X	Valid	Valid	Valid	Valid	X	Valid
A14	Valid	X	X	X	X	X	X
A16	X	Valid	Valid	Valid	Valid	X	Valid
A18	X	Valid	Valid	Valid	Valid	Valid	Valid
A21	Valid	Valid	X	X	X	X	X
A36	Valid	Valid	Valid	Valid	Valid	Valid	Valid
A44	X	Valid	Valid	Valid	Valid	Valid	X
A45	X	Valid	Valid	Valid	Valid	Valid	X
A50	Valid	Valid	Valid	Valid	Valid	Valid	X
A56	Valid	Valid	X	X	X	X	X
A59	Valid	Valid	Valid	Valid	Valid	Valid	X
A60	X	Valid	Valid	Valid	Valid	Valid	X
A61	Valid	Valid	Valid	Valid	Valid	Valid	X
A80	X	Valid	Valid	Valid	Valid	Valid	X
A81	Valid	Valid	Valid	Valid	Valid	Valid	X
A87	Valid	Valid	Valid	Valid	Valid	Valid	X
A89	Valid	Valid	Valid	Valid	Valid	Valid	X
Exhibit statistics: None							
**Miscellaneous**	**Federal**	**State**	**County**	**Municipal**	**Township**	**Special District**	**School District**
Regular statistics:							
U01	X	Valid	Valid	Valid	Valid	Valid	Valid
U11	Valid	Valid	Valid	Valid	Valid	Valid	Valid
U20	Valid	Valid	Valid	Valid	Valid	Valid	Valid
U21	X	Valid	X	X	X	X	X
U30	Valid	Valid	Valid	Valid	Valid	Valid	Valid
U40	X	Valid	Valid	Valid	Valid	X	X
U41	X	Valid	Valid	Valid	Valid	Valid	Valid
U50	X	Valid	Valid	Valid	Valid	Valid	Valid
U95	X	Valid	Valid	Valid	Valid	Valid	Valid
U99	Valid	Valid	Valid	Valid	Valid	Valid	Valid
Exhibit statistics:							
CGR[5]	X	Valid	X	X[5]	X	X	X
Z51[5]	X	Valid	X	X[5]	X	X	X
Z54[5]	X	Valid	X	X[5]	X	X	X

Key:
Valid Statistics can be reported for these governments.
X Statistics cannot be reported for these governments.

Notes:

[5]Valid code at the local government level for Washington, DC, only.

Table 4.2 Revenue Code Validity by Level and Type of Government: Liquor Stores and Utilities Sectors							
**Revenue Category**	**Level and Type of Government**						
**Liquor Stores Sector**	**Federal**	**State**	**County**	**Municipal**	**Township**	**Special District**	**School District**
Regular statistics:							
A90	X	Valid	Valid	Valid	Valid	X	X
Exhibit statistics:							
Z41	X	Valid	X	X	X	X	X
Z44	X	Valid	X	X	X	X	X
Z48	X	Valid	X	X	X	X	X
**Utilities Sector**	**Federal**	**State**	**County**	**Municipal**	**Township**	**Special District**	**School District**
Regular statistics:							
A91	X	Valid	Valid	Valid	Valid	Valid	X
A92	X	Valid	Valid	Valid	Valid	Valid	X
A93	X	Valid	Valid	Valid	Valid	Valid	X
A94	X	Valid	Valid	Valid	Valid	Valid	X
Exhibit statistics: None							

Key:

Valid Statistics can be reported for these governments.

X Statistics cannot be reported for these governments.

**Table 4.3 Revenue Code Validity by Level and Type of Government: Social Insurance Trust Sector –** page 1 of 2							
**Revenue Category**	**Level and Type of Government**						
**Federal Only Categories**	**Federal**	**State**	**County**	**Municipal**	**Township**	**Special District**	**School District**
Regular Statistics:							
Y20	Valid	X	X	X	X	X	X
Y31	Valid	X	X	X	X	X	X
Y41	Valid	X	X	X	X	X	X
Exhibit Statistics:							
Y22	Valid	X	X	X	X	X	X
Y32	Valid	X	X	X	X	X	X
Y42	Valid	X	X	X	X	X	X
**Employee Retirement**	**Federal**	**State**	**County**	**Municipal**	**Township**	**Special District**	**School District**
Regular statistics:							
X01	X	Valid	Valid	Valid	Valid	Valid	Valid
X02	X	Valid	X	X	X	X	X
X03	Valid	X	X	X	X	X	X
X05	X	Valid	Valid	Valid	Valid	Valid	Valid
X08	X	Valid	Valid	Valid	Valid	Valid	Valid
Exhibit statistics:							
V87	X	Valid	X	X	X	X	X
X04	X	X	Valid	Valid	Valid	Valid	Valid
X06	X	Valid	X	X	X	X	X
X99	Valid	X	X	X	X	X	X
Z71	X	Valid	Valid	Valid	Valid	Valid	Valid
Z72	X	Valid	Valid	Valid	Valid	Valid	Valid
Z73	X	Valid	Valid	Valid	Valid	Valid	Valid
Z91	X	Valid	Valid	Valid	Valid	Valid	Valid
Z95	X	Valid	Valid	Valid	Valid	Valid	Valid
Z96	X	Valid	Valid	Valid	Valid	Valid	Valid
Z98	X	Valid	X	X	X	X	X
Z99	X	Valid	X	X	X	X	X

Table 4.3 Revenue Code Validity by Level and Type of Government: **Social Insurance Trust Sector –** page 2 of 2							
**Revenue Category**	**Level and Type of Government**						
**Unemployment Compensation**	**Federal**	**State**	**County**	**Municipal**	**Township**	**Special District**	**School District**
Regular statistics:							
Y01[1]	Valid	Valid	X	X[1]	X	X	X
Y02[1]	X	Valid	X	X[1]	X	X	X
Y04[1]	X	Valid	X	X[1]	X	X	X
Exhibit statistics:							
Y99	Valid	X	X	X	X	X	X
**Workers' Compensation**	**Federal**	**State**	**County**	**Municipal**	**Township**	**Special District**	**School District**
Regular statistics:							
Y11	X	Valid	X	X	X	X	X
Y12	X	Valid	X	X	X	X	X
Exhibit statistics:							
Y10	X	Valid	X	X	X	X	X
**Other State Social Insurance Trusts**	**Federal**	**State**	**County**	**Municipal**	**Township**	**Special District**	**School District**
Regular statistics:							
Y51[1]	X	Valid	X	X[1]	X	X	X
Y52[1]	X	Valid	X	X[1]	X	X	X
Exhibit statistics:							
Y50[1]	X	Valid	X	X[1]	X	X	X

Key:

Valid Statistics can be reported for these governments.

X Statistics cannot be reported for these governments.

Notes:

[1]These codes are valid for Washington, DC, only at the local level.

Chapter 5. Expenditure

Introduction

Expenditure statistics are classified by function, and by character and object, within the Census Bureau's classification system for government finance. This cross classification provides a detailed picture of the services provided by governments – what is the service, how much money is being spent, and how is the service being performed or provided.

5.1 Expenditure Definition

Expenditure includes all amounts of money paid out by a government during its fiscal year – net of recoveries and other correcting transactions – other than for retirement of debt, purchase of investment securities, extension of loans, and agency or private trust transactions. Under this definition, expenditure relates to external payments of a government and excludes amounts transferred to funds or agencies of the same government (other than payments to intragovernmental service funds – see Section 3.9.2).

Expenditure includes payments from all sources of funds, including not only current revenues but also proceeds from borrowing and prior year fund balances. Note, however, that the statistics do not relate expenditure to the source of funding.

Expenditure includes amounts spent by all agencies, boards, commissions, or other organizations categorized as dependent on the government concerned. Stated in terms of the accounting procedures from which these data originate, expenditure covers outlays of all accounting funds of a government other than intragovernmental service (revolving), agency, and private trust funds.

Expenditures of business-type activities of governments (utilities and other commercial or auxiliary enterprises) are reported on a gross basis. That is, related revenues are not deducted from their expenditures to derive net expenditure amount.

See Table 5.1 for detailed explanations of expenditure transactions.

5.1.1 Financial Transactions Excluded From the Definition of Expenditure

The following types of outlays are excluded from expenditure, as a result of the above definition:

- Loans or other extensions of credit, except contingent loans and advances to other governments which are reported as intergovernmental expenditure (see Section 3.7.1).

- Refunds of revenues collected during the same fiscal year, which are deducted from gross receipts of revenue item involved (see Section 3.10.4).

- Erroneous payments and other outlays that are recovered during the same fiscal year, which are deducted from the expenditures that originally included them (see Section 3.10.4).

- Purchase of securities for investment purposes. Recorded loss on the sale of investments, however, is treated as an expenditure.

- Payments for the retirement of debt principal (long- or short-term), which are reported with

debt statistics (see Chapter 6). Interest on debt, however, is reported as an expenditure.

- Transfers to other agencies or funds of the same government, such as intragovernmental service funds and utilities operated by the same government.

- Agency or private trust transactions, i.e., where the government is acting on behalf of others (see Section 3.10).

- Noncash transactions, such as provision of perquisites (like the use of government-owned housing), technical services, commodities, property, noncash gifts or bequests, and other "payments in kind." The cost of items distributed in-kind, however, are treated as purchase of goods and services (i.e., as a current operations expenditure).

- Depreciation of capital assets.

5.1.2 Refunds and Correcting Transactions

Expenditure data are adjusted for refunds and other correcting transactions, including those originally made in prior fiscal years. Adjustment transactions are not reported separately in Census statistics; instead, they reduce or increase amounts reported as revenue or expenditure. The exact treatment of such transactions depends on when the refund or correction occurred – i.e., whether it was in the same fiscal year or a prior fiscal year. The exact treatment of such transactions was detailed in Section 3.10.4.

5.2 Cross Classification of Expenditure – Function, Character, and Object Definitions

All expenditure is classified by character, object, and function in the Census Bureau system. This results in a three character, alpha numeric code, typically of the format "Ann," where "A" represents the object code of the expenditure transaction and "nn" indicates the numeric functional code. Most expenditure code combinations, but not all, apply to all three levels of government, and to all types of local government. However, definitions restrict some expenditure codes by level or type of government. The applicability and restriction of expenditure codes is noted in the description code pages in Section 5.6.3 and in the valid code tables of this *Classification Manual*, especially Table 5.2. Also, Appendix Table 2.2 in Appendix 2 provides a different perspective of how expenditures are cross-classified.

Expenditure functions and character and object codes are defined and explained below.

5.2.1 Expenditure Function Definition

The expenditure "function" refers to the purpose for which a government spends money and, by extension, the service being provided by the government. Currently, there are 64 functions in the classification system for "regular" statistics. These are broken down as follows among the four sectors of government:

General government – 50
Utilities sector – 4
Liquor stores sector – 1
Social insurance trust sector – 9

In addition, there are sixteen exhibit code expenditure functions. The expenditure description pages of

Section 5.6.3 contain definitions and explanations of the functions.

5.2.2 Expenditure Character and Object Definitions

The character and object classifications reflect the time period benefited by the expenditure and the type of expenditure payment involved. The two classifications always are linked together in Census Bureau statistics on government finance. Expenditure character defines the nature of the expenditure payment and refers to the broad group of related expenditure types used for tabulation purposes. Expenditure object, on the other hand, refers to the specific type of financial transaction. Object classification categories are used in data collection, compilation, and processing. There are no specific classification codes for the character of expenditure, only categories. There are expenditure object codes, however, and they are critical in the Census Bureau's classification system.

Character categories (and their object codes) are as follows:

- Total expenditure
  - Direct expenditure
    - Current operations (E)
    - Interest on debt (I)
    - Assistance and Subsidies (J)
    - Insurance benefits and repayments (X,Y)
    - Capital outlay (F, G, K)
  - Intergovernmental expenditure (L, M, Q, S)

Object codes define the specific type of expenditure within the character groupings. Currently, the Census Bureau classification system contains twelve object codes to define the type of expenditure and one additional object code (Z) for some expenditure exhibit statistics. There have been some changes to the object codes used by Census Bureau over the years.

Character groupings and the specific object codes in the classification system are summarized in Chart 5.A, below. Summary definitions are found in Sections 5.2.2.1 and 5.2.2.2, while Table 5.1 at the close of this chapter contains more detailed definitions.

Chart 5.A Character and Object Categories	
**Object Code**	**Character and Object Category**
**Direct Expenditure Categories:**	
E	Current Operations
I	Interest on Debt
J[1]	Assistance and Subsidies
	**Capital Outlay:**
F	Construction
G[1]	Capital Outlay Other than Construction<br>OR<br>Purchase of Land and Existing Structures
K[1]	Purchase of Equipment
	**Social Insurance Trust Expenditure:**
X	Public Employee Retirement Systems
Y	All Other Social Insurance Trust Systems
**Intergovernmental Expenditure Categories:**	
L	To State Governments
M	To Local Governments
Q	To Independent School Districts
S	To the Federal Government
**Exhibit Expenditure Categories** :[2]	
Z	Used for various Public Employee Retirement System, Lottery, and Liquor Stores exhibit categories, plus total salaries and wages of a government.

[1]See the paragraphs following the table for a more detailed discussion of “G”, “J”, and “K.”

[2]One additional exhibit code exists that is an exception to the overall classification pattern. This is Tobacco Lawsuit Settlement – Expenditure of Proceeds, code CGE. See description pages in Section 5.6.3 for details.

The “G” and “K” object codes require additional explanation. Object code “K” is used for “Purchase of Equipment” expenditure statistics of the Federal government and the state governments only. When this happens, object codes “G” represents the “Purchase of Land and Existing Structures” for expenditure statistics of Federal and state governments. These definitions apply in public use data files containing state only or Federal only data.

For expenditure statistics on local governments and for all expenditure statistics that cover more than

one level of government, however, “G” represents a different component of capital expenditure, namely “Capital Outlay Other Than Construction.” There is no separate measure of equipment only purchases for local governments in the Census Bureau program.

The use of the “G” code for “Purchase of Land and Existing Structures” applies only during the original compilation of data for the Federal and state governments. When these data are inserted into the Census Bureau’s data files for state and local governments, they are combined with “Purchase of Equipment” data (“K” codes) to produce the category called “Capital Outlay Other Than Construction.” The equipment only category (“K” codes), therefore, becomes a subcategory under this one, but applies only to states. For all local governments, and for all state plus local statistics, the “G” code represents the purchase of land, equipment, and existing structures – i.e., all capital outlays other than construction.

The “J” object code, representing “Assistance and Subsidies,” was introduced effective with 2005. Prior to 2005, this object category was represented by a mix of “E,” “I”, and “J” object codes that were used for multiple object categories.

5.2.2.1 Direct Expenditures Defined

Direct expenditure comprises all final expenditures paid to current employees, former employees (retirees) and to private sector entities outside of the government itself (e.g. all expenditure other than intergovernmental expenditure).

Types of direct expenditure are noted in Chart 5.A above, with fully detailed definitions and explanations contained in Table 5.1. Summary definitions of the types of direct expenditure follow:

Current Operations: Direct expenditure for compensation of own officers and employees and for supplies, materials, and contractual services except any amounts for capital outlay (i.e., for personal services or other objects used in contract construction or government employee construction of permanent structures and for acquisition of property and equipment).

Interest on Debt: Amounts paid for the use of borrowed money.

Assistance and Subsidies: Direct cash assistance to foreign governments, private individuals, and nongovernmental organizations (e.g., foreign aid, agricultural supports, public welfare, veteran bonuses, and cash grants for tuition and scholarships) neither in return for goods and services nor in repayment of debt and other claims against the government.

Capital Outlay: Direct expenditure for purchase or construction, by contract or government employee, construction of buildings and other improvements; for purchase of land, equipment, and existing structures; and for payments on capital leases.

Construction: Production, additions, replacements, or major structural alterations to fixed works, undertaken either on a contractual basis by private contractors or through a government’s own staff.

Purchase of Land and Existing Structures: Acquisition of these assets as such by outright purchase; payments on capital lease-purchase agreements or installment purchase contracts; costs associated with eminent domain (including purchase of rights-of-way); and tax or special assessment foreclosure.

Purchase of Equipment: Purchase and installation of apparatus, furnishings, motor vehicles, office equipment, and the like having a life expectancy of more than five years.

Other Than Construction: This object category represents the combined definitions for "Purchase of Land and Existing Structures" plus "Purchase of Equipment."

5.2.2.2 Intergovernmental Expenditures Defined

Intergovernmental expenditure is defined as amounts paid to other governments for performance of specific functions or for general financial support. Includes grants, shared taxes, contingent loans and advances, and any significant and identifiable amounts or reimbursement paid to other governments for performance of general government services or activities.

By definition, it excludes amounts paid to other governments for purchase of commodities, property, or utility services and for any tax levied as such on facilities of the government. See Section 3.7.3 for a more extensive discussion of this topic.

Specific object codes further define the type of intergovernmental expenditure by level or type of receiving government, as noted in Chart 5.A (see Section 5.2.2).

5.3 The Four Sectors of Government – Expenditure Issues

As described in Section 2.1, the activities of governments are divided into four sectors for Census Bureau purposes. The explanations below provide additional information about these sectors in terms of how they apply to government expenditure statistics.

5.3.1 General Expenditure

General expenditure comprises all expenditure except that classified as utility, liquor store, or social insurance trust expenditure.

5.3.2 Utility Expenditure

Utility expenditure comprises outlays for the purchase or construction of utility facilities, interest on utility debt (including utility debt held by other funds of the same government), and production or acquisition and distribution of utility commodities and services for sale to the general public or to other governments by the four types of state and local government utilities recognized by the Census Bureau: water supply, electric power, gas supply, and public mass transit systems. Utility expenditure is reported on a gross amount without deducting its related revenue.

Utility expenditure excludes any identifiable costs for providing services or commodities to the parent government, which are reported at the function using them. For example, the cost of water used by a

fire department is classified as *Local Fire Protection*, code E24.

The following expenditures are excluded from the utility sector:

- Depreciation of assets (instead, capital outlays are reported at the time of their payment).

- Contributions or other intragovernmental transfers by a utility to its parent government. As a practical matter, interest on utility debt held for investment purposes by other funds of the parent government are included in utility interest expenditure.

- Expenditures relating to utility facilities owned but leased to others for operation (reported as general expenditure), except where the government maintains day-to-day financial oversight of the utility operation.

- Intergovernmental payments (which are always general expenditure) for support of or for contributions toward construction of utilities operated by other governments (see Section 5.3.2.2). Note that purchase of utility services or commodities as a regular customer from another government is treated as a current operation expenditure of the paying function.

- Any expenditure not directly related to the utility's acquisition, operation, capital improvements, or interest on its debt. This includes, for instance, administration of investments and other nonoperating expenses, and payments to other governments as "in-lieu-of-taxes" or as actual taxes on utility property. Generally, these would be classed in the general government sector.

- Contributions to an insurance trust fund administered by the same government (an intragovernmental transfer) or benefit payments to former utility employees who are members of a government public employee retirement system (reported as an insurance trust expenditure). Contributions to a social insurance trust system administered by another government, however, would be classified as utility current operation expenditure.

- Cost of producing electricity or natural gas as a by-product of waste resource recovery facilities (report at *Solid Waste Management*, code *81).

Utility expenditure is categorized according to the type of utility involved and by character and object.

5.3.2.1 Special Topics: Intergovernmental Expenditure Codes for Utilities

The Census Bureau classification system includes intergovernmental expenditure codes for utility purposes. However, despite the fact that these categories possess utility function codes (*91, *92, *93, and *94), they are classified as general government expenditure. These categories were added effective with fiscal year 1988 data. Prior to that time, they were reported under other intergovernmental expenditure codes (primarily *Other and Unallocable*, code *89, which still exists, and Transit Subsidies, code *47, which no longer exists).

5.3.2.2 Special Topics: Joint Utility Projects (Public and Private)

Because of the massive size of utility facilities (especially electric power), governments sometimes enter into joint construction projects with other private or public utilities. Below are two possible situations and their proper classification for Census Bureau purposes:

- In joint public/private utility projects, capital outlay expenditure from bond funds for new plants are classified as utility Construction expenditure (“F” code) rather than Purchase of Land and Existing Structures (“G” code). That is, it is assumed that the government is paying the private firm for the construction work unless there is strong evidence indicating that the government utility is taking over an existing private utility.

- In joint public/public utility projects, payments to another government either for construction or debt service generally is treated as intergovernmental expenditure (using the codes for utilities described in Section 5.3.2.1). However, if the receiving government utility treats the contribution as part of its basic rate structure (i.e., classified as utility revenue), then the government’s contribution is treated as a current operation expenditure.

5.3.3 Liquor Stores Expenditure

Liquor store expenditure is restricted to outlays for the establishment and operation of retail liquor stores or wholesale liquor monopolies owned and operated by state and local governments. It includes amounts for purchase of liquor for resale. It excludes expenditures for enforcement and licensing under liquor laws, even if financed by liquor store sales (reported as general expenditure). It also excludes the transfer of surplus or earnings of the liquor store system to the parent government (an intragovernmental transfer); and state liquor store earnings that are distributed to local governments (reported at *General Local Government Support*, code *30).

5.3.4 Social Insurance Trust Expenditure

Social insurance trust expenditure consists of social insurance payments to beneficiaries, public employee retirement annuities and other benefits, and withdrawals of insurance or employee retirement contributions. In effect, it only includes amounts paid to beneficiaries or members of the systems.

The following are excluded from social insurance trust expenditures:

- Costs of administering social insurance trust programs, including management of investments (classified as a general government expenditure).

- Contributions to a government’s own insurance trust funds (an intragovernmental transfer).

- Contributions or other payments to insurance trust funds administered by another government (classified as current operation expenditure when such amounts represent employer contributions on behalf of the contributing government’s employees and as agency transaction when such amounts represent sums withheld from employee salaries and wages).

- “Pay-as-you-go” plans or “gratuities” to former employees – i.e., payments not involving a plan of employee assessments or accumulation of retirement reserves (classified as current operation expenditure).

• Group insurance premiums covering the government's own employees and contributions to private insurers or to public employee association retirement systems (classified as current operation expenditure).

Social insurance trust expenditure is classified according to the major types of insurance trust systems recognized by the Census Bureau.

5.4 Measurement Issues for Expenditure

This section notes several important concepts related to the measurement of expenditure in the Census Bureau's statistical system. Further detail is available in Chapters 1, 2, and 3.

5.4.1 Measurement Issues: Timing

Expenditure statistics represent all transactions made during the fiscal year. In cases where a dependent agency of a government has a fiscal year that differs from the parent government, expenditures for the dependent agency are combined with that of the parent, even though they represent different time frames.

Similarly, when expenditure statistics are tabulated for more than one government, the fiscal year differences are ignored. All data are summed as if they covered the same time periods, without any adjustments.

The treatment of fiscal years and the definition applied by the Census Bureau are explained in Section 3.2.

5.4.2 Measurement Issues: Valuation

All statistics are reported or compiled in current dollars only. The Census Bureau makes no effort to account for historical price level changes in its statistical program on government finances. This is because of the diversity of users, and uses for, the data. By reporting only in current dollar values, the Census Bureau recognizes the various needs and special circumstances of its data users and does not introduce any statistical bias to the time series.

5.4.3 Measurement Issues: Aggregation and Tabulation

Aggregate statistics for an individual government reflect the expenditure of the parent government and all of its dependent agencies. However, flows of funds between these entities are considered internal transfers and are excluded, by definition, from expenditure totals. The basic concept of total expenditure for an individual government thus represents all monies spent outside of the defined government entity.

Tabulated statistics on expenditure for multiple governments reflect the fiscal years of the governments being summed. Since these fiscal years differ, total statistics (such as for all local government in a state, or all townships nationally) reflect a mix of fiscal periods. The Census Bureau makes no effort to adjust aggregates so that they represent a standard time period.

5.5 Types of Expenditure Statistics – Regular, Exhibit, and Derived

Government expenditures are presented using three of the four basic statistic types in the classification

system – regular, exhibit, and derived. These were explained in Section 2.2. There are no descriptive statistics for government expenditure categories.

The exhibit codes and derived statistics provide additional detail about financial transactions that are deemed important for certain statistical users, or that are used to develop special tabulations of government activities.

These special codes are labeled "expenses" to distinguish them from the "expenditure" categories used in the Census Bureau's classification system. These expense codes are explained briefly in this chapter under their related expenditure category. Additional information is contained in Chapters 8, 9, and 10. The exhibit codes and derived statistics related to expenditure are noted in the description sheets for expenditure function categories (Section 5.6.3), as well as in the tables that accompany this chapter. In total, there are seventeen exhibit code statistics and one derived statistic (found in the liquor stores sector) for expenses.

Two exhibit codes fall outside of the standard "sector" designations used in the classification system:

Z00 = total salaries and wages
V98 = total capital outlay

See the Expenditure Function description pages in Section 5.6.3 for more detail.

5.6 Functional Categories for Regular Finance Expenditure Statistics

The following pages contain the complete list of functional categories and codes used for classifying the Census Bureau's government finance statistics on expenditures.

5.6.1 Overview

The functional expenditure categories used in the finance survey are similar to those used for statistics in the government employment survey. Most of the functional categories are the same in both surveys, and they are defined identically, covering the same types of government services or programs. See Chapter 12 for detailed information about employment statistic functions.

5.6.2 Special Topics: Federal Government Expenditure Statistics

The Census Bureau has included government expenditure statistics of the Federal Government in its government finance program over the years. However, such statistics were temporarily discontinued effective with the 1996 survey year. Nevertheless, this edition of the *Classification Manual* contains detailed information on classification of expenditure statistics for the Federal Government.

There are several aspects of Federal expenditure statistics that differ from the state and local government expenditure statistics developed by the Census Bureau. There are eleven general sector expenditure functions that apply only to the Federal Government. There also are three specific function/object combinations within the insurance trust sector that represent Federal-only benefit payments. These are shown here and detailed later in this chapter:

Code *02 - Federal Space Research and Technology
Code *06 - Federal National Defense and International Relations
Code *14 - Federal Postal Service
Code *20 - Federal Veterans' Education Benefits

Code *28 - Federal Veterans' Health
Code *37 - Federal Own Hospitals - Veterans
Code *39 - Federal Other Hospitals - Veterans
Code *51 - Federal Farm Credit Programs
Code *53 - Federal Farm Income Stabilization
Code *57 - Federal Soil, Water, and Electric Energy Resources
Code *58 - Federal Mineral Resources
Code Y25 - Federal Social Security and Medicare
Code Y34 - Federal Veterans' Life Insurance
Code Y45 - Federal Railroad Retirement

Similarly, there are some expenditure functions that, by definition, do not apply to the Federal Government. These include general sector activities as well as all liquor store and utility functions.

Finally, the "object character" code for Assistance and Subsidies, code "J," is applicable to most functions for Federal Government statistics. Where applicable, it is shown in the applicability table at the bottom of each functional page. In contrast, this object category is restricted for state governments to only four functions (J19, J67, J68, and J85), and local governments to only two functions (J67 and J68).

5.6.3 Key to the Expenditure Function Pages

The complete listing of functional categories for government finance expenditure statistics follows. Each function is presented on a separate page, using a standard format that contains the following information:

Definition
Included activities or employees
Excluded activities or employees
Examples (specific governments, by name, or governmental agencies/programs, by name), if applicable
Special Considerations (explains caveats, restrictions in applicability, or other useful information)
Applicability table (by level of government)

The applicability table, at the bottom of each page, indicates which object codes apply to each function. This is needed because some objects apply only to selected functions. The table contains two columns representing expenditure character – direct expenditure and intergovernmental expenditure. Valid object codes for each function are shown under each column.

The Expenditure Function Pages also contain detail about which classification function codes are restricted in use. This is displayed in two methods. First, within the two expenditure character columns (direct and intergovernmental), the applicability table shows only the object codes applicable for the function. If an object code is not listed, then it cannot be used for that function (and there are no data for it). Also, if any of the object codes are restricted (i.e. they do not apply to a level of government), that fact is noted as well. Where restrictions exist, they reflect limitation by definition or statistical methodology. For example, the object code "L" represents intergovernmental expenditure to state governments. By definition, this is restricted to Federal and local governments only. State governments do not make intergovernmental payments to other states, according to the framework of Census Bureaus' government finance statistics program.

The second manner in which classification restrictions are depicted is via the Special Considerations section on each page. If a function is not used for a level or type of government, this fact is indicated under "Special Considerations." In theory, there are very few of these situations, because restricting classification categories creates conflicts with existing statistical standards. However, as a practical matter, some functions will not apply to a level or type of government. School districts, for example, do not operate airports. Similarly, the functional category *Local Fire Protection* does not apply to Federal and state levels of government.

Note the following excerpt from the Airport Function applicability table, as an example:

K01 Equipment (Federal, states)

This reference indicates that the object and function combination of K01 (expenditure for equipment) is valid only for statistics of Federal and state governments.

Users should reference the complete and detailed table of expenditure function applicability (Table 5.2), by level and type of government, which is found at the end of this chapter and repeated in Appendix 2.

EXPENDITURE FUNCTIONS

Code *01 **Air Transportation (Airports)**

Sector: General Government

Definition: Provision, operation, construction, and support of airport facilities serving the public at-large on a scheduled or unscheduled basis. Also includes the regulation of airline industry, if applicable.

Includes: Publicly-operated airfields and related facilities (runways, terminals, control towers, maintenance facilities, and the like); intergovernmental payments for construction, operation, or support of publicly-owned airports; support of private airports; airport police if either an integral part of the airport operating authority or a payment to regular police agency (see Special Considerations below).

Excludes: Purchase and operation of government-owned aircraft – e.g., police helicopters (report at function involved); state civil air patrol or militia (report at *Other and Unallocable*, code *89).

Examples:

- State – Baltimore/Washington International Thurgood Marshall Airport (a Maryland state government dependent agency)
- Special District –Dulles International Airport in Loudoun County, Virginia (operated by a special district government, the Washington Metropolitan Airports Authority).

Special Considerations:

1. Includes publicly-owned airports, even if no scheduled airlines service it, or if its clientele consists of private pilots and aircraft only.
2. For leased facilities, include government's expenditures and exclude the lessees' expenditures.
3. Report payments by an airport authority to a regular police department of the same government at E01 and deduct an equal amount from *Police Protection*, code E62. The rationale for this procedure is that the primary function involved is air transportation.

Applicable Coding Options for this Expenditure Function

Direct Expenditure

E01	Current Operations
F01	Construction
G01	Land and Existing Structures
K01	Equipment (Federal, states)
J01	Assistance and Subsidies (Federal)

Intergovernmental Expenditure

L01	To state governments
M01	To local governments

EXPENDITURE FUNCTIONS

Code *02 **Federal Space Research and Technology**

Sector: General Government

Definition: Federal Government activities for research, development, applications, and support in the areas of aeronautics and space transportation, sciences, and technology.

Includes: Comprises activities of the National Aeronautics and Space Administration (NASA), except payments to public and private universities for scientific research.

Excludes: Department of Defense research and other activities related to missiles, satellites, space weapons, etc. (report at *Federal National Defense and International Relations*, code *06); NASA payments to public and private universities for scientific research (report at *Federal and State Other Education*, code *21).

Examples: The National Aeronautics and Space Administration (NASA) is the only Federal agency whose employees and activity are included at this function.

Special Consideration:
This function applies only to Federal Government financial statistics.

Applicable Coding Options for this Expenditure Function

Direct Expenditure

E02 Current Operations
F02 Construction
G02 Land and Existing Structures
K02 Equipment

J02 Assistance and Subsidies

Intergovernmental Expenditure

L02 To state governments
M02 To local governments

Code *03 **Miscellaneous Commercial Activities, NEC**

Sector: General Government

Definition: Provision and operation of publicly-owned commercial facilities not classified under particular functions, utilities, or social insurance trust activities.

Includes: Expenditure for operating public markets, cemeteries, grain elevators, or disaster insurance systems if classified as part of the general government.

Excludes: Public utilities (use *Utilities* codes *91 – *94); toll highways such as bridges (report at *Toll Highways*, code *45), sewerage systems (report at *Sewerage*, code *80); public hospitals (report at *Hospitals*, code *36); parks and recreational facilities (use *Parks and Recreation*, code *61); dormitories, cafeterias, bookstores, and other auxiliary enterprises connected with institutions of higher education (use *Higher Education Auxiliary Enterprises*, code *16); nursing homes (report at *Public Welfare*, codes *77 and *79); public airports (use *Airports*, code *01); and other commercial-type activities classifiable in particular functions.

Examples:

- State government – the Bank of North Dakota, North Dakota Mill and Elevator Association, Florida Hurricane Catastrophe Fund, and the Alaska Railroad.
- Local government – New Orleans Public Belt Railroad and Passenger Terminal.

Special Considerations:

1. This function includes direct expenditures only – report any intergovernmental expenditure data at *Other and Unallocable*, code *89.
2. Effective with 1977 data, state government utility expenditures were removed from this category and reclassified at the *Utility* functions, codes *91–*94.
3. Excludes disaster insurance systems if they have been established as separate "other social insurance trust systems." An example of this exclusion is the Florida Citizens Property Insurance Corporation.
4. This function does not apply to Federal government financial statistics.

Applicable Coding Options for this Expenditure Function

Direct Expenditure		Intergovernmental Expenditure
E03	Current Operations	None
F03	Construction	
G03	Land and Existing Structures	
K03	Equipment (states)	

Code *04 **Correctional Institutions**

Sector: General Government

Definition: Residential institutions or facilities for the confinement, correction, and rehabilitation of convicted adults, or juveniles adjudicated, delinquent or in need of supervision, and for the detention of adults and juveniles charged with a crime and awaiting trial.

Includes: Facilities generally referred to as prisons, reformatories, jails, penitentiaries, correctional farms, workhouses, reception centers, industrial schools, training schools, and detention centers. Includes hospitals for the criminally insane IF operated by a corrections agency. Includes expenditure on education, training, and health care programs devoted to inmates. Includes residential work release units, and residential halfway houses, and community corrections centers. For prison industries include only amounts involved in the manufacture, sale, and distribution of articles or services for resale or use outside the government involved.

Excludes: Payments to other governments for care of prisoners (report at *Other Corrections*, code *05); nonresidential facilities (use code *05); any identifiable costs of products or allocable value of prison labor used by other agencies of same government (report at function involved); hospitals for criminally insane operated by mental health or hospital agency (report at *Hospitals*, code *36).

Examples:
• Federal Government – U.S. Penitentiary, Leavenworth

• State government – Pelican Bay State Prison, California

Special Considerations:
1. Prior to 2005, this function applied solely to Federal and state government direct expenditures. Related local expenditures, as well as all intergovernmental expenditures, were included at *Other Corrections*, code *05.

2. Include only facilities operated by the government concerned.

3. Prior to the 1988 version of this Classification Manual, this function was defined to exclude jails holding persons awaiting trial or serving short-term sentences and to report them as *Police Protection*, code *62.

Applicable Coding Options for this Expenditure Function

Direct Expenditure

E04 Current Operations
F04 Construction
G04 Land and Existing Structures
K04 Equipment (Federal, states)

J04 Assistance and Subsidies (Federal)

Intergovernmental Expenditure

L04 To state governments
M04 To local governments

EXPENDITURE FUNCTIONS

Code *05 **Other Corrections**

Sector: General Government

Definition: Correctional activities other than Federal, state and local residential institutions or facilities, as described under *Correctional Institutions.*

Includes: Probation offices (whether operated by courts or correctional agencies) boards of parole, boards of pardon, and the like; noninstitutional activities such as administration of a correctional agency, training of correctional employees, and nonresidential halfway houses and community corrections centers.

Excludes: Supervision of individual state or local correctional institutions (use *Correctional Institutions*, code *04); state and local programs strictly devoted to inmates of its institutions (training, education, rehabilitation, health care, etc.), even if recorded under an administrative unit of a corrections agency – e.g., Director's Office (use code *04).

Special Considerations:

1. Exercise care when recording construction expenditures for corrections reported under an administrative unit (e.g., Director’s Office, Division of Planning). Construction outlays for noninstitutional activities are generally quite small; thus, such amounts should often be classified at *Correctional Institutions*, code *04, instead of *Other Corrections*.
2. Prior to this 2005 edition, this function included all local government residential facilities described under *Correctional Institutions*, code *04.

Applicable Coding Options for this Expenditure Function

Direct Expenditure

E05 Current Operations
F05 Construction
G05 Land and Existing Structures
K05 Equipment (Federal, states)

Intergovernmental Expenditure

L05 To state governments
M05 To local governments

EXPENDITURE FUNCTIONS

Code *06 **Federal National Defense and International Relations**

Sector: General Government

Definition: Federal Government activities to protect the United States and its allies from foreign aggression, to maintain military capabilities for deterring war, to protect and advance its interests in international affairs, and to provide military, economic, and humanitarian aid to other nations.

Includes: Comprises primarily the Departments of Defense, Energy (part), and State as well as such agencies as U.S. Information Agency, Agency for International Development (AID), Peace Corps, Export-Import Bank, and International Monetary Fund. Activities covered include the military services, National Guard and Reserves forces, intelligence agencies, military procurement, defense research and evaluation, military housing, and selective service system; outlays of the Military Retirement Fund; conduct of diplomatic and consular relations, payments to international organizations like the United Nations, and international communication, education, and cultural activities; military assistance; foreign aid for economic support, functional development, and humanitarian assistance; Food for Peace (PL 480 Food Aid) and Foreign Agriculture Service; atomic energy defense research, development, and production.

Excludes: Military and economic loans to other countries (except "forgiven loans"); Soldiers' and Airmen's Home (report at *Public Welfare - Other,* code *79); payments to states for defense activities (report at *Other and Unallocable*, code *89); tuition payments for training (report at *Federal and State Other Education*, code *21); Army Corps of Engineers (allocate among *Federal Soil, Water, and Electric Energy Resource*, code *57, *Sea and Inland Port Facilities*, code *87, and code *89); Department of Energy other than atomic defense activities (report balance at code *57); state-local militia, civil air patrol, civil defense, and other defense-related activities (use code *89).

Special Considerations:

1. This function applies solely to Federal Government financial statistics. Report any related state or local government activities at *Other and Unallocable*, code *89.

2. Report military service academies (the Military Academy at West Point, the Air Force Academy, the Naval Academy, Merchant Marine Academy, and the Coast Guard Academy) here rather than at *Education*, codes *12 – *21.

Applicable Coding Options for this Expenditure Function

Direct Expenditure		Intergovernmental Expenditure
E06	Current Operations	None
F06	Construction	
G06	Land and Existing Structures	
K06	Equipment	
J06	Assistance and Subsidies	

Code *12 **Elementary and Secondary Education**

Sector: General Government

Definition: The operation, maintenance, and construction of public schools and facilities for elementary and secondary education (kindergarten through high school), vocational-technical education, and other educational institutions except those for higher education. Covers operations by independent governments (school districts) as well as those operated as integral agencies of state, county, municipal, or township governments. Also covers financial support of public elementary and secondary schools.

Includes: Instructional, support, and auxiliary services operated through school systems (school lunch, student activities, community services, pupil transportation, health services, guidance counseling, and the like); administration and supervision of school systems; special education, classes for the handicapped, and vocational education provided through school systems; libraries operated by public schools; and plant maintenance and operation. For state governments, includes payments in support of local school systems as well as direct expenditures on their behalf (e.g., for construction of school buildings, purchase of textbooks and other instructional materials, acquisition and operation of school buses, and other local school activities).

Excludes: Institutions of higher education (use *Higher Education Auxiliary Enterprises*, code *16, and *Other Higher Education*, code *18); schools for the blind, deaf, or handicapped (if primarily for training and education, report at *Federal and State Other Education*, code *21; if primarily for physical rehabilitation and care, report at *Hospitals*, codes *36); state aid to private schools (report at *State Government Scholarships and Other Subsidies*, code *19); state adult, vocational, and special education programs operated outside school systems (use code *21). For state governments also exclude administrative expenses of school building agencies and supervision of local public and private elementary-secondary education (use code *21).

Examples:

- State governments – The Patterson City School District (New Jersey), under temporary state control. The elementary and secondary education system in Hawaii, where the state government operates the sole school system.
- Local governments – The Los Angeles Unified School District (an independent local government) and the New York City School System (classified as a dependent agency of New York City).

Code *12 **Elementary and Secondary Education**

Special Considerations:

1. Includes all expenditures for public charter schools offering elementary and secondary educations, if they have been classified as in-scope for Census Bureau surveys on government finances. State payments to charter schools classified as private for Census Bureau statistics (report payment at *State Government Scholarships and Other Subsidies*, code J19, for state governments and E12 for local governments).

2. Q12 is used for payments to independent school districts, where separately identifiable, rather than using M12.

3. This function applies to state and local governments only. All Federal expenditure for education is reported at *Federal and State Other Education*, code *21.

Applicable Coding Options for this Expenditure Function

Direct Expenditure		Intergovernmental Expenditure	
E12	Current Operations	L12	To state governments (locals)
F12	Construction	M12	To local governments
G12	Land and Existing Structures	Q12	To independent school districts (states, schools only)
K12	Equipment (states)		

EXPENDITURE FUNCTIONS

Code *14 **Federal Postal Service**

Sector: General Government

Definition: Activities of the U.S. Postal Service (USPS).

Includes: Includes all postal activities on a gross reported basis (i.e., without deducting postal fees and charges).

Excludes: Subsidies to airlines (report at *Air Transportation*, code *01).

Special Considerations:

1. The former cabinet-level Post Office Department became an independent Federal corporation and renamed the U.S. Postal Service on July 1, 1971, as a result of the Postal Reorganization Act of 1970 (Public Law 91-375).
2. Report USPS criminal and fraud investigation activity here, rather than at *Police Protection*, code *62.

Applicable Coding Options for this Expenditure Function

Direct Expenditure		Intergovernmental Expenditure
E14	Current Operations	None
F14	Construction	
G14	Land and Existing Structures	
K14	Equipment	
J14	Assistance and Subsidies	

EXPENDITURE FUNCTIONS

Code *16 **Higher Education Auxiliary Enterprises**

Sector: General Government

Definition: Higher education activities and facilities that provide supplementary services to students, faculty or staff, and which are self-supported (wholly or largely through charges for services) and operated on a commercial basis.

Includes: Dormitories; cafeterias; bookstores; athletic facilities, contests, or events; student activities; lunch rooms; student health services; college unions; college stores, and the like.

Excludes: Hospitals operated by medical schools (report at *Hospitals*, code *36); agricultural extension services (report at *Natural Resources, Other*, code *59).

Special Considerations:

1. Report expenditures on a gross basis without deduction for related revenue from charges.
2. Report any employment data related to these activities at *Other Higher Education*, code *18.
3. Prior to this 2005 edition, this function applied solely to state governments only. Auxiliary enterprises associated with local institutions of higher education were reported at *Other Higher Education*, code *18.
4. Prior to this 2005 edition, the exclusion for agricultural extension services was coded at Agriculture, code *54, instead of *Natural Resources, Other*, code *59.
5. This function does not apply to Federal Government financial statistics.

Applicable Coding Options for this Expenditure Function

Direct Expenditure		Intergovernmental Expenditure
E16	Current Operations	None
F16	Construction	
G16	Land and Existing Structures	
K16	Equipment (states)	

EXPENDITURE FUNCTIONS

Code *18 **Other Higher Education**

Sector: General Government

Definition: Degree-granting institutions (associate, bachelor, master, or doctorate) operated by state or local governments that provide academic training beyond the high school (grade 12) level, other than for auxiliary enterprises of the state or local institution.

Includes: Junior colleges; community colleges; universities; law schools; medical and nursing schools; agricultural colleges; land grant institutions; engineering schools; and other institutions granting postsecondary degrees. Includes all related activities for instruction, research, public service (except agricultural extension services), academic support, libraries, student services (other than self-supporting enterprises), administration, and plant maintenance.

Excludes: Dormitories, bookstores, cafeterias, and other commercial-type services of institutions (report at *Higher Education Auxiliary Enterprises,* code *16); training academies or programs which do not confer college-level degrees (e.g., police academies); state vocational-technical schools which award certificates equal to less than 2-years of college (report at *Federal and State Other Education*, code *21); hospitals for the general public operated by universities (report at *Hospitals*, code *36); agricultural experiment stations, farms, and extension services (report at *Natural Resources, Other*, code *59); state scholarships and fellowships awarded to students (report at *State Government Scholarships and Other Subsidies*, code *19); state aid to or in support of private colleges (use code J19); state administration of school building authorities (use code *21).

Examples: State governments – State University of New York (SUNY) system, including all graduate programs, four year universities, and colleges (dependent agencies of the state of New York).

Special Considerations:

1. Finance data for both *Higher Education Auxiliary Enterprises,* code *16, and code *18 are obtained in part from the Integrated Postsecondary Education Data Survey (IPEDS) conducted by the U.S. Department of Education.
2. This function does not apply to Federal Government financial statistics, or to statistics for special district governments.

Applicable Coding Options for this Expenditure Function

Direct Expenditure		Intergovernmental Expenditure	
E18	Current Operations	L18	To state governments (locals)
F18	Construction	M18	To local governments
G18	Land and Existing Structures	Q18	To local school districts (states)
K18	Equipment (states)		

EXPENDITURE FUNCTIONS

Code *19 **State Government Scholarships and Other Subsidies (Education)**

Sector: General Government

Definition: State government direct cash payments to individuals for tuition, scholarships, and other financial aid to meet educational expenses (other than loans), whether based on academic merit, financial need, athletic ability, or educational disadvantage. This function also includes direct cash subsidies to private schools and colleges.

Includes: Scholarships (including those for specific areas of study, such as medicine or dentistry); fellowships; tuition or fee aid, credits, waivers, remissions, etc.; payments under outright grants; trainee stipends; student prizes; state veterans educational opportunity grants; financial aid to private or parochial schools and colleges; State Student Incentive Grants (SSIG); Supplemental Educational Opportunity Grants (SEOG).

Excludes: Pell Grants passed through state institutions (treat as direct Federal aid to student – i.e., as an agency transaction); student loan programs; college work-study awards or other payments under programs where recipient must provide services to the educational institution, such as teaching undergraduate students (report at *Other Higher Education*, code *18)

Special Considerations:

1. This function applies solely to state governments. Classify any related local cash assistance payments at *Other Higher Education*, code E18.
2. Statistics on direct cash payments made by institutions themselves are collected in part through the Integrated Postsecondary Education Data Survey (IPEDS) – see Other Higher Education, code *18 for details, while data on direct cash payments made by other agencies (e.g., state scholarship commissions or boards of higher education) are obtained from other sources.
3. There is no corresponding function in the Census Bureau's employment statistics.
4. This function does not apply to Federal Government financial statistics.
5. Payments to charter schools classified as private for Census Bureau statistics (report payments by state governments at J19 and payments by local governments at *Elementary and Secondary Education*, code E12).

Applicable Coding Options for this Expenditure Function

Direct Expenditure		Intergovernmental Expenditure
J19	Assistance and Subsidies	None

EXPENDITURE FUNCTIONS

Code *20 **Federal Veterans' Education Benefits**

Sector: General Government

Definition: Federal Government direct payments to or on behalf of veterans, former military personnel, and their eligible dependents for education and training purposes and intergovernmental aid in support of state and local government veterans educational benefits programs.

Includes: Veterans readjustment benefits for education and training; educational assistance under various "GI Bills;" All Volunteer Force education benefits; Post-Vietnam Era Education Program payments.

Excludes: Loans to veterans for education and training (nonexpenditures); distribution of funds from the Post-Vietnam Era Veterans Education Trust Fund which represents repayments of voluntary contributions by eligible military personnel during their service (report at *Federal and State Other Education*, code *21); tuition payments for training by Department of Defense for active personnel (use code *21).

Special Considerations:

1. The benefit programs cited above are administered by the U.S. Department of Veterans Affairs although some are funded by the Department of Defense.
2. This function applies solely to Federal Government financial statistics. Report any state government direct cash payments to veterans for tuition, scholarships, or other educational assistance at *State Government Scholarships and Other Subsidies*, code *19, and any such local government assistance at *Other Higher Education*, code *18.

Applicable Coding Options for this Expenditure Function

Direct Expenditure

E20 Current Operations
F20 Construction
G20 Land and Existing Structures
K20 Equipment

J20 Assistance and Subsidies

Intergovernmental Expenditure

L20 To state governments
M20 To local governments

Code *21 **Federal and State Other Education**

Sector: General Government

Definition: State government special programs and institutions primarily for training and education (rather than care) of the blind, deaf, or other handicapped; programs for adult, vocational, or special education that operate outside school systems; and activities not assignable to other education functions.

Includes: State schools for the blind, visually impaired, deaf, or other handicapped; adult education and vocational rehabilitation and education not provided by school systems; technical or vocational-technical schools which award certificates equal to less than two years of college; overall supervision of and general services to local elementary and secondary schools, public or private; administration of state education activities; costs not allocable between elementary-secondary and higher education, such as paying fringe benefit costs of employees of local schools and state colleges; administration of state school building authorities.

Excludes: Institutions for blind, deaf, or other handicapped that are primarily for physical rehabilitation and care (report at *Hospitals*, code *36); direct payments on behalf of local schools, such as for textbooks, purchase of buses, and construction of school buildings (report at *Elementary and Secondary Education*, code *12); state intergovernmental aid to support local school districts or dependent public school systems (use code *12); administration of state institutions of higher education (report at *Other Higher Education*, code *18).

Examples:

- Federal Government – The Department of Education and the National Science Foundation, plus parts of the Bureau of Indian Affairs.
- State governments – Includes boards of governors of the state college/university systems, school finance authorities, educational facilities authorities, and commissions on higher education.

Special Considerations:

1. This function applies only to Federal and state governments and to special district governments. For the Federal Government, it represents all education expenditures except veterans' benefits (*Federal Veterans' Education Benefits*, code *20). Prior to this 2005 edition, it was named "Other Education," but still excluded local governments. The code name was modified accordingly.
2. Report any related local data at either *Elementary and Secondary Education*, code *12 or *Other Higher Education*, code *18.

EXPENDITURE FUNCTIONS

Code *21 **Federal and State Other Education**

page 2 of 2

Applicable Coding Options for this Expenditure Function

Direct Expenditure

E21	Current Operations
F21	Construction
G21	Land and Existing Structures
K21	Equipment
J21	Assistance and Subsidies (Federal)

Intergovernmental Expenditure

L21	To state governments
M21	To local governments

EXPENDITURE FUNCTIONS

Code *22 **Social Insurance Administration**

Sector: General Government

Definition: Administration of unemployment compensation systems, public employment services, and the Federal Social Security, Medicare, and Railroad Retirement trusts.

Includes: Unemployment compensation, unemployment insurance, and equivalent agencies involved in administering the cooperative Federal-state unemployment insurance compensation system; associated public employment, job services, employment services, and other agencies providing job placement, counseling, veterans readjustment allowances, or related services; and determination of eligibility for disability benefits under Federal Social Security (Old-Age and Survivors Insurance and Disability Insurance) and Medicare (Hospital Insurance and Supplementary Medical Insurance) programs. Also includes intergovernmental payments from the Federal government to state governments for administering unemployment compensation, job services, and disability determination programs.

Excludes: Benefits paid under these programs (report at appropriate *Social Insurance Trust* code); administration of public employee retirement, workers' compensation, or miscellaneous insurance trusts (report at *Financial Administration*, code *23); administration of Federal Supplemental Security program, or SSI (report at *Public Welfare - Other*, code *79); activities funded by the Federal Workforce Investment Act – WIA (formerly the Federal Job Training Partnership Act – JTPA, report at *Other and Unallocable*, code *89); monies channeled through public employment offices (report at function involved).

Special Considerations:

1. Report any state government intergovernmental payments at *Other and Unallocable*, code *89.
2. Washington, DC is the only local government for which data can be reported at this function.
3. This function is titled *Employment Security Administration* when applied to state governments and Washington, DC only.

Applicable Coding Options for this Expenditure Function

Direct Expenditure

E22	Current Operations (Federal, states, DC)
F22	Construction (Federal, states, DC)
G22	Land and Existing Structures (Federal, states, DC)
K22	Equipment (Federal, states)
J22	Assistance and Subsidies (Federal)

Intergovernmental Expenditure

L22	To state governments (Federal)
M22	To locals (Federal)

EXPENDITURE FUNCTIONS

Code *23 **Financial Administration**

Sector: General Government

Definition: Officials and central staff agencies concerned with tax assessment and collection, accounting, auditing, budgeting, purchasing, custody of funds, and other finance activities.

Includes: Expenditures to administer the offices of auditor, comptroller, treasurer, finance director, and other central accounting, budgeting, and purchasing offices; tax administration, assessment, billing, and collection; custody and disbursement of funds; revenue collection activities like delinquent tax sales, tax litigation, and charges of depositories; state supervision of local finances; management of debt and of investments (including that of own utilities); cost of insurance for issuing debt; administration of employee-retirement, workers' compensation, and federal and state other insurance trust funds; lottery administrative costs (equal to the amount at *Administrative Expenses*, exhibit code Z53); licensing and tax collection activities of motor vehicle departments; distinctive tax collection activities of regulatory agencies; central data processing centers; and other finance activities not recorded elsewhere.

Excludes: Internal financial management activities of functional agencies (report at function of agency involved); administration of unemployment compensation systems and of Federal Social Security and Railroad Retirement Trust Funds (report at *Social Insurance Administration*, code *22); benefits paid from employee retirement, workers' compensation, and state other insurance trusts (report at appropriate *Social Insurance Trust* code); activities of motor vehicle departments other than licensing (report at *Protective Inspection and Regulation, NEC*, code *66); installation and maintenance of government owned parking meters (report at *Parking Facilities*, code *60); purchase and maintenance of real property held for investment (report at *Other and Unallocable*, code *89).

Examples: Federal Government – the Federal Reserve System and Bureau of Engraving and Printing and U.S. Mint.

Special Consideration:
This function covers central offices and excludes internal financial management activities of functional agencies (report at function of agency involved). This function does not apply to special districts or to school district governments.

Applicable Coding Options for this Expenditure Function

Direct Expenditure

E23 Current Operations
F23 Construction
G23 Land and Existing Structures
K23 Equipment (Federal, states)

J23 Assistance and Subsidies (Federal)

Intergovernmental Expenditure

L23 To state governments
M23 To local governments

EXPENDITURE FUNCTIONS

Code *24 **Local Fire Protection**

Sector: General Government

Definition: Prevention, avoidance, and suppression of fires and provision of ambulance, medical, rescue, or auxiliary services provided by fire protection agencies.

Includes: Expenditures of regular fire departments; financial, technical, and operational support of volunteer fire forces; fire hydrant maintenance; rescue squads; fire inspection, investigation, and regulation; fire marshals; fire prevention education; fire suppression training; auxiliary services; fire stations, buildings, and other facilities used exclusively for fire protection purposes; and the following activities IF handled by a fire department: ambulances, emergency medical technicians (EMTs), paramedic squads, and arson investigation. Also include any identifiable amounts for services rendered by other agencies of the same government for fire protection or suppression, such as provision of water by water supply utility.

Excludes: Forest-fire protection and suppression (report state expenditure at *Federal and State Forestry*, code *56, and local amounts at *Natural Resources, Other*, code *59); ambulances, emergency medical technicians, and paramedic squads handled by non-fire departments (report at *Health*, code *32).

Examples: Expenditures of the New York City Fire Department, including its EMT and ambulance activity, and all its training activities.

Special Considerations:

1. Effective with 1988 data, the treatment of ambulance and emergency medical services was clarified to include it here only if handled by a fire protection agency.

2. This function applies only to local governments. Federal and state government data are reported at *Other and Unallocable*, code *89, except forest fire protection and suppression (use *Federal and State Forestry,* code *56).

Applicable Coding Options for this Expenditure Function

Direct Expenditure		Intergovernmental Expenditure	
E24	Current Operations	M24	To local governments (locals)
F24	Construction		
G24	Land and Existing Structures		

Code *25 **Judicial and Legal**

Sector: General Government

Definition: Courts (criminal and civil) and activities associated with courts, legal services, and legal counseling of indigent or other needy persons.

Includes: Expenditures for criminal and civil courts of limited and general jurisdiction; appellate courts; juries, court reporters, witness fees, and law libraries; medical and social service activities of courts (except probation); court activities of sheriff offices (bailiffs or "civil" functions); registers of wills and other probate activities; legal departments, general counsels, solicitors, prosecuting and district attorneys; attorneys providing government-wide services; public defenders; payments for court-appointed lawyers; indigent defense; child support enforcement; and contributions to legal aid societies.

Excludes: Probation (report at *Other Corrections*, code *05); judgments and claims and crime victim compensation or reparation (report at *Other and Unallocable*, code *89); boards of appeal for zoning, tax assessment, workers' compensation, or other nonjudicial areas adjudication related strictly to administrative rule-making, "judges" which are administrative or executive positions (report at *Central Staff Services*, code *29).

Examples:
- Federal Government – U.S. Supreme Court and Circuit Courts of Appeal.
- State governments – Supreme Court of California and State Courts of Appeal.
- Local governments – Superior Courts in California, classified as county agencies, except for San Francisco (city).

Special Considerations:
1. Effective with 1982 data, this category was expanded from court activities only to also encompass legal services and public defense, formerly classified under *Central Staff Services*, code *29, and *Public Welfare - Other*, code *79, respectively, and to cover all general purpose governments instead of states, and large cities and counties.
2. This function does not apply to special district or to school district governments, by definition.

Applicable Coding Options for this Expenditure Function

Direct Expenditure		Intergovernmental Expenditure	
E25	Current Operations	L25	To state governments
F25	Construction	M25	To local governments
G25	Land and Existing Structures		
K25	Equipment (Federal, states)		
J25	Assistance and Subsidies (Federal)		

Code *26 **Federal and State Legislative**

Sector: General Government

Definition: Legislative bodies and related activities involved in the making, enacting, and repeal of law.

Includes: Expenditures for Federal and state government legislatures, including their research and investigative agencies and all committees directly responsible to the legislature. Includes support activities such as construction of legislative office buildings and the like.

Excludes: Major agencies within the legislative branch operating autonomously and having a specific function. Report U.S. Library of Congress at *Libraries*, code *52, and the U.S. Government Accountability Office (GAO) at *Financial Administration*, code *23.

Examples: Federal Government – United State Congress and its committees.

Special Considerations:

1. This function applies only to Federal and state government expenditures. Report any related local government expenditure data at *Central Staff Services*, code *29.
2. By definition, there is no intergovernmental expenditure at this function. Report any intergovernmental outlays at *Central Staff Services*, code *29.
3. There is no corresponding function in the Census Bureau's employment statistics.

Applicable Coding Options for this Expenditure Function

Direct Expenditure		Intergovernmental Expenditure
E26	Current Operations	None
F26	Construction	
G26	Land and Existing Structures	
K26	Equipment	

EXPENDITURE FUNCTIONS

Code *28 **Federal Veterans' Health**

Sector: General Government

Definition: Federal Government provision of health care services to veterans other than for hospital care.

Includes: Health care activities of the U.S. Department of Veterans Affairs (VA) for: veterans outpatient medical and dental care; veterans medical, rehabilitation, and prosthetic research; payments to private physicians and dentists for medical care provided to veterans.

Excludes: Inpatient health care services at VA hospitals (report at *Federal Own Hospitals - Veterans*, code *37); payments for health care for veterans in other public or private hospitals (report at *Federal Other Hospitals - Veterans*, code *39); Department of Defense health care services for active or retired military personnel (report at *Federal National Defense and International Relations*, code *06).

Special Consideration:
This function applies only to Federal Government financial statistics. Report any related state or local government expenditure data at *Health*, code *32.

Applicable Coding Options for this Expenditure Function

Direct Expenditure		Intergovernmental Expenditure	
E28	Current Operations	L28	To state governments
F28	Construction	M28	To local governments
G28	Land and Existing Structures		
K28	Equipment		
J28	Assistance and Subsidies		

Code *29 **Central Staff Services**

Sector: General Government

Definition: Government-wide executive, administrative, and staff service agencies other than financial, judicial, legal, and Federal or state legislative activities.

Includes: Expenditures for the office of the chief executive, mayor, city manager, county administrator; central personnel administration; overall planning and zoning; clerk's office, recorder, and general public reporting; central staff executive and administrative agencies. For local governments also includes legislative activities such as city or county council, board of supervisors, commissioners, and so forth.

Excludes: Planning activities limited to a specific function and internal control or administrative activities of functional agencies (report at function of agency involved); conduct of elections (report at *Other and Unallocable*, code *89); central finance agencies and data processing centers (report at *Financial Administration*, code *23); multi-purpose public buildings and related services (report at *General Public Buildings*, code *31); clerk of court and other judicial recording activities (report at *Judicial and Legal*, code *25).

Special Considerations:

1. Effective with 1982 data, legal activities formerly included here were reclassified at *Judicial and Legal*, code *25.
2. This function does not apply to special district or to school district governments, by definition.

Applicable Coding Options for this Expenditure Function

Direct Expenditure

E29	Current Operations
F29	Construction
G29	Land and Existing Structures
K29	Equipment (Federal, states)
J29	Assistance and Subsidies (Federal)

Intergovernmental Expenditure

L29	To state governments
M29	To local governments

EXPENDITURE FUNCTIONS

Code *30 **General Local Government Support**

Sector: General Government

Definition: State government intergovernmental payments to local governments that are unrestricted as to the function or purpose for which they may be spent.

Includes: State-collected taxes shared with localities, usually on a formula basis (e.g., taxes on general sales, cigarettes, income, etc.); distribution of profits from alcoholic beverage monopolies; payments-in-lieu-of-taxes; per capita aid; revenue sharing programs; general municipal or county aid unrestricted in its uses (e.g., distributed on basis of population); amounts to reimburse local units for property tax relief, homestead exemptions, and other tax losses; and state redistribution of Federal grazing fees.

Excludes: Aid or grant programs that apply distinctively either to school districts (report at the appropriate *Education* function, codes *12 – *21) or to single-purpose special districts – e.g., drainage districts, road districts, etc. (report at function involved); state taxes collected by local governments and retained as a fee or for other purposes (treat as tax revenue of local government).

Special Considerations:

1. This function applies only to state intergovernmental expenditures. Report any related direct costs for the administration of these programs according to the function of the distributing agency.
2. Report any comparable Federal or local intergovernmental expenditure data at *Other and Unallocable,* code *89.
3. There is no corresponding function in the Census Bureau's employment statistics.

Applicable Coding Options for this Expenditure Function

Direct Expenditure	Intergovernmental Expenditure
None	M30 To local governments

EXPENDITURE FUNCTIONS

Code *31 **General Public Buildings**

Sector: General Government

Definition: Construction, equipping, maintenance, and operation of general public buildings not related to specific functions or agencies.

Includes: General county offices buildings, city halls, multi-purpose office buildings and annexes; and lighting, janitorial, custodial, and other services furnished for general public buildings.

Excludes: School buildings, police stations, firehouses, libraries, institutional buildings, jails, hospitals, courthouses that hold only courts and related judicial agencies, etc. (report at function involved to the extent practicable).

Special Considerations:

1. This function applies solely to state and local government expenditures. Report any related Federal expenditure data at *Other and Unallocable*, code *89.
2. This function includes direct expenditures only. Report any intergovernmental outlays at *Other and Unallocable*, code *89.
3. There is no corresponding function in the Census Bureau's employment statistics.
4. This function does not apply to special district or to school district governments, by definition.

Applicable Coding Options for this Expenditure Function

Direct Expenditure		Intergovernmental Expenditure
E31	Current Operations	None
F31	Construction	
G31	Land and Existing Structures	
K31	Equipment (states)	

Code *32 **Health**

Sector: General Government

Definition: Provision of services for the conservation and improvement of public health, other than hospital care, and financial support of other governments' health programs.

Includes: Expenditures for general health activities, categorical health activities and programs, health-related inspections, community health care programs, regulation of air and water quality, rabies and animal control, and ambulance and emergency medical services ONLY IF handled separately from the local fire department. Also includes state or local expenditure financed by Federal Government "Superfund" for cleanup of hazardous waste sites. Additional examples by category are listed below.

Excludes: Vendor payments for medical appliances, supplies, or services under public assistance programs (use *Vendor Payments for Medical Care*, code E74); examination and licensing of health-related professions – e.g., doctors and nurses (report at *Protective Inspection and Regulation, NEC*, code *66); operation or construction of nursing homes (report at *Public Welfare*, codes *77/*79); vocational rehabilitation (report at *Education*, codes *18/*21); coroners and crime labs (report at *Police Protection*, code *62).

Examples:

- General health activities – public health administration, laboratories, public education, vital statistics, research, alcohol and drug abuse prevention/rehabilitation and other general health activities.
- Categorical health activities – control of cancer, TB, socially transmitted diseases, mental illness, etc. and maternal, activities funded by Federal W.I.C. funds – Women, Infants, and Children, and child health care.
- Health related inspections – inspection of restaurants, water supplies, food handlers, nursing homes, agricultural standards or protection of agricultural products from disease.
- Community health care programs – community and visiting nurses; immunization programs; out-patient health clinics.
- Regulation of air and water quality – sanitary engineering and other environmental activities.
- Animal control – general animal control plus rabies control, abatement of mosquitoes, rodents, and other vermin.
- Federal Government – includes the Food and Drug Administrations and the Environmental Protection Agency (with the exception of grant programs for sewerage construction).

Code *32 **Health**

Special Considerations:

1. Effective 1988, the Census Bureau clarified the classification of nursing homes at *Public Welfare – Institutions* (except inspection of such homes), code *77, and of ambulance services at *Health* only if such service is not organized under a fire department.
2. Effective 2005, the *Agriculture* function was removed as a valid function in government finance surveys. Expenditure for maintaining agricultural standards or for the protection of agricultural products from disease was moved to *Natural Resources, Other,* code *59, rather than to this *Health* function.

Applicable Coding Options for this Expenditure Function

Direct Expenditure

E32 Current Operations
F32 Construction
G32 Land and Existing Structures
K32 Equipment (Federal, states)

J32 Assistance and Subsidies (Federal)

Intergovernmental Expenditure

L32 To state governments
M32 To local governments

Code *36 **Hospitals**

Sector: General Government

Definition: Expenditures related to a government's own hospitals as well as expenditures for the provision of care in other hospitals (public or private). Own hospitals are facilities directly administered by the government, including those operated by public universities. Other expenditures cover the provision of care in other hospitals and support of other public and private hospitals. This function also covers direct payments for acquisition or construction of hospitals (whether or not the government will operate the completed facility) and payments to private corporations that lease and operate government-owned hospitals.

Includes: Hospitals include government operated general hospitals providing in-patient medical care and facilities that provide specialized care. Among the latter are: Institutions for the custody, treatment, or general care of the mentally insane, or emotionally disturbed; TB sanatoria; maternity and children hospitals; orthopedic hospitals; hospitals for chronic diseases; institutions for care and treatment of blind, deaf, developmentally disabled, or other special classes of handicap; hospitals associated with university medical schools.

Expenditures for hospitals not operated by the government include payments for the hospitalization of persons in other public or private hospitals, except payments made under public welfare programs; financial support of other public or private hospitals, including construction; payments to private corporations who lease and operate government-owned hospitals; construction of hospitals to be leased or turned over to others to operate IF government actually supervises or controls their construction.

Excludes: For the Federal Government, exclude veterans hospitals operated by U.S. Department of Veterans Affairs (report at *Federal Own Hospitals - Veterans*, code *37). Most other exclusions involve expenditure for care or treatment under public welfare or expenditure for specialized care that falls into another function.

The most prominent exclusions are: nursing homes (or other welfare institutions) not directly associated with a public hospital (report at *Public Welfare*, codes *77/*79), payments to private vendors for medical care under welfare programs (use *Public Welfare – Vendor Payments for Medical Care*, code E74), payments to private vendors for hospital care administered as part of public medical assistance programs (report at *Public Welfare – Vendor Payments for Medical Care*, code E74), payments for medical care in nursing homes or other welfare institutions unless facility is associated with a hospital (report at *Public Welfare*, codes *77 and *79), infirmaries serving particular institutions, like college infirmaries and prison hospitals (report at function involved), hospitals for criminally insane operated by corrections agency (report at *Correctional Institutions*, code *04), state schools for blind, deaf, or other handicapped primarily for education and training (report at *Federal and State Other Education*, code *21), payments to public hospitals by other agencies of same government (internal transfers).

Code *36 **Hospitals**
page 2 of 2

Examples:

- State governments – Louisiana State University Health Sciences Center – HCSD.
- Local governments – Hurley Medical Center, Flint, Michigan (a dependent agency of the city government); King County Hospital District, state of Washington (classified as a special district government).

Special Considerations:

1. Report public hospital expenditures from Federal Medicaid funds here.
2. Effective 2005, this function includes all state and local government hospital expenditure. From 1985 through 2004, there were two hospital function codes: Hospitals – Own (government operated), code *36 and Other Hospitals, code *38.
3. Effective with 1985 data, the following four state-only categories were consolidated into this single function due to the growing difficulty of distinguishing them: Regular Mental Hospitals, Other Mental Hospitals, General Hospitals, and Own Hospitals, NEC.

Applicable Coding Options for this Expenditure Function

Direct Expenditure

E36	Current Operations
F36	Construction
G36	Land and Existing Structures
K36	Equipment (Federal, states)
J36	Assistance and Subsidies (Federal)

Intergovernmental Expenditure

L36	To state governments
M36	To local governments

EXPENDITURE FUNCTIONS

Code *37 **Federal Own Hospitals - Veterans**

Sector: General Government

Definition: Hospital facilities providing medical care to veterans and institutions primarily for the care and treatment of service-connected disabilities and which are directly administered by the Federal Government.

Includes: Hospitals and related medical facilities operated by the U.S. Department of Veterans Affairs (VA).

Excludes: Payments to other hospital facilities (public or private) for medical care of veterans (report at *Federal Other Hospitals - Veterans*, code *39); VA outpatient medical care (report at *Federal Veterans Health*, code *28); veterans medical and prosthetic research (use code *28); domiciliary medical care and contract nursing home care (report at *Public Welfare - Other*, code *79); health training and education (report at *Federal and State Other Education*, code *21); hospitals operated by the Department of Defense (report at *Federal National Defense and International Relations*, code *06).

Examples: Bay Pines VA Medical Center, St. Petersburg, Florida

Special Consideration:
This function applies only to Federal Government financial statistics. Report any state-operated veterans hospitals at *Hospitals*, code *36.

Applicable Coding Options for this Expenditure Function

Direct Expenditure

E37 Current Operations
F37 Construction
G37 Land and Existing Structures
K37 Equipment

J37 Assistance and Subsidies

Intergovernmental Expenditure

L37 To state governments
M37 To local governments

Code *39 **Federal Other Hospitals - Veterans**

Sector: General Government

Definition: Federal Government provision of hospital care to eligible veterans in hospitals (public or private) other than those operated by the U.S. Department of Veterans Affairs (VA).

Includes: Contract hospitalization of veterans; grants to state governments for hospitalizing veterans in state hospitals and for constructing or improving extended medical care facilities furnishing care to veterans; other payments for care and treatment of veterans in hospital facilities not under the jurisdiction of the VA.

Excludes: Contract care or grants for nursing home care and domiciliary medical care (report at *Public Welfare - Other*, code *79); hospitals operated by the Department of Defense (report at *Federal National Defense and International Relations*, code *06).

Special Considerations:

1. This function applies only to Federal Government financial statistics. Report state or local government payments for care of veterans in other public or private hospitals at *Hospitals*, code *36.
2. There is no corresponding function in the Census Bureau's employment statistics.

Applicable Coding Options for this Expenditure Function

Direct Expenditure

E39	Current Operations
F39	Construction
G39	Land and Existing Structures
K39	Equipment
J39	Assistance and Subsidies

Intergovernmental Expenditure

L39	To state governments
M39	To local governments

EXPENDITURE FUNCTIONS

Code *44 **Regular Highways**

Sector: General Government

Definition: Maintenance, operation, repair, and construction of highways, streets, roads, alleys, sidewalks, bridges, tunnels, ferry boats, viaducts, and related non-toll structures.

Includes: Snow and ice removal and application of salt and sand (including that by sanitation or street cleaning agencies, if identifiable); street or highway lighting and related fixtures; traffic signals; highway and traffic design, planning, and engineering IF handled by public works or highways agency; highway safety; nontoll ferries; operation of drawspans; garages, administrative buildings, and other facilities of highway agencies; construction and maintenance of such highway-related items as curbs, gutters, crosswalks, grade separations, trestles, railroad crossings, and storm drains that are integral to highway projects; intergovernmental payments for highways (toll or free), including state aid for debt service on local highway debt.

Excludes: Patrol or policing of streets and highways and traffic control activities of police or public safety agencies (report at *Police Protection*, code *62), street cleaning activities (report at *Solid Waste Management*, code *81), public parking facilities or meters (report at *Parking Facilities*, code *60), operation of sea and inland port facilities (use *Sea and Inland Port Facilities*, code *87), and roads and walkways within parks maintained by a park agency (report at *Parks and Recreation*, code *61).

Examples:

- Federal Government – most expenditure of the Federal Highway Administration (part of the Department of Transportation), including Highway Trust Fund grants.
- State governments – Virginia Department of Transportation expenditure for interstate highway construction (nontoll).

Special Considerations:

1. Report cost of street lighting furnished by an electric utility operated by same government here and deduct an equal amount from *Electric Power Systems*, code E92.
2. Effective with 1988 data, the treatment of traffic engineering was clarified to include it here unless expressly handled by a police agency.
3. Report all intergovernmental expenditure for highways here, including outlays in support of toll highways.

Applicable Coding Options for this Expenditure Function

Direct Expenditure		Intergovernmental Expenditure	
E44	Current Operations	L44	To state governments
F44	Construction	M44	To local governments
G44	Land and Existing Structures		
K44	Equipment (Federal, states)		
J44	Assistance and Subsidies (Federal)		

EXPENDITURE FUNCTIONS

Code *45 **Toll Highways**

Sector: General Government

Definition: Maintenance, operation, repair, and construction of highways, roads, bridges, ferries, and tunnels operated on a fee or toll basis.

Includes: Turnpikes; toll roads, toll bridges, toll ferries (including docks and related terminals), toll tunnels, and all related activities and facilities such as snow and ice removal, highway police and fire protection units IF administered by the toll authority, lighting and light fixtures, design and engineering, garages and administrative buildings of toll authorities, operation of toll booths, drawspans, rest stops, and service areas by the toll authority itself.

Excludes: Debt service on toll facility debt (report interest payments at *Interest on General Debt,* code I89 and repayment of principal at *Long-term Debt Retired*, *Public Debt for Private Purposes*, code 39U); intergovernmental aid for toll facilities, including state aid for debt service on local highway debt (report at *Regular Highways*, code *44); police and fire protection services not provided by toll authority itself (report at *Police Protection*, code *62, or *Local Fire Protection*, code *24); free ferries (use code *44).

Examples:

- State governments – Oversight, maintenance, and toll operations of the New York State Thruway.
- Local governments – Highway and bridge operations of special district governments such as the Delaware River and Bay Authority and the Port Authority of New York and New Jersey.

Special Considerations:

1. This function includes direct expenditures only. Report any intergovernmental outlays at *Regular Highways*, code *44.
2. Report expenditures without deducting any related tolls or charges.
3. For leased facilities include government's expenditures and exclude those of the lessees.
4. Effective with 2005, this function includes related special district expenditures. Prior to 2005, special district government expenditure for toll highways was reported at *Regular Highways*, code *44.
5. This function applies only to state and local government expenditure. Report Federal Government expenditure, if any, at *Regular Highways*, code *44.

Applicable Coding Options for this Expenditure Function

Direct Expenditure		Intergovernmental Expenditure
E45	Current Operations	None
F45	Construction	
G45	Land and Existing Structures	
K45	Equipment (states)	

Code *50 **Housing and Community Development**

Sector: General Government

Definition: Construction, operation, and support of housing and redevelopment projects and other activities to promote or aid public and private housing and community development.

Includes:

Housing: Planning, construction, furnishing, and operation of public housing projects (generally for persons not adequately served by private sector); rent subsidies (e.g., "Section 8" assistance); housing and mortgage finance agencies; promotion of home ownership; assistance for repair and renovation of existing homes; and programs to encourage private sector housing production.

Community development: Urban renewal and slum clearance; redevelopment and rehabilitation of substandard or deteriorated facilities and areas; rural redevelopment; and revitalization of commercial areas.

Excludes:

Building inspection and enforcement of housing codes or standards (report at *Protective Inspection and Regulation, NEC*, code *66); direct loans to individuals, builders, landlords, or others (nonexpenditure, by definition); distribution of proceeds from mortgage revenue bonds (see Chapter 6 on debt); temporary shelters or housing for the homeless (report at *Public Welfare*, codes *77/*79); and construction and maintenance of military housing by the U.S. Department of Defense (report at *Federal National Defense and International Relations*, code *06).

Examples: Local government – The Minneapolis Public Housing Authority, classified as a special district government serving the Minneapolis, MN area.

Special Considerations:

1. The emphasis of this function is on physical plant (construction, provision, improvement, financing) and the operation of public housing facilities. Activities that directly aid homeowners or renters themselves (e.g., housing expense relief) generally fall under *Public Welfare* functions.

2. Report community development block grants clearly identified as being for other functions, such as sewers, streets, parks, etc., with those functions UNLESS they are an integral part of a housing project.

3. *Intergovernmental Expenditure to State Governments*, code L50, became applicable to local government (except school districts) effective with fiscal year 2006 survey. For 2005, it was a Federal code only. For 2004 and earlier, it applied to Federal and local governments.

Code *50 **Housing and Community Development**

Applicable Coding Options for this Expenditure Function

Direct Expenditure

E50	Current Operations
F50	Construction
G50	Land and Existing Structures
K50	Equipment (Federal, states)
J50	Assistance and Subsidies (Federal)

Intergovernmental Expenditure

L50	To state (Federal, locals effective FY 2006)
M50	To local governments

EXPENDITURE FUNCTIONS

Code *51 **Federal Farm Credit Program**

Sector: General Government

Definition: Federal Government programs for providing direct loans and loan guarantees to farmers, for the construction and operation of rural electric power and telephone systems, and for the regulation and examination of financial institutions constituting the Farm Credit System.

Includes: Comprises the activities of the Rural Housing and Community Development Service and parts of the Farm Service Agency of the U.S. Department of Agriculture (agricultural credit insurance, rural housing insurance, and related administration); Rural Utilities Service (comprised of the former Rural Electrification Administration and Rural Telephone Bank), which is engaged in rural electrification and telephone loans and guaranteed loans, and the Farm Credit Administration.

Excludes: Extensions of loans themselves and the value of guaranteed loans (nonexpenditures); rural water and wastewater disposal grants (report at *Other and Unallocable*, code *89); rural economic development loan programs (use code *89); Federal crop insurance and other farm income or price support programs (report at *Federal Farm Income Stabilization*, code *53).

Special Considerations:

1. There is no corresponding function in the Census Bureau's employment statistics.
2. By definition, there are no capital outlay objects applicable to this function.

Applicable Coding Options for this Expenditure Function

Direct Expenditure		Intergovernmental Expenditure	
E51	Current Operations	L51	To state governments
		M51	To local governments
J51	Assistance and Subsidies (Federal)		

Code *52 **Libraries**

Sector: General Government

Definition: Establishment and provision of libraries for use by the general public and the technical and financial support of privately-operated libraries.

Includes: Expenditures for general public libraries, community libraries, consolidated libraries, regional libraries, and their variously-named equivalents. Also includes library extension services (including bookmobiles), public library (special) districts, state library commissions and boards, and programs to promote, develop, and coordinate library services and facilities. Include here all aid for the construction or operation of other libraries, whether governmental or private. This function also includes the U.S. Library of Congress (including Copyright Office).

Excludes: Law libraries (report at *Judicial and Legal,* code *25); libraries operated by school systems – elementary, secondary, or higher education – primarily for the benefit of students and teachers (report at *Education,* codes *12/*18); specialized libraries which do not serve the general public, such as a medical library of a university hospital (report at function involved).

Examples: Federal Government – U. S. Library of Congress.

Special Consideration:
This function is not applicable for school district governments, even though some operate libraries (report at *Elementary and Secondary Education*, code *12).

Applicable Coding Options for this Expenditure Function

Direct Expenditure		Intergovernmental Expenditure	
E52	Current Operations	L52	To state governments
F52	Construction	M52	To local governments
G52	Land and Existing Structures		
K52	Equipment (Federal, states)		
J52	Assistance and Subsidies (Federal)		

Code *53 **Federal Farm Income Stabilization**

Sector: General Government

Definition: Federal Government programs to stabilize, support, and protect farm income and prices through commodity loans, purchases, payments, production limits, and crop insurance.

Includes: Comprises three activities of the U.S. Department of Agriculture: Commodity Credit Corporation (CCC), Farm Service Agency, and the purchase of commodities for child nutrition programs. The Farm Service Agency was created in 1994 and combined the former Agricultural Stabilization and Conservation Service (ASCS) and the Federal Crop Insurance Corporation (FCIC). The ASCS administered much of the CCC's commodities program as well as related land use programs designed for voluntary production adjustment, resource protection, and price and farm income stabilization. The FCIC sought to improve agricultural economic stability through a system of crop insurance covering losses from natural hazards. This activity is now handled by the Farm Service Agency.

Excludes: Extensions of loans themselves (nonexpenditures); sale or provision of agricultural commodities to other countries, such as Food for Progress and Food for Peace programs (report at *Federal National Defense and International Relations*, code *06); transfer of funds from the CCC to the Farm Service Agency (an intragovernmental transfer); conservation programs unrelated to price or income support; agricultural credit programs unrelated to farm income stabilization (report at *Federal Farm Credit Programs*, code *51).

Special Consideration:
There is no corresponding function in the Census Bureau's employment statistics.

Applicable Coding Options for this Expenditure Function

Direct Expenditure		Intergovernmental Expenditure	
E53	Current Operations	L53	To state governments
F53	Construction	M53	To local governments
G53	Land and Existing Structures		
K53	Equipment		
J53	Assistance and Subsidies		

Code *55 **State Fish and Game**

Sector: General Government

Definition: Conservation, improvement, development, and propagation of fish and game resources, as well as the regulation and enforcement of fish and game laws.

Includes: Expenditures for fish and game commissions or similarly titled agencies. This function includes the regulation and enforcement of laws relating to commercial or sport fishing and wildlife (including inspection of processing facilities) and activities of fish and game wardens (including those with arrest powers). This also covers the operation of fish hatcheries, stocking of lakes and streams, and the "seeding" of waterways for propagation. Include here expenditures on research studies and surveys to measure fish and wildlife populations, development and maintenance of wildlife management or hunting and fishing areas, management and protection of wildlife or fish species, and related public education programs.

Excludes: Water or soil conservation activities not directly related to fish and game (report such activities at *Natural Resources, Other*, code *59).

Special Considerations:

1. Effective 2005, this function was re-titled *State Fish and Game* to reflect its applicability to state governments only. Any local government expenditures for this activity should be reported at *Natural Resources, Other*, code *59.
2. For tabulation purposes, state expenditures at this function are summed at *Natural Resources, Other,* code *59, when state and local expenditure totals are developed.
3. There is no corresponding function in the Census Bureau's employment statistics.

Applicable Coding Options for this Expenditure Function

Direct Expenditure		Intergovernmental Expenditure	
E55	Current Operations	M55	To local governments
F55	Construction		
G55	Land and Existing Structures		
K55	Equipment		

EXPENDITURE FUNCTIONS

Code *56 **Federal and State Forestry**

Sector: General Government

Definition: Federal and state government expenditures for the conservation, development, management, and protection of forests and forest resources. This function includes regulation and inspection of forest products and industries, as well as the provision of assistance to private or local government owners of woodlands.

Includes: Cooperative forest management; forest crop land administration; promotion of the use and marketing of forest products; forest fire prevention, control, and suppression; state forest land management; detection and control of insects and tree diseases; operation and support of tree seedling nurseries; regulation and inspection of timber producers, forest product industries, and other wood processors; acquisition, development, and management of state forests; reforestation activities; and urban and community forestry.

Excludes: Forest-related activities of park agencies (report at *Parks and Recreation*, code *61); wildlife management unless an inseparable part of a forestry agency (report at *State Fish and Game*, code *55).

Special Considerations:

1. Effective 2005, this function was re-titled *Federal and State Forestry* to reflect its applicability to Federal and state governments only. Any local government expenditures for this activity are reported at *Parks and Recreation*, code *61, or at *Natural Resources, Other*, code *59.

2. For tabulation purposes, state expenditures at this function are summed at *Natural Resources, Other,* code *59, when state and local expenditure totals are developed.

3. There is no corresponding function in the Census Bureau's employment statistics. Employees, if any, are reported at *Natural Resources*, *Other*, code *59.

Applicable Coding Options for this Expenditure Function

Direct Expenditure

E56 Current Operations
F56 Construction
G56 Land and Existing Structures
K56 Equipment

J56 Assistance and Subsidies (Federal)

Intergovernmental Expenditure

L56 To state governments (Federal)
M56 To local governments

EXPENDITURE FUNCTIONS

Code *57 **Federal Soil, Water, and Electric Energy Resources**

Sector: General Government

Definition: Federal Government programs for the conservation, management, and development of soil and water resources; flood prevention and control; management of public lands; generation and distribution of electric power (including hydroelectric energy); and development of new or improved energy sources and technologies.

Includes: Comprises the activities of: U.S. Department of Energy (energy supply, research, and development; uranium supply and enrichment; strategic petroleum reserves; electric power marketing administrations; and Federal Energy Regulatory Commission); Tennessee Valley Authority (TVA); Army Corps of Engineers' Civil Works Program (flood control and prevention; beach erosion control; dam safety; and hydro power stations); Department of the Interior's Bureau of Reclamation (management and development of water for irrigation, public and industrial use, salinity control, dam safety, and flood prevention; and hydroelectric generation) and Bureau of Land Management (management of public lands and their resources); Department of Agriculture's Soil Conservation Service; and the Nuclear Regulatory Commission.

Excludes: Department of Energy atomic energy defense activities (report at *Federal National Defense and International Relations*, code *06) and nuclear waste disposal (report at *Other and Unallocable*, code *89); Army Corps of Engineers' navigation and harbors activities (report at *Sea and Inland Port Facilities*, code *87); Bureau of Land Management programs for managing minerals (report at *Federal Mineral Resources*, code *58) and payments-in-lieu-of-taxes (use code *89); Rural Utilities Services (formerly the Rural Electrification Administration) – report at *Federal Farm Credit Programs*, code *51.

Special Considerations:

1. Report state-local soil and water conservation activities at *Natural Resources, Other*, code *59.
2. There is no corresponding function in the Census Bureau's employment statistics. Employees, if any, are reported at *Natural Resources, Other*, code *59.
3. This function applies only to Federal Government financial statistics.

Applicable Coding Options for this Expenditure Function

Direct Expenditure

E57	Current Operations
F57	Construction
G57	Land and Existing Structures
K57	Equipment
J57	Assistance and Subsidies

Intergovernmental Expenditure

L57	To state governments
M57	To local governments

EXPENDITURE FUNCTIONS

Code *58 **Federal Mineral Resources**

Sector: General Government

Definition: Federal Government programs related to the exploration and production of oil, gas, and other minerals; reclamation of mines and abatement of the negative environmental effects of mining; minerals research, information, and analysis; and enforcement of mine safety laws and regulations.

Includes: Comprises the activities of: U.S. Department of the Interior's Minerals Management Service (overseeing offshore minerals development and distributing funds to states from receipts under the Mineral Leasing Act), Office of Surface Mining Reclamation and Enforcement (including abandoned mine reclamation), and Bureau of Mines, and the Department of Labor's Mine Safety and Health Administration.

Excludes: Department of Energy strategic petroleum reserves program (report at *Federal Soil, Water, and Electric Energy Resources,* code *57); mineral resource development on public lands managed by the Interior Department's Bureau of Land Management (use code *57).

Special Considerations:

1. Report state-local soil and water conservation activities at *Natural Resources, Other,* code *59.
2. There is no corresponding function in the Census Bureau's employment statistics. Employees, if any, are reported at *Natural Resources, Other,* code *59.
3. This function applies only to Federal Government financial statistics.

Applicable Coding Options for this Expenditure Function

Direct Expenditure		Intergovernmental Expenditure	
E58	Current Operations	L58	To state
F58	Construction	M58	To local governments
G58	Land and Existing Structures		
K58	Equipment		
J58	Assistance and Subsidies		

Code *59 **Natural Resources, Other**

Sector: General Government

Definition: Expenditures related to water resources, mineral resources, agriculture, and the regulation of industries which develop, utilize, or affect natural resources, as well as the regulation of agricultural products and establishments. Includes conservation, promotion, and development activities related to agriculture and natural resources (soil, water, energy, minerals, etc.). For state governments, expenditures in this function cover activities not reported in other *Natural Resources* functions (*Federal and State Forestry,* code *56, and *State Fish and Game,* code *55).

Includes: Irrigation; drainage; flood control; soil conservation and reclamation including prevention of soil erosion; surveying, development, and regulation of water resources; regulation of mineral resources and related industries including land reclamation; wetlands and watershed management and protection; geological surveying and mapping; purchase of land for open space and conservation programs; regulation of gas and oil drilling and production; dam and reservoir safety; public education programs related to the above.

Excludes: Hydroelectric power facilities (report Federal activities at *Federal Soil, Water, and Electrical Energy Resources*, code *57, and state and local activities at *Electric Power* utilities, code *92); improvement of waterways, construction and maintenance of canals, and operation of public water transportation facilities (report at *Sea and Inland Port Facilities,* code *87), inspection of public drinking water and activities related to air and water quality or pollution control (report at *Health*, code *32); advertising of state resources (report at *Other and Unallocable*, code *89); agricultural schools and land grant colleges (report at *Other Higher Education*, code *18) except for agricultural experiment stations and extension services (report at Natural Resources, Other).

Examples:

- Federal – National Oceanic and Atmospheric Administration (U.S. Department of Commerce) and the U.S. Geological Survey (U. S. Department of the Interior).
- State agricultural fairs and promotions.

Special Considerations:

1. Report all local government expenditure data related to *Natural Resources* here.
2. Effective 2005, this function includes expenditures for agricultural activities previously reported at function Other Agriculture, code *54. Thus it includes expenditures for agricultural associations, fairs, livestock; agricultural boards and advisory commissions; provision of agricultural extension services; agricultural experiment stations and other research activities; and all regulatory and licensing activities (for livestock, dairy, crops, and all other products). See Appendix 1.6.4 for detail.
3. By definition, this function and the applicable object codes do not apply to school districts.

Code *59 **Natural Resources, Other**

Applicable Coding Options for this Expenditure Function

Direct Expenditure		Intergovernmental Expenditure	
E59	Current Operations	L59	To state governments
F59	Construction	M59	To local governments
G59	Land and Existing Structures		
K59	Equipment (Federal, states)		
J59	Assistance and Subsidies (Federal)		

EXPENDITURE FUNCTIONS

Code *60 **Parking Facilities**

Sector: General Government

Definition: Provision, construction, maintenance, and operation of public parking facilities operated on a commercial basis.

Includes: Public parking lots and garages. Includes the purchase and maintenance of parking meters, on streets or in parking lots, and the collection of monies from them.

Excludes: Enforcement of parking regulations and laws are reported at *Police Protection,* code *62; parking facilities for exclusive use of government employees (report at *General Public Buildings*, code *31); parking areas connected to a specific type of facility, such as those for a public sports stadium (*report at Parks and Recreation, code *61*), public hospital (*Hospitals,* code *36), or public college (report at *Higher Education Auxiliary Enterprises,* code *16).

Special Considerations:

1. Effective 2005, this function applies to state governments. Prior to 2005, this function applied only to local government expenditures.
2. This function does not apply to Federal Government financial statistics.
3. For leased facilities, include government expenditures and exclude those of the lessees.
4. There is no corresponding function in the Census Bureau's employment statistics. Employees, if any, are reported at *Other and Unallocable*, code *89.

Applicable Coding Options for this Expenditure Function

Direct Expenditure		Intergovernmental Expenditure	
E60	Current Operations	L60	To state governments (locals)
F60	Construction	M60	To local governments
G60	Land and Existing Structures		
K60	Equipment (states)		

Code *61 **Parks and Recreation**

Sector: General Government

Definition: Provision and support of recreational and cultural-scientific facilities maintained for the benefit of residents and visitors.

Includes: Golf courses, playgrounds, tennis courts, public beaches, swimming pools, play fields, parks, camping areas, recreational piers and marinas, etc., including support of private facilities. Also includes expenditures for or support of galleries, museums, zoos, botanical gardens, memorials, auditoriums, stadiums, recreational centers, convention centers, and exhibition halls. Also includes expenditures in support of cultural activities such as community music, drama, and celebrations.

Excludes: Recreational and cultural-scientific activities and facilities operated as part of school systems (report at *Education*, codes *12 or *18); marinas operated for commerce rather than recreation (report at *Sea and Inland Port Facilities*, code *87).

Examples:

- Federal Government – Smithsonian Institution and National Foundation on the Arts and the Humanities.
- State governments – state park authorities such as the Virginia State Parks, a division of the Virginia Department of Conservation and Recreation.
- Local governments – Maryland National Capitol Parks and Planning Commission (a joint dependent agency of Montgomery County and Prince Georges County, Maryland).

Special Consideration:
Prior to 1977 data, this function applied solely to local governments. Effective with 1977 data, it was extended to cover Federal and state governments, whose activities were reported previously at *Forestry* [and Parks], code *56.

Applicable Coding Options for this Expenditure Function

Direct Expenditure

E61 Current Operations
F61 Construction
G61 Land and Existing Structures
K61 Equipment (Federal, states)

J61 Assistance and Subsidies (Federal)

Intergovernmental Expenditure

L61 To state governments
M61 To local governments

Code *62 **Police Protection**

Sector: General Government

Definition: Expenditures for general police, sheriff, state police, and other governmental departments that preserve law and order, protect persons and property from illegal acts, and work to prevent, control, investigate, and reduce crime.

Includes: All law enforcement activities of regular police departments, sheriff and constable offices, and state highway patrols, including the following: criminal investigation, forensic services, crime labs and testifying in court; buildings, including training academies, used exclusively for police purposes; coroners, medical examiners; payments for transporting criminals; police communications and radios services; criminal justice planning and general support activities if handled by law enforcement; the following activities IF handled by a police agency: motor vehicle inspection and regulation, liquor law enforcement, and traffic control (e.g., enforcing speed laws, directing traffic);

Excludes: Special police forces of nonpolice agencies such as park rangers or fish and game wardens (report at function of agency involved); law enforcement units of legal offices (report at *Judicial and Legal*, code *25); traffic control and engineering performed by non-police agencies (report at *Highways*, codes *44 and*45); police jails that hold people beyond arraignment (report at *Corrections,* codes *04 and *05); civil or bailiff activities of sheriff offices (use code *25).

Examples: Federal Government – Federal Bureau of Investigation (FBI), Drug Enforcement Administration (DEA), and Bureau of Alcohol, Tobacco, Firearms, and Explosives of the Department of Justice, as well as law enforcement activities of the Department of Homeland Security - the Border and Transportation Security Directorate (BTS), the U.S. Customs and Border Protection Service (CBP), the U.S. Immigration and Customs Enforcement (ICE), and the U.S. Secret Service.

Special Considerations:

1. Effective with 1988 data, the treatment of traffic control and engineering was clarified to include it here only if handled by a police agency.
2. Effective with 2005 data, this function applies to special district governments.

Applicable Coding Options for this Expenditure Function

Direct Expenditure

E62 Current Operations
F62 Construction
G62 Land and Existing Structures
K62 Equipment (Federal, states)

J62 Assistance and Subsidies (Federal)

Intergovernmental Expenditure

L62 To state governments
M62 To local governments

EXPENDITURE FUNCTIONS

Code *66 **Protective Inspection and Regulation, NEC**

Sector: General Government

Definition: Regulation and inspection of private establishments for the protection of the public or to prevent hazardous conditions NOT classified under another Census Bureau function, and the regulation of professional occupational licensing.

Includes: Inspection of plans, permits, construction, or installations related to buildings, housing, plumbing, electrical systems, gas, air conditioning, boilers, elevators, electric power plant sites, nuclear facilities, weights and measures, etc.; regulation of financial institutions, taxicabs, public service corporations, insurance companies, private utilities (telephone, electric, etc.), and other corporations; licensing, examination, and regulation of professional occupations, including health-related ones like doctors, nurses, etc.; inspection and regulation or working conditions and occupational hazards; motor vehicle inspection and weighing unless handled by a police agency; regulation and enforcement of liquor laws and sale of alcoholic beverages unless handled by a police department.

Excludes: Distinctive license revenue collection activities (report at *Financial Administration*, code *23); regulatory or inspection activities related to food establishments or to environmental health (report at *Health*, code *32); motor vehicle inspection, liquor law enforcement, and other regulatory type activities of police agencies (report at *Police Protection*, code *62); regulatory and inspection activities related to other major functions, such as fire inspections, health permits, water permits, and the like (report at function involved).

Special Considerations:

1. This function does not apply to Federal Government financial statistics.
2. There is no corresponding function in the Census Bureau's employment statistics. Employees, if any, are reported at *Other and Unallocable,* code *89.

Applicable Coding Options for this Expenditure Function

Direct Expenditure		Intergovernmental Expenditure	
E66	Current Operations	L66	To state governments (locals)
F66	Construction	M66	To local governments
G66	Land and Existing Structures		
K66	Equipment (states)		

Code *67 **Public Welfare - Federal Categorical Assistance Programs**

Sector: General Government

Definition: This function covers expenditures associated with only three Federal programs –
Supplemental Security Income (SSI)
Temporary Assistance for Needy Families (TANF)
Medical Assistance Program (Medicaid)

Includes: SSI payments to beneficiaries are made directly by the Federal government. Many state governments supplement the Federal benefits. States have the option of allowing the Federal Government to administer the supplemental payments and to include them with the Federal payment, even though they are funded by the state. In such cases, this function includes state-to-Federal intergovernmental payments on behalf of SSI recipients. These are coded at S67. If a state government pays the supplement portion directly to beneficiaries, the benefit payments are coded at J67.

Expenditures by state governments under the Medicaid program are included at this function only if they are in the form of intergovernmental payments to a government that operates a hospital (e.g. where a local government hospital is the vendor agency) or if they are payments to a local government to finance administration of a local Medicaid program (rare).

Report all “Disproportionate Share” and related Medicaid payments here. Local to state payments for this activity are reported at code L67 and state to local government shares are reported at M67.

This function includes cash payments by states to families under the TANF program, including related Emergency Assistance aid, and any intergovernmental payments to finance locally-administered TANF programs.

Excludes: Cash payments to needy persons under programs other than SSI and TANF (report at *Public Welfare - Other Cash Assistance*, code J68); intergovernmental payments to finance locally-administered assistance programs other than TANF and Medicaid (use code *68); state-only programs unrelated to SSI providing cash grants to aged, blind, or disabled (use code *68); payments directly to vendors of goods or services, including Medicaid reimbursements to private hospitals (report at *Public Welfare - Vendor Payments*, codes E74 and E75). Also excluded are direct administration costs for these categorical assistance programs, such as the fee charged by the Federal Government to administer supplement payments under SSI (report at *Public Welfare - Other*, code *79).

Examples: CFDA 93.778 Medical Assistance Program (Medicaid)
CFDA 93.558 Temporary Assistance for Needy Families (TANF)
CFDA 96.006 Supplemental Security Income

Code *67 **Public Welfare - Federal Categorical Assistance Programs**

Special Considerations:

1. Coding option J67 in this function represents the character "Assistance and Subsidies," for all levels of government. Prior to 2005, object code "E" was used for state and local governments to represent "Assistance and Subsidies" and object code "I" was used for the Federal Government to represent "Assistance and Subsidies."

2. In 1974, the U.S. Congress replaced the former Federally-funded, state-administered categorical assistance programs (Old Age Assistance, Aid to the Blind, and Aid to the Disabled) with the Supplemental Security Income Program (SSI). At the time, the Federal Government required that selected states supplement the new Federal SSI payments because the standard Federal payments were actually lower than what previously existed in some states. The Federal Government also allowed all states the option of paying additional, supplemental benefits. These two factors led to the existence of the supplemental payments described above.

3. Although administrative costs of the state governments are excluded here, the Census Bureau recognizes that it might be difficult to identify the fee charged by the Federal Government to administer any state supplements under SSI, and to separate that fee from the actual benefit payment portion coded at S67. Hence the latter might include small amounts of administrative costs.

4. Temporary Assistance to Needy Families (TANF) replaced the former Aid to Families with Dependent Children (AFDC) program in 1997.

5. Payments under the Federal Medicaid program and its variants (such as Medi-Cal in California) are difficult to classify and follow in the Census Bureau's statistics on government finance. Such payments are reported at this function only if they represent payments on the part of the government unit, to other governments units (such as state to local governments). The latter almost always involve a state payment to a public hospital operated by a local government.

6. There is no corresponding function in the Census Bureau's employment statistics. Employees, if any, are reported at *Public Welfare - Other*, code *79.

Applicable Coding Options for this Expenditure Function

Direct Expenditure		Intergovernmental Expenditure	
J67	Assistance and Subsidies	L67	To state governments
		M67	To local governments
		S67	To Federal Government (states, DC only)

EXPENDITURE FUNCTIONS

Code *68 **Public Welfare - Other Cash Assistance Programs**

Sector: General Government

Definition: Cash payments made directly to individuals contingent upon their need, other than those under Federal categorical assistance programs.

Includes: Poor relief; general relief; home relief; emergency relief; general assistance; refugee assistance; medical assistance, housing expense relief, energy assistance (e.g., Federal Low Income Home Energy Assistance Program, or LIHEAP), emergency assistance, etc. paid directly to individuals and not to vendors; other direct assistance to needy persons not covered by or eligible for Federal categorical assistance; payments to other governments in support of, or as reimbursement for costs of, these types of assistance programs.

Excludes: Administration of such programs (report at *Public Welfare - Other*, code *79); cash payments made under Federal categorical assistance programs (use *Public Welfare - Federal Categorical Assistance Programs*, code J67); payments made to vendors rather than the individuals benefited (report at *Public Welfare - Vendor Payments*, codes J74 and J75); bonus payments to veterans as a class and not contingent on their need (report at *Federal and State Veterans' Bonuses*, code J85 for state governments); noncash or in-kind contributions – e.g., milk, cheese, clothing, etc. (report purchases of such items at function of contributing agency).

Special Considerations:

1. This function does not apply to Federal Government financial statistics or to special district governments.
2. There is no corresponding function in the Census Bureau's employment statistics. Employees, if any, are reported at *Public Welfare - Other*, code *79.
3. Coding option J68 in this function represents the character "Assistance and Subsidies." Use of the "J" designation for state and local governments became effective in 2005.

Applicable Coding Options for this Expenditure Function

Direct Expenditure		Intergovernmental Expenditure	
J68	Assistance and Subsidies	M68	To local governments

Code *74 **Public Welfare - Vendor Payments for Medical Care**

Sector: General Government

Definition: Public welfare payments made directly to private vendors for medical assistance and hospital or health care, including Medicaid (Title XIX), plus mandatory state payments to the Federal Government to offset costs of prescription drugs under Medicare Part D. Payments to vendors or the Federal Government must be made on behalf of low-income or means tested beneficiaries, or other medically-qualified persons.

Includes: Payments for physician and other professional medical services, private hospital care, drugs and medicines, dental services, long-term health care (including hospices), home health care, dialysis treatment, medical appliances (e.g., prostheses), ambulatory care, laboratory services, eyeglasses and hearing aids, and so forth. Premiums paid to insurers for future medical costs of needy persons. Includes all direct payments to private hospitals or health care providers under Medicaid, general relief, public assistance, and any other Federal or state welfare program. Payments to the Federal Government, as mandated by the Medicare Modernization Act of 2003, for prescription drug coverage under Medicare part D.

Excludes: Direct payments to benefitted persons themselves (report at *Other Cash Assistance Payments,* code J68); administrative activities such as setting of provider fees and rates, establishing standards, etc. (report at *Public Welfare - Other*, code *79); intergovernmental payments to other public hospitals for medical assistance under public welfare programs (report payments from Medicaid funds at *Public Welfare - Federal Categorical Assistance Programs*, code *67, and payments from all other funds at *Public Welfare - Other*, code *79); intergovernmental payments for Disproportionate Share and similar transfers related to the Medicaid program (report at *Public Welfare - Federal Categorical Assistance Programs*, code *67); medical commodities, services, or other assistance provided through government's own hospitals or health agencies (report at *Health*, code *32, or *Hospitals*, code *36); premiums for health insurance coverage for a government's employees (report at function of paying agency or, if a government-wide payment, at *Other and Unallocable*, code *89).

Examples: State governments – Payments to the Federal Government under Medicare Part D.

Special Considerations:

1. Effective 2006, this function includes intergovernmental payments by state governments to offset Federal Medicare program expenditures for prescription drug coverage (often referred to as "phase-down" or "claw back" payments). The Medicare Modernization Act of 2003, Part D went into effect in 2006. Part D includes coverage for certain beneficiaries previously covered under Medicaid. State governments are required to pay for a portion of that coverage. Since these payments support Federal expenditures for vendors supplying prescription medicines, the Census Bureau decided to classify these payments at *Public Welfare - Vendor Payments for Medical Care* and assign code S74.

2. State payments to help finance local Medicaid operations are reported at *Public Welfare - Federal Categorical Assistance Programs*, code M67.

Code *74 **Public Welfare - Vendor Payments for Medical Care**

Special Considerations: (continued)

3. There is no corresponding function in the Census Bureau's employment statistics. Employees, if any, are reported at *Public Welfare* - *Other*, code *79.
4. This function does not apply to Federal Government financial statistics.

Applicable Coding Options for this Expenditure Function

Direct Expenditure		Intergovernmental Expenditure	
E74	Current Operations	S74	To Federal Government (states)

Code *75 **Public Welfare - Vendor Payments for Other Purposes**

Sector: General Government

Definition: Payments under public welfare programs made directly to private vendors (i.e., individuals or nongovernmental organizations furnishing goods and services) for services and commodities, other than medical, hospital, and health care, on behalf of low-income or other means-tested beneficiaries.

Includes: Payments to private vendors for: funeral or burial services, food, clothing, home heating fuel, winter energy assistance, and the like.

Excludes: Direct payments to the benefitted persons themselves (report at *Public Welfare - Other Cash Assistance Payments*, code J68); administrative activities related to payments (report at *Public Welfare - Other*, code *79); legal services and public defense including stipends to court-appointed attorneys (report at *Judicial and Legal*, code *25); reimbursements effected through tax credits for the benefited person (treat as a deduction to tax revenue); commodities or other services and assistance provided through a governments own agencies (report at function involved); rent subsidies to landlords including “Section 8” lower income housing assistance (report at *Housing and Community Development*, code *50).

Special Considerations:

1. This function does not apply to the Federal Government or to special or school district governments, by definition.
2. There is no corresponding function in the Census Bureau’s employment statistics. Employees, if any, are reported at *Public Welfare - Other*, code *79.

Applicable Coding Options for this Expenditure Function

Direct Expenditure		Intergovernmental Expenditure
E75	Current Operations	None

Code *77 **Public Welfare - Institutions**

Sector: General Government

Definition: Provision, construction, and maintenance of nursing homes and welfare institutions owned and operated by a government for the benefit of needy persons (contingent upon their financial or medical need), and veterans.

Includes: Public nursing homes; veterans' homes; soldiers' homes; orphanages; homes for the elderly or aged; indigent care institutions (nonhospitals).

Excludes: Hospitals and institutions for the handicapped, crippled, developmentally-disabled, veterans, etc. (report at *Hospitals*, code *36); institutions for the blind, deaf, or other impairments primarily for education or training (report at *Other Education*, code *21); provision of care of needy persons in private institutions or homes (report at *Public Welfare - Other*, code *79); support of privately-operated welfare facilities (use code *79); nursing homes directly associated with a public hospital (use code *36); distinctive educational services limited to needy persons – e.g., education of orphans in schools (report at appropriate *Education* function, codes *12 - *21).

Special Considerations:

1. Effective 2005, expenditures in this function are applicable to special district government.
2. Effective with 1988 data, the classification of nursing homes was clarified: henceforth, they were classed as a public welfare function (unless directly associated with a public hospital). Previously, they may have been reported at *Health*, code *32, *Hospitals*, code *36, or *Public Welfare - Institutions*.
3. This function includes direct expenditures only – report any intergovernmental outlays at *Public Welfare - Other,* code *79.
4. There is no corresponding function in the Census Bureau's employment statistics. Employees, if any, are reported at *Public Welfare - Other,* code *79.
5. This function does not apply to Federal Government financial statistics.

Applicable Coding Options for this Expenditure Function

Direct Expenditure		Intergovernmental Expenditure
E77	Current Operations	None
F77	Construction	
G77	Land and Existing Structures	
K77	Equipment (states)	

Code *79 **Public Welfare - Other**

Sector: General Government

Definition: All expenditures for welfare activities not classified elsewhere.

Includes: Administration of medical and cash assistance, general relief, vendor, and other programs; all intergovernmental payments for welfare other than for cash assistance programs or for Medicare Part D phase-down payments and all intergovernmental payments to public hospitals for medical assistance other than under the Medicaid program; regulation of private welfare activities; children services, such as foster care, adoption, day care, nonresidential shelters, and the like; social services for the physically disabled, such as transportation; low-income energy assistance and weatherization intergovernmental payments; temporary shelters and other services for the homeless; welfare-related community action programs;

Excludes: Benefits not contingent upon need, such as bonuses or payments to veterans as a class (use *Federal and State Veterans' Services*, code J85), pensions to former employees (use *Benefit Payments*, code X11), and the like; public defenders and indigent legal defense (report at *Judicial and Legal*, code *25); health and hospital care provided to needy or homeless through government's own hospitals or health agencies and payments to other governments for such purposes (report at appropriate *Health* or *Hospital* function); medical assistance paid directly to private hospitals (use *Public Welfare - Vendor Payments for Medical Care*, code E74) or to public hospitals under the Medicaid program (use *Public Welfare - Federal Categorical Assistance Programs*, code *67); activities funded by Federal Women, Infants, and Children (WIC) funds (report at *Health*, code *32).

Examples: Expenditures supported by Federal Social Services Block Grant (Title XX) funds.

Special Considerations:

1. Report public hospital expenditures from Medicaid funds at *Hospitals*, code *36.
2. Report all Federal Government public welfare activities here (except for *Public Welfare - Federal Categorical Assistance Programs* reported at code *67).
3. In corresponding public employment statistics, this function is used to report all employees and payroll for public welfare.

Applicable Coding Options for this Expenditure Function

Direct Expenditure

E79	Current Operations
F79	Construction
G79	Land and Existing Structures
K79	Equipment (Federal, states)
J79	Assistance and Subsidies (Federal)

Intergovernmental Expenditure

L79	To state governments
M79	To local governments

EXPENDITURE FUNCTIONS

Code *80 **Sewerage**

Sector: General Government

Definition: Provision, maintenance, and operation of sanitary and storm sewer systems and sewage disposal and treatment facilities, as well as all intergovernmental payments for such activities.

Includes: Expenditures for the construction and maintenance of sanitary and storm sewers and for sewage disposal, including the following: **c**onstruction and maintenance of sanitary sewer lines and related lift and pump stations; sewer cleaning and flushing; sewage treatment and water pollution control plants; storm drains that are not connected with highway projects; systems for the collection and disposal of storm runoff;

Excludes: Storm drains that are an integral part of highway projects (report at either *Regular Highways*, code *44, or *Toll Highways*, code *45); drainage for agricultural or other non-street purposes (report Federal activities at *Federal Soil, Water, and Electric Energy Resources*, code *57, and report state-local activities at *Natural Resources, Other*, code *59).

Examples:

- State governments – Narragansett Bay Water Quality Management, Massachusetts Water Resources Authority.
- Local governments – Buffalo Sewer Authority (a municipal sewerage department), as well as special district water and sewer districts.

Special Considerations:

1. For combined water supply and sewer systems, include segregable amounts related to sewage collection and disposal here and report *Water Supply* activities at code *91.
2. This function does not apply to Federal Government financial statistics. Report grants to state and local governments for wastewater treatment plant construction at *Other and Unallocable*, code *89.

Applicable Coding Options for this Expenditure Function

Direct Expenditure		Intergovernmental Expenditure	
E80	Current Operations	L80	To state governments (locals)
F80	Construction	M80	To local governments
G80	Land and Existing Structures		
K80	Equipment (states)		

EXPENDITURE FUNCTIONS

Code *81 **Solid Waste Management**

Sector: General Government

Definition: Collection, removal, and disposal of garbage, refuse, hazardous, and other solid wastes and the cleaning of streets, alleys, and sidewalks.

Includes: Garbage collection; sanitary landfills; non-nuclear hazardous waste disposal sites; incinerators; pyrolysis facilities; cleanup of toxic chemical spills and dumps; collection and disposal of abandoned vehicles; resource recovery authorities, including those which co-generate electricity or gas as a by-product; recycling centers; cleaning and washing of streets; and collection and disposal of street debris and trash.

Excludes: Distinctive amounts for snow and ice removal by a sanitation agency (report at *Highways*, codes *44 and *45); nuclear waste disposal (report at *Other and Unallocable*, code *89).

Examples: Local governments – Expenditure for landfills and recycling centers, such as the Prince William County Sanitary Landfill, a dependent agency of Prince William County, Virginia.

Special Considerations:

1. Effective with 1988 data, this category was expanded to include the activities of Federal and state governments, formerly classed at *Other and Unallocable*, code *89, and renamed from "Sanitation other than Sewerage."
2. The rationale for including at this function waste or resource recovery facilities that co-generate gas or electricity is that their primary purpose is disposing of wastes.

Applicable Coding Options for this Expenditure Function

Direct Expenditure		Intergovernmental Expenditure	
E81	Current Operations	L81	To state governments
F81	Construction	M81	To local governments
G81	Land and Existing Structures		
K81	Equipment (Federal, states)		

Code *85 **Federal and State Veterans' Services**

Sector: General Government

Definition: Administration of state veterans bonus payments, as well as other Federal and state veterans services NOT classifiable under *Public Welfare, Education, Health, Hospitals, Social Insurance Administration,* or any other major function.

Includes: Veterans' information and guidance services; claims representation; assistance in obtaining Federal and state benefits or rights to which they are entitled; general veterans outreach services; financial grants (other than bonuses) not contingent on need; certification of training and education programs for participation in Federal "GI bills" programs; administration of VA home loans program; administration of Federal veterans life insurance programs.

Excludes: Services that can be classified under other major functions, such as: veterans hospitals (report at *Hospitals,* codes *36/*37), veterans or soldiers retirement homes (report at *Public Welfare*, codes *77/*79), tuition assistance and scholarships (use *Education* codes *19/*20), and veterans health care (report at *Health*, codes *28/*32); grants for home improvements or acquisition (report at *Housing and Community Development*, code *50); loans for purchase or improvement of farms and homes (nonexpenditures); state distribution of Federal aid for veterans' education to local governments (report at *Other and Unallocable*, code *89); agency transactions to transmit Federal unemployment benefits to veterans or Federal education funds to private schools and individual veterans (nonexpenditures); state-local defense activities like national guard, civil air patrol, militia, etc. (use code *89).

Special Considerations:

1. This function applies only to Federal and state government expenditures. Report any related local expenditures at *Other and Unallocable,* code *89.
2. This function includes direct expenditure only. Report intergovernmental outlays at *Other and Unallocable*, code *89.
3. Report U.S. Department of Veterans Affairs payments for veterans' compensation and pensions at code J85. Prior to 2005, these were classified at I85.
4. Report state cash bonus payments to veterans' or their survivors at code J85. Prior to 2005, these were reported at E84.
5. There is no corresponding function in the Census Bureau's employment statistics.

Applicable Coding Options for this Expenditure Function

Direct Expenditure		Intergovernmental Expenditure
E85	Current Operations	None
F85	Construction	
G85	Land and Existing Structures	
K85	Equipment	
J85	Assistance and Subsidies	

Code *87 **Sea and Inland Port Facilities**

Sector: General Government

Definition: Provision, construction, operation, maintenance, and support of public waterways and harbors, docks, wharves, and related marine terminal facilities; and the regulation of the water transportation industry.

Includes: Commercial port facilities, canals, harbors, and other public waterways; dredging of same; public docks, piers, wharves, warehouses, cranes, and associated terminal facilities; regulation and inspection of the commercial water transportation industry.

Excludes: Recreational types of docks and marine facilities – e.g., public marinas devoted to pleasure boaters (report at *Parks and Recreation*, code *61); terminals and dock facilities for public ferries (if toll, report expenditures at *Toll Highways*, code *45, else report expenditures and any employment at *Regular Highways*, code *44).

Examples:
- Federal Government – includes waterways navigation activities of the Army Corps of Engineers and most of the Maritime Administration (Department of Transportation).
- State governments – Pennsylvania Port Authority or the Port of New Orleans (Louisiana), which are both dependent agencies of their respective state governments.
- Local governments – San Diego Unified Port District or the Port of Seattle (Washington).

Special Considerations:
1. This category is devoted to commercial water transportation and terminals rather than the provision of water-related activities and facilities for pleasure or recreation.
2. For leased facilities include only government's expenditures and employees and exclude the lessees' expenditures and contractual employment.
3. For the Federal Government, this function included the former Panama Canal Commission in the historical time series.
4. This function was formerly titled "Water Transport and Terminals."

Applicable Coding Options for this Expenditure Function

Direct Expenditure

E87 Current Operations
F87 Construction
G87 Land and Existing Structures
K87 Equipment (Federal, states)

J87 Assistance and Subsidies (Federal)

Intergovernmental Expenditure

L87 To state governments
M87 To local governments

Code *89 **Other and Unallocable**

Sector: General Government

Definition: Activities not applicable to other general functions, or multi-functional general sector activities that cannot be separated into specific functions.

Includes: The following types of expenditure activity: State-local military activities (militia, National Guard, armories, civil defense, etc.); lump-sum contributions for employee benefits (retirement, unemployment and workers' compensation, health and life insurances, etc.) other than transfers to own insurance trusts; premiums for government-wide fire, auto, liability, and other such insurances; judgments and compensation for injury to persons or property; recorded losses on the sale of investments (except those assigned to a specific insurance trust category); central service agencies (e.g., public works, motor pools, communications) other than financial administration and government-wide executive services; administration of multi-functional agencies; purchase of computer equipment for government-wide use; general economic development promotional activities; voter registration and elections; job training and employment programs (e.g., WIA); programs for senior citizens not based on need; crime victim compensation; geo-mapping services of a government agency; refund of receipts reported as revenue in prior fiscal years (other than taxes – see Chapter 4); nuclear waste disposal; activities for which a specific function is not applicable to that type of government (although this is of limited use subsequent to the 2005 redesign of the government finance statistics program), e.g. Federal and state government fire protection.

Excludes: State aid payments unrestricted in use or purpose (report at *General Local Government Support*, code *30); refund of taxes reported as revenue in prior fiscal years (deduct from this year's tax revenue); lump-sum contributions for employee benefits to insurance trusts administered by same government (interfund transfers); comprehensive planning and zoning (report at *Central Staff Services*, code *29).

Examples: Federal Government – report all disaster assistance spending, intergovernmental and direct, at this code.

Special Considerations:

1. In publications or special tabulations, the phrase "Other and Unallocable" often is used to represent amounts for functions not shown separately in a specific table. Thus it could include some specific functions in addition to this general function of "Other and Unallocable."
2. Intergovernmental expenditure to the Federal Government (object code S) is valid for state governments and Washington, DC only. Prior to 2005, this object was valid for other large local governments.

Code *89 **Other and Unallocable**

Applicable Coding Options for this Expenditure Function

Direct Expenditure

E89	Current Operations
F89	Construction
G89	Land and Existing Structures
K89	Equipment (Federal, states)
J89	Assistance and Subsidies (Federal)

Intergovernmental Expenditure

L89	To state governments
M89	To local governments
S89	To Federal (states)

Code I89 **Interest on General Debt**

Sector: General Government

Definition: Amounts paid for use of borrowed monies, except those on utility debt, paid by all funds of the government.

Includes: Interest on both short- and long-term debt for non-utility purposes, whether general obligation or nonguaranteed, including public debt for private purposes and debt to cover deficits of unemployment compensation or other insurance trust funds.

Excludes: Interest on debt for utilities (report at *Utilities* interest codes, I91 - I94); premiums paid on debt retired (report at *Other and Unallocable*, code E89, for general debt and at *Utilities* codes E91 - E94, for utility debt); Federal Government interest payments on own securities held by its insurance trusts (nonexpenditures).

Special Considerations:

1. Make no adjustment to interest expenditure for premiums or discounts on debt issued or retired.
2. Due to the difficulty of tracing such payments, do not deduct from interest expenditure any state or local government interest payments on own securities held by their employee retirement funds.
3. Deduct from interest expenditure for the year in which they were received for the following amounts: funds included in the proceeds from debt issues to cover interest accrued on debt prior to its sale.
4. The distinction between general interest and utility interest is determined by the purpose of the outstanding debt which generated the interest payment.
5. There is no corresponding function in the Census Bureau's employment statistics.

Applicable Coding Options for this Expenditure Function

Direct Expenditure		Intergovernmental Expenditure
I89	Interest on General Debt	None

Code *90 **Liquor Stores**
page 1 of 2

Sector: Liquor Stores

Definition: Establishment and operation of alcoholic beverage distribution facilities and retail outlets owned and operated by state and selected local governments.

Includes: Activities associated with both retail and wholesale government control of alcoholic beverages. The following are often referenced – alcoholic beverage monopolies, ABC stores, liquor control boards, and state liquor stores.

Excludes: Law enforcement (report at *Police Protection*, code *62, if handled by a regular police force), licensing (report at *Financial Administration*, code *23), and regulation of private liquor outlets carried out in conjunction with liquor store operations (report at *Protective Inspection and Regulation, NEC*, code *66); collection of liquor taxes and licenses (use code *23); transfer of profits or surplus earnings to parent government (an internal transfer); state distribution of earnings to local governments (if undesignated in purpose report at *General Local Government Support*, code *30, else report at function involved).

Examples: State governments – West Virginia Alcohol Beverage Control Administration – controlling the wholesale distribution of alcoholic beverages in that state.

Special Considerations:

1. This function does not apply to Federal Government financial statistics.
2. Expenditures related only to amounts for purchase of goods for resale and for provision and operation of stores, warehouses, and related facilities (including administration and capital improvements).
3. Report expenditures without deducting cost of goods sold or operating expenses.
4. See Chapter 10 for discussion of special exhibit codes and derived statistics for liquor stores.
5. Includes direct expenditures only – report any related intergovernmental outlays at *Other and Unallocable*, code *89.
6. This function applies only to state governments in Census Bureau statistics on public employment.

Applicable Coding Options for this Expenditure Function

Direct Expenditure		Intergovernmental Expenditure
E90	Current Operations	None
F90	Construction	
G90	Land and Existing Structures	
K90	Equipment (states)	

Code *90 **Liquor Stores**
page 2 of 2

Sector: Liquor Stores

Exhibit Codes and Derived Statistics Related to Liquor Stores Expenditure (Summarized – see Chapter 10 for detail)

Liquor Stores Exhibit and Derived Statistics: Net Income Statement Perspective

<u>Code and Description</u>

Liquor Store Income and Expense Statistics:

Z42	Cost of Goods Sold
Z43	Operating Expenses
Z45	Nonoperating Expenses

Liquor Stores Exhibit and Derived Statistics: Contributions to General Fund Perspective

Code and Description		Formula, If Calculated
Z46	Transfers to General Fund (Gross)	
Z47	Expenditure for Licensing and Law Enforcement	
---	Net Contributions to General Funds	+Z46 + Z47 - Z48

Special Consideration:
These are liquor stores codes related to expenses only. See Chapter 4 for statistics related to receipts, and Chapter 10 for the full array of exhibit and derived statistical codes for the liquor stores sector.

Code *91 **Water Supply**

Sector: Utilities

Definition: Operation, maintenance, and construction of public water supply systems, whether for distribution of water to the general public or to other public or private utilities. This function covers government water supply activities for residential, commercial, and industrial water usage.

Includes: Dams and reservoirs expressly for water supply; purification and filtration plants, including desalinization plants; pumping stations; aqueducts and transmission systems; water storage tanks; purchase of water for resale; and distribution lines and meters.

Excludes: Contributions to parent government; purchase of water for other than resale and provision of water to parent government (report, where identifiable, at function involved); facilities owned but leased to other governments or persons without financial oversight (report at *Miscellaneous Commercial Activities*, code *03); depreciation of assets; activities not directly related to utility operations, such as administration of utility debt or investments (report at *Financial Administration*, code *23) and payments-in-lieu-of-taxes; benefits paid to utility employees by employee retirement systems (use *Benefit Payments*, code X11); acquisition and distribution of water for irrigation (report at *Natural Resources, Other*, code *59).

Special Considerations:

1. This function does not apply to Federal Government financial statistics.
2. Relates only to systems owned and operated by a government or managed by private contractor when government maintains day-to-day financial oversight (e.g., by directly paying utility employees).
3. For combined water-sewer systems, include segregable amounts related to water supply here (report *Sewerage* activities at code *80).
4. Intergovernmental expenditure codes were added, effective with 1988 data, for users who seek to identify certain utility transactions already classified as general intergovernmental expenditure. An example would be state grants for local purification plants. In tabulations, estimates, and publications, however, these amounts continue to be treated as general expenditures despite their utility suffix. Utility expenditures continue to exclude them and to represent direct expenditure only.

Applicable Coding Options for this Expenditure Function

Direct Expenditure

E91	Current Operations
F91	Construction
G91	Land and Existing Structures
K91	Equipment (states)
I91	Interest on water utility debt

Intergovernmental Expenditure

L91	To state governments (locals)
M91	To local governments

Code *92 **Electric Power**

Sector: Utilities

Definition: Operation, maintenance, and construction of public electric power systems, including production, acquisition, and distribution of electricity to general public or to other public or private utilities. This function covers government electric power activities for residential, commercial, and industrial electrical usage.

Includes: Generating stations; power plants; transmission lines; switching stations; pollution abatement equipment; purchase of electricity for resale; distribution lines and meters.

Excludes: Contributions to parent government; purchase of electricity other than for resale and provision of electricity to parent government, such as for street lights (report, where identifiable, at function involved); facilities owned but leased to other governments or persons without financial oversight (report at *Miscellaneous Commercial Activities*, code *03); depreciation of assets; activities not directly related to utility operations, such as administration of utility debt or investments (report at *Financial Administration*, code *23) and payments-in-lieu-of-taxes; benefits paid to utility employees by employee retirement systems (use code X11); waste or resource recovery facilities which co-generate electricity as a by-product (report at *Solid Waste Management*, code *81).

Examples: State governments – Power Authority of the State of New York, which operates the hydroelectric generating facility in Niagara County, New York.

Special Considerations:

1. This function does not apply to Federal Government financial statistics.
2. Relates only to systems owned and operated by a government or managed by private contractor when government maintains day-to-day financial oversight (e.g., by directly paying utility employees).
3. Intergovernmental expenditure codes were added, effective with 1988 data, for users who seek to identify certain utility transactions already classed as general intergovernmental expenditure, such as support of joint power projects (see Section 5.3.2.2). In publications, however, these amounts will continue to be treated as general expenditures despite their utility suffix. Utility expenditures will continue to exclude them and to represent direct outlays only.

Applicable Coding Options for this Expenditure Function

Direct Expenditure		Intergovernmental Expenditure	
E92	Current Operations	L92	To state governments (locals)
F92	Construction	M92	To local governments
G92	Land and Existing Structures		
K92	Equipment (states)		
I92	Interest on electric utility debt		

Code *93 **Gas Supply**

Sector: Utilities

Definition: Operation, maintenance, and construction of public natural gas supply systems, including production, acquisition, and distribution of gas to general public or to other public or private utilities. This function covers government gas supply activities for residential, commercial, and industrial gas usage.

Includes: Public gas works; liquefaction equipment; pumping stations; gas mains; service lines and meters; purchase of natural gas for resale.

Excludes: Contributions to parent government; purchase of gas other than for resale and provision of gas to parent government, such as for incinerators (report, where identifiable, at function involved); facilities owned but leased to other governments or persons without financial oversight (report at *Miscellaneous Commercial Activities*, code *03); depreciation of assets; activities not directly related to utility operations, such as administration of utility debt or investments (report at *Financial Administration*, code *23) and payments-in-lieu-of-taxes; benefits paid to utility employees by employee retirement systems (use *Benefit Payments*, code X11); waste or resource recovery facilities which co-generate gas as a by-product (report at *Solid Waste Management*, code *81).

Examples: Local governments – Southeast Alabama Natural Gas District (classified as a special district government).

Special Considerations:

1. This function does not apply to Federal Government financial statistics.
2. Relates only to systems owned and operated by a government or managed by private contractor when government maintains day-to-day financial oversight (e.g., by directly paying utility employees).
3. Intergovernmental expenditure codes were added, effective with 1988 data, for users who seek to identify certain utility transactions already classed as general intergovernmental expenditure (examples being rare for gas supply systems). In publications, however, these amounts continue to be treated as general expenditures despite their utility suffix. Utility expenditures continue to exclude them and to represent direct outlays only.

Applicable Coding Options for this Expenditure Function

Direct Expenditure		Intergovernmental Expenditure	
E93	Current Operations	L93	To state governments (locals)
F93	Construction	M93	To local governments
G93	Land and Existing Structures		
K93	Equipment (states)		
I93	Interest on gas utility debt		

EXPENDITURE FUNCTIONS

Code *94 **Public Mass Transit Systems**

Sector: Utilities

Definition: Operation, maintenance, and construction of public mass transit systems, including subways, surface rails, and buses.

Includes: Rapid transit; subways, buses, surface rail, and street railroad systems; commuter rail lines; trolleys and light rail; related stations, tracks, depots, and rail yards; acquisition of right-of-ways; transit police employed directly by utility; subsidies to public mass transit systems (but not private ones).

Excludes: Systems solely to transport students (report at *Elementary and Secondary Education*, code *12); ferries (if free, report at *Regular Highways*, code *44, else report at *Toll Highways*, code *45); systems exclusively for handicapped or senior citizens (report at *Public Welfare - Other,* code *79); subsidies for private mass transit systems (report at *Other and Unallocable,* code *89); contributions to own system (an interfund or intragovernmental transfer); systems owned but operated under private contract without financial oversight (report at *Miscellaneous Commercial Activities*, code *03); depreciation of assets; activities not directly related to utility operation, such as administration of utility debt (report at *Financial Administration*, code *23) and payments-in-lieu-of-taxes; benefits paid to utility employees by employee retirement systems (use *Benefit Payments*, code X11).

Examples:

- State governments – Long Island Railroad, a component of the Metropolitan Transportation Authority, which is a dependent agency of New York State.
- Local governments – Washington Metropolitan Area Transit Authority, a special district government.

Special Considerations:

1. This function does not apply to Federal Government financial statistics.
2. Relates only to systems owned and operated by a government or managed by private contractor when government maintains day-to-day financial oversight (e.g., by directly paying utility employees).
3. Intergovernmental expenditure codes were added, effective with 1988 data, for users who seek to identify certain utility transactions already classed as general intergovernmental expenditure, such as subsidies to public transit systems (previously reported at Transit Subsidies, code *47). In publications, however, these amounts continue to be treated as general expenditures despite their utility suffix. Utility expenditures continue to exclude them and to represent direct outlays only.

Applicable Coding Options for this Expenditure Function

Direct Expenditure

E94	Current Operations
F94	Construction
G94	Land and Existing Structures
K94	Equipment (states)
I94	Interest on transit utility debt

Intergovernmental Expenditure

L94	To state governments (locals)
M94	To local governments

EXPENDITURE FUNCTIONS

Exhibit Code Z00 **Total Salaries and Wages**

Sector: Not Applicable

Definition: Total expenditure during fiscal year for salaries and wages, covering all functions and activities of the government and its dependent agencies. Includes the general government, liquor stores, and utilities sectors.

Includes: Salaries and wages consist of gross amounts paid for compensation of own officers and employees (prior to deductions for withheld taxes, retirement contributions, charges for subsistence, or other purposes), including both current operations expenditure and capital outlay expenditure.

Excludes: This category excludes employer contributions for any type of employee fringe benefit and the value of subsistence, quarters, or other payments-in-kind to military or other public employees.

Special Consideration:
This exhibit code applies to all levels and types of governments.

Applicable Coding Options for this Expenditure Function

Direct Expenditure		Intergovernmental Expenditure
Z00	Federal, states, all locals	Not applicable

Exhibit Code CGE **Tobacco Lawsuit Settlement – Expenditure of Proceeds**

Sector: General

Definition: Total amount expended during the fiscal year, from proceeds of settlement between the state governments' and the major tobacco manufacturers, pursuant to Federal judicial proceedings.

Includes: Total amount expended, regardless of functional activity and regardless of which fiscal year the monies were received.

Special Considerations:

1. All amounts reported here also are reported in their proper functional categories and under appropriate object codes.
2. This exhibit code applies only to state governments and to Washington, DC, by definition. The Census Bureau does not track the total amount of lawsuit proceeds expended at the local government level, but rather reports such expenditure by function only.
3. The related revenue exhibit is *Tobacco Lawsuit Settlement – Receipts,* code CGR. See Chapter 4 for details.

Applicable Coding Options for this Expenditure Function

Direct Expenditure		Intergovernmental Expenditure
CGE	States and Washington, DC only	Not applicable

EXPENDITURE FUNCTIONS

Exhibit Code V98 **Total Capital Outlay**

Sector: All Sectors

Definition: Total amount expended during the fiscal year for all capital outlay.

Includes: Capital outlay expenditure for all functions and all sectors.

Special Considerations:

1. This code is used primarily for internal Census Bureau processing purposes, but can appear in various public use files available in electronic format.
2. This is a calculated statistic. It represents the sum of all expenditure amounts coded with object codes "F" (Construction) + "G" (Capital Outlay Other than Construction).
3. This exhibit code applies to local governments only, all types.

Applicable Coding Options for this Expenditure Function

Direct Expenditure		Intergovernmental Expenditure
V98	Locals (all types)	Not applicable

Codes X11 and X12 **Public Employee Retirement Systems**

page 1 of 3

Sector: Social Insurance Trusts

Definition: Distribution of cash benefits to, or withdrawals by, eligible persons under government-administered employee retirement systems covering public employees.

Includes:

- Benefits: Cash payments to, or on behalf of, participants for retirement benefits and annuities, death and disability benefits, life and disability insurance on behalf of retirees, pre-retirement death benefit premiums, benefits due on termination of employment, survivors benefits, and other benefits as allowed.
- Withdrawals: Cash withdrawals of employees, former employees, or their survivors as return of contributions made during employment, plus any interest on such amounts. Includes transfers of investment holdings and reimbursements for benefits paid when another pension fund assumes responsibility for paying retirement benefits.
- Other Payments: Exhibit-only codes for expenditure statistics. See Chapter 8 for these codes.

Excludes:

- Expenditures for administering retirement system or managing its assets (report at *Financial Administration*, code 23); recorded losses on investments (also deduct from *Total Earnings on Investments*, code X08); other costs or payments not representing benefits or withdrawals (except purchase of investments).
- Payments to own retirement system (interfund transfer).
- "Pay-as-you-go" pensions and other direct payments to retired employees from current appropriations (report as current operations expenditure of function involved).
- Payments to retirement plans administered by another government, such as Federal Social Security and local payments to state-administered retirement systems; payments of premiums for annuity policies from private insurance carriers; contributions to pension plans administered by private companies or by public employee associations. Report these as current operations expenditure of function involved.
- Unrealized loss in market value of securities (deduct from total earnings on investments – see Chapter 8).
- Direct pensions paid out of Federal Military Retirement Fund (use *Federal National Defense and International Relations*, code *06).
- Other – purchase of investments, loans to members, and amortization of premiums on purchase of investments (all of which are nonexpenditures).

Examples:
- State Government system for state employees – California Public Employees Retirement System, or CALPERS.
- State Government system for local employees – Kentucky County Employees Retirement System.
- Local Government system for general local workers – Los Angeles County Employee retirement Association.
- Local Government system for specialized employees – Fulton County (GA) School Pension Board.

Special Considerations:
1. These data are collected by a separate survey of state and local government-administered public employee retirement systems.
2. Payments and withdrawals must be those of a dependent agency classified as a public employee retirement system for purposes of Census Bureau statistics. See Chapter 8 for details.
3. Report state aid to local retirement systems that does not represent employer contributions at *Other and Unallocable*, code E89. Since the local systems treat this aid as an insurance trust revenue, recording the state payment as an intergovernmental expenditure would distort the intergovernmental flow for that state.
4. For Federal Government, this includes Civil Service, Foreign Service Retirement and Disability Funds, and Federal Employees' Retirement System (FERS) defined benefit plan portion only. There are other Federal-only plans, such as for Congressional, Federal Judiciary and classified employees (CIA). These are included if data are available.
5. Report employment data for administering these systems at *Financial Administration*, code 23.

Applicable Coding Options for this Expenditure Function

Direct Expenditure	Intergovernmental Expenditure
X11 Benefit Payments (Federal, states, and locals)	None
X12 Withdrawals (Federal, states, and locals)	

Note on Coverage: X11 and X12 apply to all levels of government (Federal, state, and local) and to all types of local government (county, municipal, township, special district, and school district), as long as the governments administer a public employee retirement system, defined by the Census Bureau as a dependent agency of the administering government. See Chapter 8 for additional information.

Exhibit Codes Related to Public Employee Retirement Systems Expenditure
(Summarized – see Chapter 8 for detail)

Code and Description

Z13 Retirement Benefits
Z14 Disability Benefits
Z15 Survivor Benefits
Z16 Other Benefits
Z93 Administrative Expenses

Special Consideration for Exhibit Codes:

Code Z90 is found on some survey questionnaires, but is not part of the classification system. It is a cell for data collection purposes only. Any amounts reported are evaluated and moved to an appropriate, valid classification code.

EXPENDITURE FUNCTIONS

Codes Y05 and Y06 **Unemployment Compensation Systems**

Sector: Social Insurance Trusts

Definition: Distribution of cash benefits to eligible persons under the cooperative Federal-state unemployment compensation insurance programs.

Includes:

Regular Benefits: Covers basic payments to individuals temporarily out of work and seeking employment (funded by state payroll taxes) and to former Federal civilian and military employees (funded by Federal agencies that employed them).

Extended and Special Benefits: Payments for extended unemployment benefits in states with high rates of unemployment (funded equally by Federal and state payroll taxes), benefits under special programs (such as trade adjustment assistance and disaster assistance funded by Federal appropriations), and benefits to special groups of former workers, such as railroad employees (funded by Federal tax on railroad payrolls).

Excludes:

Administration of unemployment insurance programs (report at *Social Insurance Administration*, code *22), employment services provided to the unemployed (such as job training, placement, and guidance – use code *22),

Government payments as employer contributions on behalf of its employees to unemployment insurance system of another government (report as current operation of function involved) or to one it administers (interfund transfer); distinctive health or disability insurance programs carried out in conjunction with unemployment programs (report at *Other Insurance Trusts,* code Y5*).

Special Considerations:

1. This function applies only to statistics for the Federal Government, state governments, and to Washington, DC.
2. Data for this function are obtained from the Employment and Training Administration of the U.S. Department of Labor.
3. There is no corresponding function in the Census Bureau's employment statistics. Report employment data for administering these programs at *Social Insurance Administration,* code *22.

Applicable Coding Options for this Expenditure Function

Direct Expenditure Only

Y05 Benefit Payments (Federal and state governments, plus Washington, DC)

Y06 Extended and Special Benefits (Federal and state governments, plus Washington, DC)

Note on Coverage: Y05 and Y06apply only to the Federal and state governments, plus to Washington, DC. These codes do not apply to any other local governments. See Chapter 9 for additional information.

Sector: Social Insurance Trusts

Definition: Distribution of cash benefits to eligible persons under state-administered plans for compulsory accident and injury insurance of workers.

Includes:

- Benefits: Claims paid directly to, or on behalf of, injured beneficiaries for compensation of wages lost, medical care, rehabilitation, funeral expenses, and other eligible benefits. Includes payments made from regular workers' compensation funds as well as from subsequent injury funds, second injury funds, and the like.

- Other Payments: Exhibit-only codes for expenditure statistics.

Excludes:

- Administrative Expenses:
  - Crime victim compensation benefits administered by a workers' compensation agency (report at *Other and Unallocable*, code *89).
  - Cost for adjudication of claims (also report at *Financial Administration*, code *23).
  - Purchase of investments.
  - Exclude administrative costs from benefits and report them at both codes Y15 and *23.
  - Exclude recorded losses on investments from benefits (also deduct from *Earnings on Investments*, code Y12).

- Governments' Own Costs:
  - State government payments to system on behalf of its employees (interfund transfer).
  - Local government payments to state systems (report as local current operation expenditure of function involved).
  - "Pay-as-you-go" workers' compensation programs and other direct payments from current appropriations (report at function of paying agency).

Special Considerations:

1. These data are collected by a separate mail canvass of state workers' compensation systems. However, not all states administer systems that meet the Census Bureau's definition Social Insurance Trust Systems.

2. This function applies solely to state governments. Both the Federal Government and the District of Columbia have "pay-as-you-go" plans that do not meet the Census Bureau's definition of social insurance trust systems.

3. There is no corresponding function in the Census Bureau's employment statistics. Report employment data for administering these programs at *Financial Administration*, code 23.

Codes Y14 and Y15 **Workers' Compensation Systems**
page 2 of 2

Applicable Coding Options for this Expenditure Function

Direct Expenditure

Y14 Benefit Payments (applies to selected state governments only)

Exhibit Codes Related to Workers' Compensation Systems Expenditure
(Summarized – see Chapter 9 for detail)

<u>Code and Description</u>

Y15 Administrative Expenses

Code Y25 **Federal Social Security and Medicare**

Sector: Social Insurance Trusts

Definition: Distribution of cash benefits to eligible persons under Federal-administered retirement, disability, hospital, and health social insurance programs.

Includes: Comprises cash benefits: To retirees or their dependents and survivors from the Federal Old-Age and Survivors Insurance Trust Fund (OASI); to disabled workers or their dependents from the Federal Disability Insurance Trust Fund (DI); to elderly or disabled for hospital, nursing home, and other related care from the Federal Hospital Insurance Trust Fund (HI); to such persons for physician services, outpatient care, renal dialysis, and other health care from the Federal Supplementary Medical Insurance Trust Fund (SMI).

The OASI and DI funds are administered by the Department of Health and Human Services' (HHS) Social Security Administration (SSA) while the HI and SMI funds are operated by HHS Health Care Financing Administration (HCFA).

Excludes: Federal, state, or local governmental contributions as employers for Social Security and Medicare on behalf of their own employees (report as current operation expenditure of function involved); Federal transfers to these Social Security or Health Care trust funds (nonexpenditure); transfers from Social Security Trust Funds to the railroad retirement accounts (nonexpenditure); interest payments on inter-trust borrowings (nonexpenditure); Federal Government administration of these insurance trust funds (report at *Social Insurance Administration*, code *22); payments from HCFA for Medicaid program (report at *Public Welfare - Federal Categorical Assistance Programs*, code *67); payments of SSA other than for the Social Security Trusts, such as SSI and TANF (use code *67) and black lung benefits, child support enforcement, low income energy assistance, etc. (report at *Public Welfare - Other*, code *79).

Special Considerations:

1. The OASI and DI trusts comprise what is commonly referred to as Social Security. The HI and SMI trusts comprise what is commonly referred to as Medicare, Parts A and Part B, respectively.
2. In contrast to public welfare programs funded by appropriations and classed as general expenditure, these social insurance programs all have separate trust funds with dedicated revenues (either payroll taxes or voluntary premiums). See definition of social insurance trust system in Chapters 8 and 9.
3. There is no corresponding function in the Census Bureau's employment statistics. Report employment data for administering these programs at *Financial Administration*, code 23.

Applicable Coding Options for this Expenditure Function

Direct Expenditure

Y25 Benefit Payments

EXPENDITURE FUNCTIONS

Code Y34 **Federal Veterans' Life Insurance**

Definition: Distribution of cash benefits and dividends to eligible veterans and their beneficiaries under Federal-administered veterans' life insurance programs.

Includes: Death claims to beneficiaries, cash values of surrendered policies, distribution of excess earnings and related interest (dividends), disability claims or income payments under veterans' life insurance policies, and matured endowments. Comprises activities of the U.S. Department of Veterans Affairs programs specified below.

Excludes: Two general types of related expenditures are excluded – administrative expenses and the Federal Government's own costs for contributing to this program as an employer.

- Administrative expenses: Loans to policyholders borrowing against cash value of their policies and administration of these life insurance programs (report at *Federal and State Veterans' Services*, code *85)
- Federal Government's own costs: Federal Government contributions to these funds, purchase of securities (nonexpenditures), Federal payments as employer contributions for group life insurance for its employees (report as current operation expenditure of function involved), and burial benefits not paid out of veterans' life insurance funds (use code *Federal and State Veterans' Services*, *85).

Examples:
United State Government Life Insurance
National Service Life Insurance
Veterans Special Life Insurance
Veterans Reopened Insurance
Service Disabled Veterans' Insurance
Veterans' Mortgage Life Insurance
Traumatic Injury Protection Under Servicemembers Group Life Insurance (TSGLI)

Special Consideration:
There is no corresponding function in the Census Bureau's employment statistics. Report employment data for administering these programs at *Other and Unallocable*, code 89.

Applicable Coding Options for this Expenditure Function

Direct Expenditure

Y34 Benefit Payments

Code Y45 **Federal Railroad Retirement**

Definition: Distribution of cash annuities and benefits to eligible retirees and their survivors under Federal-administered rail industry pension programs.

Includes: Annuities for retirement, disability, survivor, occupational disability, and supplemental benefits; and social security equivalent benefits. Comprises activities of the Rail Industry Pension Fund and Railroad Social Security Equivalent Benefit Account, both administered by the U.S. Railroad Retirement Board.

Excludes: Federal payments (transfers) to the railroad retirement accounts (nonexpenditure); Federal Old-Age and Survivors Insurance Trust Fund payments to the railroad retirement accounts (nonexpenditure); purchase of securities (nonexpenditure); benefits from related Railroad Unemployment Insurance Fund (report at *Unemployment Compensation Benefits*, code Y05); and administration of railroad retirement program and railroad unemployment insurance fund (report at *Social Insurance Administration*, code *22).

Special Considerations:

1. Unlike public employee retirement systems, this category covers private sector workers rather than public employees.
2. There is no corresponding function in the Census Bureau's employment statistics. Report employment data for administering these programs at *Social Insurance Administration*, code *22.

Applicable Coding Options for this Expenditure Function

Direct Expenditure

Y45 Benefit Payments

Codes Y53 and Y54 **Other State Social Insurance Trust Systems**

Sector: Social Insurance Trusts

Definition: Distribution of cash benefits to eligible persons under state-administered plans for compulsory or voluntary social insurance programs not elsewhere classified.

Includes:

- Benefits: Claims paid directly to, or on behalf of, beneficiaries for lost income, medical care, disaster reimbursement, and other eligible expenses.
- Other Payments: Exhibit-only code for administration of insurance plans (also report at *Financial Administration,* code *23); recorded losses on investments (also deduct from *Other Investment Earnings*, code Y52); other costs or payments not representing benefits (except purchase of investments).

Excludes: Local government payments to state systems (report as local current operation expenditure of function involved); state government payments to own insurance trusts (interfund transfer); purchase of investments; self-insurance, risk management, insurance against fire or other hazards, and other types of non-social insurance (report at *Other and Unallocable,* code *89); payments on group health, life, or other fringe benefit insurance plans for its employees (report at appropriate function code).

Examples: Colorado – Uninsurable Health Insurance Plan, Maryland – Maryland Automobile Insurance Fund.

Special Considerations:

1. State administered systems must meet all Census Bureau definitions of a social insurance trust system to be classified here. See Chapters 2 and 9 for additional information. Only a limited number of such systems exist.
2. Report state employment data for administering these systems at *Financial Administration*, code 23.
3. Report here any state-administered disaster assistance programs, ONLY IF they have been classified as social insurance trust systems within the Census Bureau's definitions. Otherwise, disaster assistance activity is reported at *Miscellaneous Commercial Activities, NEC*, code *03.
4. Exclude administrative costs from benefits and report them at both code Y54 and *Financial Administration*, code *23.

Codes Y53 and Y54 **Other State Social Insurance Trust Systems**

Applicable Coding Options for this Expenditure Function

Direct Expenditure

Y53 Benefit and Withdrawal Payments

Exhibit Codes Related to Other State Social Insurance Trust Systems Expenditure (Summarized – see Chapter 9 for detail)

Code and Description

Code Y54 Administrative Expenses

5.7 Tables

There are three tables pertaining to the government finance program expenditure statistics. Table 5.1, Description of Character and Object Categories, is extremely important for understanding the character and object designations used in Census Bureau statistics. Table 5.2, Function and Object Code Validity, by Level and Type of Government, contains the detailed combinations of object and function codes that exist in the Census Bureau classification system, and indicates which of these combinations are valid by level and type of government. Where valid, this means that financial statistics on government expenditure could exist for that level or type of government. Appendix Table 2.2, Cross-Classification of Expenditure Function and Object Codes, is a useful cross-reference of coding options currently found in the Census Bureau classification system.

Table 5.1
Description of Character and Object Categories – page 1 of 5

This chart lists and describes the various character and object categories used for classifying expenditure data. Note that a few categories use more than one code. Appendix Table 2.2 provides a cross-classification. of these codes with the function codes.

Code	Category	Description
	Character and object codes can be divided into two main categories – those related to direct expenditure and those related to intergovernmental expenditure.	
	DIRECT EXPENDITURE CATEGORIES:	
		Direct expenditure comprises all final expenditure paid to own employees, former employees (retirees) and to private sector entities outside of the government itself (e.g. all expenditure other than intergovernmental expenditure).
E	Current Operations	Direct expenditure for compensation of own officers and employees and for supplies, materials, and contractual services except any amounts for capital outlay (i.e., for personal services or other objects used in force account construction of permanent and for acquisition of property and equipment).<br><br>Includes repair and maintenance services (e.g., contracts and agreements, materials, and supplies) for the upkeep of buildings, infrastructure, and equipment to maintain required standards of compliance for their intended use. This covers the following activities:<br><br>*Cleaning* — *Refinishing*<br>*Fire repair* — *Refurbishing*<br>*Landscaping (unrelated to new construction)* — *Removal*<br>*Maintenance* — *Repair*<br>*Painting* — *Replacement*<br>*Patching* — *Upkeep*<br>*Reconditioning* — *Waterproofing*
J[1]	Assistance and Subsidies	Direct cash assistance to foreign governments, private individuals, and nongovernmental organizations (e.g., foreign aid, agricultural supports, public welfare, veteran bonuses, and cash grants for tuition and scholarships) neither in return for goods and services nor in repayment of debt and other claims against the government. Excludes payments to private vendors for medical care or other services for the needy; cash benefits under insurance trust programs; intergovernmental expenditure, and administrative costs, assistance-in-kind, and payments for purchase of commodities or services to be furnished free to needy or other individuals (the latter classified as current operations).<br><br>Except for the Federal Government, this category is limited to just four codes: education grants, including scholarships to individuals and aid to private schools or colleges (code J19); public welfare cash assistance payments, either Federal categorical ones (code J67) or others (code J68); and bonuses to veterans (code J85).

Table 5.1
Description of Character and Object Categories – page 2 of 5

Code	Category	Description
I	Interest on Debt	Amounts paid for the use of borrowed money. Includes amounts for interest on debt of the government paid by all its accounting funds. No adjustment is made for premiums or discounts on debt issued or retired. Also excludes premiums paid on debt retired (report at code E89 for general debt retired or codes E91 – E94 for utility debt retired).<br><br>Where amounts to cover interest accruing on debt prior to date of sale are included in the proceeds from issuance of securities, such amounts are treated as offsets to interest expenditure for the year in which they were received.<br><br>There are five codes for interest expenditure, subdivided into interest on general debt (I89) and interest on utility debt, by type of utility (I91, I92, I93, and I94). This distinction is made on the basis of the classification of outstanding debt to which particular interest payments relate.
	CAPITAL OUTLAY CATEGORIES:	Direct expenditure for purchase or construction, by contract or force account, of buildings and other improvements; for purchase of land, equipment, and existing structures; and for payments on capital leases.<br><br>Certain terms (such as rehabilitate, remodel, resurface, renovate, etc.) can denote either construction or maintenance and repair (i.e., current operations). Their classification is based on the circumstances surrounding each situation. If the term refers to activities that materially extend the life or add value to the property, then they are classified under construction; otherwise, they are classified under current operations. No other practical standard (such as dollar amounts) can be uniformly applied to all levels of government.<br><br>Receipts arising from insurance adjustments, sales of equipment, reimbursements, and the like are classified as revenue rather than as offsets to capital outlay expenditure.<br><br>Capital outlay expenditures are subdivided into the three object classes below:
F	Construction	Production, additions, replacements, or major structural alterations to fixed works, undertaken either on a contractual basis by private contractors or through a government's own staff.<br><br>Includes initial production of buildings and structures; initial permanent improvements (other than buildings) that add value to land; and subsequent improvements representing major permanent structural alterations that materially extend the useful life of fixed works. Covers all costs of materials, supplies, and labor that are reasonable and necessary to place an asset in its intended location and prepare it for its intended use, namely, professional fees or architects, engineers, appraisers, and attorneys associated with feasibility studies; preliminary engineering, planning, and design; and related developmental costs such as overhead, office costs, and other purchased construction-related services. Also includes centralized architecture, design, and planning offices whose main role is support of public construction projects, including related salaries and wages, insurance, professional services, etc.

Table 5.1
Description of Character and Object Categories – page 3 of 5

Code	Category	Description
F	Construction (con.)	INCLUDES for buildings and structures: initial or additional installation of equipment and facilities that are integral parts of the structure. Also includes largely site-fabricated equipment integral to enterprise operations, such as power, water, and sewage treatment plants (generators, reactors, boilers, storage tanks, steam engines, and the like).<br><br>Examples of activities included:<br><br>*Additions*<br>*Alterations*<br>*Clearing and grading of land*<br>*Construction-related landscaping*<br>*Conversions*<br>*Demolition needed for construction*<br>*Expansion*<br>*Fire rebuilding*<br>*Improvements*<br>*Installation*<br>*Machinery and equipment integral to structure*<br>*Major replacements*<br>*Mechanical-electrical installations:*<br>*- Central air conditioning*<br>*- Electrical work*<br>*- Elevator*<br>*- Escalators*<br>*- Heating*<br>*- Plumbing*<br>*Modernization*<br>*Modifications*<br>*New building*<br>*Reconstruction*<br>*Redevelopment*<br>*Rehabilitation*<br>*Relocation*<br>*Remodeling*<br>*Renovation*<br>*Resurfacing*<br>*Reroofing*<br><br>Examples of labor and fees included:<br><br>*Contract price*<br>*Insurance during construction*<br>*Job order cost*<br>*Overhead and office costs*<br>*Professional fees:*<br>*- Architects*<br>*- Appraisers*<br>*- Attorneys*<br>*- Designers*<br>*- Engineers*<br>*- Financial advisors*<br>*- Planning*<br>*- Similar purchased services*<br><br>INCLUDES for nonbuilding improvements: initial or additional improvements that add value to land, such as:<br><br>*Airport runways*<br>*Bridges*<br>*Curbs, drains, and gutters*<br>*Fences*<br>*Outdoor lighting*<br>*Parking lots*<br>*Pavements*<br>*Piers*<br>*Railroad tracks*<br>*Retaining walls*<br>*Roadways*<br>*Sidewalks*<br>*Swimming pools*<br>*Traffic signs/signals*<br>*Tunnels*<br>*Utility connections*<br>*Viaducts*<br>*Wharves and docks*

Table 5.1
Description of Character and Object Categories – page 4 of 5

Code	Category	Description
F	Construction (con.)	EXCLUDES expenditure for maintenance and repairs to existing structures or service facilities for the purpose of keeping property in an ordinarily efficient operating condition and which are not considered permanent structural alterations that materially extend the life of the asset – e.g., painting, cleaning, patching, refurbishing, reconditioning, etc. (report under current operations). Also excludes identifiable payments to other governments for construction work (classify under intergovernmental expenditure) as well as purchase or installation of machinery and equipment not integral to fixed structures. In addition, excludes costs attributable to the capitalization of net interest expense incurred during the construction period (report under interest on debt).
G	Purchase of Land and Existing Structures	Acquisition of these assets as such by outright purchase; payments on capital lease-purchase agreements or installment purchase contracts; costs associated with eminent domain (including purchase of rights-of-way); and tax or special assessment foreclosure. Covers all ancillary costs associated with the transaction such as legal and title fees, surveying fees, appraisal and negotiation fees, damage claims, and nonconstruction-related site preparation costs including clearing, filling, leveling, and razing unwanted structures.<br><br>The use of the "G" code for purchase of land and existing structures applies only during the original compilation of data for the Federal and state governments. When these data are inserted into the Bureau's computerized records, they are combined with purchase of equipment data ("K" codes) to produce a category called "Capital Outlay Other Than Construction." The equipment only category ("K" codes), therefore, becomes a subcategory under this one in all subsequent computer files, printouts, and publications. For all other governments, the "G" code represents the purchase of land, equipment, and existing structures – i.e., all capital outlays other than construction.
K[2]	Purchase of Equipment	Purchase and installation of apparatus, furnishings, motor vehicles, office equipment, and the like having a life expectancy of more than five years. Includes both additional equipment and replacements purchased outright or through capital lease or installment purchase contracts. Includes related expenditure necessary to place equipment in its intended state of operation (e.g., transportation charges, installation fees, taxes, etc.).<br><br>Excludes rental of equipment (report under current operations) and equipment and facilities that are integral parts of constructed or purchased structures (report under construction or purchase of land and existing structures, respectively).

X, Y, Z	Social Insurance Trust	Social insurance payments to beneficiaries, employee retirement annuities and other benefits, and withdrawal of insurance or employee retirement contributions. Includes only monies paid to beneficiaries; administrative expenditure for social insurance trust systems is classified as current operations.<br><br>The "X" codes are used for expenditure of employee retirement systems while the "Y" codes are used for expenditure of all other insurance trusts. "Z" is used for employee retirement exhibit codes.

Table 5.1
Description of Character and Object Categories – page 5 of 5

Code	Category	Description
	INTERGOVERNMENTAL EXPENDITURE CATEGORIES:	
		Amounts paid to other governments for performance of specific functions or for general financial support. Includes grants, shared taxes, contingent loans and advances, and any significant and identifiable amounts or reimbursement paid to other governments for performance of general government services or activities.<br>Excludes amounts paid to other governments for purchase of commodities, property, or utility services and for any tax levied as such on facilities of the government.<br><br>The functional classification of intergovernmental expenditure is determined by the nature of the activities to which the receiving government is required to apply it by the paying government, or by the functional classification of activities giving rise to particular items of reimbursement. By definition, all intergovernmental expenditures for activities administered by independent or dependent school systems are classified under education (e.g., school lunch and health programs).<br><br>The character and object codes for intergovernmental expenditure indicate the type of receiving government, as follows:
L	To state government	
M	To local governments	
Q	To independent school districts	
S	To the Federal Government	
	EXHIBIT CODES:	
ZOO	Salaries and Wages	Total expenditure during fiscal year for salaries and wages for all functions and activities, including the general government, liquor store, and utility sectors. Salaries and wages consist of gross amounts paid for compensation of own officers and employees (prior to deducting for withheld taxes, retirement contributions, charges for subsistence, or other purposes), including both current operations expenditure and capital outlay expenditure.
V98	Total Capital Outlay	A calculated statistic representing the total capital outlay expenditure during the fiscal year, for all functions and sectors. Equals the sum of all F and G codes.

Notes:
[1]Prior to 2005, Assistance and Subsidies was represented by multiple object codes, depending on the level and type of government. See Appendix 1 for additional detail.
[2]Object code K is restricted to use for Federal and state governments only.

Table 5.2<br>**Function and Object Code Validity, by Level and Type of Government** – page 1 of 9								
		**Valid for:**						
**Function Codes**	**Object Codes**	**Federal**	**State**	**County**	**Municipal**	**Township**	**Special Districts**	**School Districts**
**REGULAR STATISTICS General Government:**								
01	E	Valid	Valid	Valid	Valid	Valid	Valid	X
	F	Valid	Valid	Valid	Valid	Valid	Valid	X
	G	Valid	Valid	Valid	Valid	Valid	Valid	X
	J	Valid	X	X	X	X	X	X
	K	Valid	Valid	X	X	X	X	X
	L	Valid	X	Valid	Valid	Valid	Valid	X
	M	Valid	Valid	Valid	Valid	Valid	Valid	X
02	E	Valid	X	X	X	X	X	X
	F	Valid	X	X	X	X	X	X
	G	Valid	X	X	X	X	X	X
	J	Valid	X	X	X	X	X	X
	K	Valid	X	X	X	X	X	X
	L	Valid	X	X	X	X	X	X
	M	Valid	X	X	X	X	X	X
03	E	X	Valid	Valid	Valid	Valid	Valid	X
	F	X	Valid	Valid	Valid	Valid	Valid	X
	G	X	Valid	Valid	Valid	Valid	Valid	X
	K	X	Valid	X	X	X	X	X
04	E	Valid	Valid	Valid	Valid	Valid	Valid	X
	F	Valid	Valid	Valid	Valid	Valid	Valid	X
	G	Valid	Valid	Valid	Valid	Valid	Valid	X
	J	Valid	X	X	X	X	X	X
	K	Valid	Valid	X	X	X	X	X
	L	Valid	X	Valid	Valid	Valid	Valid	X
	M	Valid	Valid	Valid	Valid	Valid	Valid	X
05	E	Valid	Valid	Valid	Valid	Valid	Valid	X
	F	Valid	Valid	Valid	Valid	Valid	Valid	X
	G	Valid	Valid	Valid	Valid	Valid	Valid	X
	K	Valid	Valid	X	X	X	X	X
	L	Valid	X	Valid	Valid	Valid	Valid	X
	M	Valid	Valid	Valid	Valid	Valid	Valid	X
06	E	Valid	X	X	X	X	X	X
	F	Valid	X	X	X	X	X	X
	G	Valid	X	X	X	X	X	X
	J	Valid	X	X	X	X	X	X
	K	Valid	X	X	X	X	X	X
12	E	X	Valid	Valid	Valid	Valid	Valid	Valid
	F	X	Valid	Valid	Valid	Valid	Valid	Valid
	G	X	Valid	Valid	Valid	Valid	Valid	Valid
	K	X	Valid	X	X	X	X	X
	L	X	X	Valid	Valid	Valid	Valid	Valid
	M	X	Valid	Valid	Valid	Valid	Valid	Valid
	Q	X	Valid	X	X	X	X	Valid

Table 5.2
Function and Object Code Validity, by Level and Type of Government – page 2 of 9

Function Codes	Object Codes	Valid for: Federal	State	County	Municipal	Township	Special Districts	School Districts
14	E	Valid	X	X	X	X	X	X
	F	Valid	X	X	X	X	X	X
	G	Valid	X	X	X	X	X	X
	J	Valid	X	X	X	X	X	X
	K	Valid	X	X	X	X	X	X
16	E	X	Valid	Valid	Valid	Valid	X	Valid
	F	X	Valid	Valid	Valid	Valid	X	Valid
	G	X	Valid	Valid	Valid	Valid	X	Valid
	K	X	Valid	X	X	X	X	X
18	E	X	Valid	Valid	Valid	Valid	X	Valid
	F	X	Valid	Valid	Valid	Valid	X	Valid
	G	X	Valid	Valid	Valid	Valid	X	Valid
	K	X	Valid	X	X	X	X	X
	L	X	X	Valid	Valid	Valid	X	Valid
	M	X	Valid	Valid	Valid	Valid	X	Valid
	Q	X	Valid	X	X	X	X	X
19	J	X	Valid	X	X	X	X	X
20	E	Valid	X	X	X	X	X	X
	F	Valid	X	X	X	X	X	X
	G	Valid	X	X	X	X	X	X
	J	Valid	X	X	X	X	X	X
	K	Valid	X	X	X	X	X	X
	L	Valid	X	X	X	X	X	X
	M	Valid	X	X	X	X	X	X
21	E	Valid	Valid	X	X	X	X	X
	F	Valid	Valid	X	X	X	X	X
	G	Valid	Valid	X	X	X	X	X
	J	Valid	X	X	X	X	X	X
	K	Valid	Valid	X	X	X	X	X
	L	Valid	X	X	X	X	X	X
	M	Valid	Valid	X	X	X	X	X
22[1]	E	Valid	Valid	X	X[1]	X	X	X
	F	Valid	Valid	X	X[1]	X	X	X
	G	Valid	Valid	X	X[1]	X	X	X
	J	Valid	X	X	X	X	X	X
	K	Valid	Valid	X	X	X	X	X
	L	Valid	X	X	X	X	X	X
	M	Valid	X	X	X	X	X	X
23	E	Valid	Valid	Valid	Valid	Valid	X	X
	F	Valid	Valid	Valid	Valid	Valid	X	X
	G	Valid	Valid	Valid	Valid	Valid	X	X
	J	Valid	X	X	X	X	X	X
	K	Valid	Valid	X	X	X	X	X
	L	Valid	X	Valid	Valid	Valid	X	X
	M	Valid	Valid	Valid	Valid	Valid	X	X
24	E	X	X	Valid	Valid	Valid	Valid	X
	F	X	X	Valid	Valid	Valid	Valid	X
	G	X	X	Valid	Valid	Valid	Valid	X
	M	X	X	Valid	Valid	Valid	Valid	X

Table 5.2<br>Function and Object Code Validity, by Level and Type of Government – page 3 of 9								
		Valid for:						
Function Codes	Object Codes	Federal	State	County	Municipal	Township	Special Districts	School Districts
25	E	Valid	Valid	Valid	Valid	Valid	X	X
	F	Valid	Valid	Valid	Valid	Valid	X	X
	G	Valid	Valid	Valid	Valid	Valid	X	X
	J	Valid	X	X	X	X	X	X
	K	Valid	Valid	X	X	X	X	X
	L	Valid	X	Valid	Valid	Valid	X	X
	M	Valid	Valid	Valid	Valid	Valid	X	X
26	E	Valid	Valid	X	X	X	X	X
	F	Valid	Valid	X	X	X	X	X
	G	Valid	Valid	X	X	X	X	X
	K	Valid	Valid	X	X	X	X	X
28	E	Valid	X	X	X	X	X	X
	F	Valid	X	X	X	X	X	X
	G	Valid	X	X	X	X	X	X
	J	Valid	X	X	X	X	X	X
	K	Valid	X	X	X	X	X	X
	L	Valid	X	X	X	X	X	X
	M	Valid	X	X	X	X	X	X
29	E	Valid	Valid	Valid	Valid	Valid	X	X
	F	Valid	Valid	Valid	Valid	Valid	X	X
	G	Valid	Valid	Valid	Valid	Valid	X	X
	J	Valid	X	X	X	X	X	X
	K	Valid	Valid	X	X	X	X	X
	L	Valid	X	Valid	Valid	Valid	X	X
	M	Valid	Valid	Valid	Valid	Valid	X	X
30	M	X	Valid	X	X	X	X	X
31	E	X	Valid	Valid	Valid	Valid	X	X
	F	X	Valid	Valid	Valid	Valid	X	X
	G	X	Valid	Valid	Valid	Valid	X	X
	K	X	Valid	X	X	X	X	X
32	E	Valid	Valid	Valid	Valid	Valid	Valid	X
	F	Valid	Valid	Valid	Valid	Valid	Valid	X
	G	Valid	Valid	Valid	Valid	Valid	Valid	X
	J	Valid	X	X	X	X	X	X
	K	Valid	Valid	X	X	X	X	X
	L	Valid	X	Valid	Valid	Valid	Valid	X
	M	Valid	Valid	Valid	Valid	Valid	Valid	X
36	E	Valid	Valid	Valid	Valid	Valid	Valid	Valid
	F	Valid	Valid	Valid	Valid	Valid	Valid	Valid
	G	Valid	Valid	Valid	Valid	Valid	Valid	Valid
	J	Valid	X	X	X	X	X	X
	K	Valid	Valid	X	X	X	X	X
	L	Valid	X	Valid	Valid	Valid	Valid	Valid
	M	Valid	Valid	Valid	Valid	Valid	Valid	Valid
37	E	Valid	X	X	X	X	X	X
	F	Valid	X	X	X	X	X	X
	G	Valid	X	X	X	X	X	X
	J	Valid	X	X	X	X	X	X
	K	Valid	X	X	X	X	X	X
	L	Valid	X	X	X	X	X	X
	M	Valid	X	X	X	X	X	X

Table 5.2<br>**Function and Object Code Validity, by Level and Type of Government –** page 4 of 9								
		**Valid for:**						
**Function Codes**	**Object Codes**	**Federal**	**State**	**County**	**Municipal**	**Township**	**Special Districts**	**School Districts**
39	E	Valid	X	X	X	X	X	X
	F	Valid	X	X	X	X	X	X
	G	Valid	X	X	X	X	X	X
	J	Valid	X	X	X	X	X	X
	K	Valid	X	X	X	X	X	X
	L	Valid	X	X	X	X	X	X
	M	Valid	X	X	X	X	X	X
44	E	Valid	Valid	Valid	Valid	Valid	Valid	X
	F	Valid	Valid	Valid	Valid	Valid	Valid	X
	G	Valid	Valid	Valid	Valid	Valid	Valid	X
	J	Valid	X	X	X	X	X	X
	K	Valid	Valid	X	X	X	X	X
	L	Valid	X	Valid	Valid	Valid	Valid	X
	M	Valid	Valid	Valid	Valid	Valid	Valid	X
45	E	X	Valid	Valid	Valid	Valid	Valid	X
	F	X	Valid	Valid	Valid	Valid	Valid	X
	G	X	Valid	Valid	Valid	Valid	Valid	X
	K	X	Valid	X	X	X	X	X
50[2]	E	Valid	Valid	Valid	Valid	Valid	Valid	X
	F	Valid	Valid	Valid	Valid	Valid	Valid	X
	G	Valid	Valid	Valid	Valid	Valid	Valid	X
	J	Valid	X	X	X	X	X	X
	K	Valid	Valid	X	X	X	X	X
	L	Valid	X	Valid[2]	Valid[2]	Valid[2]	Valid[2]	X
	M	Valid	Valid	Valid	Valid	Valid	Valid	X
51	E	Valid	X	X	X	X	X	X
	J	Valid	X	X	X	X	X	X
	L	Valid	X	X	X	X	X	X
	M	Valid	X	X	X	X	X	X
52	E	Valid	Valid	Valid	Valid	Valid	Valid	X
	F	Valid	Valid	Valid	Valid	Valid	Valid	X
	G	Valid	Valid	Valid	Valid	Valid	Valid	X
	J	Valid	X	X	X	X	X	X
	K	Valid	Valid	X	X	X	X	X
	L	Valid	X	Valid	Valid	Valid	Valid	X
	M	Valid	Valid	Valid	Valid	Valid	Valid	X
53	E	Valid	X	X	X	X	X	X
	F	Valid	X	X	X	X	X	X
	G	Valid	X	X	X	X	X	X
	J	Valid	X	X	X	X	X	X
	K	Valid	X	X	X	X	X	X
	L	Valid	X	X	X	X	X	X
	M	Valid	X	X	X	X	X	X
55	E	X	Valid	X	X	X	X	X
	F	X	Valid	X	X	X	X	X
	G	X	Valid	X	X	X	X	X
	K	X	Valid	X	X	X	X	X
	M	X	Valid	X	X	X	X	X

**Table 5.2** **Function and Object Code Validity, by Level and Type of Government**- page 5 of 9								
		**Valid for:**						
**Function Codes**	**Object Codes**	**Federal**	**State**	**County**	**Municipal**	**Township**	**Special Districts**	**School Districts**
56	E	Valid	Valid	X	X	X	X	X
	F	Valid	Valid	X	X	X	X	X
	G	Valid	Valid	X	X	X	X	X
	J	Valid	X	X	X	X	X	X
	K	Valid	Valid	X	X	X	X	X
	L	Valid	X	X	X	X	X	X
	M	Valid	Valid	X	X	X	X	X
57	E	Valid	X	X	X	X	X	X
	F	Valid	X	X	X	X	X	X
	G	Valid	X	X	X	X	X	X
	J	Valid	X	X	X	X	X	X
	K	Valid	X	X	X	X	X	X
	L	Valid	X	X	X	X	X	X
	M	Valid	X	X	X	X	X	X
58	E	Valid	X	X	X	X	X	X
	F	Valid	X	X	X	X	X	X
	G	Valid	X	X	X	X	X	X
	J	Valid	X	X	X	X	X	X
	K	Valid	X	X	X	X	X	X
	L	Valid	X	X	X	X	X	X
	M	Valid	X	X	X	X	X	X
59	E	Valid	Valid	Valid	Valid	Valid	Valid	X
	F	Valid	Valid	Valid	Valid	Valid	Valid	X
	G	Valid	Valid	Valid	Valid	Valid	Valid	X
	J	Valid	X	X	X	X	X	X
	K	Valid	Valid	X	X	X	X	X
	L	Valid	X	Valid	Valid	Valid	Valid	X
	M	Valid	Valid	Valid	Valid	Valid	Valid	X
60	E	X	Valid	Valid	Valid	Valid	Valid	X
	F	X	Valid	Valid	Valid	Valid	Valid	X
	G	X	Valid	Valid	Valid	Valid	Valid	X
	K	X	Valid	X	X	X	X	X
	L	X	X	Valid	Valid	Valid	Valid	X
	M	X	Valid	Valid	Valid	Valid	Valid	X
61	E	Valid	Valid	Valid	Valid	Valid	Valid	X
	F	Valid	Valid	Valid	Valid	Valid	Valid	X
	G	Valid	Valid	Valid	Valid	Valid	Valid	X
	J	Valid	X	X	X	X	X	X
	K	Valid	Valid	X	X	X	X	X
	L	Valid	X	Valid	Valid	Valid	Valid	X
	M	Valid	Valid	Valid	Valid	Valid	Valid	X
62	E	Valid	Valid	Valid	Valid	Valid	Valid	X
	F	Valid	Valid	Valid	Valid	Valid	Valid	X
	G	Valid	Valid	Valid	Valid	Valid	Valid	X
	J	Valid	X	X	X	X	X	X
	K	Valid	Valid	X	X	X	X	X
	L	Valid	X	Valid	Valid	Valid	Valid	X
	M	Valid	Valid	Valid	Valid	Valid	Valid	X

Table 5.2<br>**Function and Object Code Validity, by Level and Type of Government**- page 6 of 9								
		**Valid for:**						
**Function Codes**	**Object Codes**	**Federal**	**State**	**County**	**Municipal**	**Township**	**Special Districts**	**School Districts**
66	E	X	Valid	Valid	Valid	Valid	X	X
	F	X	Valid	Valid	Valid	Valid	X	X
	G	X	Valid	Valid	Valid	Valid	X	X
	K	X	Valid	X	X	X	X	X
	L	X	X	Valid	Valid	Valid	X	X
	M	X	Valid	Valid	Valid	Valid	X	X
67[1]	J	Valid	Valid	Valid	Valid	Valid	X	X
	L	Valid	X	Valid	Valid	Valid	X	X
	M	Valid	Valid	Valid	Valid	Valid	X	X
	S	X	Valid	X	X[1]	X	X	X
68	J	X	Valid	Valid	Valid	Valid	X	X
	M	X	Valid	Valid	Valid	Valid	X	X
74[1]	E	X	Valid	Valid	Valid	Valid	X	X
	S	X	Valid	X	X[1]	X	X	X
75	E	X	Valid	Valid	Valid	Valid	X	X
77	E	X	Valid	Valid	Valid	Valid	Valid	X
	F	X	Valid	Valid	Valid	Valid	Valid	X
	G	X	Valid	Valid	Valid	Valid	Valid	X
	K	X	Valid	X	X	X	X	X
79	E	Valid	Valid	Valid	Valid	Valid	Valid	X
	F	Valid	Valid	Valid	Valid	Valid	Valid	X
	G	Valid	Valid	Valid	Valid	Valid	Valid	X
	J	Valid	X	X	X	X	X	X
	K	Valid	Valid	X	X	X	X	X
	L	Valid	X	Valid	Valid	Valid	Valid	X
	M	Valid	Valid	Valid	Valid	Valid	Valid	X
80	E	X	Valid	Valid	Valid	Valid	Valid	X
	F	X	Valid	Valid	Valid	Valid	Valid	X
	G	X	Valid	Valid	Valid	Valid	Valid	X
	K	X	Valid	X	X	X	X	X
	L	X	X	Valid	Valid	Valid	Valid	X
	M	X	Valid	Valid	Valid	Valid	Valid	X
81	E	Valid	Valid	Valid	Valid	Valid	Valid	X
	F	Valid	Valid	Valid	Valid	Valid	Valid	X
	G	Valid	Valid	Valid	Valid	Valid	Valid	X
	K	Valid	Valid	X	X	X	X	X
	L	Valid	X	Valid	Valid	Valid	Valid	X
	M	Valid	Valid	Valid	Valid	Valid	Valid	X
85	E	Valid	Valid	X	X	X	X	X
	F	Valid	Valid	X	X	X	X	X
	G	Valid	Valid	X	X	X	X	X
	J	Valid	Valid	X	X	X	X	X
	K	Valid	Valid	X	X	X	X	X
87	E	Valid	Valid	Valid	Valid	Valid	Valid	X
	F	Valid	Valid	Valid	Valid	Valid	Valid	X
	G	Valid	Valid	Valid	Valid	Valid	Valid	X
	J	Valid	X	X	X	X	X	X
	K	Valid	Valid	X	X	X	X	X
	L	Valid	X	Valid	Valid	Valid	Valid	X

	M	Valid	Valid	Valid	Valid	Valid	Valid	X

Table 5.2

Function and Object Code Validity, by Level and Type of Government – page 7 of 9

Function Codes	Object Codes	Valid for: Federal	State	County	Municipal	Township	Special Districts	School Districts
89	E	Valid	Valid	Valid	Valid	Valid	Valid	X
	F	Valid	Valid	Valid	Valid	Valid	Valid	X
	G	Valid	Valid	Valid	Valid	Valid	Valid	X
	I	Valid	Valid	Valid	Valid	Valid	Valid	Valid
	J	Valid	X	X	X	X	X	X
	K	Valid	Valid	X	X	X	X	X
	L	Valid	X	Valid	Valid	Valid	Valid	X
	M	Valid	Valid	Valid	Valid	Valid	Valid	
	S	X	Valid	X	X	X	X	X
**Liquor Stores:**								
90	E	X	Valid	Valid	Valid	Valid	X	X
	F	X	Valid	Valid	Valid	Valid	X	X
	G	X	Valid	Valid	Valid	Valid	X	X
	K	X	Valid	X	X	X	X	X
**Utilities:**								
91[3]	E	X	Valid	Valid	Valid	Valid	Valid	X
	F	X	Valid	Valid	Valid	Valid	Valid	X
	G	X	Valid	Valid	Valid	Valid	Valid	X
	I	X	Valid	Valid	Valid	Valid	Valid	X
	K	X	Valid	X	X	X	X	X
	L	X	X	Valid	Valid	Valid	Valid	X
	M	X	Valid	Valid	Valid	Valid	Valid	X
92[3]	E	X	Valid	Valid	Valid	Valid	Valid	X
	F	X	Valid	Valid	Valid	Valid	Valid	X
	G	X	Valid	Valid	Valid	Valid	Valid	X
	I	X	Valid	Valid	Valid	Valid	Valid	X
	K	X	Valid	X	X	X	X	X
	L	X	X	Valid	Valid	Valid	Valid	X
	M	X	Valid	Valid	Valid	Valid	Valid	X
93[3]	E	X	Valid	Valid	Valid	Valid	Valid	X
	F	X	Valid	Valid	Valid	Valid	Valid	X
	G	X	Valid	Valid	Valid	Valid	Valid	X
	I	X	Valid	Valid	Valid	Valid	Valid	X
	K	X	Valid	X	X	X	X	X
	L	X	X	Valid	Valid	Valid	Valid	X
	M	X	Valid	Valid	Valid	Valid	Valid	X
94[3]	E	X	Valid	Valid	Valid	Valid	Valid	X
	F	X	Valid	Valid	Valid	Valid	Valid	X
	G	X	Valid	Valid	Valid	Valid	Valid	X
	I	X	Valid	Valid	Valid	Valid	Valid	X
	K	X	Valid	X	X	X	X	X
	L	X	X	Valid	Valid	Valid	Valid	X
	M	X	Valid	Valid	Valid	Valid	Valid	X

Table 5.2
Function and Object Code Validity, by Level and Type of Government – page 8 of 9

Insurance Trusts	Object Codes	Valid for: Federal	State	County	Municipal	Township	Special Districts	School Districts
Employee Retirement	X11	Valid	Valid	Valid	Valid	Valid	Valid	Valid
	X12	X	Valid	Valid	Valid	Valid	Valid	Valid
Unemployment Compensation	Y05	Valid	Valid	X	X[1]	X	X	X
	Y06	X	Valid	X	X[1]	X	X	X
Workers' Compensation	Y14	X	Valid	X	X	X	X	X
Other	Y53	X	Valid	X	X	X	X	X
Federal Social Security	Y25	Valid	X	X	X	X	X	X
Federal Veterans' Life Insurance	Y34	Valid	X	X	X	X	X	X
Federal Railroad Retirement	Y45	Valid	X	X	X	X	X	X

Table 5.2<br>**Function and Object Code Validity, by Level and Type of Government –** page 9 of 9								
		**Valid for:**						
**EXHIBIT CODES**	**Object Codes**	**Federal**	**State**	**County**	**Municipal**	**Township**	**Special Districts**	**School Districts**
**General Government:**								
Lotteries:	Z52[4]	X	Valid	X	X[4]	X	X	X
	Z53[4]	X	Valid	X	X[4]	X	X	X
Liquor Stores:	Z42	X	Valid	X	X	X	X	X
	Z43	X	Valid	X	X	X	X	X
	Z45	X	Valid	X	X	X	X	X
	Z46	X	Valid	X	X	X	X	X
	Z47	X	Valid	X	X	X	X	X
**Insurance Trusts:**								
Employee Retirement	Z13	Valid	Valid	Valid	Valid	Valid	Valid	Valid
	Z14	Valid	Valid	Valid	Valid	Valid	Valid	Valid
	Z15	Valid	Valid	Valid	Valid	Valid	Valid	Valid
	Z16	Valid	Valid	Valid	Valid	Valid	Valid	Valid
	Z93	Valid	Valid	Valid	Valid	Valid	Valid	Valid
Workers' Compensation	Y15	X	Valid	X	X	X	X	X
Other	Y54	X	Valid	X	X	X	X	X
**All Sectors:**								
Tobacco	CGE[1]	X	Valid	X	X[1]	X	X	X
Salaries & wages	Z00	Valid	Valid	Valid	Valid	Valid	Valid	Valid
Total capital	V98	X	X	Valid	Valid	Valid	Valid	Valid

Notes:

[1]These function/object codes combinations also are valid for Washington, DC, owing to its unique relationship to the Federal Government (for the following object and function codes combinations):

E22	S67	Y05
F22	S74	Y06
G22	CGE	

[2]Code L50 is valid for local government types 1, 2, 3, and 4, effective with the fiscal year 2006 survey of government finance. It was not effective for the 2005 survey, other than for the Federal Government. For 2004 and earlier, this code was valid for the Federal and local governments.

[3]Theses object/function combinations are classified in the General Government Sector, not in the Utilities Sector:

L91	M91
L92	M92
L93	M93
L94	M94

[4]These exhibit codes are valid for Washington, DC only at the local government level.

Chapter 6. Indebtedness

Introduction

This edition of the *Classification Manual* contains revisions to the classification system used by the Census Bureau for government debt statistics. The revisions were the result of the 2005 redesign of the government finance statistics program. In keeping with the theme of the 2005 redesign, revisions were made to enhance internal consistency in statistical categories and to simplify the classification categories across levels and types of governments. To meet this objective, the number of categories used to classify state and local government regular debt statistics was reduced from sixty-six to eight, with the former categories combined into broader groupings.

Even though the debt classification categories were simplified, there was no change to the basic concepts and coverage, or to the overall definitions. Rather, the revisions represented a simplification of the classification structure, applied to the manner in which the statistics are presented and reported.

The 2005 changes to the classification system for debt statistics are in sharp contrast to the revisions made to government debt statistics that took effect with the 1988 government finance program. The latter revisions included coverage and conceptual changes to the definition of government debt. Summarized in Section 6.1.6, the 1988 conceptual changes covered how the Census Bureau treated the following debt topics:

- Refunding debt (advanced refunding, defeasance, etc.).
- Public debt for private purposes (industrial revenue bonds, mortgage and housing revenue bonds, and so forth).
- Taxable public debt and related options.

Users should refer to the previous edition of the *Classification Manual* for additional information on the1988 revisions.

The 2005 redesign did not result in any major shifts in the levels and trends of government debt statistics reported by the Census Bureau. Nor did the 2005 redesign create any need for historical revisions to the time series for government debt statistics. Since the changes in this edition of the *Classification Manual* represent primarily consolidations of debt categories, all aggregates in the historical data are consistent, in terms of coverage and concept, with statistics from earlier years.

6.1 Concepts and Definitions

The Census Bureau concept of government debt is both inclusive and broad in scope. The inclusive nature of the definition refers to the types of debt instruments included in the definition. The broad scope of the definition refers to the range of government activity covered. These concepts are explained in more detail in the following Sections (6.1.1 through 6.1.4).

6.1.1 Definitions – Debt Instruments

Government debt comprises all interest-bearing short-term credit obligations and all long-term credit obligations incurred in the name of the government and all its dependent agencies. This definition

includes all debt, whether backed by the government's full faith and credit or nonguaranteed. It includes tax-exempt as well as taxable public debt.

Government debt also includes judgments, mortgages, "revenue" and "earning" bonds, and special assessment obligations, as well as the more traditional general obligation bonds, notes, and interest-bearing short-term warrants. It includes not only government debt for public improvements (roads, sewers, airports, etc.), but also debt issued for the direct benefit of the private sector entities (industrial development, mortgage revenue, pollution control and abatement, etc.).

6.1.2 Liabilities Outside Census Bureau Definition

The concept of government debt does not cover every type of liability listed on the balance sheets or elsewhere in government finance reports. The following types of liabilities are excluded from Census Bureau statistics:

- Non-interest-bearing short-term obligations, such as accounts payable, non-interest-bearing warrants, and the like. Note, however, that non-interest-bearing long-term obligations having a formal debt instrument (such as a fixed repayment schedule) are classified as government debt.

- Interfund advances, loans, or other obligations transacted between accounting funds of the same government (see Section 3.9 for a discussion of intragovernmental transactions). As a practical matter, these debt statistics do include formal debt instruments of a government that are held as investments by its own agencies or fund on behalf of others.

- Amounts held and owned by a government in a trust or agency capacity on behalf of others (see Section 3.10).

- Contingent loans and advances from other governments, which are classified as intergovernmental transactions (see Section 3.7).

- Rights of individuals to benefits from retirement funds or other social insurance trust systems.

- Unfunded liabilities of retirement systems, unpaid annual or sick leave, or other obligations without a formal debt instrument specifying terms, length, interest rate, etc.

- Leases, including both capital and operating leases (see Section 6.4.5).

- Loan guarantees and nonguaranteed obligations of the Federal Government.

Chart 6.A provides a sample of the most common types of government liabilities that are included and excluded from the Census Bureau's definition of public debt.

Chart 6.A
Examples of Liabilities Included and Excluded from Census Bureau Definition of Public Debt

This chart is by no means an exhaustive list of all possible liabilities that governments incur; rather, it shows the most common types of debt found in annual finance reports.

Examples of Liabilities Included in Census Bureau Public Debt	Examples of Liabilities Excluded from Census Bureau Public Debt
Bank lines of credit	Accounts payable
Bank loans	Accrued expenses
Bond anticipation notes	Accrued interest payable
Certificates of obligations	Accrued vacation or sick leave
Certificated of participation (COPs)	Contingent loans and advances
Construction loan notes (CLNs)	Defeased debt (from advance refunding operations)
Conduit debt	Deferred compensation payable
"Deep-discount" debt, such as:	Deferred income or revenue
Compound interest bonds (CIBs)	Installment purchase agreements or contracts
Zero-coupon bonds	Interfund loans and advances ("due to other funds")
Demand bonds	Leases (operating or capital)
General obligation bonds (GOBs)	Long-term contracts payable
General obligation self-supporting bonds	Noninterest-bearing short-term warrants and obligations
General obligation tax increment bonds	Purchase contracts
Grant anticipation notes	Refundable deposits
Industrial revenue bonds	Reverse repurchase agreements
Lease revenue bonds	Rights of persons to benefits from public employee
Lease-rental bonds	retirement or other insurance trust systems,
Limited tax bonds	including health benefits for retirees.
Mortgage notes	Salaries payable
Non-interest-bearing long-term warrants and	Severance payable
obligations with debt instruments	Vouchers payable
Notes payable	
Pollution control bonds	
Revenue anticipation notes	
Serial bonds	
Special assessment bonds	
Special obligation bonds	
Special revenue bonds	
Tax anticipation notes	
Taxable public debt	
Term bonds	

6.1.3 Definitions – Reporting Entity

A second aspect of the Census Bureau definition of government debt is that it covers debt obligations of all dependent agencies of the government, including agencies, boards, commissions, or other organizations (see Chapter 1 for a more comprehensive explanation of dependent agencies). Government business-type activities, particularly utilities, have debt that is payable exclusively from earnings of the facilities which the debt financed. Many special assessment obligations are paid completely from levies on the property benefiting from such improvements, without recourse to the general credit of the government. State authorities, educational institutions, and other agencies frequently have debt secured only by their own revenues, other dedicated receipts, or agency properties. These types of obligations are issued widely by dependent agencies of a government, such as special improvement districts of city corporations, state dormitory authorities, or housing finance agencies.

The Census Bureau has been consistent in using this broad coverage and including debt of dependent agencies, going back to the development of the Census of Governments program in 1952. This was true even though the parent governments often did not maintain central accounts of such agency debt.

The Census Bureau assigns debt, as defined above, to the government in whose name it is incurred, regardless of the location of responsibility for debt service. In the case of public debt for private purposes, this generally represents the government whose tax-exempt status was used to issue such debt. State obligations for which interest and principal payments are financed by local government payments to the state are treated as state debt. Similarly, debt of an agency classified as dependent on a local government is treated as local government debt.

6.1.4 Census Bureau Reporting and the Governmental Accounting Standards Board

The Governmental Accounting Standards Board (GASB) was established in 1984. Its purpose is to develop and maintain standards of financial accounting and reporting for state and local governments in the United States. The Census Bureau and GASB have nearly identical approaches to defining coverage for debt statistics that should be reported on behalf of the parent government. The following hierarchy shows the GASB terminology as it relates to parent government and dependent agency debt activity that is included in a government's debt statistics for Census Bureau purposes.[1]

- *Parent Government (including major permanent funds within each category below)
    - *General Fund
    - *Special Revenue Funds
    - *Capital Projects Funds
    - *Debt Service Funds
- *Proprietary Funds
    - *Enterprise Funds
    - Internal Service Funds
- *Component Units
    - *Blended Component Units
    - *Discrete Presented Component Units (including major and non-major units)
- Fiduciary Funds

[1] Users should refer to GASB 39 and to Section 2100 of the GASB Codification of Governmental Accounting and Financial Reporting Standards for more detailed information.

Pension and Other Employee Benefit Trust Funds
Investment Trust Funds
Private Purpose Trust Funds
Agency Funds

In the above listing, those reporting funds noted with an asterisk (*) often have government debt that could be in-scope for Census Bureau reporting purposes. However, each dependent agency or activity of what the Census Bureau considers to be the parent government (GASB primary government, usually) must fall within the definitions outlined in Chapter 1. In the above listing, for example, it is possible that some Internal Service Funds could reflect activity that should be included in the Census Bureau debt statistics.

The Census Bureau continues to monitor and analyze GASB statements for relevance to Census Bureau reporting and for purposes of developing relational classifications.

6.1.5 Three Way Classification of Public Debt

For Census Bureau statistics, government debt is classified into three categories using a 3-digit coding scheme. The first category applies to short and long-term debt, while the second and third categories apply only to long-term debt. Below is a summary of each:

- *By length of term*. Debt is classified as being either short or long-term. Note that for short-term debt, only the amounts outstanding at the beginning and end of the fiscal year are reported. Unlike long-term debt data, no effort is made to collect the entire amount of short-term debt sold and paid off during the fiscal year or to categorize short-term debt by function.

- *By type of long-term debt.* Long-term debt is classified by one of four types: outstanding at the beginning of the fiscal year, issued during the fiscal year, retired during the fiscal year, or outstanding at the end of the fiscal year. Note that two types record debt activity over a period of time, while two measure debt at a specific point. Also, the end of fiscal year refers to the government's own fiscal year, not the Census Bureau reporting period.

- *By purpose of long-term debt.* Long-term debt is classified according to the purpose for funding. There are currently two categories:

  Public Debt For Private Purposes
  Public Debt, Unspecified Public Purposes

6.1.6 Classification Changes Effective With 2005 Survey Year

The Census Bureau made two changes to the classification system for government debt statistics that was in place from 1988 through 2004. First, the number of categories used in classifying the "purpose" of state and local government long-term debt (the functional activity the debt supported) was reduced from 8 to 2. The pre-2005 statistical classifications are referenced in Table 6.2. To summarize, the previous categories utilized the basic concept of "government sectors" described in Chapter 2, in combination with the functional concepts used for government expenditure statistics:

General Debt:	Elementary and Secondary Education<br>Higher and Other Education<br>Public Debt for Private Purposes<br>All Other Debt, N.E.C.
Utility Debt:	Water Supply Systems<br>Electric Power Systems<br>Natural Gas Supply Systems<br>Public Mass Transit Systems

The second classification change effective with 2005 debt statistics was that the distinct categories used to classify "character" of long-term debt were eliminated. Prior to the 2005 survey year, the Census Bureau statistics on government debt also included the following additional classification category:

- *By character of long-term debt.* Long-term debt is classified as either guaranteed (backed by the full-faith and credit of the government) or as nonguaranteed (such as revenue bonds).

Census Bureau statistics no longer distinguish between debt that was "guaranteed" and "nonguaranteed." This change meant that the Census Bureau statistics on government debt went from a four way classification system to a three way classification system.

Table 6.2 contains a historical crosswalk of the various classification categories in use for the following historical time frames:

Current surveys (effective with the 2005 redesign)
1988 to 2004
1987 and earlier

The table shows where each historical debt category is contained in the current Census Bureau classification structure.

6.2 Measurement Issues for Debt Statistics

This section notes several important concepts related to the measurement of debt in the Census Bureau's statistical system. Some of these concepts were explained earlier in Chapters 1, 2, and 3, but are reiterated here.

6.2.1 Measurement Issues: Timing

Debt statistics represent a mix of transactions made during the fiscal year and amounts measured as of specific points in the fiscal year. For short-term debt, statistics reflect amounts at the beginning and end of the government fiscal year only. There are no statistics on short-term debt issued or retired. As a result, if a government issues short-term debt during the fiscal year and retires it prior to the end of the fiscal year, the transactions are excluded completely from Census Bureau statistics.

For long-term debt, all transactions are included in the statistics. Debt outstanding is measured at the beginning and at the close of the fiscal year. In addition, all debt issued and retired transactions are measured, regardless of when they occurred during the fiscal year.

In cases where a dependent agency of a government has a fiscal year that differs from the parent government, debt for the dependent agency are combined with that of the parent, even though they represent different time frames. Similarly, when debt statistics are tabulated for more than one government, the fiscal year differences are ignored. All data are summed as if they covered the same time periods, without any adjustments.

The treatment of fiscal years and the definition applied by the Census Bureau were explained in Section 3.2.

6.2.2 Measurement Issues: Valuation

All statistics are reported or compiled in current dollars only. The Census Bureau makes no effort to account for historical price level changes in its statistical program on government finances. This is because of the diversity of users, and uses for, the data. By reporting only in current dollar values, the Census Bureau recognizes the various needs and special circumstances of its data users and does not introduce any statistical bias to the time series.

The primary implication for statistics on debt is that the amounts outstanding reflect the value when issued, less current and prior year retirements. Long-term debt carried for lengthy periods of time, such as 20 years, thus was valued at the time it was issued and is not adjusted for price level changes.

6.2.3 Measurement Issues: Aggregation and Tabulation

Aggregate statistics for an individual government reflect the debt of the parent government and all of its dependent agencies. There are no issues involving flows of funds between or among these entities and their accounting funds, since by definition a government cannot borrow from itself. The basic concept of total debt for an individual government thus represents all monies owed to entities outside of the defined government.

Tabulated statistics on debt for multiple governments reflect the debt measured for all fiscal years of the governments being summed. Since these fiscal years differ, total statistics (such as for all local governments in a state, or all townships nationally) reflect a mix of fiscal periods. The Census Bureau makes no effort to adjust aggregates so that they represent a standard time period, at least for annual surveys.

6.3 Statistical Classification Categories for Debt

The following pages contain the classification categories and their definitions for statistics on government debt, as currently in use by the Census Bureau. These categories, consisting of fifteen variables, became effective with the 2005 survey of state and local government finances. Ten of the variables apply to state and local governments, while five apply to Federal Government statistics.

The pages contain all of the debt statistics categories – regular, exhibit, and derived. There are no descriptive statistics for debt (See Chapter 2 for an explanation of these types within the Census Bureau's statistical program). Each category is defined and explained, with examples provided where appropriate. The categories for regular statistics are presented in terms of the three way classification currently in use by the Census Bureau:

By Length of Term Outstanding:	By Type:	By Purpose:
- Short-Term	- Beginning	- Public Debt For Private Purposes
- Long-term	- Issued	- Public Debt, Unspecified Public Purposes
	- Retired	
	- Outstanding	

The categories for exhibit statistics are part of the official Census Bureau classification system. As such, these statistics are assigned to specific government units, as applicable, and can be used in aggregate statistics by level and type of government. There are two exhibit debt statistics in use in the current Census Bureau program on government finance statistics, both applicable only to state governments.

The categories for derived debt statistics are calculated using regular Census Bureau debt statistics. However, they are not intended to represent accounting identities that can be used to measure a government's (or governments') financial position. Some of these also use data gathered on cash and security holdings. Note that there are no census codes associated with these types of statistics. There are four derived debt statistics in use in the current Census Bureau program on government finance statistics.

GOVERNMENT DEBT CATEGORIES

SHORT-TERM DEBT – State and Local Government Statistics

Definition: Interest-bearing debt payable one year or less from its date of issue. Includes obligations having no fixed maturity date (even where outstanding for more than one year if payable from a tax levied for collection in the same year it was issued).

Includes: Bond anticipation notes, tax anticipation notes and warrants, bank loans, tax-exempt commercial paper, interest-bearing short-term warrants and obligations, and revenue anticipation notes (RANS).

Excludes: Accounts payable, non-interest-bearing warrants and obligations, interfund loans (non-liabilities), and long-term debt payable within the current fiscal year (report at *Long-term Debt*).

Applicable Classification Codes:

61V – Short-Term Debt Outstanding, Beginning Of Fiscal Year
64V – Short-Term Debt Outstanding, End Of Fiscal Year

Special Considerations:

1. Short-Term debt is not further classified beyond the first category of "length of term," in contrast to long-term debt.
2. Short-term debt is measured at two points in time for each government – start of fiscal year and end of fiscal year. Short-Term debt issued and retired during the year thus are not recorded in Census Bureau debt statistics for governments.
3. This category does not apply to statistics on Federal Government debt.

Applicability By Level and Type of Government

Federal	State	Local
No	Yes	Yes – All Types

GOVERNMENT DEBT CATEGORIES

LONG-TERM DEBT – State and Local Government Statistics

Definition: Debt payable more than one year from its date of issue, including those redeemable in less time under a "daily put option" (option tender bonds).

> **Includes:** General obligation bonds (GOB), term bonds, serial bonds, revenue bonds, industrial revenue bonds, pollution control bonds, special assessment bonds, certificates of participation (COPs), zero coupon or compound interest bonds, judgments, mortgages, and construction loan notes (CLNs).
>
> **Excludes**: Leases (operating or capital), interfund loans (except own securities held as investments by other funds, such as employee retirement systems), amounts owed in an agency or trust capacity (e.g., employee tax withholdings), interest payable, advances and contingent loans from other governments, unfunded obligations of employee pension funds, accrued vacation or sick leave, and rights of individuals to benefits from insurance trust systems.
>
> **Examples:** Public Debt for Private Purposes – often referred to as "conduit debt" in Comprehensive Annual Financial Reports of governments.

Special Consideration:
Treat tax anticipation warrants or notes outstanding for more than one year as short-term debt if they are payable from a specific tax levied for collection in same year debt was issued.

Applicable Classification Codes

19T Beginning Long-term Debt Outstanding, Public Debt For Private Purposes
19U Beginning Long-term Debt Outstanding, Unspecified Public Purposes

24T Long-term Debt Issued, Public Debt For Private Purposes
29U Long-term Debt Issued, Unspecified Public Purposes

34T Long-term Debt Retired, Public Debt For Private Purposes
39U Long-term Debt Retired, Unspecified Public Purposes

44T Long-term Debt Outstanding, End Of Fiscal Year, Public Debt For Private Purposes
49U Long-term Debt Outstanding, End Of Fiscal Year, Unspecified Public Purposes

Applicability By Level and Type of Government

Federal	State	Local
No	Yes	Yes – All Types

GOVERNMENT DEBT CATEGORIES

LONG-TERM DEBT – Federal Government Statistics

Definition: All debt, regardless of length of term, of the parent government, all its agencies and trust funds, and all Federally-backed organizations (as defined by the Census Bureau for statistical purposes).

Includes: See specific descriptions below.

Excludes: Debt of associated international agencies, such as the International Monetary Fund.

Special Considerations:

1. Debt statistics collected for the Federal Government are limited to several outstanding codes that neither distinguish between short- and long-term nor indicate functional use.
2. These debt classification codes apply only to Federal Government statistics.
3. Note the overlap in these debt codes: total debt outstanding is equal to both the sum of 41V plus 41Y and the sum of 41Q plus 41W.

Applicable Classification Codes:

41I Increase in debt during the fiscal year (debt outstanding at end of current fiscal year minus debt outstanding at end of prior fiscal year).

41V Debt held by Federal Government accounts, primarily Federal insurance trust funds (e.g., Social Security, Medicare, as well as Military and Civil Service retirement trusts, including Federal Employee Retirement System).

41Y Debt held by persons and organizations outside the Federal Government (including Federal Reserve Banks holdings of Federal securities purchased for monetary policy reasons).

41Q Debt obligations of the U.S. Treasury, including short-term notes and Treasury borrowing on behalf of the Federal Financing Bank.

41W Debt obligations of Federal agencies, such as the Federal Savings and Loan Insurance Corporation (FSLIC), Federal Housing Administration (FHA), Federal Deposit Insurance Corporation (FDIC), Postal Service, and Tennessee Valley Authority (TVA). Excludes agency borrowing from the Federal Financing Bank and debt of government-sponsored enterprises (e.g., Federal National Mortgage Association, Federal Housing Finance Board, and Farm Credit Administration).

Applicability By Level and Type of Government

Federal	State	Local
Yes	No	No – All Types

GOVERNMENT DEBT CATEGORIES

LONG-TERM DEBT: EXHIBIT STATISTICS ON REFUNDING DEBT

Definition: The issuance of long-term obligations in exchange for, or to finance the retirement of, existing long-term debt.

Includes: Regular refunding and advance refunding (see Section 6.4.2).

Excludes: Short-Term debt issued as a temporary measure to pay off existing long-term debt service.

Applicable Classification Codes:

52T Long-term Refunding Bonds Issued During The Fiscal Year
53T Long-term Debt Retired By Refunding During The Fiscal Year

Special Considerations:

1. These classifications were first used in the 1979 survey year.
2. Prior to 2005, these codes applied to selected, large local governments.
3. Prior to 2005, the Census Bureau used two additional exhibit codes for cash and securities (assets) that were related to debt statistics. These were 71W (offsets to full-faith and credit long-term debt) and 74W (offsets to nonguaranteed long-term debt). These codes were used for state and large local governments.

Applicability By Level and Type of Government

Federal	State	Local
No	Yes	No – All Types

GOVERNMENT DEBT CATEGORIES

DERIVED DEBT STATISTICS – BORROWING

Definition: *Borrowing* is an estimate of the net amount of new money that a government has borrowed during the fiscal year, including short- and long-term debt. It consists of the par value of long-term debt issued during the year (other than for refunding purposes) plus any net increase in short-term debt between the beginning and end of the fiscal year. Note that it does not reflect the total amount of short-term debt sold during the year.

Calculation: Shown as a formula using Census Bureau 3-digit debt codes, borrowing is computed as follows (the asterisk (*) in the formulas below stands for the appropriate function code for the debt issued or retired):

If net short-term debt has **increased** during the fiscal year:
Current classification system = 24T + 29U + (64V – 61V) – 52T
Pre 2005 classification system = 21* + 24* + 29* + (64V – 61V) – 52T

If net short-term debt has **decreased** (or remained constant) during the fiscal year:
Current classification system = 24T + 29U – 52T
Pre 2005 classification system = 21* + 24* + 29* – 52T

Special Consideration:
When calculated, these statistics apply only to state governments and their aggregates. There is no applicability to Federal Government statistics.

GOVERNMENT DEBT CATEGORIES

DERIVED DEBT STATISTICS – REDEMPTION

Definition: *Redemption* is an estimate of the net amount of debt that a government has paid off during the fiscal year, including short- and long-term debt. It consists of the par value of long-term debt retired during the year (other than debt retired by refunding) minus any net decrease in short-term debt between the beginning and end of the fiscal year.

Calculation: Shown as a formula using Census Bureau 3-digit debt codes, redemption is computed as follows:

If net short-term debt has **increased** during the fiscal year:
Current classification system = 34T + 39U – 53T
Pre 2005 classification system = 31* + 34* + 39* – 53T

If net short-term debt has **decreased** (or remained constant) during the fiscal year:
Current classification system = 34T + 39U + (64V – 61V) – 53T
Pre 2005 classification system = 31* + 34* + 39* + (64V – 61V) – 53T

Special Considerations:

1. Redemption does not reflect the total amount of short-term debt paid off during the year.
2. Note that debt redemption includes debt redeemed not only from current revenue or prior year fund balances but also from the sale of assets accumulated in debt service funds (sinking funds). The transfer of current revenue to such funds for future debt service is considered an intragovernmental transaction and, therefore, is not included in either revenue or expenditure statistics.
3. When calculated, these statistics apply only to state governments, and their aggregates. There is no applicability to Federal Government statistics.

GOVERNMENT DEBT CATEGORIES

DERIVED DEBT STATISTICS – CHANGE IN DEBT

Definition: Change in debt is an estimate of the net change in a government's indebtedness during the fiscal year. It consists of the par value of long-term debt issued during the fiscal year less the par value of long-term debt retired during the year plus (or minus) the change in short-term debt between the beginning and end of the fiscal year.

Calculation: Shown as a formula using Census Bureau 3-digit debt codes, change in debt is computed as follows:

Current classification system = 24T + 29U – 34T – 39U + (64V – 61V)
Pre 2005 classification system = 21* + 24* + 29* – 31* – 34* – 39* + (64V – 61V)

Special Considerations:

1. When calculated, these statistics apply only to state and local governments, and their aggregates. There is no applicability to Federal Government statistics.
2. "Change in debt" does not reflect the total amount of short-term debt sold or paid off during the year.

GOVERNMENT DEBT CATEGORIES

DERIVED DEBT STATISTICS – NET LONG-TERM DEBT OUTSTANDING

Definition: The amount of long-term debt held by a government for which no funds have been set aside for its repayment. It consists of total long-term debt outstanding less total offsets to debt (i.e. cash and security holdings in debt service or sinking funds).

Calculation: Shown as a formula using Census Bureau 3-digit debt codes, net long-term debt outstanding is computed as follows:

Current classification system = 44T +49U –W01
Pre 2005 classification system = 41* + 44* – W01

Special considerations:

1. When calculated, these statistics apply only to state and local governments, and their aggregates. There is no applicability to Federal Government statistics.
2. W01 is not a debt code, but rather a code used in the statistics for *Cash and Security Holdings – Offsets to Debt.* See Chapter 7.

6.4 Topical Issues Related to Statistics on Government Debt

The following sections contain additional explanations of some of the conceptual issues pertaining to how the Census Bureau covers government debt in its government finance statistical program.

6.4.1 Public Debt for Private Purposes

This functional debt category, first used in the fiscal year 1988 survey, represents a consolidation of two former categories plus certain debt from others. It now represents one of the largest categories of state and local government debt outstanding.

Public debt for private purposes comprises credit obligations of a government or any of its dependent agencies for the purpose of funding private sector activities,[2] including debt that is backed solely by the private organization(s) whose activity is being financed. Government accountants often refer to this as Conduit Debt in official records, such as CAFRs. The Census Bureau assigns this debt to the government whose bond-issuing authority was used to secure its tax-exempt status or, in the case of taxable debt, was used for its issuance. Examples of private sector activities funded include:

- Industrial and commercial development
- Pollution control and abatement
- Housing and mortgage loans
- Private hospital facilities
- Student loans
- Private ventures such as sports stadiums, convention centers, and shopping malls.

Historically, this type of debt has presented certain data collection problems for the Census Bureau. Often, it was not listed in the financial statements of the issuing government. In these cases, Census analysts relied on secondary sources to "discover" such debt. Even where its issuance was identifiable, its retirement schedule, interest payments, and amounts outstanding were often unavailable. This often required the Bureau to estimate the statistics. The governments' themselves often did not construe such debt to be their own and objected to its being included in Census Bureau statistics about their finances. One of the objections government officials had was that this debt could distort the presentation of data, such as its effect on per capita debt when a small government issues a large amount of public debt for private purposes.

In recent years, however, GASB rules have addressed government reporting of public debt for private purposes. Statistics on such debt are now much more readily available in government fiscal reports, such as their CAFR's. The Census Bureau continues to include this debt as in-scope in terms of coverage for its statistical program on government finances.

---

[2] Users should note that this statistical category is broader than the "private activity bonds" that are regulated by such Federal laws such as the Tax Reform Act of 1984, which excludes such debt as private multi-family rental housing and mortgage subsidy bonds that are included in the Census Bureau category.

Public debt for private purposes also generates special treatment regarding its related revenue, expenditure, and even cash and security holdings. This treatment was revised for the fiscal year 1988 survey to cover all types of similar debt. Previously, it was limited to mortgage revenue debt (the old "W" code debt in Table 6.2).

- Revenue: An amount equal to the interest expenditure (*Interest on General Debt*, code I89) on all public debt for private purposes is classified as *Interest Earnings*, code U20.

- Expenditure: Unlike all other forms of debt issued, no expenditures from the proceeds of public debt for private purposes are reported. The actual (or estimated) interest payments on such debt are classified as *Interest on General Debt*, code I89.

- Cash and Security Holdings: An amount equal to the public debt for private purposes outstanding at the end of the fiscal year is classified as an *Offset to Debt* (code W01). This figure is revised annually to account for amounts retired or issued.

6.4.2 Refunding of Long-Term Debt

Governments often retire debt before it matures by issuing more debt at a lower interest rate, yielding savings in the form of lower debt service costs. A variation of this type of transaction is "advance refunding," where a government issues new debt but sets aside the proceeds rather than actually paying off the old debt. The old debt, in turn, is "defeased" and removed from the government's accounting statement. The next two sections describe how this transaction is classified in Census Bureau statistics.

6.4.2.1 Regular Refunding

Regular (or direct) refunding refers to the issuance of long-term obligations in exchange for, or to finance the retirement of, existing long-term debt, typically on or after the first call date of the debt to be refunded. This rather straightforward transaction is classified for Census Bureau statistics as described below:

- *Refunding debt issued:* The par value of debt issued during the fiscal year is reported twice: (1) under regular *Debt Issued*, codes 24T or 29U, and (2) under *Refunding Debt Issued*, code 52T, the exhibit code. (Note that the "T" in code 52T does not refer to public debt for private purposes.) Any amounts authorized but not actually issued are excluded.

- *Debt retired by refunding:* The par value of debt retired by refunding during the fiscal year is reported twice: (1) under regular *Debt Retired*, codes 34T or 39U, and (2) under the exhibit code *Refunding Debt Retired*, code 53T.

- *Refunding debt outstanding:* The par value of debt outstanding that was issued for refunding purposes is reported under regular *Debt Outstanding*, codes 44T or 49U. The debt that has been retired by the refunding bonds is excluded.

6.4.2.2 Advance Refunding

A more complex situation occurs when a government issues refunding debt but cannot legally retire the old debt under the terms of the original debt issuance. Typically the original debt's "first call" date, usually a term of 10 years, has not yet been reached. "Advance refunding" generally occurs when interest rates are falling dramatically. In these cases, the government places the proceeds of the refunding bonds in escrow. The proceeds must include enough monies to cover the debt service (principal and interest) until the original debt's first call date is reached, at which time the funds in escrow are used to retire the original debt's remaining balance.

The government generally has two choices on treating the refunded debt. One, if the refunding debt issued is sufficient to pay the remaining principal and all future interest on the original debt, then the government can remove the original debt from its balance sheet, an action called defeasance. For purposes of Census Bureau statistics, the debt is defeased whether or not the government is released from its legal obligation for the debt (legal defeasance) or remains the primary obligor (in-substance defeasance).

Two, the government may use the escrow funds to pay the interest and principle due on the refunding debt until a certain date is reached, at which time the escrowed money is used to retire the original debt, which is then defeased. This type of advance refunding is called "crossover" refunding.

This classification structure and subsequent treatment was adopted first with fiscal year 1979 data. At that time, the Census Bureau made a one-time adjustment of –$5.6 billion to state and local government long-term debt outstanding for that year to accomplish the revision. The negative adjustment reflected the fact that debt that was defeased had to be removed from the statistical measure in accordance with the Census Bureau's new definition.

6.4.3 Zero-Coupon and Other Deep Discount Bonds

Deep discount bonds and their equivalents (zero-coupon bonds, compound interest bonds, etc.) are debt instruments sold at a price much below their face value. The interest they earn is added to the value of the bond rather than paid out serially (similar to how U. S. savings bonds work). Stated formally, interest is reinvested, compounded at the original rate that applied to principal, and paid at maturity.

For Census Bureau statistics, deep discount debt transactions are reported as follows:

Debt issued: Only the actual proceeds from the sale of the bonds are reported as long-term debt issued (i.e., not the higher face value amount).

Debt retired: When the bonds are finally paid off, only the original sale price is reported as long-term debt retired (i.e., not the higher face value which includes accumulated interest). Thus, the amount retired is equal to the amount originally reported as issued.

Debt outstanding: The amount of proceeds from the original sale of the bonds is reported as long-term debt outstanding (i.e., the outstanding amount is not incremented by the interest earned and added to its face value). Thus, the amount outstanding remains constant during the life of the bond.

Interest expenditure: The amount of interest earned by the bond and added to its face value is reported as interest expenditure (even if it does not involve actual cash disbursements).

6.4.4 Bond Banks and Pooled Debt

Governments sometimes issue debt jointly rather than individually, to reduce the cost of issuing debt and to achieve a lower interest rate. For instance, a state government may create a "bond bank" that issues debt in the state's name, with the proceeds then used to purchase securities from local governments. All of the state debt is "U" debt since the debt is for public purposes (borrowing by local governments in the state). Local governments also can create their own bond banks or enter into "pooled debt" arrangements where one member issues debt and others borrow from the proceeds.

For Census Bureau statistics, these debt transactions are reported as follows:

> *Debt issued, retired, and outstanding:* The government in whose name the bond bank or pooled debt was issued is recorded with the amount issued, retired, and outstanding using regular census debt codes.
>
> *Loaning of proceeds to other governments:* The distribution of the proceeds from the bond bank or pooled debt is not reported as an intergovernmental transaction. Instead, for the government issuing such debt, the purchase of another government's securities or the borrowing of debt proceeds by other governments is reported as an investment under *Offsets to Debt*, code W01. For the governments whose securities are sold or which borrows the proceeds, it is treated a *Long-Term Debt Issued*, codes 24T or 29U.
>
> *Repayment of proceeds:* When the member government repays part or all of the loan from the issuing government, it is reported as long-term debt retired by the former. For the issuing government, the amount repaid each year reduces the security holding for *Offsets to Debt*, code W01.
>
> *Interest on debt:* For the issuing government, the interest paid on the debt is reported under the appropriate *Interest Expenditure* category (I89, I91, I92, I93, or I94). For the member governments, it is treated as in intergovernmental expenditure (and for the issuing government as intergovernmental revenue).

Note that in some cases, the original debt is issued by a non-public entity (such as a league of cities) that operates more like the joint activities described in Section 1.5. In these cases, the debt and its related transactions are assigned to the member governments and reported as regular long-term debt.

An example of a bond bank is the Indiana Bond Bank. The state issues bonds or otherwise allocates funds for the express purpose of creating a pool of money from which local governments can borrow. The resulting financial transactions are reported as indicated above, for the state and the participating local governments.

6.4.5 Leases and Lease-Purchase Agreements

For Census Bureau statistics, leases, lease-purchase arrangements, lease-rental agreements, and the

like are not considered public debt.[3] Instead, payments on them are reported as capital outlay (see Section 3.11.2 for details).

A government may enter into a leasing arrangement with a private firm in lieu of issuing debt and building their own facilities. The private firm obtains funding for the project, builds it, and leases the facility or equipment back to the government. For the government, leases offer the advantages of not requiring voter approval and are not counted toward its debt limitation ceiling.

Note that Census Bureau debt categories do include lease-rental bonds issued by a dependent agency of a government that builds a facility, which it leases back to the parent government. These lease payments would not be reported since they represent intragovernmental transfers.

6.4.6 Taxable Public Debt

Most government debt is tax-exempt. The interest paid to bondholders is exempt from Federal income taxes. In some cases, however, a government will issue taxable debt, where the interest paid to bondholders is not exempt from Federal taxation. Although relatively uncommon, governments might issue debt that does not receive exemption from Federal taxes to bypass debt limitations imposed on the issuing government. For Census Bureau statistics, taxable public debt is reported in the same manner as tax-exempt public debt.

6.4.7 Special Topics: Debt Statistics and the Four Sectors of Government

Previous versions of this *Classification Manual* contained separate classification categories for utilities sector debt and general government sector debt, as noted previously. However, the Census Bureau classification system never has had separate debt categories for all four sectors of government as described in Chapter 2 – general, utilities, social insurance trusts, and liquor stores.

For social insurance trusts, any debt would be issued by the parent government, thereby making it part of the general sector. For public employee retirement systems, the Census Bureau does not classify (measure) unfunded liabilities, so that this aspect of retirement system finances is not included in the debt statistics. Where "borrowing" occurs for purposes of unemployment compensation systems, in the form of funding extended benefits, such transactions are treated as revenue (positive or negative). See Chapters 8 and 9 for additional information.

Debt would be very rare for liquor stores. If there is any debt issued, it is the responsibility of the parent government. Again, this falls into the general government sector.

6.5 Tables

Table 6.1 shows which debt categories (codes) are applicable by level and type of government, effective with the 2005 survey year. Table 6.2 shows the current codes in use for Census Bureau statistics on

---

[3]Leases are not classified as debt for Census Bureau purposes for various reasons. Unlike bonded debt, leases rarely generate any cash flow. With no proceeds available, there are no funds to expend on capital outlays or to turn over to the private sector in return for an investment security. Moreover, leases are rarely negotiable instruments, do not require voter approval or apply to debt ceiling limits, are funded by annual appropriations rather than dedicated taxes or other revenue sources (in effect, making them renewable one-year contracts), can be canceled in some cases, and often have an "interest" component that is simply an imputed amount. Thus, for Census Bureau statistics, leases are closer to the "pay-as-you-go" way of financing capital improvements than to debt.

government debt, along with the equivalent codes from previous versions of the Census Bureau classification system. It can be used to develop comparative aggregates of time series data.

Table 6.1
Applicability of Debt Categories and Codes

Category	Code	Federal	State	Local Governments – By Type				
				County	Municipal	Township	Special District	School District
Short-Term Beginning	61V	X	Valid	Valid	Valid	Valid	Valid	Valid
Short-Term Ending	64V	X	Valid	Valid	Valid	Valid	Valid	Valid
Long-term Beginning – Private Purposes	19T	X	Valid	Valid	Valid	Valid	Valid	Valid
Long-term Beginning – Unspecified Public Purposes	19U	X	Valid	Valid	Valid	Valid	Valid	Valid
Long-term Issued – Private Purposes	24T	X	Valid	Valid	Valid	Valid	Valid	Valid
Long-term Issued – Unspecified Public Purposes	29U	X	Valid	Valid	Valid	Valid	Valid	Valid
Long-term Retired – Private Purposes	34T	X	Valid	Valid	Valid	Valid	Valid	Valid
Long-term Retired – Unspecified Public Purposes	39U	X	Valid	Valid	Valid	Valid	Valid	Valid
Long-term Outstanding – Private Purposes	44T	X	Valid	Valid	Valid	Valid	Valid	Valid
Long-term Outstanding – Unspecified Public Purposes	49U	X	Valid	Valid	Valid	Valid	Valid	Valid
Exhibit: Refunding Debt Issued	52T	X	Valid	X	X	X	X	X
Exhibit: Debt Retired by Refunding	53T	X	Valid	X	X	X	X	X
Federal – Increase in Debt	41I	Valid	X	X	X	X	X	X
Federal – Debt Held by Federal Accounts	41V	Valid	X	X	X	X	X	X
Federal – Debt Held by Outside Persons/Agencies	41Y	Valid	X	X	X	X	X	X
Federal – Debt Obligations of US Treasury	41Q	Valid	X	X	X	X	X	X
Federal – Debt Obligations of Federal Agencies	41W	Valid	X	X	X	X	X	X

Key:

X — Not a valid code for the level or type of government indicated (there are no statistics).

Valid — Code is applicable for the level or type of government indicated (statistics are collected and reported).

**Table 6.2 Historical Crosswalk: State and Local Debt Classification Categories** – page 1 of 2			
General Description of Debt Category	Current Codes (effective 2005)	Equivalent Codes From 1988 to 2004	Equivalent Codes Pre-1988
STATE AND LOCAL SHORT-TERM DEBT:			
**Short-Term, Beginning**	**61V**	61V	61V
**Short-Term, Ending**	**64V**	64V	64V
STATE AND LOCAL LONG-TERM DEBT – BY TYPE:			
**LT Begin, Public Debt for Private Purposes**	**19T**	19T	19T, 19W
**LT Begin, Unspecified Public Purposes**	**19U**	19*	19*
**LT Issued, Public Debt for Private Purposes**	**24T**	24T	24T, 24W
**LT Issued, Unspecified Public Purposes**	**29U**	21*, 24*, 29*	21*, 22*, 23* 24*, 29*
FFC Issued	None	21*	21*
FFC Issued, General Obligation	None	None	22*
FFC Issued, Nontax	None	None	23*
NG Issued	None	24*	24*
Unspecified Issued	None	29*	29*
**LT Debt Retired, Public Debt for Private Purposes**	**34T**	34T	34T, 34W
**LT Debt Retired, Unspecified Public Purposes**	**39U**	31*, 34*, 39*	31*, 32*,33*, 34* 39*
FFC Retired	None	31*	31*
FFC Retired, General Obligation	None	None	32*
FFC Retired, Nontax	None	None	33*
NG Retired	None	34*	34*
Unspecified Type, Retired	None	39*	39*
**LT Outstanding, Public Debt for Private Purposes**	**44T**	44T	44T, 44W
**LT Outstanding, Unspecified Public Purposes**	**49U**	41*, 44*	41*, 42*, 43*, 44*
FFC Outstanding	None	41*	41*
FFC Outstanding, General Obligation	None	None	42*
FFC Outstanding, Nontax	None	None	43*
NG Outstanding	None	44*	44*

Table 6.2 Historical Crosswalk: State and Local Debt Classification Categories – page 2 of 2			
General Description of Debt Category	Current Codes (effective 2005)	Equivalent Codes From 1988 to 2004	Equivalent Codes Pre-1988
STATE AND LOCAL LONG-TERM DEBT – BY PURPOSE:			
**Public Debt for Private Purposes**	****T**	**T	**T, **W
**Public Debt, Unspecified Public Purposes**	****U**	**A, **B, **C, **D, **F, **G, **H, **X	**A, **B, **C, **D, **E, **F, **G, **H, J**, K**, L**, M**, N**, P**, R**, S**, T**, **W, **X
Water Utilities	None	**A	**A
Electric Utilities	None	**B	**B
Gas Utilities	None	**C	**C
Transit Utilities	None	**D	**D
El-Sec Education	None	**F	**F
Higher Education	None	**G	**G
Other Education	None	**H	**H
Not Elsewhere Classified	None	**X	**X
Airports	None	None	**E
Hospitals	None	None	**J
Regular Highways	None	None	**K
Toll Highways	None	None	**L
Housing and Community Development	None	None	**M
Parks and Recreation	None	None	**N
Sewerage	None	None	**P
Veterans' bonuses	None	None	**R
Sea and Inland Port Facilities	None	None	**S
Industrial Development and Pollution Control	None	None	**T
Mortgage Revenue	None	None	**W

Abbreviations:
LT = Long-term
FFC = Full faith and credit (guaranteed debt)
Nontax = Guaranteed debt, but pledged initially from nontax revenue
General Obligation = Guaranteed debt, pledged from taxing authority
El-Sec = Elementary and Secondary Education

Chapter 7. Cash and Security Holdings

Introduction

Governments possess numerous financial and capital assets. Financial assets are represented by the cash and security holdings of the government and its dependent agencies. Capital assets consist of the buildings, land, and infrastructure (highways, sewer lines, and so forth) owned by the government. In the Census Bureau's classification system on government finances, only financial assets are included in the statistics.[1] They are labeled as "Cash and Security Holdings." This chapter contains explanations of how the Census Bureau defines and classifies these statistics.

7.1 Cash and Security Holdings Definition

Census Bureau statistics on government assets include only cash and security holdings. The definition covers cash on hand, demand or time deposits, savings accounts, government securities (of Federal, state, and local governments), and private securities (bonds, notes, mortgages, corporate stocks, etc.). Also included are loans and other credit paper held by government loan and investment funds. Table 7.1 contains a listing of the more common types of assets found in government accounting records, and their status (in-scope or out-of-scope) with respect to the Census Bureau's classification categories. In summary, the following are included:

- Cash and Short-Term Deposits
- Government Securities:
  - Federal Government Securities
  - Federal Agency Securities
  - State and Local Government Securities
- Private Securities:
  - Corporate Bonds
  - Corporate Stocks
  - Mortgages Held Directly (by a government as an investment)
  - International Securities
  - All Other Securities, NEC

The definition explicitly excludes other types of assets often reflected in the accounting records of a government. In this regard, the Census Bureau definition is more limited than some other statistical measures of a government's financial resources, because it does not include physical plant and other types of assets (see Section 7.1.1).

As with other Census Bureau statistics on government finances and employment, the concept includes cash and security holdings of the general government as well as of the government's utility, liquor stores, and insurance trust sectors. The statistics represent the parent government and also the assets of all autonomous agencies, boards, commissions, or other organizations classified as dependent on the parent government. Statistics on individual governments include the combined assets of each parent government and its dependent agencies. Aggregate statistics, including those by type of government, level of government, or geographic area, represent the sum of the appropriate individual parent governments and their respective dependent agencies.

[1]Capital assets are measured indirectly, by the level of expenditure for such activities during each fiscal year, as noted in Chapter 5. However, the classification system excludes the standing value of such assets and also excludes the amount of depreciation attributable to them.

7.1.1 Assets Outside of Census Bureau Definition

Unlike the other three major types of finance data collected by the Census Bureau, the Bureau's concept of cash and security holdings is an exclusive one, limited to the most fluid types of assets governments own.

The following types of assets are excluded from Census Bureau statistics on governments:

- Real property, land, buildings, improvements, machinery, equipment, and other fixed assets.[2]
- Construction in progress.
- Materials, supplies, and inventories.
- Accounts receivables, taxes receivables, special assessment receivables, interfund receivables, tax liens, advances to other funds, and other such monies owed to a government.
- Unamortized premiums on investments.
- Prepaid or deferred expenses.
- Goods or commodities held for resale (e.g., of liquor stores or utilities).
- Intangible assets.
- Assets held by private trust funds or by funds representing agency transactions (e.g., withholdings of employee Social Security taxes).

See Table 7.1 for additional detail.

7.2 Measurement Issues

There are two important conceptual issues related to statistics on cash and security holdings of governments. Unlike revenue and expenditure, such financial assets are measured at a particular point in time, namely the close of the fiscal year. Also, the Census Bureau must determine how to value the financial assets, for statistical purposes. Recent changes in government accounting standards have influenced this aspect of the statistical program and are explained in Section 7.2.2.

7.2.1 Measurement Issues: Timing

There are two important considerations in the Census Bureau methodology for reporting statistics on government assets – timing and valuation. Each of these presents measurement problems that require resolution for statistical purposes.

The timing problem relates to a government's fiscal year, since the dollar value of assets fluctuates

[2] Data on real property holdings are collected for the survey of public employee retirement systems, using special exhibit codes (see Chapter 8 for details).

during the fiscal year period. To overcome this problem, the Census Bureau definition uses the last day of each government's fiscal year for measuring assets. This definition has been used consistently in the Census Bureau's statistical program, so that all historical data on government assets have been measured in the same manner (with respect to timing) over the life of the statistical program.

The approach to resolving the timing problem is similar to that used to measure government debt (see Chapter 6), although the latter statistics also include measures of debt issued and retired during the fiscal year. There are no similar variables for asset changes during the fiscal year in the Census Bureau's statistical classification system. There are, however, statistics that cover the gain or loss on investment transactions during the year for public employee retirement systems, as discussed later and in Chapter 8.

Aggregate statistics on government assets, such as by state area, necessarily represent a mix of time points that depend on the fiscal years of the governments included in the aggregation. Even within a government unit, some dependent agencies could have a fiscal year that differs from that of the parent government. The rule for measuring assets at the end of the fiscal year still applies in such cases, however, even for the component unit with a different fiscal year.

7.2.2 Measurement Issues: Valuation

Assigning a value to assets is one of the most complex aspects of the Census Bureau's program on government finance statistics. As discussed in Section 3.12, the Census Bureau program is first and foremost statistical in nature and is not intended to reflect the same types of measures developed for purely accounting purposes. Nevertheless, the Census Bureau uses standard accounting records to develop its government finance statistics, so accounting definitions and concepts must be taken into consideration.

Consequently, Census Bureau statistics are impacted by accounting industry changes such as the GASB guidelines noted throughout this *Classification Manual.* GASB Statement 34 (issued in 1999) had a major impact on the way governments (and especially government administered employee retirement systems) valued their assets for accounting purposes. The Census Bureau decided to adopt GASB standards for reporting the value of assets that were in-scope for Census Bureau surveys.[3] This change was phased in over two stages, as summarized below. Appendix 1 contains a more lengthy explanation of this change to the definition used in the Census Bureau's statistical program.

This edition of the *Classification Manual* contains this major change to asset valuation. It was first applied to statistics effective with the 2002 Census of Governments. At that time, the Census Bureau began to use the market value of corporate stocks, corporate bonds, and international securities held by public employee retirement systems. Prior to the 2002 Census of Governments, Census Bureau statistics for these three asset types represented the book value (cost) of the asset. For assets other than corporate stocks, corporate bonds, and international securities, the statistics continued to reflect the book value (cost) of the holdings.

Then, effective with this edition of the *Classification Manual* and with government finance statistics for 2005, market value became the basis of measurement for all government cash and security

[3] The Census Bureau obtained approval for this change in definition from the Office of Management and Budget. The Census Bureau also sought and received concurrence from two major users affected by the change, the Federal Reserve Board and the Bureau of Economic Analysis.

holdings. Prior to 2005, these assets were valued using a mix of concepts, but primarily using book value (cost) of the asset. Appendix Table 1.1 in Appendix 1 provides an explanation of the historical changes to the valuation method applied to cash and security holdings for the three time periods:

2005 to Current
2002 through 2004
Pre - 2002

The Census Bureau made no adjustment to historical data on assets to reflect this change in definition. This was because of the difficulty of obtaining reliable statistics on the differences in reporting at the government unit level. Analysts noted that some components units of governments, particularly employee retirement systems, had been changing to market valuation in their reporting for Census surveys.

Market value of cash and security holdings is defined in two ways, depending on the type of asset. For cash and cash-based short-term investments such as certificates of deposit, the valuation is based on current dollar value of the holdings. For all other security holdings, market value is the amount that a government can reasonably expect to receive for an investment in a current sale between a willing buyer and willing seller (other than in a force or liquidation sale). This definition is essentially the same as found in GASB statement 25. In both cases, the measure is taken as of the close of the government's fiscal year. Market value also is referred to as "fair value" in many accounting references.

7.3 Special Topics: Assets of State Lottery Systems

Assets held in funds of state-operated lottery systems present a special classification case. Lottery system assets that are held for future distribution of prize monies are excluded from the Census Bureau statistics on cash and security holdings. In effect, they are treated as agency funds, where the state has no discretion as to disbursement of the funds. Such funds are held in trust for the prize winners (usually those persons who choose to receive their winnings over a period of time, rather than in one lump sum). Similarly, interest earnings on such lottery funds held in trust are excluded from the Census Bureau statistic *Interest Earnings*, code U20.

This treatment of lottery system finances is consistent within the Census Bureau classification system, where code U95 represents *Net Lottery System Revenue* (see Chapter 4). See also Chapter 10 for additional information on classifying lottery financial activity.

7.4 Special Topics: Federal Government Cash and Security Holding Statistics

Census Bureau statistics on government finances do not include measures of cash and security holdings of the Federal Government. The Federal Government is included in the classification system (and data exist) for the other three general areas of financial coverage – revenue, expenditure, and indebtedness. However, the area of cash and security holding never has been covered in the classification system, nor in the actual data compiled and released to the public.

7.5 Special Topics: Liquor Stores Cash and Security Holdings

Even though liquor stores constitute one of the four sectors of government for purposes of regular Census Bureau statistics on government finance, there are no separate cash and security holding statistical

categories for liquor stores. Instead, any such assets of state or local liquor store operations are reported in the general government sector at the appropriate code, usually at *All Other Holdings,* code W61.

7.6 Statistical Classification Categories for Cash and Security Holdings

The Census Bureau uses a two-way classification system for statistics on government cash and security holdings – By Purpose and By Type. More specifically, the “By Purpose” classification categories (and sub-categories) apply to the overall government and cover all sectors. However, within the social insurance trust government sector, assets also are classified “By Type” for two sub-sectors – government administered public employee retirement systems and the state-administered unemployment compensation systems. This special classification treatment reflects the primary use for which these statistics are collected. Employee retirement systems hold over two trillion dollars, and economists have a long-standing interest in knowing how these systems are investing their assets. Consequently, the Census Bureau uses additional statistical categories, patterned after accounting standards, to classify assets of these retirement systems. Chart 7.A on the next page summarizes this classification topic.

Chart 7.A Statistical Classification Categories for Cash and Security Holdings	
**Classification By Purpose (applies to all Census Bureau statistics on Cash and Security Holdings)**	**Classification By Type of Holding**
Other Than Social Insurance Trust Systems:	
Sinking Funds	Not applicable
Bond Funds	Not applicable
Other Funds	Not applicable
Social Insurance Trust Systems:	
Unemployment Compensation Systems	- Cash in Trust Fund Account of US Treasury<br>- Cash in Clearing and Special Benefit Account of US Treasury
Workers Compensation Systems	Not applicable
Other State Social Insurance Trust Systems	Not applicable
Employee Retirement Systems	Cash and Short-Term Investments<br>- Cash and Demand Deposits<br>- Time or Saving Deposits<br>- All Other Short-Term Investments<br>Total Federal Government Securities<br>- Federal Treasury Securities<br>- Federal Agency Securities<br>Total Corporate Bonds<br>- Federally-Sponsored Agencies<br>- Corporate Bonds, Other<br>Corporate Stocks<br>Mortgages Held Directly<br>Total Other Securities<br>- State and Local Governments<br>- Foreign and International<br>- Investment Held in Trust<br>- Other Securities<br>Total Other Investments<br>- Real Property<br>- Other Investments

The “By Type” categories shown above are defined in the Description Pages later in this chapter, as well as in Chapter 8.

7.7 Description of Cash and Security Holdings Categories

The following pages contain the detailed Description Pages explaining the statistical classification categories used for cash and security holdings of a government. The codes apply only to state and local governments. No comparable data are collected for the Federal Government.

CASH AND SECURITY HOLDINGS CATEGORIES

PURPOSE CATEGORY: OTHER THAN SOCIAL INSURANCE TRUST SYSTEMS

Code W01 **Offsets to Debt (Debt Service or Sinking Funds)**

Definition: Cash and security holdings held specifically for debt service purposes (interest payments and redemption of principal) on long-term debt, including those of utilities, regardless of debt purpose.

Includes: Cash and security assets as delineated in Table 7.1 and industrial leases recorded as offsets to industrial development debt for the private sector. Includes cash on hand with fiscal agents for debt service or for redemption of uncanceled debt; bond reserve funds; balances in refunding bond accounts held pending completion of refunding transactions (other than for advance refunding); and credit paper or other assets of credit funds pledged to the ultimate redemption of debt incurred to finance loan activities of such funds. Covers assets held for debt redemption up to the amount of the specific debt for which they were accumulated plus interest.

Also includes any mortgages and land contracts, notes receivables held as offsets to public debt for financing private sector activities (i.e., an amount equal to debt outstanding classified at *Public Debt for Private Purposes*, code 44T), and the value of loans made by credit funds from debt proceeds.

Excludes: Balances in advance refunding accounts held for future payment of debt that has been defeased and removed from the official accounts of the government (exclude entirely from survey). Excludes value set on land acquired by foreclosure, other real property, taxes or accounts receivable, interfund loans, and other noninvestment assets.

Special Considerations:

1. An amount recorded as offsets to public debt for private purposes is reduced as the debt creating it is retired. Prior to fiscal year 1988 data, the treatment of these offsets was limited to mortgage revenue bonds. Effective with fiscal year 1988 data, it was expanded to cover all types of public debt for private purposes.

2. Prior to fiscal year 1988 data, this code was further divided into cash and deposits (code W01), Federal treasury securities (code W10), Federal agency securities (code W13), state and local government securities (code W15), and nongovernmental securities (code W24).

3. Prior manuals made a distinction between reserves for the redemption of debt principal (reported under this category) and for the payment of interest (reported under *All Other Funds*, code W61). In recent years, however, data reported here has included an increasing amount for interest since the majority of financial reports from which the data are gathered no longer separate the two components. Note, too, that the GASB definition of a debt service fund makes no distinction between interest and principal.

Applicability By Level and Type of Government

Federal	State	Local
No	Yes	Yes - All Types

CASH AND SECURITY HOLDINGS CATEGORIES

PURPOSE CATEGORY: OTHER THAN SOCIAL INSURANCE TRUST SYSTEMS

Code W31 **Bond Funds**

Definition: Cash and security holdings of accounting funds established specifically to hold proceeds of bond issues (and related revenues) pending their disbursement.

Includes: Various cash and security assets as delineated in Table 7.1.

Excludes: "Capital project funds" whose sources of revenue do not include proceeds of bond issues (report at *All Other Funds*, code W61) and proceeds of public debt for private purposes not yet distributed (report at *Offsets to Debt*, code W01).

Special Consideration:
Prior to fiscal year 1988 data, this code was further divided into cash and deposits (code W31), Federal treasury securities (code W40), Federal agency securities (code W43), state and local government securities (code W45), and nongovernmental securities (code W54).

Applicability By Level and Type of Government

Federal	State	Local
No	Yes	Yes - All Types

CASH AND SECURITY HOLDINGS CATEGORIES

PURPOSE CATEGORY: OTHER THAN SOCIAL INSURANCE TRUST SYSTEMS

Code W61 **All Other Funds**

Definition: Cash and security holdings of funds and other accounts except those cited above, including utilities and liquor stores.

Includes: Various cash and security assets as delineated in Table 7.1.

Excludes: Cash and security holdings of social insurance trust systems (report at appropriate public employee retirement system, unemployment trust, workers compensation trust, or other sub-category as delineated below, and in Chapters 8 and 9).

Special Consideration:
Prior to fiscal year 1988 data, this code was further divided into cash and deposits (code W61), Federal treasury securities (code W70), Federal agency securities (code W73), state and local government securities (code W75), and nongovernmental securities (code W84).

Applicability By Level and Type of Government

Federal	State	Local
No	Yes	Yes - All Types

CASH AND SECURITY HOLDINGS CATEGORIES

PURPOSE CATEGORY: SOCIAL INSURANCE TRUST SYSTEMS

Public Employee Retirement Systems

As noted, the Census Bureau uses additional "By Type" classification categories for cash and securities of public employee retirement systems. The applicable "By Type" codes are listed here, along with their descriptions and any special considerations. In addition to the categories below, there are also special exhibit codes for employee retirement cash and security holding categories. They are referenced here, but described more completely in Chapter 8.

Code X21 Total Cash and Short-Term Investments **Public Employee Retirement Systems**

Definition: Cash on hand and on deposit; demand deposits; time or savings deposits; certificates of deposits; repurchase agreements ("repos"); reverse repurchase agreements ("reverse repos"); commercial and finance company paper; bankers acceptances; and money market funds.

Special Consideration:
This is a calculated statistic. It is the sum of three exhibit codes (see Chapter 8):
+ Z68 All Other Short-Term Investments
+ Z87 Time or Savings Deposits
+ Z88 Cash on Hand and Demand Deposits

Code X30 Total Federal Government Securities **Public Employee Retirement Systems**

Definition: Obligations of the U.S. Treasury (including short-term notes) and the Federal Financing Bank (FFB); and bonds and mortgage-backed securities issued by Federal agencies, such as the Commodity Credit Corporation (CCC), Export-Import Bank, Federal Housing Administration (FHA), Government National Mortgage Association (GNMA, or "Ginnie Mae"), Saving Association Insurance Fund (SAIF), Small Business Administration (SBA), U.S. Postal Service, and Tennessee Valley Authority (TVA).

Excludes: Mortgages directly held by employee retirement system (use *Mortgages Held Directly*, code X42) and securities of Federally-Sponsored or chartered private and quasi-private credit organizations, such as the Federal National Mortgage Association, FNMA or "Fannie Mae" (use *Corporate Bonds, Federally-Sponsored Agencies*, code Z62).

Special Considerations:

1. This is a calculated statistic, representing the sum of two exhibit codes (see Chapter 8).
   + X33 Federal Agency Securities
   + Z89 Federal Treasury Securities

2. Prior to fiscal year 1988 data, these securities were differentiated between Federal Treasury notes (code X30) and Federal agency securities (code X33). Code X30 is now the total holdings, while Z89 represents the *Federal Treasury Securities*. Also see Chapter 8 for a discussion of special exhibit codes for employee retirement cash and securities.

PURPOSE CATEGORY: SOCIAL INSURANCE TRUST SYSTEMS

Code Z77 Total Corporate Bonds **Public Employee Retirement Systems**

Definition: Debentures; convertible bonds; railroad equipment certificates; and securities of Federally-Sponsored or chartered private and quasi-private organizations like Federal Home Loan Banks (FHLB), Federal Home Loan Mortgage Corporation (FHLMC, or "Freddie Mac"), Federal National Mortgage Association (FNMA, or "Fannie Mae"), Federal farm credit banks, and Student Loan Marketing Association (SLMA, or "Sallie Mae").

Special Considerations:

1. Report such securities at their market value at the end of the fiscal year, as determined by the government. This valuation methodology was effective in survey year 2002. Market value also is referred to as "fair value" in many CAFRs.

2. This is a calculated statistic. It represents the sum of two exhibit codes (both measured at market value). See Chapter 8.
   + Z62 Corporate Bonds, Federally-Sponsored Agencies
   + Z63 Corporate Bonds, Other

3. Prior to 2002, corporate bonds were reported at book value (i.e., their original cost less any discounts), not at market value. The code used for that category was X40.

4. Code Z77 was added to regular finance statistics effective with 2002. Prior to that, it was an exhibit code used occasionally during Census of Government surveys only.

Code Z78 Corporate Stocks **Public Employee Retirement Systems**

Definition: Common and preferred stock, warrants, and shares in investment companies.

Special Considerations:

1. Report corporate stocks at their market value at the end of the fiscal year, as determined by the government. This valuation methodology was effective in survey year 2002. Market value also is referred to as "fair value" in many CAFRs.

2. Prior to 2002, corporate stocks were reported at book value (i.e., their original cost less any discounts), not at market value. The code used for that category was X41.

3. Code Z78 was added to regular finance statistics effective with 2002. Prior to that, it was an exhibit code used occasionally during Census of Government surveys only.

Code X42 Mortgages Held Directly **Public Employee Retirement Systems**

Definition: Mortgages held by (owed to) the public employee retirement system and issued as an investment.

Excludes: Mortgage-backed Federal securities (use *Total Federal Government Securities,* code X30) and real property directly owned by system (use exhibit *Real Property,* code X46).

PURPOSE CATEGORY: SOCIAL INSURANCE TRUST SYSTEMS

Code X44 Total Other Securities **Public Employee Retirement Systems**

Definition: Securities of state and local governments (including those issued by parent government administering the system and purchased for investment purposes); investments held in trust by other agencies, including funds administered by private agencies, guaranteed investment accounts, and governmental pooled investment accounts (if allocation among previous categories is not possible); shares held in mutual funds; foreign and international securities; conditional sales contracts; pooled life insurance investments; direct loans; and loans to system members.

Special Considerations:

1. This is a calculated statistic. It is the sum of four exhibit codes (see Chapter 8):
   + X35 State and Local Government Securities
   + Z70 Foreign and International Securities
   + Z83 Other Securities
   + Z84 Investments Held in Trust by Other Agencies
2. Securities of state and local governments were added to this category effective with fiscal year 1988 data. Prior to that time, they were separately identified in regular government finance statistics and coded at X35.
3. Report international securities, included in this category, at their market value at the end of the fiscal year, as determined by the government. This valuation methodology was effective in survey year 2002. Market value also is referred to as "fair value" in many CAFRs.
4. Foreign and International Securities, code Z70, was reported at code Z69 from 1997 to 2001.

Code X47 Other Investments **Public Employee Retirement Systems**

Definition: All other investments of the retirement system, not classified in other categories.

Includes: Venture capital; partnerships; and real estate investment trusts (REITs).

Excludes: Real property held directly by system (use exhibit code X46).

Special Considerations:

1. Prior to fiscal year 1988 data, this category was classified as an exhibit code.
2. For public employee retirement statistics, this category is combined with *Real Property*, exhibit code X46, to create the calculated statistic *Total Other Investments*, exhibit code Z82. See Chapter 8.

CASH AND SECURITY HOLDINGS CATEGORIES

PURPOSE CATEGORY: SOCIAL INSURANCE TRUST SYSTEMS

Applicability By Level and Type of Government
(All regular codes – X21, X30, Z77, Z78, X42, X44, X47)

Federal	State	Local
No	Yes	Yes - All Types *

* These codes apply only where parent governments have dependent agencies that meet Census Bureau definitions of public employee retirement systems. As of the 2002 Census of Governments, such systems had been recognized in all local government types.

The following exhibit codes are also used for classifying assets of Public Employee Retirement Systems. They are not included directly in the regular Census Bureau statistics program, but are used for the annual survey Finances of Public Employee Retirement Systems. Some are used indirectly in the regular government finance statistics, in that they are used to calculate specific variables (codes) noted above. Chapter 8 contains more detailed explanations of these codes.

Exhibit Codes Related to Cash and Securities Holdings for Public Employee Retirement Systems (Summarized – see Chapter 8 for detail)

Code and Description		Used to Calculate Regular Code
Z68	All Other Short-Term Investments	X21
Z87	Time or Savings Deposits	X21
Z88	Cash on Hand and Demand Deposits	X21
X33	Federal Agency Securities	X30
Z89	Federal Treasury Securities	X30
Z62	Corporate Bonds, Federally-Sponsored Agencies	Z77
Z63	Corporate Bonds, Other	Z77
Z84	Investments Held in Trust by Other Agencies	X44
X35	State and Local Government Securities	X44
Z70	Foreign and International Securities	X44
Z83	Other Securities	X44
Z82	Total Other Investments	Not used
X46	Real Property	Not used
Z81	Total Holdings and Investments	Not used

CASH AND SECURITY HOLDINGS CATEGORIES

PURPOSE CATEGORY: SOCIAL INSURANCE TRUST SYSTEMS

Unemployment Compensation Systems

The Census Bureau uses two "By Type" classification categories for cash and securities of the state-administered Unemployment Compensation Systems. Both of the categories shown below are considered to be "cash and deposits." There are no securities, per se, for the holdings of the state Unemployment Compensation System accounts.

Unemployment Compensation Systems are explained more fully in Chapter 9. The Census Bureau defines these systems as dependent activities of the fifty state governments and the District of Columbia. The latter is the only local government to which these codes apply. These categories do not apply to Federal Government statistics.

Data for this category are obtained primarily from the Employment and Training Administration of the U.S. Department of Labor. However, as states have become more inclusive in their reporting as a result of GASB guidelines, Census Bureau analysts can make use of individual state financial statements, where the states include the Unemployment Compensation Systems activity in such accounting records.

Code Y07 Trust Fund Account in U.S. Treasury **Unemployment Compensation Systems**

Code Y08 Other (Clearing and Benefit Accounts) **Unemployment Compensation Systems**

Special Considerations:

1. Y08 can be a positive or a negative number. Negative balances occur during periods of higher than normal unemployment, where the state governments "borrow" from the Federal Government to cover extended or special benefit unemployment benefits.
2. Pre-1988 versions of the *Classification Manual* showed code Y09 for negative balances and limited Y08 to positive balances. Y08 now represents the net balance, plus or minus.

Applicability By Level and Type of Government

Federal	State	Local
No	Yes	No - All Types *

* These codes apply to Washington, DC (classified by the Census Bureau as a municipal government).

CASH AND SECURITY HOLDINGS CATEGORIES

PURPOSE CATEGORY: SOCIAL INSURANCE TRUST SYSTEMS

Workers' Compensation Systems

The Census Bureau uses no "By Type" classification categories for cash and securities of the state-administered Workers' Compensation Systems. The category shown below is not further subdivided.

Statistics included in the category below must be from Workers' Compensation Systems recognized by the Census Bureau as dependent agencies of a parent state government. Such systems do not exist in all states. See Chapter 9 for additional information.

Code Y21 Total Cash and Securities **Workers' Compensation Systems**

Definition: Cash and security holdings of state government compulsory accident and injury insurance systems for workers' compensation.

Includes: Various cash and security assets as delineated in Table 7.1.

Special Considerations:

1. Prior to fiscal year 1988 data, this code was divided further into cash and deposits (represented by code Y21), Federal treasury securities (code Y30), Federal agency securities (code Y33), state and local government securities (code Y35), and nongovernmental securities (code Y44).

2. As a consequence of the 1988 revisions, code Y21 has two meanings in historical Census Bureau historical statistics on government finances.

Applicability By Level and Type of Government

Federal	State	Local
No	Yes	No - All Types

CASH AND SECURITY HOLDINGS CATEGORIES

PURPOSE CATEGORY: SOCIAL INSURANCE TRUST SYSTEMS

Other State Social Insurance Trust Systems

The Census Bureau uses no "By Type" classification categories for cash and securities of the state-administered Other Social Insurance Trust Systems. The category shown below is not further subdivided. Statistics included in the category below must be from Other Social Insurance Trust Systems recognized by the Census Bureau as dependent agencies of a parent state government. Such systems do not exist in all states.

Code Y61 Cash and Securities **Other State Social Insurance Trust Systems**

Definition: Cash and security holdings of state government insurance trust systems other than employee retirement, unemployment compensation, and workers' compensation.

Includes: Various cash and security assets as delineated in Table 7.1.

Examples: Maryland Automobile Insurance Fund, a dependent agency of the state of Maryland.

Special Consideration:

Prior to fiscal year 1988 data, this code was further divided into cash and deposits (code Y61), Federal treasury securities (code Y70), Federal agency securities (code Y73), state and local government securities (code Y75), and nongovernmental securities (code Y84).

Applicability By Level and Type of Government

Federal	State	Local
No	Yes	No - All Types

7.8 Tables

The tables on the following pages contain valuable information on how the Census Bureau classifies statistics on cash and security holdings. Table 7.1 describes assets generally held by government units and shows whether they are included or excluded (in-scope or out-of-scope) from the Census Bureau classification system. Table 7.2 shows the applicability of all cash and security codes (regular and exhibit) by level and type of government. Note also that Appendix Table 1.1 contains important historical information on how cash and security holdings were measured.

Table 7.1
Examples of Assets Included and Excluded from
Census Bureau Definition of Cash and Securities – page 1 of 2

Examples of assets found on a government's balance sheet and their classification for Census Bureau purposes. Note: This list is not exhaustive. It contains the most common types of assets found in annual financial reports for purposes of illustration.

Included in Census Bureau Definition of Cash And Securities	Excluded from Census Bureau Definition of Cash And Securities
Bankers acceptances	Accounts receivables
Cash on deposit	Accrued interest or dividends on investments
Cash on hand	Advances to other funds
Cash with fiscal agent (e.g., for debt service)	Allowances for delinquent taxes
Cash "equivalents"	Amortized or deferred bond issue costs
Certificates of deposit (CDs)	Construction-in-Progress
Commercial paper	Customer deposits
Conditional sales contracts	Deferred charges (or other revenues)
Corporate bonds (including debentures, convertible bonds, and railroad equipment certificates)	Deferred compensation
	Deposits with other governments
	Depreciation
Corporate stock (common and preferred)	Dividends receivables
Current investments	Due from other funds
Debentures held	Fixed assets (land, equipment, buildings, and other structures)
Demand deposits	
Equity in pooled cash or investments accounts	Goodwill
Federal agency securities (FHA, TVA, etc.)	Grants receivables
Federally-Sponsored agency securities	Improvements "other than buildings"
Federal treasury notes	Interest receivables
Finance company papers	Interfund receivables
Foreign and international securities	Inventory
Guaranteed investment accounts	Investment in general fixed assets
Leveraged buyouts	Leases receivable (other than industrial leases as offsets to industrial development debt)
Loans to members of insurance trusts systems	
Loans receivables	

Table 7.1
Examples of Assets Included and Excluded from
Census Bureau Definition of Cash and Securities – page 2 of 2

Examples of assets found on a government's balance sheet and their classification for Census Bureau purposes. Note: This list is not exhaustive. It contains the most common types of assets found in annual financial reports for purposes of illustration.

Included in Census Bureau Definition of Cash And Securities	Excluded from Census Bureau Definition of Cash And Securities
Marketable equity securities	Letters of credit
Marketable securities	Machinery and equipment
Money market funds	Materials and supplies
Mortgages (receivable) held directly	Net depreciation
Mutual funds	Prepaid expenses
Notes receivables	Real property*
Partnerships	Special assessments receivables
Real estate investment trusts (REITS)	Tax liens receivables
Repurchase agreements ('repos')	Taxes receivables
"Restricted" cash and investments	Unamortized premiums on investments
Savings accounts	Unbilled accounts receivables
Share of funds in governmental investment accounts or pools	Unrealized gains on investments **
State and local government securities (including own securities held by its insurance trusts)	
Time deposits	
Venture capital	
Warrants (to purchase securities)	
Unrealized gains on investments **	

**For employee retirement statistics real property is reported at a special exhibit code. See Chapter 8.*

***See Section 7.2.2*

**Table 7.2<br>Applicability of Cash and Security Codes, by Level of Government**					
**Category**	**Code**	**Code Type**	**Federal**	**State**	**Local - All Types**
**Other Than Social Insurance Trust Systems:**					
Sinking Funds	W01	R	X	Valid	Valid
Bond Funds	W31	R	X	Valid	Valid
Other Funds	W61	R	X	Valid	Valid
**Social Insurance Trust Systems – Public Employee Retirement Systems:**					
Cash and Short-Term Investments	X21	R	X	Valid	Valid
Cash and Demand Deposits	Z88	E	X	Valid	Valid
Time or Savings Deposits	Z87	E	X	Valid	Valid
All Other Short-Term Investments	Z68	E	X	Valid	Valid
Total Federal Government Securities	X30	R	X	Valid	Valid
Federal Treasury Securities	Z89	E	X	Valid	Valid
Federal Agency Securities	X33	E	X	Valid	Valid
Total Corporate Bonds	Z77	R	X	Valid	Valid
Federally-sponsored Agencies	Z62	E	X	Valid	Valid
Corporate Bonds, Other	Z63	E	X	Valid	Valid
Corporate Stocks	Z78	R	X	Valid	Valid
Mortgages Held Directly	X42	R	X	Valid	Valid
Total Other Securities	X44	R	X	Valid	Valid
Investments Held in Trust	Z84	E	X	Valid	Valid
State and Local Government Securities	X35	E	X	Valid	Valid
Foreign and International Securities	Z70	E	X	Valid	Valid
Other Securities	Z83	E	X	Valid	Valid
Total Other Investments	Z82	E	X	Valid	Valid
Real Property	X46	E	X	Valid	Valid
Other Investments	X47	R	X	Valid	Valid
Total Holdings and Investments	Z81	E	X	Valid	Valid
**Social Insurance Trust Systems – Unemployment Compensation System:**					
Trust Fund Account - US Treasury	Y07[1]	R	X	Valid	X[1]
Clearing and Benefit Account Balance	Y08[1]	R	X	Valid	X[1]
**Social Insurance Trust Systems – Workers' Compensation Systems:**					
Total Cash and Security Holdings	Y21	R	X	Valid	X
**Social Insurance Trust Systems – Other State Social Insurance Trust Systems:**					
Total Cash and Security Holdings	Y61	R	X	Valid	X

Key:

X — Not a valid code for the level or type of government indicated (there are no statistics).

Valid — Applicable code for the level or type of government indicated (statistics are collected and reported).

Code Type

R = Regular Statistic
E = Exhibit Statistic
D = Descriptive Statistic

Notes:

[1]Applies to Washington, DC, only, for local governments.

Chapter 8. Public Employee Retirement Systems

Introduction

As described in Chapter 2, Social Insurance Trust Systems constitute one of the four sectors of government in Census Bureau statistics on government finance. The Census Bureau recognizes four types of social insurance trust systems:

- Public employee retirement systems
- Unemployment compensation systems
- Workers' compensation systems
- Other Federal and state social insurance trust systems

This chapter contains information on the classification of financial statistics of the first type – public employee retirement systems. The focus of this chapter is on public employee retirement systems as entities, separate and apart from the parent government. Explanations of how statistics on public employee retirement systems fit into the Census Bureau's regular statistics on government finance can be found in Chapter 4 (revenue), Chapter 5 (expenditure), and Chapter 7 (cash and security holdings), as well as Section 8.4. Table 8.2 at the end of this chapter shows how specific codes applicable to these systems relate to the regular statistics.

The Census Bureau develops three types of statistics on public employee retirement systems. First, they are reported with the regular finance data about governments (*regular statistics*). In this regard they are included with such government finance measures as total revenue and total cash and security holdings. Second, since public employee retirement systems involve major financial activities that fall outside the scope of the regular classification system for government finances, the Census Bureau develops additional *exhibit statistics.* Exhibit statistics complement the statistical coverage of this particular sub-sector of government finance activity. A third set of statistics for public employee retirement systems can be labeled as descriptive (*descriptive statistics*). These contain information on the characteristics of each public employee retirement system and are used by the Census Bureau in its effort to explain the structure and organization of government units. Section 8.2 contains additional information.

This chapter on public employee retirement system statistics has no applicability to the Census Bureau's statistical program on public employment. This follows from the Census Bureau's definition of government sectors. For purposes of *regular finance statistics*, all administrative costs associated with public employee retirement systems are classified in the general government sector, by definition. Consequently, all employees engaged in the activity of administering public employee retirement systems also are classified in the general government sector.

8.1 Public Employee Retirement Systems Definition

Public employee retirement systems are a sub-sector of the more general sector of social insurance trust systems. For the latter, many governments administer a variety of insurance-type programs in addition to maintaining various "trust" funds. For Census Bureau statistics, however, only a selected few of these qualify as "social insurance trust systems." To be categorized as a social insurance trust for Census

Bureau purposes, a government's system must meet all of the following criteria:[1]

1. The system must be financed by a separate accounting fund of the administering government. This criterion excludes many pay-as-you-go insurance plans.

2. This fund must have some type of assured revenue stream or dedicated revenue source other than appropriations from the administering government (generally, contributions or premiums imposed on its members and/or member employers).

3. The system must be a *social* insurance plan – that is, it must have a social purpose such as benefiting the disabled or disadvantaged, aiding individuals who cannot afford private insurance, providing future income, and the like.

4. The members of the system must be outside the government itself – that is, the plan cannot be for insuring the government itself against risks. For this criterion, public employees are considered to be outside the government.

5. The plan must be administered directly by the government itself – that is, it cannot just collect contributions and then turn them over to a private insurer who assumes the actual risks and administers the plan.

Within the concept of a social insurance trust system, as outlined above, public employee retirement systems must meet several additional criteria:

- They must be sponsored by a recognized unit of government as defined by the Census Bureau.

- Their membership must be comprised of current or former public employees. These must be the same as those employees eligible for inclusion in the employment phase of the Census of Governments.

- The system must consist of a separately identifiable fund (or funds) within a recognized unit of government and must be financed in whole or in part with public contributions. The assets used to compensate its members, when eligible for benefits, must be identifiable and fully dedicated to finance retirement and associated benefits.

Public employee retirement systems include both contributory and noncontributory systems, administered by a government for public employees (including employees of other governments). Contributory systems are those wherein employees (in addition to employers) make contributions for future benefits. Noncontributory systems are those wherein only employers contribute – employees are not required to do so.

8.1.1 Examples of Public Employee Retirement Systems Included by this Definition

Defined benefit plans are the primary type of retirement system plan included in the Census Bureau definition. Traditionally, these involve a promise by the system to pay benefits to members or their

[1] Note that of the four types of social insurance trust systems recognized by the Census Bureau, only the public employee retirement systems category applies to local government finances (plus the District of Columbia's unemployment compensation system). All other types apply to Federal and/or state governments only.

survivors. The benefit payments are linked to some combination of years of service, salary, and age. Defined benefit plans are funded by employer contributions, and might also include employee contributions (voluntary or otherwise).

Traditionally, most state governments administer defined benefit plans for their employees or for selected types of local government employees. Teachers, police, and fire employees are the most common types of retirement systems dedicated to specific employee types. Descriptive information about the types of retirement systems that exist can be found in the Census of Governments volume 4, number 6, *Employee Retirement Systems of State and Local Governments.*

Local governments administer a wide variety of defined benefit plans, although many participate in state administered systems designed to cover local employees. Again, systems for teachers or school employees are the most common.

For its statistics on Federal Government finances, the Census Bureau currently recognizes three Federally-administered systems. See Section 8.5 for explanations.

8.1.2 Examples of Public Employee Retirement Systems Excluded by this Definition

Several general methods of providing retirement benefits for public employees are excluded from the Census Bureau definition, even though they are used by some government entities.

- Defined contribution plans. These plans are established by a government but administered by a private entity or trust. Even where the government contributes to such plans, they are out-of-scope for Census Bureau purposes. The contributions made by the government (generally representing a “matching” share of employee contributions) are classified as a general expenditure of the government. Such expenditures are classified at the appropriate function, based on employee activity (see Chapter 4).

- Plans that are supported entirely by employee contributions only. These are most often defined contribution plans, but there can be variations. By definition, financial transactions of funds handling employee money only are excluded from all financial reporting on governments in any of the Census Bureau series dealing with government finances. For example, the Teachers’ Insurance Annuity Association (TIAA) provides public employee retirement coverage, but without any contribution or supplemental coverage administered by a government.

- Deferred compensation option plans, such as those developed under section 457 of the Internal Revenue Code. These 457 plans, available to many governmental workers, are similar to the standard 401(k) plans, but with no penalty for pre-retirement withdrawals.

- “Pay-as-you-go” retirement programs (i.e., direct payments to retired or disabled employees from appropriation of general funds) and similar cash balance plans.

- Payments to private trustee or insurance carrier that administers the investments and benefit payments.

- Other post-employment benefit plans (OPEB plans) that offer specific benefits such as health care for former employees. These are specifically excluded from the public

employee retirement sub-sector, but might qualify as “other social insurance trust systems” under the Census Bureau’s classification system. See Chapter 9.

Under these exclusions, any direct payments to individuals and private corporations are recorded in the finances of the general government as direct expenditures or current operations.

Another type of public employee retirement system is often referred to as a “Hybrid Plan.” Such plans represent an area of difficulty for determining whether a system is in-scope or out-of-scope for Census Bureau purposes. Hybrid plans generally are a mix of traditional defined benefit plans and other types of compensation. In this regard, they provide certain benefit guarantees to the member, but with financial conditions attached. Among the latter are member requirements for minimum own-contributions, or that members must take a lump-sum payout at retirement and forfeit rights to any long-term annuity. Two examples of hybrid plans are cash balance plans and equity plans.

The difficulty in classifying hybrid plans lies in the fact that the plans can be fully-administered by the sponsoring government unit, much like a regular defined benefit plans. As a result, the plan might meet all of the criteria noted above for classifying a public employee retirement system. The Census Bureau would include a hybrid plan in its coverage for this survey, if those criteria were met. This determination is made on a case-by-case basis.

Although it is a retirement system, the Federal Social Security program is classified as an “other social insurance trust system.” This is because its membership is not limited to public employees. Similarly, the Railroad Retirement system that provides retirement-survivor benefits to former public and private railroad workers and their families is not classified as a public employee retirement system.

8.2 Regular Finance Statistics, Exhibit Finance Statistics, and Descriptive Statistics – Explanations

Regular Census Bureau finance statistics provide only selected (partial) information on insurance trusts. The critical perspective in the regular statistics program is to present the financial activity for the government as a whole. Regular finance statistics covering government financial activity for public employee retirement systems include the revenue statistics described in Chapter 4, the expenditure statistics described in Chapter 5, and the cash and security holdings statistics described in Chapter 7. There are no debt statistics associated with public employee retirement systems directly, since they are not authorized to issue debt or otherwise borrow money. Any such borrowing is always the responsibility of the parent government administering the system. See Section 8.4.6, for additional information on this debt topic.

Exhibit statistics are used to enhance the regular statistics in two ways. First, they add additional detail to the regular statistics, showing further breakdowns of financial activity not required in the development of regular finance statistics. Secondly, the exhibit statistics are designed to account for financial transactions that occur between the insurance trust system and its parent government. By definition, such transactions are excluded from regular statistics.

Descriptive statistics represent information on the characteristics of each public employee retirement system. They are used by the Census Bureau to explain the structure and organization of government units, primarily for classification purposes related to the Census of Governments. These statistics do not cover fiscal year financial activity, per se, although some of them provide “point in time” measurements of selected financial transactions. Consequently, they are not used on overall financial measurements for regular statistics, nor are they used in exhibit statistics related to the enhanced measurement of public

employee retirement systems as stand alone entities. These descriptive statistics also are developed for specific user needs to describe or characterize the important role of public employee retirement systems. Descriptive statistics include data on membership, the type of system, and numbers of annuitants. They generally are confined to survey years where a complete Census of Governments is conducted. Descriptive statistics are detailed later in this chapter.

8.2.1 Exhibit Statistics – Additional Detail

In the case of public employee retirement systems, the additional detail added to the regular statistics consists primarily of statistics on cash and security holdings. At the state and local government level, such holdings of public employee retirement systems totaled $2.2 trillion in the last Census of Governments (2002) and represented sixty percent of total government cash and security holdings.

The Census Bureau does not collect this type of additional detail for all sectors of the government. There are a number of reasons for this, including the fact that other government sectors and sub-sectors do not have the extensive array of cash and security holdings found in public employee retirement systems. Rather than attempt to develop such detail, the Census Bureau uses these additional exhibit statistics for sectors where such holdings are significant to users.

The additional detail provided by exhibit statistics meets the needs of certain types of special users of the Census Bureau program. For public employee retirement system statistics, the Federal Reserve Board uses these additional exhibit statistics in its statistical programs (such as the Flow of Funds Accounts).

8.2.2 Exhibit Statistics – Enterprise Perspective

As described in Chapter 2, the basis of reporting for Census Bureau statistics is the government entity. The definitions are such that many financial activities of dependent agencies, or financial transactions between the parent government and its dependent public employee retirement system, are excluded from basic measures. Exhibit statistics enable the Census Bureau to treat selected government activities independently of the parent government and give users an important perspective on financial activities of government-related enterprises. Such is the case with public employee retirement systems.

For public employee retirement system finances, the principle differences between the regular statistics are detailed in Section 8.4.

8.3 Measurement Issues for Public Employee Retirement System Statistics

The following are explanations of how financial transactions of public employee retirement systems are measured for statistical purposes.

8.3.1 Measurement Issues: Timing

As with other Census Bureau statistics on government finances, the fiscal year is the basis of measurement for public employee retirement statistics. The same rules described in Section 3.2 for governments apply to public employee retirement systems. Most, but not all, public employee retirement systems have the same fiscal year as their parent government. Where their fiscal year differs from that of the parent government, the Census Bureau uses the 12-month fiscal year of the retirement system as the basis for measuring finances. For this reason, Census Bureau statistics on

public employee retirement system finances might differ from financial statistics that appear in the parent government records, such as the CAFR. The following considerations are applicable to measurement during the fiscal year:

- Receipts and expenses are measured over the course of the fiscal year and represent total amounts received or expended for that year.

- Cash and security holdings are measured as of the end of the fiscal year. For assets measured using market value, the end-of-year market value is used.

- Most descriptive statistics, such as membership, are measured as of the end of the fiscal year. However, monthly categories are measured for the last month of the fiscal year. These are clearly labeled in Census Bureau reports and data files.

8.3.2 Measurement Issues: Aggregation and Tabulation

The Census Bureau uses three different methods for aggregating and tabulating statistics involving public employee retirement systems. First, only regular statistics are incorporated into the aggregate statistics used to measure the finances of the parent government. Hence selected “receipts” statistics are aggregated into the parent government’s revenue data, and selected “expenses” statistics are aggregated into the parent government’s expenditure statistics. In turn, such statistics are tabulated in totals by type of government, by state, by level of government, and so forth. For example, national totals of employee retirement revenue, as used in a Census Bureau publication on government finances, include only the regular statistics identified in this chapter.

Secondly, exhibit statistics for individual public employee retirement systems complement the regular statistics. Regular and exhibit statistics are combined for individual public employee retirement systems. The aggregations present a complete financial picture of the individual employee retirement systems, without regard for the parent government. This is an important concept, since many parent governments administer multiple employee retirement systems. Statistics on receipts and expenses can be tabulated for multiple retirement systems only, but not for parent governments.

Third, descriptive statistics stand alone and are not combined with regular or exhibit statistics for any tabulation purposes. Descriptive statistics can be aggregated for multiple retirement systems, when tabulations are made by type of system or by geographic area, such as by state.

8.3.3 Measurement Issues: Valuation

As discussed in Section 3.12, the Census Bureau program is first and foremost statistical in nature and is not intended to reflect the same types of measures developed for purely accounting purposes. Nevertheless, the Census Bureau uses standard accounting records to develop its government finance statistics, so accounting definitions and concepts must be taken into account in the Census Bureau statistical program.

For statistics on public employee retirement systems, all receipts and expenses are measured only in current dollars. Each represents the total of financial transactions that occurred during the fiscal year.

As described previously in Section 7.2.2, the Census Bureau uses market value to measure such holdings. This valuation method became effective with the 2002 Census of Governments. Prior to 2002, statistics for cash and security holdings were collected using a mix of valuation methods.

These are detailed in Appendix 1 and in Appendix Table 1.1.

8.4 Public Employee Retirement System Finances – Basic Concepts

Regular Census Bureau finance statistics provide only selected data on insurance trusts, excluding such activities as contributions from the administering government, the cost of administering systems (reported in the general government sector), and certain investment assets that are neither cash nor securities.

The principal differences in insurance trust finances between the regular statistics and the exhibit data can be summarized as follows:

1. Regular finance insurance trust revenue excludes (as an intragovernmental transfer) contributions and other payments from the administering government, including payments made on behalf of its own employees who are members of the system. Insurance trust statistics on receipts include these sources of funds.

2. Intergovernmental transfers, taxes levied specifically to support insurance trusts, or other sources of general revenue to finance insurance trusts activities are classified as general revenue, not as insurance trust revenue. Note that intergovernmental transfers representing contributions from other governments on behalf of their employees who are members of the system are treated as insurance trust revenue.

3. The cost of administering an insurance trust system is classified as a general expenditure of the administering government. It is recorded also under a special exhibit code for displaying insurance trust statistics.

4. For public employee retirement systems, revenue includes unrealized gains on the value of cash and security investments held at the end of the fiscal year (or the value of unrealized losses, which are deducted from revenue).

The following Sections explain the concepts pertinent to receipts, expenses, and cash and security holdings, as well as special treatment of selected financial activities of public employee retirement systems.

8.4.1 Public Employee Retirement Systems Revenue and Receipts

As described in Chapter 4, insurance trust revenue comprises only retirement and social insurance contributions, and earnings on investments. Earnings on investments, in turn consist of both realized and unrealized gains or losses. See Section 8.4.1.1 following.

For purposes of regular statistics on government finances, transfers or contributions from other funds of the same government are not classified as insurance trust revenue. Thus, public employee retirement system revenue consists of contributions distinctively imposed for the support of public employee retirement and earnings on their investment assets. Insurance trust revenue excludes the following from receipts:

- Contributions from the government which administers the system (as interfund transfers), whether they are paid on behalf of its employees covered by the plan or for supplemental

support.[2]

- Tax receipts credited directly to insurance trust funds. These are classified as general revenue.

- Intergovernmental aid, such as grants and shared taxes for support of insurance trust activities.[3] These also are classified as general revenue.

- Proceeds from borrowing for insurance trust purposes. These are excluded entirely as revenue (insurance trust or general – see Chapter 3).

However, Census Bureau statistics on public employee retirement system receipts are broader in concept and include both the regular and exhibit statistics explained earlier. For public employee retirement systems, "receipts" include the following categories:

- Amounts derived from contributions required of employers and employees (whether mandatory or voluntary).

- Net earnings on investments set aside to provide income for insurance trusts.[4] Note that losses on investments are deducted from receipts, rather than classified as expenses.

- Transfers or contributions from the parent government that administers the system (either as employer contributions or for general financial support).

- Rentals from real property leased to other agencies of the parent government.

- Interest earnings on their own or other government's securities held for investment are included because of the difficulty in identifying such transactions.

- Grants and shared taxes for support of employee retirement systems, whether received directly or through the parent government.

An important exception to the above involves payment by one government to an insurance trust system administered by another, most commonly public employee retirement systems.

The payment by one government, either on behalf of its employees who are members of the plan or for general financial support, to another governments insurance trust is treated as a current operation

---

[2]Such contributions by the administering government, however, are recorded under special exhibit codes and included in insurance trust revenue when data are published solely for insurance trust systems.

[3]On the other hand, funds from other governments which represent the latter's employer share of contributions to an insurance trust system to which their employees are members are classified as insurance trust revenue.

[4]The substantial amount of interest paid by the U.S. Treasury to Federal insurance trust systems (which have all their reserves invested in Federal securities) is excluded from Federal insurance trust revenue since it is an intragovernmental transfer. The principle of eliminating these interfund transactions, however, is not followed in the case of interest paid by a state or local government on any of its own securities held as investments by insurance trust funds it administers – mainly because of the difficulty of identifying such transactions.

expenditure of the paying government (for the function involved) and as an insurance trust revenue of the receiving government, not as intergovernmental transactions. Thus, one side of the transaction is current operations and the other side is an insurance trust revenue.

The purpose of this treatment is to avoid an imbalance between intergovernmental revenue and expenditure. Since intergovernmental revenue and expenditure are "two sides of the same coin," in theory (or a perfect data collection system) the two should always equal. Also, contributions for an insurance trust system are insurance trust revenue so long as they come from outside the administering government. To avoid the imbalance between intergovernmental revenue and expenditure that would result if the payment of the contribution were treated as an intergovernmental expenditure and the receipt were treated as an insurance trust revenue, neither of these transactions is treated as intergovernmental.

8.4.1.1 Unrealized Gains or Losses as Revenue

Unrealized gains (or losses) on cash and security holdings are included as revenue in Census Bureau statistics for public employee retirement systems. This treatment was effective for the 2002 Census of Governments, and represented a major change in statistical methodology for the government finance statistical program. Explained more fully in Appendix 1, the primary reasons for including unrealized gains were twofold: the widespread acceptance of public accounting industry standards as promulgated by GASB, and the Census Bureau's reliance on respondent accounting records, such as CAFRs, as data sources.

The basic methodology for measuring unrealized gains or losses involves comparing the market value of cash and security holdings at the end of the current fiscal year to the value of the same holdings at the end of the prior fiscal year. The difference in total value for these holdings represents an unrealized gain or loss on investments.

The pertinent finance codes for reporting unrealized gains and losses are *Total Earnings on Investments*, regular code X08, *Gains on Investments, Realized and Unrealized,* exhibit codes Z96, and *Losses on Investments, Realized and Unrealized*, exhibit code Z91. Unrealized gains are included in the X08 statistic along with other types of realized gains, according to the formula noted on the Description Pages later in this chapter. Unrealized losses are classified as a revenue item, not an expenditure, in statistics for public employee retirement systems. If unrealized losses are present, they are netted from *Total Earnings on Investments*. In some rare cases, large amounts of unrealized losses can result in *Total Earnings on Investments* being a negative number. When that occurs, the amount is carried as a negative in the Census Bureau statistics.

8.4.2 Public Employee Retirement Systems Expenditure and Expenses

As described in Chapter 4, statistics on insurance trust expenditure are limited to the perspective of the parent government and its dependent agencies in total. As such, for public employee retirement systems, expenditure consists of social insurance payments to beneficiaries, public employee retirement annuities and other benefits, and withdrawals of employee retirement contributions. In effect, the concept of expenditure includes only amounts paid to beneficiaries or members of the systems. Under this concept, the following are excluded from public employee retirement system expenditures:

- Costs of administering the system, including management of investments (classified as a general government expenditure).

- Contributions to a government's own system (an intragovernmental transfer).

- Contributions or other payments to systems administered by another government (classified as current operation expenditure when such amounts represent employer contributions on behalf of the contributing governments employees and as an agency transaction when such amounts represent sums withheld from employee salaries and wages).

- "Pay-as-you-go" plans or "gratuities" to former employees – i.e., payments not involving a plan of employee assessments or accumulation of retirement reserves (classified as current operation expenditure).

- Group insurance premiums covering the government's own employees and contributions to private insurers or to public employee association retirement systems (classified as current operation expenditure).

Census Bureau statistics on public employee retirement system expenses are broader in concept and include both the regular and exhibit statistics explained earlier. Public employee retirement system expenses are of three types.

1. Benefits: Cash payments to, or on behalf of, participants for retirement benefits and annuities, death and disability benefits, life and disability insurance on behalf of retirees, pre-retirement death benefit premiums, benefits due on termination of employment, survivors benefits, and other benefits as allowed.

2. Withdrawals: Cash benefits to employees, former employees, or their survivors as return of contributions made by employees and any interest on such amounts.

3. Other Payments: Expenses include the exhibit codes for administering retirement system or managing its assets (also reported at *Financial Administration*, code *23 for regular statistics on government finances) and other costs or payments not representing benefits or withdrawals (except purchase of investments). Note that losses on investments are treated as deductions from receipts.

Public employee retirement system expenses exclude the following, by design and definition:

- Payments to own retirement system (interfund transfer).

- Payments to retirement plans administered by another government, such as Federal Social Security and local payments to state-administered retirement systems.

- Payments of premiums for annuity policies from private insurance carriers.

- Contributions to pension plans administered by private companies or by public employee associations.

- "Pay-as-you-go" pensions and other direct payments to retired employees from current appropriations (report preceding items as current operations expenditure of function

involved).

- For the Federal Government, pensions paid out of Federal Military Retirement Fund (use *Federal National Defense and International Relations,* code *06).

- Purchase of investments (see Section 8.4.4).

- Loans to members (see Section 8.4.6.1).

- Amortization of premiums on purchase of investments (nonexpenditures by definition).

State government aid to local retirement systems, which does not represent employer contributions, is reported as a general expenditure at *Other and Unallocable*, code E89, in regular statistics on government finance. Since the local retirement systems treat this aid as an insurance trust revenue, recording the state payment as an intergovernmental expenditure would distort the intergovernmental flows within the state.

8.4.3 Public Employee Retirement System Cash and Security Holdings

Unlike statistics on receipts and expenses, there are very few differences between regular statistics and exhibit statistics on cash and security holdings of public employee retirement systems. The concepts and definitions in Chapter 7 apply almost equally to both sets of statistics, as do the definitions for each type of asset included in the Census Bureau program. The only difference is that exhibit statistics covering public employee retirement systems include one category of asset that is out-of-scope for purposes of the regular financial statistics program. Specifically, the value of real property held by the retirement system is classified as an asset for public employee retirement purposes, but excluded by definition from regular government finance cash and security holding statistics.

One special note on cash and security holdings involves the retirement systems of the Federal Government. The Census Bureau does not include cash and security holdings of the Federal systems. These are invested entirely in special “own government” Federal securities and are therefore not counted as assets.

8.4.4 Special Topics: Purchase and Sale of Investments

Insurance trust exhibit statistics exclude the purchase and sale of investments in statistics for “receipts” and “expenses.” Similarly, insurance trust statistics included in the regular Census Bureau finance statistics exclude these transactions from “revenue” and “expenditure” aggregates.

When a retirement system purchases an investment, the Census Bureau does not record an expense. This is because the financial transaction represents a transfer, essentially, from one type of financial activity to another. The expense becomes an asset, and the latter still is measured (included) in the retirement system’s data. Recording the purchase as an expense would double count the amount of the transaction, from the perspective of the individual retirement system. When an investment is sold, the transaction is not recorded as a receipt because it would overstate income, since there was no expense recorded.

Instead, the Census Bureau methodology for reporting the purchase and sale of investments is to include recorded (actual) gains and/or losses on the investment at the time of final sale. This is

consistent with reporting only final expenditures (not intragovernmental flows) in the insurance trust expense statistics (or in expenditures for regular statistics). The retirement system is asked to report the net amount of gains or losses, in the aggregate, for all investments actually sold during the fiscal year. This amount is one component of the calculated statistic *Earnings on Investments*, code X08. See the previous sub-Section 8.4.1 for additional information about how realized and unrealized gains are measured.

8.4.5 Special Topics: "Internal" Investment Transactions

Insurance trust systems often hold securities issued by their own government. Since these types of transactions are generally not identified in a government's records, the interest paid by the government for such securities is reported as interest expenditure (e.g., at *Interest on General Debt*, I89) and the interest received by the insurance trust system is reported as earnings on investments. (The exception is the interest earnings of Federal Government insurance trusts since all their investments are in U.S. securities.)

8.4.6 Special Topics: Loans and Borrowing Associated with Employee Retirement System Finances

Public employee retirement systems engage in some special lending transactions, as permitted by their authorizing legislation or administrative governing authority. Many of them are permitted to make loans (advances) to members. A small number make loans or advances to their parent government. The sections below describe how such transactions are reported.

8.4.6.1 Special Topics: Loans to Members

Insurance trust exhibit statistics exclude the extension and recoupment of loans to members. Loans (or advances) to members are a common practice of public employee retirement systems. Such loans are classified as investments only. In other words, loans are neither classified as expenses of the system, nor are amounts paid back classified as receipts. Instead, when a member borrows on future benefits, the amount is re-classified in terms of the type of cash and security holding. If the borrower had designated funds invested in Federal Government notes, then the amount of that investment is reduced by the amount borrowed (advanced), while the category "other securities" is increased by the amount borrowed. In this case, the other securities category is defined to include loans to members.

Note that interest on the amount borrowed, as repaid by the member, is counted as a receipt of the retirement system, since it represents newly received monies not previously counted in the Census Bureau statistics.

8.4.6.2 Special Topics: Loans to Parent Government

Because of their large amounts of cash and security holdings, insurance trust systems (especially public employee retirement systems) sometimes help out their parent government during times of fiscal distress. How these financial transactions are treated depends on the situation involved.

The insurance trust system may help out its parent government by purchasing its regular debt instruments. These are treated the same as other debt and investment transactions, as follows:

- It is not reported as either revenue of the parent government or as expenditure of the insurance trust.

- It is reported under debt statistics of the parent government (debt issued and outstanding) and under cash and security holdings data of the insurance trust.

- Interest payments on the debt are reported as an interest expenditure of the parent government (generally, *General Interest on Debt*, code I89) and as earnings on investment of the insurance trust. No effort is made to exclude this transaction as an interfund transfer.

The insurance trust system also might lend money directly to the parent government. In these situations, the loan is not treated as debt of the parent government nor as a cash and security holding of the parent government. It is not reported as an insurance trust expenditure. The purpose of this treatment is to avoid duplication.

8.4.7 Special Topics: Fiscal Conditions of Retirement Systems

This topic constitutes one of the major differences between Census Bureau statistics on public employee retirement systems and the actual accounting records and reports of the systems themselves. The Census Bureau does not include these liabilities in its statistics, even though all systems are required to report them. The Census Bureau's classification system is not designed to measure the future liabilities of a system, such as how much a system owes in future benefit payments to members or their survivors. Census Bureau statistics are limited to payments made during the fiscal year only. As a direct result of this omission, the accounting concept of "unfunded liabilities" also is excluded from Census Bureau statistics.

The omission of this category is intentional and was largely the result of such information being unavailable from most systems over the years. Other considerations in this omission were the issue of how to measure such amounts (all Census Bureau statistics are in current dollars only), whether such "estimated" amounts should be reported in a classification system designed to reflect actual cash flows (transactions) only, and the general concern about avoiding any appearance of oversight or making judgments about the fiscal conditions of individual retirement systems. The Census Bureau is a statistical agency only, not involved in financial oversight. The latter is essentially a state government function, and a function of select Federal agencies involved in financial monitoring.

The statistics generated from the Census Bureau's survey about the finances of public employee retirement systems, therefore, should not be equated to information found in accounting statements. Users should not use the data to infer the financial condition of specific government retirement systems or of their parent governments. Section 3.12 covered this same topic for government finances in general, but the caution applies even more strongly to the statistics for public employee retirement systems.

8.4.8 Special Topics: Retirement Revenue Gains and Losses

The Census Bureau's definition of governmental revenue has traditionally included only realized gains and losses on the sale of securities held for investment. Recent changes in the financial reporting practices of government-administered employee retirement systems, however, have affected that policy and resulted in an apparent inconsistency with the definition of other government revenue statistics.

As noted in Section 3.4.3, government reporting on its financial operations and condition has been

influenced by rules issued by the Governmental Accounting Standards Board (GASB). For employee retirement systems, these rules have greatly affected the basis on which "revenues" are reported. That is, the concept of "revenues" has been expanded to include all additions to the net assets under the fiduciary care of the retirement system. Under GASB rules of reporting, these additions include both realized and unrealized gains and losses on investments.

Because of these rules, employee-retirement systems have been increasingly reporting "revenues" in the same manner on the survey forms for the Census Bureau's employee retirement survey. Also, to reduce reporting burdens on government officials, a growing amount of data is now collected using standardized Comprehensive Annual Finance Reports (CAFR), many of which are available in electronic format. Efforts to separate the unrealized gains and losses from other investment income have become increasingly difficult and time-consuming. The result was investment revenue statistics that were being reported in a very inconsistent manner.

In the Fiscal Year 2002 finance survey, the Census Bureau recognized the futility of trying to collect employee retirement revenue data from retirement systems in a manner that differed from the GASB procedures they employed for their regular reporting. As a result, the Bureau reluctantly changed its definition of employee retirement investment revenues to match that of the systems reporting them: that is, it now includes both realized and unrealized gains and losses during the year.

It is important to stress that the basis for this change was procedural, or survey-based, rather than definitional. As a result, this treatment was not extended to any other revenue categories on government finances, including those of other types of insurance trust systems.

8.5 Federal Government Public Employee Retirement System Statistics

The Census Bureau develops only a limited number of statistics about the Federal Government's public employee retirement systems. As of 2005, only three systems are recognized and included in the Federal Government statistics – the Civil Service Retirement System, the Foreign Service Retirement and Disability System, and the Federal Employees' Retirement System (FERS). The Civil Service Retirement System was closed to new members on January 1, 1987, when FERS became effective. The Foreign Service System is currently open to new participants.

The FERS System requires more explanation. It consists of three components. First, all covered employees are required to participate in the Social Security System. Second, there is a small defined benefit component, whereby members have their basic Social Security coverage supplemented by employer and employee contributions. The third component is a defined contribution plan – the Thrift Savings Plan, or TSP. If employees decide to participate, they can contribute up to a set percentage of their pay (as allowed by Federal law just as for purposes of a 401K plan). The Federal Government will make a matching contribution up to a set limit (5% of employee contributions, as of 2005). For purposes of Census Bureau financial statistics on the Federal Government, only the supplement defined benefit component of FERS is classified as a public employee retirement system.

Although it is a retirement system, the Federal Social Security program is classified under "Other Federal and State Social Insurance Trust Systems." This is because the membership is not limited to Federal government employees, one of the conditions required by the Census Bureau's definition.

The following special notes apply to Federal Government employee retirement system statistics:

- Earnings on investments by Federal employee retirement systems are excluded from regular

financial statistics (as are earnings of other Federal social insurance trust systems, see Chapter 9). This is because all Federal systems (trust funds) are invested solely in Federal securities, thereby making any earnings (interest) intragovernmental transfers. Hence the earnings of investments are shown only as an exhibit code.

- With respect to expenses, only two categories apply to Federal systems – benefit payments (all five codes) and withdrawals. Note that these refer only to withdrawals from the three public employee retirement systems of the Federal Government that meet the Census Bureau definition: Civil Service, Foreign Service, and the defined benefit component of FERS.

- Cash and security holdings of Federal employee retirement systems are not classified separately unto themselves.

Table 8.1 details the codes applicable to the Federal Government for purposes of public employee retirement system statistics.

8.6 Description Pages for Public Employee Retirement System Statistics

The following pages contain all of the codes used in Census Bureau surveys on public employee retirement systems. These include the regular and exhibit statistic codes that comprise all financial categories (receipts, expenses, and cash and security holdings), as well as all descriptive statistics used in the survey. In this respect, Chapter 8 is intended to be self contained and cover all aspects of the annual, quarterly, and census of government surveys covering public employee retirement systems.[5]

The presentation is similar to that used in Chapters 4 through 7 for regular finance statistics. The codes are presented in order (receipts, expenses, cash and security holdings). Descriptive statistics are presented separately and follow the three financial categories. For all statistics, each code is defined. As appropriate, inclusions and exclusions are listed for each code, and examples are cited if necessary.

Each code is clearly identified as being part of the regular finance statistics or as being an exhibit statistic. While this is not so important from the perspective of the three surveys covering public employee retirement systems, it is useful information for understanding how these retirement statistics are incorporated into the Census Bureau's regular statistics on government finances. Codes that are sub-categories of a larger aggregate category follow in order.

The description pages for RECEIPTS, EXPENSES, AND CASH AND SECURITY HOLDINGS apply to public employee retirement systems of state and local governments only. These are followed by the description pages containing codes applicable to the Federal Government, presented separately and all together. Note that some of these codes repeat from those applicable to state and local systems.

---

[5]As of survey year 2005, the Census Bureau conducts three surveys covering the financial activity of public employee retirement systems. These are *Employee Retirement Systems of State and Local Governments* (a phase of the Census of Governments), the annual *State and Local Government Employee Retirement Systems*, and the quarterly survey *Finances of Selected Public Employee Retirement Systems.*

SUB-CATEGORY: Employee Contributions

Regular Code X01 Contributions From Local Government Employees

Definition: Amounts contributed or withheld from salaries and wages of employees, and voluntary employee contributions, to provide for retirement or survivors' benefits.

Includes: Includes reimbursements for disability payments, interest on employee loans, and interest on buyback contributions, in addition to regular employee contributions. Also includes contributions from general employees as well as those of public colleges and other institutions or dependent agencies.

Special Consideration:
Code X01 applies to state governments, in addition to all types of local governments, since many states administer retirement systems where membership is open to local government employees.

Regular Code X02 Contributions From State Government Employees

Definition: Amounts contributed or withheld from salaries and wages of employees, and voluntary employee contributions, to provide for retirement or survivors' benefits.

Includes: Reimbursements for disability payments, interest on employee loans, and interest on buyback contributions, in addition to regular employee contributions. Also includes contributions from general employees as well as those of public colleges and other institutions or dependent agencies.

Special Consideration:
This code applies only to systems administered by state governments. No locally-administered employee retirement systems are open to state government employees.

SUB-CATEGORY: Employer (Government) Contributions

Exhibit Code X04 From Parent Local Government

Definition: Amounts received from parent government that is administering the system, as the employer share of contributions for their own employees who are members of the system.

Includes: Contributions from dependent agencies of the government, if the dependent agency employees are members of the system administered by the parent government.

Special Consideration:
This code is applicable to locally-dependent retirement systems only.

Regular Code X05 From Other Governments

Definition:

- For local governments – amounts received from other local governments as employer contributions for their own employees, plus contributions from state governments to local retirement systems. The latter includes state aid (e.g., contributions on behalf of local teachers) and shared taxes (e.g., portion of taxes imposed on insurance companies), even if such amounts are received through the parent government.
- For state governments – includes amounts received from local governments as employer contributions for their own employees.

Excludes: Contributions from the parent government administering the employee retirement system, including its share as an employer (use *From Parent Local Government*, exhibit code X04 or *State Contributions To Own System, Total*, exhibit code X06) and repayment of loans made to members (nonrevenue).

Exhibit Code X06 State Contributions To Own System, Total

Definition: State government contributions to a system it administers, whether for its own employees or on behalf of local employees.

Special Considerations:

1. This is a calculated statistic. It is the sum of two other exhibit codes.
   + V87 State Contributions to Own System, on Behalf of Local Employees
   + Z99 State Contributions to Own System, on Behalf of State Employees
2. This code applies only to systems administered by state governments.

Exhibit Code V87 State Contributions to Own System, on Behalf of Local Employees

Definition: State government contributions to a system it administers, on behalf of local employees who are members of the state-administered system.

Includes: Contributions made on a "per employee" basis, as well as lump sum contributions made in support of the system. The latter can include proceeds from taxes or borrowing designated specifically for funding the retirement system.

Special Consideration:
This code applies only to systems administered by state governments.

Exhibit Code Z99 State Contributions to Own System, on Behalf of State Employees

Definition: State government contributions to a system it administers, on behalf of a state's own employees who are members of the state-administered system.

Exhibit Code Z99 State Contributions to Own System, on Behalf of State Employees - continued

Includes: Contributions made on a "per employee" basis, as well as lump sum contributions made in support of the system. The latter can include proceeds from taxes or borrowing designated specifically for funding the retirement system. Also includes contributions on behalf of, or for, employees of dependent agencies, as defined by the Census Bureau.

Special Consideration:
This code applies only to systems administered by state governments.

SUB-CATEGORY: Other Receipts

Regular Code X08 Total Earnings on Investments

Definition: Total earnings on investments, net of losses, as summed from all the earnings codes defined below. Includes interest earnings, dividends, profits on investment transactions (minus any losses), rentals; and other earnings on investments. Includes unrealized gains (and losses), as reported for accounting purposes.

Excludes: Rental of property owned by a retirement system and leased to its parent government, and amortization of discounts (nonrevenues).

Special Considerations:
1. This is a calculated statistic. It is the sum of the following six exhibit codes.
    +Z98 Rentals from State Government
    +Z71 Interest Earnings
    +Z72 Dividend Earnings
    +Z73 Other Investment Earnings
    +Z96 Gains on Investments (Realized and Unrealized)
    - Z91 Losses on Investments (Realized and Unrealized)

2. For Census Bureau Regular Statistics on government finance, *Rentals from State Government*, code Z98, is excluded from calculation of code X08, *Earnings on Investments*. Such receipts reflect an intragovernmental transaction and thus cannot be included as "revenue" for a government.

3. Previous *Classification Manuals* separated earnings on investments between interest income (code X08) and other types of investment earnings (X09). However, in practice this distinction was not made on the survey schedules themselves. Consequently, these two codes were consolidated effective with fiscal year 1990 data.

Exhibit Code Z71 Interest Earnings

Definition: Regular interest earnings on investment securities, deposits, and other interest-bearing accounts, including accrued interest on investment securities sold.

Exhibit Code Z72 Dividend Earnings

Definition: Revenue from the distribution of earnings by a corporation for which the system has an ownership claim, based on the purchase of investments (such as stocks) in that corporation.

Excludes: Interest earnings (report at *Interest Earnings*, code Z71).

Special Consideration:
This classification category was introduced effective with fiscal year 2002. Previously, dividends were essentially combined with *Interest Earnings*, code Z71.

Exhibit Code Z98 Rentals from State Government

Definition: Rentals of property owned and leased by the system to the parent government administering the system.

Special Considerations:

1. This code is NOT included in the formula for calculating the regular finance variable *Earnings on Investments*, code X08. This follows from the fact that such a transaction would be an intragovernmental transfer (one fund of the government to another).
2. For Retirement Survey purposes, Z98 is included in the calculation of *Earnings on Investments*, code X08.
3. This code applies only to systems administered by state governments.

Exhibit Code Z73 Other Investment Earnings

Definition: Earnings from investments not elsewhere classified.

Includes: Rentals from property owned and leased by the system, except for rentals from the parent government administering the system (report at *Rentals from State Government*, code Z98).

Exhibit Codes Z91 and Z96 NET GAINS/LOSSES ON INVESTMENTS

Definition: The overall net gain or loss on investments. This code is comprised of two components. For investments sold during the fiscal year, the net realized gain or loss is recorded. For investments held at the end of the fiscal year, their unrealized gains or losses are recorded and then aggregated. Unrealized gains and losses are measured as noted in Section 8.4.1.1, using the difference between the market value of the asset at the end of the current fiscal year and at the end of the previous fiscal year.

Exhibit Codes Z91 and Z96 NET GAINS/LOSSES ON INVESTMENTS – continued

Special Considerations:

1. The Census Bureau currently reports only one of these codes per retirement system, depending on whether the system gained or lost money during the fiscal year. In theory, both could be used, as long as investments that earned money were separated from investments that lost money during the fiscal year. That is not what is done in practice, however.

2. For arithmetic purposes, both of these codes are recorded as positive numbers. If code Z96 is recorded, then it is "added" in to the components for computing total *Earnings on Investments*, X08. If the system experienced net combined losses from the sale and valuation of investments it holds, then Z96 is used and the amount is deducted when computing total *Earnings on Investments*, X08.

Exhibit Code Z95 Other Receipts, NEC

Definition: Private gifts, donations, and other receipts not classified elsewhere.

***

Regular Code X11 Total Benefit Payments

Definition: Distribution of cash benefits to eligible persons under government-administered employee retirement systems covering public employees.

Excludes: Payments of premiums for annuity policies from private insurance carriers, contributions to pension plans administered by private companies or by public employee associations, "pay-as-you-go" pensions and other direct payments to retired employees from current appropriations (report preceding items as current operations expenditure of function involved), and loans to members.

Special Considerations:

1. This is a calculated statistic. It is the sum of four exhibit codes.
   + Z13 Retirement Benefits
   + Z14 Disability Benefits
   + Z15 Survivor Benefits
   + Z16 Withdrawals
2. Prior to 2005, Code X11 was a reported variable, with no breakout of exhibit codes Z13 – Z16.

Exhibit Code Z13 Retirement Benefits

Definition: Cash payments to (or on behalf of) members for retirement benefits and annuities.

Exhibit Code Z14 Disability Benefits

Definition: Cash payments to (or on behalf of) members for disability benefits and annuities.

Exhibit Code Z15 Survivor Benefits

Definition: Cash payments to (or on behalf of) qualified survivors of deceased members, commonly referred to as survivor benefits.

Exhibit Code Z16 Other Benefits

Definition: Life and disability insurance on behalf of retirees, pre-retirement death benefit premiums, benefits due on termination of employment, and other benefits as allowed.

Regular Code X12 Withdrawals

Definition: Lump sum amounts paid to employees, former employees or their survivors, representing a return of contributions made by employees during the period of their employment.

Includes: Interest credited on contributions and paid with the withdrawal.

Regular Code X12 Withdrawals - continued

Excludes: Loans to members.

Exhibit Code Z93 Administrative Expenses

Definition: The cost of administering the retirement system, including salaries of employees, investment fees, building rentals or costs, and the like.

Special Considerations:

1. Administrative expenses are treated as a general expenditure in regular Census Bureau statistics on government finances. Most are reported at *Financial Administration*, code *23 although there could be some variation depending on the transaction.

2. Formerly reported at X14, along with *Other Payments*, exhibit code Z90.

3. In Census Bureau statistics on public employment, employees of public employee retirement systems are classified at *Financial Administration*, code *23. There is no Insurance Trust Sector for purpose of the public employee statistics program. See Chapters 11 and 12.

Special Consideration for Exhibit Codes:

Code Z90 is found on some survey questionnaires, but is not part of the classification system. It is a cell for data collection purposes only. Any amounts reported are evaluated and moved to an appropriate, valid classification code.

The Census Bureau uses additional "By Type" classification categories for cash and securities of public employee retirement systems. The applicable "By Type" codes are listed here, along with their descriptions and special considerations, if any. In order to classify public employee retirement systems cash and security holdings fully, the Census Bureau uses both regular codes for statistics on government finance and the exhibit codes for statistics on employee retirement systems. Both types of codes are defined below.

Regular Code X21 Total Cash and Short-Term Investments

Definition: Cash on hand and on deposit; demand deposits; time or savings deposits; certificates of deposits; repurchase agreements ("repos"); reverse repurchase agreements ("reverse repos"); commercial and finance company paper; bankers acceptances; and money market funds.

Special Consideration:
This is a calculated statistic. It is the sum of three exhibit codes.
+ Z88 Cash on Hand and Demand Deposits
+ Z87 Time or Savings Deposits
+ Z68 All Other Short-Term Investments

Exhibit Code Z88 Cash on Hand and Demand Deposits

Definition: Cash and demand deposits.

Exhibit Code Z87 Time or Savings Deposits

Definition: Regular savings deposits and certificates of deposit (time deposits).

Exhibit Code Z68 All Other Short-Term Investments

Definition: Securities in repurchase agreements, reverse repurchase agreements, commercial and finance company paper, bankers' acceptances, and miscellaneous money market funds.

Regular Code X30 Total Federal Government Securities

Definition: Obligations of the U.S. Treasury, the Federal Financing Bank (FFB), as well as bonds and mortgage-backed securities issued by Federal agencies. See definitions for *Federal Agency Securities,* code X33, and *Federal Treasury Securities,* code Z89, for detail.

Excludes: Mortgages directly held by employee retirement system (use *Mortgages Held Directly*, code X42) and securities of Federally-Sponsored or chartered private and quasi-private credit organizations, such as the Federal National Mortgage Association, FNMA or "Fannie Mae" (use *Corporate Bonds, Federally-Sponsored Agencies*, code Z62).

Regular Code X30 Total Federal Government Securities - continued

Special Considerations:

1. This is a calculated statistic, representing the sum of two exhibit codes.
   + Z89 Federal Treasury Securities
   + X33 Federal Agency Securities

2. Prior to fiscal year 1988 data, these securities were differentiated between Federal Treasury notes, code X30, and *Federal Agency Securities*, code X33. Now, X30 is the total holdings, while Z89 represents the *Federal Treasury Securities*.

Exhibit Code X33 Federal Agency Securities

Definition: Bonds and mortgage-backed securities issued by Federal agencies, such as the Commodity Credit Corporation (CCC), Export-Import Bank, Federal Housing Administration (FHA), Financing Corporation (FICO), Government National Mortgage Association (GNMA, or "Ginnie Mae"), Federal Savings and Loan Insurance Corporation (FSLIC), Small Business Administration (SBA), U.S. Postal Service, and Tennessee Valley Authority (TVA).

Exhibit Code Z89 Federal Treasury Securities

Definition: Obligations of the U.S. Treasury (including short-term notes) and the Federal Financing Bank (FFB).

Regular Code Z77 Total Corporate Bonds

Definition: Debentures and related private corporate bonds, as well as securities of Federally-Sponsored or chartered private and quasi-private organizations. See definition under *Corporate Bonds, Federally-Sponsored Agencies*, code Z62, and *Corporate Bonds, Other*, code Z63, for detail.

Special Considerations:

1. This is a calculated statistic. It represents the sum of two exhibit codes.
   + Z62 Corporate Bonds, Federally-Sponsored Agencies
   + Z63 Corporate Bonds, Other

2. Prior to 2002, corporate bonds were reported at book value (i.e., their original cost less any discounts). The code used for that category was X40.

3. Code Z77 was added to regular finance statistics effective with survey year 2002. Prior to that, it was an exhibit code used occasionally during Census of Government surveys only.

Exhibit Code Z62 Corporate Bonds, Federally-Sponsored Agencies

Definition: Securities of Federally-Sponsored or chartered private and quasi-private organizations like Federal Home Loan Banks (FHLB), Federal Home Loan Mortgage Corporation (FHLMC, or "Freddie Mac"), Federal National Mortgage Association (FNMA, or "Fannie Mae"), Federal farm credit banks, and Student Loan Marketing Association (SLMA, or "Sallie Mae").

Exhibit Code Z63 Corporate Bonds, Other

Definition: Debentures, convertible bonds, and railroad equipment certificates.

Regular Code Z78 Corporate Stocks

Definition: Common and preferred stock, warrants, and shares in investment companies. Report at market ("fair") value, as of the end of the system's fiscal year.

Special Considerations:

1. Prior to 2002, these securities were reported at their book value (i.e., their original cost less any discounts), not at their current market value. The code used was X41.
2. Code Z78 was added to regular finance statistics effective with 2002. Prior to that, it was an exhibit code used occasionally during Census of Government surveys.

Regular Code X42 Mortgages Held Directly

Definition: Mortgages held by (owed to) the public employee retirement system and issued as an investment.

Excludes: Mortgage-backed Federal securities (use *Total Federal Government Securities,* code X30) and real property directly owned by system (use *Real Property*, exhibit code X46).

Regular Code X44 Total Other Securities

Definition: Securities of state and local governments, investments held in trust by other agencies, shares held in mutual funds; foreign and international securities, pooled life insurance investments, direct loans and loans to system members. See definitions for *State and Local Government Securities*, code X35, *Foreign and International Securities,* code Z70, *Other Securities,* code Z83, and *Investments Held in Trust by Other Agencies,* code Z84, for additional detail.

Regular Code X44 Total Other Securities - continued

Special Considerations:

1. This is a calculated statistic. It is the sum of four exhibit codes.
   + Z84 Investments Held in Trust
   + X35 State and Local Government Securities
   + Z70 Foreign and International Securities
   + Z83 Other Securities

2. Securities of state and local governments were added to this category effective with fiscal year 1988 data. Prior to that time, they were separately identified in regular government finance statistics and reported at *State and Local Government Securities*, code X35.

3. Foreign and International Securities, code Z70, was reported as code Z69 from 1997 to 2001.

Exhibit Code X35 State and Local Government Securities

Definition: Securities of state and local governments, including those issued by parent government administering the system and purchased for investment purposes.

Exhibit Code Z70 Foreign and International Securities

Definition: Securities of foreign and international private companies, foreign-sponsored agencies, and foreign national governments (central or otherwise).

Includes: Corporate equities and stocks.

Special Consideration:
Foreign and International Securities, code Z70, was reported as code Z69 from 1997 to 2001.

Exhibit Code Z84 Investments Held in Trust by Other Agencies

Definition: Investments held in trust by other agencies.

Includes: Funds administered by private agencies, guaranteed investment accounts, and system shares of funds in governmental investment accounts (including pooled investment accounts). Pooled investment accounts are allocated when sufficient information is given.

Exhibit Code Z83 Other Securities

Definition: All other securities not classified elsewhere.

Includes: Shares held in mutual funds, conditional sales contracts, direct loans, and loans to members.

Exhibit Code Z82 Total Other Investments

Definition: Other investments not elsewhere classified. See *Real Property*, code X46, and *Other Investments*, code X47, for detailed definitions.

Special Considerations:

1. This is a calculated statistic. It is the sum of two other codes.
   + X46 Real Property
   + X47 Other Investments
2. X46 is, by definition, excluded from the Census Bureau's regular finance statistics. Therefore, Z82 also is excluded. Instead, X47 is used in the regular finance statistics to represent *Total Other Investments* for public employee retirement systems.

Exhibit Code X46 Real Property

Definition: Value of real property owned by the system (directly held).

Excludes: Property held in real estate investment trusts and pooled or partnership agreements (report at *Other Investments,* code X47).

Regular Code X47 Other Investments

Definition: All other investments of the retirement system, not classified in other categories.

Includes: Venture capital, partnerships, real estate investment trusts, pooled or partnership agreements, and leveraged buyouts.

Excludes: Real property held directly by system (use *Real Property,* exhibit code X46).

Special Considerations:

1. Prior to fiscal year 1988 data, this category was classified as an exhibit code.
2. For public employee retirement statistics, this category is combined with *Real Property*, exhibit code X46, to create *Total Other Investments,* exhibit code Z82.
3. For regular finance statistics, this category is used to represent *Other Investments* of public employee retirement systems, in place of code Z82. The latter is not used because it includes *Real Property*, code X46, which is excluded from regular finance statistics by definition.

Exhibit Code Z81 Total Cash and Security Holdings of Public Employee Retirement System

Definition: Sum of all investments, including all exhibit code categories and regular code categories.

Special Considerations:

1. This code serves two purposes. It is a convenient aggregate for users, and also is used for Census Bureau processing purposes.

Exhibit Code Z81 **Total Cash and Security Holdings of Public Employee Retirement System** - continued

Special Considerations: (continued)

2. This total is neither reported for individual governments, nor is it in summary tables or files, for regular finance statistics. It is found only in data files containing public employee retirement system financial statistics.

The following codes are used by the Census Bureau to develop important information about the characteristics of public employee retirement systems. Amounts reported for these codes are excluded from the Census Bureau's general statistics on government finances (referred to as regular finance statistics). Amounts also are excluded from the financial statistics on individual public employee retirement systems (regular and exhibit finance statistics). The Census Bureau uses these descriptive statistics primarily to identify and define the structure and organization of the government sector, a major component of the Census of Governments. However, these descriptive statistics are released to the public as well, since many users have expressed an interest in them.

Note that these statistics are collected intermittently. Some are annual and others are collected only at five year intervals, during each Census of Governments. The measurement period for these statistics varies. Descriptive statistics involving financial transactions are defined to reflect the transaction level for the last month of a system's fiscal year.

Two final notes regarding these descriptive statistics are important. First, V** codes, below, are not aggregated in Census Bureau reports. These are used and reported strictly at the individual retirement system level. Secondly, descriptive statistics apply only to public employee retirement systems of state and local governments.

Descriptive Code Z01 Number of Active Members

Definition: Current contributors, and/or current employees covered by the system.

Collected: Annually.

Descriptive Code Z75 Active Members Employed by Local Governments

Definition: The number of current members of a state-administered system who are employed by local governments.

Special Consideration:
This code applies only to systems administered by state governments.

Descriptive Code Z76 Active Members Employed by State Government

Definition: The number of current members of a state-administered system who are employed by the state government or its dependent agencies.

Special Consideration:
This code applies only to systems administered by state governments.

Descriptive Code Z02 Number of Inactive Members

Definition: Former employees or employees on military or extended leave without pay, having retained retirement credits but not currently receiving retirement benefit payments.

Collected: Annually.

Descriptive Code Z03 Number of Beneficiaries Retired, Age or Service

Definition: The number of beneficiaries receiving periodic benefit payments during the month who are formerly active members of the system retired on account of age or length of service

Collected: Annually.

Descriptive Code Z04 Number of Beneficiaries Retired, Disability

Definition: The number of beneficiaries receiving periodic benefit payments during the month who are formerly active members of the system retired on account of disability.

Collected: Annually.

Descriptive Code Z05 Number of Beneficiaries, Survivors

Definition: The number of beneficiaries receiving periodic benefit payments during the month who are survivors of deceased former active members.

Collected: Annually.

Descriptive Code Z06 Number of Recipients of Lump-sum Payments, Current or Former Members

Definition: The number of current or former members of the system receiving lump-sum withdrawals, other than loans, during the month.

Collected: Annually.

Descriptive Code Z07 Number of Recipients of Lump-sum Payments, Survivors

Definition: The number of survivors of current or former members of the system who were paid lump-sum withdrawals during the month.

Collected: Annually.

Descriptive Code Z08 Monthly Amount Paid, To Retirees

Definition: Amount paid during the month to former active members retired on account of age or length of service, or some combination thereof.

Collected: Annually.

Special Consideration:
This amount differs from expense *Retirement Benefits*, code Z13, in that it reflects monthly payments during the last month of the fiscal year.

Descriptive Code Z09 Monthly Amount Paid, Disability

Definition: Amount paid during the month to former active members retired on account of disability.

Collected: Annually.

Special Consideration:
This amount differs from expense *Disability Benefits*, code Z14, in that it reflects monthly payments during the last month of the fiscal year.

Descriptive Code Z10 Monthly Amount Paid, To Survivors

Definition: Amount paid during the month to survivors of deceased former active members.

Collected: Annually.

Special Consideration:
This amount differs from expense *Survivor Benefits*, code Z15, in that it reflects monthly payments during the last month of the fiscal year.

Descriptive Code Z11 Monthly Amount Paid, Lump-sum, To Members

Definition: Withdrawals and other one-time payments (other than loans) made to current or former members of the system.

Excludes: Loans made to members.

Collected: Annually.

Special Consideration:
This amount differs from expense *Withdrawals*, code X12, in that it reflects withdrawals paid during the last month of the fiscal year, to current or former members (excluding survivors of current or former members).

Descriptive Code Z12 Monthly Amount Paid, Lump-sum, To Survivors

Definition: Withdrawals and other one-time payments (other than loans) made to survivors of deceased former members of the system.

Excludes: Loans made to members.

Collected: Annually.

Special Consideration:
This amount differs from expense *Withdrawals*, code X12, in that it reflects the withdrawals paid during the last month of the fiscal year, to survivors of deceased former members of the system only (excluding payments to current or former surviving members).

Descriptive Code V01 Description of Retirement System

Definition: Code used to characterize the system.
1 = Contributions for retirement are forwarded to a private insurance carrier as premiums paid for the purchase of annuity policies for the members of the plan.
2 = Members of plan belong to the Teachers Insurance and Annuity Association (TIAA) without any state or locally administered supplemental retirement coverage.
3 = Payment of service, disability, or survivor benefits to eligible persons are paid directly from general funds appropriated annually. There is no separate retirement system fund.
4 = System is administered by the sponsoring government and is subject to the accounting and auditing controls of that government.

Collected: Census of Governments only (every five years).

Special Consideration:
This code is used to determine eligibility within the Census Bureau classification system for public employee retirement systems.

Descriptive Code V02 Type of Coverage

Definition: Code used to characterize the type of coverage provided by the system.
1 = Policemen only
2 = Firemen only
3 = Policemen and firemen only
4 = School employees only – Including non-teaching personnel as well as teachers
5 = Teachers only – Instructional staff (including supervisory personnel, but not other school employees)
6 = Other specific group(s)
7 = General coverage – All employees (or all regular or full-time employees), subject only to the following exclusions – Specify

Collected: Census of Governments only (every five years).

Descriptive Code V03 Basis of Membership and Participation

Definition: Code used to characterize the basis of membership for the system.
1 = System coverage automatically applies to all eligible employees
2 = System coverage automatically applies to some eligible employees, but with membership optional for certain classes
3 = System coverage is optional (by employee choice) for all eligible employees

Collected: Census of Governments only (every five years).

Descriptive Code V04 Employee Contribution Status

Definition: Code used to characterize whether member employees contribute to the system.
1 = Yes
2 = No

Collected: Census of Governments only (every five years)

Descriptive Code V05 Optional Benefits Available

Definition: Code used to characterize whether optional benefits are available to members.
1 = Yes
2 = No

Collected: Census of Governments only (every five years).

Descriptive Code V07 Time Required to Vest

Definition: The minimum time period required for employees before they are vested (qualified for retirement benefits) under the system rules. The time is reported in years, as a single response.

Collected: Census of Governments only (every five years).

Regular Code X03 Contributions From Federal Government Employees

Definition: Amounts contributed from salaries and wages of Federal employees. Includes mandatory amounts withheld and voluntary contributions, if any. Includes reimbursements for disability payments, interest on employee loans, and interest on buyback contributions.

Exhibit Code X99 Interest on U.S. Securities Held By Federal Retirement Systems

Regular Code X11 Total Benefit Payments

Definition: Distribution of cash benefits to eligible persons under government-administered employee retirement systems covering public employees.

Excludes: Pensions paid out of Federal Military Retirement Fund. These are reported at *Federal National Defense and International Relations*, code *06, in the general government sector.

Special Considerations:

1. This is a calculated variable. It is the sum of four exhibit codes.
   + Z13 Retirement Benefits
   + Z14 Disability Benefits
   + Z15 Survivor Benefits
   + Z16 Other Benefits

2. Prior to 2005, X11 was a reported variable, with no breakout of exhibit codes Z13 – Z16.

Exhibit Code Z13 Retirement Benefits

Definition: Cash payments to (or on behalf of) members for retirement benefits and annuities, and payments to (or on behalf of) qualified survivors of deceased members, commonly referred to as survivor benefits.

Exhibit Code Z14 Disability Benefits

Definition: Cash payments to (or on behalf of) members for disability benefits and annuities.

Exhibit Code Z15 Survivor Benefits

Definition: Cash payments to (or on behalf of) qualified survivors of deceased members, commonly referred to as survivor benefits.

Exhibit Code Z16 Other Benefits

Definition: Life and disability insurance on behalf of retirees, pre-retirement death benefit premiums, benefits due on termination of employment, and other benefits as allowed.

Regular Code X12 Withdrawals

Definition: Lump-sum amounts paid to employees, former employees or their survivors, representing a return of contributions made by employees during the period of their employment.

Includes: Interest credit on such contributions and repaid along with the withdrawal.

Excludes: Loans to members.

Special Consideration:
This applies only to withdrawals paid under the Civil Service and Foreign Service retirement systems, plus the defined benefit component of the FERS program. Withdrawals of funds from the TSP component of FERS are excluded.

***

8.7 Tables

There are two tables associated with this chapter. Table 8.1, Applicability of Public Employee Retirement System Codes, by Level and Type of Government, shows how the statistical codes apply to governments, by level and type. Where codes apply, they are valid and the implication is that data could exist for that type of financial category or transaction. Table 8.2, Summary of Public Employee Retirement System Codes, lists all the codes in the classification system that pertain to public employee retirement systems, and indicates what type of statistics they represent (regular, exhibit or descriptive). There are no derived statistical codes for public employee retirement systems. In addition to these two tables, Appendix Table 1.2, Public Employee Retirement System Regular and Exhibit Variables, Current and Historical, contains useful information about how the classification categories for public employee retirement systems have changed since 1987.

**Table 8.1**<br>**Applicability of Public Employee Retirement System Codes,**<br>**by Level and Type of Government –** page 1 of 2					
**Category**	**Code**	**Type**	**Federal**	**State**	**Local - All types**
**Receipts**					
Contributions, Local Employees	X01	R	X	Valid	Valid
Contributions, State Employees	X02	R	X	Valid	X
Contributions, Federal Employees	X03	R	Valid	X	X
Contributions, From Parent Local Government	X04	E	X	X	Valid
Contributions, From Other Governments	X05	R	X	Valid	Valid
State Contributions to Own System, Total	X06	E	X	Valid	X
State Own Contributions for Local Employees	V87	E	X	Valid	X
State Own Contributions for State Employees	Z99	E	X	Valid	X
Total Earnings on Investments	X08	R	X	Valid	Valid
Interest	Z71	E	X	Valid	Valid
Dividends	Z72	E	X	Valid	Valid
Rentals, from State Government	Z98	E	X	Valid	X
Other Investment Earnings	Z73	E	X	Valid	Valid
Losses on Investments	Z91	E	X	Valid	Valid
Gains on Investments	Z96	E	X	Valid	Valid
Other Receipts	Z95	E	X	Valid	Valid
Interest on Federal Retirement System Holdings	X99	E	Valid	X	X
**Expenses**					
Total Benefit Payments	X11	R	Valid	Valid	Valid
Retirement Benefits	Z13	E	Valid	Valid	Valid
Disability Benefits	Z14	E	Valid	Valid	Valid
Survivor Benefits	Z15	E	Valid	Valid	Valid
Other Benefits	Z16	E	Valid	Valid	Valid
Withdrawals	X12	R	Valid	Valid	Valid
Administrative Expenses	Z93	E	Valid	Valid	Valid
**Cash and Security Holdings**					
Total Cash and Short-Term Investments	X21	R	X	Valid	Valid
Cash on Hand and Demand Deposits	Z88	E	X	Valid	Valid
Time or Savings Deposits	Z87	E	X	Valid	Valid
All Other Short-term Investments	Z68	E	X	Valid	Valid
Total Federal Government Securities	X30	R	X	Valid	Valid
Federal Treasury Securities	Z89	E	X	Valid	Valid
Federal Agency Securities	X33	E	X	Valid	Valid
Total Corporate Bonds	Z77	R	X	Valid	Valid
Bonds of Federally-Sponsored Agencies	Z62	E	X	Valid	Valid
Corporate Bonds, Other	Z63	E	X	Valid	Valid
Corporate Stocks	Z78	R	X	Valid	Valid

Table 8.1
Applicability of Public Employee Retirement System Codes,
by Level and Type of Government – page 2 of 2

Category	Code	Type	Federal	State	Local - All types
**Cash and Security Holdings** - continued					
Mortgages Held Directly	X42	R	X	Valid	Valid
Total Other Securities	X44	R	X	Valid	Valid
Investments Held in Trust	Z84	E	X	Valid	Valid
State and Local Government Securities	X35	E	X	Valid	Valid
Foreign and International Securities	Z70	E	X	Valid	Valid
Other Securities	Z83	E	X	Valid	Valid
Total Other Investments	Z82	E	X	Valid	Valid
Real Property	X46	E	X	Valid	Valid
Other Investments	X47	R	X	Valid	Valid
Total Cash and Security Holdings	Z81	E	X	Valid	Valid
**Descriptive Statistics**					
Number of Active Members	Z01	D	X	Valid	Valid
Members Employed by Local Governments	Z75	D	X	Valid	X
Members Employed by State Government	Z76	D	X	Valid	X
Number of Inactive Members	Z02	D	X	Valid	Valid
Number of Beneficiaries Retired, Age or Service	Z03	D	X	Valid	Valid
Number of Beneficiaries Retired, Disability	Z04	D	X	Valid	Valid
Number of Beneficiaries, Survivors	Z05	D	X	Valid	Valid
Number of Recipients of Lump-sum, Members	Z06	D	X	Valid	Valid
Number of Recipients of Lump-Sum, Survivors	Z07	D	X	Valid	Valid
Monthly Amount Paid - Retired, Age or Service	Z08	D	X	Valid	Valid
Monthly Amount Paid - Retired, Disability	Z09	D	X	Valid	Valid
Monthly Amount Paid - Survivors	Z10	D	X	Valid	Valid
Monthly Amount Paid - Withdrawals	Z11	D	X	Valid	Valid
Monthly Amount Paid - Lump-Sum Payments	Z12	D	X	Valid	Valid
Description of System	V01	D	X	Valid	Valid
Type of Coverage	V02	D	X	Valid	Valid
Basis of Membership and Participation	V03	D	X	Valid	Valid
Employee Contribution Status	V04	D	X	Valid	Valid
Optional Benefits Availability	V05	D	X	Valid	Valid
Time Required to Vest	V07	D	X	Valid	Valid

Key:
X Not a valid code for the level or type of government indicated (there are X statistics).
Valid Applicable code for the level or type of government indicated (statistics are collected and reported).

Type of Statistic (see Chapter 8.2):
R Regular
E Exhibit
D Descriptive

Table 8.2<br>**Summary of Public Employee Retirement System Codes** – page 1 of 2				
**Finance code**	**Brief description**	**Regular code**	**Exhibit code**	**Descriptive code**
	**Receipts:**			
X01	Contributions, Local Employees	Yes	-	-
X02	Contributions, State Employees	Yes	-	-
X03	Contributions, Federal Employees.	Yes	-	-
X04	Contributions, From Parent Local Government	-	Yes	-
X05	Contributions, From Other Government	Yes	-	-
X06	State Contributions to Own System, Total	-	Yes	-
V87	State Own Contributions for Local Employees	-	Yes	-
Z99	State Own Contributions for State Employees	-	Yes	-
X08	Total Earnings on Investments	Yes	-	-
Z71	Interest	-	Yes	-
Z72	Dividends	-	Yes	-
Z98	Rentals from State Government	-	Yes	-
Z73	Other Investment Earnings	-	Yes	-
Z91	Losses on Investments	-	Yes	-
Z96	Gains on Investments	-	Yes	-
Z95	Other Receipts	-	Yes	-
X99	Interest on Federal Retirement System Holdings	-	Yes	-
	**Expenses:**			
X11	Total Benefit Payments	Yes	-	-
Z13	Retirement Benefits	-	Yes	-
Z14	Disability Benefits	-	Yes	-
Z15	Survivor Benefits	-	Yes	-
Z16	Other Benefits	-	Yes	-
X12	Withdrawals	Yes	-	-
Z93	Administrative Expense	-	Yes	-
	**Cash and Security Holdings:**			
Z81	Total Cash and Security Holdings	-	Yes	-
X21	Total Cash and Short-Term Investments	Yes	-	-
Z88	Cash on Hand and Demand Deposits	-	Yes	-
Z87	Time or Savings Deposits	-	Yes	-
Z68	All Other Short-Term Investments	-	Yes	-
X30	Total Federal Government Securities	Yes	-	-
Z89	Federal Treasury Notes	-	Yes	-
X33	Federal Agency Securities	-	Yes	-

Table 8.2<br>**Summary of Public Employee Retirement System Codes –** page 2 of 2				
**Finance code**	**Brief description**	**Regular code**	**Exhibit code**	**Descriptive code**
	**Cash and Security Holdings:** (continued)			
Z77	Total Corporate Bonds	Yes	-	-
Z62	Federally-Sponsored Agencies	-	Yes	-
Z63	Corporate Bonds, Other	-	Yes	-
Z78	Corporate Stocks	Yes	-	-
X42	Mortgages Held Directly	Yes	-	-
X44	Total Other Securities	Yes	-	-
Z84	Investments Held in Trust by Other Agencies	-	Yes	-
X35	State and Local Government Securities	-	Yes	-
Z70	Foreign and International Securities	-	Yes	-
Z83	Other Securities	-	Yes	-
Z82	Total Other Investments	-	Yes	-
X46	Real Property	-	Yes	-
X47	Other Investments	Yes	-	-
	**Descriptive Statistics:**			
Z01	Number of Active Members	-	-	Yes
Z75	Members Employed by Local Government	-	-	Yes
Z76	Members Employed by State Government	-	-	Yes
Z02	Number of Inactive Members	-	-	Yes
Z03	Number of Beneficiaries Retired, Age or Service	-	-	Yes
Z04	Number of Beneficiaries Retired, Disability	-	-	Yes
Z05	Number of Beneficiaries, Survivors	-	-	Yes
Z06	Number of Recipients - Lump-sum, Members	-	-	Yes
Z07	Number of Recipients - Lump-sum, Survivors	-	-	Yes
Z08	Monthly Amount Paid - Retired, Age or Service	-	-	Yes
Z09	Monthly Amount Paid - Retired, Disability	-	-	Yes
Z10	Monthly Amount Paid - Survivors	-	-	Yes
Z11	Monthly Amount Paid - Withdrawal	-	-	Yes
Z12	Monthly Amount Paid - Lump Sum Payments	-	-	Yes
V01	Description of System	-	-	Yes
V02	Type of Coverage	-	-	Yes
V03	Basis of Membership and Participation	-	-	Yes
V04	Employee Contribution Status	-	-	Yes
V05	Optional Benefits Available	-	-	Yes
V07	Time Required to Vest	-	-	Yes

Key: "Yes" Indicates applicable for that type of statistic.
- Indicates not applicable for that type of statistic.

Chapter 9. Federal and State Social Insurance Trust Systems, Other Than Public Employee Retirement

Introduction

As noted in Chapter 2 and described also in Chapter 8, the social insurance trust sector is comprised of two major groups:

- Public employee retirement systems, embracing both contributory and noncontributory systems administered by a government for public employees (including employees of other governments). These are detailed in Chapter 8.

- Federal and state social insurance trust systems other than public employee retirement systems, including the unemployment compensation system, state government workers' compensation programs, and other state social insurance trusts, and Federal social insurance trusts. The latter consist of the Federal Government's Social Security and Medicare program (OASDHI), veterans' life insurance, and railroad retirement. These types of other social insurance systems are not applied to local government finances, with the exception of the District of Columbia's unemployment compensation plan.

To be categorized as a social insurance trust for purposes of Census Bureau statistics, a system must meet all five characteristics of a social insurance trust described below in Section 9.1. As of 2005, many state governments were beginning to expand their activity in the area of social programs. Included are disaster assistance programs, as well as health care coverage programs. The latter include coverage for retirees and, in some cases, coverage for selected groups of citizens such as children under a certain age. These will be classified, by sector, in accordance with standard Census Bureau methodology on a case-by-case basis.

This chapter has no applicability to the Census Bureau's statistical program on public employment. This follows from the Census Bureau's definition of government sectors. For purposes of *regular finance statistics*, all administrative costs associated with other social insurance trust systems are classified in the general government sector, by definition. Consequently, all employees engaged in the activity of administering these systems also are classified in the general government sector.

9.1 Social Insurance Trust System Definition

To be categorized as a social insurance trust system for Census Bureau purposes, a government's system must meet all of the following criteria:[1]

1. The system must be financed by a separate accounting fund of the administering government. This criterion excludes many "pay-as-you-go" plans.

2. This fund must have some type of assured revenue stream or dedicated revenue source other than appropriations from the administering government (generally, contributions or premiums imposed on its members and/or member employers).

[1]Note that of the four types of insurance trust systems recognized by the Census Bureau, only the public employee retirement systems category applies to local government finances (plus the District of Columbia's unemployment compensation system). All other types apply to Federal and/or state governments.

3. The system must be a social insurance plan – that is, it must have a social purpose such as benefiting the disabled or disadvantaged, aiding individuals who cannot afford private insurance, providing future income, and the like.

4. The members of the system must be outside the government itself – that is, the plan cannot be for insuring the government itself against risks. For this criterion, public employees are considered to be outside the government.

5. The plan must be administered directly by the government itself – that is, it cannot just collect contributions and then turn them over to a private insurer who assumes the actual risks and administers the plan.

The Federal Government administers a number of other insurance-type programs, such as crop and farm mortgage insurance, investment guarantees, and home mortgage insurance (e.g., FHA and VA). For Census Bureau purposes, these are reported in the general government sector rather than as insurance trusts, because they do not meet all of the criteria contained in the Census Bureau's definition, outlined above.

Note that the insurance trust category applies only to the government actually administering the system. A government's participation in an insurance trust administered either privately or by another government is not treated as an insurance trust activity of the participating government. Generally, it would be treated as a current operations expenditure for the paying activity in the general government, utilities, or liquor stores sector.

9.1.1 Examples of Social Insurance Trust Systems Included by this Definition

The examples below illustrate the types of insurance programs (other than for public employee retirement) that meet the above criteria. Note that as of this edition of Classification Manual, "other" social insurance trust systems are confined to those administered by the Federal Government and the state governments only, with one exception for the Washington, DC unemployment insurance trust system.

- For the Federal Government, the Railroad retirement system is included. It provides retirement-survivor benefits to railroad workers and their families.

- Many, but not all, state governments operate a workers' compensation insurance trust system. One example of such a system that meets the Census Bureau criteria noted above is the New York State Workers Compensation Board.

- A state government insurance plan to protect homeowners against certain catastrophic risks, either because private insurance is unavailable or unaffordable, would meet the social criteria test and would be classified as an insurance trust as long as it met the other four criteria. The Citizens Property Insurance Corporation in Florida is an example.

9.1.2 Examples of Social Insurance Trust Systems Excluded by this Definition

The following examples illustrate the types of insurance programs (other than for public employee retirement) that do not meet the above criteria:

- Since the Federal Government's own workers' compensation program is a "pay-as-you-go" plan funded by agency operating funds and having no separate accounting fund, it fails the first two criteria for being an insurance trust system.

- Some state governments operate pooled, self-insurance programs providing general liability insurance to local governments within the state, funded by premiums levied against its members. Generally, their purpose is to provide a low-cost alternative to more expensive (or unavailable) private insurance. Since these types of insurance plans, however, are designed to protect the governments themselves, they do not meet the social benefit test of criteria three.

- Since most self-insurance or risk management funds are designed to insure the government itself, they fail to meet the fourth criteria regarding outside membership.

- Selected Federal Government programs that are generally labeled as insurance, including crop and farm mortgage insurance, investment guarantees, and home mortgage insurance (e.g., FHA and VA), are excluded by definition. Their exclusion is based primarily on the fact that they are funded through general appropriations on an annual basis, and not maintained as actuarially-based systems.

9.2 Types of Social Insurance Trust Systems

The Census Bureau recognizes four types of insurance trust systems, including an "other" category that encompasses three major Federal-only social insurance trusts.

9.2.1 Public Employee Retirement Systems

This category covers retirement systems sponsored by a recognized unit of government whose membership is comprised primarily of public employees compensated with public funds and which holds identifiable assets to finance retirement and associated benefits. They were described in Chapter 8.

9.2.2 Unemployment Compensation Systems

This category refers to the Federal-state cooperative program for unemployment insurance and also covers the District of Columbia, the only local government member. These data are obtained from the Employment and Training Administration of the U.S. Department of Labor, but can also be found in the CAFRs of most state governments. CAFR data are used especially where state fiscal years differ from the standard June 30 ending date.

The unemployment compensation system is unique.[2] The financial transactions of the Federal-state and railroad unemployment insurance systems are made through the Unemployment Trust Fund. All state and Federal unemployment "tax" receipts are deposited in the trust fund and invested in Federal Government securities. If necessary, the Federal general fund advances monies to the trust fund.

State governments and the District of Columbia pay for unemployment benefits from this trust fund.

[2]Based largely on information in the appendix to the Budget of the United States. References to state governments in this description also refer to the District of Columbia.

They also might receive repayable advances from the fund when their balances in the fund are insufficient to pay benefits.

State payroll "taxes" (i.e., *Contributions*, code Y01) finance all regular state government benefits. For Census Bureau purposes, these are classified as insurance trust contributions, rather than as taxes. The overriding factor in this classification decision is that these payroll "taxes" are dedicated for unemployment insurance use only, and the latter is classified as an insurance trust activity.

During high periods of unemployment, extended benefits are paid. These are financed equally between state and Federal payroll taxes. Benefits to former Federal civilian employees, Postal Service workers, and ex-service members are also paid by the state governments from the trust fund. However the respective state trust fund accounts are reimbursed by the various Federal agencies, on an as-needed basis.

9.2.3 Workers' Compensation Systems

This category includes state government-administered plans for compulsory accident and injury insurance of workers (public or private) through the accumulation of assets in order to provide disability or death benefits related to on-the-job injury or accident. Not all states have such a system, while some states actually operate more than one such system, or operate a mix of state-administered and privately-administered systems. As of 2005, the Census Bureau classified systems in forty-one states as workers' compensation insurance trust systems.

The Federal Government workers' compensation program is not classified as an insurance trust system because it is financed directly from agency operating funds rather than through an insurance fund financed by contributions (i.e., it is a pay-as-you-go plan).

9.2.4 Other Federal and State Social Insurance Trust Systems

This category includes any other Federal or state government-administered insurance trust system that meets the Bureau's definition of a social insurance trust.

Two examples are noted here:

- The state of Maryland administers the Maryland Automobile Insurance Fund. Legal owner/operators who cannot obtain insurance via private carriers are required to purchase insurance through this state-sponsored carrier.

- A state government insurance plan to protect homeowners against certain catastrophic risks, either because private insurance is unavailable or unaffordable, would meet the social criteria test and would be classified as an insurance trust as long as it met the other four criteria. The Citizens Property Insurance Corporation in Florida is an example.

The number of such systems and the states that sponsor them change regularly.

For the Federal Government, this category includes social insurance programs unique to the U.S. Government, including the biggest insurance trust:[3]

[3]The Federal Government also administers its own public employee retirement systems and is a member of the Federal-state cooperative unemployment compensation program.

- The Social Security and Medicare programs, also referred to as OASDHI (Old-Age Survivors', Disability, and Health Insurance program). It actually consists of multiple insurance funds, including the Old-Age and Survivors Insurance Trust Fund (OASI), Disability Insurance Trust Fund (DI), Hospital Insurance Trust Fund (HI), and Supplementary Medical Insurance Trust Fund (SMI), The last two funds constitute the Federal Medicare program.

- Veterans' life insurance system which provides low-cost life insurance to eligible veterans of the armed services.

- Railroad retirement system which provides retirement-survivor benefits to railroad workers and their families. The unemployment portion of this system is included with the unemployment compensation data.

Many Federal-only insurance programs are excluded from insurance trust data, by definition, and reported in the general government sector. These include Federal insurance programs such as crop and farm mortgage insurance, investment guarantees, home mortgage insurance (e.g., FHA, TVA), and the like. The rationale for their classification in the general sector, as opposed to the insurance trust sector, lies in the fact that these do not meet the second criterion in the definition of social insurance trust systems. Specifically, these programs are funded by general, annual appropriations of the Federal Government and not by a dedicated revenue source (contributions or premiums imposed on its members and/or member employers).

9.3 Regular Finance Statistics and Exhibit Finance Statistics – Explanations

The Census Bureau classification system contains two types of statistics on other social insurance trust systems. First, they are reported with the *regular statistics* for finance data about governments. Second, since insurance trust systems involve major financial activities that fall outside the scope of the regular classification system, the Census Bureau uses additional *exhibit statistics.*

Regular Census Bureau finance statistics provide only selected (partial) information on insurance trusts. The critical perspective in the regular statistics program is to present the financial activity for the government as a whole. Regular statistics covering government financial activity for other social insurance trust systems include the revenue statistics described in Chapter 4, the expenditure statistics described in Chapter 5, and the cash and security holding statistics described in Chapter 7. There are no debt statistics associated with other social insurance trust systems directly, since they are not authorized to issue debt or otherwise borrow money. Any such borrowing always is the responsibility of the parent government administering the system. See Section 9.6.1 for additional information.

Exhibit statistics are used to enhance the regular statistics in two ways. First, they add additional detail to the regular statistics, showing further breakdowns of financial activity not needed for purpose of the regular finance statistics. Secondly, the exhibit statistics are designed to include financial transactions that occur between the insurance trust system and its parent government. Such transactions are excluded from regular statistics, by definition.

9.3.1 Exhibit Statistics – Additional Detail

In the case of public employee retirement systems, the additional detail added to the regular statistics consisted primarily of statistics on cash and security holdings. This is not so important a factor for other social insurance trust systems.

9.3.2 Exhibit Statistics – Enterprise Perspective

As described in Chapter 2, the basis of reporting for Census Bureau statistics is the government entity. The definitions are such that many financial activities of dependent agencies, or financial transactions between the parent government and its dependent public employee retirement system, are excluded from basic measures. Exhibit statistics enable the Census Bureau to treat selected government activities independently of the parent government and give users an important perspective on financial activities of government-related enterprises. For other social insurance trust systems, the additional detail covered by exhibit statistics primarily covers internal transactions between the parent government and the system. These transactions "balance" out the flows of funds between receipts and expenses when users view the social insurance trust systems as individual entities.

9.3.3 Other Federal and State Social Insurance Trust Systems – Exhibit Codes

As explained in Chapter 8, statistics on public employee retirement systems have more exhibit codes than any other insurance trust category, reflecting the greater complexity of their finances. Some of these codes represent additional detail for a regular finance code (e.g., *Cash and Short-Term Investment*, code X21, is divided into three exhibit categories). Other codes are used solely for data collection and are never published (although they do appear in the special retirement data file).

The only exhibit code for unemployment compensation systems pertains to the Federal Government and reflects the fact that all its investments are in Federal securities whose interest payments represent interfund transfers. Code Y99 represents interest on U.S. securities held by the Federal Government.

Exhibit codes for workers' compensation system are limited to one each for revenue and expenditure. *Contributions to Own System*, code Y10, represents a state government's own contributions to its own workers' compensation system. *Administrative Expenses*, code Y15, represents administration, investment fees, losses on sale of investments, and other costs not representing benefits or withdrawals.

Similarly, exhibit codes for other state government social insurance trust systems are limited to one each for revenue and expenditure. *Contributions to Own System*, code Y50, again represents a state's contributions to its own system and *Administrative Expenses*, code Y54, represents administration, investment fees, losses on sale of investments, and other costs not representing benefits or withdrawals.

In addition, each of the three Federal-only social insurance trust systems has an exhibit code reflecting the fact that all its investments are in Federal securities whose interest payments represent interfund transfers. These are *Interest on Investments in U.S. Securities*, codes Y22, Y32, and Y42.

Table 9.1 contains the complete listing of all codes related to social insurance trust finances (exclusive of public employee retirement systems described in Chapter 8) including the exhibit codes.

9.4 Measurement Issues for Social Insurance Trust System Statistics

The following are explanations of how financial transactions of social insurance trust systems (other than for public employee retirement) are measured for statistical purposes.

9.4.1 Measurement Issues: Timing

As with other Census Bureau statistics on government finances, the fiscal year is the basis of measurement for social insurance trust statistics. The same rules described in Section 3.2 for governments apply to social insurance trust systems. Most, but not all, social insurance trust systems have the same fiscal year as their parent government. Where their fiscal year differs from that of the parent government, the Census Bureau uses the 12-month fiscal year of the social insurance trust system as the basis for measuring finances. For this reason, Census Bureau statistics on social insurance trust system finances might differ from financial statistics that appear in the parent government records, such as the CAFR. The following considerations are applicable to measurement during the fiscal year:

- Receipts and expenses are measured over the course of the fiscal year and represent total amounts received or expended for that year.

- Cash and security holdings are measured as of the end of the fiscal year.

9.4.2 Measurement Issues: Aggregation and Tabulation

The Census Bureau uses three different methods for aggregating and tabulating statistics involving social insurance trust systems. First, the regular statistics only are incorporated into the aggregate statistics used to measure the finances of the parent government. Hence selected "receipts" statistics are aggregated into the parent government's revenue data, and selected "expenses" statistics are aggregated into the parent government's expenditure statistics. In turn, such statistics are tabulated in totals by type of government, by state, by level of government, and so forth. For example, national totals of social insurance trust revenue, as used in Census Bureau publications on government finance, include only the regular statistics identified in this chapter.

Exhibit statistics for individual social insurance trust systems complement the regular statistics. When combined, regular and exhibit statistics are aggregated for individual insurance trust systems. These two statistical types represent a complete financial picture of the insurance trust system, without regard for the parent government.

9.4.3 Measurement Issues: Valuation

As discussed in Section 3.12, the Census Bureau program is first and foremost statistical in nature and is not intended to reflect the same types of measures developed for purely accounting purposes. Nevertheless, the Census Bureau uses standard accounting records to develop its government finance statistics, so accounting definitions and concepts must be taken into account in the Census Bureau statistical program.

For other social insurance trust systems, all receipts and expenses are measured only in current dollars. Each represents the total of financial transactions that occurred during the fiscal year.

Measurement is handled differently for statistics on cash and security holdings, however. Such holdings are measured at market value as of the close of each system's fiscal year.

9.5 Federal and State Social Insurance Trust System Finances – Basic Concepts

The following sections explain the concepts pertinent to receipts, expenses, and cash and security holdings,

as well as special treatment of selected financial activities of other social insurance trust systems. These essentially repeat information explained in Chapter 8 for public employee retirement systems.

The principle differences between the regular statistics and the exhibit statistics can be summarized as follows:

1. Regular finance insurance trust revenue excludes (as an intragovernmental transfer) contributions and other payments from the administering government. Insurance trust revenue statistics, on the other hand, include these sources of funds.

2. Intergovernmental transfers, taxes levied specifically to support insurance trusts, or other sources of general revenue to finance insurance trusts activities are classified as general revenue, not as insurance trust revenue. For Census Bureau purposes, the state unemployment payroll "taxes" are outside the scope of the general sector, and are classified as insurance trust contributions.

3. The cost of administering an insurance trust system is classified as a general expenditure of the administering government. It is recorded also under a special exhibit code for displaying insurance trust statistics.

Both regular and insurance trust exhibit statistics on revenue and expenditure exclude the purchase and sale of investments. Primarily applicable to public employee retirement systems, this financial activity was described previously in Chapter 8.

9.5.1 Social Insurance Trust Systems: Revenue and Receipts

As described in Chapter 4, insurance trust revenue comprises only social insurance contributions and net earnings on investments. Transfers or contributions from other funds of the same government are not classified as insurance trust revenue for purposes of regular statistics on government finances. Insurance trust revenue excludes (as interfund transfers) contributions from the government which administers the system, whether they are paid on behalf of its employees covered by the plan or for supplemental support.[4] Also excluded from insurance trust revenue and classified as general revenue are tax receipts credited directly to insurance trust funds and intergovernmental aid, such as grants and shared taxes for support of insurance trust activities.[5] Excluded entirely as revenue (insurance trust or general) are proceeds from borrowing for insurance trust purposes.

However, Census Bureau statistics on other social insurance trust system receipts are broader in concept and include both the regular and exhibit statistics explained earlier. "Receipts" include the following:

- Amounts derived from contributions (mandatory or voluntary).

[4]Such contributions by the administering government, however, are recorded under special exhibit codes and included in insurance trust revenue when data are published solely for insurance trust systems.

[5]On the other hand, funds from other governments which represent the latter's employer share of contributions to an insurance trust system to which their employees are members are classified as insurance trust revenue.

- Net earnings on investments set aside to provide income for insurance trusts.[6] Note that losses on investments are classified as expenditures for other social insurance trust systems.

- Transfers or contributions from the parent government that administers the system (either as employer contributions or for general financial support).

- Interest earnings on their own or other governments' securities held for investment are included because of the difficulty in identifying such transactions.

The payment by one government, for general financial support, to another government's insurance trust is treated as a current operation expenditure of the paying government (for the function involved) and as an insurance trust revenue of the receiving government, not as intergovernmental transactions. The purpose of this treatment is to avoid an imbalance between intergovernmental revenue and expenditure. Since intergovernmental revenue and expenditure are "two sides of the same coin," in theory the two should always equal. Also, contributions for an insurance trust system are insurance trust revenue so long as they come from outside the administering government. To avoid the imbalance between intergovernmental revenue and expenditure that would result if the payment of the contribution were treated as intergovernmental expenditure and the receipt were treated as insurance trust revenue, neither of these transactions is treated as intergovernmental.

9.5.2 Social Insurance Trust Systems: Expenditure and Expenses

As described in Chapter 4, insurance trust expenditure statistics are limited to the perspective of the parent government and its dependent agencies in total. As such, regular expenditure statistics consist of social insurance payments to beneficiaries and withdrawals of insurance contributions. In effect, it only includes amounts paid to beneficiaries or members of the systems. Under this concept, the following are excluded from regular statistics on expenditure:

- Costs of administering the system, including management of investments (classified as a general government expenditure).

- Contributions to a government's own system (an intragovernmental transfer).

- Contributions or other payments to systems administered by another government (classified as current operation expenditure when such amounts represent employer contributions on behalf of the contributing government's employees and as agency transaction when such amounts represent sums withheld from employee salaries and wages).

- "Pay-as-you-go" plans.

Census Bureau statistics on other social insurance trust system expenses are broader in concept and include both the regular and exhibit statistics explained earlier.

---

[6]The substantial amount of interest paid by the U.S. Treasury to Federal insurance trust systems (which have all their reserves invested in Federal securities) is excluded from Federal insurance trust revenue since it is an intragovernmental transfer. The principle of eliminating these interfund transactions, however, is not followed in the case of interest paid by a state or local government on any of its own securities held as investments by insurance trust funds it administers – mainly because of the difficulty of identifying such transactions.

1. Benefits and Withdrawals: Cash payments to, or on behalf of, participants in the system or members thereof, depending on the systems coverage. For unemployment compensation systems, expenses reflect monthly payments while unemployed. For workers' compensation systems, these could reflect payments while unable to work. For other state systems, benefits could reflect compensation for losses covered by the system, such as from Maryland's uninsured motorists fund.

2. Other Payments: Expenses include the exhibit codes for administering the system or managing its assets (also reported at *Financial Administration*, code *23, for regular expenditure statistics on government finances) and other costs or payments not representing benefits or withdrawals (except purchase of investments).

9.5.3 Social Insurance Trust System Cash and Security Holdings

Unlike statistics on receipts and expenses, there are very few differences between regular statistics and exhibit statistics on cash and security holdings of other social insurance trust systems. The concepts and definitions in Chapter 7 apply almost equally to both sets of statistics, as do the definitions for each type of asset included in the Census Bureau program. The only difference involves the insurance trust systems of the Federal Government. The Census Bureau does not include cash and security holdings of the Federal systems.

9.6 Special Topics

Insurance trust finances are among the most complicated in the field, partly as a result of the large amount of intragovernmental transactions. This section discusses some of the more complex topics and describes how they are treated for Census Bureau statistics.

9.6.1 Special Topics: Classifying Debt for Unemployment Compensation Funds

In rare cases, a government may issue debt to bolster its unemployment compensation (UC) system. Typically, it raises the contribution rate it levies on employers to cover debt service. This situation is classified in the following manner:

- Revenue, *Contributions*, Code Y01: The government's unemployment compensation revenue consists of two parts: the "normal" payment to the UC fund and the amount needed to cover debt service (principal and interest). The former is treated as an UC revenue, code Y01; the latter is classified under *Miscellaneous General Revenue* , code U99.

- Revenue, *Federal Advances and Contributions*, Code Y04: One possible reason for such debt is to repay the Federal Government for advancing funds when the government's UC account was insufficient to pay benefits. As described in Chapter 7, repayment of Federal advances is **deducted** from code Y04, leading to the possibility of a negative value for this revenue code. Note that, in a situation requiring a government to issue debt to repay the advances, it is possible that the repayment is so large that not only is code Y04 negative, but so is total unemployment compensation revenue.

- Debt Retired, Issued, and Outstanding: This debt is classified in the "unspecified public purpose" category. Note that the transfer of the proceeds to the insurance trust fund would

be an intragovernmental transfer. (Expenditures from it will eventually be reported as an insurance trust outlay.)

- Interest on Debt: Interest on the debt outstanding is reported under *Interest on General Debt*, code I89.

9.6.2 Special Topics: Loans to Parent Government by Social Insurance Trust Systems

Because of their large amounts of cash and security holdings, insurance trust systems sometimes help out their parent government during times of fiscal distress. How these financial transactions are treated depends on the situation involved.

The insurance trust system may help out its parent government by purchasing its regular debt instruments; these are treated the same as other debt and investment transactions. That is:

- It is not reported as either revenue of the parent government or as expenditure of the insurance trust.

- It is reported under debt statistics of the parent government (debt issued and outstanding) and under cash and security holdings data of the insurance trust.

- Interest payments on the debt are reported as interest expenditure of the parent government (*Interest on Debt,* code I89) and as earnings on investment of the insurance trust. No effort is made to exclude this transaction as an interfund transfer.

The insurance trust system may loan money directly to the parent government with a special note being involved. In these situations, the loan is not treated as debt of the parent government nor as a cash and security holding of the parent government. It is not reported as an insurance trust expenditure. The purpose of this treatment is to avoid duplication.

9.6.3 Special Topics: "Internal" Investment Transactions

Insurance trust systems often hold for investment purposes securities issued by their own government. Since these types of transactions are generally not identified in a government's records, the interest paid by the government for such securities is reported as interest expenditure (e.g., at *Interest on Debt*, code I89) and the interest received by the insurance trust system is reported as earnings on investments. The exception is the interest earnings of Federal Government insurance trusts since all their investments are in U.S. securities. These are treated exclusively as exhibit code items in Census Bureau statistics on Federal Government finance.

9.7 Description Pages for Federal and State Social Insurance Trust System Statistics

The following pages contain all of the codes used in Census Bureau for other Federal and state social insurance trust systems. These include the regular and exhibit statistic codes that comprise all financial categories (receipts, expenses, and cash and security holdings). The presentation is similar to that used in Chapters 4 through 7 for regular finance statistics. The codes are presented in order (receipts, expenses, cash and security holdings). For all statistics, each code is defined and, as appropriate, inclusions and exclusions are listed. Examples are cited if necessary.

Each code is clearly identified as being part of the regular finance statistics or as being an exhibit statistic. This is useful information for understanding how these statistics are incorporated into the Census Bureau's regular statistics on government finances. Codes that are sub-categories of a larger aggregate category follow in order.

RECEIPTS

Contributions from employees and governments (as employers) and interest earnings on deposits in U.S. Treasury for the cooperative Federal-state unemployment compensation insurance system and for Federal railroad unemployment compensation. This category applies only to the Federal Government, state governments, and the District of Columbia.

Regular Code Y01 Contributions

Definition: Payroll levies (or "taxes") collected from private and public employers (and in some cases employees) to provide for unemployment compensation benefits.

Excludes: Borrowing of funds (nonrevenues) and contributions (including special payroll levies) to pay debt service on loans issued to cover fund deficits (report at *Miscellaneous General Revenue, NEC*, code U99).

Regular Code Y02 Interest Credited by U.S. Government

Definition: Interest earned on deposits held in the Unemployment Trust Fund of the U.S. Treasury and credited to individual state governments and to the District of Columbia.

Regular Code Y04 Federal Advances and Contributions

Definition: Net amounts credited to the accounts of the individual state governments and District of Columbia during periods where contributions and interest are insufficient to pay benefits.

Special Consideration:
Report repayment of advances as deductions to this code, not as unemployment compensation expenditures.

Exhibit Code Y99 Interest on U.S. Securities Held by Federal Trusts

Definition: Interest on Federal securities held in trust by Federal portion of the Unemployment Compensations trust funds

Special Consideration:
This code applies only to statistics of the Federal Government.

EXPENSES

Distribution of cash benefits to eligible persons under the cooperative Federal-state unemployment compensation insurance programs.

Regular Code Y05 Regular Benefits

Definition: Basic payments to individuals temporarily out of work and seeking employment (funded by state payroll taxes) and to former Federal civilian and military employees (funded by Federal agencies that employed them).

Excludes: A number of important aspects of the Unemployment Compensation Funds are excluded from these codes, as noted below.

- Administration of unemployment insurance programs (report at *Social Insurance Administration*, code *22 for regular statistics).
- Employment services provided to the unemployed, such as job training, placement, and guidance (use code *22);
- Government payments as employer contributions on behalf of its employees to unemployment insurance system of another government (report as current operation of function involved) or to one it administers (interfund transfer).
- Distinctive sickness or disability insurance programs carried out in conjunction with unemployment programs (generally, report at *Other Insurance Trusts*, code Y5*).

Special Consideration:
This code applies to the Federal Government, to state governments, and to Washington, DC only. Former Federal employees, including military, receive benefits from their respective states, whose trust fund accounts are funded in part by employing agencies of former Federal/military workers.

Regular Code Y06 Extended and Special Benefits

Definition: Payments for extended unemployment benefits in states with high rates of unemployment (funded equally by Federal and state payroll taxes)

Includes: Benefits under special programs, such as trade adjustment assistance and disaster assistance (funded by Federal appropriations), and benefits to special groups of former workers, such as railroad employees (funded by Federal tax on railroad payrolls).

Special Consideration:
This code applies to state governments and to Washington, DC only. It does no apply to Federal Government statistics. All Federal payments are reported at *Regular Benefit Payments*, code Y05, as funded by employing agencies of former Federal/military workers.

CASH AND SECURITY HOLDINGS

The Census Bureau uses two "By Type" classification categories for cash and securities of the state-administered Unemployment Compensation Systems. Both of the categories shown below are considered to be "cash and deposits." There are no securities, per se, for the holdings of the state Unemployment Compensation System accounts.

Data for this category are obtained primarily from the Employment and Training Administration of the U.S. Department of Labor. However, as states have become more inclusive in their reporting as a result of GASB guidelines, Census Bureau analysts can make use of individual state financial statements where to obtain data, where the states include the Unemployment Compensation Systems activity in such accounting records.

Regular Code Y07 Trust Fund Account in U.S. Treasury

Definition: Balance held in each state's basic trust fund account in the U.S. Treasury.

Special Consideration:
This code does not apply to statistics on the Federal Government.

Regular Code Y08 Other (Clearing and Benefit Accounts)

Definition: Balance held in each state's special trust fund account in the U.S. Treasury.

Special Considerations:

1. Y08 can be a positive or a negative number. Negative balances occur during periods of higher than normal unemployment, where the state governments "borrow" from the Federal Government to cover extended or special benefit unemployment benefits.
2. Pre-1988 versions of the *Classification Manual* showed code Y09 for negative balances and limited Y08 to positive balances. Code Y08 now represents the net balance, plus or minus.
3. This code does not apply to statistics on the Federal Government.

Applicability of Codes, By Level and Type of Government

Category	Federal	State	Local*
Receipts	Y01, Y99	Y01, Y02, Y04	No - All Types
Expenses	Y05	Y05, Y06	No - All Types
Assets	No	Y07, Y08	No - All Types

* Statistics are applicable to Washington, DC, even though it is classified as a local (municipal) government for Census Bureau purposes.

RECEIPTS

Contributions and interest earnings of state government-operated insurance trust funds to provide accident, disability, and death benefits for private and public employees. This category applies solely to state governments. These codes apply only to state government systems. Both the Federal Government and the government of Washington, DC operate "pay-as-you-go" systems that do not meet the Census Bureau's definition.

Exhibit Code Y10 Contributions to Own System

Definition: Contributions from a state government to its own workers' compensation system.

Regular Code Y11 Contributions

Definition: Premiums, assessments, payroll "taxes" or other contributions collected from employers and employees to provide for workers' compensation benefits, including contributions from local governments as employers on behalf of their own employees. Also includes amounts received from employees of either state or local governments.

Excludes: Contributions from state government administering insurance trust system (use exhibit *Contributions to Own System*, code Y10); dividends or return of excess premiums (report as a deduction from revenues); penalties and fines (use *Fines and Forfeits,* code U30); charges for such services as copying documents (report at *All Other General Charges*, code A89); recovery of benefits and other miscellaneous revenues (use *Miscellaneous General Revenue, NEC*, code U99); and Federal grants (report at appropriate *Intergovernmental Revenue from the Federal Government* code).

Regular Code Y12 Earnings on Investments

Definition: Interest earnings on investment securities, deposits, and other interest-bearing accounts, including accrued interest on investment securities sold; dividends; recorded profits on investment transactions (minus any recorded losses); rentals; and other earnings on investments.

Special Consideration:
Also report losses on investments at exhibit *Administrative Expenses*, code Y15. Previous manuals separated earnings on investments between interest income (code Y12) and other types of investment earnings (Y13). However, in practice this distinction was not made on the survey schedules themselves. Consequently, these two codes were consolidated effective with fiscal year 1990 data.

EXPENSES

Distribution of cash benefits to eligible persons under state-administered plans for compulsory accident and injury insurance of workers.

Regular Code Y14 Benefit Payments

Definition: Claims paid directly to, or on behalf of, injured beneficiaries for compensation of wages lost, medical care, rehabilitation, funeral expenses, and other eligible benefits. Includes payments made from regular workers' compensation funds as well as from subsequent injury funds, second injury funds, and the like.

Exhibit Code Y15 Administrative Expenses

Definition: Administration, investment fees, losses on the sale of investments (also deducted at Y12), and other costs not representing either benefits or withdrawals.

Includes: Adjudication of claims (also report at *Financial Administration*, code *23); recorded losses on investments (deduct from *Investment Earnings*, code Y12); other costs or payments not representing benefits (except purchase of investments).

CASH AND SECURITY HOLDINGS

Regular Code Y21 Cash and Securities for Workers' Compensation Systems

Definition: Cash and security holdings of state government compulsory accident and injury insurance systems for workers' compensation.

Includes: Various cash and security assets as delineated in Table 7.1.

Special Consideration:
Prior to fiscal year 1988 data, this code was divided further into cash and deposits (code Y21), Federal treasury securities (code Y30), Federal agency securities (code Y33), state and local government securities (code Y35), and nongovernmental securities (code Y44).

Applicability of Codes, By Level and Type of Government

Category	Federal *	State	Local *
Receipts	No - all codes	Y10, Y11, Y12	No - All Types
Expenses	No - all codes	Y14, Y15	No - All Types
Assets	No - all codes	Y21	No - All Types

* The Workers' Compensation activity for both the Federal Government and the government of Washington, DC are classified as "pay-as-you-go" systems for purposes of Census Bureau statistics. These codes thus do not apply to either government.

OTHER STATE SOCIAL INSURANCE TRUST SYSTEMS

Data on these systems are collected by a separate mail canvass survey of state insurance trust systems. There is no equivalent classification categories for Census Bureau statistics on public employment. State employees for administering these system are classified at *Financial Administration*, code *23.

The following categories and codes apply only to state governments. See Table 9.1.

RECEIPTS

Contributions and earnings on investments for state-administered social insurance systems for health benefits, disability benefits, and other purposes. This category applies solely to state governments.

Exhibit Code Y50 Contributions to Own System

Definition: State government contribution to its own "other social insurance trust system."

Regular Code Y51 Contributions

Definition: Premiums, assessments, or contributions collected from members to provide for benefits, including contributions from local governments as employers on behalf of their own employees. Also includes amounts received from employees of either state or local governments.

Excludes: Contributions from state government administering insurance trust system (use exhibit *Contributions to Own System*, code Y50).

Regular Code Y52 Earnings on Investments

Definition: Interest earnings on investment securities, deposits, and other interest-bearing accounts, including accrued interest on investment securities sold; dividends; recorded profits on investment transactions (minus any recorded losses); rentals; and other earnings on investments.

Special Consideration:
Also report losses on investments at exhibit *Other Payments*, code Y54. Previous manuals separated earnings on investments between interest income (code Y52) and other types of investment earnings (Y55). However, in practice this distinction was not made on the survey schedules themselves. Consequently, these two codes were consolidated effective with fiscal year 1990 data.

EXPENSES

Regular Code Y53 Benefits and Withdrawals

Definition: Distribution of cash benefits to eligible persons under state-administered plans for compulsory or voluntary social insurance programs not elsewhere classified, or withdrawals from such systems, as allowed by law.

OTHER STATE SOCIAL INSURANCE TRUST SYSTEMS

EXPENSES - continued

Regular Code Y53 **Benefits and Withdrawals -** continued

Includes: Benefits include claims paid directly to, or on behalf of, beneficiaries for lost income, medical care, disaster reimbursement, and other eligible expenses.

Excludes: Payments on group health, life, or other fringe benefit insurance plans for its employees (report at function involved or at *Other and Unallocable*, code *89). Exclude administrative costs from benefits and report them both at code Y54 and *Financial Administration*, code *23.

Exhibit Code Y54 **Other Payments**

Definition: Expenses for administration of insurance plans (also report at *Financial Administration*, regular code *23)

Includes: Administration, investment fees, recorded losses on investments (also deduct from *Investment Earnings*, code Y52) and other costs or payments not representing benefits (except purchase of investments).

Excludes:

- Local government payments to state systems (report as local current operation expenditure of function involved).
- State government payments to own insurance trusts (interfund transfer).
- Purchase of investments.
- Self-insurance, risk management, insurance against fire or other hazards, and other types of non-social insurance (generally, report at *Other and Unallocable*, code *89).

CASH AND SECURITY HOLDINGS

Regular Code Y61 **Total Cash and Security Holdings, Other State Social Insurance Trust Systems**

Definition: Cash and security holdings of state government insurance trust systems other than employee retirement, unemployment compensation, and workers' compensation.

Includes: Various cash and security assets as delineated in Table 7.1.

Examples: Maryland Automobile Insurance Fund, a dependent agency of the state of Maryland.

Special Considerations:

Prior to fiscal year 1988 data, this code was further divided into cash and deposits (code Y61), Federal treasury securities (code Y70), Federal agency securities (code Y73), state and local government securities (code Y75), and nongovernmental securities (code Y84).

OTHER STATE SOCIAL INSURANCE TRUST SYSTEMS

Applicability of Codes, By Level and Type of Government

Category	Federal	State	Local
Receipts	No - all codes	Y50, Y51, Y52	No - All Types
Expenses	No - all codes	Y53, Y54	No - All Types
Assets	No - all codes	Y61	No - All Types

OTHER SOCIAL INSURANCE TRUST SYSTEMS: FEDERAL ONLY

SOCIAL SECURITY AND MEDICARE RECEIPTS

Regular Code Y20 Contributions

Definition: Contributions (employee, employer, and self-employed payroll taxes) to provide for benefits under the Federal Old-Age, Survivors, and Health and Disability Insurance Programs, commonly referred to as Social Security and Medicare.

Exhibit Code Y22 Interest on Investments in U.S. Securities, Social Security and Medicare

Definition: Interest earnings on Social Security and Medicare Funds, invested solely in special U.S. Treasury Securities.

Special Consideration:
These receipts represent an internal Federal transfer and thus are excluded from regular finance statistics.

SOCIAL SECURITY AND MEDICARE EXPENSES

Regular Code Y25 Benefits and Withdrawals

Definition: Distribution of cash benefits to eligible persons under Federal-administered retirement, disability, hospital, and health social insurance programs.

Includes: Comprises cash benefits: to retirees or their dependents and survivors from the Federal Old-Age and Survivors' Insurance Trust Fund (OASI); to disabled workers or their dependents from the Federal Disability Insurance Trust Fund (DI); to elderly or disabled for hospital, nursing home, and other related care from the Federal Hospital Insurance Trust Fund (HI); and to such persons for physician services, outpatient care, renal dialysis, and other health care from the Federal Supplementary Medical Insurance Trust Fund (SMI). The OASI and DI funds are administered by the Department of Health and Human Services' (HHS) Social Security Administration (SSA) wile the HI and SMI funds are operated by HHS' Health Care Financing Administration (HCFA).

Excludes: Governmental contributions as employer for Social Security and Medicare on behalf of its employees (report as current operation expenditure of function involved); Federal transfers to these Social Security or Health Care trust funds; transfers from Social Security Trust Funds to the railroad retirement accounts; interest payments on inter-trust borrowings (the preceding three are nonexpenditures); administration of these insurance trust funds (report at *Social Insurance Administration*, code *22); payments of HCFA for Medicaid program (report at *Federal Categorical Assistance Programs*, code *67); payments of SSA other than for the Social Security Trusts, such as SSI and AFDC (use code *67) and black lung benefits, child support enforcement, low income energy assistance, etc. (report at *Other Public Welfare*, code *79).

OTHER SOCIAL INSURANCE TRUST SYSTEMS: FEDERAL ONLY

SOCIAL SECURITY AND MEDICARE EXPENSES

Regular Code Y25 Benefits and Withdrawals - continued

Special Considerations:

1. This function applies soles to Federal Government expenditure
2. The OASI and DI trusts comprise what is commonly referred to as Social Security. The HI and SMI trusts comprise what is commonly referred to as Medicare, Parts A and Part B, respectively.
3. In contrast to public welfare programs funded by appropriations and classified as general expenditure, these social insurance programs all have separate trust funds with dedicated revenues (either payroll taxes or voluntary premiums).
4. There is no equivalent function code for Census Bureau statistics on public employment. Employees engaged in administering this activity are classified at *Social Insurance Administration*, code *22.

VETERANS' LIFE INSURANCE RECEIPTS

Regular Code Y31 Veterans' Life Insurance Contributions

Definition: Insurance premiums from veterans for purchase of annuities in life insurance programs administered by the U.S. Department of Veterans Affairs (formerly, U.S. Veterans Administration).

Excludes: Appropriations from general and special revenue funds (intragovernmental transfers).

Exhibit Code Y32 Interest on Investments in U.S. Securities, Veterans' Life Insurance

Definition: Interest earnings on Veterans' Life Insurance Funds, invested solely in special U.S. Treasury Securities.

Special Consideration:

These receipts represent an internal Federal transfer and thus are excluded from regular finance statistics.

VETERANS' LIFE INSURANCE EXPENSES

Regular Code Y34 Benefits and Withdrawals

Definition: Distribution of cash benefits and dividends to eligible veterans and their beneficiaries under Federal-administered veterans' life insurance programs.

VETERANS' LIFE INSURANCE EXPENSES - continued

Regular Code Y34 Benefits and Withdrawals - continued

Includes: Death claims to beneficiaries; cash values of surrendered policies; distribution of excess earnings and related interest (dividends); disability claims or income payments under veterans' life insurance policies; and matured endowments. Comprises activities of National Service Life Insurance Fund as well as the Veterans Special Life Insurance Fund, Veterans Reopened Insurance Fund, US Government Life Insurance Fund, and others administered by the U.S. Department of Veterans Affairs.

Excludes: Loans to policyholders borrowing against cash value of their policies; Federal Government contributions to these funds; purchase of securities (the preceding are nonexpenditures); Federal payments as employer contributions for group life insurance for its employees (report as current operation expenditure of function involved); administration of these life insurance programs (report at *Other Veterans Services*, code *85); and burial benefits not paid out of veterans' life insurance funds (use code *85).

RAILROAD RETIREMENT FUND RECEIPTS

Regular Code Y41 Contributions

Definition: Contributions (employee and employer) to provide for benefits under the Federal Railroad Retirement and Disability system.

Excludes: Contributions from Social Security trusts, tax subsidies, and any other Federal receipts representing interfund transfers; and contributions for Federal railroad unemployment compensation (use *Contributions*, code Y01).

Exhibit Code Y42 Interest on Investments in U.S. Securities, Railroad Retirement

Definition: Interest earnings on Railroad Retirement and related funds, invested solely in special U.S. Treasury Securities.

Special Consideration:
These receipts represent an internal Federal transfer and thus are excluded from regular finance statistics.

RAILROAD RETIREMENT FUND EXPENSES

Regular Code Y45 Benefits and Withdrawals

Definition: Distribution of cash annuities and benefits to eligible retirees and their survivors under Federal-administered rail industry pension programs.

RAILROAD RETIREMENT FUND EXPENSES - continued

Regular Code Y45 Benefits and Withdrawals - continued

Includes: Distribution of cash annuities and benefits to eligible retirees and their survivors under Federal-administered rail industry pension programs.

Excludes: Federal payments (transfers) to the railroad retirement accounts; Federal Old-Age and Survivors Insurance Trust Fund payments to the railroad retirement accounts; purchase of securities (the preceding are nonexpenditures); benefits from related Railroad Unemployment Insurance Fund (report at *Unemployment Compensation Benefits*, code Y05); and administration of railroad retirement program and railroad unemployment insurance fund (report at *Social Insurance Administration*, code *22).

Special Considerations:

1. This function applies soles to Federal Government expenditure; report any related employment data at *Social Insurance Administration*, code *22).

2. Unlike public employee retirement systems, this category covers private sector workers rather than public employees.

Applicability of Codes, By Level and Type of Government

Category	Federal	State	Local
Receipts	Y20, Y22, Y31, Y32, Y41, Y42	No - all codes	No - All Types
Expenses	Y25, Y34, Y45	No - all codes	No - All Types
Assets	None	No - all codes	No - All Types

9.8 Tables

There are two tables associated with this chapter. Table 9.1, Applicability of Other Social Insurance Trust System Codes, by Level and Type of Government, contains information on regular and exhibit finance codes, and details their applicability by level of government. Where codes apply, they are valid and the implication is that data could exist for that type of financial category or transaction. Table 9.2, Summary of Federal and State Social Insurance Trust System Codes, lists all the codes in the classification system that pertain to Federal and state social insurance trust systems (other than for public employee retirement) and indicates what type of statistics they represent (regular, exhibit or descriptive). There are no derived statistical codes for these Federal and state social insurance trust systems.

Table 9.1
Applicability of Other Social Insurance Trust System Codes,
by Level and Type of Government – page 1 of 2

Category	Code	Type	Federal	State	Local - All Types
**Unemployment Compensation Systems**					
Receipts:					
Contributions	Y01[1]	R	Valid	Valid	X[1]
Interest	Y02[1]	R	X	Valid	X[1]
Federal Advances and Contributions	Y04[1]	R	X	Valid	X[1]
Interest on Securities Held by Federal Trust Fund	Y99	E	Valid	X	X
Expenses:					
Regular Benefit Payments	Y05[1]	R	Valid	Valid	X[1]
Extended and Special Benefit Payments	Y06[1]	R	X	Valid	X[1]
Cash and Security Holdings:					
Trust Fund Account in U.S. Treasury	Y07[1]	R	X	Valid	X[1]
Other (Clearing and Benefit Accounts)	Y08[1]	R	X	Valid	X[1]
**Workers' Compensation Systems**					
Receipts:					
Contributions to Own System	Y10	E	X	Valid	X
Contributions	Y11	R	X	Valid	X
Earnings on Investments	Y12	R	X	Valid	X
Expenses:					
Benefit Payments	Y14	R	X	Valid	X
Administrative Expenses	Y15	E	X	Valid	X
Cash and Security Holdings:					
Total Cash and Security Holdings	Y21	R	X	Valid	X
**Other State Social Insurance Trust Systems**					
Receipts:					
Contributions to Own System	Y50	E	X	Valid	X
Contributions	Y51	R	X	Valid	X
Earnings on Investments	Y52	R	X	Valid	X
Expenses:					
Benefits and Withdrawals	Y53	R	X	Valid	X
Other Payments	Y54	E	X	Valid	X
Cash and Security Holdings:					
Total Cash and Security Holdings	Y61	R	X	Valid	X

Notes:
[1]These codes apply to Washington, DC, but to no other local governments, in addition to applying to state governments.

Table 9.1
Applicability of Other Social Insurance Trust System Codes,
by Level and Type of Government – page 2 of 2

Category	Code	Type	Federal	State	Local - All Types
**Federal Government Only: Social Security and Medicare**					
Receipts:					
Contributions	Y20	R	Valid	X	X
Interest on Investments in U.S. Securities	Y22	E	Valid	X	X
Expenses:					
Benefits and Withdrawals	Y25	R	Valid	X	X
**Federal Government Only: Veterans' Life Insurance**					
Receipts:					
Contributions	Y31	R	Valid	X	X
Interest on Investments in U.S. Securities	Y32	E	Valid	X	X
Expenses:					
Benefits and Withdrawals	Y34	R	Valid	X	X
**Federal Government Only: Railroad Retirement Funds**					
Receipts:					
Contributions	Y41	R	Valid	X	X
Interest on Investments in U.S. Securities	Y42	E	Valid	X	X
Expenses:					
Benefits and Withdrawals	Y45	R	Valid	X	X

Key:
X Not a valid code for the level or type of government indicated (there are no statistics).
Valid Applicable code for the level or type of government indicated (statistics are collected and reported).
Type Type of statistic (see Chapter 9.2).
R Regular
E Exhibit

**Table 9.2**<br>**Summary of Federal and State Social Insurance Trust System Codes –** page 1 of 2				
**Finance code**	**Brief description**	**Regular code**	**Exhibit code**	**Descriptive code**
	**Unemployment Compensation Systems**			
	Receipts and related categories:			
Y01	Contributions	Yes	-	-
Y02	Interest	Yes	-	-
Y04	Federal Advances and Contributions	Yes	-	-
Y99	Interest on Securities Held by Federal Trust Fund	-	Yes	-
	Expenses and related categories:			
Y05	Regular Benefit Payments	Yes	-	-
Y06	Extended and Special Benefit Payments	Yes	-	-
	Cash and Security Holdings:			
Y07	Trust Fund Account in U.S. Treasury	Yes	-	-
Y08	Other (Clearing and Benefit Accounts)	Yes	-	-
	**Workers' Compensation Systems**			
	Receipts and related categories:			
Y10	Contributions to Own System	-	Yes	-
Y11	Contributions	Yes	-	-
Y12	Earnings on Investments	Yes	-	-
	Expenses and related categories:			
Y14	Benefit Payments	-	Yes	-
Y15	Administrative Expenses	-	Yes	-
	Cash and Security Holdings:			
Y21	Total Cash and Security Holdings	Yes	-	-
	**Other State Social Insurance Trust Systems**			
	Receipts and related categories:			
Y50	Contributions to Own System	-	Yes	-
Y51	Contributions	Yes	-	-
Y52	Earnings on Investments	Yes	-	-
	Expenses and related categories:			
Y53	Benefits and Withdrawals	Yes	-	-
Y54	Other Payments	-	Yes	-
	Cash and Security Holdings:			
Y61	Total Cash and Security Holdings	Yes	-	-

Table 9.2<br>**Summary of Federal and State Social Insurance Trust System Codes –** page 2 of 2				
**Finance code**	**Brief description**	**Regular code**	**Exhibit code**	**Descriptive code**
	**Federal Government Only: Social Security and Medicare**			
	Receipts and related categories:			
Y20	Contributions	Yes	-	-
Y22	Interest on Investments in U.S. Securities	-	Yes	-
	Expenses and related categories:			
Y25	Benefits and Withdrawals	Yes	-	-
	**Federal Government Only: Veterans' Life Insurance**			
	Receipts and related categories:			
Y31	Contributions	Yes	-	-
Y32	Interest on Investments in U.S. Securities	-	Yes	-
	Expenses and related categories:			
Y34	Benefits and Withdrawals	Yes	-	-
	**Federal Government Only: Railroad Retirement Funds**			
	Receipts and related categories:			
Y41	Contributions	Yes	-	-
Y42	Interest on Investments in U.S. Securities	-	Yes	-
	Expenses and related categories:			
Y45	Benefits and Withdrawals	Yes	-	-

Key:
"Yes Indicates applicable for that type of statistic.
- Indicates not applicable for that type of statistic.

There are no descriptive statistics for these social insurance trust systems.
There are no cash and security holdings for Federal Government social insurance trust systems.

Chapter 10. Liquor Stores and Lotteries – All Statistics

Introduction

This chapter contains the complete classification categories and their codes for reporting statistics on government liquor store and lottery system operations. It includes three of the four types of statistics used by the Census Bureau for classifying government finance activity, as described in Chapter 2. *Regular finance statistics* apply to selected liquor stores and lottery activity and also are incorporated into the Census Bureau's finance statistics for the parent government that operates the liquor store or lottery. *Exhibit finance statistics* show additional detail pertaining to these commercial type activities of governments. They give a more complete picture of the liquor and lottery system finances, treating them as if they were stand-alone enterprise activities of the government. There are no *descriptive statistics* for liquor store and lottery finances in the Census Bureau classification system, as there are for social insurance trust systems (public employee retirement systems). There are, however, additional *derived statistics* for liquor store financial activity that exist in the Census Bureau classification system. These are described later in Section 10.4.

The 2005 redesign of the Census Bureau's government finance statistics program had some impact on the liquor store and lottery classifications. Specifically, regular codes for both liquor and lottery regular finance statistics were made valid for all local general purpose governments. Prior to 2005, no regular lottery statistics could be used for local governments, with the exception of Washington, DC. Regular liquor statistics applied only to large local governments, a handful of which administered liquor store operations.

Even with the 2005 redesign, however, exhibit and derived statistics for liquor store operations are limited to state governments only. Also, exhibit statistics for lotteries are limited to state governments and Washington, DC only.

The decision to retain these limitations was based on several factors. First, as of 2005 there were 430 locally-administered liquor store operations (and none for Washington, DC). Secondly, the Census Bureau's annual government finance sample survey is designed to yield statistical estimates for regular statistics only.[1] Since exhibit and derived statistics cause additional data collection and processing burdens, but would not be tabulated for local governments (the sample-based portion of the Census Bureau's annual survey of government finances), it was decided not to burden the small number of local governments involved for additional information on the survey questionnaires. Third, prior to the 2005 redesign, liquor store and lottery exhibits applied only to state governments and Washington, DC, so the post 2005 classification system was left unchanged to maintain historical continuity.

10.1 Liquor Stores Statistics – Overview

Liquor store statistics cover the liquor store systems owned and operated by state governments, as well as local governments in a few states. As of 2005, the Census Bureau has identified seventeen state governments that have retail and/or wholesale liquor store operations. Also, there are selected local governments in seven states that have retail liquor store operations.

[1]An exception exists for the annual survey of public employee retirement systems, which is sample based but does yield estimates of many exhibit statistics. However, none of the latter are used in the broader, annual survey of government finances covering parent governments and all their dependent agencies.

10.2 Liquor Stores Definition

As defined for Census Bureau purposes, liquor store financial activity covers only the operation and maintenance of government operated retail or wholesale liquor monopolies. If a government does not operate any such facilities, then it has no statistics for liquor store activity. Even when a government undertakes law enforcement activity related to the oversight of liquor stores, it does not constitute liquor store activity for Census Bureau purposes. The existence of government operated liquor store retail or wholesale facility is mandatory before any regular, exhibit, or derived statistics for liquor stores can exist in the Census Bureau classification system.

Such facilities could be referred to as alcoholic beverage monopolies, Alcoholic Beverage Control (ABC) stores, state liquor stores, or even liquor control boards, if the latter actually oversee government (not private) liquor operations. Such operations need not be limited to retail activity, but could include governmental control of wholesale liquor operations.

When such operations exist, the Census Bureau collects regular, exhibit, and derived statistics for liquor stores, as described below.

10.3 How Liquor Stores are Treated in Regular Finance Statistics

Liquor store activity represents one of the four distinct sectors of government defined for purposes of the Census Bureau's classification system for regular statistics on government finances, as explained in Chapter 2. Statistics for this activity are limited to expenditures for, and revenue from, the establishment and operations of alcoholic beverage distribution facilities and retail outlets. Revenue represents the money received from the sale of retail or wholesale liquor from such facilities. Expenditure represents the costs of goods sold, plus the direct cost of operation and maintenance of such facilities, including capital outlay if applicable.

For purposes of regular financial statistics, many related but indirect activities are excluded from the liquor stores sector. These exclusions are:

- Law enforcement, licensing, and regulation of private liquor outlets carried out in conjunction with liquor store operations.

- Collection of liquor taxes and licenses.

- Transfer of profits or surplus earnings to parent government (an internal transfer).

- State distribution of earnings to local governments.

These activities are classified in the general government sector for Census Bureau regular finance statistics purposes. However, if a government liquor store operation exists at the state government level, then the Census Bureau develops additional exhibit and derived statistics about the state liquor stores operations, as explained in Section 10.4.

Regular Census Bureau finance statistics thus provide only selected (partial) information on liquor stores activity. The critical perspective in the regular statistics program is to present the financial activity for the government as a whole. Regular finance statistics covering government activity for liquor stores include the revenue statistics described in Chapter 4 and the expenditure statistics described in Chapter 5. There are no separate cash and security holding statistics, or any separate debt statistics for liquor stores. Liquor

store cash and security holdings are classified in the general government sector at their appropriate code (*All Other Cash and Securities Holdings*, code W61, see Section 7.5). Government issued debt for liquor store purposes is rare, but if it exists such debt is classified in the general government sector in the appropriate category (see Section 6.4.7).

10.4 Liquor Stores Exhibit Statistics and Derived Statistics

As noted in Chapter 2, exhibit and derived statistics are used to enhance the regular statistics in two ways. First, they add additional detail to the regular statistics, showing further breakdowns of financial activity not used in the regular finance statistics, but that are important for a particular dependent activity of a parent government. Such is the case here, as the exhibit and derived statistics describe liquor stores from the perspective of a commercial enterprise activity. Secondly, the exhibit statistics are designed to account for (include) financial transactions that occur between the liquor stores operation and the parent government. Such transactions are excluded from regular statistics, by definition.

These additional exhibit and derived statistics apply only to state government liquor stores, as noted in the introduction to this chapter. The exhibit and derived statistics for state government operated liquor stores fall into two groups. The first provides a type of net income statement and is presented in this chapter with the regular finance statistics. The second group of exhibit and derived statistics shows the overall contribution of liquor stores, taxes, and liquor law enforcement activities on the government.

10.5 Description Pages for Liquor Stores

The following pages contain the liquor stores variables found in the Census Bureau statistical program. These pages contain all the codes in the Census Bureau classification system for government finances – regular statistics, exhibit statistics, and derived statistics.

The format for these description pages is similar to that used throughout the other chapters of this Manual. However, code titles are prefaced by the terms “Regular,” “Exhibit,” and “Derived Statistic” to distinguish the type of statistical code. This is a slight variation on the presentation format compared to other chapters.

Revenue, Receipts, and Income Sub-Category

Regular Code A90 Liquor Store Revenue - Current Charges

Definition: Gross receipts (less discounts and any sales taxes included in receipts) from sale of alcoholic beverages and related operations revenue of publicly-owned and operated liquor stores, ABC stores, and their variously-named equivalents.

Excludes: Sales and license taxes on alcoholic beverages collected through these stores (report at *Alcoholic Beverage Sales Tax*, code T10, or at *Alcoholic Beverage License Tax*, code T20); contributions from parent government (nonrevenues); receipt of state liquor store profits distributed to local governments (if unrestricted in use, report at *General Local Government Support from State Government*, code C30).

Examples: State governments – West Virginia Alcohol Beverage Control Administration, which controls the wholesale distribution of alcoholic beverages in that state.

Exhibit Code Z41 Net Sales of Goods

Definition: Amounts received from sales of liquor and associated services or products. Excludes any amounts for taxes, sales discounts, customer refunds, and other offsets to gross receipts from sales.

Exhibit Code Z44 Other Income

Definition: Nonoperating income – i.e., income from nonoperating properties of the system and sources other than sales, excluding any receipts from sales taxes, licenses, and permits that may be recorded by the system as "other income."

Exhibit Code Z48 Receipts from Sales Taxes, Licenses, and Permits

Definition: Any amounts received from alcoholic beverage sales and license taxes (including consumer permits to purchase liquor) that are included in the income accounts of the alcoholic beverage monopoly system. Such amounts are excluded from figures compiled for the income and expense statistics above and classified under general revenue categories.

Derived Statistic: Gross Profit on Sales

Definition: A derived statistic representing the profit on the sale of liquor before taking into account operating costs – i.e., *Net Sales of Goods* less *Cost of Goods Sold* (Z41 - Z42).

Derived Statistic: Net Operating Revenue

Definition: A derived statistic representing the net profit before taking into account other revenues and costs – i.e., *Gross Profit on Sales* less *Operating Expenses* (Z41 - Z42 - Z43).

Revenue, Receipts, and Income Sub-Category - continued

Derived Statistic: Net Income

Definition: A derived statistic representing the final profit of the liquor store system – i.e., *Net Operating Revenue* plus *Other Income* less *Nonoperating Expenses* (Z41- Z42 - Z43 + Z44 - Z45). Note that this figure may not agree with that reported by the liquor store system's own financial report, which could calculate its income on another basis.

Expenditure and Expenses Sub-Category

Regular Code 90 Liquor Stores Expenditure

Definition: Establishment and operation of alcoholic beverage distribution facilities and retail outlets owned and operated by seventeen states and by local governments in a few states.

Includes: Alcoholic beverage monopolies; ABC stores; liquor control boards; state liquor stores.

Excludes: Law enforcement, licensing, and regulation of private liquor outlets carried out in conjunction with liquor store operations (report licensing at *Financial Administration*, code *23, regulation and enforcement at *Protective Inspection and Regulation, NEC*, code *66, and law enforcement handled by regular police forces at *Police Protection*, code *62); collection of liquor taxes and licenses (use code *23); transfer of profits or surplus earnings to parent government (an internal transfer); state distribution of earnings to local governments (if undesignated in purpose report at *General Local Government Support*, code *30, else report at function involved).

Special Considerations:

1. Expenditures related only to amounts for purchase of goods for resale and for provision and operation of stores, warehouses, and related facilities (including administration and capital improvements).
2. Report expenditures without deducting cost of goods sold or operating expenses.
3. Includes direct expenditures only – report any related intergovernmental outlays at *Other and Unallocable*, code *89.
4. These regular statistics apply to state governments and to local governments. Exhibit and derived statistics, detailed following, apply only to state governments.

Expenditure and Expenses Sub-Category - continued

Applicable Object Coding Options for this Expenditure Function

Direct Expenditure		Intergovernmental Expenditure
E90	Current Operations	None
F90	Construction	
G90	Land and Existing Structures	
K90	Equipment	

Exhibit Code Z42 Cost of Goods Sold

Definition: Cost to the activity (net of any purchase discounts and other offsets to purchase price) of goods sold to produce the receipts from sales reported under *Net Sale of Goods*, code Z41, above.

Exhibit Code Z43 Operating Expenses

Definition: Expenditures applicable to operation and maintenance of liquor stores, including administration, advertising, purchase, handling, storage, and sale of merchandise (other than cost of goods sold), and other related costs of the liquor stores system. Excludes noncash outlays such as deprecation as well as licensing and law enforcement activities undertaken by the liquor store system.

Exhibit Code Z45 Nonoperating Expenses

Definition: Any costs of the system for interest and for current costs not falling within *Operating Expenses* above (code Z43), excluding any licensing and enforcement costs that may be recorded by the system as "nonoperating expense."

Exhibit Code Z46 Transfers to General Fund (Gross)

Definition: Total amount actually turned over to general government funds during the fiscal year, as shown in the system accounts (as opposed to the above calculated amounts), including any funds for distribution to local governments. Also includes any amounts derived from tax revenue collected by the system that appear in income accounts of the liquor system.

Exhibit Code Z47 Expenditure for Licensing and Law Enforcement

Definition: Any amounts recorded in the liquor system accounts as expense for licensing activities undertaken for the state government and enforcement of state liquor laws and regulations. Such amounts are excluded from figures reported under *Operating Expenses,* code Z43, and *Nonoperating Expenses,* code Z45, in the income and expense statistics above and classified under general expenditure categories.

Expenditure and Expenses Sub-Category - continued

Derived Statistic: Net Contributions to General Fund

Definition: A derived statistic representing the net effect of the liquor control system on the government's overall finances – i.e., *Transfers to General Fund (Gross)* plus *Expenditure for Licensing and Law Enforcement* minus *Receipts from Sales Taxes, Licenses, and Permits* (Z46 + Z47 - Z48).

10.6 Lottery System Statistics – Overview

Most state governments (and the government of Washington, DC) operate a lottery for purposes of raising revenue. Often, all or part of the lottery proceeds are dedicated to a particular function or functions, such as education. Chapter 4, on revenue statistics, contained some information on how lottery operations are treated in regular Census Bureau statistics on government finances. This chapter expands on that information, and further explains how the Census Bureau classification system takes into account all the financial transactions involved in lottery operations.

Unlike liquor stores, lottery operations do not constitute a distinct sector of government. Lotteries are classified as an activity of the general government sector for purposes of revenue and cash and security holding statistics. Expenditure of lottery proceeds is classified in whichever government sector and function the proceeds are spent. Usually that will be the general government sector, since lottery proceeds would not likely be spent for utility, liquor, or insurance trust purposes. However, that possibility remains within the Census Bureau's classification system.

10.7 Lottery System Definitions

Lotteries are games of chance, for which individuals pay for the privilege of participating and the probability of winning a prize. To be classified as a lottery, the lottery game(s) must be government sponsored, must accumulate revenue that is used to create a pool from which the prizes are awarded, and must yield proceeds over and above the value of prizes paid out. The proceeds are then used by the parent government for some public purpose.

Government sponsored lotteries take multiple forms, with options ranging from standard numbers-based games, to variations of what is commonly referred to as "keno," variations of "scratch off tickets," and even variations of video poker. As long as these are government-sponsored, all are included in the Census Bureau definition of lotteries.

10.8 How Lotteries are Treated in Regular Finance Statistics

Lottery operations are reported on a net basis in regular Census Bureau statistics on government finances. This treatment is different from utility and other commercial-type activities of state and local governments, which are reported on a gross basis without deduction for the costs of providing them. Lottery operations are distinctly identified only in the revenue category in regular finance statistics, by definition. This amounts, essentially, to reporting net lottery revenue as *Total Ticket Sales*, exhibit code Z51, minus the cost of *Prizes Awarded*, exhibit code Z52. Consequently, the only distinct lottery statistic that appears in the regular finance statistics is revenue statistic *Net Lottery Revenue*, code U95.

The cost of administering the lottery is reported under *Financial Administration*, code *23, in the regular finance statistics. Other expenditures from net lottery proceeds available are reported in whatever function spends them – education, general local government support, highways, etc. Since the Census Bureau's classification scheme does not relate expenditures to their source of funding, it is not possible to determine how net lottery proceeds are distributed among the standard Census Bureau expenditure functions.

The cost of prizes is not reported as an expenditure anywhere in regular finance statistics.

The rationale for treating lottery activity on a modified net basis for purposes of regular statistics was based on two considerations. One was the magnitude of money involved. Reporting revenue in the form

of gross lottery sales would have made lottery revenue the largest, or one of the largest, revenue items in many states. The same is true for lottery prizes and expenditure statistics. A second consideration concerned the uses (expenditure) of lottery proceeds. The Census Bureau already had a well established functional classification system for expenditures, and categorizing the expenditure of lottery proceeds by function was deemed to be more useful than creating a separate functional category exclusively for the expenditure of lottery proceeds.

Consequently, as lotteries became a significant revenue source in the early 1970's, the Census Bureau decided to report them on a modified net basis in the regular statistics on government revenue, and to functionalize the expenditure of net proceeds. This classification decision was effective with the 1972 Census of Governments.

At the same time, however, the Census Bureau created a number of exhibit statistics related to lottery operations, to give interested users the ability to review lottery operations on a gross basis, from a commercial enterprise perspective. These exhibit statistics are explained in detail in this chapter as well.

10.8.1 Special Topics: How Lottery System Cash and Security Holdings are Reported in Regular Finance Statistics

As noted in Section 7.3, cash and security holdings of lottery systems are reported in the general government sector for regular finance statistics. Assets held in funds of state-operated lottery systems present a special classification case. Lottery system assets that are held for future distribution of prize monies are excluded from the Census Bureau statistics on cash and security holdings. In effect, they are treated as agency funds, where the state has no discretion as to disbursement. Such funds are held in trust for the prize winners (usually those persons who choose to receive their winnings over a period of time, rather than in one lump sum). Similarly, interest earnings on such lottery funds held in trust are excluded from Census Bureau statistics on *Interest Earnings*, code U20. This overall treatment of lottery system finances is consistent within the Census Bureau classification system, where code U95 represents *Net Lottery System Revenue* (see Chapter 4, Revenue).

Generally, lottery system cash and security holdings other than those held for payout of prizes are included in Census Bureau statistics. Examples would include current operating funds and capital funds, if any. They are reported at *Cash and Security Holdings, All Other Funds*, code W61 (see Chapter 7).

10.9 Exhibit Statistics for Lotteries – Receipts and Expenses

Census Bureau exhibit statistics for lottery systems cover receipts and expenses only. They provide users with a stand-alone perspective of the lottery system operations of each government, separate from the lottery's relationship with the parent government. There are four statistics, as noted below. These are defined fully on the description pages for lottery statistics.

**Code**	**Title**
Z51	Total Ticket Sales
Z52	Prizes Awarded
Z53	Administrative Expense
Z54	Proceeds Available

10.10 Description Pages for Lottery Systems

The following pages contain the lottery system variables found in the Census Bureau statistical program. These pages contain all the codes in the Census Bureau classification system for government finances – regular statistics and exhibit statistics.

The format for these description pages is similar to that used throughout the other chapters of this Manual. However, code titles are prefaced by the terms “Regular” or “Exhibit” to distinguish the type of statistical code. This is a slight variation on the presentation format compared to other chapters.

Regular Code U95 Net Lottery Revenue

Definition: Proceeds from the operation of government-sponsored lotteries after deducting the cost of prizes.

Special Considerations:

1. In terms of lottery exhibit codes, this item is computed by subtracting *Prizes Awarded*, code Z52, from *Total Ticket Sales*, code Z51. Thus, code U95 includes amounts used for administration that are reported at *Administrative Expenses*, code Z53.

2. This code does not apply to Federal Government statistics. While U95 can apply to all types of local governments, the related lottery exhibit statistics below (Z51 through Z54) apply only to state governments and to Washington, DC.

Exhibit Code Z51 Total Ticket Sales

Definition: Gross amounts received from the sale of lottery tickets or games, before deduction for cost of lottery tickets and prizes. Excludes commissions and bonuses allowed to merchants for selling the tickets or games.

Exhibit Code Z52 Prizes Awarded

Definition: Total value of prizes awarded during the fiscal year, whether actually paid out or incurred. Includes “instant winners,” lotto jackpots to be paid as annuities, funds transferred to prize reserves, and the like.

Exhibit Code Z53 Administrative Expense

Definition: The cost of administering the lottery, except for prizes. Includes salaries of officials as well as advertising, supplies, and the like. An identical amount is reported as an expenditure under *Financial Administration,* code *23.

Exhibit Code Z54 Proceeds Available

Definition: The net funds available after deducting the costs of prizes and administration (Z51 - Z52 - Z53).

Special Consideration:

Note that this amount is not the same as that classified as *Net Lottery Revenue*, code U95; the latter represents the amount left over after deducting prizes only (Z51 - Z52).

10.11 Applicability of Liquor Stores and Lottery Codes by Level and Type of Government

Table 10.1 at the end of this chapter displays the applicability of liquor store and lottery codes, by level and type of government. Note that there are no Federal statistics for these two types of financial activities. For liquor stores, the regular statistics for revenue and expenditure apply to state, county, municipal and township governments. There are no special district liquor stores identified for Census Bureau statistical purposes. The exhibit and derived statistics apply only to state government liquor store operations.

For lottery systems, the regular revenue statistic Net Lottery Revenue, code U95, applies to state and all types of local governments. However, as of 2005, the only local government identified by the Census Bureau as having a lottery system is the municipality of Washington, DC. The lottery exhibit statistics apply only to state governments and to Washington, DC. If local governments in other states are permitted to operate lottery systems, the Census Bureau will not collect the lottery exhibit statistics from them.

10.12 Tables

There are two tables associated with this chapter. Table 10.1, Liquor Stores and Lottery System Code Validity, by Level and Type of Government, shows the regular, exhibit, and derived statistics that exist in the classification system and how they apply to each level and type of government. Table 10.2, Summary of Liquor Stores and Lottery System Finance Codes and Statistics, is an easy reference for determining which codes defined in this chapter are regular, exhibit, or derived statistics in the Census Bureau classification system.

Table 10.1<br>Liquor Stores and Lottery System Code Validity,<br>by Level and Type of Government									
			Applicability by Level and Type of Government:						
**Liquor Stores**		**Code Type**	**Federal**	**State**	**County**	**Municipal**	**Township**	**Special District**	**School District**
Receipts or revenue related categories:									
A90	Current charges	R	X	Valid	Valid	Valid	Valid	X	X
Z41	Net sales	E	X	Valid	X	X	X	X	X
Z44	Other income	E	X	Valid	X	X	X	X	X
Z48	Taxes and licenses	E	X	Valid	X	X	X	X	X
.....	Net operating revenue	D	X	Valid	X	X	X	X	X
.....	Gross profit	D	X	Valid	X	X	X	X	X
.....	Net income	D	X	Valid	X	X	X	X	X
Expenses or expense related categories:									
E90	Current operations	R	X	Valid	Valid	Valid	Valid	X	X
F90	Construction	R	X	Valid	Valid	Valid	Valid	X	X
G90	Land and structures	R	X	Valid	Valid	Valid	Valid	X	X
K90	Equipment only	R	X	Valid	X	X	X	X	X
Z42	Cost of goods sold	E	X	Valid	X	X	X	X	X
Z43	Operating expenses	E	X	Valid	X	X	X	X	X
Z45	Nonoperating expenses	E	X	Valid	X	X	X	X	X
Z46	Transfer - general fund	E	X	Valid	X	X	X	X	X
Z47	Licensing and enforcement	E	X	Valid	X	X	X	X	X
.....	Net contributions	D	X	Valid	X	X	X	X	X
**Lotteries**		**Code Type**	**Federal**	**State**	**County**	**Municipal**	**Township**	**Special District**	**School District**
U95	Net lottery revenue	R	X	Valid	Valid	Valid	Valid	Valid	Valid
Z51[1]	Total ticket sales	E	X	Valid	X	X[1]	X	X	X
Z52[1]	Prizes awarded	E	X	Valid	X	X[1]	X	X	X
Z53[1]	Administrative expense	E	X	Valid	X	X[1]	X	X	X
Z54[1]	Proceeds available	E	X	Valid	X	X[1]	X	X	X

Key:
Valid   Statistics can be reported for these governments.
X   Statistics cannot be reported for these governments.

Code Type = type of statistic
R = regular
E = Exhibit
D = Derived

Note:
[1]Valid for Washington, DC, only.

Table 10.2<br>Summary of Liquor Stores and Lottery System Finance Codes and Statistics				
**Code**	**Brief description**	**Regular code**	**Exhibit code**	**Derived code**
	**Liquor Stores**			
	Receipts and related categories:			
A90	Current charges	Yes	-	-
Z41	Net sales	-	Yes	-
Z44	Other income	-	Yes	-
Z48	Taxes and licenses	-	Yes	-
....	Net operating revenue	-	-	Yes
....	Gross profit	-	-	Yes
....	Net income	-	-	Yes
	Expenses and related categories:			
E90	Current operations	Yes	-	-
	Capital outlay:			
F90	Construction	Yes	-	-
G90	Purchase of land and structures	Yes	-	-
K90	Equipment only	Yes	-	-
Z42	Cost of goods sold	-	Yes	-
Z43	Operating expenses	-	Yes	-
Z45	Nonoperating expenses	-	Yes	-
Z46	Transfer to general fund	-	Yes	-
Z47	Licensing and enforcement	-	Yes	-
....	Net contributions	-	-	Yes
	**Lottery Systems**			
U95	Net lottery revenue	Yes	-	-
Z51	Total ticket sales	-	Yes	-
Z52	Prizes awarded	-	Yes	-
Z53	Administrative expense	-	Yes	-
Z54	Proceeds available	-	Yes	-

Key:

"Yes" Indicates applicable for that type of statistic.

\- Indicates not applicable for that type of statistic.

PART 3. PUBLIC EMPLOYMENT AND PAYROLL STATISTICS

This part of the *Classification Manual* covers the Census Bureau's surveys on public employment. The Census Bureau first collected data on state and local government employment and payrolls in 1940. The Department of Labor now conducts monthly surveys of public employment, while the Census Bureau continues to compile detailed statistics on an annual basis, as described in the following two chapters.

Public employment statistics are collected and developed during each census of governments, for all 80,000 plus governments in the nation. The Census Bureau also conducts an annual sample survey of state and local government employment, and develops statistics on Federal Government employment that serve to complement the state and local government numbers.

In addition to a detailed discussion of the Census Bureau's survey on public employment statistics, the following two chapters also contain a description of the differences between the surveys on government finance and employment. While the basic concepts are identical, there are coverage differences in time frames and differences in the functionality for categorizing statistics.

Chapter 11. Overview of the Public Employment Statistics

Introduction

The purpose of the Census Bureau's employment survey is to measure the number of public employees and their payrolls annually, according to a detailed cross-classification by function and type of employee (full- or part-time). The primary focus of the annual survey is state and local governments. However, the Census Bureau compiles Federal Government employment statistics from the U.S. Office of Personnel Management, to complement the state and local data and to yield complete coverage of the nation's government sector employment activity.

11.1 Survey Coverage and Definitions

The basis of coverage for the employment surveys is the government unit, as defined in Chapter 1. Employment statistics are collected for individual government units, then aggregated by geographic area, level of government, or type of government. The employment surveys cover all three levels of government – Federal, state, and local.

11.1.1 Employment Statistics on State and Local Governments

In total, there are eight basic statistics in the annual and census employment surveys:

Number of full-time employees	Payrolls (converted to Monthly Payrolls)
Number of part-time employees	Average monthly earnings
Full-time equivalent employees	Hours worked by part-time employees
Standard hours worked	Pay interval

Six of the eight statistics are collected from (reported by) the individual government units canvassed in the surveys, including the payrolls statistic. However, this statistic is converted during processing to a monthly basis by the Census Bureau, as described later in this chapter (see Section 11.1.4). Two statistics are calculated by the Census Bureau using standard methodology, also described later in this chapter. The chart on the next page summarizes how the eight employment statistics are obtained.

Chart 11.A Sources of the Eight Employment Statistics	
**Statistic:**	**Source:**
Full-time employees.................................	Reported by respondent
Part-time employees	Reported by respondent
Full-time equivalent employees	Calculated
Standard hours worked..............................	Reported by respondent
Payrolls ....……...................................	Reported, then converted to monthly basis
Average monthly earnings	Calculated
Hours worked by part-time employees .….	Reported by respondent
Pay interval	Reported by respondent

Each of these statistics is categorized by function. Functional categories are used in the employment surveys to group statistics so that they can be aggregated and compared across governments or geographic areas. The functional categories for employment surveys are similar to, but not identical with, those used for expenditure statistics in the Census Bureau's surveys on government finance. The functional categories are explained in more detail in Chapter 12, along with definitions and examples.

11.1.2 Employees/Employment Definition

Employment refers to all persons gainfully employed by and performing services for a government. Employees include all persons paid for personal services performed from all sources of funds, including persons paid from Federally-funded programs, paid elected officials, persons in a paid leave status, and persons paid on a per meeting, annual, semiannual, or quarterly basis.

Several categories of "workers" are excluded from the public employment statistics. These do not fall within the definition of paid employees of the government unit, even though the work they perform is done on behalf of the government or benefits the government in some way. Unpaid officials, pensioners, persons whose work is performed on a fee basis, volunteers (including volunteer firemen who receive no remuneration), and contractors and their employees, all are excluded from the counts of employees for a government.

11.1.3 Full-Time and Part-Time Employees

The classification of an employee as full- or part-time is determined by the standard used by the reporting government itself:

Full-time employees are defined to include those persons whose hours of work represent full-time employment in their employer government.

Part-time employees are those persons who work less than the standard number of hours for full-time work in their employer government.

11.1.4 Payrolls

Payroll amounts are gross payrolls for the one-month period of March. Gross payrolls includes all salaries, wages, fees, commissions, and overtime paid to employees before withholdings for taxes, insurance, etc. It also includes incentive payments that are paid at regular pay intervals. It excludes employer share of fringe benefits like retirement, Social Security, health and life insurance, lump sum payments, and so forth.

Monthly Payrolls represent a calculated statistic. Data collected for the one pay period that includes March 12 are converted to monthly figures using the conversion factors noted in the following chart:

Chart 11.B Conversion Factors for Monthly Pay

Frequency of Pay Period	Conversion Factor
Weekly	4.429
Biweekly	2.214
Twice a month	2.000
Monthly	1.0
Quarterly	0.340 or 0.442[1]
Semi-annually	0.170 or 0.277[1]
Annually	0.085

[1]Conversion used for institutions of higher education only.

11.1.5 Hours Worked by Part-Time Workers

First collected during the October 1986 survey, these data represent the number of hours worked by part-time employees during the pay period. Note that these data are not collected for publication but rather are used to calculate full-time equivalent employment statistics.

11.1.6 Full-Time Equivalent Employment

The term "full-time equivalent employment" refers to a calculated statistic representing the number of full-time employees that could have been employed if the reported number of hours worked by part-time employees had been worked by full-time employees. This statistic is calculated separately for each function of a government by dividing the "part-time hours paid" by the standard number of

hours for full-time employees in the particular government, and then adding the resulting quotient to the number of full-time employees.

This statistic is computed for each functional category of a government using the following formula:

$$\text{Full-Time Employees} + \frac{\text{Part-Time Hours Paid}}{\text{Standard Number of Hours for Full-Time Employees in that Government}}$$

<u>11.1.6.1 Special Topics: Note Regarding the October 1986 Revision to Full-Time Equivalent Calculation Method</u>

The method for calculating full-time equivalent employment based on part-time hours worked has been in use since the 1986 employment survey. Prior to the 1985 survey, the Bureau used a different method, one based on payrolls. (For the October 1985 employment survey no full-time equivalent employment data were calculated.)

This payroll method calculated full-time equivalent employment for each functional category of a government using the following formula:

$$\text{Full-Time Employees} \times \frac{\text{Full-Time Payrolls}}{\text{Full-Time Payrolls} + \text{Part-Time Payrolls}}$$

This payroll-based method was discontinued when “hours of work” data became more generally available as a result of changes to the Fair Labor Standards Act.

<u>11.1.7 Average Monthly Earnings – Full-Time Employees</u>

Average monthly earnings is a calculated statistic representing the average monthly payrolls per full-time employee. This statistic is calculated for each functional category of a government using the following formula:

$$\frac{\text{Full-Time Employee Monthly Payrolls}}{\text{Number of Full-Time Employees}}$$

Average earnings data may not convert to accurate annual earning rates because of overtime, unusual situations (e.g., special events, natural disasters), and such factors as employees who are paid only during 9 or 10 months of the year (especially in education and natural resources).

Other factors affecting average monthly earnings rates include (1) the proportion of highly trained or skilled personnel, (2) the concentration of employees in metropolitan or urban areas where the cost-of-living is higher, and (3) the exclusion of housing, meals, or other “in kind” compensation, which may be provided to employees (especially for hospitals).

<u>11.1.8 Standard Hours Worked</u>

This is the number of hours worked by employees considered to be "full-time" by the responding government. This statistic varies for each government. It is used in the calculation of the "full-time equivalent employment" statistic, as described above in Section 11.1.6.

11.1.9 Pay Interval

This represents the length of the standard pay period for the respondent government or government agency. This statistic is collected from each respondent. It is necessary in order to standardize March payrolls (convert payrolls to a monthly basis in accordance with the methodology described in Section 11.1.4).

11.2 Employment Statistics for the Federal Government

The Census Bureau includes the Federal Government in its surveys of public employment. Coverage involves two sets of statistics:

- Functional measures consistent with the statistics for state and local governments, and
- Federal employment by state area

11.2.1 Federal Government – Employment By Function

These annual statistics are compiled from the U.S. Office of Personnel Management and based on that agency's Monthly Report of Federal Civilian Employment (Standard Form 113A). Only three statistics are developed, compared to the eight statistics for state and local governments:

- Full-time employees
- Part-time employees
- March payrolls

The payrolls data are a total monthly payrolls only. There is no detail available for full-time or part-time employee payrolls. Consequently, there are no statistics calculated on full-time equivalent employment.

The data cover all civilian employees, including seasonal and intermittent employees, as well as employees on foreign assignments residing outside of the fifty states and the District of Columbia. Employees of the Central Intelligence Agency, National Security Agency, and Defense Intelligence Agency are not included in the functional data. Federal judges, members of Congress and their staffs, employees of the Congressional Budget Office, and elected and appointed officials of the Executive Branch are included.

The data are classified by function in the same manner as for state and local governments (as detailed in Chapter 12). Three function categories are applicable to the Federal Government only and do not apply to state and local governments. These are shown below and explained fully in Chapter 12:

- Federal National Defense and International Relations

- Federal Postal Service

- Federal Space Research and Technology

11.2.2 Federal Government – Employment By State Area

Federal civilian employment data by state are compiled biennially in even numbered years. There is one statistic only, total employment, with no measures of full- or part-time employment, pay intervals, payrolls, or full-time equivalent employment.

The data also differ from the annual functional measures of Federal Government employment in other ways involving coverage. First, the following employees are excluded:

- Seasonal and intermittent employees.

- Employees on foreign assignment.

- Commissioned Corps of the Public Health Service.

- Intelligence agencies (Central Intelligence Agency, National Security Agency, Defense Intelligence Agency).

Second, two categories of employees are included in the total count but not allocated by state. These are Federal judges and other employees of the Federal courts, plus employees of the Federal Bureau of Investigation stationed outside of the District of Columbia.

Members of Congress and their staffs (including the Congressional Budget Office) and appointed officials of the Executive Branch working in the District of Columbia are included in counts for Washington, DC.

11.3 Reporting Periods

The Census Bureau measures government employment and payrolls statistics in a traditional manner, by using a specific time frame applicable to all governments. Currently, the time frame used is the month of March. Statistics are collected from each government for its pay period that includes March 12, regardless of the length of the pay period.

March has been the measurement period since the 1997 survey year. However, the time frame has changed twice since 1957, when the Census Bureau began collecting public employment statistics in the current survey format. The month used for different survey years is shown below:

1997 - present -	March
1958-1995 -	October
1957 -	April

(The employment survey was not conducted in 1996)

For counts of employees, the statistics are straightforward and represent the number employed during the pay period that included March 12. However, for government payrolls measures, the Census Bureau converts the statistics reported by government respondents to a monthly basis, as described earlier (Section 11.1.4). This is because governments have pay periods that differ in length. Converting the

payrolls statistics to a standard monthly basis enables aggregates to be tabulated and allows comparisons among government units to be made more easily.

During the survey years from 1958 to 1995, the payrolls period for coverage purposes had to include the date of October 12.

The reporting period for statistics covering the Federal Government differ. For annual statistics that show employment by function, the reporting period is December, but this has varied as follows:

2003 - present -	December
1997 - 2002 -	March
1992 - 1995 -	October

(The employment survey was not conducted in 1996)

For the biennial statistics on Federal employment by state area (compiled only in even number years), the statistics reflect December of the year indicated. Users can refer to the Census Bureau's website for updated information on reporting periods.

11.4 Joint Activities of Governments

As noted in Section 1.5, government units often cooperate to provide a specific service or activity. Section 1.5 explained three methods for the classifying employment activity under these cases. The first two methods, summarized below, are straightforward. This section further explains the third methodology.

1. Governments might establish an independent special district to carry out the activity, in which case the related employees and payrolls are assigned to that government unit.

2. One of the governments might be solely responsible for administering the activity, but the other governments share its financial support. In such situations, all employment statistics are assigned to the administering government.

3. The participating governments might create a separate organizational body that is neither independent nor dependent on any one member, but is jointly administered by all of them. An example of this occurrence is where the joint agency issues debt that is not in the name of any of the participating governments. These types of joint activities provide special problems in a classification system where the focus is the individual government entity. Such joint activities, therefore, are classified on the basis of the circumstances involved in each case. For this third type of situation, the Census Bureau has three options for classification:

    3a. A joint agency having substantial financial and employment activity can be treated (classified) as if it were an independent special district government.

    3b. A joint inter-local agency having substantial financial and employment activity might be assigned as a dependent agency of one of the participating governments. Its employees are classified in the same manner as the second case cited above.

    3c. A joint inter-local agency not having substantial financial activity or employment is allocated to each of the participating governments to the extent of their respective participation. In this case, employees and employee payrolls should be reported as part of the government that issues employee paychecks or, if the joint agency issues

paychecks, allocated to the sponsoring governments on the basis of their financial contribution to the “joint” activity.

11.5 Special Topics: Classification Issues for Washington, DC

The District of Columbia and the city of Washington, contained within it, constitute special handling in Census Bureau statistics on government finance and public employment. The District of Columbia is classified as a municipal government for Census of Governments purposes. However, it is a unique government entity in many respects, having characteristics of both state and local governments. As a result, there are several finance and employment classification categories that apply solely to Washington, DC and to no other local governments. The finance categories are detailed in Section 3.13.

Like all state governments, Washington, DC administers a Federally-Sponsored unemployment insurance program. Hence, Washington, DC has employee and payrolls statistics for *Social Insurance Administration*, code 22. This employment classification function applies to state governments and to Washington, DC, only.

11.6 Special Topics: Additional Statistics During Census of Governments

The Census Bureau occasionally develops additional statistics about public employees and public employment, especially during the Census of Governments.[1] This statistical information has varied since the first Census of Governments in 1957. Two primary topics were labor management relations and government costs for employee benefits. Users should refer to the Census Bureau’s website for additional information on the availability of special topics during Census of Governments survey years.

[1]The Census of Governments is taken at 5 year intervals as part of the Nation’s Economic Census.

Chapter 12. Functional Categories for Employment Statistics

Introduction

This chapter contains the detailed list of functional categories used for classifying the Census Bureau's public employment statistics. The functional categories are similar to the expenditure categories in the government finances statistics program. However, there are some differences, as described throughout Chapter 12.

12.1 Overview

All eight of the basic employment statistics defined and explained in Chapter 11 are applicable to each of the functions (with the exception of functions applicable only to the Federal Government – see Section 11.2):

Number of full-time employees	Payrolls (converted to Monthly Payrolls)
Number of part-time employees	Average monthly earnings
Full-time equivalent employees	Hours worked by part-time employees
Standard hours worked	Pay interval

The functional categories used in the employment survey are similar to those used for expenditure statistics in the government finance survey. Most of the functional categories are the same in both surveys, and they are defined identically, covering the same types of government services or programs. However, there are two areas where the employment survey functions differ from those used for government finance statistics. First, there are fewer functional categories in the employment survey. Second, the employment survey uses functional sub-categories that are not found in the finance survey. These are summarized below and defined in detail later in this chapter:

- Higher Education
  - Instructional Employees
  - Other Higher Education Employees
- Elementary and Secondary Education
  - School Instructional Employees
  - All Other [1]
    - Administrative and Clerical Employees
    - Operations and Maintenance Employees
    - Cafeteria Employees
    - Bus Transportation Employees
    - Health and Recreation Employees
    - Paid Student Employees
    - Unallocable Elementary and Secondary Education Employees
- Police Protection
  - Police Officers
  - Other Police Protection Employees
- Fire Protection
  - Firefighters

[1] Prior to 2001, data were collected separately for the items listed under "All Other." These are no longer collected separately.

Other Fire Protection Employees

Table 12.1, at the end of Chapter 12, compares the functions applicable to expenditure statistics in the government finance surveys to the functional categories used in the employment statistics.

12.2 Key to the Employment Function Pages

The complete listing of functional categories for employment statistics follows. Each function is presented on a separate page, using a standard format that contains the following information:

Definition
Included activities or employees
Excluded activities or employees
Examples (specific governments, by name, or governmental agencies/programs, by name), if applicable
Sub-categories (with definitions and codes; applicable to four functions only – listed in Section 12.2.1)
Special Considerations (explains caveats, restrictions in applicability, or other useful information)
Applicability table (by level and type of government)

At the bottom of each page, a table whether each function applies to the types of governments covered in the employment survey. This is needed because some employment functions apply only to selected types of governments. The table contains three columns representing the three levels of government – Federal, state, and local. Within the Federal and state columns, a straightforward "Yes" or "No" indicates whether the statistics are collected for that level of government at each function. Within the local column, the following key applies:

No – no statistics for local governments
Yes – statistics for all types of local governments are collected
Type 1 – No or Yes (indicates applicability to county governments)
Type 2 – No or Yes (indicates applicability to municipal governments)
Type 3 – No or Yes (indicates applicability to township governments)
Type 4 – No or Yes (indicates applicability to special district governments)
Type 5 – No or Yes (indicates applicability to school district governments)
No (except Washington, DC) – there are statistics for Washington, DC only, at the local level (limited to only one function, Social Insurance Administration, code 22), where Washington, DC represents a special case owing to its unique relationship with the Federal government.

Note that in all cases, "Yes" indicates only that statistics are collected for the level and type of government if they perform the function or provide the particular service. In function 01, for example, the table reads "Yes" for types 1, 2, 3, and 4 local governments. However, only a limited number of local governments actually provide airport services, so for most governments there will be no airport employees counted. The table reads "No" for type 5 local governments, because school districts do not operate airports.

12.3 Description Pages for Employment Functions

The following pages contain the detailed classification categories used in the Census Bureau's program on public employment. Each category is defined and explained, with examples provided where appropriate. All statistical codes represent regular statistics within the Census Bureau's classification system. There are no exhibit, derived, or descriptive statistics associated with the public employment surveys. There are, however, special sub-categories for four employment functions, as explained in Section 12.1. These are noted in the following Description Pages.

EMPLOYMENT FUNCTIONS

Code 01 **Air Transportation (Airports)**

Sector: General Government

Definition: Employees involved in the provision, operation, construction, and support of airport facilities serving the public at-large on a scheduled or unscheduled basis. Also includes the regulation of airline industry, if applicable.

Includes: Publicly-operated airfields and related facilities (runways, terminals, control towers, maintenance facilities, and the like); airport police and airport firefighters if either are an integral part of the airport operating authority or a payment is made to a regular police or fire agency.

Excludes: Operation of government-owned aircraft – e.g., police helicopters (report at function involved); state civil air patrol or militia (report at *Other and Unallocable*, code 89). For the Federal Government, excludes activities of the National Aeronautics and Space Administration (NASA), classified at *Federal Space Research and Technology,* code 02.

Examples:

- Federal Government – Federal Aviation Administration.
- State governments – Baltimore/Washington International Thurgood Marshall Airport (a Maryland state government dependent agency).
- Local governments – Washington Dulles International Airport in Loudoun County, Virginia (operated by a special district government, the Washington Metropolitan Airports Authority), Tucson Airport Authority (dependent agency of Tucson, Arizona).

Sub-Categories for Employment Statistics

None.

Special Considerations:

1. Includes publicly-owned airports, even if no scheduled airlines service it, or if its clientele consists of only private pilots and aircraft.
2. For leased facilities include government’s expenditures and employees and exclude the lessees’ expenditures and contractual employment.

Applicability of Function 01, by Level and Type of Government

Federal	State	Local
Yes	Yes	Types 1,2,3,4 -Yes<br>Type 5 - No

EMPLOYMENT FUNCTIONS

Code 02 **Federal Space Research and Technology**

Sector: General Government

Definition: Federal Government activities for research, development, applications, and support in the areas of aeronautics and space transportation, sciences, and technology.

Includes: Comprises employees of the National Aeronautics and Space Administration (NASA).

Excludes: Department of Defense research and other activities related to missiles, satellites, space weapons, etc. (report at *Federal National Defense and International Relations*, code 06).

Examples: The National Aeronautics and Space Administration (NASA) is the only Federal agency whose employees and activity are included at this function.

Sub-Categories for Employment Statistics

None.

Special Consideration:

This function applies only to the Federal Government.

Applicability of Function 02, by Level and Type of Government

Federal	State	Local
Yes	No	No

EMPLOYMENT FUNCTIONS

Code 05 **Corrections**
page 1 of 2

Sector: General Government

Definition: All institutional and non-institutional correctional activities. Institutional activities are residential institutions or facilities for the confinement, correction, and rehabilitation of convicted adults or juveniles adjudicated delinquent or in need of supervision, and for the detention of adults and juveniles charged with a crime and awaiting trial. Non-institutional correctional activities consist of pardon, probation, and parole activities.

Includes: For institutional activities, includes prisons; reformatories; jails; houses of corrections; penitentiaries; correctional farms; workhouses; reception centers; diagnostic centers; industrial schools; training schools; detention centers; multi-institutional programs and administration; education, training, and health care programs devoted to inmates; hospitals for the criminally insane IF operated by a corrections agency; and these types of facilities IF residential: work release units, halfway houses, and community corrections centers.

For non-institutional activities, includes probation offices, whether operated by courts or correctional agencies, boards of parole, boards of pardon, and the like; noninstitutional activities such as administration of a correctional agency, training of correctional employees, and nonresidential halfway houses and community corrections centers.

Excludes: Employees at police "lockups" or jails holding people awaiting arraignment (report at *Police Protection*, code 62); employees of hospitals for criminally insane that are operated by mental health or hospital agencies (report at *Hospitals*, code 36).

Examples:

- Federal Government – Federal prison system.
- State governments – State departments of correction, boards of pardon and parole.
- Local governments – Note that cities in Texas and California do not operate correctional facilities (although an exception for California is San Francisco, which does operate a prison).

Sub-Categories for Employment Statistics

None.

Code 05 **Corrections**
page 2 of 2

Special Consideration:

This is the only "corrections" function for employment statistics and therefore includes both institutional and other corrections. This differs from the government finance statistics, which has two functions for corrections (*Correctional Institutions,* code *04 and *Other Corrections*, code *05).

Applicability of Function 05, by Level and Type of Government

Federal	State	Local
Yes	Yes	Types 1, 2, 3 - Yes<br>Types 4, 5 - No

EMPLOYMENT FUNCTIONS

Code 06 **Federal National Defense and International Relations**

Sector: General Government

Definition: Federal Government activities to protect the United States and its allies from foreign aggression, to maintain military capabilities for deterring war, to protect and advance its interests in international affairs, and to provide military, economic, and humanitarian aid to other nations.

Includes: Employment activities covered include three general areas: military, diplomatic, and foreign assistance.

- The military services, National Guard and Reserves forces, intelligence agencies, defense research and evaluation, atomic energy defense research, development, and production.
- Conduct of diplomatic and consular relations, and international communication, education, and cultural activities.
- Employees engaged in such programs as Food for Peace and the Foreign Agriculture Service.

Excludes: Armed Forces Retirement Home and the Cemeterial Expenses Department (report at *Other Public Welfare*, code 79); Army Corps of Engineers (allocate between *Natural Resources*, code 59, and *Sea and Inland Port Facilities*, code 87); Department of Energy other than atomic defense activities (report at *Natural Resources*, code 59); state-local militia, civil air patrol, civil defense, and other defense-related activities (use code 89).

Examples: See above. Comprises primarily the departments of Defense, Energy (part), and State, as well as such agencies as U.S. Information Agency, Central Intelligence Agency, Agency for International Development (AID), Peace Corps, Export-Import Bank and International Monetary Fund, and the Foreign Agriculture Service (part of U.S. Department of Agriculture).

Sub-Categories for Employment Statistics

None.

Special Considerations:

1. This function applies solely to the Federal Government. Report related state or local government activities at *Other and Unallocable,* code 89.
2. Report military service academies, such as West Point, here rather than at *Education,* code 21.

Applicability of Function 06, by Level and Type of Government

Federal	State	Local
Yes	No	No

Code 12 **Elementary and Secondary Education**

Sector: General Government

Definition: Employees involved in the operation, maintenance, and construction of public schools and facilities for elementary and secondary education (kindergarten through high school), vocational-technical education, and other educational institutions except those for higher education, whether operated by independent governments (school districts) or as integral agencies of state, county, municipal, or township governments; and financial support of public elementary and secondary schools.

Includes: Employees in instructional, support, and auxiliary services operated through school systems (school lunch, student activities, community services, pupil transportation, health services, guidance counseling, and the like); administration and supervision of school systems; special education, classes for the handicapped, and vocational education provided through school systems; Headstart; libraries operated by public schools; and plant maintenance and operation.

Excludes: Employees of institutions of higher education (use *Higher Education*, codes 16 and 18); schools for the blind, deaf, or handicapped (if primarily for training and education, report at *Other Education*, code 21; if primarily for physical rehabilitation and care, report at *Hospitals*, code 36); state adult, vocational, and special education programs operated outside school systems (use code 21). For state governments also exclude administrative expenses of school building agencies and supervision of local public and private elementary-secondary education (use code 21).

Examples:

- State governments – Employees of Patterson City School District, under temporary state control; elementary and secondary education employees in Hawaii, where the state government operates the sole school system.
- Local governments – Employees of the Los Angeles Unified School District (an independent local government); elementary and secondary education employees of the New York City School System (classified as a dependent agency of New York City).

Sub-Categories for Employment Statistics – Education Services

Code 012. School Instructional Employees – Includes classroom teachers, principals, supervisors of instruction, superintendents, teacher aides, substitute teachers, school librarians, library aides, and guidance and psychological personnel.
Code 112. Administrative and Clerical Employees
Code 112. Operations and Maintenance Employees
Code 112. Cafeteria Employees
Code 112. Bus Transportation Employees
Code 112. Health and Recreation Employees
Code 112. Paid Student Employees
Code 112. Unallocable Elementary and Secondary Education Employees

Special Considerations:

1. Employment statistics at code 12 represent the sum of the eight sub-categories.
2. Includes charter schools offering elementary and secondary education, if they have been classified as in-scope for the Census of Governments.
3. Includes employees of Education Service Agencies and Regional Occupational Programs, whether independent or dependent on other school districts.
4. As noted under examples, state government statistics can include local school systems or districts that have been taken over by the state temporarily, for oversight purposes, whether by administrative action or by court order.
5. The state of Hawaii operates both public elementary and secondary schools, as well as public libraries. The latter are reported at *Libraries*, code 52, for Census Bureau statistics on public employment, but financial activity for the library is reported as part of the elementary and secondary school activity at code *12.

Applicability of Function 12, by Level and Type of Government

Federal	State	Local
No	Yes	Yes

Code 14 **Federal Postal Service**

Sector: General Government

Definition: Activities of the U.S. Postal Service (USPS).

Includes: Includes employees engaged in all postal activities, such as mail delivery, sorting and distribution, sales, financial administration, management, and investigation of fraud or criminal activity.

Excludes: Employees not under the USPS.

Sub-Categories for Employment Statistics

None.

Special Considerations:

1. The former cabinet-level Post Office Department became an independent Federal corporation and was renamed the U.S. Postal Service on July 1, 1971, as a result of the Postal Reorganization Act of 1970 (P.L. 91-375).

2. Report USPS criminal and fraud investigation activity here, rather than at *Police Protection*, code 62.

Applicability of Function 14, by Level and Type of Government

Federal	State	Local
Yes	No	No

EMPLOYMENT FUNCTIONS

Code 18 **Higher Education**

Sector: General Government

Definition: Employees of degree-granting institutions (associate, bachelor, master, or doctorate) operated by state or local governments, that provide academic training beyond the high school (grade 12) level.

Includes: Junior colleges; community colleges; universities; law schools; medical and nursing schools; agricultural colleges; land grant institutions; engineering schools; and other institutions granting postsecondary degrees. Includes employees engaged in all related activities for instruction, research, public service (including agricultural extension services), academic support, libraries, student services, administration, and plant maintenance.

Excludes: Employees of training academies or programs that do not confer college-level degrees (e.g., police academies); state vocational-technical schools that award certificates equal to less than 2-years of college (report at *Other Education*, code 21); non-instructional staff of hospitals for the general public operated by universities (report at *Hospitals*, code 36); agricultural experiment stations, farms, and extension services (report at *Natural Resources*, code 59); state administration of school building authorities (use code 21); Federal military academies such as West Point (report at *Federal National Defense and International Relations*, code 06).

Examples: State governments – State University of New York (SUNY) system, including all graduate programs, four-year universities, and colleges (dependent agencies of the state of New York).

Sub-Categories for Employment Statistics

Code 018. Instructional Employees

Code 016. Other Higher Education Employees

Special Considerations:

1. Report employment data for the instructional staff only of university hospitals and agricultural experiment stations here. Note that this classification differs slightly from that used in the Census Bureau’s statistics on government finance.

2. For the University of California system, report the Department of Energy employees at *Other and Unallocable*, code 89, rather than here.

Applicability of Function 18, by Level and Type of Government

Federal	State	Local
No	Yes	Types 1, 2, 3 - Yes<br>Type 4 - No<br>Type 5 - Yes

Code 21 **Federal and State Other Education**

Sector: General Government

Definition: Employees in support of special programs and institutions primarily for:

- Training and education (rather than care) of the blind, deaf, or other handicapped.
- Programs for adult, vocational, or special education that operate outside school systems
- Educational activities not assignable to other education functions.

Includes: State schools for the blind, visually impaired, deaf, or other handicapped; adult education and vocational rehabilitation and education not provided by school systems; technical or vocational-technical schools which award certificates equal to or less than two years of college; overall supervision of and general services to local elementary and secondary schools, public or private; administration of state education activities; administration of state school building authorities.

Excludes: Institutions for blind, deaf, or other handicapped that are primarily for physical rehabilitation and care (report at *Hospitals*, code 36); administration of state institutions of higher education (report at *Other Higher Education*, code 18).

Examples:

- Federal Government – Includes Department of Education and the National Science Foundation, plus parts of the Bureau of Indian Affairs.
- State governments – Includes boards of governors of the state college/university systems, school finance authorities, educational facilities authorities, and commissions on higher education. State institutions in support of education for handicapped, such as the Alabama Institute for Deaf and Blind.

Sub-Categories for Employment Statistics

None.

Special Considerations:

1. For Census Bureau statistics on employment, this function applies only to Federal and state governments.
2. Refer to dependent agency checklists for state or local institutions included at this function.

Applicability of Function 21, by Level and Type of Government

Federal	State	Local
Yes	Yes	No (report at functions 12 or 18 only)

Code 22 **Social Insurance Administration**

Sector: General Government

Definition: Administration of unemployment compensation systems, public employment services, and the Federal Social Security, Medicare, and Railroad Retirement trusts.

Includes: Employees of unemployment compensation, unemployment insurance, and equivalent agencies involved in administering the cooperative Federal-state unemployment compensation system. Includes associated public employment, job services, employment services, and other agencies providing job placement, counseling, veterans readjustment allowances, or related services; and determination of eligibility for disability benefits under Federal Social Security (Old-Age and Survivors Insurance and Disability Insurance) and Medicare (Hospital Insurance and Supplementary Medical Insurance). For the Federal Government, this function also includes administration of Social Security, Medicare, and Railroad Unemployment and Retirement programs.

Excludes: Employees in support of similar services that can be classified at a specific function.

- Administration of public employee retirement, workers compensation, or miscellaneous insurance trusts (report at *Financial Administration*, code 23).
- Activities funded by the Workforce Investment Act - WIA (formerly the Federal Job Training Partnership Act - JTPA), or monies channeled through public employment offices (report at specific function being financed).

Sub-Categories for Employment Statistics

None.

Special Considerations:

1. This function applies to Washington, DC.
2. This function is titled *Employment Security Administration* when applied just to state and local governments.

Applicability of Function 22, by Level and Type of Government

Federal	State	Local
Yes	Yes	No (except Washington, DC)

Code 23 **Financial Administration**
page 1 of 2

Sector: General Government

Definition: Officials and central staff agencies concerned with tax assessment and collection, accounting, auditing, budgeting, purchasing, custody of funds, and other finance activities.

Includes: Employees in the offices of auditor, comptroller, treasurer, office of the finance director and other central accounting, budgeting, information technology, and purchasing offices.

- Tax administration, assessment, billing, and collection, as well as other revenue collection activities like tax sales, tax litigation, and charges of depositories.
- State supervision of local government finances.
- Management of debt and of investments (including that of own utilities).
- Employees engaged in administration of employee-retirement, workers' compensation, and state other insurance trust funds.
- Licensing and tax collection activities of motor vehicle departments, and other distinctive tax collection activities of regulatory agencies.
- Staff of central data processing centers.

Excludes: Employees engaged in internal (non-central) financial management activities of functional agencies (report at function of agency involved); administration of unemployment compensation systems (report at *Social Insurance Administration*, code 22); employees of motor vehicle departments other than licensing (report at *Other and Unallocable*, code 89).

Examples:

- Federal Government – Employees of the Federal Reserve System, Bureau of Engraving and Printing, U.S. Mint, and Internal Revenue Service (Department of the Treasury).
- State governments – Arkansas Information Technology and Arizona State Board of Equalization.

Sub-Categories for Employment Statistics

None.

Special Consideration:

By definition, this function covers central offices and excludes internal financial management activities of functional agencies (report at function of agency involved). Therefore, it cannot apply to specialized government units – special districts (type 4) and school districts (type 5).

Applicability of Function 23, by Level and Type of Government

**Federal**	**State**	**Local**
Yes	Yes	Types 1, 2, 3 - Yes<br>Types 4, 5 - No

Code 24 **Local Fire Protection**

Sector: General Government

Definition: Prevention, avoidance, and suppression of fires and provision of ambulance, medical, rescue, or auxiliary services provided by fire protection agencies.

Includes: Employees of regular fire departments, including financial, technical, and operational support of volunteer fire forces; rescue squads; fire inspection, investigation, and regulation; fire marshals; fire prevention education; fire suppression training; auxiliary services; and these activities IF handled by a fire department: ambulances, emergency medical technicians (EMTs), paramedic squads, and arson investigation.

Excludes: Forest-fire protection and suppression (report at *Natural Resources*, code 59); ambulances, emergency medical technicians, and paramedic squads handled by non-fire departments (report at *Health*, code 32).

Examples: Local governments – Employees of the Fire Department of New York City (a dependent agency of the city of New York).

Sub-Categories for Employment Statistics

Code 024. Firefighters

Code 124. Other Fire Protection Employees – Includes ambulance and medical rescue that operate as a part of the fire department.

Special Considerations:

1. For 2005, this function was renamed *Local Fire Protection.* This is a change in title only, as this function has never applied to the Federal or state governments. State government data are reported at *Other and Unallocable*, code 89, except forest fire protection and suppression as described under the exclusions above.

2. Effective with 1988 data, the treatment of ambulance and emergency medical services was clarified to include it here only if handled by a fire protection agency.

3. Classify volunteer fire fighters remunerated on a “per fire” or other such basis as part-time employees. Unpaid volunteer firefighters are excluded from coverage, by definition.

Applicability of Function 24, by Level and Type of Government

Federal	State	Local
No	No	Types 1, 2, 3, 4 - Yes<br>Type 5 - No

EMPLOYMENT FUNCTIONS

Code 25 **Judicial and Legal**

Sector: General Government

Definition: Courts (criminal and civil) and activities associated with courts, legal services, and legal counseling of indigent or other needy persons.

Includes: Employees of criminal and civil courts of limited and general jurisdiction; appellate courts; juries, court reporters, and law libraries; medical and social service activities of courts (except probation); court activities of sheriff offices (bailiffs or civil functions); registers of wills and other probate activities; legal departments, general counsels, solicitors, prosecuting and district attorneys; attorneys providing government-wide services; public defenders; indigent defense; and employees engaged in child support enforcement.

Excludes: Probation (report at *Other Corrections*, code 05); boards of appeal for zoning, tax assessment, workers' compensation, or other nonjudicial areas adjudication related strictly to administrative rule-making; "judges" which are administrative or executive positions (report at *Central Staff Services*, code 29).

Examples:

- Federal Government – U.S. Supreme Court and activities of the nine judicial circuits of the Federal appeals courts.
- State governments – Supreme Court of California and State Courts of Appeal.
- Local governments – Superior Courts in California, classified as county agencies, except for San Francisco (city).

Sub-Categories for Employment Statistics

None.

Special Consideration:

Exclude from employment data private attorneys on retainer and court-appointed private counsel.

Applicability of Function 25, by Level and Type of Government

Federal	State	Local
Yes	Yes	Types 1, 2, 3 - Yes<br>Types 4, 5 - No

EMPLOYMENT FUNCTIONS

Code 29 **Central Staff Services**

Sector: General Government

Definition: Government-wide executive, legislative, administrative, and staff service agencies other than financial, judicial, legal (which are separate functions).

Includes: Office of the chief executive, mayor, city manager, county administrator; central personnel administration; overall planning and zoning; clerk's office, recorder, and general public reporting; central staff executive and administrative agencies. Also includes legislative activities at the Federal, state and local level.

- U.S. Congress; state legislatures; research and investigative agencies and committees directly responsible to these legislative bodies.
- For local governments, includes city or county council, board of supervisors, and so forth.

Excludes: Exclusions generally involve activities that fit into specific functional categories.

- Planning activities limited to a specific function and internal control or administrative activities of functional agencies (report at function of agency involved)
- Central finance agencies and data processing (report at *Financial Administration*, code 23).
- Clerk of court and other judicial recording activities (report at *Judicial and Legal*, code 25).
- Conduct of elections (report at *Other and Unallocable*, code 89).

For legislative activities, exclude agencies within the legislative branch operating autonomously and having a specific function (e.g., report U.S. Library of Congress at *Libraries*, code 52, and the U.S. Government Accountability Office (GAO) at *Financial Administrative*, code 23).

Examples: See above.

- Federal Government – Includes the U.S. Congress and its staff/committee activities.
- State governments – Include offices of the Governor and Lieutenant Governor, as well as the Secretary of State.
- Local governments- Includes offices of the aldermen, commissioners, mayors, county or city managers, human resources, and zoning.

Sub-Categories for Employment Statistics:

None.

Special Consideration:

For Census Bureau statistics on employment, this function includes legislative employees at the Federal, state, and local level. Thus this function differs slightly from coverage in the Census Bureau's government finance statistics, where it excludes federal and state legislative activity.

Applicability of Function 29, by Level and Type of Government

Federal	State	Local
Yes	Yes	Types 1, 2, 3 - Yes<br>Types 4, 5 - No

EMPLOYMENT FUNCTIONS

Code 32 **Health**

Sector: General Government

Definition: Provision of services for the conservation and improvement of public health, other than hospital care.

Includes: General health activities, categorical health activities and programs, health-related inspections, community health care programs, regulation of air and water quality, rabies and animal control, and ambulance and emergency medical services ONLY IF handled separately from the local fire department. Additional examples are listed below.

Excludes: Employees related to the following:
1. Examination and licensing of related professions – e.g., doctors and nurses (report at *Other and Unallocable*, code 89).

2. Operation or construction of nursing homes (report at *Public Welfare*, code 79).

3. Vocational rehabilitation (report at *Education*, codes 18 and 21).

4. Coroners and crime labs (report at *Police Protection*, code 62).

Examples:

- Federal Government – includes the Food and Drug Administration and the Environmental Protection Agency.

- State governments – includes general health activities, public health administration, laboratories, public education, vital statistics, research, alcohol and drug abuse prevention/rehabilitation and other general health. Includes categorical health activities – control of cancer, TB, socially transmitted diseases, mental illness, etc. and maternal activities under Federal W.I.C. program – Women, Infants, and Children, and child health care. Also includes emissions control activities.

- Local governments
  - Health related inspections – Inspection of restaurants, water supplies, food handlers, nursing homes, agricultural standards or protection of agricultural products from disease.
  - Community health care programs – Community and visiting nurses; immunization programs; out-patient health clinics.
  - Regulation of air and water quality – Sanitary engineering and other environmental activities
  - Animal control – General animal control plus rabies control, abatement of mosquitoes, rodents, and other vermin.

Sub-Categories for Employment Statistics

None.

Special Consideration:

Effective in 1988, the Census Bureau clarified the classification of nursing homes at *Public Welfare* (except inspection of such homes) and of ambulance services at *Health* only if such service is not organized under a fire department.

Applicability of Function 32, by Level and Type of Government

Federal	State	Local
Yes	Yes	Types 1, 2, 3, 4 - Yes<br>Type 5 - No

Code 36 **Hospitals**

Sector: General Government

Definition: Hospital facilities providing in-patient medical care and institutions primarily for care and treatment of handicapped (rather than education) which are directly administered by a government, including those operated by public universities.

Includes: Hospitals include government operated general hospitals providing in-patient medical care and facilities that provide specialized care. Among the latter are:

- Institutions for the custody, treatment, or general care of the mentally insane, or emotionally disturbed.
- TB sanatoria.
- Maternity and children hospitals.
- Orthopedic hospitals.
- Hospitals for chronic diseases.
- Institutions for care and treatment of blind, deaf, developmentally disabled, or other special classes of handicap.
- Hospitals associated with university medical schools (including paid student help).

Excludes: For the Federal Government, exclude veterans hospitals operated by U.S. Department of Veterans Affairs (report at *Federal Own Hospitals - Veterans*, code 37). Most other exceptions involve employment associated with care or treatment under public welfare, or specialized care that falls into another Census Bureau function. The most prominent exclusions are listed below:

- Nursing homes (or other welfare institutions) not directly associated with a public hospital (report at *Public Welfare*, code 79).
- Infirmaries serving particular institutions, like college infirmaries and prison hospitals (report at function involved).
- Hospitals for criminally insane operated by corrections agency (report at *Corrections,* code 05).
- Medical school hospital instructional staff (use *Other Higher Education*, code 18).
- State schools for the blind, visually impaired, deaf, or other handicap (report at *Other Education*, code 21).

Examples:

- State governments – Louisiana State University Health Sciences Center – HCSD.
- Local governments – Hurley Medical Center, Flint, Michigan (a dependent agency of the city government); King County Hospital District, state of Washington (classified as a special district government).

Code 36 **Hospitals**
page 2 of 2

Sub-Categories for Employment Statistics
None.

Special Consideration:
For purposes of dissemination, public employment uses code 40 to report hospital employees and payroll, for most public use files.

Applicability of Function 36, by Level and Type of Government

Federal	State	Local
Yes	Yes	Yes - all types

EMPLOYMENT FUNCTIONS

Code 37 **Federal Own Hospitals - Veterans**

Sector: General Government

Definition: Hospital facilities providing medical care to veterans and institutions primarily for the care and treatment of service-connected disabilities. This function covers Federally-administered and operated hospitals only.

Includes: Comprises hospitals and related medical facilities operated by the U.S. Department of Veterans Affairs (VA). For Employment surveys, include Department of Veterans Affairs outpatient medical care here.

Excludes: Hospitals operated by the Department of Defense (report at *Federal National Defense and International Relations,* code 06).

Examples:
- Federal Government – Bay Pines VA Medical Center, St. Petersburg, Florida (includes inpatient and outpatient treatment).

Sub-Categories for Employment Statistics

None.

Special Consideration:

This function applies solely to the Federal Government; report employees of state-operated veterans hospitals at *Hospitals,* code 36.

Applicability of Function 37, by Level and Type of Government

Federal	State	Local
Yes	No	No

Code 44 **Highways**
page 1 of 2

Sector: General Government

Definition: Maintenance, operation, repair, and construction of highways, streets, roads, alleys, sidewalks, bridges, tunnels, ferry boats, and related structures, including those operated on a toll basis.

Includes: In addition to the general operation or construction of highways, includes employees engaged in the following activities.

- Snow and ice removal and application of salt and sand (including that by sanitation or street cleaning agencies, if identifiable).
- Street or highway lighting and traffic signals.
- Highway and traffic design, and engineering IF handled by public works or highways agency.
- Operations of ferries (toll and nontoll) and drawspans (including toll takers).
- Construction and maintenance of such highway-related items as curbs, gutters, crosswalks, grade separations, trestles, railroad crossings, and storm drains integral to highway projects.
- Operation of garages, administrative buildings, and other facilities of highway agencies.

Excludes: Employees engaged in activities classified more appropriately at specific functions

- Patrol or policing of streets and highways and traffic control activities of police or public safety agencies (report at *Police Protection*, code 62).
- Street cleaning activities (report at *Solid Waste Management*, code 81).
- Local public parking facilities or meters (report at *Other*, code 89).
- Operation of water transport and terminal facilities (use *Sea and Inland Port Facilities*, code 87).
- Roads and walkways within parks and maintained by a park agency (report at *Parks and Recreation*, code 61).

Examples:

- Federal Government – Most employees of the Federal Highway Administration (part of the Department of Transportation), including those involved in Highway Trust Fund grant administration.
- State governments – Employees engaged in oversight, maintenance, and toll operations of the New York State Thruway.
- Local governments – Highway and bridge operations of special district governments such as the Delaware River and Bay Authority and the Port Authority of New York and New Jersey.

Sub-Categories for Employment Statistics

None.

Code 44 **Highways**
page 2 of 2

Special Consideration:

Report employment data for toll highways, toll ferries, and so forth, here. There is no separate Toll Highway function in the Census Bureau's statistics on government employment.

Applicability of Function 44, by Level and Type of Government

Federal	State	Local
Yes	Yes	Types 1, 2, 3, 4 - Yes<br>Type 5 - No

EMPLOYMENT FUNCTIONS

Code 50 **Housing and Community Development**

Sector: General Government

Definition: Construction, operation, and support of housing and redevelopment projects and other activities to promote or aid public and private housing and community development.

Includes:

- Housing – Planning, constructing, furnishing, and operating public housing projects (generally for persons not adequately served by private sector); administration of rent subsidy and related programs (e.g., "Section 8" assistance); housing and mortgage finance agencies; promotion of home ownership; assistance for repair and renovation of existing homes; and programs to encourage private sector housing production.

- Community development – Urban renewal and slum clearance; redevelopment and rehabilitation of substandard or deteriorated facilities and areas; rural redevelopment; and revitalization of commercial areas.

Excludes: Building inspection and enforcement of housing codes or standards (report at *Other and Unallocable*, code 89); employees engaged in providing temporary shelters or housing for the homeless (report at *Public Welfare*, code 79); and construction and maintenance of military housing by the U.S. Department of Defense (report at *National Defense and International Relations*, code 06).

Examples: Local governments – Employees funded by Federal grants for housing code enforcement activity. Minneapolis Public Housing Authority, classified as a special district government serving the Minneapolis, MN area.

12.1.1 Sub-Categories for Employment Statistics

None.

Special Consideration:

The emphasis of this function is on physical plant – its construction, provision, improvement, financing, and the operation of public housing facilities. Activities that directly aid homeowners or renters themselves (e.g., housing expense relief) generally fall under *Public Welfare*, code 79.

Applicability of Function 50, by Level and Type of Government

Federal	State	Local
Yes	Yes	Types 1, 2, 3, 4 - Yes<br>Type 5 - No

Code 52 **Libraries**

Sector: General Government

Definition: Establishment and provision of libraries for use by the general public and the technical support of privately-operated libraries.

Includes: Public libraries, community libraries, consolidated libraries, regional libraries, and their variously-named equivalents; library extension services including bookmobiles; public library (special) districts; U.S. Library of Congress (including Copyright Office); state library commissions and boards; programs to promote, develop, and coordinate library services and facilities.

Excludes: Primary exclusions are specialized libraries associated with an activity defined primarily at another Census Bureau function.

- Law libraries (report at *Judicial and Legal*, code 25).
- Libraries operated by school systems – elementary, secondary, or higher education – primarily for the benefit of students and teachers (report at *Education*, codes 12 and 18).
- Specialized libraries which do not serve the general public, such as a medical library of a university hospital (report at the function involved).

Examples:

- Federal Government – Library of Congress.
- State governments – Hawaii Public Schools Library (see note below).

Sub-Categories for Employment Statistics

None.

Special Consideration:

At the state level, this function applies only to the State of Hawaii.

Applicability of Function 52, by Level and Type of Government

Federal	State	Local
Yes	Yes	Types 1, 2, 3, 4, 5 - Yes

Code 59 **Natural Resources**

Sector: General Government

Definition: Conservation, promotion, and development of natural resources (soil, water, energy, minerals, etc.) and the regulation of industries which develop, utilize, or affect natural resources.

Includes: Employees engaged in irrigation; drainage; flood control; soil conservation and reclamation including prevention of soil erosion; surveying, development, and regulation of water resources; regulation of mineral resources and related industries including land reclamation; wetlands and watershed management and protection; geological surveying and mapping; regulation of gas and oil drilling and production; dam and reservoir safety; public education programs related to the above; fairs; technical assistance to private or other governmental efforts in these areas.

Excludes: Hydroelectric power facilities (report state and local government activities at *Electric Power* utilities, code 92); improvement of waterways, construction and maintenance of canals, and operation of public water transportation facilities (report at *Sea and Inland Port Facilities*, code 87); inspection of public drinking water and activities related to air and water quality or pollution control (report at *Health*, code 32); advertising of state resources (report at *Other and Unallocable*, code 89).

Examples:

- Federal Government – Includes the National Oceanic and Atmospheric Administration (Department of Commerce) and the U.S. Geological Survey (Department of the Interior).
- State and local governments – Includes inspectors of agricultural products and livestock.

Sub-Categories for Employment Statistics

None.

Special Consideration:

This is the only Natural Resources function applicable to the Census Bureau's statistics on government employment. In contrast, there are multiple "Natural Resource" functions applicable to Federal, state, and local governments in the Census Bureau's statistics on government finances.

Applicability of Function 59, by Level and Type of Government

Federal	State	Local
Yes	Yes	Types 1, 2, 3, 4 - Yes<br>Type 5 - No

Code 61 **Parks and Recreation**

Sector: General Government

Definition: Provision and support of recreational and cultural-scientific facilities maintained for the benefit of residents and visitors.

Includes: Golf courses, playgrounds, tennis courts, public beaches, swimming pools, playing fields, parks, camping areas, recreational piers and marinas, etc.; galleries, museums, zoos, and botanical gardens; auditoriums, stadiums, recreational centers, convention centers, and exhibition halls; community music, drama, and celebrations including public support of cultural activities. For the Federal Government, also includes the Smithsonian Institution and National Foundation on the Arts and the Humanities.

Excludes: Recreational and cultural-scientific activities and facilities operated as part of school systems (report at *Education*, codes 12 and 18); marinas operated for commerce rather than recreation (report at *Sea and Inland Port Facilities*, code 87).

Examples:

- Federal Government – Smithsonian Institution and National Foundation on the Arts and the Humanities.
- State governments – State park authorities such as the Virginia State Parks, a division of the Virginia Department of Conservation and Recreation.
- Local governments – Maryland National Capitol Parks and Planning Commission (a joint dependent agency of Montgomery and Prince Georges counties, Maryland).

Sub-Categories for Employment Statistics

None.

Special Considerations:

None.

Applicability of Function 61, by Level and Type of Government

Federal	State	Local
Yes	Yes	Types 1, 2, 3, 4 - Yes<br>Type 5 - No

Code 62 **Police Protection**
page 1 of 2

Sector: General Government

Definition: Employees of general police, sheriff, state police, and other governmental departments that preserve law and order, protect persons and property from illegal acts, and work to prevent, control, investigate, and reduce crime.

Includes: All law enforcement activities of regular police departments, sheriff and constable offices, and state highway patrols, as well as criminal justice planning.

- Criminal investigation, forensic services, crime labs, coroners and medical examiners.
- Temporary "lockups" and transporting criminals.
- Police communications and radios services.

Excludes: Special police forces of non-police agencies (report at function of agency involved):

- For the Federal Government, Postal Service inspectors are reported at *Federal Postal Service,* code 14.
- For state and local governments, report park rangers at *Parks and Recreation*, code 61, fish and game wardens at *Natural Resources*, code 59, campus police at *Higher Education*, code 18, transit police at *Public Mass Transit Systems*, code 94, and so forth. This function also excludes law enforcement employees of legal offices (report at *Judicial and Legal*, code 25); traffic control and engineering performed by non-police agencies (report at *Highways*, code 44); police jails that hold people beyond arraignment (report at *Corrections*, code 05); civil or bailiff activities of sheriff offices (use *Judicial and Legal*, code 25).

Examples:

- Federal Government – Includes the U.S. Department of Justice agencies of the Bureau of Alcohol, Tobacco, Firearms and Explosives (ATF), Federal Bureau of Investigation (FBI), and Drug Enforcement Administration (DEA); the Department of Homeland Security agencies U.S. Immigration and Customs Enforcement, Coast Guard, Transportation Security Administration (TSA), U.S. Secret Service (parts of the Department of Homeland Security), and law enforcement activities of the U.S. Customs and Border Protection.
- State governments – Includes employees of the California Highway Patrol.

Sub-Categories for Employment Statistics

Code 062. Police Officers – Police employees with power of arrest

Code 162. Other Police Protection Employees – Includes school crossing guards (as part-time employees only).

Special Consideration:

Effective with 1988 data, the treatment of traffic control and engineering and liquor control enforcement were clarified to include them here only if handled by a police agency.

Applicability of Function 62, by Level and Type of Government

Federal	State	Local
Yes	Yes	Types 1, 2, 3, 4 - Yes<br>Types 5 - No

Code 79 **Public Welfare**

Sector: General Government

Definition: Employees engaged in all public welfare activities, including those involved in administration of public assistance programs as well as those providing direct assistance.

Includes: Administration of medical and cash assistance, general relief, vendor, and other welfare programs. Maintenance of nursing homes or other institutions for the benefit of veterans or needy persons (contingent upon their financial or medical need). Provision of veterans services, senior citizen and handicapped transportation, services to the homeless, and child services (such as foster care, adoption, day care, nonresidential shelters, and the like). Social workers. Regulation of private welfare institutions and activities. For local governments, vocational rehabilitation for blind and other handicapped, in the form of commercial activity, is reported here rather than at *Education*.

Excludes:

• Employees engaged in programs where benefits are not contingent upon need.

• Public defenders and indigent legal defense (report at *Judicial and Legal*, code 25).

• Health and hospital employees providing care to needy or homeless through government's own hospitals or health agencies (report at appropriate *Health*, code 32, or *Hospitals*, codes 36 and 37, functions).

• Employees of nursing homes directly associated with a public hospital (use *Hospitals*, code 36).

Examples: Federal Government – Includes employees involved in administering Temporary Assistance for Needy Families (TANF).

Sub-Categories for Employment Statistics

None.

Special Consideration:

This is the only Public Welfare function applicable to the Census Bureau's statistics on government employment. In contrast, there are multiple "Public Welfare" functions applicable to Federal, state, and local governments in the Census Bureau's statistics on government finances.

Applicability of Function 79, by Level and Type of Government

Federal	State	Local
Yes	Yes	Types 1, 2, 3, 4 - Yes<br>Type 5 - No

EMPLOYMENT FUNCTIONS

Code 80 **Sewerage**

Sector: General Government

Definition: Provision, maintenance, and operation of sanitary and storm sewer systems and sewage disposal and treatment facilities.

Includes: Employees engaged in the construction or maintenance of sanitary sewer lines; sewer cleaning; lift or pump stations; sewage treatment plants; water pollution control plants; storm sewer and storm drains except when integral parts of highway departments; systems for the collection and disposal of storm runoff.

Excludes: Employees constructing storm drains that are an integral part of highway projects (report at *Regular Highways*, code 44). Provision of drainage for agricultural or other non-street purposes (report at *Natural Resources*, code 59).

Examples:

- State governments – Narragansett Bay Water Quality Management, Massachusetts Water Resources Authority.
- Local governments – Buffalo Sewer Authority (a municipal sewerage department), as well as special district water and sewer districts.

Sub-Categories for Employment Statistics

None.

Special Considerations:

1. For combined water supply and sewer systems, include segregable amounts related to sewage collection and disposal here and *Water Supply* activities at code 91.
2. There is no applicability to the Federal Government employees at this function.

Applicability of Function 80, by Level and Type of Government

Federal	State	Local
No	Yes	Types 1, 2, 3, 4 - Yes<br>Type 5 - No

EMPLOYMENT FUNCTIONS

Code 81 **Solid Waste Management**

Sector: General Government

Definition: Collection, removal, and disposal of garbage, refuse, hazardous, and other solid wastes; and cleaning of streets, alleys, and sidewalks.

Includes: Garbage collection; sanitary landfills; hazardous waste disposal sites; incinerators; pyrolysis facilities; cleanup of toxic chemical spills and dumps; collection and disposal of abandoned vehicles; resource recovery authorities, including those which co-generate electricity or gas as a by-product; recycling centers; cleaning and washing of streets; and collection and disposal of street debris and trash.

Excludes: Snow and ice removal by a sanitation agency (report at *Highways*, code 44).

Examples: Local governments – Employees of landfills and recycling centers, such as the Prince William County Sanitary Landfill, a dependent agency of Prince William County, Virginia.

Sub-Categories for Employment Statistics

None.

Special Considerations:

1. The rationale for including at this function waste or resource recovery facilities that co-generate gas or electricity is that their primary purpose is disposing of wastes.
2. There is no applicability to Federal Government employees at this function.

Applicability of Function 81, by Level and Type of Government

Federal	State	Local
No	Yes	Types 1, 2, 3, 4 - Yes<br>Type 5 - No

Code 87 **Sea and Inland Port Facilities**

Sector: General Government

Definition: Provision, construction, operation, maintenance, and support of public waterways, harbors, docks, wharves, and related marine terminal facilities. Regulation of the water transportation industry.

Includes: Employees engaged in administering commercial port facilities, canals, harbors, and other public waterways, as well as dredging of same. Public docks, piers, wharves, warehouses, cranes, and associated terminal facilities. Regulation and inspection of the commercial water transportation industry. For Federal Government includes waterways navigation activities of the Army Corps of Engineers and the St. Lawrence Seaway.

Excludes: Recreational types of docks and marine facilities – e.g., public marinas devoted to pleasure boaters (report at *Parks and Recreation*, code 61); terminals and dock facilities for public ferries (report *Highways*, code 44).

Examples:
- State governments – Pennsylvania Port Authority or the Port of New Orleans (Louisiana), which are both dependent agencies of their respective state governments.
- Local governments – San Diego Unified Port District or the Port of Seattle (Washington).

Sub-Categories for Employment Statistics
None.

Special Considerations:
1. This category is devoted to commercial water transportation and terminals rather than the provision of water-related activities and facilities for pleasure or recreation.
2. For leased facilities include only the government's own employees and exclude the lessees' contractual employees.
3. For the Federal Government, this function included the former Panama Canal Commission in the historical time series.
4. This function was formerly titled "Water Transport and Terminals."

Applicability of Function 87, by Level and Type of Government

Federal	State	Local
Yes	Yes	Types 1, 2, 3, 4 - Yes<br>Type 5 - No

Code 89 **Other and Unallocable**
page 1 of 2

Sector: General Government

Definition: Employees engaged in activities that are not applicable to other employment functions, or are multi functional.

Includes: TO THE EXTENT NOT ALLOCABLE TO OTHER FUNCTIONS

- State-local military activities (militia, National Guard, armories, civil defense, etc.).
- Central service agencies (e.g., public works, motor pools, communications) other than financial administration and government-wide executive services.
- Administration of multi-functional agencies.
- Economic development.
- Voter registration and elections.
- Job training and employment programs (e.g., Federal Workforce Investment Act).
- Programs for senior citizens not based on need.
- Engineering.
- Emergency (911) dispatching.
- Geographic information systems and mapping services.
- Liquor stores operated by local governments.
- Code Enforcement.
- Employment for functions that apply only to Census Bureau statistics on government finances. These are listed below along with the applicable function code for government expenditure.

Miscellaneous commercial activities (*Miscellaneous Commercial Activities, NEC*, finance code *03).
General Public Buildings (*General Public Buildings*, finance code *31).
Parking Facilities (*Parking Facilities*, finance code *60).
Protective Inspection and Regulation, NEC (*Protective Inspection and Regulation, NEC*, finance code *66).
Federal and State Veterans Services (*Federal and State Veterans' Services*, finance code *85).

Excludes: Comprehensive planning and zoning (report at *Central Staff Services*, code 29).

Sub-Categories for Employment Statistics

None.

Code 89 **Other and Unallocable**
page 2 of 2

Special Considerations:

1. Note that there are functions applicable only to Census Bureau statistics on government finances that are not listed in the "Includes" section above. These almost never involve employment, but rather are intended to cover activities involving large amounts of expenditure (e.g. *General Local Government Support*, code *30, used to classify state financial aid programs for their local governments).
2. In tabular presentations involving employment statistics, "Other and Unallocable" will vary in meaning depending on the level of functional detail in table.

Applicability of Function 89, by Level and Type of Government

Federal	State	Local
Yes	Yes	Types 1, 2, 3, 4 - Yes<br>Type 5 - No

EMPLOYMENT FUNCTIONS

Code 90 **Liquor Stores**

Sector: Liquor Stores

Definition: Establishment and operation of alcoholic beverage distribution facilities and retail outlets owned and operated by state governments.

Includes: Alcoholic beverage monopolies; ABC stores; liquor control boards; state liquor stores.

Excludes: Employees engaged in law enforcement, licensing, and regulation of private liquor outlets carried out in conjunction with liquor store operations (report licensing at *Financial Administration*, code 23, regulation and enforcement at *Other and Unallocable*, code 89, and law enforcement handled by regular police forces at *Police Protection*, code 62).

Examples: State governments – West Virginia Alcohol Beverage Control Administration, which controls the wholesale distribution of alcoholic beverages in that state.

Sub-Categories for Employment Statistics

None.

Special Consideration:

For employment data, applies only to state governments. Report any local government activity at *Other and Unallocable*, code 89.

Applicability of Function 90, by Level and Type of Government

Federal	State	Local
No	Yes	No

EMPLOYMENT FUNCTIONS

Code 91 **Water Supply**

Sector: Utilities

Definition: Operation, maintenance, and construction of public water supply systems, including production, acquisition, and distribution of water to general public or to other public or private utilities, for residential, commercial, and industrial use.

> **Includes:** Employees engaged in operating dams and reservoirs expressly for water supply; purification and filtration plants; pumping stations; aqueducts and transmission systems; water storage tanks; distribution lines and meters; and government operated desalinization plants.
>
> **Excludes:** Private employees of facilities that have been leased to a private company for operational purposes. Activities not directly related to utility operations, such as administration of utility debt or investments (report at *Financial Administration*, code 23).

Sub-Categories for Employment Statistics

None.

Special Considerations:

1. Pertains only to systems owned and operated by a government.
2. For combined water-sewer systems, include segregable employment related to water supply here (report *Sewerage* activities at code 80).

Applicability of Function 91, by Level and Type of Government

Federal	State	Local
No	Yes	Types 1, 2, 3, 4 - Yes<br>Type 5 - No

Code 92 **Electric Power**

Sector: Utilities

Definition: Operation, maintenance, and construction of public electric power systems, including production, acquisition, and distribution of electricity to general public or to other public or private utilities, for residential, commercial, and industrial use.

Includes: Employees engaged in the operation and maintenance of generating stations, power plants, transmission lines, switching stations, pollution abatement equipment, and distribution lines and meters.

Excludes:

- Employees of facilities owned but leased to other governments or persons without financial oversight.
- Activities not directly related to utility operations, such as administration of utility debt or investments (report at *Financial Administration*, code 23).
- Waste or resource recovery facilities which cogenerate electricity as a by-product (report at *Solid Waste Management*, code 81).

Examples: State governments – Power Authority of the State of New York, which operates the hydroelectric generating facility in Niagara County, New York.

Sub-Categories for Employment Statistics

None.

Special Consideration:

Relates only to systems owned and operated by a government, or managed by private contractor where government maintains day-to-day financial oversight (e.g., by directly paying utility employees).

Applicability of Function 92, by Level and Type of Government

Federal	State	Local
No	Yes	Types 1, 2, 3, 4 - Yes<br>Type 5 - No

Code 93 **Gas Supply**

Sector: Utilities

Definition: Operation, maintenance, and construction of public natural gas supply systems, including production, acquisition, and distribution of gas to general public or to other public or private utilities, for residential, commercial, and industrial use.

Includes: Employees engaged in the operation and maintenance of public gas works, liquefaction equipment, pumping stations, gas mains, and service lines and meters.

Excludes:
- Employees of facilities owned but leased to other governments or persons without financial oversight.
- Activities not directly related to utility operations, such as administration of utility debt or investments (report at *Financial Administration*, code 23).
- Waste or resource recovery facilities which cogenerate gas as a by-product (report at *Solid Waste Management*, code 81).

Examples: Local governments – Southeast Alabama Natural Gas District (classified as a special district government and serving southeastern Alabama).

Sub-Categories for Employment Statistics

None.

Special Consideration:

Relates only to systems owned and operated by a government or managed by private contractor when government maintains day-to-day financial oversight (e.g., by directly paying utility employees).

Applicability of Function 93, by Level and Type of Government

Federal	State	Local
No	Yes	Types 1, 2, 3, 4 - Yes<br>Type 5 - No

Code 94 **Public Mass Transit Systems**

Sector: Utilities

Definition: Operation, maintenance, and construction of public mass transit systems, including subways, surface rails, and buses.

Includes: Employees of the following variations of transit systems: rapid transit, subways, surface rail, and street railroad systems, commuter rail lines including light rail trolleys, and bus systems. Includes employees operating and maintaining related stations, tracks, depots, and rail yards. Includes transit police employed directly by the utility.

Excludes:
- Systems solely to transport students (report at *Elementary and Secondary Education*, code 12).
- Ferries (report at *Highways*, code 44).
- Systems exclusively for handicapped or senior citizens (report at *Public Welfare*, code 79).
- Employees of systems owned but operated under private contract without financial oversight.
- Activities not directly related to utility operation, such as administration of utility debt (report at *Financial Administration*, code 23).

Examples:
- State governments – Long Island Railroad, a component of the Metropolitan Transportation Authority serving the New York City area, which is a dependent agency of New York State.
- Local governments – Washington Metropolitan Area Transit Authority, a special district government serving Washington, DC and parts of Maryland and Virginia.

Sub-Categories for Employment Statistics

None.

Special Consideration:

Relates only to systems owned and operated by a government or managed by private contractor when government maintains day-to-day financial oversight (e.g., by directly paying utility employees).

Applicability of Function 94, by Level and Type of Government

Federal	State	Local
No	Yes	Types 1, 2, 3, 4 - Yes<br>Type 5 - No

12.4 Tables

There are two tables associated with this chapter. Table 12.1, Functional Categories Applicable to Employment Data, lists all the functions for classifying public employment statistics, arranged by the groups used for their publication. The table also shows the related function(s) that apply to Census Bureau statistics on government finance, including multiple functions where applicable. However, the list of finance functions is not designed to be complete for that survey (see Chapter 5).

Table 12.2 is Applicability of Employment Function Codes, by Level and Type of Government. This is an important table for processing and for users, since there are restrictions on some codes. Where restricted, there will be no separate public employment statistics for a particular level or type of local government.

Table 12.1<br>**Functional Categories Applicable to Employment Data** – page 1of 2			
This table lists all the functions for classifying public employment statistics, arranged by the groups used for their publication. It contains the relevant function(s) that apply to finance statistics, including multiples where applicable. However, the list of finance functions is not designed to be complete – there are other finance functions with no corresponding employment code (see Chapter 5 for complete descriptions). Selected sub-functions apply only to employment statistics.			
**Function code(s)**	**Item**	**Applies to**	
		**Relevant finance functions**	**Employment sub-codes only**
	GENERAL GOVERNMENT		
	Selected Federal Programs:		
06	National Defense and International Relations	06	
14	Federal Postal Service	14	
02	Federal Space Research and Technology	02	
	Education Services:		
	Education –		
18	Higher Education	16,18,19,21	
	Instructional		018
	Other		016
12	Elementary and Secondary Education	12	
	School Instructional		012
	Other School Employees:		112
	Administrative and Clerical		212[1]
	Operations and Maintenance		312[1]
	Cafeteria		412[1]
	Bus Transportation		512[1]
	Health and Recreation		612[1]
	Student Employees (paid)		712[1]
	Unallocable		812[1]
21	Other Education	21	
52	Libraries	52	
	Social Services and Income Maintenance:		
79	Public Welfare	67,68,74,77,79	
36, 37	Hospitals	36, 37	
32	Health	28,32	
22	Social Insurance Administration	22	
	Transportation:		
44	Highways	44,45	
01	Air Transportation (Airports)	01	
87	Sea and Inland Port Facilities	87	

[1]Prior to 2001, data were collected separately for the items listed under “All Other.” These are no longer collected separately.

Table 12.1<br>**Functional Categories Applicable to Employment Data –** page 2 of 2			
**Function code(s)**	**Item**	**Applies to**	
		**Relevant finance functions**	**Employment sub-codes only**
	Public Safety:		
62	Police Protection	62	
	Police with Arrest Powers		062
	Other Police		162
24	Fire Protection	24	
	Firefighters		024
	Other		124
05	Corrections	04,05	
	Environment and Housing:		
59	Natural Resources	51,53,55,56,57,58,59	
61	Parks and Recreation	61	
50	Housing and Community Development	50	
	Sanitation –		
80	Sewerage	80	
81	Solid Waste Management	81	
	Governmental Administration:		
23	Financial Administration	23	
25	Judicial and Legal	25	
29	Central Staff Services (including Legislative)	26,29	
	General Government, NEC:		
89	Other and Unallocable (functions not listed elsewhere)	03,31,60,66,85,89	
	LIQUOR STORES **(State Governments Only)**		
90	Liquor Stores	90	
	UTILITIES		
91	Water Supply	91	
92	Electric Power	92	
93	Gas Supply	93	
94	Public Mass Transit Systems	94	

Table 12.2
Applicability of Employment Function Codes, by Level and Type of Government – page 1 of 2

Category	Code	Federal	State	Local Governments - By Type				
				County	Municipal	Township	Special district	School district
Air Transportation	01	Valid	Valid	Valid	Valid	Valid	Valid	X
Federal Space Research	02	Valid	X	X	X	X	X	X
Corrections	05	Valid	Valid	Valid	Valid	Valid	X	X
Federal National Defense	06	Valid	X	X	X	X	X	X
Elementary and Secondary Education	12	X	Valid	Valid	Valid	Valid	Valid	Valid
Federal Postal Service	14	Valid	X	X	X	X	X	X
Higher Education	18	X	Valid	Valid	Valid	Valid	X	Valid
Federal and State Other Education	21	Valid	Valid	X	X	X	X	X
Social Insurance Administration[1]	22	Valid	Valid	X	X[1]	X	X	X
Financial Administration	23	Valid	Valid	Valid	Valid	Valid	X	X
Local Fire Protection	24	X	X	Valid	Valid	Valid	Valid	X
Judicial and Legal	25	Valid	Valid	Valid	Valid	Valid	X	X
Central Staff Services	29	Valid	Valid	Valid	Valid	Valid	X	X
Health	32	Valid	Valid	Valid	Valid	Valid	Valid	X
Hospitals[2]	36	Valid	Valid	Valid	Valid	Valid	Valid	Valid
Federal Veterans Hospitals	37	Valid	X	X	X	X	X	X
Highways	44	Valid	Valid	Valid	Valid	Valid	Valid	X
Housing and Community Development	50	Valid	Valid	Valid	Valid	Valid	Valid	X
Libraries[3]	52	Valid	Valid[3]	Valid	Valid	Valid	Valid	Valid
Natural Resources	59	Valid	Valid	Valid	Valid	Valid	Valid	X
Parks and Recreation	61	Valid	Valid	Valid	Valid	Valid	Valid	X
Police Protection	62	Valid	Valid	Valid	Valid	Valid	Valid	X

Table 12.2

Applicability of Employment Function Codes, by Level and Type of Government – page 2 of 2

Category	Code	Federal	State	Local Governments - By Type				
				**County**	**Municipal**	**Township**	**Special district**	**School district**
Public Welfare	79	Valid	Valid	Valid	Valid	Valid	Valid	X
Sewerage	80	X	Valid	Valid	Valid	Valid	Valid	X
Solid Waste Management	81	X	Valid	Valid	Valid	Valid	Valid	X
Sea and Inland Port Facilities	87	Valid	Valid	Valid	Valid	Valid	Valid	X
Other and Unallocable	89	Valid	Valid	Valid	Valid	Valid	Valid	X
Liquor Stores	90	X	Valid	X	X	X	X	X
Water Utilities	91	X	Valid	Valid	Valid	Valid	Valid	X
Electric Utilities	92	X	Valid	Valid	Valid	Valid	Valid	X
Gas Utilities	93	X	Valid	Valid	Valid	Valid	Valid	X
Transit Utilities	94	X	Valid	Valid	Valid	Valid	Valid	X

Key:

X Not a valid code for the level or type of government indicated (there are no statistics).

Valid Applicable code for the level or type of government indicated (statistics are collected and reported).

Notes:

[1]Applies to Washington, DC only for local governments.

[2]Public use files containing employment statistics also can use code 40 for this function.

[3]Applies to the state of Hawaii, only.

PART 4: APPENDICES

This edition of the *Classification Manual* contains three appendices that were not found in previous editions. They were developed to provide useful reference information to Census Bureau analysts and users alike.

Appendix 1 contains explanations about modifications that have taken effect since 1988, when the last edition of the *Classification Manual* was released. All modifications are grouped into one of three general categories. The first general category describes modifications to the structure of the *Classification Manual* itself. The second category of modifications explained in Appendix 1 are revisions made to the public employment statistics program, including statistical classifications, definitions, and methodology for calculating selected types of statistics.

The third general category of modifications described in Appendix 1 covers revisions to the government finance statistics program. These include definitions, classification categories and codes, and a lengthy explanation of major changes to the statistical program for public employee retirement system finances. Many of the revisions to the government finance program were the result of the major redesign that took place for the 2005 survey year.

Appendix 1 also contains two additional tables not found in the main chapters of the *Classification Manual*. Both tables contain detailed information on historical revisions made to specific government finance statistical classification categories (one for cash and security holdings and one for public employee retirement systems).

Appendix 2 consists of tables that appeared throughout the *Classification Manual*, but were separated by chapter. All these tables have a common theme, namely that they contain the detailed finance and employment codes and show whether each code is valid, by level and type of government. These are important references for analysts and users working with the Census Bureau's data, and hence are consolidated in Appendix 2 for ease of reference.

APPENDIX 1. Modifications Effective with this Edition of the Classification Manual

This edition of the *Government Finance and Employment Classification Manual* became effective with the 2005 surveys of government finance and public employment. This edition represents a major change in the structure of the *Classification Manual* itself, compared to the previous edition (1988). This edition also contains several significant revisions to the classification system, especially for the statistical programs covering government finances. This appendix consolidates these revisions for ease of reference and offers explanations for them.

Appendix 1.1 Revisions to Presentation and Format

This edition of the *Classification Manual* contains four major modifications to the presentation of the statistical classification categories. First, it is separated into distinct parts for the two major survey areas of government finance and public employment. Second, the surveys covering the finances of public employee retirement systems were presented separately in Chapter 8, within Part 2 (Government Finances). Third, a number of classification topics about government organization were added to Part 1, Chapter 1. Fourth, this edition contains the full array of detailed tables showing the validity (applicability) of every classification code and coding option, for both the government finance and public employment surveys. Each of these modifications is described below.

Appendix 1.1.1 Separate Explanations of Government Finance and Public Employment

This edition of the *Classification Manual* separated the definitions and categories applicable to the two major surveys – government finance and public employment. Thus, Part 2 (chapters 3 through 10) contains classification details about the government finance surveys, while Part 3 (chapters 11 and 12) covers the public employment survey. Some classification topics are repeated in both parts, but from a different perspective that applies to either finance or employment statistics.

Similarly, all "functional" categories are described twice, once in Chapter 5 pertaining to government expenditure and once in Chapter 12 pertaining to public employment. There are two differences between functional categories for government finance versus public employment. First, there are significant differences in the number of functional categories used in the two survey areas, with more functional categories applying to the government finance survey than to public employment. Second, there are subtle differences in some definitions for functional categories that are used in both surveys. For example, the functional category *Highways* (code 44) in the public employment survey area includes measures of employees engaged in toll highway activity, which is not the case for government finance statistics. As a result, Governments Division statisticians decided that classification systems used in the two survey areas should be presented separately.

Appendix 1.1.2 Separate Presentation of Classification Categories for Public Employee Retirement Systems

Chapter 8 of this edition is dedicated to the classification categories used for financial statistics on public employee retirement systems. Previous editions of this document did not present this topic separately.

Governments Division statisticians thought this separate presentation would be useful for two reasons. First, the topic is extremely important. Public employee retirement systems comprise a major financial activity for governments. The financial assets of these systems represent the majority

of government cash and security holdings nationwide and the finances of these systems have profound implications for the long-term fiscal condition of state and local governments.

Second, the major movements that occurred in the financial markets since the 1988 edition of this *Classification Manual* attest to the importance of accurate statistics on this aspect of government activity. The 1990s saw tremendous growth in the value of equities and most retirement systems invested heavily in them. The 2000-01 recession had a profound impact on the value of these investments. Both factors influenced the method by which retirement systems (and governments in general) assigned values to their assets, and especially their equity holdings. The latter was greatly influenced by the government-wide effort to standardize reporting and accounting practices, as best exemplified by the rising importance of the Governmental Accounting Standards Board, GASB (as noted in several places throughout this edition).

Consequently, Chapter 8 contains a separate description of the classification system used for public employee retirement systems. Additional information about revisions to the classification system for this government activity are noted below in Appendix 1.5.

<u>Appendix 1.1.3 Introduction of Government Organization Topics</u>

The third modification to the presentation used for this edition of the *Classification Manual* was the introduction of topics pertaining to government organization and structure. These are found in Chapter 1, The Basic Unit of Reporting – The Government Entity. Specific information added included an explanation of statistical aggregation and tabulation methodology, an explanation of the Governments Integrated Directory, and explanations of the following government reference coding applications:

- Government Unit Code (including historical coding patterns)
- Government Function Codes
- Government Activity Codes
- School Level Codes

Most of the coding topics are "internal" to the processing methodologies used by the Governments Division. As such, these topics might be of little use to outside users of this *Classification Manual*. Nevertheless, the addition of these topics will assist analysts in understanding the overall classification system and how statistics are processed within the system. Prior to this edition, these topics could be found primarily in documents internal to the Governments Division.

<u>Appendix 1.1.4 Addition of Valid Code Tables</u>

Appendix 2 contains all of the "valid codes tables" for the two survey areas of government finance and public employment. These tables are extremely useful reference tools for the statisticians and analysts involved in the Census Bureau surveys on government finance and employment. They represent code validity by level and type of government. In this sense, they convey the statistical categories for which data can be reported (or can exist). Prior to this edition, these tables were summarized in the *Classification Manual*, but with the precise parameters contained only in documents internal to Governments Division processing.

These tables will be maintained and updated in the on-line version of this *Classification Manual*. Changes generally are very limited from one survey year to the next, but even a single change can impact processing for an individual unit of government, for estimation, and for tabulation. Hence the inclusion of these detailed, but necessary, tables is an important component. The tables are presented

in each chapter, but also are repeated in Appendix 2 for ease of reference.

Appendix 1.2 Revisions to Public Employment Statistical Classifications

One significant change to methodology took effect since the last edition of this *Classification Manual* was released. This change involved the base reporting period for measuring employment and payrolls, which was changed from October to March. This change became effective with the 1997 Census of Governments. As a result, there was no Annual Survey of Public Employment for 1996 (which would have covered the October 1996 employment and payroll statistics).

There were no major changes to the public employment classification system for this edition of the *Classification Manual*. The number of functional categories used to classify employment and payroll statistics, as well as their definitions, remained unchanged from 1988 through the release of this edition.

One minor clarification was introduced for function code 37, Own Hospitals - Veterans. This function applies to Federal Government statistics only. The clarification was that this function would include employees and payrolls for clinic and outpatient medical care. Most Veterans Affairs facilities now offer such services and distinguishing them from inpatient care is virtually impossible.

Appendix 1.3 Summary of Revisions to Government Finance Classifications

This edition of the *Classification Manual* reflects three areas of change that were applied to the statistical program for government finances. The first consisted of general changes in definitions and processing that were applied post-1988. These are the normal types of changes made to any statistical program, and for this program they reflect the need to keep up-to-date with financial reporting practices of governments, as well as changes to government structure and organization among the states and for the Federal Government. The second area consisted of major changes to the statistical reporting used in the surveys of public employee retirement systems. Most of these were effective with the 2002 Census of Governments, although some additional modifications have taken place since. The third area consisted of the 2005 redesign of the government finance statistical program, which resulted in major changes to the internal applicability of finance variables (codes) by level and type of government, and to the elimination of several classification categories.

These revisions are explained in the following sections, Appendices 1.4 thorough 1.6.

Appendix 1.4 Revisions to Definitions

- Revenue: Taxes on public utilities (*Public Utilities*, codes T15 and T27) – the definition was expanded to reflect the taxation of cellular telephone and internet service providers.

- Revenue: *Individual Income Tax and Corporate Net Income Tax* (codes T40 and T41) – the reference to historical data for *Individual Income Taxes*, code T40, was expanded to note that the category includes combined individual and corporation income taxes where proceeds could not be separated in older, historical data.

- Revenue: *Miscellaneous* (code U99) – the definition was modified to reflect the exclusion of earnings from dividends, for state governments. The latter was added to the classification system as a separate category for state governments, effective 2005.

- Expenditure: *Miscellaneous Commercial Activities* (code *03) – the definition was clarified by

noting specific exclusion of those activities that qualify for classification as a social insurance trust system.

- Expenditure: *Elementary and Secondary Education* (code *12) – the definition was expanded to cover charter schools that have been classified as "governmental" (public) by the Census Bureau.

- Expenditure: *Public Utilities, Water Supply* (codes *91) – the definition was expanded to include desalinization plants.

- Expenditure: *Public Utilities, Gas Supply* (codes *93) – the definition was expanded to include liquefied petroleum gas terminals.

- Cash and Security Holdings: The definition for Lotteries cash and securities holdings was clarified to be more explicit about excluding prize monies held pending disbursement.

- Cash and Security Holdings: Valuation – the definition of all cash and security holdings was modified to reflect that assets are to be reported at market value. This methodology was effective with the 2002 Census of Governments for selected assets of public employee retirement systems (as explained in Appendix 1.5 following), and effective with the 2005 government finance surveys for all other types of cash and security holdings.

- Social Insurance Trusts, *Federal Veterans' Life Insurance* – the references in the definition were updated to reflect name changes and additional programs.

- Exhibit codes CGE and CGR – these were added to the *Classification Manual* for the first time. They had been added to the classification system for the 1999 survey year. The categories were re-titled as follows: CGR became *Tobacco Lawsuit Settlement - Receipts*, and CGE became *Tobacco Lawsuit Settlement - Expenses*.

<u>Appendix 1.5 Revisions to Classification Categories and Codes for Statistics on Public Employee Retirement Systems</u>

Most of these changes were effective with the 2002 Census of Governments. They were made after research and consultation with participating respondents (the retirement systems themselves), as well as major users (the Bureau of Economic Analysis and the Federal Reserve Board).

- Market Value of Cash and Security Holdings – The method for measuring the value of certain cash and security holdings was modified. Effective 2002, the following assets were to be reported at market value only, as carried on the books of the reporting systems. Note that some of the codes were changed, since newly defined categories were significantly different in definition from the previous categories.

  Total Corporate Bonds (code Z77), consisting of:
  - *Corporate Bonds, Federally-Sponsored Agencies* (code Z62)
  - *Corporate Bonds, Other* (code Z63)

  Corporate Stocks (code Z78)
  Foreign and International Securities (code Z70)

  Prior to 2002, these categories were reported at book value only (the cost of the asset when it

was purchased). The appropriate codes used prior to 2002 (but eliminated and replaced by the codes above) are noted below:

Corporate Stocks (code X41)
Corporate Bonds (code X40)
Federally-Sponsored Agency Securities (code Z67)

Foreign and International Securities were reported in the category for Other Securities (exhibit code Z69 and also included in regular code X44) from 1997 to 2002. Prior to 1997, these were reported (at book value) at exhibit code Z83.

• Governments Division added a separate category for *State Government – Other Dividends* (code U21). These are treated differently from interest earnings in many economic accounts, and hence there was a need to identify them separately.

• Two little-used categories of public employee retirement system transactions were eliminated effective 2002:
Z97 - Receipts for Transmittal to Federal Social Security System
Z92 - Amounts Transmitted to Federal Social Security System

• *Earnings on Investments* (code X08) underwent significant changes, reflecting both definition and methodology. This ensured internal consistency in the annual surveys, at both the unit level (the individual retirement system) and for any tabulations.

As noted in Chapter 8, the definition was modified to read as follows:

***
Regular Code X08 Earnings on Investments

Definition: Total earnings on investments, net of losses, as summed from all the earnings codes defined below. Includes interest earnings, dividends, recorded profits on investment transactions (minus any recorded losses), rentals; and other earnings on investments. Includes unrealized gains (and losses), as reported for accounting purposes.

Excludes: Rental of property owned by a retirement system and leased to its parent government, and amortization of discounts (nonrevenues).

Special Considerations:
1. This is a calculated statistic. It is the sum of the following six exhibit codes.
+Z71 Interest Earnings
+Z72 Dividend Earnings
+Z73 Other Investment Earnings
+Z96 Gains on Investments, (Realized and Unrealized)
- Z91 Losses on Investments (Realized and Unrealized)
+Z98 Rentals from State Government
2. For Census Bureau Regular Statistics on government finance, Z98 is excluded from calculation of code X08, *Earnings on Investments*. Such receipts reflect an intragovernmental transactions and thus cannot be included as “revenue” for a government.
3. Previous *Classification Manuals* separated earnings on investments between interest income (code X08) and other types of investment earnings (X09). However, in practice this distinction was not made on the survey schedules themselves. Consequently, these two codes were

consolidated effective with fiscal year 1990 data.
***

The primary change was that earnings on investments came to reflect both realized and unrealized gains/losses, owing to the change in definition for those two categories. Prior to 2002, the definition noted only realized gains and realized losses (X14).

• *Losses on the Sale of Investments* (code Z91) and *Gains on the Sale of Investments* (code Z96) variables were both moved to revenue codes. Only one is used, depending on whether the system experienced gains or losses. In either case, the amount is netted from the revenue code X08, *Earnings on Investments,* as noted above. More importantly, the title of these categories had to be changed to reflect the post-2002 methodology used to establish their value. That methodology called for measuring both realized gains/losses and unrealized gains/losses. Realized gains/losses applied to investments that were disposed of (sold) during the fiscal year. For all other investments, the change in market value, or the unrealized gains/losses, and this became a reported amount as well and was combined with the realized gains/losses. The new category was titled *Net Gains or Losses on Investments.*

• New exhibit codes for benefit payments were added. These were added effective 2005 and represent a subset of the code X11, which was re-titled to be *Total Benefit Payments.*
Z13 *Retirement Benefits*
Z14 *Disability Benefits*
Z15 *Survivor Benefits*
Z16 *Other Benefits*

Many of the coding changes for public employee retirement systems are summarized in Appendix Table 1.2.

Appendix 1.6 Revisions from the 2005 Redesign of the Government Finance Program

The 2005 redesign of the government finance program resulted in many revisions to coding applicability. The primary purpose of the redesign was to bring the annual survey into compliance with new and stricter statistical standards for Federal government sample surveys. The statistical standards impacted data collection and estimation methodology. In summary, the resulting methodological changes resulted in many classification categories being added to the local government component of the survey. Prior to 2005, many categories had been restricted to Federal and state governments only, or to "large local governments" (as defined by the Governments Division). As such, estimates of U.S. totals of government finances for these classification categories did not represent true aggregates, reflecting the financial transactions of all levels of government.

There were three other objectives for the 2005 redesign.

1. To simplify the variable set being collected, with the goals of reducing processing time and redesigning the survey data collection instruments (print and electronic).

2. To revise the variables set as needed so that it reflected current government sector financial reporting standards and practices.

3.To update and clarify the definitions, which had been in place since 1988.

The sections that follow (Appendix 1.6.1 to Appendix 1.6.7) explain the revisions emanating from the 2005 redesign.

Appendix 1.6.1 Changes to Object Codes for Expenditure Statistics

There were two major changes to the object coding used for government finance expenditure statistics. These are explained below.

Appendix 1.6.1.1 Assistance and Subsidies. Effective with this edition of the *Classification Manual*, the object code for all "assistance and subsidies" expenditures was changed from "E" and "I" to "J." This was made to avoid duplication in the use of object codes "I" and "E." Prior to this change:

- Object code "I" represented two types of expenditures – interest on debt for all three levels of government, plus assistance and subsidies for Federal Government expenditure statistics. However, since "I89" was the code for interest on general debt, the pre-2005 Federal Government object code for "Other and Unallocable, Assistance and Subsidies" was "J89."

- Object code "E" represented either current operations or assistance and subsidies, for state and local government expenditure statistics. The distinction between the two types of expenditures was based on the function code only, which was confusing and unique within the overall classification system.

Using the "J" object code to represent "assistance and subsidies" for all levels and types of governments was a major simplification to the classification system. However, it is noted that the Governments Division has not applied this object code change to historical publications and data files. Hence all pre-2005 statistics will contain the object code of "I" for Federal Government assistance and subsidies (for all functions except *89, where J89 was used)), and the use of object code "E" for state and local government assistance and subsides. See Chapter 5 for additional information.

Appendix 1.6.1.2 Elimination of Selected Intergovernmental Object Codes

Two intergovernmental object codes were eliminated from the classification system, effective with the 2005 surveys on government finance. Both were eliminated for the same reasons, namely the increasing difficulty of obtaining the data and the need to simplify the overall classification system. The latter also involved a case of limited applicability for these two codes, which had been restricted to state governments only.

Object code "N" was eliminated. This represented intergovernmental expenditure payments to general purpose local governments (counties, municipalities, and townships) from state governments. Object code "R" also was eliminated. It represented intergovernmental expenditure payments from state governments to special district governments. In both cases, any payments previously code at "N" or "R" are reported at object code "M," effective with data for the 2005 surveys. For comparative purposes when using historical statistics, pre-2005 data under object codes "M" plus "N" plus 'R" will compare to the data coded at M only, for 2005 and out years. The definition of object code M remains unchanged, however. It reflects intergovernmental payments to local governments not elsewhere classified, or "NEC."

Appendix 1.6.1.3 Revisions to Object Code K

Object code K is a limited use code that represents expenditure of the purchase of equipment. It represents a subset of object code G – capital outlay other than construction. Prior to 2005, this object codes was used for statistics of the Federal and state governments, plus selected large local governments. The latter condition was corrected by the 2005 redesign, with the result that this code was removed from the valid code set for local governments. Effective 2005, object code K applies only to statistics for the Federal and state governments.

Appendix 1.6.2 Changes for Liquor and Lotteries

The 2005 redesign of the Census Bureau's government finance statistics impacted the liquor stores and lottery classifications. Specifically, codes for lottery regular finance statistics were made valid for all local governments. Prior to 2005, no regular lottery statistics could be used for local governments, with the exception of Washington, DC. Lottery system exhibit statistics remained valid only for state governments and Washington, DC, however.

Regular statistics for liquor stores apply to state and local general purpose governments (some of which administered liquor store operations). Even with the 2005 redesign, however, exhibit and derived statistics for liquor store operations remained limited to state governments. The decision to retain this limitation was based on several factors. First, as of 2005 there were only 430 locally-administered liquor store operations. Secondly, the Census Bureau's annual government finance sample survey is designed to yield statistical estimates for regular statistics only.[1] Since exhibit and derived statistics cause additional data collection and processing burdens but would not be tabulated for local governments (the sample-based portion of the Census Bureau's annual survey of government finances), it was decided not to burden the small number of local governments involved for additional information on the survey questionnaires. Third, prior to the 2005 redesign, liquor store exhibits applied only to state governments, so the post 2005 classification system was left unchanged to maintain historical continuity.

Appendix 1.6.3 Revenue Classification Category Revisions

The revenue codes below were expanded to cover all local general purpose governments (types 1, 2, and 3), in addition to state governments, Washington, DC, and large cites and counties. In all cases, the codes do not apply to school district governments, by definition.

- Natural Resources, Intergovernmental Revenue from the Federal Government (code B59) – data were reported at B89 prior to 2005 (for most local general purpose governments).

- Airports, Intergovernmental Revenue from the Federal Government (code B01) – data were reported at B89 prior to 2005 (for most local general purpose governments).

The revenue codes below were expanded to cover all local governments. However, definitions restrict some applicability as noted, mostly for school districts, which cannot operate or provide the services.

---

[1]An exception exists for the annual survey of public employee retirement systems, which is sample based but does yield estimates of many exhibit statistics. However, none of the latter are used in the broader, annual survey of government finances covering parent governments and all their dependent agencies.

• All tax revenue (codes T**) – without restriction.

• Current charges (various codes) – prior to 2005, a number of current charges categories applied only to state and general purpose local governments. Effective 2005, they became applicable to all local governments. This change affected the following "current charges" categories:

- *Miscellaneous Commercial Activities*, code A03 (not valid for school districts)
- *Higher Education Auxiliary Enterprises*, code A16 (previously state governments only)
- *Other Higher Education Charges*, code A18
- *Public Hospitals*, code A36
- *Toll Highways*, code A45 (not valid for school districts)
- *Other Natural Resources*, code A59 (not valid for school districts)

• Miscellaneous Revenue, *Special Assessments,* code U01 (prior to 2005, did not apply to school district governments)

• *Fines and Forfeits*, code U30 (prior to 2005, did not apply to all local governments)

• Royalties, code U41 (prior to 2005, did not apply to local governments)

• *Donations from Private Sources*, code U50 (prior to 2005, did not apply to local governments)

• *Net Lottery Revenue*, code U95, (prior to 2005, this did not apply to local governments – with exception for Washington, DC).

The revenue codes below were expanded to cover state governments.

• *Current Charges, Parking Facilities*, code A60 – data were reported at A89 prior to 2005.

The revenue codes below were eliminated altogether, effective 2005.

• *Current Charges, Agriculture*, code A54 – report at code A59 instead.

• *Intergovernmental Revenue from Federal Government, Agriculture,* code B54 – report at code B59 instead.

The revenue codes below were added as new to the classification system, effective 2005.

• *State Government –Other Dividends,* code U21 – this code applies to state governments only, for which dividends previously were reported at *Miscellaneous General Revenue, NEC*, code U99, except for public employee retirement systems, which had a separate code for this item (itself effective in 2002). Other social insurance trust systems use a separate category for dividends, namely "earnings on investments." Finally, it is noted that for local governments and their dependent agencies (except for public employee retirement systems) dividends continue to be classified at code U99.

The revenue codes below were consolidated, coverage was expanded, and the definition was modified.

- Sale of Property, Housing and Community Development, code U10, and *Sale of property, Other,* code U11 – code U10 was eliminated. All proceeds from the sale of sale of property are now reported at code U11 only. Also, code U11 was expanded to include school districts.

Other revenue code changes effective with 2005 are noted below.

- Revenue: Education charges (code A21) – the category was re-titled to *Federal and State Education Charges NEC*, to reflect its limited applicability by level of government.

- Current Charges, Forestry (code A56) – this was re-titled to *Federal and State Forestry* to reflect its limited applicability.

Appendix 1.6.4 Expenditure Classification Category Revisions

The revisions to expenditure codes in the 2005 redesign of the government finance program consisted primarily of expanding the categories to all levels and types of government. However, several categories that were not expanded were instead redefined, or re-titled, to limit them explicitly by level of government.

The expenditure codes below were expanded to cover all local governments, in theory. However, definitions restrict applicability as noted, mostly for school districts, which cannot operate or provide the services. Hence these codes are shown as invalid for school district governments in the valid codes tables for this edition of the *Classification Manual*.

- Miscellaneous commercial activities (E03, F03, and G03) – expanded to all local governments, although school districts do not perform the functions. Prior to 2005, this function applied only to local general purpose governments.

- Correctional institutions (E04, F04, G04, L04, M04) – expanded to all local governments, although school districts do not perform the functions. Prior to 2005, this function applied only to Federal and state governments. Also, object codes “M” and “L” were added for this function.

- Other corrections (E05, F05, G05, L05, M05) – expanded to include special district governments, in addition to state and local general purpose governments. School districts do not perform the function.

- Elementary and secondary education (L12) – expanded to include special district governments.

- Higher education auxiliary enterprises (E16, F16, G16) – expanded to all local governments, although special district governments do not perform the function, by definition. Prior to 2005, this function applied only to state governments.

- Toll highways (E45, F45, G45) – expanded to include special district governments, in addition to state and local general purpose governments. School districts do not perform the function.

- Police protection (E62, F62, G62, L62, M62) – expanded to include special district governments, in addition to state and local general purpose governments. School districts do not perform the function, although consideration is being given to expanding the applicability to schools for the future.

The expenditure codes below were expanded to include state governments.

- Parking facilities (E60, F60, G60, K60, M60) – prior to 2005, these codes were for local governments only. State expenditure, it any, was reported at function code 89.

The expenditure codes below were consolidated and redefined.

- Own hospitals (code *36) and Other hospitals (code *38) – expenditure function 38 was eliminated and all transactions consolidated into function 36, which was redefined and re-titled to reflect "Hospitals" rather than "Own hospitals." Furthermore, the newly redefined function became valid for all types of local governments. As a result, new codes L36 and M36 were created, and the following codes eliminated: E38, F38, G38, K38, L38, and M38.

- Veterans' bonuses (code *84) and Other veterans services (code *85) – expenditure function *84 is eliminated and combined with expenditure function *85, which is now titled *Federal and State Veterans Services*. Note that this function also is limited in applicability to Federal and state governments only. Code E84 is eliminated and code J85 is added, effectively replacing E84.

The expenditure codes below were eliminated altogether, effective 2005.

- Private transit subsidies (E47) – expenditures, if any, now are reported at functions 89 or 94. This code previously applied to state and local governments. The associated exhibit code of Z61 (subsidies transferred to own transit system) also was eliminated.

- Other agriculture (E54, I54, F54, G54, K54, L54, M54) – this function applied to Federal and state governments only. Expenditures, if any, are to be reported at function code 59, *Other Natural Resources.*

The expenditure codes below are redefined or re-titled only.

- Fish and game (codes E55, F55, G55, K55, M55) – the title of this function was changed to *State Fish and Game* to reflect its limited applicability.

- *State government scholarships and Other Subsidies (Education),* code J19 – the title of this function was changed to reflect its restricted applicability to state governments only.

- Other Education (codes E21, F21, G21, K21, J21, L21, M21) – the title of this function was changed to reflect its limited applicability to the Federal and state governments only. The new title is *Federal and State Other Education.*

- Fire protection (codes E24, F24, G24, and M24) – the title of this function was changed to reflect its limited applicability, for local governments only. The new title is *Local Fire Protection*.

- Other Health (codes E32, F32, G32, K32, J32, L32, M32) – the title of this function was changed to *Health*. Also, health inspections related to agricultural products were added to this function. Previously, they were reported at function code 54, Agriculture, which was eliminated effective 2005.

- Forestry (codes E56, F56, G56, K56, J56, L56, M56) – this function was changed to reflect its limited applicability to Federal and state governments only. The new title is *Federal and State Forestry*.

Other expenditure code changes effective with 2005 are noted below.

- *Public Welfare, Federal Categorical Assistance Programs* (code *67) – this code was changed from E67 to J67, owing to the previously explained modification to the object code for assistance and subsidies.

- *Public Welfare, Other Cash Assistance Programs* (code *68) – this code was changed from E68 to J68, owing to the previously explained modification to the object code for assistance and subsidies.

- *Public Welfare, Vendor Payments for Medical Care* (code S74) – object code "S" was added as valid for this function to reflect payments to the Federal Government under Medicare Part D.

- *Total Capital Outlay* (exhibit code V98) – this category was added to the *Classification Manual* for the first time.

<u>Appendix 1.6.5 Debt Classification Category Revisions</u>

This edition of the *Classification Manual* contains significant revisions to the classification system used for government debt statistics. In keeping with the theme of the 2005 redesign, these revisions enhanced internal consistency in statistical categories and simplified the classification categories across levels and types of governments. Overall, the number of categories used to classify state and local government regular debt statistics was reduced from 66 to 8, with many of the former categories combined into broader groupings.

The 2005 revisions resulted in two changes to the classification system for government debt statistics that was in place from 1988 through 2004. First, the number of categories used in classifying the "purpose" of state and local government long-term debt (the functional activity the debt supported) was reduced from 8 to 2. The previous edition of this *Classification Manual* had debt categories that utilized the basic concept of "government sectors" described in Chapter 2, in combination with the functional concepts used for government expenditure statistics. The following categories of "purpose" were recognized:

General Debt: Elementary and Secondary Education
Higher and Other Education
Public Debt for Private Purposes
All Other Debt, N.E.C.

Utility Debt:	Water Supply Systems<br>Electric Power Systems<br>Natural Gas Supply Systems<br>Public Mass Transit Systems

Effective with 2005 surveys, these categories were consolidated, with only two categories remaining. These were:

Public Debt for Private Purposes
Debt for Unspecified Public Purposes

The second classification change was that the distinct categories used to classify "character" of long-term debt were eliminated. Prior to the 2004-05 survey year, the Census Bureau statistics on government debt also included the following additional classification category:

- *By character of long-term debt.* Long-term debt is classified as either backed by the full-faith and credit of the government or as nonguaranteed.

Effective with the 2005 surveys, however, Census Bureau statistics no longer distinguish between debt that was "guaranteed" (such as by the taxing power of the issuing government) and nonguaranteed (such as revenue bonds).

In summary, these changes meant that the Census Bureau statistics on government debt went from a four way classification system to a three way classification system.

By Length of Term Outstanding:	By Type:	By Purpose:
- Short-term	- Beginning	- Public Debt For Private Purpose
- Long-term	- Issued	- Public Debt, Unspecified Public Purpose
	- Retired	- Outstanding

The following pre-2005 debt codes thus were eliminated:

19A, 19B, 19C, 19D, 19H, 19X, 21A, 21B, 21C, 21D, 21F, 21G, 21X, 24A, 24B, 24C, 24D, 24F, 24G, 24X, 29A, 29B, 29C, 29D, 29F, 29G, 29X, 31A, 31B, 31C, 31D, 31F, 31G, 31X, 34A, 34B, 34C, 34D, 34F, 34G, 34X, 39A, 39B, 39C, 39D, 39F, 39G, 39X, 41A, 41B, 41C, 41D, 41F, 41G, 41X, 44A, 44B, 44C, 44D, 44F, 44G, 44X

Effective with the 2005 survey year, debt codes consisted of the following:

- 19T, 24T, 34T, and 44T were retained, and had the same meaning as previously.

- 19U, 29U, 39U, and 49U will be used for all other debt. It was necessary to create these new codes (using the "U" designation as opposed to the previous "X" designation for all other debt) so that historical data comparisons would not be misleading.

Table 6.2 in Chapter 6 shows the history of classification changes for debt categories for the three time periods – current (effective 2005), 1988 to 2004, and pre-1988.

The 2005 redesign did not result in any major shifts in the levels and trends of government debt statistics reported by the Census Bureau. Nor did the 2005 redesign create any need for historical revisions to the time series for government debt statistics. Since the changes in this edition of the *Classification Manual* represent consolidations of debt categories, all aggregates in the historical data are consistent, in terms of coverage and concept, with statistics from earlier years.

<u>Appendix 1.6.6 Cash and Security Classification Changes – Market Value</u>

There were no changes to the statistical classification categories for reporting cash and security holdings, based on the 2005 redesign of the government finance program. The changes to cash and security holdings categories for public employee retirement systems, effective 2002, were explained previously in Appendix 1.5.

The 2005 redesign of the government finance program did include a major change in definition for all cash and security holdings, however. The definition for all of the categories was modified to reflect market value of holdings. This change was made to maintain consistency with current accounting practices used by governments.

This major change in how the Census Bureau "valued" cash and security holdings was based largely on the evolution of governmental accounting practices. As a direct result of GASB guidelines, described earlier in this *Classification Manual*, most governments and the associated government accounting industry converted to market valuation for official financial reports. The definition of market value for purpose of the Census Bureau's government finance classification system, is noted in Chapter 7 and repeated here (see Section 7.2.2):

> Market value of cash and security holdings is defined in two ways, depending on the type of asset. For cash and cash-based short-term investments such as certificates of deposit, the valuation is based on current dollar value of the holdings. For all other security holdings, market value is the amount that a government can reasonably expect to receive for an investment in a current sale between a willing buyer and willing seller (other than in a force or liquidation sale). This definition is essentially the same as found in GASB statement 25. In both cases, the measure is taken as of the close of the government's fiscal year. Market value also is referred to as "fair value" in many accounting references.

As noted earlier in this Appendix, the market value concept had been applied to several categories of cash and security holdings for public employee retirement systems, effective with the 2002 Census of Governments. Those categories included corporate stocks (code Z78), corporate bonds (codes Z77, Z62, and Z63), and foreign and international securities (code Z70). The decision to value all assets at market value applied the same methodology to the remaining categories.

Appendix Table 1.1, *Pre 2005 Basis of Measurement for Cash and Security Holdings*, summarizes how cash and security holdings were measured in Census Bureau surveys.

<u>Appendix 1.7 Coverage Changes for Washington, DC</u>

One final result of the 2005 redesign involved coverage for Washington, DC. As noted throughout this *Classification Manual*, Washington, DC is classified as a municipality for purposes of Census Bureau surveys on government finance and public employment. However, prior to 2005, many of the more detailed classification categories that were used for state governments also were used for Washington, DC. As a result of the 2005 redesign, Governments Division decided to retain the status of Washington,

DC as a local (municipal) government for statistical purposes. Most of the statistical codes that apply to state governments only (such as the K object codes) were deemed to be invalid for Washington, DC. See Sections 3.13 and 11.5 for additional information.

Appendix Table 1.1
Pre 2005 Basis of Measurement for Cash and Security Holdings

This list includes general categories of assets that are covered in the Census Bureau definition of Cash & Security Holdings. It also contains Census Bureau "By Type" classification categories of regular codes used in the Census Bureau's government finance statistics program (see Chapter 7), plus exhibit codes used in the surveys of public employee retirement systems (see Chapter 8).

General Category, or "By Type" Classification (applicable Census Codes)	Measurement Method
Cash and Short-term Investments (X21)	Par value, current dollars
Cash & Demand Deposits (Z88)	Par value, current dollars
Time or Savings Deposits (Z87)	Par value, current dollars
All Other Short-term Investments (Z68)	Par value, current dollars
Government Securities:	
Total Federal Government Securities (X30)	Book value
Federal Treasury Securities (Z89)	Book value
Federal Agency Securities (X33)	Book value
State and Local Government (X35)	Book value
Nongovernmental Securities:	
Total Corporate Bonds (Z77)	Market value (Fair value) 1/
Corporate Bonds, Federally-Sponsored Agencies (Z62)	Market value (Fair value) 1/
Corporate Bonds, Other (Z63)	Market value (Fair value) 1/
Corporate Stocks (Z78)	Market value (Fair value) 1/
Mortgages Held Directly (X42)	Par (face) value of amount due
Foreign and International Securities (Z70)	Market value (Fair Value) 1/
All Other Securities (X44)	Book value
Investments Held in Trust (Z84)	Book value
Other Securities (Z83)	Book value
Total Other Investments (Z82):	
Real Property (X46)	Book value
Miscellaneous Investments (X47)	Book value
Unemployment Compensation Funds:	
Trust Fund Account - US Treasury (Y07)	Par value, current dollars
Clearing Account - US Treasury (Y08)	Par value, current dollars

Book Value = actual cost of holding, which could be net of discounts allowed
Par Value = actual face value of the security, loan or other holding, including cash.
Market Value = estimated value if holding were to be sold or otherwise liquidated.

1/ These asset types were measured at market value beginning in 2002, but at book value prior to 2002.

Appendix Table 1.2
State and Local Government Public Employee Retirement System Regular and Exhibit Variables, Current and Historical – page 1 of 2

Category	Currently reported at:	Where reported 1988-2001:	Historical notes
**Receipts:**			
Contributions, From Local Employees	X01	X01	
Contributions, From State Employees	X02	X02	
Contributions, From Parent Local Government	X04	X04	
Contributions, From Other Governments	X05	X05	
State Contributions to Own System, Total	X06	X06	
State Contributions for Local Employees	V87	V87	
State Contributions for State Employees	Z99	Z99	
Total Earnings on Investments	X08	X08	
Interest	Z71	Z73	Included in Z96, pre-2002
Dividends	Z72	Z72	Included in Z96, pre-1988
Rentals, from State Government	Z98	Z98	
Other Investment Earnings	Z73	Z73	Included in Z96, pre-1988
Losses on Investments	Z91	Z91	Pre-2002, Z91 also was included at X14 and reflected realized losses
Gains on Investments	Z96	Z96	Pre-2002, reflected realized gains
Other Receipts	Z95	Z95	
**Expenses:**			
Total Benefit Payments	X11	X11	
Retirement Benefits	Z13	...	No separate code - included at X11
Disability Benefits	Z14	...	No separate code - included at X11
Survivor Benefits	Z15	...	No separate code - included at X11
Other Benefits	Z16	...	No separate code - included at X11
Withdrawals	X12	X12	
Administrative Expenses	Z93	Z93	

**Appendix Table 1.2**<br>**Public Employee Retirement System Regular and Exhibit Variables,**<br>**Current and Historical** – page 2 of 2			
**Category**	**Currently reported at:**	**Where reported 1988 - 2001**	**Historical notes**
**Cash and Security Holdings:**			
Total Cash and Short-term Investments	X21	X21	
Cash on Hand and Demand Deposits	Z88	Z88	
Time or Savings Deposits	Z87	Z87	
All Other Short-term Investments	Z68	Z68	
Total Federal Government Securities	X30	X30	
Federal Treasury Securities	Z89	Z89	
Federal Agency Securities	X33	X33	
Total Corporate Bonds	Z77	Z77	
Bonds of Federally-Sponsored Agencies	Z62	...	
Corporate Bonds, Other	Z63	...	
Corporate Bonds	...	X40	Pre-2002, reflected book value
Federally-Sponsored Agencies	...	Z67	Pre-2002, reflected book value
Corporate Stocks	Z78	Z78	
Corporate Stocks	...	X41	Pre-2002, reflected book value
Mortgages Held Directly	X42	X42	
Total Other Securities	X44	X44	
Investments Held in Trust	Z84	Z84	
State and Local Government Securities	X35	X35	
Foreign and International Securities	Z70	...	1997-2001, included at Z69, book value. Pre-1997 included at Z83, book value.
Other Securities	Z83	Z83	Pre-1997, included foreign and international securities.
Total Other Investments	Z82	Z82	
Real Property	X46	X46	
Other Investments	X47	X47	
Total Cash and Security Holdings	Z81	Z81	

Notes:
Reflects codes for receipts (revenue), expenses (expenditure), and cash and security holdings only. Descriptive statistics are excluded from this presentation.

APPENDIX 2. Valid Codes Tables

This Appendix 2 of the *Government Finance and Employment Classification Manual* repeats all of the tables that depict finance code validity by level and type of government. These tables are repeated from their original occurrence in Chapters 3 through 9 with duplicates removed. Table 12.2, depicting valid codes for public employment functions by level and type of government, also is repeated as Appendix Table 2.3.

This appendix also contains one additional table for both analysts and users of the Census Bureau statistics on government finance. Appendix Table 2.2, Cross-Classification of Expenditure Function and Object Codes, is a useful reference when working with public use files.

Appendix Table 2.1<br>**Code Validity, by Level and Type of Government** – page 1 of 11								
**Code**	**Code Title**	**Code Type**	**Federal**	**State**	**General Local**	**Special District**	**School District**	**Retirement System**
A01	Air Transportation	R	Valid	Valid	Valid	Valid	X	X
A03	Miscellaneous Commercial Activities	R	X	Valid	Valid	Valid	X	X
A06	Nat. Defense/Int'National Relations	R	Valid	X	X	X	X	X
A09	El-Sec Education School Lunch	R	X	Valid	Valid	X	Valid	X
A10	El-Sec Education School Tuition	R	X	Valid	Valid	X	Valid	X
A12	Other El-Sec Education	R	X	Valid	Valid	X	Valid	X
A14	Federal Postal Service	R	Valid	X	X	X	X	X
A16	Higher Ed. Auxiliary Enterprises	R	X	Valid	Valid	X	Valid	X
A18	Other Higher Education	R	X	Valid	Valid	Valid	Valid	X
A21	Federal and State Ed. Charges NEC	R	Valid	Valid	X	X	X	X
A36	Public Hospitals	R	Valid	Valid	Valid	Valid	Valid	X
A44	Regular Highways	R	X	Valid	Valid	Valid	X	X
A45	Toll Highways	R	X	Valid	Valid	Valid	X	X
A50	Housing and Community Develop.	R	Valid	Valid	Valid	Valid	X	X
A56	Federal and State Forestry	R	Valid	Valid	X	X	X	X
A59	Other Natural Resources	R	Valid	Valid	Valid	Valid	X	X
A60	Parking Facilities	R	X	Valid	Valid	Valid	X	X
A61	Parks and Recreation	R	Valid	Valid	Valid	Valid	X	X
A80	Sewerage	R	X	Valid	Valid	Valid	X	X
A81	Solid Waste Management	R	Valid	Valid	Valid	Valid	X	X
A87	Sea and Inland Port Facilities	R	Valid	Valid	Valid	Valid	X	X
A89	All Other General Current Charges	R	Valid	Valid	Valid	Valid	X	X
A90	Liquor Store Revenue	R	X	Valid	Valid	X	X	X
A91	Water Supply Systems	R	X	Valid	Valid	Valid	X	X
A92	Electric Power Systems	R	X	Valid	Valid	Valid	X	X
A93	Gas Supply Systems	R	X	Valid	Valid	Valid	X	X
A94	Public Mass Transit Systems	R	X	Valid	Valid	Valid	X	X
B01	Air Transportation	R	X	Valid	Valid	Valid	X	X
B21	Education	R	X	Valid	Valid	Valid	Valid	X
B22[1]	Employment Security Administration	R	X	Valid	X[1]	X	X	X
B30	General Local Government Support	R	X	Valid	Valid	X	X	X
B42	Health and Hospitals	R	X	Valid	Valid	Valid	X	X
B46	Highways	R	X	Valid	Valid	Valid	X	X
B50	Housing and Community Develop.	R	X	Valid	Valid	Valid	X	X
B59	Natural Resources	R	X	Valid	Valid	Valid	X	X
B79	Public Welfare	R	X	Valid	Valid	Valid	X	X
B80	Sewerage	R	X	Valid	Valid	Valid	X	X
B89	All Other	R	X	Valid	Valid	Valid	X	X
B91	Water Supply Systems	R	X	Valid	Valid	Valid	X	X
B92	Electric Power Systems	R	X	Valid	Valid	Valid	X	X
B93	Gas Supply Systems	R	X	Valid	Valid	Valid	X	X
B94	Public Mass Transit Systems	R	X	Valid	Valid	Valid	X	X
C21[2]	Education	R	X	X	Valid[2]	Valid	Valid	X
C30[2]	General Local Government Support	R	X	X	Valid[2]	X	X	X
C42[2]	Health and Hospitals	R	X	X	Valid[2]	Valid	X	X
C46[2]	Highways	R	X	X	Valid[2]	Valid	X	X
C50[2]	Housing and Community Develop.	R	X	X	Valid[2]	Valid	X	X
C79[2]	Public Welfare	R	Valid	X	Valid[2]	Valid	X	X
C80[2]	Sewerage	R	X	X	Valid[2]	Valid	X	X
C89[2]	All Other	R	Valid	X	Valid[2]	Valid	X	X
C91[2]	Water Supply Systems	R	X	X	Valid[2]	Valid	X	X
C92[2]	Electric Power Systems	R	X	X	Valid[2]	Valid	X	X
C93[2]	Gas Supply Systems	R	X	X	Valid[2]	Valid	X	X

Appendix Table 2.1
Code Validity, by Level and Type of Government – page 2 of 11

Code	Code Title	Code Type	Federal	State	General Local	Special District	School District	Retirement System
C94[2]	Public Mass Transit Systems	R	X	X	Valid[2]	Valid	X	X
CGE[1]	Tobacco Lawsuit Settlement – Expenditure	E	X	Valid	X[1]	X	X	X
CGR[1]	Tobacco Lawsuit Settlement – Receipts	E	X	Valid	X[1]	X	X	X
D11	Interschool System Revenue	R	X	X	X	X	Valid	X
D21	Education	R	X	Valid	Valid	Valid	Valid	X
D30	General Local Government Support	R	X	Valid	Valid	X	X	X
D42	Health and Hospitals	R	X	Valid	Valid	Valid	X	X
D46	Highways	R	X	Valid	Valid	Valid	X	X
D50[3]	Housing and Community Develop.	R	X	Valid[3]	Valid	Valid	X	X
D79	Public Welfare	R	X	Valid	Valid	Valid	X	X
D80	Sewerage	R	X	Valid	Valid	Valid	X	X
D89	All Other	R	X	Valid	Valid	Valid	X	X
D91	Water Supply Systems	R	X	Valid	Valid	Valid	X	X
D92	Electric Power Systems	R	X	Valid	Valid	Valid	X	X
D93	Gas Supply Systems	R	X	Valid	Valid	Valid	X	X
D94	Public Mass Transit Systems	R	X	Valid	Valid	Valid	X	X
E01	Air Transportation	R	Valid	Valid	Valid	Valid	X	X
E02	Federal Space Research and Technology	R	Valid	X	X	X	X	X
E03	Miscellaneous Commercial Activities NEC	R	X	Valid	Valid	Valid	X	X
E04	Correctional Institutions	R	Valid	Valid	Valid	Valid	X	X
E05	Other Corrections	R	Valid	Valid	Valid	Valid	X	X
E06	Nat. Defense/Int'National Relations	R	Valid	X	X	X	X	X
E12	El-Sec Education	R	X	Valid	Valid	Valid	Valid	X
E14	Federal Postal Services	R	Valid	X	X	X	X	X
E16	Higher Ed. Auxiliary Enterprises	R	X	Valid	Valid	X	Valid	X
E18	Other Higher Education	R	X	Valid	Valid	X	Valid	X
E20	Federal Veterans Education Benefits	R	Valid	X	X	X	X	X
E21	Federal and State Other Education	R	Valid	Valid	X	X	X	X
E22[1]	Social Insurance Administration	R	Valid	Valid	X[2]	X	X	X
E23	Financial Administration	R	Valid	Valid	Valid	X	X	X
E24	Local Fire Protection	R	X	X	Valid	Valid	X	X
E25	Judicial and Legal	R	Valid	Valid	Valid	X	X	X
E26	Federal and State Legislative	R	Valid	Valid	X	X	X	X
E28	Federal Veterans' Health	R	Valid	X	X	X	X	X
E29	Central Staff Services	R	Valid	Valid	Valid	X	X	X
E31	General Public Buildings	R	X	Valid	Valid	X	X	X
E32	Health	R	Valid	Valid	Valid	Valid	X	X
E36	Hospitals	R	Valid	Valid	Valid	Valid	Valid	X
E37	Federal Own Hospitals – Veterans	R	Valid	X	X	X	X	X
E39	Federal Other Hospitals – Veterans	R	Valid	X	X	X	X	X
E44	Regular Highways	R	Valid	Valid	Valid	Valid	X	X
E45	Toll Highways	R	X	Valid	Valid	Valid	X	X
E50	Housing and Community Development	R	Valid	Valid	Valid	Valid	X	X
E51	Federal Farm Credit Programs	R	Valid	X	X	X	X	X
E52	Libraries	R	Valid	Valid	Valid	Valid	X	X
E53	Federal Farm Income Stabilization	R	Valid	X	X	X	X	X
E55	State Fish and Game	R	X	Valid	X	X	X	X
E56	Federal and State Forestry	R	Valid	Valid	X	X	X	X
E57	Federal Soil, Water, and Electric Resource	R	Valid	X	X	X	X	X
E58	Federal Mineral Resources	R	Valid	X	X	X	X	X
E59	Natural Resources, Other	R	Valid	Valid	Valid	Valid	X	X
E60	Parking Facilities	R	X	Valid	Valid	Valid	X	X
E61	Parks and Recreation	R	Valid	Valid	Valid	Valid	X	X

Appendix Table 2.1
Code Validity, by Level and Type of Government – page 3 of 11

Code	Code Title	Code Type	Federal	State	General Local	Special District	School District	Retirement System
E62	Police Protection	R	Valid	Valid	Valid	Valid	X	X
E66	Protective Inspection and Regulation NEC	R	X	Valid	Valid	X	X	X
E74	Vendor Payments for Medical Care	R	X	Valid	Valid	X	X	X
E75	Vendor Payments for Other Purposes	R	X	Valid	Valid	X	X	X
E77	Public Welfare – Institutions	R	X	Valid	Valid	Valid	X	X
E79	Public Welfare – Other	R	Valid	Valid	Valid	Valid	X	X
E80	Sewerage	R	X	Valid	Valid	Valid	X	X
E81	Solid Waste Management	R	Valid	Valid	Valid	Valid	X	X
E85	Federal and State Veterans' Services	R	Valid	Valid	X	X	X	X
E87	Sea and Inland Port Facilities	R	Valid	Valid	Valid	Valid	X	X
E89	Other and Unallocable	R	Valid	Valid	Valid	Valid	X	X
E90	Liquor Stores	R	X	Valid	Valid	X	X	X
E91	Water Supply	R	X	Valid	Valid	Valid	X	X
E92	Electric Supply	R	X	Valid	Valid	Valid	X	X
E93	Gas Supply	R	X	Valid	Valid	Valid	X	X
E94	Public Mass Transit Systems	R	X	Valid	Valid	Valid	X	X
F01	Air Transportation	R	Valid	Valid	Valid	Valid	X	X
F02	Federal Space Research and Technology	R	Valid	X	X	X	X	X
F03	Miscellaneous Commercial Activities NEC	R	X	Valid	Valid	Valid	X	X
F04	Correctional Institutions	R	Valid	Valid	Valid	Valid	X	X
F05	Other Corrections	R	Valid	Valid	Valid	Valid	X	X
F06	Nat. Defense/Int'National Relations	R	Valid	X	X	X	X	X
F12	El-Sec Education	R	X	Valid	Valid	Valid	Valid	X
F14	Federal Postal Services	R	Valid	X	X	X	X	X
F16	Higher Ed. Auxiliary Enterprises	R	X	Valid	Valid	X	Valid	X
F18	Other Higher Education	R	X	Valid	Valid	X	Valid	X
F20	Federal Veterans Education Benefits	R	Valid	X	X	X	X	X
F21	Federal and State Other Education	R	Valid	Valid	X	X	X	X
F22[1]	Social Insurance Administration	R	Valid	Valid	X[1]	X	X	X
F23	Financial Administration	R	Valid	Valid	Valid	X	X	X
F24	Local Fire Protection	R	X	X	Valid	Valid	X	X
F25	Judicial and Legal	R	Valid	Valid	Valid	X	X	X
F26	Federal and State Legislative	R	Valid	Valid	X	X	X	X
F28	Federal Veterans' Health	R	Valid	X	X	X	X	X
F29	Central Staff Services	R	Valid	Valid	Valid	X	X	X
F31	General Public Buildings	R	X	Valid	Valid	X	X	X
F32	Health	R	Valid	Valid	Valid	Valid	X	X
F36	Hospitals	R	Valid	Valid	Valid	Valid	Valid	X
F37	Federal Own Hospitals – Veterans	R	Valid	X	X	X	X	X
F39	Federal Other Hospitals – Veterans	R	Valid	X	X	X	X	X
F44	Regular Highways	R	Valid	Valid	Valid	Valid	X	X
F45	Toll Highways	R	X	Valid	Valid	Valid	X	X
F50	Housing and Community Development	R	Valid	Valid	Valid	Valid	X	X
F52	Libraries	R	Valid	Valid	Valid	Valid	X	X
F53	Federal Farm Income Stabilization	R	Valid	X	X	X	X	X
F55	State Fish and Game	R	X	Valid	X	X	X	X
F56	Federal and State Forestry	R	Valid	Valid	X	X	X	X
F57	Federal Soil, Water, and Electric Resource	R	Valid	X	X	X	X	X
F58	Federal Mineral Resources	R	Valid	X	X	X	X	X
F59	Natural Resources, Other	R	Valid	Valid	Valid	Valid	X	X
F60	Parking Facilities	R	X	Valid	Valid	Valid	X	X
F61	Parks and Recreation	R	Valid	Valid	Valid	Valid	X	X
F62	Police Protection	R	Valid	Valid	Valid	Valid	X	X

Appendix Table 2.1<br>**Code Validity, by Level and Type of Government** – page 4 of 11								
**Code**	**Code Title**	**Code Type**	**Federal**	**State**	**General Local**	**Special District**	**School District**	**Retirement System**
F66	Protective Inspection and Regulation NEC	R	X	Valid	Valid	X	X	X
F77	Public Welfare – Institutions	R	X	Valid	Valid	Valid	X	X
F79	Public Welfare – Other	R	Valid	Valid	Valid	Valid	X	X
F80	Sewerage	R	X	Valid	Valid	Valid	X	X
F81	Solid Waste Management	R	Valid	Valid	Valid	Valid	X	X
F85	Federal and State Veterans' Services	R	Valid	Valid	X	X	X	X
F87	Sea and Inland Port Facilities	R	Valid	Valid	Valid	Valid	X	X
F89	Other and Unallocable	R	Valid	Valid	Valid	Valid	X	X
F90	Liquor Stores	R	X	Valid	Valid	X	X	X
F91	Water Supply	R	X	Valid	Valid	Valid	X	X
F92	Electric Supply	R	X	Valid	Valid	Valid	X	X
F93	Gas Supply	R	X	Valid	Valid	Valid	X	X
F94	Public Mass Transit	R	X	Valid	Valid	Valid	X	X
G01	Air Transportation	R	Valid	Valid	Valid	Valid	X	X
G02	Federal Space Research and Technology	R	Valid	X	X	X	X	X
G03	Miscellaneous Commercial Activities NEC	R	X	Valid	Valid	Valid	X	X
G04	Correctional Institutions	R	Valid	Valid	Valid	Valid	X	X
G05	Other Corrections	R	Valid	Valid	Valid	Valid	X	X
G06	Nat. Defense/Int'National Relations	R	Valid	X	X	X	X	X
G12	El-Sec Education	R	X	Valid	Valid	Valid	Valid	X
G14	Federal Postal Services	R	Valid	X	X	X	X	X
G16	Higher Ed. Auxiliary Enterprises	R	X	Valid	Valid	X	Valid	X
G18	Other Higher Education	R	X	Valid	Valid	X	Valid	X
G20	Federal Veterans Education Benefits	R	Valid	X	X	X	X	X
G21	Federal and State Other Education	R	Valid	Valid	X	X	X	X
G22[1]	Social Insurance Administration	R	Valid	Valid	X[1]	X	X	X
G23	Financial Administration	R	Valid	Valid	Valid	X	X	X
G24	Local Fire Protection	R	X	X	Valid	Valid	X	X
G25	Judicial and Legal	R	Valid	Valid	Valid	X	X	X
G26	Federal and State Legislative	R	Valid	Valid	X	X	X	X
G28	Federal Veterans' Health	R	Valid	X	X	X	X	X
G29	Central Staff Services	R	Valid	Valid	Valid	X	X	X
G31	General Public Buildings	R	X	Valid	Valid	X	X	X
G32	Health	R	Valid	Valid	Valid	Valid	X	X
G36	Hospitals	R	Valid	Valid	Valid	Valid	Valid	X
G37	Federal Own Hospitals – Veterans	R	Valid	X	X	X	X	X
G39	Federal Other Hospitals – Veterans	R	Valid	X	X	X	X	X
G44	Regular Highways	R	Valid	Valid	Valid	Valid	X	X
G45	Toll Highways	R	X	Valid	Valid	Valid	X	X
G50	Housing and Community Development	R	Valid	Valid	Valid	Valid	X	X
G52	Libraries	R	Valid	Valid	Valid	Valid	X	X
G53	Federal Farm Income Stabilization	R	Valid	X	X	X	X	X
G55	State Fish and Game	R	X	Valid	X	X	X	X
G56	Federal and State Forestry	R	Valid	Valid	X	X	X	X
G57	Federal Soil, Water, and Electric Resource	R	Valid	X	X	X	X	X
G58	Federal Mineral Resources	R	Valid	X	X	X	X	X
G59	Natural Resources, Other	R	Valid	Valid	Valid	Valid	X	X
G60	Parking Facilities	R	X	Valid	Valid	Valid	X	X
G61	Parks and Recreation	R	Valid	Valid	Valid	Valid	X	X
G62	Police Protection	R	Valid	Valid	Valid	Valid	X	X
G66	Protective Inspection and Regulation NEC	R	X	Valid	Valid	X	X	X
G77	Public Welfare – Institutions	R	X	Valid	Valid	Valid	X	X

Appendix Table 2.1
Code Validity, by Level and Type of Government – page 5 of 11

Code	Code Title	Code Type	Federal	State	General Local	Special District	School District	Retirement System
G79	Public Welfare – Other	R	Valid	Valid	Valid	Valid	X	X
G80	Sewerage	R	X	Valid	Valid	Valid	X	X
G81	Solid Waste Management	R	Valid	Valid	Valid	Valid	X	X
G85	Federal and State Veterans' Services	R	Valid	Valid	X	X	X	X
G87	Sea and Inland Port Facilities	R	Valid	Valid	Valid	Valid	X	X
G89	Other and Unallocable	R	Valid	Valid	Valid	Valid	X	X
G90	Liquor Stores	R	X	Valid	Valid	X	X	X
G91	Water Supply	R	X	Valid	Valid	Valid	X	X
G92	Electric Supply	R	X	Valid	Valid	Valid	X	X
G93	Gas Supply	R	X	Valid	Valid	Valid	X	X
G94	Public Mass Transit	R	X	Valid	Valid	Valid	X	X
I89	Interest on General Debt	R	Valid	Valid	Valid	Valid	Valid	X
I91	Interest on Water Utility Debt	R	X	Valid	Valid	Valid	X	X
I92	Interest on Electric Power Debt	R	X	Valid	Valid	Valid	X	X
I93	Interest on Gas Supply Debt	R	X	Valid	Valid	Valid	X	X
I94	Interest on Public Mass Transit Debt	R	X	Valid	Valid	Valid	X	X
J01	Air Transportation	R	Valid	X	X	X	X	X
J02	Federal Space Research and Technology	R	Valid	X	X	X	X	X
J04	Correctional Institutions	R	Valid	X	X	X	X	X
J06	Nat. Defense/Int'National Relations	R	Valid	X	X	X	X	X
J14	Federal Postal Services	R	Valid	X	X	X	X	X
J19	State Scholarships and Subsidies, Ed	R	X	Valid	X	X	X	X
J20	Federal Veterans Education Benefits	R	Valid	X	X	X	X	X
J21	Federal and State Other Education	R	Valid	X	X	X	X	X
J22	Social Insurance Administration	R	Valid	X	X	X	X	X
J23	Financial Administration	R	Valid	X	X	X	X	X
J25	Judicial and Legal	R	Valid	X	X	X	X	X
J28	Federal Veterans' Health	R	Valid	X	X	X	X	X
J29	Central Staff Services	R	Valid	X	X	X	X	X
J32	Health	R	Valid	X	X	X	X	X
J36	Hospitals	R	Valid	X	X	X	X	X
J37	Federal Own Hospitals – Veterans	R	Valid	X	X	X	X	X
J39	Federal Other Hospitals – Veterans	R	Valid	X	X	X	X	X
J44	Regular Highways	R	Valid	X	X	X	X	X
J50	Housing and Community Development	R	Valid	X	X	X	X	X
J51	Federal Farm Credit Programs	R	Valid	X	X	X	X	X
J52	Libraries	R	Valid	X	X	X	X	X
J53	Federal Farm Income Stabilization	R	Valid	X	X	X	X	X
J56	Federal and State Forestry	R	Valid	X	X	X	X	X
J57	Federal Soil, Water, and Electric Resource	R	Valid	X	X	X	X	X
J58	Federal Mineral Resource	R	Valid	X	X	X	X	X
J59	Natural Resources, Other	R	Valid	X	X	X	X	X
J61	Parks and Recreation	R	Valid	X	X	X	X	X
J62	Police Protection	R	Valid	X	X	X	X	X
J67	Federal Categorical Assistance Programs	R	Valid	Valid	Valid	X	X	X
J68	Other Cash Assistance Programs	R	X	Valid	Valid	X	X	X
J79	Public – Welfare Other	R	Valid	X	X	X	X	X
J85	Federal and State Veterans' Services	R	Valid	Valid	X	X	X	X
J87	Sea and Inland Port Facilities	R	Valid	X	X	X	X	X
J89	Other and Unallocable	R	Valid	X	X	X	X	X
K01	Air Transportation	R	Valid	Valid	X	X	X	X
K02	Federal Space Research and Technology	R	Valid	X	X	X	X	X

Appendix Table 2.1<br>Code Validity, by Level and Type of Government – page 6 of 11								
**Code**	**Code Title**	**Code Type**	**Federal**	**State**	**General Local**	**Special District**	**School District**	**Retirement System**
K03	Miscellaneous Commercial Activities NEC	R	X	Valid	X	X	X	X
K04	Correctional Institutions	R	Valid	Valid	X	X	X	X
K05	Other Corrections	R	Valid	Valid	X	X	X	X
K06	Nat. Defense/Int'National Relations	R	Valid	X	X	X	X	X
K12	El-Sec Education	R	X	Valid	X	X	X	X
K14	Federal Postal Services	R	Valid	X	X	X	X	X
K16	Higher Ed. Auxiliary Enterprises	R	X	Valid	X	X	X	X
K18	Other Higher Education	R	X	Valid	X	X	X	X
K20	Federal Veterans Education Benefits	R	Valid	X	X	X	X	X
K21	Federal and State Other Education	R	Valid	Valid	X	X	X	X
K22	Social Insurance Administration	R	Valid	Valid	X	X	X	X
K23	Financial Administration	R	Valid	Valid	X	X	X	X
K25	Judicial and Legal	R	Valid	Valid	X	X	X	X
K26	Federal and State Legislative	R	Valid	Valid	X	X	X	X
K28	Federal Veterans' Health	R	Valid	X	X	X	X	X
K29	Central Staff Services	R	Valid	Valid	X	X	X	X
K31	General Public Buildings	R	X	Valid	X	X	X	X
K32	Health	R	Valid	Valid	X	X	X	X
K36	Hospitals	R	Valid	Valid	X	X	X	X
K37	Federal Own Hospitals – Veterans	R	Valid	X	X	X	X	X
K39	Federal Other Hospitals – Veterans	R	Valid	X	X	X	X	X
K44	Regular Highways	R	Valid	Valid	X	X	X	X
K45	Toll Highways	R	X	Valid	X	X	X	X
K50	Housing and Community Development	R	Valid	Valid	X	X	X	X
K52	Libraries	R	Valid	Valid	X	X	X	X
K53	Federal Farm Income Stabilization	R	Valid	X	X	X	X	X
K55	State Fish and Game	R	X	Valid	X	X	X	X
K56	Federal and State Forestry	R	Valid	Valid	X	X	X	X
K57	Federal Soil, Water, and Electric Resource	R	Valid	X	X	X	X	X
K58	Federal Mineral Resources	R	Valid	X	X	X	X	X
K59	Natural Resources, Other	R	Valid	Valid	X	X	X	X
K60	Parking Facilities	R	X	Valid	X	X	X	X
K61	Parks and Recreation	R	Valid	Valid	X	X	X	X
K62	Police Protection	R	Valid	Valid	X	X	X	X
K66	Protective Inspection and Regulation NEC	R	X	Valid	X	X	X	X
K77	Public Welfare – Institutions	R	X	Valid	X	X	X	X
K79	Public Welfare – Other	R	Valid	Valid	X	X	X	X
K80	Sewerage	R	X	Valid	X	X	X	X
K81	Solid Waste Management	R	Valid	Valid	X	X	X	X
K85	Federal and State Veterans' Services	R	Valid	Valid	X	X	X	X
K87	Sea and Inland Port Facilities	R	Valid	Valid	X	X	X	X
K89	Other and Unallocable	R	Valid	Valid	X	X	X	X
K90	Liquor Stores	R	X	Valid	X	X	X	X
K91	Water Supply	R	X	Valid	X	X	X	X
K92	Electric Supply	R	X	Valid	X	X	X	X
K93	Gas Supply	R	X	Valid	X	X	X	X
K94	Public Mass Transit	R	X	Valid	X	X	X	X
L01	Air Transportation	R	Valid	X	Valid	Valid	X	X
L02	Federal Space Research and Technology	R	Valid	X	X	X	X	X
L04	Correctional Institutions	R	Valid	X	Valid	Valid	X	X
L05	Other Corrections	R	Valid	X	Valid	Valid	X	X
L12	El-Sec Education	R	X	X	Valid	Valid	Valid	X

Appendix Table 2.1<br>Code Validity, by Level and Type of Government – page 7 of 11								
**Code**	**Code Title**	**Code Type**	**Federal**	**State**	**General Local**	**Special District**	**School District**	**Retirement System**
L18	Other Higher Education	R	X	X	Valid	X	Valid	X
L20	Federal Veterans Education Benefits	R	Valid	X	X	X	X	X
L21	Federal and State Other Education	R	Valid	X	X	X	X	X
L22	Social Insurance Administration	R	Valid	X	X	X	X	X
L23	Financial Administration	R	Valid	X	Valid	X	X	X
L25	Judicial and Legal	R	Valid	X	Valid	X	X	X
L28	Federal Veterans' Health	R	Valid	X	X	X	X	X
L29	Central Staff Services	R	Valid	X	Valid	X	X	X
L32	Health	R	Valid	X	Valid	Valid	X	X
L36	Hospitals	R	Valid	X	Valid	Valid	Valid	X
L37	Federal Own Hospitals – Veterans	R	Valid	X	X	X	X	X
L39	Federal Other Hospitals – Veterans	R	Valid	X	X	X	X	X
L44	Regular Highways	R	Valid	X	Valid	Valid	X	X
L50[4]	Housing and Community Development	R	Valid	X	Valid[4]	Valid[4]	X	X
L51	Federal Farm Credit Programs	R	Valid	X	X	X	X	X
L52	Libraries	R	Valid	X	Valid	Valid	X	X
L53	Federal Farm Income Stabilization	R	Valid	X	X	X	X	X
L56	Federal and State Forestry	R	Valid	X	X	X	X	X
L57	Federal Soil, Water, and Electric Resource	R	Valid	X	X	X	X	X
L58	Federal Mineral Resource	R	Valid	X	X	X	X	X
L59	Natural Resources – Other	R	Valid	X	Valid	Valid	X	X
L60	Parking Facilities	R	X	X	Valid	Valid	X	X
L61	Parks and Recreation	R	Valid	X	Valid	Valid	X	X
L62	Police Protection	R	Valid	X	Valid	Valid	X	X
L66	Protective Inspection and Regulation NEC	R	X	X	Valid	X	X	X
L67	Federal Categorical Assistance Programs	R	Valid	X	Valid	X	X	X
L79	Public Welfare – Other	R	Valid	X	Valid	Valid	X	X
L80	Sewerage	R	X	X	Valid	Valid	X	X
L81	Solid Waste Management	R	Valid	X	Valid	Valid	X	X
L87	Sea and Inland Port Facilities	R	Valid	X	Valid	Valid	X	X
L89	Other and Unallocable	R	Valid	X	Valid	Valid	X	X
L91	Water Supply	R	X	X	Valid	Valid	X	X
L92	Electric Power	R	X	X	Valid	Valid	X	X
L93	Gas Supply	R	X	X	Valid	Valid	X	X
L94	Public Mass Transit	R	X	X	Valid	Valid	X	X
M01	Air Transportation	R	Valid	Valid	Valid	Valid	X	X
M02	Federal Space Research and Technology	R	Valid	X	X	X	X	X
M04	Correctional Institutions	R	Valid	Valid	Valid	Valid	X	X
M05	Other Corrections	R	Valid	Valid	Valid	Valid	X	X
M12	El-Sec Education	R	X	Valid	Valid	Valid	Valid	X
M18	Other Higher Education	R	X	Valid	Valid	X	Valid	X
M20	Federal Veterans Education Benefits	R	Valid	X	X	X	X	X
M21	Federal and State Other Education	R	Valid	Valid	X	X	X	X
M22	Social Insurance Administration	R	Valid	X	X	X	X	X
M23	Financial Administration	R	Valid	Valid	Valid	X	X	X
M24	Local Fire Protection	R	X	X	Valid	Valid	X	X
M25	Judicial and Legal	R	Valid	Valid	Valid	X	X	X
M28	Federal Veterans' Health	R	Valid	X	X	X	X	X
M29	Central Staff Services	R	Valid	Valid	Valid	X	X	X
M30	General Local Government Support	R	X	Valid	X	X	X	X
M32	Health	R	Valid	Valid	Valid	Valid	X	X
M36	Hospitals	R	Valid	Valid	Valid	Valid	Valid	X

Appendix Table 2.1<br>Code Validity, by Level and Type of Government – page 8 of 11								
**Code**	**Code Title**	**Code Type**	**Federal**	**State**	**General Local**	**Special District**	**School District**	**Retirement System**
M37	Federal Own Hospitals – Veterans	R	Valid	X	X	X	X	X
M39	Federal Other Hospitals – Veterans	R	Valid	X	X	X	X	X
M44	Regular Highways	R	Valid	Valid	Valid	Valid	X	X
M50	Housing and Community Development	R	Valid	Valid	Valid	Valid	X	X
M51	Federal Farm Credit Programs	R	Valid	X	X	X	X	X
M52	Libraries	R	Valid	Valid	Valid	Valid	X	X
M53	Federal Farm Income Stabilization	R	Valid	X	X	X	X	X
M55	State Fish and Game	R	X	Valid	X	X	X	X
M56	Federal and State Forestry	R	Valid	Valid	X	X	X	X
M57	Federal Soil, Water, and Electric Resource	R	Valid	X	X	X	X	X
M58	Federal Mineral Resource	R	Valid	X	X	X	X	X
M59	Natural Resources – Other	R	Valid	Valid	Valid	Valid	X	X
M60	Parking Facilities	R	X	Valid	Valid	Valid	X	X
M61	Parks and Recreation	R	Valid	Valid	Valid	Valid	X	X
M62	Police Protection	R	Valid	Valid	Valid	Valid	X	X
M66	Protective Inspection and Regulation NEC	R	X	Valid	Valid	X	X	X
M67	Federal Categorical Assistance Programs	R	Valid	Valid	Valid	X	X	X
M68	Other Cash Assistance Programs	R	X	Valid	Valid	X	X	X
M79	Public Welfare – Other	R	Valid	Valid	Valid	Valid	X	X
M80	Sewerage	R	X	Valid	Valid	Valid	X	X
M81	Solid Waste Management	R	Valid	Valid	Valid	Valid	X	X
M87	Sea and Inland Port Facilities	R	Valid	Valid	Valid	Valid	X	X
M89	Other and Unallocable	R	Valid	Valid	Valid	Valid	X	X
M91	Water Supply	R	X	Valid	Valid	Valid	X	X
M92	Electric Power	R	X	Valid	Valid	Valid	X	X
M93	Gas Supply	R	X	Valid	Valid	Valid	X	X
M94	Public Mass Transit	R	X	Valid	Valid	Valid	X	X
Q12	El-Sec Education	R	X	Valid	X	X	Valid	X
Q18	Other Higher Education	R	X	Valid	X	X	X	X
S67[1]	Federal Categorical Assistance Programs	R	X	Valid	X[1]	X	X	X
S74[1]	Other Cash Assistance Programs	R	X	Valid	X[1]	X	X	X
S89	Other and Unallocable	R	X	Valid	X	X	X	X
T01	Property Taxes	R	X	Valid	Valid	Valid	Valid	X
T08	Federal Customs Duties	R	Valid	X	X	X	X	X
T09	General Sales and Gross Receipts Taxes	R	X	Valid	Valid	Valid	Valid	X
T10	Alcoholic Beverages	R	Valid	Valid	Valid	Valid	Valid	X
T11	Amusements	R	X	Valid	Valid	Valid	Valid	X
T12	Insurance Premiums	R	X	Valid	Valid	Valid	Valid	X
T13	Motor Fuels	R	Valid	Valid	Valid	Valid	Valid	X
T14	Pari-mutuels	R	X	Valid	Valid	Valid	Valid	X
T15	Public Utilities	R	Valid	Valid	Valid	Valid	Valid	X
T16	Tobacco Products	R	Valid	Valid	Valid	Valid	Valid	X
T19	Other Selective Sales and Gross Receipts	R	Valid	Valid	Valid	Valid	Valid	X
T20	Alcoholic Beverages	R	X	Valid	Valid	Valid	Valid	X
T21	Amusements	R	X	Valid	Valid	Valid	Valid	X
T22	Corporations in General	R	X	Valid	Valid	Valid	Valid	X
T23	Hunting and Fishing	R	X	Valid	Valid	Valid	Valid	X
T24	Motor Vehicles	R	X	Valid	Valid	Valid	Valid	X
T25	Motor Vehicle Operators	R	X	Valid	Valid	Valid	Valid	X
T27	Public Utilities	R	X	Valid	Valid	Valid	Valid	X
T28	Occupation and Businesses NEC	R	X	Valid	Valid	Valid	Valid	X
T29	Other License Taxes	R	X	Valid	Valid	Valid	Valid	X

Appendix Table 2.1<br>**Code Validity, by Level and Type of Government** – page 9 of 11								
**Code**	**Code Title**	**Code Type**	**Federal**	**State**	**General Local**	**Special District**	**School District**	**Retirement System**
T40	Individual Income Taxes	R	Valid	Valid	Valid	Valid	Valid	X
T41	Corporation Net Income Taxes	R	Valid	Valid	Valid	Valid	Valid	X
T50	Death and Gift Taxes	R	Valid	Valid	Valid	Valid	Valid	X
T51	Documentary and Stock Transfer Taxes	R	X	Valid	Valid	Valid	Valid	X
T53	Severance Taxes	R	X	Valid	Valid	Valid	Valid	X
T99	Taxes NEC	R	Valid	Valid	Valid	Valid	Valid	X
U01	Special Assessments	R	X	Valid	Valid	Valid	Valid	X
U11	Sale of Property	R	Valid	Valid	Valid	Valid	Valid	X
U20	Interest Earnings	R	Valid	Valid	Valid	Valid	Valid	X
U21	State - Other Dividends	R	X	Valid	X	X	X	X
U30	Fines and Forfeits	R	Valid	Valid	Valid	Valid	Valid	X
U40	Rents	R	X	Valid	Valid	X	X	X
U41	Royalties	R	X	Valid	Valid	Valid	Valid	X
U50	Donations from Private Sources	R	X	Valid	Valid	Valid	Valid	X
U95	Net Lottery Revenue	R	X	Valid	Valid	Valid	Valid	X
U99	Miscellaneous General Revenue NEC	R	Valid	Valid	Valid	Valid	Valid	X
V01	Description of System	D	X	Valid	Valid	Valid	Valid	Valid
V02	Type of Coverage	D	X	Valid	Valid	Valid	Valid	Valid
V03	Basis of Membership and Participation	D	X	Valid	Valid	Valid	Valid	Valid
V04	Employee Contribution Status	D	X	Valid	Valid	Valid	Valid	Valid
V05	Optional Benefits Available	D	X	Valid	Valid	Valid	Valid	Valid
V07	Time Required to Vest	D	X	Valid	Valid	Valid	Valid	Valid
V87	State Contributions to Own System	E	X	Valid	X	X	X	Valid
V98	Total Capital Outlay	E	X	X	Valid	Valid	Valid	X
W01	Cash and Security – Sinking Funds	R	X	Valid	Valid	Valid	Valid	X
W31	Cash and Security – Bond Funds	R	X	Valid	Valid	Valid	Valid	X
W61	Cash and Security – All Other Funds	R	X	Valid	Valid	Valid	Valid	X
X01	Contributions, Local Employees	R	X	Valid	Valid	Valid	Valid	Valid
X02	Contributions, State Employees	R	X	Valid	X	X	X	Valid
X03	Contributions, Federal Employees	R	Valid	X	X	X	X	Valid
X04	From Parent Local Government	E	X	X	Valid	Valid	Valid	Valid
X05	Contributions from Other Governments	R	X	Valid	Valid	Valid	Valid	Valid
X06	State Contributions to Own System, Total	E	X	Valid	X	X	X	Valid
X08	Earnings on Investment	R	X	Valid	Valid	Valid	Valid	Valid
X11	Benefit Payments	R	Valid	Valid	Valid	Valid	Valid	Valid
X12	Withdrawals	R	X	Valid	Valid	Valid	Valid	Valid
X21	Total Cash and Short-Term Investments	R	X	Valid	Valid	Valid	Valid	Valid
X30	Total Federal Government Securities	R	X	Valid	Valid	Valid	Valid	Valid
X33	Federal Agency Securities	E	X	Valid	Valid	Valid	Valid	Valid
X35	State and Local Government Securities	E	X	Valid	Valid	Valid	Valid	Valid
X42	Mortgages Held Directly	R	X	Valid	Valid	Valid	Valid	Valid
X44	Total Other Securities	R	X	Valid	Valid	Valid	Valid	Valid
X46	Real Property	E	X	Valid	Valid	Valid	Valid	Valid
X47	Other Investments	R	X	Valid	Valid	Valid	Valid	Valid
X99	Interest on U.S. Securities	E	Valid	X	X	X	X	Valid
Y01[1]	Contributions	R	X	Valid	X[1]	X	X	X
Y02[1]	Interest Credited by U.S. Government	R	X	Valid	X[1]	X	X	X
Y04[1]	Federal Advances and Contributions	R	X	Valid	X[1]	X	X	X
Y05[1]	Benefit Payments	R	Valid	Valid	X[1]	X	X	X
Y06[1]	Extended and Special Benefits	R	X	Valid	X[1]	X	X	X
Y07[1]	Trust Fund Account in U.S. Treasury	R	X	Valid	X[1]	X	X	X
Y08[1]	Other – Clearing and Benefit Account	R	X	Valid	X[1]	X	X	X

Appendix Table 2.1
Code Validity, by Level and Type of Government – page 10 of 11

Code	Code Title	Code Type	Federal	State	General Local	Special District	School District	Retirement System
Y10	Contributions to Own System	E	X	Valid	X	X	X	X
Y11	Contributions	R	X	Valid	X	X	X	X
Y12	Earnings on Investment	R	X	Valid	X	X	X	X
Y14	Benefit Payments	R	X	Valid	X	X	X	X
Y15	Administrative Expenses	E	X	Valid	X	X	X	X
Y20	Federal Social Security and Medicare	R	Valid	X	X	X	X	X
Y21	Total Cash and Security Holdings	R	X	Valid	X	X	X	X
Y22	Interest on U.S. Securities	E	Valid	X	X	X	X	X
Y25	Benefit Payments	R	Valid	X	X	X	X	X
Y31	Federal Veterans' Life Insurance	R	Valid	X	X	X	X	X
Y32	Interest on U.S. Securities	E	Valid	X	X	X	X	X
Y34	Benefit Payments	R	Valid	X	X	X	X	X
Y41	Federal Railroad Retirement	R	Valid	X	X	X	X	X
Y42	Interest on U.S. Securities	E	Valid	X	X	X	X	X
Y45	Benefit Payments	R	Valid	X	X	X	X	X
Y50	Contributions to Own System	E	X	Valid	X	X	X	X
Y51	Contributions	R	X	Valid	X	X	X	X
Y52	Earnings on Investment	R	X	Valid	X	X	X	X
Y53	Benefit and Withdrawal Payments	R	X	Valid	X	X	X	X
Y54	Administrative Expenses	E	X	Valid	X	X	X	X
Y61	Cash and Security Holdings	R	X	Valid	X	X	X	X
Y99	Interest on U.S. Securities	E	Valid	X	X	X	X	X
Z00	Total Salary and Wages	E	Valid	Valid	Valid	Valid	Valid	X
Z01	Number of Active Members	D	X	Valid	Valid	Valid	Valid	Valid
Z02	Number of Inactive Members	D	X	Valid	Valid	Valid	Valid	Valid
Z03	Beneficiaries Retired, Age or Service	D	X	Valid	Valid	Valid	Valid	Valid
Z04	Beneficiaries Retired, Disability	D	X	Valid	Valid	Valid	Valid	Valid
Z05	Beneficiaries, Survivors	D	X	Valid	Valid	Valid	Valid	Valid
Z06	Lump-sum Payments, Members	D	X	Valid	Valid	Valid	Valid	Valid
Z07	Lump-sum Payments, Survivors	D	X	Valid	Valid	Valid	Valid	Valid
Z08	Monthly Amount Paid, to Retirees	D	X	Valid	Valid	Valid	Valid	Valid
Z09	Monthly Amount Paid, Disability	D	X	Valid	Valid	Valid	Valid	Valid
Z10	Monthly Amount Paid, Survivors	D	X	Valid	Valid	Valid	Valid	Valid
Z11	Monthly Amount Paid, Lump, Members	D	X	Valid	Valid	Valid	Valid	Valid
Z12	Monthly Amount Paid, Lump, Survivors	D	X	Valid	Valid	Valid	Valid	Valid
Z13	Retirement Benefits	E	Valid	Valid	Valid	Valid	Valid	Valid
Z14	Disability Benefits	E	Valid	Valid	Valid	Valid	Valid	Valid
Z15	Survivor Benefits	E	Valid	Valid	Valid	Valid	Valid	Valid
Z16	Other Benefits	E	Valid	Valid	Valid	Valid	Valid	Valid
Z41	Net Sales of Goods	E	X	Valid	X	X	X	X
Z42	Cost of Goods Sold	E	X	Valid	X	X	X	X
Z43	Operating Expenses	E	X	Valid	X	X	X	X
Z44	Other Income	E	X	Valid	X	X	X	X
Z45	Nonoperating Expenses	E	X	Valid	X	X	X	X
Z46	Transfers to General Fund – Gross	E	X	Valid	X	X	X	X
Z47	Expenditure for Licensing and Enforcing	E	X	Valid	X	X	X	X
Z48	Sales Taxes, Licenses, and Permits	E	X	Valid	X	X	X	X
Z51[1]	Total Ticket Sales	E	X	Valid	X[1]	X	X	X
Z52[1]	Prizes Awarded	E	X	Valid	X[1]	X	X	X
Z53[1]	Administrative Expenses	E	X	Valid	X[1]	X	X	X
Z54[1]	Proceeds Available	E	X	Valid	X[1]	X	X	X
Z62	Corporate Bonds, Federally-Spon. Agency	E	X	Valid	Valid	Valid	Valid	Valid

Appendix Table 2.1<br>**Code Validity, by Level and Type of Government** – page 11 of 11								
**Code**	**Code Title**	**Code Type**	**Federal**	**State**	**General Local**	**Special District**	**School District**	**Retirement System**
Z63	Corporate Bonds, Other	E	X	Valid	Valid	Valid	Valid	Valid
Z68	All Other Short-term Investments	E	X	Valid	Valid	Valid	Valid	Valid
Z70	Foreign and International Securities	E	X	Valid	Valid	Valid	Valid	Valid
Z71	Interest Earnings	E	X	Valid	Valid	Valid	Valid	Valid
Z72	Dividend Earnings	E	X	Valid	Valid	Valid	Valid	Valid
Z73	Other Investment Earnings	E	X	Valid	Valid	Valid	Valid	Valid
Z75	Active Members Employed, Local	D	X	Valid	X	X	X	Valid
Z76	Active Members Employed, State	D	X	Valid	X	X	X	Valid
Z77	Total Corporate Bonds	R	X	Valid	Valid	Valid	Valid	Valid
Z78	Corporate Stocks	R	X	Valid	Valid	Valid	Valid	Valid
Z81	Total Holdings and Investments	E	X	Valid	Valid	Valid	Valid	Valid
Z82	Total Other Investments	E	X	Valid	Valid	Valid	Valid	Valid
Z83	Other Securities	E	X	Valid	Valid	Valid	Valid	Valid
Z84	Investment Held in Trust by Other Agency	E	X	Valid	Valid	Valid	Valid	Valid
Z87	Time or Savings Deposits	E	X	Valid	Valid	Valid	Valid	Valid
Z88	Cash on Hand and Demand Deposits	E	X	Valid	Valid	Valid	Valid	Valid
Z89	Federal Treasury Securities	E	X	Valid	Valid	Valid	Valid	Valid
Z91	Losses on Investments	E	X	Valid	Valid	Valid	Valid	Valid
Z93	Administrative Expenses	E	Valid	Valid	Valid	Valid	Valid	Valid
Z95	Other Receipts NEC	E	X	Valid	Valid	Valid	Valid	Valid
Z96	Gains on Investments	E	X	Valid	Valid	Valid	Valid	Valid
Z98	Rentals from State Government	E	X	Valid	X	X	X	Valid
Z99	State Contributions to Own System	E	X	Valid	X	X	X	Valid
19T	Beginning Debt, Public Debt for Private	R	X	Valid	Valid	Valid	Valid	X
19U	Beginning Long-term Debt, Public	R	X	Valid	Valid	Valid	Valid	X
24T	Debt Issued, Public Debt for Private	R	X	Valid	Valid	Valid	Valid	X
29U	Debt Issued, Public Purpose	R	X	Valid	Valid	Valid	Valid	X
34T	Debt Retired, Public Debt for Private	R	X	Valid	Valid	Valid	Valid	X
39U	Debt Retired, Public Purpose	R	X	Valid	Valid	Valid	Valid	X
41I	Increase in Debt During Fiscal Year	R	Valid	X	X	X	X	X
41Q	Obligations of the U.S. Treasury	R	Valid	X	X	X	X	X
41V	Outstanding Held by Federal Accounts	R	Valid	X	X	X	X	X
41W	Obligations of Federal Agencies	R	Valid	X	X	X	X	X
41Y	Held Outside Federal Government	R	Valid	X	X	X	X	X
44T	Debt Outstanding, Public Debt for Private	R	X	Valid	Valid	Valid	Valid	X
49U	Debt Outstanding, Public Purpose	R	X	Valid	Valid	Valid	Valid	X
52T	Refunding Bonds Issues	E	X	Valid	X	X	X	X
53T	Debt Retired by Refunding	E	X	Valid	X	X	X	X
61V	Short-term Debt Beginning	R	X	Valid	Valid	Valid	Valid	X
64V	Short-term Debt Ending	R	X	Valid	Valid	Valid	Valid	X

Key:
Valid Statistics can be reported for these governments.
X Statistics cannot be reported for these governments.
Code Type
R = Regular Statistic
E = Exhibit Statistic
D = Descriptive Statistic

Notes:
[1]Valid code at the local government level for Washington, DC, only.
[2]Although valid for municipal governments, this code is not valid for Washington, DC, by definition.
[3]This code becomes valid for state governments effective with 2006 statistics.
[4]This code is valid for local government types 1, 2, 3, and 4, effective with 2006 statistics.

Appendix Table 2.2 Cross-Classification of Expenditure Function and Object Codes
page 1 of 2

This table combines the character and object categories with the various functions to derive those expenditure codes currently in use (excluding insurance trust expenditure codes). Note that data for the codes listed here ***may not be collected for all types of governments****. See Appendix Table 2.1 for data collection information.*

				Capital Outlay			Intergovernmental to -			
**Function Code**	**Current Operations (E)**	**Assistance and Subsidies (J)**	**Interest on Debt (I)**	**Construction (F)**	**Land and Existing Structures (G)**	**Equipment (K)**	**State Governments (L)**	**Local Governments (M)**	**School Districts (Q)**	**Federal Govern-ment (S)**
01	E01	J01	-	F01	G01	K01	L01	M01	-	-
02	E02	J02	-	F02	G02	K02	L02	M02	-	-
03	E03	-	-	F03	G03	K03	-	-	-	-
04	E04	J04	-	F04	G04	K04	L04	M04	-	-
05	E05	-	-	F05	G05	K05	L05	M05	-	-
06	E06	J06	-	F06	G06	K06	-	-	-	-
12	E12	-	-	F12	G12	K12	L12	M12	Q12	-
14	E14	J14	-	F14	G14	K14	-	-	-	-
16	E16	-	-	F16	G16	K16	-	-	-	-
18	E18	-	-	F18	G18	K18	L18	M18	Q18	-
19	-	J19	-	-	-	-	-	-	-	-
20	E20	J20	-	F20	G20	K20	L20	M20	-	-
21	E21	J21	-	F21	G21	K21	L21	M21	-	-
22	E22	J22	-	F22	G22	K22	L22	M22	-	-
23	E23	J23	-	F23	G23	K23	L23	M23	-	-
24	E24	-	-	F24	G24	-	-	M24	-	-
25	E25	I25	-	F25	G25	K25	L25	M25	-	-
26	E26	-	-	F26	G26	K26	-	-	-	-
28	E28	J28	-	F28	G28	K28	L28	M28	-	-
29	E29	J29	-	F29	G29	K29	L29	M29	-	-
30	-	-	-	-	-	-	-	M30	-	-
31	E31	-	-	F31	G31	K31	-	-	-	-
32	E32	J32	-	F32	G32	K32	L32	M32	-	-
36	E36	J36	-	F36	G36	K36	L36	M36	-	-
37	E37	J37	-	F37	G37	K37	L37	M37	-	-
39	E39	J39	-	F39	G39	K39	L39	M39	-	-
44	E44	J44	-	F44	G44	K44	L44	M44	-	-
45	E45	-	-	F45	G45	K45	-	-	-	-
47	E47	-	-	-	-	-	-	-	-	-
50	E50	J50	-	F50	G50	K50	L50	M50	-	-
51	E51	J51	-	-	-	-	L51	M51	-	-
52	E52	J52	-	F52	G52	K52	L52	M52	-	-

Appendix Table 2.2 Cross-Classification of Expenditure and Function Codes<br>page 2 of 2											
				**Capital Outlay**			**Intergovernmental to –**			
**Function Code**	**Current Operations (E)**	**Assistance and Subsidies (J)**	**Interest on Debt (I)**	**Construction (F)**	**Land and Existing Structures (G)**	**Equipment (K)**	**State Governments (L)**	**Local Governments (M)**	**School Districts (Q)**	**Federal Govern-ment (S)**
53	E53	J53	-	F53	G53	K53	L53	M53	-	-
55	E55	-	-	F55	G55	K55	-	M55	-	-
56	E56	J56	-	F56	G56	K56	L56	M56	-	-
57	E57	J57	-	F57	G57	K57	L57	M57	-	-
58	E58	J58	-	F58	G58	K58	L58	M58	-	-
59	E59	J59	-	F59	G59	K59	L59	M59	-	-
60	E60	-	-	F60	G60	K60	L60	M60	-	-
61	E61	J61	-	F61	G61	K61	L61	M61	-	-
62	E62	J62	-	F62	G62	K62	L62	M62	-	-
66	E66	-	-	F66	G66	K66	L66	M66	-	-
67	-	J67	-	-	-	-	L67	M67	-	S67
68	-	J68	-	-	-	-	-	M68	-	-
74	E74	-	-	-	-	-	-	-	-	S74
75	E75	-	-	-	-	-	-	-	-	-
77	E77	-	-	F77	G77	K77	-	-	-	-
79	E79	J79	-	F79	G79	K79	L79	M79	-	-
80	E80	-	-	F80	G80	K80	L80	M80	-	-
81	E81	-	-	F81	G81	K81	L81	M81	-	-
85	E85	J85	-	F85	G85	K85	-	-	-	-
87	E87	J87	-	F87	G87	K87	L87	M87	-	-
89	E89	J89	I89	F89	G89	K89	L89	M89	-	S89
90	E90	-	-	F90	G90	K90	-	-	-	-
91	E91	-	I91	F91	G91	K91	L91	M91	-	-
92	E92	-	I92	F92	G92	K92	L92	M92	-	-
93	E93	-	I93	F93	G93	K93	L93	M93	-	-
94	E94	-	I94	F94	G94	K94	L94	M94	-	-

Key:

\- Indicates that no code exists for that combination.

Appendix Table 2.3<br>Employment Code Validity, by Level and Type of Government						
**Code**	**Code Title**	**Federal**	**State**	**General Local**	**Special District**	**School District**
01	Air Transportation	Valid	Valid	Valid	Valid	X
02	Federal Space Research	Valid	X	X	X	X
05	Corrections	Valid	Valid	Valid	X	X
06	Nat. Defense/Int'National Relations	Valid	X	X	X	X
12	Elementary and Secondary Education	X	Valid	Valid	Valid	Valid
14	Federal Postal Service	Valid	X	X	X	X
18	Higher Education	X	Valid	Valid	X	Valid
21	Federal and State Other Education	Valid	Valid	X	X	X
22[1]	Social Insurance Administration	Valid	Valid	X[1]	X	X
23	Financial Administration	Valid	Valid	Valid	X	X
24	Local Fire Protection	X	X	Valid	Valid	X
25	Judicial and Legal	Valid	Valid	Valid	X	X
29	Central Staff Services	Valid	Valid	Valid	X	X
32	Health	Valid	Valid	Valid	Valid	X
36[2]	Hospitals	Valid	Valid	Valid	Valid	Valid
37	Federal Veterans Hospital	Valid	X	X	X	X
44	Highways	Valid	Valid	Valid	Valid	X
50	Housing and Community Development	Valid	Valid	Valid	Valid	X
52[3]	Libraries	Valid	Valid[3]	Valid	Valid	Valid
59	Natural Resources	Valid	Valid	Valid	Valid	X
61	Parks and Recreation	Valid	Valid	Valid	Valid	X
62	Police Protection	Valid	Valid	Valid	Valid	X
79	Public Welfare	Valid	Valid	Valid	Valid	X
80	Sewerage	X	Valid	Valid	Valid	X
81	Solid Waste Management	X	Valid	Valid	Valid	X
87	Sea and Inland Port Facilities	Valid	Valid	Valid	Valid	X
89	Other and Unallocable	Valid	Valid	Valid	Valid	X
90	Liquor Stores	X	Valid	X	X	X
91	Water Utilities	X	Valid	Valid	Valid	X
92	Electric Utilities	X	Valid	Valid	Valid	X
93	Gas Utilities	X	Valid	Valid	Valid	X
94	Transit Utilities	X	Valid	Valid	Valid	X

Key:
Valid Statistics can be reported for these governments.
X Statistics cannot be reported for these governments.

Notes:
[1]Applies to Washington, DC only for local governments.
[2]Public use files containing employment statistics also can use code 40 for this function.
[3]Applies to the state of Hawaii, only.

Topical Index

(Note: All references are to chapters and sections, unless otherwise indicated.)

Accounting:
- Accounting funds in finance statistics... 3.4.1
- Cash versus accrual basis for finance statistics... 3.4.2
- Examples of accounting funds – how classified... Table 3.2
- Governmental Accounting Standards Board... 3.4.3
  - GASB accounting funds in relation to indebtedness classification categories... 6.1.4

Adjustment and correction transactions – to finance statistics... 3.10.4, Chart 3.A
- Expenditure statistics adjustments... 5.1.2
- Refunds... 3.10.4
- Revenue statistics... 4.3.1.2

Agency transactions... 3.10.1

Average March earnings... 11.1.7

Bond banks... 6.4.4

Calculated statistics – generally... 2.3.4
- Expenditure statistics... 5.5 (description pages, see Z00 and V98)
- Public employment... 11.1.1
- Public employee retirement systems... 8.6 (description pages, see X06, X08, X11, X21, X30, X44, Z77, and Z82)

Capital leases... 3.11.2

Capital outlay:
- Defined... 5.2.2, 5.2.2.1, Chart 5.A, Table 5.1
- By type... 5.2.2.1, Table 5.1
  - Construction… 5.2.2.1
  - Other than construction… 5.2.2.1
    - Purchase of land and existing structures… 5.2.2.1
    - Equipment… 5.2.2.1

Cash and security holdings – definition... 7.1
- Assets out-of-scope... 7.1.1, Table 7.1
- Real property, special treatment of... 7.1.1, 8.6 (description pages, see X46)

Cash and security holdings:
- Description pages for classification categories... 7.7
  - For public employee retirement systems – detail 8.6
  - For other Federal and state social insurance trust systems... 9.7
- Federal Government cash and security holdings – explained... 7.4
- Historical revisions to classification.... Appendix 1.6.6, Appendix Table 1.1
- Liquor stores cash and security holdings... 7.5
- Lottery systems... 7.3
- Market value – conversion to... Appendix 1.6.6
- Public employee retirement systems... 8.4.3

Valuation... 8.3.3
Purchase and sale of investments... 8.4.4
Valid codes – by level and type of government... Table 7.2

Central collection...2.3.3

Character and object codes... 5.2.2, Table 5.1

Classification Manual:
Format changes for 2005 edition...Appendix 1.1

Compilation of statistics... 2.3.2

Current charges... 4.3.3
By type – description pages... 4.9

Current dollars – use of... 3.3

Debt... see Indebtedness

Dependent agencies – explained... 1.4

Depreciation... 3.11.3

Derived statistics:
Expenditures... 5.5
Explained... 2.2.3
Indebtedness...6.3
Liquor stores... 10.4

Description pages for classification categories:
Cash and security holdings... 7.7
Employment functions... 12.3
Finance expenditure functions... 5.6
Finance revenue categories... 4.9
Finance indebtedness categories... 6.3
Finance cash and security categories... 7.7
Liquor stores... 10.5
Lottery systems... 10.10
Public employee retirement systems... 8.6
Social insurance trust systems – other than for public employee retirement systems... 9.7

Descriptive statistics:
Explained... 2.2.4
Public employee retirement systems... 8.2, 8.6 (description pages, see Z01-Z12, Z75, Z76, V01-V07), Table 8.2

Direct expenditure – defined... 5.2.2.1

Employees – defined...11.1.2
Full-time... 11.1.3
Part-time... 11.1.3

Employment functions:
Generally... 12.1
Description pages for... 12.3

Employment statistics – generally... 11.1.1
Average March earnings... 11.1.7
Description pages for classification categories... 12.3
Full-time equivalent... 11.1.6
Hours worked by part-time workers... 11.1.5
Part-time employees... 11.1.3
Pay interval... 11.1.9
Payrolls... 11.1.4
Revisions to classification categories... Appendix 1.2
Special topics during census of governments...11.6
Standard hours worked... 11.1.8
Valid codes – by level and type of government... Table 12.2

Exhibit statistics:
Explained... 2.2.2
Expenditure... 5.5
Indebtedness... 6.3
Liquor stores... 10.4
Public employee retirement systems... 8.2, Table 8.2
Revenue and receipts... 4.7

Expenditure – definition... 5.1, 5.1.1
Exclusions from definition – out-of-scope... 5.1.1

Expenditure:
Capital outlay... 5.2.2.1
Construction... 5.2.2.1, Table 5.1
Other than construction... 5.2.2.1, Table 5.1
Equipment
Purchase of land and existing structures
Character and object codes - explained... 5.2.2, Table 5.1
Cross classification of expenditure functions with character and object codes... Appendix Table 2.2
Derived expenditure statistics – explained... 5.5
Description pages for expenditure classification categories... 5.6
Direct expenditure – defined... 5.2.2.1
Exhibit expenditure statistics – explained... 5.5
Functional categories for expenditure - defined... 5.2.1, 5.6
Federal government expenditure functions – unique categories... 5.6.2
General expenditure... 5.3.1
Intergovernmental expenditure – defined... 5.2.2.2, Table 5.1
Liquor stores expenditure... 5.3.3
Object codes... 5.2.2, Table 5.1
Refunds and correcting transactions... 5.1.2
Regular expenditure statistics - explained... 5.5
Social insurance trust expenditure... 5.3.4
Utility expenditure... 5.3.2
Intergovernmental expenditure codes for utilities... 5.3.2.1
Valid codes – by level and type of government... Table 5.2
Valuation – how measured... 5.4.1, 5.4.2, 5.4.3

Federal Government:
Cash and security holdings of ... 7.4
Employment statistics... 11.2
Employment by function... 11.2.2
Employment by state area... 11.2.1
Expenditure statistics... 5.6.2
Indebtedness statistics... 6.3
Social insurance trust systems of... 9.2.4

Fiscal condition of governments:
Statistical nature of the data... 3.12

Fiscal years – generally... 3.2, Table 3.1
Cash and security holdings statistics... 7.2.1
Expenditure statistics... 5.4.1
Indebtedness statistics... 6.2.1
Revenue statistics... 4.1.2.2

Four sectors of government... 2.1, summary chart 2.1.5
Expenditure statistics – issues... 5.3
Finance statistics – generally... 3.5
General sector... 2.1.1
Indebtedness statistics – issues...6.4.7
Liquor stores sector... 2.1.3
Revenue statistics – issues... 4.2
Social insurance trust sector... 2.1.4
Utilities sector... 2.1.2

Full-time employees... 11.1.3

Full-time equivalent employment, methodology for calculating... 11.1.6
Pre-1986 methodology... 11.1.6.1

Functional categories:
Employment statistics... 12.1, 12.3, table 12.1
Finance expenditure statistics...5.6

Funds, type of:
Accounting funds – types... 3.4.1
Accounting funds – how classified... Table 3.2
Agency transaction funds... 3.10.1
Governmental Accounting Standards Board funds and debt categories... 6.1.4
Revolving funds... 3.9.2

General expenditure – defined... 5.3.1

General government sector... 2.1.1
Revenue statistics... 4.3

General revenue – defined... 4.3

GID – see Governments Integrated Directory

Governmental Accounting Standards Board (GASB)... 3.4.3
Indebtedness statistics and types of debt... 6.1.4
Unrealized gains and losses... 8.4.1.1

Government enterprises:
Revenue statistics for... 4.1.2.4

Government organization reference codes... 1.8
Activity codes... 1.8.3, Table 1.1
Function codes... 1.8.2, Table 1.1, Table 1.2
School level codes... 1.8.4

Government unit code... 1.8.1
Historical assignments... 1.8.1.1

Governments:
Definition... 1.2
Factors used in defining... 2.1
Noncritical characteristics... 1.2.2
Reporting entity for debt statistics.... 6.1.3, 6.1.4
Types of... 1.1
Dependent agencies... 1.4
Parent government... 1.3

Governments Integrated Directory... 1.7

Historical revisions to classification categories and statistics:
2005 redesign of government finance classification system... Appendix 1.6
Assistance and subsidies... Appendix 1.6.1.1
Cash and security holdings – market value… Appendix 1.6.6, Appendix Table 1.1
Expenditure classification categories... Appendix 1.6.4
Full-time equivalent employment – calculation of... 11.1.6.1
Indebtedness... 6.1.6, Appendix 1.6.5, Table 6.2
Liquor stores... Appendix 1.6.2
Lottery systems... Appendix 1.6.2
Object codes... Appendix 1.6.1, Appendix 1.6.1.2, Appendix 1.6.1.3
Public employee retirement systems – classification code revisions... Appendix 1.5, Appendix Table 1.2
Public employment statistics... Appendix 1.5
Revenue classification categories... Appendix 1.6.3
Summary of revisions to finance classifications – 1988 to 2005... Appendix 1.3
Washington DC – summary of classification changes for... Appendix 1.7

Hours worked by part-time workers... 11.1.5

Imputation... 2.3.5

Indebtedness – definition... 6.1, 6.1.2, Chart 6.A
Liabilities in-scope... 6.1.1, Chart 6.A
Liabilities out-of-scope... 6.1.2, Chart 6.A

Indebtedness:
Advanced refunding... 6.4.2.2
Bond banks and pooled debt... 6.4.4

Classification changes effective with 2005 survey year... 6.1.6, Table 6.2, Appendix 1.6.5
Debt instruments... 6.1.1
Description pages for debt classification categories... 6.3
Four sectors of government – debt issues... 6.4.7
Governmental Accounting Standards Board... 6.1.4
GASB funds in relation to Census Bureau debt statistics... 6.1.4
Historical changes to debt categories... Table 6.2, Appendix 1.6.5
Leases and lease purchase agreements... 6.4.5
Long-term debt... 6.1.5, 6.3
Public debt for private purposes... 6.4.1
Refunding... 6.4.2, 6.4.2.1
Short-term debt... 6.1.1
Taxable public debt... 6.4.6
Three-way classification of debt... 6.1.5
Valid codes – by level and type of government... Table 6.1
Valuation methodology ... 6.2.2
Zero coupon bonds... 6.4.3

Interdepartmental charges... 3.9.3
Interfund transactions... 3.9.1
Intragovernmental service funds... 3.9.2
Revolving funds... 3.9.2

Intergovernmental – fiscal transactions in general... 3.7
Intergovernmental expenditure... 5.2.2.2
Intergovernmental expenditure codes for utilities... 5.3.2.1
Intergovernmental revenue... 4.3.2
Intergovernmental revenue codes for utilities... 4.5.1
Fiscal agents for other governments... 3.7.2
Netting out – in finance statistics... 3.8
Payments to other government social insurance trust system...3.8.1
Purchases from other governments... 3.7.3

Internal transfers (intragovernmental transactions)... 3.9

Investment transactions:
Investment transactions – how classified, generally... 3.10.3
Public employee retirement systems... 3.10.3.1

Joint activities of governments... 1.5
Government finance statistics... 3.14
Joint utility projects... 5.3.2.2
Public employment statistics... 11.4

Liquor stores – definition... 10.2

Liquor stores sector... 2.1.3
Derived statistics... 10.4
Description pages for classification categories... 10.5
Exhibit statistics...10.4
Expenditures...5.3.3
Expenses...10.4
Historical changes to classification categories... Appendix 1.6.2
Overview... 10.1

Receipts... 10.4
Revenue... 4.4
Summary of classification codes... Table 10.2
Valid codes – by level and type of government... Table 10.1

Lottery systems – definition... 10.7

Lottery systems:
Cash and security holdings... 7.3, 10.8.1
Description pages for classification categories... 10.10
Exhibit statistics... 10.9
Expenses... 10.9
Historical revisions to classification categories... Appendix 1.6.2
Net lottery revenue... 10.8
Overview... 10.6
Receipts... 10.9
Summary of classification codes... Table 10.2
Treatment in regular finance statistics... 10.8
Valid codes – by level and type of government... Table 10.1

Mail canvass... 2.3.1

Market value:
Cash and security holdings – generally... 7.2.2
Public employee retirement system – cash and security holdings ... Appendix 1.6.6

Measurement issues:
Cash and security holdings... 7.2
Valuation of... 7.2.2
Current dollars... 3.3
Expenditure... 5.4
Timing... 5.4.1
Indebtedness... 6.2
Timing... 6.2.1
Market valuation of cash and security holdings... 7.2.2
Other Federal and state social insurance trust systems... 9.4
Aggregation... 9.4.1
Timing... 9.4.2
Valuation... 9.4.3
Public employee retirement systems... 8.3
Aggregation... 8.3.2
Timing... 8.3.1
Unfunded liabilities... 8.4.7
Valuation of assets... 8.3.3
Revenue... 4.1.2
Aggregation... 4.1.2.3
Government enterprises... 4.1.2.4
Timing... 4.1.2.2

Methodology:
Average March earnings – employment statistics... 11.1.7
Full-time equivalent employment – calculation... 11.1.6, 11.1.6.1,
Statistical aggregation and tabulation – generally... 1.6.1
Statistical nature of the data.... 3.12

Valuation of finance statistics:
Cash and security holdings
Expenditure... 5.4.2
Indebtedness

Miscellaneous general revenue... 4.3.4
By type – description pages... 4.9

Noncash transactions – how classified in finance statistics... 3.6
Object codes – for expenditure statistics:
Classification categories... 5.2.2, Table 5.1
Historical revisions to... Appendix 1.6.1
Use of object code “K”... Table 5.1, Appendix 1.6.1.3

Operating leases... 3.11.2

Other Federal and state social insurance trust systems – defined... 9.2.4
Description pages for classification categories... 9.7

Parent government... 1.3

Part-time employees... 11.1.3

Pay interval... 11.1.9

Payrolls... 11.1.4

Private trust transactions... 3.10.2

Property taxes – defined 4.9 (description pages, see T01)
Assignment of taxes to government units... 4.3.1.1

Public debt for private purposes... 6.4.1

Public employee retirement systems:
Defined... 8.1
In-scope... 8.1.1
Out-of-scope... 8.1.2
Description pages for classification categories... 8.6
Descriptive statistics of... 8.2, Table 8.2
Exhibit statistics of... 8.2, Table 8.2
Federal Government statistics... 8.5
Finances of... 8.4
Cash and security holdings... 7.7, 8.4.3
Expenses versus expenditure... 8.4.2
Receipts versus revenue... 8.4.1
Unrealized gains and losses... 8.4.1.1
Fiscal condition of... 8.4.8
Historical revisions to classification codes... Appendix 1.5, Appendix Table 1.2
Investment transactions of... 3.10.3.1
Internal investment transactions... 8.4.5
Loans to members... 8.4.6.1
Loans to parent government... 8.4.6.2
Purchase and sale of investments... 8.4.4

Summary of all codes, regular, descriptive, and exhibit... Table 8.2
Fiscal Conditions of Retirement Systems... 8.4.7
Valid codes by level and type of government... Table 8.1, see also Appendix 2
Valuation of cash and security holdings... 8.3.3

Real property – classification of... 7.1.1, 8.6 (description pages, see X46)

Refunds – how classified... 3.10.4
Expenditure statistics - how adjusted... 5.1.2
Revenue statistics – how adjusted... 4.1.2.1
Taxes – refunds of... 4.3.1.2

Regular statistics, explained... 2.2.1
Expenditure – generally... 5.5

Reporting periods:
Employment statistics...11.3
Employee retirement statistics... 8.3.1
Finance statistics, generally... 3.2
Fiscal years, defined... 3.2

Revenue – definition... 4.1
Exclusions – out-of scope transactions... 4.1.3

Revenue:
By type... see specific category, such as taxes
Coverage issues... 4.1.1
Current charges... 4.3.3
Descriptions pages for revenue classification categories... 4.9
Exhibit statistics for revenue... 4.7
General revenue... 4.3
Intergovernmental revenue... 4.3.2
Liquor stores revenue... 4.4
Measurement issues:
Refunds and correcting transactions... 4.1.2.1
Miscellaneous general revenue... 4.3.4
Revenue versus receipts... 4.7
Social insurance trust revenue... 4.6
Statistical codes for revenue – explained... 4.8
Taxes... 4.3.1
Utility revenue... 4.5
Intergovernmental revenue codes for utilities... 4.5.1
Valid codes – by level and type of government... Table 4.1, Table 4.2, Table 4.3

Sales taxes – generally... 4.9

Social Insurance Trust System – defined... 9.1
Examples of social insurance trust systems included – in-scope... 9.1.1
Examples of social insurance trust systems excluded – out-of-scope... 9.1.2

Social insurance trust systems:
Cash and security holdings... 7.7, 9.5.3
Debt of unemployment compensations systems – how classified... 9.6.1

Description pages for classification categories:
Public employee retirement systems... 8.6
Other Federal and state social insurance trust systems... 9.7
Exhibit statistics of... 9.3
Expenditure... 5.3.4, 9.5.2
Expenditure and expenses – basic concepts... 9.5.2
Internal investment transactions... 9.6.3
Loans to parent government... 9.6.2
Other Federal and state social insurance trust systems... 9.2.4, see also own topical heading
Public employee retirement systems... 8.1, 9.2.1, see also own topical heading
Revenue... 4.6, 9.5.1
Revenue and receipts – basic concepts... 9.5.1
Sector of government... 2.1.4
Unemployment compensation systems... 9.2.2, see also own topical heading
Valid codes by level and type of government... Table 9.1
Valuation of financial measures... 9.4.3
Workers' Compensation Systems... 9.2.3, see also under own topical heading

Special topics:
Assets of state lottery systems... 7.3
Assignment of tax revenue by government... 4.3.1.1
Classifying debt for unemployment compensations funds... 9.6.1
Debt statistics and the four sectors of government... 6.4.7
Internal investment transactions of social insurance trust systems... 9.6.3
Federal Government cash and security holdings... 7.4
Government unit code historical assignments... 1.8.1.1
How Census Bureau statistics of governments are developed... 2.3
How lottery system assets are reported in regular finance statistics... 10.8.1
Intergovernmental expenditure codes for utilities... 5.3.2.1
Intergovernmental revenue codes for utilities... 4.5.1
Internal investment transactions... 8.4.5
Joint utility projects... 5.3.2.2
Liquor stores cash and security holdings... 7.5
Loans to parent government by social insurance trust systems... 9.6.2
Fiscal Conditions of Retirement Systems... 8.4.7
Payments to other government social insurance trust systems... 3.8.1

Standard hours worked... 11.1.8

Statistical aggregation and tabulation... 1.6.1

Surplus and deficit... see fiscal conditions of governments

Suspense transactions – how classified... 3.11.1

Taxes – defined... 4.3.1
Assignment of tax revenue by government... 4.3.1.1
Descriptioin pages – by type... 4.9
On government utilities... 4.3.1.3
Refunds of taxes... 4.3.1.2

Types of statistics... 2.2
Derived statistics... 2.2.3
Descriptive statistics... 2.2.4

Exhibit statistics... 2.2.2
Regular statistics... 2.2.1

Unemployment compensation systems – defined... 9.2.2
Debt for unemployment compensation systems... 9.6.1
Description pages for classification categories... 9.7

Unrealized gains and losses... 8.4.1.1

Utilities sector... 2.1.2
Revenue... 4.5
Intergovernmental revenue codes for utilities... 4.5.1
Expenditure... 5.3.2
Intergovernmental expenditure codes for utilities... 5.3.2.1

Valid code tables (by level and type of government) – see also Appendix 2:
Cash and security holdings... Table 7.2
Employment statistics... Table 12.2
Expenditure statistics... Table 5.2
Indebtedness statistics... Table 6.1
Public employee retirement statistics... Table 8.1
Revenue statistics:
General revenue... Table 4.1
Liquor stores revenue... Table 4.2
Utility revenue... Table 4.2
Social insurance trust revenue... Table 4.3

Washington DC:
Classification as a government unit... 3.13
Employment statistics classification issues... 11.5
Finance statistics classification issues... 3.13
Lottery system statistics... 10.11
Unemployment compensation system statistics... 9.2.2

Workers' Compensation Systems – defined... 9.2.3
Description pages for classification categories... 9.7

Zero coupon bonds... 6.4.3
