# Supplementary material for: The Government Finance Database: A Common Resource for Quantitative Research in Public Financial Analysis
Source: PLoS One. 2015 Jun 24;10(6):e0130119. doi: 10.1371/journal.pone.0130119 (PMC4479543; doi:10.1371/journal.pone.0130119)
Supplement: S2 File — This file includes the state data from the Government Finance Database at the time of publication, the appendix for the Government Finance Database, and the U.S. Census 2006 Classification manual. For more up to date versions of the data please visit http://www.willamette.edu/mba/research_impact/public_datasets/. (ZIP) [file pone.0130119.s002.zip › Appendix For The Government Finance Database.pdf]

Appendix A: Replication of Data Consolidation

Instructions for replicating the the process of data organization we followed.

- Obtain the relevant files from the census: four (4) “fin_data” files from 2008 – 2011, and one (1) “IndFin_1967-2007.zip” file.
    - (Contact information is available from: http://www.census.gov/govs/local/)
- Extract these files to a directory on your computer
- Make sure that you have at least 20GB of space available on the drive where your SAS work directory is located, since the data files stored in memory get very large during this process.
- Paste the code from appendix C into a SAS program file.
- Change the directories referenced in the code so that they will work on your machine. There are several locations for these:
    - Line 4
        - Make sure to include the final “/IndFin0” after the file path pointing to the 1967-2007 folder.
    - Line 46
        - Make sure to include the final “/IndFin” (different from above) after the file path pointing to the 1967-2007 folder.
    - Line 114
        - This should point to the data file containing the 2011 data, the text “2011FinEstDAT_ALL5modp_pu.txt” fits the current census data, but these naming conventions change regularly.
    - Line 142
        - This should point to the file containing the 2011 government ID information
    - Line 168
        - Points to the 2010 financial data text file.
    - Line 196
        - Points to the 2010 government ID file.
    - Line 222
        - Points to the 2009 financial data text file.
    - Line 250
        - Points to the 2009 government ID file.
    - Line 276
        - Points to the 2008 financial data text file.
    - Line 304
        - Points to the 2008 government ID file.
    - Line 1189
        - This points to the location and file name where the final comma separated data file should be exported and saved.
- Run the SAS program, it should output a csv file with the consolidated data to the location you indicated on line 1189.

Appendix B: Mapping Natural Language Variables to Census Data Codes

In table A1 we display the census codes used to create each of the database variables, or present a formula in cases where the variable is computed from other database variables. Variables listed as "." Do not have a data code in the newest census data and will not have complete coverage in the database as a result. IGR stands for "intergovernmental revenue", NEC stands for "not elsewhere classified", LTD stands for "long term debt", FFC stands for "full faith and credit", and NG stands for "not guaranteed".

TABLE A1
Corresponding Census Codes or Formulas for Each Database Variable

Database Variable Name	Census Data Codes or Formula
Total_Revenue	B01, B21, B22, B30, B42, B46, B50, B59, B79, B80, B89, B91, B92, B93, B94, C21, C30, C42, C46, C50, C79, C80, C89, C91, C92, C93, C94, D21, D30, D42, D46, D50, D79, D80, D89, D91, D92, D93, D94, T01, T09, T10, T11, T12, T13, T14, T15, T16, T19, T20, T21, T22, T23, T24, T25, T27, T28, T29, T40, T41, T50, T51, T53, T99, A01, A03, A09, A10, A12, A16, A18, A21, A36, A44, A45, A50, A56, A59, A60, A61, A80, A81, A87, A89, U01, U11, U20, U21, U30, U40, U41, U50, U95, U99, A90, A91, A92, A93, A94, X01, X02, X05, X08, Y01, Y02, Y04, Y11, Y12, Y51, Y52
Total_Rev_Own_Sources	T01, T09, T10, T11, T12, T13, T14, T15, T16, T19, T20, T21, T22, T23, T24, T25, T27, T28, T29, T40, T41, T50, T51, T53, T99, A01, A03, A09, A10, A12, A16, A18, A21, A36, A44, A45, A50, A56, A59, A60, A61, A80, A81, A87, A89, U01, U11, U20, U21, U30, U40, U41, U50, U95, U99, A90, A91, A92, A93, A94, X01, X02, X05, X08, Y01, Y02, Y04, Y11, Y12, Y51, Y52
General_Revenue	B01, B21, B22, B30, B42, B46, B50, B59, B79, B80, B89, B91, B92, B93, B94, C21, C30, C42, C46, C50, C79, C80, C89, C91, C92, C93, C94, D21, D30, D42, D46, D50, D79, D80, D89, D91, D92, D93, D94, T01, T09, T10, T11, T12, T13, T14, T15, T16, T19, T20, T21, T22, T23, T24, T25, T27, T28, T29, T40, T41, T50, T51, T53, T99, A01, A03, A09, A10, A12, A16, A18, A21, A36, A44, A45, A50, A56, A59, A60, A61, A80, A81, A87, A89, U01, U11, U20, U21, U30, U40, U41, U50, U95, U99
Gen_Rev_Own_Sources	A01, A03, A09, A10, A12, A16, A18, A21, A36, A44, A45, A50, A56, A59, A60, A61, A80, A81, A87, A89, T01, T09, T10, T11, T12, T13, T14, T15, T16, T19, T20, T21, T22, T23, T24, T25, T27, T28, T29, T40, T41, T50, T51, T53, T99, U01, U11, U20, U21, U30, U40, U41, U50, U95, U99
Total_Taxes	T01, T09, T10, T11, T12, T13, T14, T15, T16, T19, T20, T21, T22, T23, T24, T25, T27, T28, T29, T40, T41, T50, T51, T53, T99
Property_Tax	T01
Tot_Sales___Gr_Rec_Tax	T09, T10, T11, T12, T13, T14, T15, T16, T19
Total_Gen_Sales_Tax	T09
Total_Select_Sales_Tax	T10, T11, T12, T13, T14, T15, T16, T19
Alcoholic_Beverage_Tax	T10

Amusement_Tax	T11
Insurance_Premium_Tax	T12
Motor_Fuels_Tax	T13
Pari_mutuels_Tax	T14
Public_Utility_Tax	T15
Tobacco_Tax	T16
Other_Select_Sales_Tax	T19
Total_License_Taxes	T20 , T21 , T22 , T23 , T24 , T25 , T27 , T28 , T29
Alcoholic_Beverage_Lic	T20
Amusement_License	T21
Corporation_License	T22
Hunting___Fishing_License	T23
Motor_Vehicle_License	T24
Motor_Veh_Oper_License	T25
Motor_Vehicle_License_Total	T24, T25
Public_Utility_License	T27
Occup_and_Bus_Lic_NEC	T28
Other_License_Taxes	T29
Total_Income_Taxes	T40 , T41 , T50 , T51 , T53 , T99
Individual_Income_Tax	T40
Corp_Net_Income_Tax	T41
Death_and_Gift_Tax	T50
Docum_and_Stock_Tr_Tax	T51
Severance_Tax	T53
Taxes_NEC	T99
Total_IG_Revenue	B01, B21, B22, B30, B42, B46, B50, B59, B79, B80, B89, B91, B92, B93, B94, C21, C30, C42, C46, C50, C79, C80, C89, C91, C92, C93, C94, D21, D30, D42, D46, D50, D79, D80, D89, D91, D92, D93, D94
Total_Fed_IG_Revenue	B01, B21, B22, B30, B42, B46, B50, B59, B79, B80, B89, B91, B92, B93, B94
Fed_IGR_Air_Transport	B01
Fed_IGR_Education	B21
Fed_IGR_Emp_Sec_Adm	B22
Fed_IGR_Gen_Rev_Shar	.
Fed_IGR_Gen_Support	B30
Fed_IGR_Health___Hos	B42
Fed_IGR_Highways	B46
Fed_IGR_Transit_Sub	B94
Fed_IGR_Hous_Com_Dev	B50
Fed_IGR_Natural_Res	B59
Fed_IGR_Public_Welf	B79
Fed_IGR_Sewerage	B80
Fed_IGR_Other	B89
Total_State_IG_Revenue	C21, C30, C42, C46, C50, C79, C80, C89, C91, C92, C93, C94
State_IGR_Education	C21
State_IGR_Tax_Relief	.
State_IGR_Oth_Gen_Sup	C30
State_IGR_Gen_Sup	State_IGR_Oth_Gen_Sup, State_IGR_Tax_Relief
State_IGR_Health___Hos	C42
State_IGR_Highways	C46

State_IGR_Transit_Sub	C94
State_IGR_Hous_Com_Dev	C50
State_IGR_Public_Welf	C79
State_IGR_Sewerage	C80
State_IGR_Other	C89
Tot_Local_IG_Rev	D21, D30, D42, D46, D50, D79, D80, D89, D91, D92, D93, D94
Local_IGR_InterSchool_Aid	D11
Local_IGR_Other_Education	D21
Local_IGR_Oth_Gen_Sup	D30
Local_IGR_Health___Hos	D42
Local_IGR_Highways	D46
Local_IGR_Transit_Sub	D94
Local_IGR_Hous_Com_Dev	D50
Local_IGR_Public_Welf	D79
Local_IGR_Sewerage	D80
Local_IGR_Other	D89
Tot_Chgs_and_Misc_Rev	A01, A03, A09, A10, A12, A16, A18, A21, A36, A44, A45, A50, A56, A59, A60, A61, A80, A81, A87, A89, U01, U11, U20, U21, U30, U40, U41, U50, U95, U99
Total_General_Charges	A01, A03, A09, A10, A12, A16, A18, A21, A36, A44, A45, A50, A56, A59, A60, A61, A80, A81, A87, A89
Chg_Air_Transportation	A01
Chg_Misc_Com_Activ	A03
Chg_Total_Education	A09, A10, A12, A16, A18, A21
Chg_Total_Elem_Education	Chg_Elem_Ed_Sch_Lunch, Chg_Elem_Ed_Tuition, Chg_Elem_Ed_NEC
Chg_Elem_Ed_Sch_Lunch	A09
Chg_Elem_Ed_Tuition	A10
Chg_Elem_Ed_NEC	A12
Chg_Total_High_Ed	A16, A18
Chg_Hospitals	A36
Chg_Highways	Chg_Regular_Highways, Chg_Toll_Highways
Chg_Regular_Highways	A44
Chg_Toll_Highways	A45
Chg_Housing___Comm_Dev	A50
Chg_Total_Nat_Res	A56 , A59
Chg_Parking	A60
Chg_Parks___Recreation	A61
Chg_Sewerage	A80
Chg_Solid_Waste_Mgmt	A81
Chg_Water_Transport	A87
Chg_All_Other_NEC	A89
Misc_General_Revenue	U01, U11, U20, U21, U30, U40, U41, U50, U95, U99
Special_Assessments	U01
Prop_Sale_Total	Prop_Sale_Hous_Com_Dev, Prop_Sale_Other
Prop_Sale_Hous_Com_Dev	.
Prop_Sale_Other	U11
Interest_Revenue	U20
Fines_and_Forfeits	U30
Rents_and_Royalties	U40 , U41

Net_Lottery_Revenue	U95
Misc_General_Rev_NEC	U99
Liquor_Stores_Revenue	A90
Total_Utility_Revenue	A91, A92, A93, A94
Water_Utility_Revenue	A91
Electric_Utility_Rev	A92
Gas_Utility_Rev	A93
Transit_Utility_Rev	A94
Total_Insur_Trust_Rev	X01, X02, X05, X08, Y01, Y02, Y04, Y11, Y12, Y51, Y52
Total_Insur_Trust_Ctrb	X01, X02, X05, Y01
Tot_Ins_Trust_Inv_Rev	X08 , Y02
Total_Emp_Ret_Rev	X01, X02, X05, X08
Emp_Ret_Total_Ctrib	X01, X02, X05
Emp_Ret_Loc_Emp_Ctrib	X01
Emp_Ret_Loc_To_Loc_Sys	X04
Emp_Ret_From_Other_Gov	X05
Emp_Ret_Sta_To_Sta_Ctr	X06
Emp_Ret_Int_Rev	X08
Emp_Ret_Other_Earnings	.
Total_Unemp_Rev	Y01, Y02, Y04
Unemp_Payroll_Tax	Y01
Unemp_Int_Revenue	Y02
Unemp_Federal_Advances	Y04
Total_Expenditure	E01, E03, E04, E05, E12, E16, E18, E21, E22, E23, E24, E25, E26, E29, E31, E32, E36, E44, E44, E45, E50, E52, E55, E56, E59, E60, E61, E62, E66, E74, E75, E77, E79, E80, E81, E85, E87, E89, E90, E91, E92, E93, E94, I89, I91, I92, I93, I94, J19, J67, J68, J85, X11, X12, Y05, Y06, Y14, Y53, F01, F03, F04, F05, F12, F16, F18, F21, F22, F23, F24, F25, F26, F29, F31, F32, F36, F44, F45, F50, F52, F55, F56, F59, F60, F61, F62, F66, F77, F79, F80, F81, F85, F87, F89, F90, F91, F92, F93, F94, G01, G03, G04, G05, G12, G16, G18, G21, G22, G23, G24, G25, G26, G29, G31, G32, G36, G44, G45, G50, G52, G55, G56, G59, G60, G61, G62, G66, G77, G79, G80, G81, G85, G87, G89, G90, G91, G92, G93, G94, L01, L04, L05, L12, L18, L23, L25, L29, L32, L36, L44, L52, L59, L60, L61, L62, L66, L67, L79, L80, L81, L87, L89, L91, L92, L93, L94, M01, M04, M05, M12, M18, M21, M23, M24, M25, M29, M30, M32, M36, M44, M50, M52, M55, M56, M59, M60, M61, M62, M66, M67, M68, M79, M80, M81, M87, M89, M91, M92, M93, M94, Q12, Q18, S67, S74, S89
Total_IG_Expenditure	L01, L04, L05, L12, L18, L23, L25, L29, L32, L36, L44, L50, L52, L59, L60, L61, L62, L66, L67, L79, L80, L81, L87, L89, L91, L92, L93, L94, M01, M04, M05, M12, M18, M21, M23, M24, M25, M29, M30, M32, M36, M44, M50, M52, M52, M55, M56, M59, M60, M61, M62, M66, M67, M68, M79, M80, M81, M87, M89, M91, M92, M93, M94, Q12, Q18, S67, S89
Direct_Expenditure	E01, E03, E04, E05, E12, E16, E18, E21, E22, E23, E24, E25, E26, E29, E31, E32, E36, E44, E45, E50, E52, E55, E56, E59, E60, E61, E62, E66, E74, E75, E77, E79, E80, E81, E85, E87, E89, E90, E91, E92, E93, E94, F01, F03, F04, F05, F12, F16,

	F18, F21, F22, F23, F24, F25, F26, F29, F31, F32, F36, F44, F45, F50, F52, F55, F56, F59, F60, F61, F62, F66, F77, F79, F80, F81, F85, F87, F89, F90, F91, F92, F93, F94, G01, G03, G04, G05, G12, G16, G18, G21, G22, G23, G24, G25, G26, G29, G31, G32, G36, G44, G45, G50, G52, G55, G56, G59, G60, G61, G62, G66, G77, G79, G80, G81, G85, G87, G89, G90, G91, G92, G93, G94, X11, X12, Y05, Y06, Y14, Y53, J19, J67, J68, J85, I89, I91, I92, I93, I94
Total_Current_Expend	Total_Expenditure, - Total_Capital_Outlays
Total_Current_Oper	E01, E03, E04, E05, E12, E16, E18, E21, E22, E23, E24, E25, E26, E29, E31, E32, E36, E44, E45, E50, E52, E55, E56, E59, E60, E61, E62, E66, E74, E75, E77, E79, E80, E81, E85, E87, E89, E90, E91, E92, E93, E94
Total_Capital_Outlays	F01, F03, F04, F05, F12, F16, F18, F21, F22, F23, F24, F25, F26, F29, F31, F32, F36, F44, F45, F50, F52, F55, F56, F59, F60, F61, F62, F66, F77, F79, F80, F81, F85, F87, F89, F90, F91, F92, F93, F94, G01, G03, G04, G05, G12, G16, G18, G21, G22, G23, G24, G25, G26, G29, G31, G32, G36, G44, G45, G50, G52, G55, G56, G59, G60, G61, G62, G66, G77, G79, G80, G81, G85, G87, G89, G90, G91, G92, G93, G94
Total_Construction	F01, F03, F04, F05, F12, F16, F18, F21, F22, F23, F24, F25, F26, F29, F31, F32, F36, F44, F45, F50, F52, F55, F56, F59, F60, F61, F62, F66, F77, F79, F80, F81, F85, F87, F89, F90, F91, F92, F93, F94
Total_Other_Capital_Outlays	Total_Capital_Outlays, -Total_Construction
Tot_Assist___Subsidies	J19, J67, J68, J85
Total_Interest_on_Debt	I89, I91, I92, I93, I94
Total_Insur_Trust_Ben	X11, X12, Y05, Y06, Y14, Y53
Total_Salaries___Wages	Z00
General_Expenditure	E01, E03, E04, E05, E12, E16, E18, E21, E22, E23, E24, E25, E26, E29, E31, E32, E36, E44, E45, E50, E52, E55, E56, E59, E60, E61, E62, E66, E74, E75, E77, E79, E80, E81, E85, E87, E89, E90, E91, E92, E93, E94, F01, F03, F04, F05, F12, F16, F18, F21, F22, F23, F24, F25, F26, F29, F31, F32, F36, F44, F45, F50, F52, F55, F56, F59, F60, F61, F62, F66, F77, F79, F80, F81, F85, F87, F89, F90, F91, F92, F93, F94, G01, G03, G04, G05, G12, G16, G18, G21, G22, G23, G24, G25, G26, G29, G31, G32, G36, G44, G45, G50, G52, G55, G56, G59, G60, G61, G62, G66, G77, G79, G80, G81, G85, G87, G89, G90, G91, G92, G93, G94, X11, X12, Y05, Y06, Y14, Y53, J19, J67, J68, J85, I89, I91, I92, I93, I94
IG_Exp_To_State_Govt	L01, L04, L05, L12, L18, L23, L25, L29, L32, L36, L44, L50, L52, L59, L60, L61, L62, L66, L67, L79, L80, L81, L87, L89, L91, L92, L93, L94
IG_Exp_To_Local_Govts	M01, M04, M05, M12, M18, M21, M23, M24, M25, M29, M30, M32, M36, M44, M50, M52, M52, M55, M56, M59, M60, M61, M62, M66, M67, M68, M79, M80, M81, M87, M89, M91, M92, M93, M94
IG_Exp_To_Federal_Govt	S67, S74, S89
Direct_General_Expend	E01, E03, E04, E05, E12, E16, E18, E21, E22, E23, E24, E25, E26, E29, E31, E32, E36, E44, E45, E50, E52, E55, E56, E59,

	E60, E61, E62, E66, E74, E75, E77, E79, E80, E81, E85, E87, E89, E90, E91, E92, E93, E94, F01, F03, F04, F05, F12, F16, F18, F21, F22, F23, F24, F25, F26, F29, F31, F32, F36, F44, F45, F50, F52, F55, F56, F59, F60, F61, F62, F66, F77, F79, F80, F81, F85, F87, F89, F90, F91, F92, F93, F94, G01, G03, G04, G05, G12, G16, G18, G21, G22, G23, G24, G25, G26, G29, G31, G32, G36, G44, G45, G50, G52, G55, G56, G59, G60, G61, G62, G66, G77, G79, G80, G81, G85, G87, G89, G90, G91, G92, G93, G94, X11, X12, Y05, Y06, Y14, Y53, J19, J67, J68, J85, I89, I91, I92, I93, I94
General_Current_Expend	General_Expenditure, - General_Capital_Outlay
General_Current_Oper	E01, E03, E04, E05, E12, E16, E18, E21, E22, E23, E24, E25, E26, E29, E31, E32, E36, E44, E45, E50, E52, E55, E56, E59, E60, E61, E62, E66, E74, E75, E77, E79, E80, E81, E85, E87, E89, E90, E91, E92, E93, E94
General_Capital_Outlay	F01, F03, F04, F05, F12, F16, F18, F21, F22, F23, F24, F25, F26, F29, F31, F32, F36, F44, F45, F50, F52, F55, F56, F59, F60, F61, F62, F66, F77, F79, F80, F81, F85, F87, F89, G01, G03, G04, G05, G12, G16, G18, G21, G22, G23, G24, G25, G26, G29, G31, G32, G36, G44, G45, G50, G52, G55, G56, G59, G60, G61, G62, G66, G77, G79, G80, G81, G85, G87, G89
General_Construction	F01, F03, F04, F05, F12, F16, F18, F21, F22, F23, F24, F25, F26, F29, F31, F32, F36, F44, F45, F50, F52, F55, F56, F59, F60, F61, F62, F66, F77, F79, F80, F81, F85, F87, F89, F90, F91, F92, F93, F94
General_Capital_Outlay_Other	General_Capital_Outlay, -General_Construction
General_Assist___Sub	Tot_Assist___Subsidies
General_Debt_Interest	I89
Air_Trans_Total_Expend	E01 , F01 , G01 , L01 , M01
Air_Trans_Direct_Expend	E01 , F01 , G01
Air_Trans_Cap_Outlay	F01 , G01
Air_Trans_Current_Exp	Air_Trans_Direct_Expend, -Air_Trans_Cap_Outlay
Air_Trans_Construction	F01
Air_Trans_IG_To_State	L01
Air_Trans_IG_Local_Govts	M01
Misc_Com_Activ_Tot_Exp	E03 , F03 , G03
Misc_Com_Activ_Cap_Out	F03 , G03
Misc_Com_Activ_Current_Exp	Misc_Com_Activ_Tot_Exp, -Misc_Com_Activ_Cap_Out
Misc_Com_Activ_Constr	F03
Correct_Total_Exp	E04 , F04 , G04 , E05 , F05 , G05 , L04 , L05 , M04 , M05
Correct_Direct_Exp	E04 , E05 , F04 , G04 , F05 , G05
Correct_Cap_Outlay	F04 , G04 , F05 , G05
Correct_Current_Exp	Correct_Direct_Exp, -Correct_Cap_Outlay
Correct_Construct	F04 , F05
Correct_IG_To_St	L04 , L05
Correct_IG_Loc_Govts	M04 , M05
Total_Educ_Total_Exp	E12, F12, G12, E16, F16, G16, E18, F18, G18, J19, E21, F21, G21, L12, M12, Q12, L18, M18, L21, M21
Total_Educ_Direct_Exp	E12, F12, G12, E16, F16, G16, E18, F18, G18, J19, E21, F21, G21

Total_Educ_Assist___Sub	J19
Total_Educ_Cap_Outlay	F12, F16, F18, F21, G12, G16, G18, G21
Total_Educ_Current_Exp	Total_Educ_Direct_Exp, -Total_Educ_Cap_Outlay
Total_Educ_Construct	F12 , F16 , F18 , F21
Elem_Educ_Total_Exp	E12, F12, G12, L12, M12, Q12
Elem_Educ_Direct_Exp	E12, F12, G12
Elem_Educ_Cap_Outlay	F12, G12
Elem_Educ_Current_Exp	Elem_Educ_Direct_Exp, -Elem_Educ_Cap_Outlay
Elem_Educ_Construction	F12
Elem_Educ_IG_To_State	L12
Elem_Educ_IG_Local_Govts	M12
Elem_Educ_IG_Sch_to_Sch	Q12
Higher_Ed_Total_Exp	E16, E18, F16, F18, G16, G18, L18, M18
Higher_Ed_Direct_Exp	E16, E18, F16, F18, G16, G18
Higher_Ed_Cap_Outlay	F16, F18, G16, G18
Higher_Ed_Current_Exp	Higher_Ed_Direct_Exp, -Higher_Ed_Cap_Outlay
Higher_Ed_Construct	F16, F18
Higher_Ed_IG_To_St	L18
Higher_Ed_IG_Loc_Govts	M18
Educ_NEC_Total_Expend	E21, F21, G21, L21, M21
Educ_NEC_Direct_Expend	E21, F21, G21
Educ_NEC_Assistance	.
Educ_NEC_Cap_Outlay	F21, G21
Educ_NEC_Current_Exp	Educ_NEC_Direct_Expend, -Educ_NEC_Cap_Outlay
Educ_NEC_Construction	F21
Educ_NEC_IG_To_State	L21
Educ_NEC_IG_Local_Govts	M21
Emp_Sec_Adm_Direct_Exp	E22 , F22 , G22
Emp_Sec_Adm_Cap_Outlay	F22 , G22
Emp_Sec_Adm_Current_Exp	Emp_Sec_Adm_Direct_Exp, -Emp_Sec_Adm_Cap_Outlay
Emp_Sec_Adm_Construct	F22
Fin_Admin_Total_Exp	E23, F23, G23, L23, M23
Fin_Admin_Direct_Exp	E23, F23, G23
Fin_Admin_Cap_Outlay	F23, G23
Fin_Admin_Current_Exp	Fin_Admin_Direct_Exp, -Fin_Admin_Cap_Outlay
Fin_Admin_Construction	F23
Fin_Admin_IG_To_State	L23
Fin_Admin_IG_Local_Govts	M23
Fire_Prot_Total_Expend	E24, F24, G24, L24, M24
Fire_Prot_Direct_Exp	E24, F24, G24
Fire_Prot_Cap_Outlay	F24, G24
Fire_Prot_Current_Exp	Fire_Prot_Direct_Exp, -Fire_Prot_Cap_Outlay
Fire_Prot_Construction	F24
Fire_Prot_IG_To_State	L24
Fire_Prot_IG_Local_Govts	M24
Judicial_Total_Expend	E25, F25, G25, L25, M25
Judicial_Direct_Expend	E25, F25, G25
Judicial_Cap_Outlay	F25, G25
Judicial_Current_Exp	Judicial_Direct_Expend, -Judicial_Cap_Outlay
Judicial_Construction	F25
Judicial_IG_To_State	L25

Judicial_IG_Local_Govts	M25
Cen_Staff_Total_Expend	E29, F29, G29, L29, M29
Cen_Staff_Direct_Exp	E29, F29, G29
Cen_Staff_Cap_Outlay	F29, G29
Cen_Staff_Current_Exp	Cen_Staff_Direct_Exp, -Cen_Staff_Cap_Outlay
Cen_Staff_Construction	F29
Cen_Staff_IG_To_State	L29
Cen_Staff_IG_Local_Govts	M29
Gen_Pub_Bldg_Total_Exp	E31, F31, G31
Gen_Pub_Bldg_Cap_Out	F31, G31
Gen_Pub_Bldg_Current_Exp	Gen_Pub_Bldg_Total_Exp, -Gen_Pub_Bldg_Cap_Out
Gen_Pub_Bldg_Construct	F31
Health_Total_Expend	E32, F32, G32, L32, M32
Health_Direct_Expend	E32, F32, G32
Health_Capital_Outlay	F32, G32
Health_Current_Exp	Health_Direct_Expend, -Health_Capital_Outlay
Health_Construction	F32
Health_IG_To_State	L32
Health_IG_Local_Govts	M32
Total_Hospital_Total_Exp	E36, F36, G36, L36, M36
Total_Hospital_Dir_Exp	E36, F36, G36
Total_Hospital_Cap_Out	F36, G36
Total_Hospital_Current_Exp	Total_Hospital_Dir_Exp, -Total_Hospital_Cap_Out
Total_Hospital_Construct	F36
Total_Hospital_IG_To_State	L36
Total_Hospital_IG_Loc_Govts	M36
Own_Hospital_Total_Exp	E37, F37, G37
Own_Hospital_Cap_Out	F37, G37
Own_Hospital_Current_Exp	Own_Hospital_Total_Exp, -Own_Hospital_Cap_Out
Own_Hospital_Construct	F37
Hosp_Other_Total_Exp	E39, F39, G39, L39, M39
Hosp_Other_Direct_Exp	E39, F39, G39
Hosp_Other_Cap_Outlay	F39, G39
Hosp_Other_Current_Exp	Hosp_Other_Direct_Exp, -Hosp_Other_Cap_Outlay
Hosp_Other_Construct	F39
Hosp_Other_IG_To_State	L39
Hosp_Other_IG_Loc_Govts	M39
Total_Highways_Tot_Exp	E44, F44, G44, E45, F45, G45, L44, M44
Total_Highways_Dir_Exp	E44, F44, G44, E45, F45, G45
Total_Highways_Cap_Out	F44, G44, F45, G45
Total_Highways_Current_Exp	Total_Highways_Dir_Exp, -Total_Highways_Cap_Out
Total_Highways_Construct	F44, F45
Regular_Hwy_Total_Exp	E44, F44, G44, L44, M44
Regular_Hwy_Direct_Exp	E44, F44, G44
Regular_Hwy_Cap_Outlay	F44, G44
Regular_Hwy_Current_Exp	Regular_Hwy_Direct_Exp, -Regular_Hwy_Cap_Outlay
Regular_Hwy_Construct	F44
Regular_Hwy_IG_To_Sta	L44
Regular_Hwy_IG_Loc_Govts	M44
Toll_Hwy_Total_Expend	E45, F45, G45
Toll_Hwy_Cap_Outlay	F45, G45

Toll_Hwy_Current_Exp	Toll_Hwy_Total_Expend, -Toll_Hwy_Cap_Outlay
Toll_Hwy_Construction	F45
Transit_Sub_Total_Exp	.
Transit_Sub_Direct_Sub	.
Transit_Sub_IG_To_Sta	.
Transit_Sub_IG_Loc_Govts	.
Transit_Sub_To_Own_Sys	.
Hous___Com_Total_Exp	E50, F50, G50, L50, M50
Hous___Com_Direct_Exp	E50, F50, G50
Hous___Com_Cap_Outlay	F50, G50
Hous___Com_Current_Exp	Hous___Com_Direct_Exp, -Hous___Com_Cap_Outlay
Hous___Com_Construct	F50
Hous___Com_IG_To_State	L50
Hous___Com_IG_Loc_Govts	M50
Libraries_Total_Expend	E52, F52, G52, L52, M52
Libraries_Direct_Exp	E52, F52, G52
Libraries_Cap_Outlay	F52, G52
Libraries_Current_Exp	Libraries_Direct_Exp, -Libraries_Cap_Outlay
Libraries_Construction	F52
Libraries_IG_To_State	L52
Libraries_IG_Local_Govts	M52
Natural_Res_Total_Exp	E55, F55, G55, M55, E56, F56, G56, M56, E59, F59, G59, L59, M59
Natural_Res_Direct_Exp	E55, F55, G55, E56, F56, G56, E59, F59, G59
Natural_Res_Cap_Outlay	F55, G55, F56, G56, F59, G59
Natural_Res_Current_Exp	Natural_Res_Direct_Exp, -Natural_Res_Cap_Outlay
Natural_Res_Construct	F55, F56, F59
Natural_Res_IG_To_Sta	L59
Natural_Res_IG_Loc_Govts	M55, M56 , M59
Parking_Total_Expend	E60, F60, G60, L60, M60
Parking_Direct_Expend	E60, F60, G60
Parking_Capital_Outlay	F60, G60
Parking_Current_Exp	Parking_Direct_Expend, -Parking_Capital_Outlay
Parking_Construction	F60
Parking_IG_To_State	L60
Parking_IG_Local_Govts	M60
Parks___Rec_Total_Exp	E61, F61, G61, L61, M61
Parks___Rec_Direct_Exp	E61, F61, G61
Parks___Rec_Cap_Outlay	F61, G61
Parks___Rec_Current_Exp	Parks___Rec_Direct_Exp, -Parks___Rec_Cap_Outlay
Parks___Rec_Construct	F61
Parks___Rec_IG_To_Sta	L61
Parks___Rec_IG_Loc_Govts	M61
Police_Prot_Total_Exp	E62, F62, G62, L62, M62
Police_Prot_Direct_Exp	E62, F62, G62
Police_Prot_Cap_Outlay	F62, G62
Police_Prot_Current_Exp	Police_Prot_Direct_Exp, -Police_Prot_Cap_Outlay
Police_Prot_Construct	F62
Police_Prot_IG_To_Sta	L62
Police_Prot_IG_Loc_Govts	M62
Prot_Insp_Total_Exp	E66, F66, G66, L66, M66

Prot_Insp_Direct_Exp	E66, F66, G66
Prot_Insp_Cap_Outlay	F66, G66
Prot_Insp_Current_Exp	Prot_Insp_Direct_Exp, -Prot_Insp_Cap_Outlay
Prot_Insp_Construction	F66
Prot_Insp_IG_To_State	L66
Prot_Insp_IG_Local_Govts	M66
Public_Welf_Total_Exp	J67, L67, M67, J68 , M68, E74, E75, S74, E77, F77, G77, E79, F79, G79, L79, M79
Public_Welf_Direct_Exp	J67, J68 , E74, E75, E77, F77, G77, E79, F79, G79
Public_Welf_Cash_Asst	J67, J68, M67, M68
Public_Welf_Cap_Outlay	F77, G77, F79, G79
Public_Welf_Current_Exp	Public_Welf_Direct_Exp, -Public_Welf_Cap_Outlay, -Public_Welf_Cash_Asst
Public_Welf_Construct	F77, F79
Welf_Categ_Total_Exp	J67, L67, M67
Welf_Categ_Cash_Assist	J67
Welf_Categ_IG_To_State	L67
Welf_Categ_IG_Loc_Govts	M67
Welf_Cash_Total_Exp	J68 , M68
Welf_Cash_Cash_Assist	J68
Welf_Cash_IG_Local_Govts	M68
Welf_Vend_Pmts_Medical	E74
Welf_Vend_Pmts_NEC	E75
Welf_State_Share_Part_D	S74
Welf_Ins_Total_Exp	E77, F77, G77
Welf_Ins_Cap_Outlay	F77, G77
Welf_Ins_Current_Exp	
Welf_Ins_Construction	F77
Welf_NEC_Total_Expend	E79, F79, G79, L79, M79
Welf_NEC_Direct_Expend	E79, F79, G79
Welf_NEC_Cap_Outlay	F79, G79
Welf_NEC_Current_Exp	Welf_NEC_Direct_Expend, -Welf_NEC_Cap_Outlay
Welf_NEC_Construction	F79
Welf_NEC_IG_To_State	L79
Welf_NEC_IG_Local_Govts	M79
Sewerage_Total_Expend	E80, F80, G80, L80, M80
Sewerage_Direct_Expend	E80, F80, G80
Sewerage_Cap_Outlay	F80, G80
Sewerage_Current_Exp	Sewerage_Direct_Expend, -Sewerage_Cap_Outlay
Sewerage_Construction	F80
Sewerage_IG_To_State	L80
Sewerage_IG_Local_Govts	M80
SW_Mgmt_Total_Expend	E81, F81, G81, L81, M81
SW_Mgmt_Direct_Expend	E81, F81, G81
SW_Mgmt_Capital_Outlay	F81, G81
SW_Mgmt_Current_Exp	SW_Mgmt_Direct_Expend, -SW_Mgmt_Capital_Outlay
SW_Mgmt_Construction	F81
SW_Mgmt_IG_To_State	L81
SW_Mgmt_IG_Local_Govts	M81
Water_Trans_Total_Exp	E87, F87, G87, L87, M87
Water_Trans_Direct_Exp	E87, F87, G87

Water_Trans_Cap_Outlay	F87, G87
Water_Trans_Current_Exp	Water_Trans_Direct_Exp, -Water_Trans_Cap_Outlay
Water_Trans_Construct	F87
Water_Trans_IG_To_Sta	L87
Water_Trans_IG_Loc_Govts	M87
Interest_on_Gen_Debt	I89
General_NEC_Total_Exp	E89, F89, G89, L89, M89, S89, J89
General_NEC_Direct_Exp	E89, F89, G89, J89
VetBonus	J89
General_NEC_Cap_Outlay	F89, G89
General_NEC_Current_Exp	General_NEC_Direct_Exp, -General_NEC_Cap_Outlay, -VetBonus
General_NEC_Construct	F89
General_NEC_IG_To_St	L89
General_NEC_IG_Loc_Govts	M89
General_NEC_IG_To_Fed	S89
Liquor_Stores_Tot_Exp	E90, F90, G90
Liquor_Stores_Cap_Out	F90, G90
Liquor_Stores_Current_Exp	Liquor_Stores_Tot_Exp, -Liquor_Stores_Cap_Out
Liquor_Stores_Constr	F90
Total_Util_Total_Exp	E91, I91, F91, G91, L91, M91, E92, I92, F92, G92, L92, M92, E93, I93, F93, G93, L93, M93, E94, I94, F94, G94, L94, M94
Total_Util_Inter_Exp	I91, I92, I93, I94
Total_Util_Cap_Outlay	F91, F92, F93, F94, G91, G92, G93, G94
Total_Util_Current_Exp	Total_Util_Total_Exp, -Total_Util_Inter_Exp, -Total_Util_Cap_Outlay
Total_Util_Construct	F91, F92, F93, F94
Water_Util_Total_Exp	E91, I91, F91, G91, L91, M91
Water_Util_Inter_Exp	I91
Water_Util_Cap_Outlay	F91, G91
Water_Util_Current_Exp	Water_Util_Total_Exp, -Water_Util_Inter_Exp, -Water_Util_Cap_Outlay
Water_Util_Construct	F91
Elec_Util_Total_Exp	E92, I92, F92, G92, L92, M92
Elec_Util_Inter_Exp	I92
Elec_Util_Cap_Outlay	F92, G92
Elec_Util_Current_Exp	Elec_Util_Total_Exp, -Elec_Util_Inter_Exp, -Elec_Util_Cap_Outlay
Elec_Util_Construct	F92
Gas_Util_Total_Exp	E93, I93, F93, G93, L93, M93
Gas_Util_Inter_Exp	I93
Gas_Util_Cap_Outlay	F93, G93
Gas_Util_Current_Exp	Gas_Util_Total_Exp, -Gas_Util_Inter_Exp, -Gas_Util_Cap_Outlay
Gas_Util_Construct	F93
Trans_Util_Total_Exp	E94, I94, F94, G94, L94, M94
Trans_Util_Inter_Exp	I94
Trans_Util_Cap_Outlay	F94, G94
Trans_Util_Current_Exp	Trans_Util_Total_Exp, -Trans_Util_Inter_Exp, -Trans_Util_Cap_Outlay
Trans_Util_Construct	F94

Emp_Ret_Total_Expend	X11, X12
Emp_Ret_Benefit_Paymts	X11
Emp_Ret_Withdrawals	X12
Emp_Ret_Other_Paymts	.
Unemp_Comp_Total_Exp	Y05, Y06
Unemp_Comp_Ben_Paymts	Y05
Unemp_Ext___Spec_Pmts	Y06
Total_Debt_Outstanding	_44T, _49U, _64V
Total_Long_Term_Debt_Out	_44T, _49U
ST_Debt_End_of_Year	_64V
Total_Beg_LTD_Out	_19T, _19U
Beg_LTD_Out_Private_Purp	_19T
Beg_LTD_Out_All_Other	_19U
Beg_LTD_Out_Utility	. *All detailed debt codes were discontinued in 2005
Beg_LTD_Out_Water_Util	.
Beg_LTD_Out_Elec_Util	.
Beg_LTD_Out_Gas_Util	.
Beg_LTD_Out_Trans_Util	.
Beg_LTD_Out_General	.
Beg_LTD_Out_Education	.
Beg_LTD_Out_Priv_Purp	.
Beg_LTD_Out_Other_NEC	.
Total_LTD_Issued	_24T, _29U
LTD_Iss_Private_Purp	_24T
LTD_Iss_All_Other	_29U
LTD_Iss_Utility	.
LTD_Iss_Util_Water	.
LTD_Iss_Util_Electric	.
LTD_Iss_Util_Gas_Supply	.
LTD_Iss_Util_Transit	.
LTD_Iss_General	.
LTD_Iss_Gen_Elem_Educ	.
LTD_Iss_Gen_Other_Educ	.
LTD_Iss_Gen_Other_NEC	.
Total_LTD_Iss_FFC	.
LTD_Iss_FFC_Utility	.
LTD_Iss_FFC_Water_Util	.
LTD_Iss_FFC_Elec_Util	.
LTD_Iss_FFC_Gas_Util	.
LTD_Iss_FFC_Trans_Util	.
LTD_Iss_FFC_General	.
LTD_Iss_FFC_Elem_Educ	.
LTD_Iss_FFC_Other_Educ	.
LTD_Iss_FFC_Other_NEC	.
Total_LTD_Iss_NG	.
LTD_Iss_NG_Utility	.
LTD_Iss_NG_Water_Util	.
LTD_Iss_NG_Elec_Util	.
LTD_Iss_NG_Gas_Util	.
LTD_Iss_NG_Trans_Util	.
LTD_Iss_NG_General	.

LTD_Iss_NG_Elem_Educ	.
LTD_Iss_NG_Other_Educ	.
LTD_Iss_NG_Private_Purp	.
LTD_Iss_NG_Other_NEC	.
Total_LTD_Iss_Unsp	.
LTD_Iss_Unsp_Utility	.
LTD_Iss_Unsp_Water_Util	.
LTD_Iss_Unsp_Elec_Util	.
LTD_Iss_Unsp_Gas_Util	.
LTD_Iss_Unsp_Trans_Util	.
LTD_Iss_Unsp_General	.
LTD_Iss_Unsp_Elem_Educ	.
LTD_Iss_Unsp_Other_Educ	.
LTD_Iss_Unsp_Other_NEC	.
Total_LTD_Retired	_34T, _39U
LTD_Ret_Private_Purp	_34T
LTD_Ret_All_Other	_39U
LTD_Ret_Utility	.
LTD_Ret_Util_Water	.
LTD_Ret_Util_Electric	.
LTD_Ret_Util_Gas_Supply	.
LTD_Ret_Util_Transit	.
LTD_Ret_General	.
LTD_Ret_Gen_Elem_Educ	.
LTD_Ret_Gen_Other_Educ	.
LTD_Ret_Gen_Other_NEC	.
Total_LTD_Ret_FFC	.
LTD_Ret_FFC_Utility	.
LTD_Ret_FFC_Water_Util	.
LTD_Ret_FFC_Elec_Util	.
LTD_Ret_FFC_Gas_Util	.
LTD_Ret_FFC_Trans_Util	.
LTD_Ret_FFC_General	.
LTD_Ret_FFC_Elem_Educ	.
LTD_Ret_FFC_Other_Educ	.
LTD_Ret_FFC_Other_NEC	.
Total_LTD_Ret_NG	.
LTD_Ret_NG_Utility	.
LTD_Ret_NG_Water_Util	.
LTD_Ret_NG_Elec_Util	.
LTD_Ret_NG_Gas_Util	.
LTD_Ret_NG_Trans_Util	.
LTD_Ret_NG_General	.
LTD_Ret_NG_Elem_Educ	.
LTD_Ret_NG_Other_Educ	.
LTD_Ret_NG_Private_Purp	.
LTD_Ret_NG_Other_NEC	.
Total_LTD_Ret_Unsp	.
LTD_Ret_Unsp_Utility	.
LTD_Ret_Unsp_Water_Util	.
LTD_Ret_Unsp_Elec_Utili	.

LTD_Ret_Unsp_Gas_Util	.
LTD_Ret_Unsp_Trans_Util	.
LTD_Ret_Unsp_General	.
LTD_Ret_Unsp_Elem_Educ	.
LTD_Ret_Unsp_Other_Educ	.
LTD_Ret_Unsp_Other_NEC	.
Total_LTD_Out	_44T, _49U
LTD_Out_Private_Purp	_44T
LTD_Out_All_Other	_49U
Total_LTD_Out_Utility	.
LTD_Out_Util_Water	.
LTD_Out_Util_Electric	.
LTD_Out_Util_Gas_Supply	.
LTD_Out_Util_Transit	.
LTD_Out_General	.
LTD_Out_Gen_Elem_Educ	.
LTD_Out_Gen_Other_Educ	.
LTD_Out_Gen_Other_NEC	.
Total_LTD_Out_FFC	.
LTD_Out_FFC_Utility	.
LTD_Out_FFC_Water_Util	.
LTD_Out_FFC_Elec_Util	.
LTD_Out_FFC_Gas_Util	.
LTD_Out_FFC_Trans_Util	.
LTD_Out_FFC_General	.
LTD_Out_FFC_Elem_Educ	.
LTD_Out_FFC_Other_Educ	.
LTD_Out_FFC_Other_NEC	.
Tot_LTD_Out_NG	.
LTD_Out_NG_Utility	.
LTD_Out_NG_Water_Util	.
LTD_Out_NG_Elec_Util	.
LTD_Out_NG_Gas_Util	.
LTD_Out_NG_Trans_Util	.
LTD_Out_NG_General	.
LTD_Out_NG_Elem_Educ	.
LTD_Out_NG_Other_Educ	.
LTD_Out_NG_Private_Purp	.
LTD_Out_NG_Other_NEC	.
Total_Cash___Securities	W01, W31, W61, X21, X30, Z77, Z78, X42, X44, X47, Y07, Y08, Y21, Y61
Insur_Trust_Cash___Sec	X21, X30, Z77, Z78, X42, X44, X47, Y07, Y08, Y21, Y61
Emp_Retire_Cash___Sec	X21, X30, X35, Z77, Z78, X42, X47, X44
Emp_Retire_Cash___Dep	X21
Emp_Retire_Total_Sec	X30, X35, Z77, Z78, X42, X47, X44
Emp_Retire_Sec_Tot_Fed	X30
Emp_Retire_Sec_S_L_Secur	X35
Emp_Retire_Sec_Tot_Nong	Z77, Z78, X42, X47, X44
Emp_Retire_Sec_Corp_Bds	Z77
Emp_Retire_Sec_Corp_Stk	Z78
Emp_Retire_Sec_Mortgages	X42

Emp_Retire_Sec_Misc_Inv	X47
Emp_Retire_Sec_Oth_Nong	X44
Unemp_Comp_Cash___Sec	Y07, Y08
Unemp_Comp_Bal_In_US_Trs	Y07
Unemp_Comp_Other_Balance	Y08
Nonin_Trust_Cash___Sec	W01, W31, W61
Sinking_Fd_Cash___Sec	W01
Bond_Fd_Cash___Sec	W31
Oth_Nonin_Fd_Cash___Sec	W61

Appendix C: Sas Code for Data Consolidation

The following is the complete SAS code we used to consolidate all of the files we received from the census. Please refer to appendix A for step by step instructions for using this SAS code.

```
* This sas macro merges the historical data files that the census provides;
%macro census;
* Defines the text that is repeated in each census file. Base text should be
changed to your correct file path (it is declared twice in the code, so
change both);
%LET BaseText = E:/CensusData/IndFin/IndFin0;
%LET EndTextA = a.Txt;
%LET EndTextB = b.Txt;
%LET EndTextC = c.Txt;
%do i = 7 %to 0 %by -1;
      * Import the data from each of the three physical files in the current
year;
      %Let FileName = &BaseText&i&EndTextA; *Creates concatenated file names
following the census naming conventions;
      proc import datafile="&FileName" out=DataA dbms=dlm replace;
            delimiter=",";
            getnames=yes;
            GUESSINGROWS=700; *This takes longer to run than is optimal, but
will help to ensure that the data types created by proc import are correct.;
      run;
      %Let FileName = &BaseText&i&EndTextB;
      proc import datafile="&FileName" out=DataB dbms=dlm replace;
         delimiter=",";
         getnames=yes;
      run;
      %Let FileName = &BaseText&i&EndTextC;
      proc import datafile="&FileName" out=DataC dbms=dlm replace;
         delimiter=",";
         getnames=yes;
      run;
      * Merge the three data items into one;
      Data LatestData;
            Merge DataA DataB DataC;
            by ID;
      run;
      * The if then else structure allows the larger data set to be created
for the first set of files;
      %if &i = 7 %then
            %do;
            * Add trailing zeros to the ID number to match the format in more
recent data;
            Data LargeData;
            set LatestData;
            run;
            %end;
      %else
            %do;
            * Append the most recently merged data to the larger data file;
            PROC APPEND BASE= LargeData DATA= LatestData force;
            RUN;
      %end;
```

```
%end;
%LET BaseText = E:/CensusData/IndFin/IndFin; * Make sure to change this
destination to match your file system;
%do i = 70 %to 99;
* Import the data from each of the three physical files in the current year;
      %Let FileName = &BaseText&i&EndTextA; *Creates concatenated file names
following the census naming conventions;
      proc import datafile="&FileName" out=DataA dbms=dlm replace;
            delimiter=",";
            getnames=yes;
            GUESSINGROWS=700;
      run;
      %Let FileName = &BaseText&i&EndTextB;
      proc import datafile="&FileName" out=DataB dbms=dlm replace;
         delimiter=",";
         getnames=yes;
      run;
      %Let FileName = &BaseText&i&EndTextC;
      proc import datafile="&FileName" out=DataC dbms=dlm replace;
         delimiter=",";
         getnames=yes;
      run;
      * Merge the three data items into one;
      Data LatestData;
            Merge DataA DataB DataC;
            by ID;
      run;
      * Append the most recently merged data to the larger data file;
      PROC APPEND BASE= LargeData DATA= LatestData force;
      RUN;
%end;
%do i = 67 %to 67;
* Import the data from each of the three physical files in the current year;
      %Let FileName = &BaseText&i&EndTextA; *Creates concatenated file names
following the census naming conventions;
      proc import datafile="&FileName" out=DataA dbms=dlm replace;
            delimiter=",";
            getnames=yes;
            GUESSINGROWS=700;
      run;
      %Let FileName = &BaseText&i&EndTextB;
      proc import datafile="&FileName" out=DataB dbms=dlm replace;
         delimiter=",";
         getnames=yes;
      run;
      %Let FileName = &BaseText&i&EndTextC;
      proc import datafile="&FileName" out=DataC dbms=dlm replace;
         delimiter=",";
         getnames=yes;
      run;
      * Merge the three data items into one;
      Data LatestData;
            Merge DataA DataB DataC;
            by ID;
      run;
      * Append the most recently merged data to the larger data file;
      PROC APPEND BASE= LargeData DATA= LatestData force;
```

```
      RUN;
%end;

%MEND Census;

* This line runs the macro shown above;
%Census;

*Create variables not existing in earlier data that can be calculated from
it;
Data LargeData;
      Set LargeData;
      *Manipulate certain identification variables that can be improved in
the overall database;
      FunctionCode = .;
      Enrollment = .;
      if Type_Code = '4' then
            do;
            FunctionCode = Population;
            Population = .;
            Enrollment = .;
            end;
      if Type_Code = '5' then
            do;
            FunctionCode = .;
            Enrollment = Population;
            Population = .;
            end;
      run;

* We now need to format the newer census data to roughly match the format of
the earlier data.  This process is;
* accomplished without a macro because there are only a few years of newer
data, and the file names thend to be non-standard;
* Make sure that you change the "Inflie" line on each of the data steps to
the path for the files on your system.

* Read the financial data file in fixed width format, dollar signs indicate
values which are stored as characters;
DATA FinancialData;
      INFILE 'E:/CensusData/2011/2011FinEstDAT_ALL5modp_pu.txt'
      MISSOVER;
      INPUT id_state $ 1-2 id_type $ 3 id_county $ 4-6 id_unit $ 7-9 id_add $
10-14 itemcode $ 15-17 data 18-29 year 30-33 code $ 34;
      id = cats(of id_state id_type id_county id_unit);
RUN;

* Transpose the financial information file so that every government ID number
corresponds to one row and every data item has its own column;
PROC TRANSPOSE data=work.FinancialData
                        out=work.TransposedData
                        name=Year;
                  var data;
                  by id;
                  id itemcode;
            run;
```

```
* Change the "year" variable in the finacnial data matrix to be the year we
are currently working with;
Data TransposedDates;
      Set work.TransposedData;
      Year = 2011;
run;

* Sort each of the matricies by ID code in preparation for merging them.;
proc sort data=TransposedDates;
      by id;
run;

* Read the government information file as a fixed width text file.  Dollar
signs indicate fields that are coded as text.;
DATA Identification;
      INFILE 'E:/CensusData/2011/fin_gid_2011.txt'
      MISSOVER;
      INPUT id_state $ 1-2 id_type $ 3 id_county $ 4-6 id_unit $ 7-9 id_add $
10-14 Name $ 15-78 CountyName $ 79-113 FIPSstate $ 114-115 FIPScounty $ 116-
118 FIPSplace $ 119-123 Population 124-132 PopYear $ 133-134 Enrollment 135-
141 EnrollYear $ 142-143 FunctionCode 144-145 SchoolLevel $ 146-147 FYEnd $
148-151 SurveyYear $ 152-153;
      id = cats(of id_state id_type id_county id_unit);
RUN;

* Merge the government identification file with the transposed, dated,
finacial information file;
Data LatestData;
      Merge Identification TransposedDates;
      By id;
  run;

*Concatenate the Latest data to the larger data file preserving every data
element for later reorganization;
Data NewData;
      Set LatestData;
Run;

*Begin the same process for the 2010 data;
  *
  *
  *
  *;


* Read the financial data file in fixed width format, dollar signs indicate
values which are stored as characters;
DATA FinancialData;
      INFILE 'E:/CensusData/2010/2010FinEstDAT_ALL12modp_pu.txt'
      MISSOVER;
      INPUT id_state $ 1-2 id_type $ 3 id_county $ 4-6 id_unit $ 7-9 id_add $
10-14 itemcode $ 15-17 data 18-29 year 30-33 code $ 34;
      id = cats(of id_state id_type id_county id_unit);
RUN;

* Transpose the financial information file so that every government ID number
corresponds to one row and every data item has its own column;
```

```
PROC TRANSPOSE data=work.FinancialData
                        out=work.TranspsedData
                        name=Year;
                  var data;
                  by id;
                  id itemcode;
            run;

* Change the "year" variable in the finacnial data matrix to be the year we
are currently working with;
Data TransposedDates;
      Set work.TransposedData;
      Year = 2010;
run;

* Sort each of the matricies by ID code in preparation for merging them.;
proc sort data=TransposedDates;
      by id;
run;

* Read the government information file as a fixed width text file.  Dollar
signs indicate fields that are coded as text.;
DATA Identification;
      INFILE 'E:/CensusData/2010/fin_gid_2010.txt'
      MISSOVER;
      INPUT id_state $ 1-2 id_type $ 3 id_county $ 4-6 id_unit $ 7-9 id_add $
10-14 Name $ 15-78 CountyName $ 79-113 FIPSstate $ 114-115 FIPScounty $ 116-
118 FIPSplace $ 119-123 Population 124-132 PopYear $ 133-134 Enrollment 135-
141 EnrollYear $ 142-143 FunctionCode 144-145 SchoolLevel $ 146-147 FYEnd $
148-151 SurveyYear $ 152-153;
      id = cats(of id_state id_type id_county id_unit);
RUN;

* Merge the government identification file with the transposed, dated,
finacial information file;
Data LatestData;
      Merge Identification TransposedDates;
      By id;
  run;

*Concatenate the Latest data to the larger data file preserving every data
element for later reorganization;
Data NewData;
      Set NewData LatestData;
Run;

*Begin the same process for the 2009 data;
  *
  *
  *
  *;


* Read the financial data file in fixed width format, dollar signs indicate
values which are stored as characters;
DATA FinancialData;
      INFILE 'E:/CensusData/2009/2009FinEstDAT_ALL16modp_pu.txt'
```

```
      MISSOVER;
      INPUT id_state $ 1-2 id_type $ 3 id_county $ 4-6 id_unit $ 7-9 id_add $
10-14 itemcode $ 15-17 data 18-29 year 30-33 code $ 34;
      id = cats(of id_state id_type id_county id_unit);
RUN;

* Transpose the financial information file so that every government ID number
corresponds to one row and every data item has its own column;
PROC TRANSPOSE data=work.FinancialData
                        out=work.TransposedData
                        name=Year;
                  var data;
                  by id;
                  id itemcode;
            run;

* Change the "year" variable in the finacnial data matrix to be the year we
are currently working with;
Data TransposedDates;
      Set work.TransposedData;
      Year = 2009;
run;

* Sort each of the matricies by ID code in preparation for merging them.;
proc sort data=TransposedDates;
      by id;
run;

* Read the government information file as a fixed width text file.  Dollar
signs indicate fields that are coded as text.;
DATA Identification;
      INFILE 'E:/CensusData/2009/fin_gid_2009.txt'
      MISSOVER;
      INPUT id_state $ 1-2 id_type $ 3 id_county $ 4-6 id_unit $ 7-9 id_add $
10-14 Name $ 15-78 CountyName $ 79-113 FIPSstate $ 114-115 FIPScounty $ 116-
118 FIPSplace $ 119-123 Population 124-132 PopYear $ 133-134 Enrollment 135-
141 EnrollYear $ 142-143 FunctionCode 144-145 SchoolLevel $ 146-147 FYEnd $
148-151 SurveyYear $ 152-153;
      id = cats(of id_state id_type id_county id_unit);
RUN;

* Merge the government identification file with the transposed, dated,
finacial information file;
Data LatestData;
      Merge Identification TransposedDates;
      By id;
  run;

*Concatenate the Latest data to the larger data file preserving every data
element for later reorganization;
Data NewData;
      Set NewData LatestData;
Run;

*Begin the same process for the 2008 data;
  *
  *
```

```
  *
  *;


* Read the financial data file in fixed width format, dollar signs indicate
values which are stored as characters;
DATA FinancialData;
      INFILE 'E:/CensusData/2008/2008FinInddiv15_modp3.txt'
      MISSOVER;
      INPUT id_state $ 1-2 id_type $ 3 id_county $ 4-6 id_unit $ 7-9 id_add $
10-14 itemcode $ 15-17 data 18-29 year 30-33 code $ 34;
      id = cats(of id_state id_type id_county id_unit);
RUN;

* Transpose the financial information file so that every government ID number
corresponds to one row and every data item has its own column;
PROC TRANSPOSE data=work.FinancialData LET
                        out=work.TransposedData
                        name=Year;
                  var data;
                  by id;
                  id itemcode;
            run;

* Change the "year" variable in the finacnial data matrix to be the year we
are currently working with;
Data TransposedDates;
      Set work.TransposedData;
      Year = 2008;
run;

* Sort each of the matricies by ID code in preparation for merging them.;
proc sort data=TransposedDates;
      by id;
run;

* Read the government information file as a fixed width text file.  Dollar
signs indicate fields that are coded as text.;
DATA Identification;
      INFILE 'E:/CensusData/2008/fin_gid_2008.txt'
      MISSOVER;
      INPUT id_state $ 1-2 id_type $ 3 id_county $ 4-6 id_unit $ 7-9 id_add $
10-14 Name $ 15-78 CountyName $ 79-113 FIPSstate $ 114-115 FIPScounty $ 116-
118 FIPSplace $ 119-123 Population 124-132 PopYear $ 133-134 Enrollment 135-
141 EnrollYear $ 142-143 FunctionCode 144-145 SchoolLevel $ 146-147 FYEnd $
148-151 SurveyYear $ 152-153;
      id = cats(of id_state id_type id_county id_unit);
RUN;

* Merge the government identification file with the transposed, dated,
finacial information file;
Data LatestData;
      Merge Identification TransposedDates;
      By id;
  run;
```

```
*Concatenate the Latest data to the larger data file preserving every data
element for later reorganization;
Data NewData;
      Set NewData LatestData;
Run;

*
*
*
*
*Manually convert the newer data to the format of the older data and keep
only variables that exist in the final data set;

Data ConvertedNewData (keep= SurveyYr Year4 ID State_Code Type_Code County
Name FIPS_Code_State FYEndDate YearPop SchLevCode Population Total_Revenue
Total_Rev_Own_Sources General_Revenue Gen_Rev_Own_Sources Total_Taxes
Property_Tax Tot_Sales___Gr_Rec_Tax Total_Gen_Sales_Tax
Total_Select_Sales_Tax Alcoholic_Beverage_Tax Amusement_Tax
Insurance_Premium_Tax Motor_Fuels_Tax Pari_mutuels_Tax Public_Utility_Tax
Tobacco_Tax Other_Select_Sales_Tax Total_License_Taxes Alcoholic_Beverage_Lic
Amusement_License Corporation_License Hunting___Fishing_License
Motor_Vehicle_License Motor_Veh_Oper_License Public_Utility_License
Occup_and_Bus_Lic_NEC Other_License_Taxes Total_Income_Taxes
Individual_Income_Tax Corp_Net_Income_Tax Death_and_Gift_Tax
Docum_and_Stock_Tr_Tax Severance_Tax Taxes_NEC Total_IG_Revenue
Total_Fed_IG_Revenue Fed_IGR_Air_Transport Fed_IGR_Education
Fed_IGR_Emp_Sec_Adm Fed_IGR_Gen_Rev_Shar Fed_IGR_Gen_Support
Fed_IGR_Health___Hos Fed_IGR_Highways
Fed_IGR_Transit_Sub Fed_IGR_Hous_Com_Dev  Fed_IGR_Natural_Res
Fed_IGR_Public_Welf Fed_IGR_Sewerage Fed_IGR_Other Total_State_IG_Revenue
State_IGR_Education State_IGR_Tax_Relief State_IGR_Oth_Gen_Sup
State_IGR_Health___Hos State_IGR_Highways State_IGR_Transit_Sub
State_IGR_Hous_Com_Dev State_IGR_Public_Welf State_IGR_Sewerage
State_IGR_Other Tot_Local_IG_Rev Local_IGR_InterSchool_Aid
Local_IGR_Other_Education Local_IGR_Oth_Gen_Sup Local_IGR_Health___Hos
Local_IGR_Highways
Local_IGR_Transit_Sub Local_IGR_Hous_Com_Dev Local_IGR_Public_Welf
      Local_IGR_Sewerage Local_IGR_Other Tot_Chgs_and_Misc_Rev
Total_General_Charges Chg_Air_Transportation    Chg_Misc_Com_Activ
      Chg_Total_Education     Chg_Elem_Ed_Sch_Lunch    Chg_Elem_Ed_Tuition
      Chg_Elem_Ed_NEC   Chg_Total_High_Ed Chg_Hospitals
      Chg_Regular_Highways    Chg_Toll_Highways Chg_Housing___Comm_Dev
      Chg_Total_Nat_Res Chg_Parking Chg_Parks___Recreation  Chg_Sewerage
      Chg_Solid_Waste_Mgmt
Chg_Water_Transport     Chg_All_Other_NEC Misc_General_Revenue
      Special_Assessments     Prop_Sale_Hous_Com_Dev  Prop_Sale_Other
      Interest_Revenue  Fines_and_Forfeits      Rents_and_Royalties
      Net_Lottery_Revenue     Misc_General_Rev_NEC    Liquor_Stores_Revenue
      Total_Utility_Revenue     Water_Utility_Revenue   Electric_Utility_Rev
      Gas_Utility_Rev   Transit_Utility_Rev     Total_Insur_Trust_Rev
      Total_Insur_Trust_Ctrb  Tot_Ins_Trust_Inv_Rev   Total_Emp_Ret_Rev
Emp_Ret_Total_Ctrib     Emp_Ret_Loc_Emp_Ctrib   Emp_Ret_Loc_To_Loc_Sys
      Emp_Ret_From_Other_Gov  Emp_Ret_Sta_To_Sta_Ctr  Emp_Ret_Int_Rev
      Emp_Ret_Other_Earnings  Total_Unemp_Rev   Unemp_Payroll_Tax
      Unemp_Int_Revenue Unemp_Federal_Advances  Total_Expenditure
      Total_IG_Expenditure    Direct_Expenditure      Total_Current_Expend
      Total_Current_Oper      Total_Capital_Outlays   Total_Construction
```

```
      Tot_Assist___Subsidies  Total_Interest_on_Debt  Total_Insur_Trust_Ben
      Total_Salaries___Wages
General_Expenditure     IG_Exp_To_State_Govt    IG_Exp_To_Local_Govts
      IG_Exp_To_Federal_Govt  Direct_General_Expend   General_Current_Expend
      General_Current_Oper    General_Capital_Outlay  General_Construction
      General_Assist___Sub    General_Debt_Interest   Air_Trans_Total_Expend
      Air_Trans_Direct_Expend Air_Trans_Cap_Outlay    Air_Trans_Construction
      Air_Trans_IG_To_State   Air_Trans_IG_Local_Govts
      Misc_Com_Activ_Tot_Exp  Misc_Com_Activ_Cap_Out  Misc_Com_Activ_Constr
Correct_Total_Exp Correct_Direct_Exp      Correct_Cap_Outlay
      Correct_Construct Correct_IG_To_St  Correct_IG_Loc_Govts
      Total_Educ_Total_Exp    Total_Educ_Direct_Exp   Total_Educ_Assist___Sub
      Total_Educ_Cap_Outlay   Total_Educ_Construct    Elem_Educ_Total_Exp
      Elem_Educ_Direct_Exp    Elem_Educ_Cap_Outlay    Elem_Educ_Construction
      Elem_Educ_IG_To_State   Elem_Educ_IG_Local_Govts
      Elem_Educ_IG_Sch_to_Sch Higher_Ed_Total_Exp     Higher_Ed_Direct_Exp
      Higher_Ed_Cap_Outlay
Higher_Ed_Construct     Higher_Ed_IG_To_St      Higher_Ed_IG_Loc_Govts
      Educ_NEC_Total_Expend   Educ_NEC_Direct_Expend  Educ_NEC_Assistance
      Educ_NEC_Cap_Outlay     Educ_NEC_Construction   Educ_NEC_IG_To_State
      Educ_NEC_IG_Local_Govts Emp_Sec_Adm_Direct_Exp  Emp_Sec_Adm_Cap_Outlay
      Emp_Sec_Adm_Construct   Fin_Admin_Total_Exp     Fin_Admin_Direct_Exp
      Fin_Admin_Cap_Outlay    Fin_Admin_Construction  Fin_Admin_IG_To_State
      Fin_Admin_IG_Local_Govts      Fire_Prot_Total_Expend
      Fire_Prot_Direct_Exp
Fire_Prot_Cap_Outlay    Fire_Prot_Construction  Fire_Prot_IG_To_State
      Fire_Prot_IG_Local_Govts      Judicial_Total_Expend
      Judicial_Direct_Expend  Judicial_Cap_Outlay     Judicial_Construction
      Judicial_IG_To_State    Judicial_IG_Local_Govts Cen_Staff_Total_Expend
      Cen_Staff_Direct_Exp    Cen_Staff_Cap_Outlay    Cen_Staff_Construction
      Cen_Staff_IG_To_State   Cen_Staff_IG_Local_Govts
      Gen_Pub_Bldg_Total_Exp  Gen_Pub_Bldg_Cap_Out    Gen_Pub_Bldg_Construct
Health_Total_Expend     Health_Direct_Expend    Health_Capital_Outlay
      Health_Construction     Health_IG_To_State      Health_IG_Local_Govts
      Total_Hospital_Total_Exp      Total_Hospital_Dir_Exp
      Total_Hospital_Cap_Out  Total_Hospital_Construct
      Total_Hospital_IG_To_State    Total_Hospital_IG_Loc_Govts
      Own_Hospital_Total_Exp  Own_Hospital_Cap_Out    Own_Hospital_Construct
      Hosp_Other_Total_Exp    Hosp_Other_Direct_Exp   Hosp_Other_Cap_Outlay
      Hosp_Other_Construct    Hosp_Other_IG_To_State
Hosp_Other_IG_Loc_Govts Total_Highways_Tot_Exp  Total_Highways_Dir_Exp
      Total_Highways_Cap_Out  Total_Highways_Construct
      Regular_Hwy_Total_Exp   Regular_Hwy_Direct_Exp  Regular_Hwy_Cap_Outlay
      Regular_Hwy_Construct   Regular_Hwy_IG_To_Sta
      Regular_Hwy_IG_Loc_Govts      Toll_Hwy_Total_Expend
      Toll_Hwy_Cap_Outlay     Toll_Hwy_Construction   Transit_Sub_Total_Exp
      Transit_Sub_Direct_Sub  Transit_Sub_IG_To_Sta
      Transit_Sub_IG_Loc_Govts      Transit_Sub_To_Own_Sys
      Hous___Com_Total_Exp
Hous___Com_Direct_Exp   Hous___Com_Cap_Outlay   Hous___Com_Construct
      Hous___Com_IG_To_State  Hous___Com_IG_Loc_Govts Libraries_Total_Expend
      Libraries_Direct_Exp    Libraries_Cap_Outlay    Libraries_Construction
      Libraries_IG_To_State   Libraries_IG_Local_Govts
      Natural_Res_Total_Exp   Natural_Res_Direct_Exp  Natural_Res_Cap_Outlay
      Natural_Res_Construct   Natural_Res_IG_To_Sta
      Natural_Res_IG_Loc_Govts      Parking_Total_Expend
      Parking_Direct_Expend   Parking_Capital_Outlay
```

```
Parking_Construction    Parking_IG_To_State     Parking_IG_Local_Govts
      Parks___Rec_Total_Exp   Parks___Rec_Direct_Exp  Parks___Rec_Cap_Outlay
      Parks___Rec_Construct   Parks___Rec_IG_To_Sta
      Parks___Rec_IG_Loc_Govts      Police_Prot_Total_Exp
      Police_Prot_Direct_Exp  Police_Prot_Cap_Outlay  Police_Prot_Construct
      Police_Prot_IG_To_Sta   Police_Prot_IG_Loc_Govts
      Prot_Insp_Total_Exp     Prot_Insp_Direct_Exp    Prot_Insp_Cap_Outlay
      Prot_Insp_Construction  Prot_Insp_IG_To_State
Prot_Insp_IG_Local_Govts      Public_Welf_Total_Exp   Public_Welf_Direct_Exp
      Public_Welf_Cash_Asst   Public_Welf_Cap_Outlay  Public_Welf_Construct
      Welf_Categ_Total_Exp    Welf_Categ_Cash_Assist  Welf_Categ_IG_To_State
      Welf_Categ_IG_Loc_Govts Welf_Cash_Total_Exp     Welf_Cash_Cash_Assist
      Welf_Cash_IG_Local_Govts      Welf_Vend_Pmts_Medical
      Welf_Vend_Pmts_NEC      Welf_State_Share_Part_D Welf_Ins_Total_Exp
      Welf_Ins_Cap_Outlay     Welf_Ins_Construction   Welf_NEC_Total_Expend
Welf_NEC_Direct_Expend  Welf_NEC_Cap_Outlay     Welf_NEC_Construction
      Welf_NEC_IG_To_State    Welf_NEC_IG_Local_Govts Sewerage_Total_Expend
      Sewerage_Direct_Expend  Sewerage_Cap_Outlay     Sewerage_Construction
      Sewerage_IG_To_State    Sewerage_IG_Local_Govts SW_Mgmt_Total_Expend
      SW_Mgmt_Direct_Expend   SW_Mgmt_Capital_Outlay  SW_Mgmt_Construction
      SW_Mgmt_IG_To_State     SW_Mgmt_IG_Local_Govts  Water_Trans_Total_Exp
      Water_Trans_Direct_Exp  Water_Trans_Cap_Outlay  Water_Trans_Construct
Water_Trans_IG_To_Sta   Water_Trans_IG_Loc_Govts      Interest_on_Gen_Debt
      General_NEC_Total_Exp   General_NEC_Direct_Exp  VetBonus
      General_NEC_Cap_Outlay  General_NEC_Construct   General_NEC_IG_To_St
      General_NEC_IG_Loc_Govts      General_NEC_IG_To_Fed
      Liquor_Stores_Tot_Exp   Liquor_Stores_Cap_Out   Liquor_Stores_Constr
      Total_Util_Total_Exp    Total_Util_Inter_Exp    Total_Util_Cap_Outlay
      Total_Util_Construct    Water_Util_Total_Exp    Water_Util_Inter_Exp
Water_Util_Cap_Outlay   Water_Util_Construct    Elec_Util_Total_Exp
      Elec_Util_Inter_Exp     Elec_Util_Cap_Outlay    Elec_Util_Construct
      Gas_Util_Total_Exp      Gas_Util_Inter_Exp      Gas_Util_Cap_Outlay
      Gas_Util_Construct      Trans_Util_Total_Exp    Trans_Util_Inter_Exp
      Trans_Util_Cap_Outlay   Trans_Util_Construct    Emp_Ret_Total_Expend
      Emp_Ret_Benefit_Paymts  Emp_Ret_Withdrawals     Emp_Ret_Other_Paymts
      Unemp_Comp_Total_Exp    Unemp_Comp_Ben_Paymts   Unemp_Ext___Spec_Pmts
Total_Debt_Outstanding  Total_Long_Term_Debt_Out      ST_Debt_End_of_Year
      Total_Beg_LTD_Out Beg_LTD_Out_Private_Purp      Beg_LTD_Out_All_Other
      Beg_LTD_Out_Utility     Beg_LTD_Out_Water_Util  Beg_LTD_Out_Elec_Util
      Beg_LTD_Out_Gas_Util    Beg_LTD_Out_Trans_Util  Beg_LTD_Out_General
      Beg_LTD_Out_Education   Beg_LTD_Out_Priv_Purp   Beg_LTD_Out_Other_NEC
      Total_LTD_Issued  LTD_Iss_Private_Purp    LTD_Iss_All_Other
      LTD_Iss_Utility   LTD_Iss_Util_Water      LTD_Iss_Util_Electric
LTD_Iss_Util_Gas_Supply LTD_Iss_Util_Transit    LTD_Iss_General
      LTD_Iss_Gen_Elem_Educ   LTD_Iss_Gen_Other_Educ  LTD_Iss_Gen_Other_NEC
      Total_LTD_Iss_FFC LTD_Iss_FFC_Utility     LTD_Iss_FFC_Water_Util
      LTD_Iss_FFC_Elec_Util   LTD_Iss_FFC_Gas_Util    LTD_Iss_FFC_Trans_Util
      LTD_Iss_FFC_General     LTD_Iss_FFC_Elem_Educ   LTD_Iss_FFC_Other_Educ
      LTD_Iss_FFC_Other_NEC   Total_LTD_Iss_NG  LTD_Iss_NG_Utility
      LTD_Iss_NG_Water_Util   LTD_Iss_NG_Elec_Util    LTD_Iss_NG_Gas_Util
LTD_Iss_NG_Trans_Util   LTD_Iss_NG_General      LTD_Iss_NG_Elem_Educ
      LTD_Iss_NG_Other_Educ   LTD_Iss_NG_Private_Purp LTD_Iss_NG_Other_NEC
      Total_LTD_Iss_Unsp      LTD_Iss_Unsp_Utility    LTD_Iss_Unsp_Water_Util
      LTD_Iss_Unsp_Elec_Util  LTD_Iss_Unsp_Gas_Util   LTD_Iss_Unsp_Trans_Util
      LTD_Iss_Unsp_General    LTD_Iss_Unsp_Elem_Educ  LTD_Iss_Unsp_Other_Educ
      LTD_Iss_Unsp_Other_NEC  Total_LTD_Retired LTD_Ret_Private_Purp
      LTD_Ret_All_Other LTD_Ret_Utility   LTD_Ret_Util_Water
```

```
LTD_Ret_Util_Electric   LTD_Ret_Util_Gas_Supply LTD_Ret_Util_Transit
      LTD_Ret_General   LTD_Ret_Gen_Elem_Educ   LTD_Ret_Gen_Other_Educ
      LTD_Ret_Gen_Other_NEC   Total_LTD_Ret_FFC LTD_Ret_FFC_Utility
      LTD_Ret_FFC_Water_Util  LTD_Ret_FFC_Elec_Util   LTD_Ret_FFC_Gas_Util
      LTD_Ret_FFC_Trans_Util  LTD_Ret_FFC_General     LTD_Ret_FFC_Elem_Educ
      LTD_Ret_FFC_Other_Educ  LTD_Ret_FFC_Other_NEC   Total_LTD_Ret_NG
      LTD_Ret_NG_Utility      LTD_Ret_NG_Water_Util   LTD_Ret_NG_Elec_Util
LTD_Ret_NG_Gas_Util     LTD_Ret_NG_Trans_Util   LTD_Ret_NG_General
      LTD_Ret_NG_Elem_Educ    LTD_Ret_NG_Other_Educ   LTD_Ret_NG_Private_Purp
      LTD_Ret_NG_Other_NEC    Total_LTD_Ret_Unsp      LTD_Ret_Unsp_Utility
      LTD_Ret_Unsp_Water_Util LTD_Ret_Unsp_Elec_Utili LTD_Ret_Unsp_Gas_Util
      LTD_Ret_Unsp_Trans_Util LTD_Ret_Unsp_General    LTD_Ret_Unsp_Elem_Educ
      LTD_Ret_Unsp_Other_Educ LTD_Ret_Unsp_Other_NEC  Total_LTD_Out
      LTD_Out_Private_Purp    LTD_Out_All_Other Total_LTD_Out_Utility
LTD_Out_Util_Water      LTD_Out_Util_Electric   LTD_Out_Util_Gas_Supply
      LTD_Out_Util_Transit    LTD_Out_General   LTD_Out_Gen_Elem_Educ
      LTD_Out_Gen_Other_Educ  LTD_Out_Gen_Other_NEC   Total_LTD_Out_FFC
      LTD_Out_FFC_Utility     LTD_Out_FFC_Water_Util  LTD_Out_FFC_Elec_Util
      LTD_Out_FFC_Gas_Util    LTD_Out_FFC_Trans_Util  LTD_Out_FFC_General
      LTD_Out_FFC_Elem_Educ   LTD_Out_FFC_Other_Educ  LTD_Out_FFC_Other_NEC
      Tot_LTD_Out_NG    LTD_Out_NG_Utility      LTD_Out_NG_Water_Util
LTD_Out_NG_Elec_Util    LTD_Out_NG_Gas_Util     LTD_Out_NG_Trans_Util
      LTD_Out_NG_General      LTD_Out_NG_Elem_Educ    LTD_Out_NG_Other_Educ
      LTD_Out_NG_Private_Purp LTD_Out_NG_Other_NEC    Total_Cash___Securities
      Insur_Trust_Cash___Sec  Emp_Retire_Cash___Sec   Emp_Retire_Cash___Dep
      Emp_Retire_Total_Sec    Emp_Retire_Sec_Tot_Fed
      Emp_Retire_Sec_S_L_Secur      Emp_Retire_Sec_Tot_Nong
      Emp_Retire_Sec_Corp_Bds Emp_Retire_Sec_Corp_Stk
      Emp_Retire_Sec_Mortgages
Emp_Retire_Sec_Misc_Inv Emp_Retire_Sec_Oth_Nong Unemp_Comp_Cash___Sec
      Unemp_Comp_Bal_In_US_Trs      Unemp_Comp_Other_Balance
      Nonin_Trust_Cash___Sec  Sinking_Fd_Cash___Sec   Bond_Fd_Cash___Sec
      Oth_Nonin_Fd_Cash___Sec FunctionCode Enrollment);
      set NewData;
            * Convert variables to ones that exist in the earlier dataset.
Multiplying by 1 converts character variables to numeric formats;
            SurveyYr = SurveyYear*1;
            Year4 = Year*1;
            State_Code = id_state;
            Type_Code = id_type;
            County = id_County;
            FIPS_Code_State = FIPSstate;
            FYEndDate = FYEnd;
            YearPop = PopYear;
            SchLevCode = SchoolLevel;

            *Consolidate and name variables;
            *Revenue;
            Total_Revenue = sum(B01, B21, B22, B30, B42, B46, B50, B59, B79,
B80, B89, B91, B92, B93, B94, C21, C30, C42, C46, C50, C79, C80, C89, C91,
C92, C93, C94, D21, D30, D42, D46, D50, D79, D80, D89, D91, D92, D93, D94,
T01, T09, T10, T11, T12, T13, T14, T15, T16, T19, T20, T21, T22, T23, T24,
T25, T27, T28, T29, T40, T41, T50, T51, T53, T99, A01, A03, A09, A10, A12,
A16, A18, A21, A36, A44, A45, A50, A56, A59, A60, A61, A80, A81, A87, A89,
U01, U11, U20, U21, U30, U40, U41, U50, U95, U99, A90, A91, A92, A93, A94,
X01, X02, X05, X08, Y01, Y02, Y04, Y11, Y12, Y51, Y52);
```

```
            General_Revenue = sum(B01, B21, B22, B30, B42, B46, B50, B59,
B79, B80, B89, B91, B92, B93, B94, C21, C30, C42, C46, C50, C79, C80, C89,
C91, C92, C93, C94, D21, D30, D42, D46, D50, D79, D80, D89, D91, D92, D93,
D94, T01, T09, T10, T11, T12, T13, T14, T15, T16, T19, T20, T21, T22, T23,
T24, T25, T27, T28, T29, T40, T41, T50, T51, T53, T99, A01, A03, A09, A10,
A12, A16, A18, A21, A36, A44, A45, A50, A56, A59, A60, A61, A80, A81, A87,
A89, U01, U11, U20, U21, U30, U40, U41, U50, U95, U99);
            Gen_Rev_Own_Sources = sum(A01, A03, A09, A10, A12, A16, A18, A21,
A36, A44, A45, A50, A56, A59, A60, A61, A80, A81, A87, A89, T01, T09, T10,
T11, T12, T13, T14, T15, T16, T19, T20, T21, T22, T23, T24, T25, T27, T28,
T29, T40, T41, T50, T51, T53, T99, U01, U11, U20, U21, U30, U40, U41, U50,
U95, U99);
            Total_Rev_Own_Sources = sum(T01, T09, T10, T11, T12, T13, T14,
T15, T16, T19, T20, T21, T22, T23, T24, T25, T27, T28, T29, T40, T41, T50,
T51, T53, T99, A01, A03, A09, A10, A12, A16, A18, A21, A36, A44, A45, A50,
A56, A59, A60, A61, A80, A81, A87, A89, U01, U11, U20, U21, U30, U40, U41,
U50, U95, U99, A90, A91, A92, A93, A94, X01, X02, X05, X08, Y01, Y02, Y04,
Y11, Y12, Y51, Y52);

            *Taxes;
            Total_Taxes = sum(T01, T09, T10, T11, T12, T13, T14, T15, T16,
T19, T20, T21, T22, T23, T24, T25, T27, T28, T29, T40, T41, T50, T51, T53,
T99);
            Property_Tax = T01;
            Tot_Sales___Gr_Rec_Tax = sum(T09, T10, T11, T12, T13, T14, T15,
T16, T19);
            Total_Gen_Sales_Tax = T09;
            Total_Select_Sales_Tax = sum(T10, T11, T12, T13, T14, T15, T16,
T19);
            Alcoholic_Beverage_Tax = T10;
            Amusement_Tax = T11;
            Insurance_Premium_Tax = T12;
            Motor_Fuels_Tax = T13;
            Pari_mutuels_Tax = T14;
            Public_Utility_Tax = T15;
            Tobacco_Tax = T16;
            Other_Select_Sales_Tax = T19;

            *Licenses;
            Total_License_Taxes = sum(T20 , T21 , T22 , T23 , T24 , T25 , T27
, T28 , T29);
            Alcoholic_Beverage_Lic = T20;
            Amusement_License = T21;
            Corporation_License = T22;
            Hunting___Fishing_License = T23;
            Motor_Vehicle_License = T24;
            Motor_Veh_Oper_License = T25;
            Public_Utility_License = T27;
            Occup_and_Bus_Lic_NEC = T28;
            Other_License_Taxes = T29;

            *Income Taxes;
            Total_Income_Taxes = sum(T40 , T41 , T50 , T51 , T53 , T99);
            Individual_Income_Tax = T40;
            Corp_Net_Income_Tax = T41;
            Death_and_Gift_Tax = T50;
            Docum_and_Stock_Tr_Tax = T51;
```

```
            Severance_Tax = T53;
            Taxes_NEC = T99;

            * Intergovernmental Revenue;
            Total_IG_Revenue = sum(B01, B21, B22, B30, B42, B46, B50, B59,
B79, B80, B89, B91, B92, B93, B94, C21, C30, C42, C46, C50, C79, C80, C89,
C91, C92, C93, C94, D21, D30, D42, D46, D50, D79, D80, D89, D91, D92, D93,
D94);

            * Federal;
            Total_Fed_IG_Revenue = sum(B01, B21, B22, B30, B42, B46, B50,
B59, B79, B80, B89, B91, B92, B93, B94);
            Fed_IGR_Air_Transport = B01;
            Fed_IGR_Education = B21;
            Fed_IGR_Emp_Sec_Adm = B22;
            Fed_IGR_Gen_Rev_Shar = .; *Obsolete after 1987;
            Fed_IGR_Gen_Support = B30;
            Fed_IGR_Health___Hos = B42;
            Fed_IGR_Highways = B46;
            Fed_IGR_Transit_Sub = B94;
            Fed_IGR_Hous_Com_Dev = B50;
            Fed_IGR_Natural_Res = B59;
            Fed_IGR_Public_Welf = B79;
            Fed_IGR_Sewerage = B80;
            Fed_IGR_Other = B89;

            * State;
            Total_State_IG_Revenue = sum(C21, C30, C42, C46, C50, C79, C80,
C89, C91, C92, C93, C94);
            State_IGR_Education = C21;
            State_IGR_Tax_Relief = .; *Obsolete after 1987;
            State_IGR_Oth_Gen_Sup = C30;
            State_IGR_Health___Hos = C42;
            State_IGR_Highways = C46;
            State_IGR_Transit_Sub = C94;
            State_IGR_Hous_Com_Dev = C50;
            State_IGR_Public_Welf = C79;
            State_IGR_Sewerage = C80;
            State_IGR_Other = C89;

            * Local;
            Tot_Local_IG_Rev = sum(D21, D30, D42, D46, D50, D79, D80, D89,
D91, D92, D93, D94);
            Local_IGR_InterSchool_Aid = D11;
            Local_IGR_Other_Education = D21;
            Local_IGR_Oth_Gen_Sup = D30;
            Local_IGR_Health___Hos =  D42;
            Local_IGR_Highways = D46;
            Local_IGR_Transit_Sub =  D94;
            Local_IGR_Hous_Com_Dev =  D50;
            Local_IGR_Public_Welf = D79;
            Local_IGR_Sewerage = D80;
            Local_IGR_Other = D89;

            * Charges and Misc Revenue;
```

```
            Tot_Chgs_and_Misc_Rev = sum(A01, A03, A09, A10, A12, A16, A18,
A21, A36, A44, A45, A50, A56, A59, A60, A61, A80, A81, A87, A89, U01, U11,
U20, U21, U30, U40, U41, U50, U95, U99);

            * Charges;
            Total_General_Charges = sum(A01, A03, A09, A10, A12, A16, A18,
A21, A36, A44, A45, A50, A56, A59, A60, A61, A80, A81, A87, A89);
            Chg_Air_Transportation = A01;
            Chg_Misc_Com_Activ = A03;
            Chg_Total_Education = sum(A09, A10, A12, A16, A18, A21);
            Chg_Elem_Ed_Sch_Lunch = A09;
            Chg_Elem_Ed_Tuition = A10;
            Chg_Elem_Ed_NEC = A12;
            Chg_Total_High_Ed = sum(A16, A18);
            Chg_Hospitals = A36;
            Chg_Regular_Highways = A44;
            Chg_Toll_Highways = A45;
            Chg_Housing___Comm_Dev = A50;
            Chg_Total_Nat_Res = sum(A56 , A59);
            Chg_Parking = A60;
            Chg_Parks___Recreation = A61;
            Chg_Sewerage = A80;
            Chg_Solid_Waste_Mgmt = A81;
            Chg_Water_Transport = A87;
            Chg_All_Other_NEC = A89;

            * Misc Revenue;
            Misc_General_Revenue = sum(U01, U11, U20, U21, U30, U40, U41,
U50, U95, U99);
            Special_Assessments = U01;
            Prop_Sale_Hous_Com_Dev = .; *Deleted as of 2005 and reported in
U11 Prop_Sale_Other;
            Prop_Sale_Other = U11;
            Interest_Revenue = U20;
            Fines_and_Forfeits = U30;
            Rents_and_Royalties = sum(U40 , U41);
            Net_Lottery_Revenue = U95;
            Misc_General_Rev_NEC = U99;

            * Liquor and Utilities;
            Liquor_Stores_Revenue = A90;
            Total_Utility_Revenue = sum(A91, A92, A93, A94);
            Water_Utility_Revenue = A91;
            Electric_Utility_Rev = A92;
            Gas_Utility_Rev = A93;
            Transit_Utility_Rev = A94;

            * Insurance Trusts;
            Total_Insur_Trust_Rev = sum(X01, X02, X05, X08, Y01, Y02, Y04,
Y11, Y12, Y51, Y52);
            Total_Insur_Trust_Ctrb = sum(X01, X02, X05, Y01);
            Tot_Ins_Trust_Inv_Rev = sum(X08 , Y02);

            *Retirement Plan Data;
            Total_Emp_Ret_Rev = sum(X01, X02, X05, X08);
            Emp_Ret_Total_Ctrib = sum(X01, X02, X05);
            Emp_Ret_Loc_Emp_Ctrib = X01;
```

```
            Emp_Ret_Loc_To_Loc_Sys = X04;
            Emp_Ret_From_Other_Gov = X05;
            Emp_Ret_Sta_To_Sta_Ctr = X06;
            Emp_Ret_Int_Rev = X08;
            Emp_Ret_Other_Earnings = .; *Consolidated with X08
Emp_Ret_Int_Rev in 1990;

            * Old data does not have Worker's Comp information;

            * Unemployment Revenue;
            Total_Unemp_Rev = sum(Y01, Y02, Y04);
            Unemp_Payroll_Tax = Y01;
            Unemp_Int_Revenue = Y02;
            Unemp_Federal_Advances = Y04;

            *Expenses;
            Total_Expenditure = sum(E01, E03, E04, E05, E12, E16, E18, E21,
E22, E23, E24, E25, E26, E29, E31, E32, E36, E44, E44, E45, E50, E52, E55,
E56, E59, E60, E61, E62, E66, E74, E75, E77, E79, E80, E81, E85, E87, E89,
E90, E91, E92, E93, E94, I89, I91, I92, I93, I94, J19, J67, J68, J85, X11,
X12, Y05, Y06, Y14, Y53, F01, F03, F04, F05, F12, F16, F18, F21, F22, F23,
F24, F25, F26, F29, F31, F32, F36, F44, F45, F50, F52, F55, F56, F59, F60,
F61, F62, F66, F77, F79, F80, F81, F85, F87, F89, F90, F91, F92, F93, F94,
G01, G03, G04, G05, G12, G16, G18, G21, G22, G23, G24, G25, G26, G29, G31,
G32, G36, G44, G45, G50, G52, G55, G56, G59, G60, G61, G62, G66, G77, G79,
G80, G81, G85, G87, G89, G90, G91, G92, G93, G94, L01, L04, L05, L12, L18,
L23, L25, L29, L32, L36, L44, L52, L59, L60, L61, L62, L66, L67, L79, L80,
L81, L87, L89, L91, L92, L93, L94, M01, M04, M05, M12, M18, M21, M23, M24,
M25, M29, M30, M32, M36, M44, M50, M52, M55, M56, M59, M60, M61, M62, M66,
M67, M68, M79, M80, M81, M87, M89, M91, M92, M93, M94, Q12, Q18, S67, S74,
S89);

            *Totals;
            Total_IG_Expenditure = sum(L01, L04, L05, L12, L18, L23, L25,
L29, L32, L36, L44, L50, L52, L59, L60, L61, L62, L66, L67, L79, L80, L81,
L87, L89, L91, L92, L93, L94, M01, M04, M05, M12, M18, M21, M23, M24, M25,
M29, M30, M32, M36, M44, M50, M52, M52, M55, M56, M59, M60, M61, M62, M66,
M67, M68, M79, M80, M81, M87, M89, M91, M92, M93, M94, Q12, Q18, S67, S89);
            Direct_Expenditure = sum(E01, E03, E04, E05, E12, E16, E18, E21,
E22, E23, E24, E25, E26, E29, E31, E32, E36, E44, E45, E50, E52, E55, E56,
E59, E60, E61, E62, E66, E74, E75, E77, E79, E80, E81, E85, E87, E89, E90,
E91, E92, E93, E94, F01, F03, F04, F05, F12, F16, F18, F21, F22, F23, F24,
F25, F26, F29, F31, F32, F36, F44, F45, F50, F52, F55, F56, F59, F60, F61,
F62, F66, F77, F79, F80, F81, F85, F87, F89, F90, F91, F92, F93, F94, G01,
G03, G04, G05, G12, G16, G18, G21, G22, G23, G24, G25, G26, G29, G31, G32,
G36, G44, G45, G50, G52, G55, G56, G59, G60, G61, G62, G66, G77, G79, G80,
G81, G85, G87, G89, G90, G91, G92, G93, G94, X11, X12, Y05, Y06, Y14, Y53,
J19, J67, J68, J85, I89, I91, I92, I93, I94);
            Total_Current_Oper = sum(E01, E03, E04, E05, E12, E16, E18, E21,
E22, E23, E24, E25, E26, E29, E31, E32, E36, E44, E45, E50, E52, E55, E56,
E59, E60, E61, E62, E66, E74, E75, E77, E79, E80, E81, E85, E87, E89, E90,
E91, E92, E93, E94);
            Total_Capital_Outlays = sum(F01, F03, F04, F05, F12, F16, F18,
F21, F22, F23, F24, F25, F26, F29, F31, F32, F36, F44, F45, F50, F52, F55,
F56, F59, F60, F61, F62, F66, F77, F79, F80, F81, F85, F87, F89, F90, F91,
F92, F93, F94, G01, G03, G04, G05, G12, G16, G18, G21, G22, G23, G24, G25,
```

```
G26, G29, G31, G32, G36, G44, G45, G50, G52, G55, G56, G59, G60, G61, G62,
G66, G77, G79, G80, G81, G85, G87, G89, G90, G91, G92, G93, G94);
            Total_Construction = sum(F01, F03, F04, F05, F12, F16, F18, F21,
F22, F23, F24, F25, F26, F29, F31, F32, F36, F44, F45, F50, F52, F55, F56,
F59, F60, F61, F62, F66, F77, F79, F80, F81, F85, F87, F89, F90, F91, F92,
F93, F94);
            Tot_Assist___Subsidies = sum(J19, J67, J68, J85);
            Total_Interest_on_Debt = sum(I89, I91, I92, I93, I94);
            Total_Insur_Trust_Ben = sum(X11, X12, Y05, Y06, Y14, Y53);
            Total_Salaries___Wages = Z00;
            Total_Current_Expend = sum(Total_Expenditure, -
Total_Capital_Outlays);

            * General Expenses;
            General_Expenditure = sum(E01, E03, E04, E05, E12, E16, E18, E21,
E22, E23, E24, E25, E26, E29, E31, E32, E36, E44, E45, E50, E52, E55, E56,
E59, E60, E61, E62, E66, E74, E75, E77, E79, E80, E81, E85, E87, E89, E90,
E91, E92, E93, E94, F01, F03, F04, F05, F12, F16, F18, F21, F22, F23, F24,
F25, F26, F29, F31, F32, F36, F44, F45, F50, F52, F55, F56, F59, F60, F61,
F62, F66, F77, F79, F80, F81, F85, F87, F89, F90, F91, F92, F93, F94, G01,
G03, G04, G05, G12, G16, G18, G21, G22, G23, G24, G25, G26, G29, G31, G32,
G36, G44, G45, G50, G52, G55, G56, G59, G60, G61, G62, G66, G77, G79, G80,
G81, G85, G87, G89, G90, G91, G92, G93, G94, X11, X12, Y05, Y06, Y14, Y53,
J19, J67, J68, J85, I89, I91, I92, I93, I94);
            IG_Exp_To_State_Govt = sum(L01, L04, L05, L12, L18, L23, L25,
L29, L32, L36, L44, L50, L52, L59, L60, L61, L62, L66, L67, L79, L80, L81,
L87, L89, L91, L92, L93, L94);
            IG_Exp_To_Local_Govts = sum(M01, M04, M05, M12, M18, M21, M23,
M24, M25, M29, M30, M32, M36, M44, M50, M52, M52, M55, M56, M59, M60, M61,
M62, M66, M67, M68, M79, M80, M81, M87, M89, M91, M92, M93, M94);
            IG_Exp_To_Federal_Govt = sum(S67, S74, S89);
            Direct_General_Expend = sum(E01, E03, E04, E05, E12, E16, E18,
E21, E22, E23, E24, E25, E26, E29, E31, E32, E36, E44, E45, E50, E52, E55,
E56, E59, E60, E61, E62, E66, E74, E75, E77, E79, E80, E81, E85, E87, E89,
E90, E91, E92, E93, E94, F01, F03, F04, F05, F12, F16, F18, F21, F22, F23,
F24, F25, F26, F29, F31, F32, F36, F44, F45, F50, F52, F55, F56, F59, F60,
F61, F62, F66, F77, F79, F80, F81, F85, F87, F89, F90, F91, F92, F93, F94,
G01, G03, G04, G05, G12, G16, G18, G21, G22, G23, G24, G25, G26, G29, G31,
G32, G36, G44, G45, G50, G52, G55, G56, G59, G60, G61, G62, G66, G77, G79,
G80, G81, G85, G87, G89, G90, G91, G92, G93, G94, X11, X12, Y05, Y06, Y14,
Y53, J19, J67, J68, J85, I89, I91, I92, I93, I94);
            General_Current_Oper = sum(E01, E03, E04, E05, E12, E16, E18,
E21, E22, E23, E24, E25, E26, E29, E31, E32, E36, E44, E45, E50, E52, E55,
E56, E59, E60, E61, E62, E66, E74, E75, E77, E79, E80, E81, E85, E87, E89,
E90, E91, E92, E93, E94);
            General_Capital_Outlay = sum(F01, F03, F04, F05, F12, F16, F18,
F21, F22, F23, F24, F25, F26, F29, F31, F32, F36, F44, F45, F50, F52, F55,
F56, F59, F60, F61, F62, F66, F77, F79, F80, F81, F85, F87, F89, G01, G03,
G04, G05, G12, G16, G18, G21, G22, G23, G24, G25, G26, G29, G31, G32, G36,
G44, G45, G50, G52, G55, G56, G59, G60, G61, G62, G66, G77, G79, G80, G81,
G85, G87, G89);
            General_Construction = sum(F01, F03, F04, F05, F12, F16, F18,
F21, F22, F23, F24, F25, F26, F29, F31, F32, F36, F44, F45, F50, F52, F55,
F56, F59, F60, F61, F62, F66, F77, F79, F80, F81, F85, F87, F89, F90, F91,
F92, F93, F94);
            General_Assist___Sub = Tot_Assist___Subsidies;
            General_Debt_Interest = I89;
```

```
            General_Current_Expend = sum(General_Expenditure, -
General_Capital_Outlay);

            * Air Transport;
            Air_Trans_Total_Expend = sum(E01 , F01 , G01 , L01 , M01);
            Air_Trans_Direct_Expend = sum(E01 , F01 , G01);
            Air_Trans_Cap_Outlay = sum(F01 , G01);
            Air_Trans_Construction = F01;
            Air_Trans_IG_To_State = L01;
            Air_Trans_IG_Local_Govts = M01;

            * Misc Commercial Activities;
            Misc_Com_Activ_Tot_Exp = sum(E03 , F03 , G03);
            Misc_Com_Activ_Cap_Out = sum(F03 , G03);
            Misc_Com_Activ_Constr = F03;

            * Correctional Institutions;
            Correct_Total_Exp = sum(E04 , F04 , G04 , E05 , F05 , G05 , L04 ,
L05 , M04 , M05);
            Correct_Direct_Exp = sum(E04 , E05 , F04 , G04 , F05 , G05);
            Correct_Cap_Outlay = sum(F04 , G04 , F05 , G05);
            Correct_Construct = sum(F04 , F05);
            Correct_IG_To_St = sum(L04 , L05);
            Correct_IG_Loc_Govts = sum(M04 , M05);

            * Missing national defense code 06;

            * Education;
            Total_Educ_Total_Exp = sum(E12, F12, G12, E16, F16, G16, E18,
F18, G18, J19, E21, F21, G21, L12, M12, Q12, L18, M18, L21, M21);
            Total_Educ_Direct_Exp = sum(E12, F12, G12, E16, F16, G16, E18,
F18, G18, J19, E21, F21, G21);
            Total_Educ_Assist___Sub = J19;
            Total_Educ_Cap_Outlay = sum(F12, F16, F18, F21, G12, G16, G18,
G21);
            Total_Educ_Construct = sum(F12 , F16 , F18 , F21);
            * Elementary and Secondary Education;
            Elem_Educ_Total_Exp = sum(E12, F12, G12, L12, M12, Q12);
            Elem_Educ_Direct_Exp = sum(E12, F12, G12);
            Elem_Educ_Cap_Outlay = sum(F12, G12);
            Elem_Educ_Construction = F12;
            Elem_Educ_IG_To_State = L12;
            Elem_Educ_IG_Local_Govts = M12;
            Elem_Educ_IG_Sch_to_Sch = Q12;
            * Higher Education;
            Higher_Ed_Total_Exp = sum(E16, E18, F16, F18, G16, G18, L18,
M18);
            Higher_Ed_Direct_Exp = sum(E16, E18, F16, F18, G16, G18);
            Higher_Ed_Cap_Outlay = sum(F16, F18, G16, G18);
            Higher_Ed_Construct = sum(F16, F18);
            Higher_Ed_IG_To_St = L18;
            Higher_Ed_IG_Loc_Govts = M18;
            * Education not otherwise classified;
            Educ_NEC_Total_Expend = sum(E21, F21, G21, L21, M21);
            Educ_NEC_Direct_Expend = sum(E21, F21, G21);
            Educ_NEC_Assistance = .;  *The prior code, E19 no longer exists
in the data;
```

```
Educ_NEC_Cap_Outlay = sum(F21, G21);
Educ_NEC_Construction = F21;
Educ_NEC_IG_To_State = L21;
Educ_NEC_IG_Local_Govts = M21;

* Missing post offices code 14;

*Employment Security Administration;
Emp_Sec_Adm_Direct_Exp = sum(E22 , F22 , G22);
Emp_Sec_Adm_Cap_Outlay = sum(F22 , G22);
Emp_Sec_Adm_Construct = F22;

*Financial Administration;
Fin_Admin_Total_Exp = sum(E23, F23, G23, L23, M23);
Fin_Admin_Direct_Exp = sum(E23, F23, G23);
Fin_Admin_Cap_Outlay = sum(F23, G23);
Fin_Admin_Construction = F23;
Fin_Admin_IG_To_State = L23;
Fin_Admin_IG_Local_Govts = M23;

*Fire protection;
Fire_Prot_Total_Expend = sum(E24, F24, G24, L24, M24);
Fire_Prot_Direct_Exp = sum(E24, F24, G24);
Fire_Prot_Cap_Outlay = sum(F24, G24);
Fire_Prot_Construction = F24;
Fire_Prot_IG_To_State = L24;
Fire_Prot_IG_Local_Govts = M24;

*Judicial Expenditures;
Judicial_Total_Expend = sum(E25, F25, G25, L25, M25);
Judicial_Direct_Expend = sum(E25, F25, G25);
Judicial_Cap_Outlay = sum(F25, G25);
Judicial_Construction = F25;
Judicial_IG_To_State = L25;
Judicial_IG_Local_Govts = M25;

*Central Staff Services;
Cen_Staff_Total_Expend = sum(E29, F29, G29, L29, M29);
Cen_Staff_Direct_Exp = sum(E29, F29, G29);
Cen_Staff_Cap_Outlay = sum(F29, G29);
Cen_Staff_Construction = F29;
Cen_Staff_IG_To_State = L29;
Cen_Staff_IG_Local_Govts = M29;

* General Public Buildings;
Gen_Pub_Bldg_Total_Exp = sum(E31, F31, G31);
Gen_Pub_Bldg_Cap_Out = sum(F31, G31);
Gen_Pub_Bldg_Construct = F31;

* Health;
Health_Total_Expend = sum(E32, F32, G32, L32, M32);
Health_Direct_Expend = sum(E32, F32, G32);
Health_Capital_Outlay = sum(F32, G32);
Health_Construction = F32;
Health_IG_To_State = L32;
Health_IG_Local_Govts = M32;
```

```
*Hospitals;
Total_Hospital_Total_Exp = sum(E36, F36, G36, L36, M36);
Total_Hospital_Dir_Exp = sum(E36, F36, G36);
Total_Hospital_Cap_Out = sum(F36, G36);
Total_Hospital_Construct = F36;
Total_Hospital_IG_To_State = L36;
Total_Hospital_IG_Loc_Govts = M36;

*Federal Owned Hospitals - Veterans;
Own_Hospital_Total_Exp = sum(E37, F37, G37);
Own_Hospital_Cap_Out = sum(F37, G37);
Own_Hospital_Construct = F37;

* Other Hospital Expenses - Federal but not veterans;
Hosp_Other_Total_Exp = sum(E39, F39, G39, L39, M39);
Hosp_Other_Direct_Exp = sum(E39, F39, G39);
Hosp_Other_Cap_Outlay = sum(F39, G39);
Hosp_Other_Construct = F39;
Hosp_Other_IG_To_State = L39;
Hosp_Other_IG_Loc_Govts = M39;

* Highways;
Total_Highways_Tot_Exp = sum(E44, F44, G44, E45, F45, G45, L44,
M44);

Total_Highways_Dir_Exp = sum(E44, F44, G44, E45, F45, G45);
Total_Highways_Cap_Out = sum(F44, G44, F45, G45);
Total_Highways_Construct = sum(F44, F45);

* Non-Toll Highways;
Regular_Hwy_Total_Exp = sum(E44, F44, G44, L44, M44);
Regular_Hwy_Direct_Exp = sum(E44, F44, G44);
Regular_Hwy_Cap_Outlay = sum(F44, G44);
Regular_Hwy_Construct = F44;
Regular_Hwy_IG_To_Sta = L44;
Regular_Hwy_IG_Loc_Govts = M44;

* Toll Highways;
Toll_Hwy_Total_Expend = sum(E45, F45, G45);
Toll_Hwy_Cap_Outlay = sum(F45, G45);
Toll_Hwy_Construction = F45;

* Transit Subsidies - These data were eliminated in the 2005
data;

Transit_Sub_Total_Exp = .;
Transit_Sub_Direct_Sub = .;
Transit_Sub_IG_To_Sta = .;
Transit_Sub_IG_Loc_Govts = .;
Transit_Sub_To_Own_Sys = .;

*Housing and Community Development;
Hous___Com_Total_Exp = sum(E50, F50, G50, L50, M50);
Hous___Com_Direct_Exp = sum(E50, F50, G50);
Hous___Com_Cap_Outlay = sum(F50, G50);
Hous___Com_Construct = F50;
Hous___Com_IG_To_State = L50;
Hous___Com_IG_Loc_Govts = M50;
```

```
            * Libraries;
            Libraries_Total_Expend = sum(E52, F52, G52, L52, M52);
            Libraries_Direct_Exp = sum(E52, F52, G52);
            Libraries_Cap_Outlay = sum(F52, G52);
            Libraries_Construction = F52;
            Libraries_IG_To_State = L52;
            Libraries_IG_Local_Govts = M52;

            *Natural Resources;
            Natural_Res_Total_Exp = sum(E55, F55, G55, M55, E56, F56, G56,
M56, E59, F59, G59, L59, M59);
            Natural_Res_Direct_Exp = sum(E55, F55, G55, E56, F56, G56, E59,
F59, G59);
            Natural_Res_Cap_Outlay = sum(F55, G55, F56, G56, F59, G59);
            Natural_Res_Construct = sum(F55, F56, F59);
            Natural_Res_IG_To_Sta = L59;
            Natural_Res_IG_Loc_Govts = sum(M55, M56 , M59);

            * Parking Facilities;
            Parking_Total_Expend = sum(E60, F60, G60, L60, M60);
            Parking_Direct_Expend = sum(E60, F60, G60);
            Parking_Capital_Outlay = sum(F60, G60);
            Parking_Construction = F60;
            Parking_IG_To_State = L60;
            Parking_IG_Local_Govts = M60;

            * Parks and Recreation;
            Parks___Rec_Total_Exp = sum(E61, F61, G61, L61, M61);
            Parks___Rec_Direct_Exp = sum(E61, F61, G61);
            Parks___Rec_Cap_Outlay = sum(F61, G61);
            Parks___Rec_Construct = F61;
            Parks___Rec_IG_To_Sta = L61;
            Parks___Rec_IG_Loc_Govts = M61;

            * Police Protection;
            Police_Prot_Total_Exp = sum(E62, F62, G62, L62, M62);
            Police_Prot_Direct_Exp = sum(E62, F62, G62);
            Police_Prot_Cap_Outlay = sum(F62, G62);
            Police_Prot_Construct = F62;
            Police_Prot_IG_To_Sta = L62;
            Police_Prot_IG_Loc_Govts = M62;

            * Protective Inspection and Regulation;
            Prot_Insp_Total_Exp = sum(E66, F66, G66, L66, M66);
            Prot_Insp_Direct_Exp = sum(E66, F66, G66);
            Prot_Insp_Cap_Outlay = sum(F66, G66);
            Prot_Insp_Construction = F66;
            Prot_Insp_IG_To_State = L66;
            Prot_Insp_IG_Local_Govts = M66;

            * Public Welfare;
            Public_Welf_Total_Exp = sum(J67, L67, M67, J68 , M68, E74, E75,
S74, E77, F77, G77, E79, F79, G79, L79, M79);
            Public_Welf_Direct_Exp = sum(J67, J68 , E74, E75, E77, F77, G77,
E79, F79, G79);
            Public_Welf_Cash_Asst = sum(J67, J68, M67, M68);
            Public_Welf_Cap_Outlay = sum(F77, G77, F79, G79);
```

```
Public_Welf_Construct = sum(F77, F79);

* Public Welfare-Categorical Assistance Programs;
Welf_Categ_Total_Exp = sum(J67, L67, M67);
Welf_Categ_Cash_Assist = J67;
Welf_Categ_IG_To_State = L67;
Welf_Categ_IG_Loc_Govts =  M67;

* Public Welfare-Cash assistance payments;
Welf_Cash_Total_Exp = sum(J68 , M68);
Welf_Cash_Cash_Assist = J68;
Welf_Cash_IG_Local_Govts = M68;

* Public Welfare-Vendor Payments;
Welf_Vend_Pmts_Medical = E74;
Welf_Vend_Pmts_NEC = E75;

* State Share of Medicare Part D;
Welf_State_Share_Part_D = S74;

* Public Welfare-Institutions;
Welf_Ins_Total_Exp = sum(E77, F77, G77);
Welf_Ins_Cap_Outlay = sum(F77, G77);
Welf_Ins_Construction = F77;

*Public Welfare not elsewhere classified;
Welf_NEC_Total_Expend = sum(E79, F79, G79, L79, M79);
Welf_NEC_Direct_Expend = sum(E79, F79, G79);
Welf_NEC_Cap_Outlay = sum(F79, G79);
Welf_NEC_Construction = F79;
Welf_NEC_IG_To_State = L79;
Welf_NEC_IG_Local_Govts = M79;

*Sewerage;
Sewerage_Total_Expend = sum(E80, F80, G80, L80, M80);
Sewerage_Direct_Expend = sum(E80, F80, G80);
Sewerage_Cap_Outlay = sum(F80, G80);
Sewerage_Construction = F80;
Sewerage_IG_To_State = L80;
Sewerage_IG_Local_Govts = M80;

*Solid Waste Management;
SW_Mgmt_Total_Expend = sum(E81, F81, G81, L81, M81);
SW_Mgmt_Direct_Expend = sum(E81, F81, G81);
SW_Mgmt_Capital_Outlay = sum(F81, G81);
SW_Mgmt_Construction = F81;
SW_Mgmt_IG_To_State = L81;
SW_Mgmt_IG_Local_Govts = M81;

*Sea and Inland Port Facilities;
Water_Trans_Total_Exp = sum(E87, F87, G87, L87, M87);
Water_Trans_Direct_Exp = sum(E87, F87, G87);
Water_Trans_Cap_Outlay = sum(F87, G87);
Water_Trans_Construct = F87;
Water_Trans_IG_To_Sta = L87;
Water_Trans_IG_Loc_Govts = M87;
```

```
*Interest on General Debt;
Interest_on_Gen_Debt = I89;

* General Expenditure not elsewhere classified;
General_NEC_Total_Exp = sum(E89, F89, G89, L89, M89, S89, J89);
General_NEC_Direct_Exp = sum(E89, F89, G89, J89);
VetBonus = J89;
General_NEC_Cap_Outlay = sum(F89, G89);
General_NEC_Construct = F89;
General_NEC_IG_To_St = L89;
General_NEC_IG_Loc_Govts = M89;
General_NEC_IG_To_Fed = S89;

* Liquor Stores;
Liquor_Stores_Tot_Exp = sum(E90, F90, G90);
Liquor_Stores_Cap_Out = sum(F90, G90);
Liquor_Stores_Constr = F90;

* Total Utilities;
Total_Util_Total_Exp = sum(E91, I91, F91, G91, L91, M91, E92,
I92, F92, G92, L92, M92, E93, I93, F93, G93, L93, M93, E94, I94, F94, G94,
L94, M94);
Total_Util_Inter_Exp = sum(I91, I92, I93, I94);
Total_Util_Cap_Outlay = sum(F91, F92, F93, F94, G91, G92, G93,
G94);
Total_Util_Construct = sum(F91, F92, F93, F94);

* Water Supply Utilities;
Water_Util_Total_Exp = sum(E91, I91, F91, G91, L91, M91);
Water_Util_Inter_Exp = I91;
Water_Util_Cap_Outlay = sum(F91, G91);
Water_Util_Construct = F91;

* Electric Power Utilities;
Elec_Util_Total_Exp = sum(E92, I92, F92, G92, L92, M92);
Elec_Util_Inter_Exp = I92;
Elec_Util_Cap_Outlay = sum(F92, G92);
Elec_Util_Construct = F92;

* Gas Supply Utilities;
Gas_Util_Total_Exp = sum(E93, I93, F93, G93, L93, M93);
Gas_Util_Inter_Exp = I93;
Gas_Util_Cap_Outlay = sum(F93, G93);
Gas_Util_Construct = F93;

* Transit System Utilities;
Trans_Util_Total_Exp = sum(E94, I94, F94, G94, L94, M94);
Trans_Util_Inter_Exp = I94;
Trans_Util_Cap_Outlay = sum(F94, G94);
Trans_Util_Construct = F94;

* Employee Retirement;
Emp_Ret_Total_Expend = sum(X11, X12);
Emp_Ret_Benefit_Paymts = X11;
Emp_Ret_Withdrawals = X12;
Emp_Ret_Other_Paymts = .; *This code used to be X14 is now
obsolete since 2002 represented realized losses and is coded elsewhere;
```

```
* Unemployment Compensation;
Unemp_Comp_Total_Exp = sum(Y05, Y06);
Unemp_Comp_Ben_Paymts = Y05;
Unemp_Ext___Spec_Pmts = Y06;

* Debt totals;
Total_Debt_Outstanding = sum(_44T, _49U, _64V);
Total_Long_Term_Debt_Out = sum(_44T, _49U);
ST_Debt_End_of_Year = _64V;

* Beginning Long Term Debt Outstanding;
Total_Beg_LTD_Out = sum(_19T, _19U);
Beg_LTD_Out_Private_Purp = _19T;
Beg_LTD_Out_All_Other = _19U;
Beg_LTD_Out_Utility = .; *All detailed debt codes were
discontinued in 2005;
Beg_LTD_Out_Water_Util = .;
Beg_LTD_Out_Elec_Util = .;
Beg_LTD_Out_Gas_Util = .;
Beg_LTD_Out_Trans_Util = .;
Beg_LTD_Out_General = .;
Beg_LTD_Out_Education = .;
Beg_LTD_Out_Priv_Purp = .;
Beg_LTD_Out_Other_NEC = .;

* Long Term Debt Issued;
Total_LTD_Issued = sum(_24T, _29U);
LTD_Iss_Private_Purp = _24T;
LTD_Iss_All_Other = _29U;
LTD_Iss_Utility = .;
LTD_Iss_Util_Water = .;
LTD_Iss_Util_Electric = .;
LTD_Iss_Util_Gas_Supply = .;
LTD_Iss_Util_Transit = .;
LTD_Iss_General = .;
LTD_Iss_Gen_Elem_Educ = .;
LTD_Iss_Gen_Other_Educ = .;
LTD_Iss_Gen_Other_NEC = .;

* Long-Term Debt Issued, Full-Faith and Credit; *Codes
discontinued in 2005;
Total_LTD_Iss_FFC = .;
LTD_Iss_FFC_Utility = .;
LTD_Iss_FFC_Water_Util = .;
LTD_Iss_FFC_Elec_Util = .;
LTD_Iss_FFC_Gas_Util = .;
LTD_Iss_FFC_Trans_Util = .;
LTD_Iss_FFC_General = .;
LTD_Iss_FFC_Elem_Educ = .;
LTD_Iss_FFC_Other_Educ = .;
LTD_Iss_FFC_Other_NEC = .;

* Long-Term Debt Issued, Nonguaranteed;
Total_LTD_Iss_NG =  .;
LTD_Iss_NG_Utility = .;
LTD_Iss_NG_Water_Util = .;
```

```
LTD_Iss_NG_Elec_Util = .;
LTD_Iss_NG_Gas_Util = .;
LTD_Iss_NG_Trans_Util = .;
LTD_Iss_NG_General = .;
LTD_Iss_NG_Elem_Educ = .;
LTD_Iss_NG_Other_Educ = .;
LTD_Iss_NG_Private_Purp = .;
LTD_Iss_NG_Other_NEC = .;

* Long-Term Debt Issued, Unspecified Issue;
Total_LTD_Iss_Unsp = .;
LTD_Iss_Unsp_Utility = .;
LTD_Iss_Unsp_Water_Util = .;
LTD_Iss_Unsp_Elec_Util = .;
LTD_Iss_Unsp_Gas_Util =  .;
LTD_Iss_Unsp_Trans_Util = .;
LTD_Iss_Unsp_General = .;
LTD_Iss_Unsp_Elem_Educ = .;
LTD_Iss_Unsp_Other_Educ = .;
LTD_Iss_Unsp_Other_NEC = .;

*Long-Term Debt Retired During Fiscal Year;
Total_LTD_Retired = sum(_34T, _39U);
LTD_Ret_Private_Purp = _34T;
LTD_Ret_All_Other = _39U;
LTD_Ret_Utility = .;
LTD_Ret_Util_Water = .;
LTD_Ret_Util_Electric = .;
LTD_Ret_Util_Gas_Supply = .;
LTD_Ret_Util_Transit = .;
LTD_Ret_General = .;
LTD_Ret_Gen_Elem_Educ = .;
LTD_Ret_Gen_Other_Educ = .;
LTD_Ret_Gen_Other_NEC = .;

* Long-Term Debt Retired, Full-Faith and Credit;
Total_LTD_Ret_FFC = .;
LTD_Ret_FFC_Utility = .;
LTD_Ret_FFC_Water_Util = .;
LTD_Ret_FFC_Elec_Util = .;
LTD_Ret_FFC_Gas_Util = .;
LTD_Ret_FFC_Trans_Util = .;
LTD_Ret_FFC_General = .;
LTD_Ret_FFC_Elem_Educ = .;
LTD_Ret_FFC_Other_Educ = .;
LTD_Ret_FFC_Other_NEC = .;

* Long-Term Debt Retired, Nonguaranteed;
Total_LTD_Ret_NG = .;
LTD_Ret_NG_Utility = .;
LTD_Ret_NG_Water_Util = .;
LTD_Ret_NG_Elec_Util = .;
LTD_Ret_NG_Gas_Util = .;
LTD_Ret_NG_Trans_Util = .;
LTD_Ret_NG_General = .;
LTD_Ret_NG_Elem_Educ = .;
LTD_Ret_NG_Other_Educ = .;
```

```
            LTD_Ret_NG_Private_Purp = .;
            LTD_Ret_NG_Other_NEC = .;

            * Long-Term Debt Retired, Unspecified;
            Total_LTD_Ret_Unsp = .;
            LTD_Ret_Unsp_Utility = .;
            LTD_Ret_Unsp_Water_Util = .;
            LTD_Ret_Unsp_Elec_Utili = .;
            LTD_Ret_Unsp_Gas_Util = .;
            LTD_Ret_Unsp_Trans_Util = .;
            LTD_Ret_Unsp_General = .;
            LTD_Ret_Unsp_Elem_Educ = .;
            LTD_Ret_Unsp_Other_Educ = .;
            LTD_Ret_Unsp_Other_NEC = .;

            * Long-Term Debt Outstanding;
            Total_LTD_Out = sum(_44T, _49U);
            LTD_Out_Private_Purp = _44T;
            LTD_Out_All_Other = _49U;
            Total_LTD_Out_Utility = .;
            LTD_Out_Util_Water = .;
            LTD_Out_Util_Electric = .;
            LTD_Out_Util_Gas_Supply = .;
            LTD_Out_Util_Transit = .;
            LTD_Out_General = .;
            LTD_Out_Gen_Elem_Educ = .;
            LTD_Out_Gen_Other_Educ = .;
            LTD_Out_Gen_Other_NEC = .;

            * Long-Term Debt Outstanding, Full-Faith and Credit;
            Total_LTD_Out_FFC = .;
            LTD_Out_FFC_Utility = .;
            LTD_Out_FFC_Water_Util = .;
            LTD_Out_FFC_Elec_Util = .;
            LTD_Out_FFC_Gas_Util = .;
            LTD_Out_FFC_Trans_Util = .;
            LTD_Out_FFC_General = .;
            LTD_Out_FFC_Elem_Educ = .;
            LTD_Out_FFC_Other_Educ = .;
            LTD_Out_FFC_Other_NEC = .;

            * Long-Term Debt Outstanding, Nonguaranteed;
            Tot_LTD_Out_NG = .;
            LTD_Out_NG_Utility = .;
            LTD_Out_NG_Water_Util = .;
            LTD_Out_NG_Elec_Util = .;
            LTD_Out_NG_Gas_Util = .;
            LTD_Out_NG_Trans_Util = .;
            LTD_Out_NG_General = .;
            LTD_Out_NG_Elem_Educ = .;
            LTD_Out_NG_Other_Educ = .;
            LTD_Out_NG_Private_Purp = .;
            LTD_Out_NG_Other_NEC = .;

            * Cash and Securities;
            Total_Cash___Securities = sum(W01, W31, W61, X21, X30, Z77, Z78,
X42, X44, X47, Y07, Y08, Y21, Y61);
```

```
            * Insurance Trust Funds Only Cash and Securities;
            Insur_Trust_Cash___Sec = sum(X21, X30, Z77, Z78, X42, X44, X47,
Y07, Y08, Y21, Y61);

            *  Employee Retirement Systems Cash and Securities;
            Emp_Retire_Cash___Sec = sum(X21, X30, X35, Z77, Z78, X42, X47,
X44);
            Emp_Retire_Cash___Dep = X21;
            Emp_Retire_Total_Sec = sum(X30, X35, Z77, Z78, X42, X47, X44);
            Emp_Retire_Sec_Tot_Fed = X30;
            Emp_Retire_Sec_S_L_Secur = X35;
            Emp_Retire_Sec_Tot_Nong = sum(Z77, Z78, X42, X47, X44);
            Emp_Retire_Sec_Corp_Bds = Z77;
            Emp_Retire_Sec_Corp_Stk = Z78;
            Emp_Retire_Sec_Mortgages = X42;
            Emp_Retire_Sec_Misc_Inv = X47;
            Emp_Retire_Sec_Oth_Nong = X44;

            * Unemployment Compensation Funds Cash and Securities;
            Unemp_Comp_Cash___Sec = sum(Y07, Y08);
            Unemp_Comp_Bal_In_US_Trs = Y07;
            Unemp_Comp_Other_Balance = Y08;

            * Other Insurance Trusts Holdings Cash and Securities;
            Nonin_Trust_Cash___Sec = sum(W01, W31, W61);

            * Sinking Funds (debt service funds) Cash and Securities;
            Sinking_Fd_Cash___Sec = W01;

            * Bond Funds Cash and Securities;
            Bond_Fd_Cash___Sec = W31;

            * All Other Noninsurance Funds Cash and Securities;
            Oth_Nonin_Fd_Cash___Sec = W61;
run;

*Merge the older data with the newer data that has had its format converted;

Proc append base=LargeData data=ConvertedNewData force;
run;

Data LargestData;
      Set LargeData;

      *Calculate several totals from revenue data and to correct for
differences between old and new data;
      Motor_Vehicle_License_Total = sum(Motor_Vehicle_License,
Motor_Veh_Oper_License);
      Fed_IGR_Gen_Support = sum(Fed_IGR_Gen_Support, Fed_IGR_Gen_Rev_Shar);
      State_IGR_Gen_Sup = sum(State_IGR_Oth_Gen_Sup, State_IGR_Tax_Relief);
      Chg_Total_Elem_Education = sum(Chg_Elem_Ed_Sch_Lunch,
Chg_Elem_Ed_Tuition, Chg_Elem_Ed_NEC);
      Chg_Highways = sum(Chg_Regular_Highways, Chg_Toll_Highways);
      Prop_Sale_Total = sum(Prop_Sale_Hous_Com_Dev, Prop_Sale_Other);
      Total_Other_Capital_Outlays = sum(Total_Capital_Outlays, -
Total_Construction);
```

```
      General_Capital_Outlay_Other = sum(General_Capital_Outlay, -
General_Construction);

      *Calculate current expenditure data for every function where it is
missing;
      Air_Trans_Current_Exp = sum(Air_Trans_Direct_Expend, -
Air_Trans_Cap_Outlay);
      Misc_Com_Activ_Current_Exp = sum(Misc_Com_Activ_Tot_Exp, -
Misc_Com_Activ_Cap_Out);
      Correct_Current_Exp = sum(Correct_Direct_Exp, -Correct_Cap_Outlay);
      Total_Educ_Current_Exp = sum(Total_Educ_Direct_Exp, -
Total_Educ_Cap_Outlay);
      Elem_Educ_Current_Exp = sum(Elem_Educ_Direct_Exp, -
Elem_Educ_Cap_Outlay);
      Higher_Ed_Current_Exp = sum(Higher_Ed_Direct_Exp, -
Higher_Ed_Cap_Outlay);
      Educ_NEC_Current_Exp = sum(Educ_NEC_Direct_Expend, -
Educ_NEC_Cap_Outlay);
      Emp_Sec_Adm_Current_Exp = sum(Emp_Sec_Adm_Direct_Exp, -
Emp_Sec_Adm_Cap_Outlay);
      Fin_Admin_Current_Exp = sum(Fin_Admin_Direct_Exp, -
Fin_Admin_Cap_Outlay);
      Fire_Prot_Current_Exp = sum(Fire_Prot_Direct_Exp, -
Fire_Prot_Cap_Outlay);
      Judicial_Current_Exp = sum(Judicial_Direct_Expend, -
Judicial_Cap_Outlay);
      Cen_Staff_Current_Exp = sum(Cen_Staff_Direct_Exp, -
Cen_Staff_Cap_Outlay);
      Gen_Pub_Bldg_Current_Exp = sum(Gen_Pub_Bldg_Total_Exp, -
Gen_Pub_Bldg_Cap_Out);
      Health_Current_Exp = sum(Health_Direct_Expend, -Health_Capital_Outlay);
      Total_Hospital_Current_Exp = sum(Total_Hospital_Dir_Exp, -
Total_Hospital_Cap_Out);
      Own_Hospital_Current_Exp = sum(Own_Hospital_Total_Exp, -
Own_Hospital_Cap_Out);
      Hosp_Other_Current_Exp = sum(Hosp_Other_Direct_Exp, -
Hosp_Other_Cap_Outlay);
      Total_Highways_Current_Exp = sum(Total_Highways_Dir_Exp, -
Total_Highways_Cap_Out);
      Regular_Hwy_Current_Exp = sum(Regular_Hwy_Direct_Exp, -
Regular_Hwy_Cap_Outlay);
      Toll_Hwy_Current_Exp = sum(Toll_Hwy_Total_Expend, -
Toll_Hwy_Cap_Outlay);
      Hous___Com_Current_Exp = sum(Hous___Com_Direct_Exp, -
Hous___Com_Cap_Outlay);
      Libraries_Current_Exp = sum(Libraries_Direct_Exp, -
Libraries_Cap_Outlay);
      Natural_Res_Current_Exp = sum(Natural_Res_Direct_Exp, -
Natural_Res_Cap_Outlay);
      Parking_Current_Exp = sum(Parking_Direct_Expend, -
Parking_Capital_Outlay);
      Parks___Rec_Current_Exp = sum(Parks___Rec_Direct_Exp, -
Parks___Rec_Cap_Outlay);
      Police_Prot_Current_Exp = sum(Police_Prot_Direct_Exp, -
Police_Prot_Cap_Outlay);
      Prot_Insp_Current_Exp = sum(Prot_Insp_Direct_Exp, -
Prot_Insp_Cap_Outlay);
```

```
	Public_Welf_Current_Exp = sum(Public_Welf_Direct_Exp, -
Public_Welf_Cap_Outlay, -Public_Welf_Cash_Asst);
	Welf_Ins_Current_Exp = sum(Welf_Ins_Total_Exp, -Welf_Ins_Cap_Outlay);
	Welf_NEC_Current_Exp = sum(Welf_NEC_Direct_Expend, -
Welf_NEC_Cap_Outlay);
	Sewerage_Current_Exp = sum(Sewerage_Direct_Expend, -
Sewerage_Cap_Outlay);
	SW_Mgmt_Current_Exp = sum(SW_Mgmt_Direct_Expend, -
SW_Mgmt_Capital_Outlay);
	Water_Trans_Current_Exp = sum(Water_Trans_Direct_Exp, -
Water_Trans_Cap_Outlay);
	General_NEC_Current_Exp = sum(General_NEC_Direct_Exp, -
General_NEC_Cap_Outlay, -VetBonus);
	Liquor_Stores_Current_Exp = sum(Liquor_Stores_Tot_Exp, -
Liquor_Stores_Cap_Out);
	Total_Util_Current_Exp = sum(Total_Util_Total_Exp, -
Total_Util_Inter_Exp, -Total_Util_Cap_Outlay);
	Water_Util_Current_Exp = sum(Water_Util_Total_Exp, -
Water_Util_Inter_Exp, -Water_Util_Cap_Outlay);
	Elec_Util_Current_Exp = sum(Elec_Util_Total_Exp, -Elec_Util_Inter_Exp,
-Elec_Util_Cap_Outlay);
	Gas_Util_Current_Exp = sum(Gas_Util_Total_Exp, -Gas_Util_Inter_Exp, -
Gas_Util_Cap_Outlay);
	Trans_Util_Current_Exp = sum(Trans_Util_Total_Exp, -
Trans_Util_Inter_Exp, -Trans_Util_Cap_Outlay);

run;

*Set the order of the variables in the largest data set.  Only the variables
to be placed at the front of the data set need to be listed in the retain
statement.;
*Also drop any variables that are unreliable according to the census, or that
are both unneeded and unavailable in the newest data.;
Data LargestData (Drop= SortCode Census_Region Weight YearDepSch YearRetire
Version ReviseDate Data_Flag JacketUnit ZeroData Imputed_Record);
	Retain SurveyYr Year4 YearofData ID IDChanged State_Code Type_Code
County Name FIPS_Code_State FYEndDate YearPop SchLevCode Population
FunctionCode Enrollment SurveyYr Year4 ID State_Code Type_Code County Name
FIPS_Code_State FYEndDate YearPop SchLevCode Population Total_Revenue
Total_Rev_Own_Sources General_Revenue Gen_Rev_Own_Sources Total_Taxes
Property_Tax Tot_Sales___Gr_Rec_Tax Total_Gen_Sales_Tax
Total_Select_Sales_Tax Alcoholic_Beverage_Tax Amusement_Tax
Insurance_Premium_Tax Motor_Fuels_Tax Pari_mutuels_Tax Public_Utility_Tax
Tobacco_Tax Other_Select_Sales_Tax Total_License_Taxes Alcoholic_Beverage_Lic
Amusement_License Corporation_License Hunting___Fishing_License
Motor_Vehicle_License Motor_Veh_Oper_License Motor_Vehicle_License_Total
Public_Utility_License Occup_and_Bus_Lic_NEC Other_License_Taxes
Total_Income_Taxes Individual_Income_Tax Corp_Net_Income_Tax
Death_and_Gift_Tax Docum_and_Stock_Tr_Tax Severance_Tax Taxes_NEC
Total_IG_Revenue Total_Fed_IG_Revenue Fed_IGR_Air_Transport Fed_IGR_Education
Fed_IGR_Emp_Sec_Adm Fed_IGR_Gen_Rev_Shar Fed_IGR_Gen_Support
Fed_IGR_Health___Hos Fed_IGR_Highways
Fed_IGR_Transit_Sub Fed_IGR_Hous_Com_Dev  Fed_IGR_Natural_Res
Fed_IGR_Public_Welf Fed_IGR_Sewerage Fed_IGR_Other Total_State_IG_Revenue
State_IGR_Education State_IGR_Tax_Relief State_IGR_Oth_Gen_Sup
State_IGR_Gen_Sup State_IGR_Health___Hos State_IGR_Highways
State_IGR_Transit_Sub State_IGR_Hous_Com_Dev State_IGR_Public_Welf
```

State_IGR_Sewerage State_IGR_Other Tot_Local_IG_Rev Local_IGR_InterSchool_Aid
Local_IGR_Other_Education Local_IGR_Oth_Gen_Sup Local_IGR_Health___Hos
Local_IGR_Highways
Local_IGR_Transit_Sub Local_IGR_Hous_Com_Dev Local_IGR_Public_Welf
Local_IGR_Sewerage Local_IGR_Other Tot_Chgs_and_Misc_Rev
Total_General_Charges Chg_Air_Transportation Chg_Misc_Com_Activ
Chg_Total_Education Chg_Total_Elem_Education Chg_Elem_Ed_Sch_Lunch
Chg_Elem_Ed_Tuition Chg_Elem_Ed_NEC Chg_Total_High_Ed
Chg_Hospitals Chg_Highways Chg_Regular_Highways Chg_Toll_Highways
Chg_Housing___Comm_Dev Chg_Total_Nat_Res Chg_Parking
Chg_Parks___Recreation Chg_Sewerage Chg_Solid_Waste_Mgmt
Chg_Water_Transport Chg_All_Other_NEC Misc_General_Revenue
Special_Assessments Prop_Sale_Total Prop_Sale_Hous_Com_Dev
Prop_Sale_Other Interest_Revenue Fines_and_Forfeits
Rents_and_Royalties Net_Lottery_Revenue Misc_General_Rev_NEC
Liquor_Stores_Revenue Total_Utility_Revenue Water_Utility_Revenue
Electric_Utility_Rev Gas_Utility_Rev Transit_Utility_Rev
Total_Insur_Trust_Rev Total_Insur_Trust_Ctrb Tot_Ins_Trust_Inv_Rev
Total_Emp_Ret_Rev
Emp_Ret_Total_Ctrib Emp_Ret_Loc_Emp_Ctrib Emp_Ret_Loc_To_Loc_Sys
Emp_Ret_From_Other_Gov Emp_Ret_Sta_To_Sta_Ctr Emp_Ret_Int_Rev
Emp_Ret_Other_Earnings Total_Unemp_Rev Unemp_Payroll_Tax
Unemp_Int_Revenue Unemp_Federal_Advances Total_Expenditure
Total_IG_Expenditure Direct_Expenditure Total_Current_Expend
Total_Current_Oper Total_Capital_Outlays Total_Construction
Total_Other_Capital_Outlays Tot_Assist___Subsidies Total_Interest_on_Debt
Total_Insur_Trust_Ben Total_Salaries___Wages
General_Expenditure IG_Exp_To_State_Govt IG_Exp_To_Local_Govts
IG_Exp_To_Federal_Govt Direct_General_Expend General_Current_Expend
General_Current_Oper General_Capital_Outlay General_Construction
General_Capital_Outlay_Other General_Assist___Sub General_Debt_Interest
Air_Trans_Total_Expend Air_Trans_Direct_Expend Air_Trans_Cap_Outlay
Air_Trans_Current_Exp Air_Trans_Construction Air_Trans_IG_To_State
Air_Trans_IG_Local_Govts Misc_Com_Activ_Tot_Exp
Misc_Com_Activ_Cap_Out Misc_Com_Activ_Current_Exp
Misc_Com_Activ_Constr Correct_Total_Exp Correct_Direct_Exp
Correct_Cap_Outlay Correct_Current_Exp Correct_Construct
Correct_IG_To_St Correct_IG_Loc_Govts Total_Educ_Total_Exp
Total_Educ_Direct_Exp Total_Educ_Assist___Sub Total_Educ_Cap_Outlay
Total_Educ_Current_Exp Total_Educ_Construct Elem_Educ_Total_Exp
Elem_Educ_Direct_Exp Elem_Educ_Cap_Outlay Elem_Educ_Current_Exp
Elem_Educ_Construction Elem_Educ_IG_To_State
Elem_Educ_IG_Local_Govts Elem_Educ_IG_Sch_to_Sch
Higher_Ed_Total_Exp Higher_Ed_Direct_Exp Higher_Ed_Cap_Outlay
Higher_Ed_Current_Exp Higher_Ed_Construct Higher_Ed_IG_To_St
Higher_Ed_IG_Loc_Govts Educ_NEC_Total_Expend Educ_NEC_Direct_Expend
Educ_NEC_Assistance Educ_NEC_Cap_Outlay Educ_NEC_Current_Exp
Educ_NEC_Construction Educ_NEC_IG_To_State Educ_NEC_IG_Local_Govts
Emp_Sec_Adm_Direct_Exp Emp_Sec_Adm_Cap_Outlay Emp_Sec_Adm_Current_Exp
Emp_Sec_Adm_Construct Fin_Admin_Total_Exp Fin_Admin_Direct_Exp
Fin_Admin_Cap_Outlay Fin_Admin_Current_Exp Fin_Admin_Construction
Fin_Admin_IG_To_State Fin_Admin_IG_Local_Govts
Fire_Prot_Total_Expend Fire_Prot_Direct_Exp
Fire_Prot_Cap_Outlay Fire_Prot_Current_Exp Fire_Prot_Construction
Fire_Prot_IG_To_State Fire_Prot_IG_Local_Govts
Judicial_Total_Expend Judicial_Direct_Expend Judicial_Cap_Outlay
Judicial_Current_Exp Judicial_Construction Judicial_IG_To_State

```
      Judicial_IG_Local_Govts Cen_Staff_Total_Expend  Cen_Staff_Direct_Exp
      Cen_Staff_Cap_Outlay Cen_Staff_Current_Exp Cen_Staff_Construction
      Cen_Staff_IG_To_State   Cen_Staff_IG_Local_Govts
      Gen_Pub_Bldg_Total_Exp  Gen_Pub_Bldg_Cap_Out Gen_Pub_Bldg_Current_Exp
Gen_Pub_Bldg_Construct
Health_Total_Expend     Health_Direct_Expend    Health_Capital_Outlay
Health_Current_Exp Health_Construction    Health_IG_To_State
      Health_IG_Local_Govts   Total_Hospital_Total_Exp
      Total_Hospital_Dir_Exp  Total_Hospital_Cap_Out
Total_Hospital_Current_Exp Total_Hospital_Construct
      Total_Hospital_IG_To_State    Total_Hospital_IG_Loc_Govts
      Own_Hospital_Total_Exp  Own_Hospital_Cap_Out Own_Hospital_Current_Exp
Own_Hospital_Construct  Hosp_Other_Total_Exp    Hosp_Other_Direct_Exp
      Hosp_Other_Cap_Outlay Hosp_Other_Current_Exp Hosp_Other_Construct
      Hosp_Other_IG_To_State
Hosp_Other_IG_Loc_Govts Total_Highways_Tot_Exp  Total_Highways_Dir_Exp
      Total_Highways_Cap_Out Total_Highways_Current_Exp
Total_Highways_Construct      Regular_Hwy_Total_Exp   Regular_Hwy_Direct_Exp
      Regular_Hwy_Cap_Outlay Regular_Hwy_Current_Exp Regular_Hwy_Construct
      Regular_Hwy_IG_To_Sta   Regular_Hwy_IG_Loc_Govts
      Toll_Hwy_Total_Expend   Toll_Hwy_Cap_Outlay Toll_Hwy_Current_Exp
Toll_Hwy_Construction   Transit_Sub_Total_Exp   Transit_Sub_Direct_Sub
      Transit_Sub_IG_To_Sta   Transit_Sub_IG_Loc_Govts
      Transit_Sub_To_Own_Sys  Hous___Com_Total_Exp
Hous___Com_Direct_Exp   Hous___Com_Cap_Outlay Hous___Com_Current_Exp
Hous___Com_Construct    Hous___Com_IG_To_State  Hous___Com_IG_Loc_Govts
      Libraries_Total_Expend  Libraries_Direct_Exp    Libraries_Cap_Outlay
Libraries_Current_Exp Libraries_Construction    Libraries_IG_To_State
      Libraries_IG_Local_Govts      Natural_Res_Total_Exp
      Natural_Res_Direct_Exp  Natural_Res_Cap_Outlay Natural_Res_Current_Exp
Natural_Res_Construct   Natural_Res_IG_To_Sta   Natural_Res_IG_Loc_Govts
      Parking_Total_Expend    Parking_Direct_Expend   Parking_Capital_Outlay
Parking_Current_Exp Parking_Construction  Parking_IG_To_State
      Parking_IG_Local_Govts  Parks___Rec_Total_Exp   Parks___Rec_Direct_Exp
      Parks___Rec_Cap_Outlay Parks___Rec_Current_Exp Parks___Rec_Construct
      Parks___Rec_IG_To_Sta   Parks___Rec_IG_Loc_Govts
      Police_Prot_Total_Exp   Police_Prot_Direct_Exp  Police_Prot_Cap_Outlay
Police_Prot_Current_Exp Police_Prot_Construct   Police_Prot_IG_To_Sta
      Police_Prot_IG_Loc_Govts      Prot_Insp_Total_Exp
      Prot_Insp_Direct_Exp    Prot_Insp_Cap_Outlay Prot_Insp_Current_Exp
Prot_Insp_Construction  Prot_Insp_IG_To_State
Prot_Insp_IG_Local_Govts      Public_Welf_Total_Exp   Public_Welf_Direct_Exp
      Public_Welf_Cash_Asst   Public_Welf_Cap_Outlay Public_Welf_Current_Exp
Public_Welf_Construct   Welf_Categ_Total_Exp    Welf_Categ_Cash_Assist
      Welf_Categ_IG_To_State  Welf_Categ_IG_Loc_Govts Welf_Cash_Total_Exp
      Welf_Cash_Cash_Assist   Welf_Cash_IG_Local_Govts
      Welf_Vend_Pmts_Medical  Welf_Vend_Pmts_NEC      Welf_State_Share_Part_D
      Welf_Ins_Total_Exp      Welf_Ins_Cap_Outlay Welf_Ins_Current_Exp
Welf_Ins_Construction   Welf_NEC_Total_Expend
Welf_NEC_Direct_Expend  Welf_NEC_Cap_Outlay Welf_NEC_Current_Exp
Welf_NEC_Construction   Welf_NEC_IG_To_State    Welf_NEC_IG_Local_Govts
      Sewerage_Total_Expend   Sewerage_Direct_Expend  Sewerage_Cap_Outlay
Sewerage_Current_Exp Sewerage_Construction      Sewerage_IG_To_State
      Sewerage_IG_Local_Govts SW_Mgmt_Total_Expend    SW_Mgmt_Direct_Expend
      SW_Mgmt_Capital_Outlay SW_Mgmt_Current_Exp SW_Mgmt_Construction
      SW_Mgmt_IG_To_State     SW_Mgmt_IG_Local_Govts  Water_Trans_Total_Exp
```

Water_Trans_Direct_Exp Water_Trans_Cap_Outlay Water_Trans_Current_Exp
Water_Trans_Construct
Water_Trans_IG_To_Sta   Water_Trans_IG_Loc_Govts   Interest_on_Gen_Debt
General_NEC_Total_Exp   General_NEC_Direct_Exp VetBonus
General_NEC_Cap_Outlay General_NEC_Current_Exp General_NEC_Construct
General_NEC_IG_To_St   General_NEC_IG_Loc_Govts
General_NEC_IG_To_Fed   Liquor_Stores_Tot_Exp   Liquor_Stores_Cap_Out
Liquor_Stores_Current_Exp Liquor_Stores_Constr Total_Util_Total_Exp
Total_Util_Inter_Exp   Total_Util_Cap_Outlay Total_Util_Current_Exp
Total_Util_Construct   Water_Util_Total_Exp   Water_Util_Inter_Exp
Water_Util_Cap_Outlay Water_Util_Current_Exp Water_Util_Construct
Elec_Util_Total_Exp   Elec_Util_Inter_Exp   Elec_Util_Cap_Outlay
Elec_Util_Current_Exp Elec_Util_Construct Gas_Util_Total_Exp
Gas_Util_Inter_Exp   Gas_Util_Cap_Outlay Gas_Util_Current_Exp
Gas_Util_Construct   Trans_Util_Total_Exp   Trans_Util_Inter_Exp
Trans_Util_Cap_Outlay Trans_Util_Current_Exp Trans_Util_Construct
Emp_Ret_Total_Expend   Emp_Ret_Benefit_Paymts Emp_Ret_Withdrawals
Emp_Ret_Other_Paymts   Unemp_Comp_Total_Exp   Unemp_Comp_Ben_Paymts
Unemp_Ext___Spec_Pmts
Total_Debt_Outstanding Total_Long_Term_Debt_Out   ST_Debt_End_of_Year
Total_Beg_LTD_Out Beg_LTD_Out_Private_Purp   Beg_LTD_Out_All_Other
Beg_LTD_Out_Utility   Beg_LTD_Out_Water_Util Beg_LTD_Out_Elec_Util
Beg_LTD_Out_Gas_Util   Beg_LTD_Out_Trans_Util Beg_LTD_Out_General
Beg_LTD_Out_Education   Beg_LTD_Out_Priv_Purp   Beg_LTD_Out_Other_NEC
Total_LTD_Issued LTD_Iss_Private_Purp   LTD_Iss_All_Other
LTD_Iss_Utility   LTD_Iss_Util_Water   LTD_Iss_Util_Electric
LTD_Iss_Util_Gas_Supply LTD_Iss_Util_Transit   LTD_Iss_General
LTD_Iss_Gen_Elem_Educ   LTD_Iss_Gen_Other_Educ LTD_Iss_Gen_Other_NEC
Total_LTD_Iss_FFC LTD_Iss_FFC_Utility   LTD_Iss_FFC_Water_Util
LTD_Iss_FFC_Elec_Util   LTD_Iss_FFC_Gas_Util   LTD_Iss_FFC_Trans_Util
LTD_Iss_FFC_General   LTD_Iss_FFC_Elem_Educ   LTD_Iss_FFC_Other_Educ
LTD_Iss_FFC_Other_NEC   Total_LTD_Iss_NG LTD_Iss_NG_Utility
LTD_Iss_NG_Water_Util   LTD_Iss_NG_Elec_Util   LTD_Iss_NG_Gas_Util
LTD_Iss_NG_Trans_Util   LTD_Iss_NG_General   LTD_Iss_NG_Elem_Educ
LTD_Iss_NG_Other_Educ   LTD_Iss_NG_Private_Purp LTD_Iss_NG_Other_NEC
Total_LTD_Iss_Unsp   LTD_Iss_Unsp_Utility   LTD_Iss_Unsp_Water_Util
LTD_Iss_Unsp_Elec_Util LTD_Iss_Unsp_Gas_Util   LTD_Iss_Unsp_Trans_Util
LTD_Iss_Unsp_General   LTD_Iss_Unsp_Elem_Educ LTD_Iss_Unsp_Other_Educ
LTD_Iss_Unsp_Other_NEC Total_LTD_Retired LTD_Ret_Private_Purp
LTD_Ret_All_Other LTD_Ret_Utility   LTD_Ret_Util_Water
LTD_Ret_Util_Electric   LTD_Ret_Util_Gas_Supply LTD_Ret_Util_Transit
LTD_Ret_General   LTD_Ret_Gen_Elem_Educ   LTD_Ret_Gen_Other_Educ
LTD_Ret_Gen_Other_NEC   Total_LTD_Ret_FFC LTD_Ret_FFC_Utility
LTD_Ret_FFC_Water_Util LTD_Ret_FFC_Elec_Util   LTD_Ret_FFC_Gas_Util
LTD_Ret_FFC_Trans_Util LTD_Ret_FFC_General   LTD_Ret_FFC_Elem_Educ
LTD_Ret_FFC_Other_Educ LTD_Ret_FFC_Other_NEC   Total_LTD_Ret_NG
LTD_Ret_NG_Utility   LTD_Ret_NG_Water_Util   LTD_Ret_NG_Elec_Util
LTD_Ret_NG_Gas_Util   LTD_Ret_NG_Trans_Util   LTD_Ret_NG_General
LTD_Ret_NG_Elem_Educ   LTD_Ret_NG_Other_Educ   LTD_Ret_NG_Private_Purp
LTD_Ret_NG_Other_NEC   Total_LTD_Ret_Unsp   LTD_Ret_Unsp_Utility
LTD_Ret_Unsp_Water_Util LTD_Ret_Unsp_Elec_Utili LTD_Ret_Unsp_Gas_Util
LTD_Ret_Unsp_Trans_Util LTD_Ret_Unsp_General   LTD_Ret_Unsp_Elem_Educ
LTD_Ret_Unsp_Other_Educ LTD_Ret_Unsp_Other_NEC Total_LTD_Out
LTD_Out_Private_Purp   LTD_Out_All_Other Total_LTD_Out_Utility
LTD_Out_Util_Water   LTD_Out_Util_Electric   LTD_Out_Util_Gas_Supply
LTD_Out_Util_Transit   LTD_Out_General   LTD_Out_Gen_Elem_Educ
LTD_Out_Gen_Other_Educ LTD_Out_Gen_Other_NEC   Total_LTD_Out_FFC

```
      LTD_Out_FFC_Utility     LTD_Out_FFC_Water_Util  LTD_Out_FFC_Elec_Util
      LTD_Out_FFC_Gas_Util    LTD_Out_FFC_Trans_Util  LTD_Out_FFC_General
      LTD_Out_FFC_Elem_Educ   LTD_Out_FFC_Other_Educ  LTD_Out_FFC_Other_NEC
      Tot_LTD_Out_NG    LTD_Out_NG_Utility      LTD_Out_NG_Water_Util
LTD_Out_NG_Elec_Util    LTD_Out_NG_Gas_Util     LTD_Out_NG_Trans_Util
      LTD_Out_NG_General      LTD_Out_NG_Elem_Educ    LTD_Out_NG_Other_Educ
      LTD_Out_NG_Private_Purp LTD_Out_NG_Other_NEC    Total_Cash___Securities
      Insur_Trust_Cash___Sec  Emp_Retire_Cash___Sec   Emp_Retire_Cash___Dep
      Emp_Retire_Total_Sec    Emp_Retire_Sec_Tot_Fed
      Emp_Retire_Sec_S_L_Secur      Emp_Retire_Sec_Tot_Nong
      Emp_Retire_Sec_Corp_Bds Emp_Retire_Sec_Corp_Stk
      Emp_Retire_Sec_Mortgages
Emp_Retire_Sec_Misc_Inv Emp_Retire_Sec_Oth_Nong Unemp_Comp_Cash___Sec
      Unemp_Comp_Bal_In_US_Trs      Unemp_Comp_Other_Balance
      Nonin_Trust_Cash___Sec  Sinking_Fd_Cash___Sec   Bond_Fd_Cash___Sec
      Oth_Nonin_Fd_Cash___Sec FunctionCode Enrollment;
      Set LargestData;
run;


* Write the final file to a delinimated text file, change this path to define
a valid location for the file on your system;
Proc Export Data=work.LargestData
      outfile='E:/CensusData/AllCensusData.csv'
      dbms=CSV
      replace;
      run;
```
