# Supplementary material for: The Government Finance Database: A Common Resource for Quantitative Research in Public Financial Analysis
Source: PLoS One. 2015 Jun 24;10(6):e0130119. doi: 10.1371/journal.pone.0130119 (PMC4479543; doi:10.1371/journal.pone.0130119)
Supplement: S2 File — This file includes the state data from the Government Finance Database at the time of publication, the appendix for the Government Finance Database, and the U.S. Census 2006 Classification manual. For more up to date versions of the data please visit http://www.willamette.edu/mba/research_impact/public_datasets/. (ZIP) [file pone.0130119.s002.zip › PLoS ONE The Government Finance Database.pdf]

The Government Finance Database: A Common Resource for Quantitative Research in Public Financial Analysis

Kawika Pierson[1*], Michael L. Hand[1], Fred Thompson[1]

[1] Atkinson Graduate School of Management, Center for Governance and Public Policy Research, Willamette University, Salem, Oregon, United States of America

* Corresponding Author
Email: kpierson@willamette.edu (KP)

Citation:

Pierson K., Hand M., and Thompson F. (2015). The Government Finance Database: A Common Resource for Quantitative Research in Public Financial Analysis. PLoS ONE doi: 10.1371/journal.pone.0130119

Abstract

Quantitative public financial management research focused on local governments is limited by the absence of a common database for empirical analysis. While the U.S. Census Bureau distributes government finance data that some scholars have utilized, the arduous process of collecting, interpreting, and organizing the data has led its adoption to be prohibitive and inconsistent. In this article we offer a single, coherent resource that contains all of the government financial data from 1967-2012, uses easy to understand natural-language variable names, and will be extended when new data is available.

Introduction

Widely shared and easy to use databases facilitate quantitative research and render the replication of findings practical and convenient [1]. Indeed, much of what we know about public finance has been tested against large microdata sets – in the United States, primarily merged information files based on household-level data from the IRS Individual Public-Use Tax Files, the Current Population Survey, the Consumer Expenditure Survey, and the triennial Survey of Consumer Finances. Unfortunately, students of public financial management at the local government level must often rely on one-off, custom-built datasets to pursue their inquiries, which is costly, inimical to replication, and leaves practitioners uncertain about the utility of academic insights.

For someone from outside the field of public financial management the lack of widely used and consistently applied data might seem an unlikely obstacle. After all, scholars of public financial management have access to a database that is in many respects ideally suited to their needs. The U.S. Census Bureau has surveyed state and local governments annually since 1967, and, as the Director of the U.S. Census Bureau stated in a letter accompanying the 2013 request for financial information: "This survey is the only comprehensive source of information on the finances of local governments in the United States."

Many examples of research using these data exist, including recent papers by Gore [2], Baber and Gore [3], Kido et al. [4], Murray et al. [5], Carroll [6], Mullins [7], and Fisher and Papke [8], among others. However because the government financial data retrieved from the census require substantial effort to obtain, interpret, translate, consolidate, and use, every example of its scholarly application is unique in the years included for analysis, variables consolidated or ignored, and types of governments considered.

The diversity of treatments and time horizons in work using the Census of Governments data isn't surprising given the investment of time and resources necessary to work with the data, but it is potentially damaging to the interpretation and application of research in our field. By consolidating the Census

Bureau's government financial data into a single, coherent database we hope to alleviate these concerns and move quantitative research in public finance progressively forward.

Arguably, the situation is similar to the situation in corporate finance prior to the availability of the CRSP-COMPUSTAT database of stock prices and accounting data. Accounting data were available from the Securities and Exchange Commission and data on share prices could be obtained from various vendors, but merging and matching observations from these files was prohibitively costly. Consequently, the data were rarely used and, when they were, it was nearly impossible to explain, let alone resolve, the numerous discrepancies in the findings that resulted, which held back sustained intellectual progress in the field. Corporate financial research no longer suffers from this problem. The CRSP-COMPUSTAT database has secured the field's sustained progress.

This article describes the steps we have taken to make the Census Bureau's annual surveys of state and local government finances equally easy to interpret and use. It offers a single, comprehensive database of government finance statistics, which includes detailed financial data from states, municipalities, townships, special districts, and school districts for the years 1967 through 2012, processed to make it user friendly – uncomplicated to use and convenient for replication. The database is freely available and can be downloaded from:

http://www.willamette.edu/mba/research_impact/public_datasets/

We will demonstrate some applications of the database here, but its potential for scholarly inquiry is staggering. The data include extensive information on government revenue from both tax and non-tax sources, facilitating a more general understanding of strategies to increase revenue streams [9], the interdependencies of local government and school district revenue [10], or the budgetary impacts of revenue diversity [11], just to name a few possibilities.

The data include detailed breakdowns of expenditures by both type and function, which can propel answers to questions about spending on education and transportation [12], the importance of the business cycle for budgets [13], geographic impacts on categories of municipal spending [14], or the applicability

of aggregate budget functions [15]. The database also contains information about the cash positions of governments, the issuance and retirement of debt, and the investments of social insurance trusts.

Some caveats are appropriate however. The government finance database is not a perfect resource. In particular the data do not include measures of accomplishment or effort, except where money spent is a reasonable proxy, and so the database must be supplemented if such measures are important to the question being studied, e.g. by merging it with performance data, such as the Texas school-district performance data [16]. However, given the push towards both methodological [17] and theoretical [18] innovation in public administration research, and given the existing diversity the field displays in those areas (see for instance [19] [20]) this breadth of financial information provided from a single, standardized source has the potential to facilitate a diverse body of inquiry.

After explaining the overall structure of the data, what variables are included, and how the data is transformed from its raw state, we will transition to discussing several insights that arose from our initial analysis of the data. These include examples of using the data to better understand patterns in government finance as well as important advice for other researchers working with the database.

Materials and Methods

Issues with Data Availability and Coding

The basic unit of reporting in government accounting is the fund, essentially a separate bucket of financial resources tasked with accomplishing some objective [21]. While more recent accounting guidance mandates that some government-wide information be reported in addition to fund level reports [22], the census extends this reference frame by consolidating information across funds and presenting all of its data on a government-wide basis. This approach is broadly beneficial for studies that seek to understand something about governments as separate financial entities, and better conforms to the way that citizens and financial intermediaries (as opposed to governmental managers charged with oversight) use government accounting data [23].

While many government funds report information on a modified-accrual basis, some are required to report using a cash basis, a modified cash basis, or (rarely) a full accrual basis [24]. This diversity of reporting practices within and across governments presents some difficulty to anyone attempting to present or utilize government financial data in a consistent manner. Given that a transformation of data between the different accounting treatments is not possible, the census adopts the accounting basis declared by each government fund "so long as that basis (1) conforms to generally accepted accounting procedures and (2) is applied consistently from year-to-year." In practice this means that the data are best conceptualized as roughly equivalent to cash flows, even though they will not always represent actual cash flows during the periods reported.

The data are reported in *thousands* of nominal dollars, unadjusted for changes in prices or wages over time, allowing researchers to choose whether and how best to convert the information into real dollars. The time period represented in the data is 1967-2012, however the number of governments included varies significantly from year to year. The primary source of this variation is the fact that the Census Bureau collects financial data from governments in two separate, but related efforts. During years ending in a 2 or 7 the government collects a census (essentially a population) of government financial statistics in the "Census of Government Finance and Employment Data". Every year when a census is not being conducted a sample of governments report data through the "Annual Survey of State & Local Government Finances".

The data include federal (type 6), state (type 0), county (type 1), municipal (type 2), township (type 3), special district (type 4), and school district data (type 5), each of which can be isolated by censoring the data on the "Type_Code" variable. While every state is included in the sample every year the coverage for other government types is less complete. Fig 1 shows the number of governments of each type that are included in the data each year.

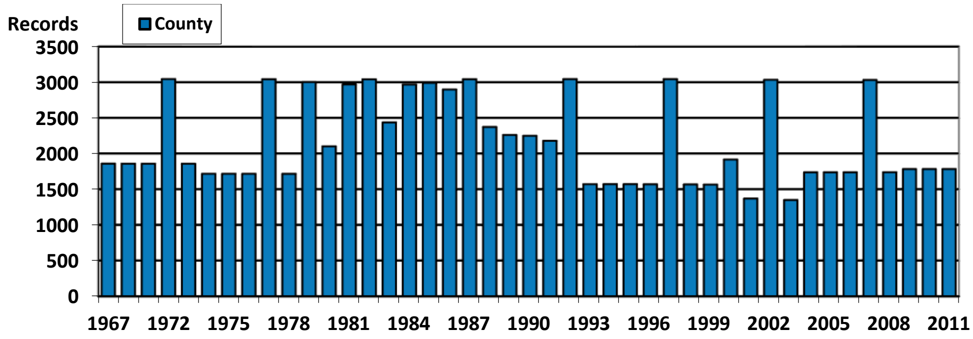
Records
County
3500
3000
2500
2000
1500
1000
500
0
1967 1972 1975 1978 1981 1984 1987 1990 1993 1996 1999 2002 2005 2008 2011

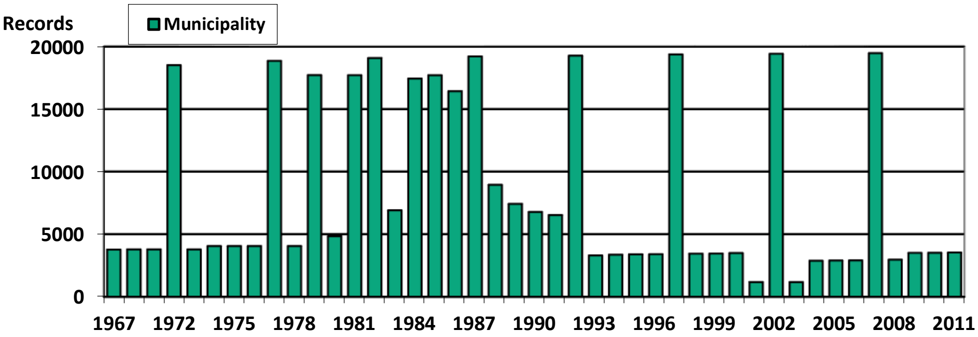
Records
Municipality
20000
15000
10000
5000
0
1967 1972 1975 1978 1981 1984 1987 1990 1993 1996 1999 2002 2005 2008 2011

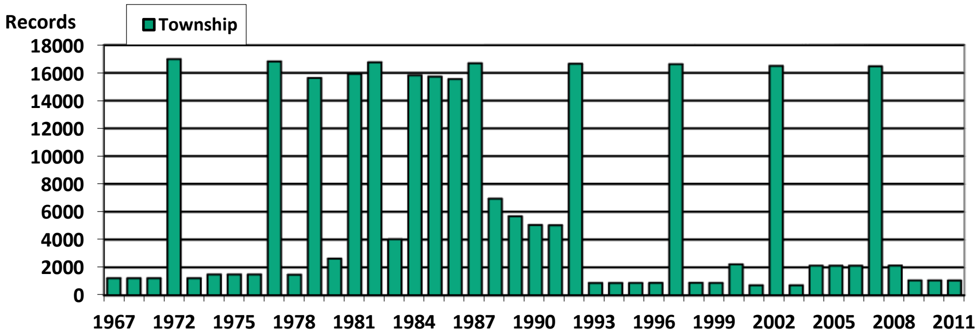
Records
Township
18000
16000
14000
12000
10000
8000
6000
4000
2000
0
1967 1972 1975 1978 1981 1984 1987 1990 1993 1996 1999 2002 2005 2008 2011

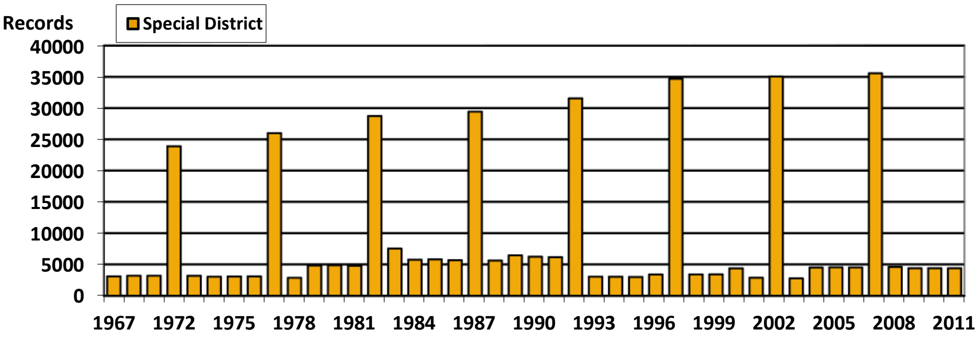
Records
Special District
40000
35000
30000
25000
20000
15000
10000
5000
0
1967 1972 1975 1978 1981 1984 1987 1990 1993 1996 1999 2002 2005 2008 2011

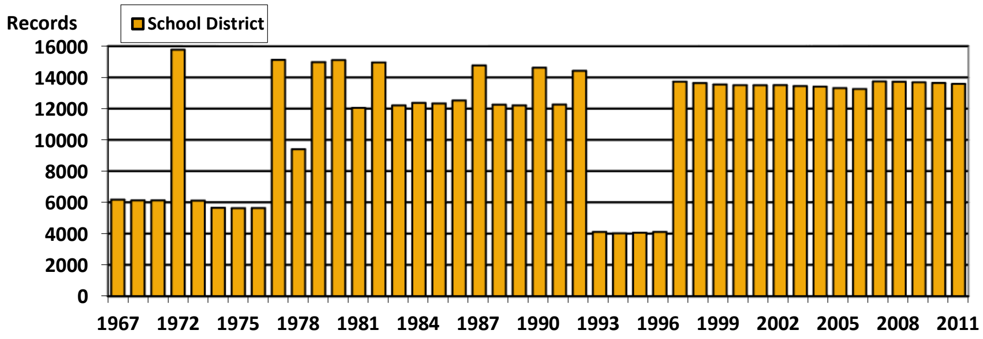

Fig 1. Report Counts by Government Type.
This figure shows five bar graphs. One for each of counties, municipalities, townships, special districts, and school districts showing the number of records for each government type that exist in each year of the data.

Fig 1 highlights several important insights into the coverage of the data over time. Reporting rates are uniformly high during years when a full census was conducted. In addition, school districts report at much higher rates than other governments, but show a large reduction in reporting during the years between 1993 and 1996. Closer examination of the data for other government types shows a similar (but less visually pronounced) reduction in coverage during those years.

In 1993 the census began sampling a smaller, but still significant, portion of all government types. Because of their work with the National Center for Education Statistics, the census was able to resume nearly complete coverage of school districts following the 1997 census, but the other government types were never again sampled at the levels seen in the late 1980's.

One of the largest hurdles in the process of organizing the government financial data as a single, coherent database is learning to interpret the codes used by the census to identify what each data point represents. The database we present replaces these codes with natural language variable names borrowed from the census' classification manual, however understanding the codes that the census uses internally will help readers to validate, interpret, and apply our work.

Each census code combines an "object code" with a "function code". Object codes are one character long and represent large categories or types of data. For instance, the object code T is used for

all tax revenues. Function codes are double digit numbers that indicate what the funds in question were used for. Combining an object code, such as A, for current charges, with a function code, such as 12, for elementary and secondary education, results in a pointer to a particular variable, in this case A12: current charges from elementary and secondary education. Function codes are not applied consistently across the entire data set, but are still useful to understanding data within large sections of it. For instance, the function code 01 represents property taxes whenever it is used with object code T, but represents air transportation with every expenditure function code.

Creating a Single, Coherent Database

The government financial data comes in two forms. Data from 1967 through 2007 is more or less organized in the manner that researchers expect from panel data. The files are divided by year. Each row of each file corresponds to one government. There are several columns for identifying information and a column for each financial variable. These columns are all labeled with natural language names that make it easy to understand what they represent. One wrinkle arises from the fact that data for this period is always provided in three separate text files each year. Each file contains a row for every government and some identifying information, but the three files contain different subsets of the financial information available.

Overcoming this challenge is straightforward. Given the consistent naming scheme used by the census for these years we simply merge the three data files each year so that all of the columns are available in one large matrix. We then loop through the years available and continue aggregating the data into what we call the “early database”.

The newer data presents a much more substantial challenge, and the process of consolidating it with the early database to create one source of data is our main contribution. Data after 2007 is organized into two files per year. The first file is a fixed width text file called the “Individual Unit File”. On each row of this file there is one government ID number, one census data code, one number representing data, the year of the data, and a character that encodes something about how the data was gathered.

This organization presents the first major hurdle to merging the recent data with the early database, since each row of the individual unit file holds data that must comprise one cell in the final matrix. For this reason the individual unit file is transposed so that there is one row per government and one column for each census data code.

The second file the Census provides contains identifying information for every government in that year's data, and is organized by government ID code. This "Government ID" file has the name of each government, population figures, and several other pieces of identifying information. Once the individual unit files are transposed they are merged with this identifying information to create the "recent database".

The second major hurdle presented by the more recent government financial data is the fact that the data are not encoded with natural language variable names the way that the early data is. This needs to be fixed, and so the final step in our data consolidation process is a mapping of each of the census codes onto the variable names used in the early database. The Census provides some resources to facilitate the process, including a user's guide to the early data and classification manuals describing the recent data, but the process is still time consuming and meticulous in a way that likely deters other researchers from incorporating the recent data into their studies.

In the end we take the recoded recent data and merge it with the early data to present a single coherent database of government financial data between 1967 and 2012. Specific instructions for replicating our consolidation, the SAS code we employed, and a mapping of data codes to variable names is available in our supplementary files (S1 File) and are also included with the database when you download it from our website (http://www.willamette.edu/mba/research_impact/public_datasets/). A high-level view of the process of organizing and consolidating all of the government financial data is shown in Fig 2.

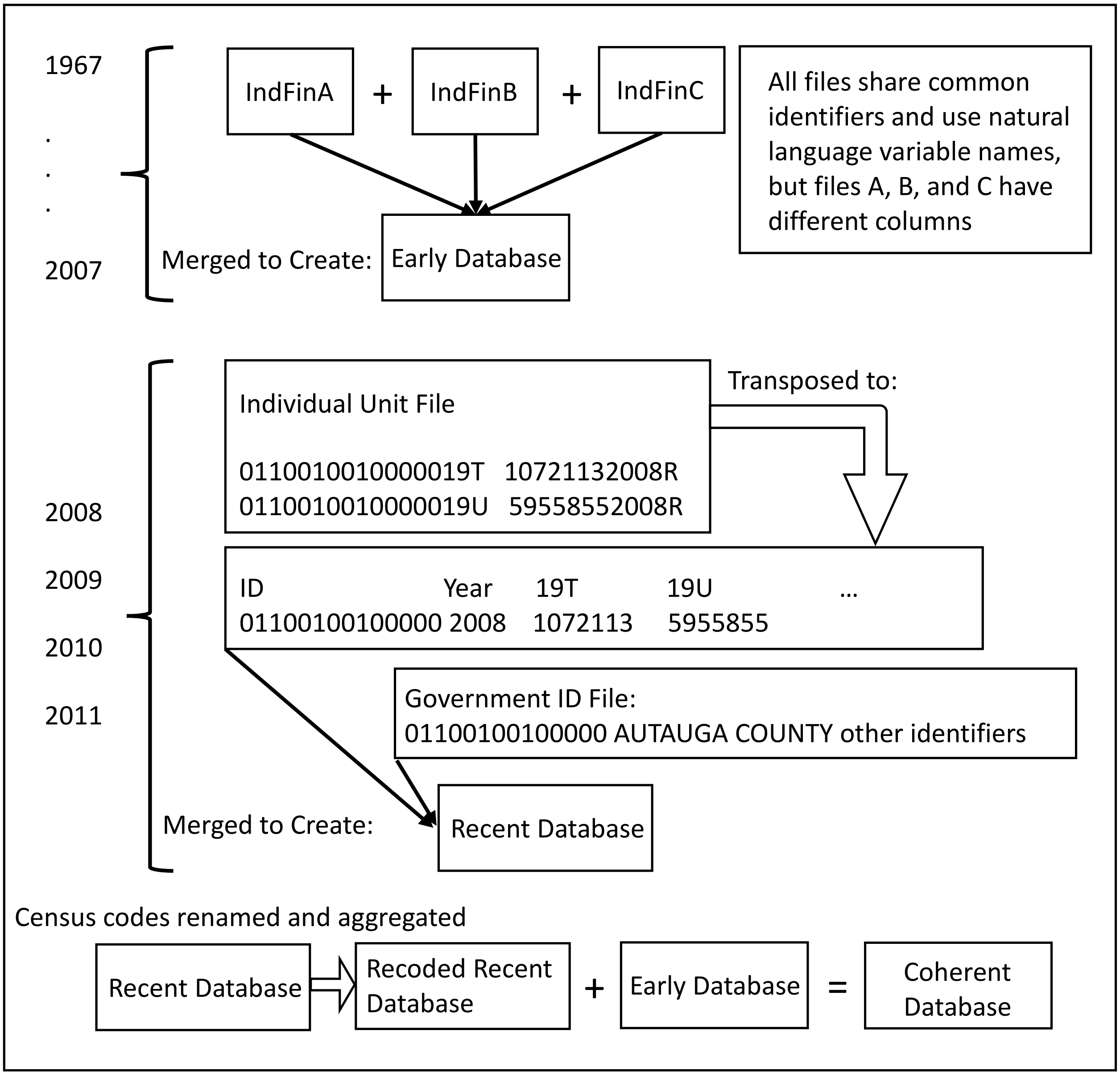

Fig 2. Consolidation of the Census of Governments Data.
A conceptual overview of the process of consolidating the various data files to form a single coherent database.

Categories of Financial Data

At a high level the data for each government are grouped into four large categories: revenue flows, expenditure flows, cash and investment positions, and debt positions.

Revenue

The revenue data are organized by sector into general revenue, utility revenue, liquor store revenue, and social insurance trust revenue. Each of these sectors is comprised of a number of smaller subcategories, as shown in Table 1.

Table 1. Revenue Categories.

Revenue Categories	Census Object	Description
General Revenue	T B C D A U	All revenue not arising from utilities, liquor stores, or social insurance
Taxes	T	All taxes other than those assessed for social insurance
Intergovernmental Revenue	B C D	Transfers to the government from others, including grants and shared taxes
From Federal	B	Intergovernmental revenue from federal sources
From State	C	Intergovernmental revenue from state sources
From Local	D	Intergovernmental revenue from local sources
Current Charges	A	Fees collected for providing services, other than utility service charges or liquor store charges
Miscellaneous General Rev	U	Other general revenue from a government's own sources
Utility Revenue	A	Revenue from providing water, electric, gas, or transportation services
Liquor Store Revenue	A	Sales revenue from government run liquor stores
Social Insurance Trust Rev	X Y	Contributions and investment earnings (or losses) for all social insurance programs.
Retirement Plans	X	Contributions and investment earnings (or losses) for public employee retirement programs
Unemployment Revenue	Y	Contributions and investment earnings (or losses) for the unemployment compensation insurance system

This table shows the high-level organization of the different revenue variables in the database. It references the census object codes used to create these categories, and provides a short description of each. The indentation of the variables in the first column indicates how subcategories of data collapse into larger categories. More detailed descriptions of each category can be found in the Census' 2006 classification manual (http://www.census.gov/govs/classification/) included with the database download.

The revenue data within each subcategory are further broken down in order to identify more specific sources of funds. Tax revenues have the largest number of subcategories in the data. Table 2 summarizes the organization of tax revenue subcategories.

Table 2. Tax Revenue Categories.

Tax Revenue Categories	Census Code	Description
Total Taxes		The sum of all of the tax categories
Property Tax	T01	All taxes on property that use its value as a basis
Total Sales Taxes		The sum of general and selective sales taxes
General Sales Tax	T09	Taxes on the sale of all types of goods and services

Total Selective Sales Taxes		The sum of the eight selective sales tax categories
Alcoholic Beverage	T10	Sales taxes on government and private sales of alcohol
Amusement	T11	Sales taxes on all types of amusement businesses
Insurance Premium	T12	Sales taxes on insurance
Motor Fuel	T13	Sales taxes on fuels for vehicles and aircraft
Pari-mutuels	T14	Sales taxes on wagers and betting
Public Utilities	T15	Sales taxes on government owned utilities
Tobacco	T16	Sales taxes on tobacco products
Other Selective Sales Tax	T19	All other selective sales taxes
Total License Taxes		The sum of the nine licensing tax subcategories
Alcoholic Beverage	T20	Licenses pertaining to alcohol
Amusement	T21	Licenses pertaining to amusement businesses
Corporate	T22	Licenses pertaining to all corporations
Hunting and Fishing	T23	Licenses pertaining to hunting and fishing
Total Motor Vehicle		The sum of motor vehicle and operator licenses
Motor Vehicle	T24	Licenses pertaining to the right to operate a vehicle (Registration, plates, inspection ect.)
Operator Licenses	T25	Licenses pertaining to the right to drive
Public Utility	T27	Licenses imposed on public utilities
Occupation and business	T28	Licenses for certain professions and businesses
Other Licenses	T29	All other licenses.
Total income Taxes		The sum of individual and corporate income taxes.
Individual	T40	Taxes on the income of individuals
Corporate	T41	Taxes on the income of corporations
Death and Gift Tax	T50	Taxes on the transfer of property after death
Documentary Tax	T51	Taxes on the transfer of documents
Severance Tax	T53	Taxes on the removal of natural resources
Taxes NEC	T99	All other taxes not listed above

This table gives a detailed breakdown of the different tax revenue data reported. NEC stands for not elsewhere classified. The indentation of the variables in the first column indicates how subcategories of data collapse into larger categories. More detailed descriptions of each category can be found in the Census' 2006 classification manual (http://www.census.gov/govs/classification/) included with the database download.

Intergovernmental revenue data is first separated based on its source (from the federal, state, or local government) as shown in Table 1. Within each of these sources intergovernmental revenue is categorized by its intended use. Table 3 displays this structure.

Table 3. Intergovernmental Revenue Functions.

Intergovernmental Revenue Categories	Census Number	Description
Air Transportation	01	Aid in support of public airports
Interschool revenue	11	Aid from one school district to another (schools only)
Education	21	Aid for public schools

Employment Security	22	Transfers to the states from the federal government for unemployment insurance
General Support	30	Aid that can be applied for any purpose
Health and Hospitals	42	Aid intended for public health or hospitals
Highways	46	Aid to be used for roads, streets, and highways
Transit Subsidies	94	Aid for mass transit systems
Housing and Community Dev	50	Aid for public housing and other community development
Natural Resources	59	Federal aid for conservation resource protection
Public Welfare	79	Aid for social welfare programs
Sewerage	80	Aid for sewage systems, disposal and treatment
Other Uses	89	All other aid not classified above

This table describes a detailed breakdown of the different intergovernmental revenue function codes reported by governments in the data. More detailed descriptions of each function code can be found in the Census' 2006 classification manual (http://www.census.gov/govs/classification/) included with the database download.

The precise application of each of these categories changes somewhat based on the source of the intergovernmental revenue. For instance federally sourced intergovernmental revenue for public welfare includes programs such as TANF (Temporary Assistance for Needy Families) and Medicaid, whereas state sourced intergovernmental revenue for public welfare includes pass-through of these programs, as well as revenue arising from state specific programs. An exhaustive documentation of what each variable contains and excludes is available in the census classification manual included in the database download.

Current charges are amounts that the government collects from individuals and corporations in exchange for providing services. They are reported in gross amounts, ignoring any cost of service. Liquor stores and utilities are excluded from current charges and given their own category of revenue in order to distinguish them from general revenue. Charges are separated based on the type of service provided as shown in Table 4.

Table 4. Current Charge Functions.

Charge Functions	Census Number	Description
Total General Charges		The sum of all charges
Airport Charges	01	Charges relating to air transportation
Misc. Commercial Charges	03	Charges from all publicly owned enterprises NEC
Total Education Charges		The sum of the three education subcategories
Total Elem-Secondary		The sum of the next three variables
School Lunch	09	Revenue from the sale of milk and school lunches
Tuition	10	Charges for tuition and transportation
Other	12	Other charges (athletics, textbooks ect.)
Higher Education	16 18	All charges from public higher education
All Other Education	21	Charges from all other state or federally run schools

Hospital Charges	36	Charges for care in publicly run hospitals
Total Highway Charges		The sum of the next two variables
Regular Highways	44	Assessments and fees for the maintenance of non-toll roads
Toll Highways	45	Fees from toll roads
Housing and Com Dev	50	Revenue from the rental of public housing
Natural Resources	56 59	Charges from forestry and other natural resources
Parking Charges	60	Charges from on and off-street parking, and lots
Parks and Recreation	61	Revenue from facilities, parks, stadiums ect.
Sewerage	80	Charges for sewage connection, collection and disposal
Solid Waste Management	81	Fees from garbage collection and the operation of landfills
Water Transport	87	Charges relating to port terminals and canal operation
All Other General Charges	89	All charges NEC

This table describes a detailed breakdown of the different current charge function codes reported in the data. NEC stands for not elsewhere classified. The indentation of the variables in the first column indicates how subcategories of data collapse into larger categories. More detailed descriptions of each function code can be found in the Census' 2006 classification manual (http://www.census.gov/govs/classification/) included with the database download.

Liquor store and utility revenue are not disaggregated to the extent that the other revenue data is. Total liquor store revenue is reported, and utility revenue is broken into revenue from each of the four types of utilities: water, electricity, gas, and mass transit.

Several categories of general revenue are listed under miscellaneous general revenue. Their organization is shown in Table 5.

Table 5. Miscellaneous General Revenue Variables.

Variable Name	Census Code	Description
Total Charges and Misc. Revenue		The sum of total charges and total misc. revenue
Total Misc. General Revenue		The sum of the seven variables below
Special Assessments	U01	Charges to individuals benefiting from improvements
Property Sale Other	U11	Gross receipts from all property sales
Interest Revenue	U20	Interest earnings from all sources
Fines and Forfeits	U30	Revenue from legal penalties
Rents and Royalties	U40 U41	The sum of rent and royalty income
Net Lottery Revenue	U95	Lottery proceeds net of the cost of prizes
Misc. General Revenue NEC	U99	All general revenue NEC

This table describes the coding of the miscellaneous revenue variables and provides a short description of each. NEC stands for not elsewhere classified. The indentation of the variables in the first column indicates how subcategories of data collapse into larger categories. More detailed descriptions of each variable can be found in the Census' 2006 classification manual (http://www.census.gov/govs/classification/) included with the database download.

The last category of revenue is revenue from social insurance trusts. Insurance trust revenue is separated into retirement plan revenue and unemployment revenue, and several smaller partitions of both are reported as shown in table 6.

Table 6. Insurance Trust Revenue Variables.

Variable Name	Census Code	Description
Total Insurance Trust Revenue		The sum of all insurance trust revenue
Total Insurance Trust Contributions		The sum of the contribution variables below
Total Trust Investment Revenue		The sum of the investment variables below
Total Retirement Plan Revenue		The sum of all retirement plan revenue
Total Retirement Contributions		The sum of the following four contribution variables
Local Government Employees	X01	Contributions from employees of local governments
State Government Employees	X04	Contributions from employees of state governments
From Other Governments	X05	Contributions coming from other governments
Contribution to Own System	X06	Contributions to the government's own system
Investment Earnings	X08	All earnings on the investments of the retirement plan
Total Unemployment Revenue		The sum of the following three variables
Unemployment Payroll Tax	Y01	Included in total insurance trust contributions
Unemployment Interest Revenue	Y02	Included in total investment revenue
Unemployment Federal Advances	Y04	Funds received when taxes and investments cannot cover the benefits due to unemployed workers

This table describes the coding of the social insurance trust revenue variables and provides a short description of each. NEC stands for not elsewhere classified. The indentation of the variables in the first column indicates how subcategories of data collapse into larger categories. More detailed descriptions of each variable can be found in the Census' 2006 classification manual (http://www.census.gov/govs/classification/) included with the database download.

Expenditure

Expenditures are organized according to their category and function. The category of each expenditure refers to how the cash was used, while the function of the expenditure refers to the type of service it was used to accomplish. In general every expenditure variable is a combination of one category and one function, following the logic of the census codes. For instance, "Air Transportation Capital Outlay" is in the capital outlay category and was used for the air transportation function.

Table 7 shows the different categories of expenditures that are recorded in the data. Total expenditures are the sum of direct expenditures and intergovernmental expenditures. Direct expenditures can further be broken down into current expenditures used to pay employees, purchase supplies and hire contractors; construction expenditures used to build long term assets; and expenditures used to purchase (rather than build) long term assets. Capital outlay expenditures are the sum of construction and purchase expenditures.

Table 7. Expenditure Categories.

Expenditure Categories	Census Object	Description
Total	E F G L M	The sum of all expenditures
Direct	E F G	Current expenditures (such as salaries and supplies), plus any expenditures for capital improvements
Capital Outlay	F G	Purchase or construction of capital improvements
Construction	F	Construction expenditures only
Intergovernmental to State	L	Paid to state governments for performance of functions or aid related to those functions
Intergovernmental to Local	M	Paid to local governments for performance of functions or aid related to those functions

Every set of expenditure data follows a similar organization. This table shows how to interpret the names given to the variables in the database, references the census object codes used to create them, and provides a short description of each. The indentation of the variables in the first column indicates how subcategories of data collapse into larger categories. More detailed descriptions of each category can be found in the Census' 2006 classification manual (http://www.census.gov/govs/classification/) included with the database download.

Intergovernmental expenditures are defined by the census as "amounts paid to other governments for performance of specific functions or for general financial support." They are included in total expenditure, and are separated based on whether the funds went to state governments or local ones.

In a very small number of instances assistance, subsidies, and interest on debt are added to direct expenditures and total expenditures. Assistance and subsidies are coded by the census as object J, and occur four times in the data: state government scholarships (J19), federal categorical assistance programs (J67), other cash assistance programs (J68), and federal and state veterans' services (J85). Interest on debt is coded by the census as object I and occurs five times: interest on general debt (I89), and interest on debt for the four classes of utilities (I91, I92, I93, and I94). When this occurs the data always include a separate line item reporting the amount of assistance, subsidies, or interest, allowing researchers to correct for their inclusion in direct expenditure if necessary.

Expenditures are also separated by function within the database. Table 8 shows the various expenditure functions considered in the data and the census function codes that correspond to them. Some of the expenditure functions recorded by the census only exist at the federal level and have been excluded from the database otherwise. Other codes exist in the newest census data but do not exist for years prior to 2007 and have been consolidated into their earlier versions to create a more coherent database.

Table 8. Expenditure Function Codes.

Expenditure Functions	Census Number	Expenditure Functions Continued	Census Number
Air Transport	01	Parking Facilities	60
Miscellaneous Commercial Activities, NEC	03	Parks and Recreation	61
Correctional Institutions	04	Police Protection	62
Elementary and Secondary Education	12	Protective Inspection & Reg., NEC	66
Higher Education	16 18	Public Welfare – sum of several smaller functions	67 68 74 75 77 79
State Government Scholarships	19	Federal Categorical Assistance	67
Education NEC	21	Other Cash Assistance Programs	68
Employment Security Administration	22	Vendor Payments Medical Care	74
Financial Administration	23	Vendor Payments Other Purposes	75
Fire Protection	24	Institutions	77
Judicial and Legal	25	Public Welfare - Other	79
Central Staff Services	29	Sewerage	80
General Public Buildings	31	Solid Waste Management	81
Health	32	Sea and Inland Port Facilities	87
Hospitals	36	General Expenditure NEC	89
Federal Owned Hospitals - Veterans	37	Liquor Stores	90
Federal Other Hospitals - Veterans	39	Utilities Total – sum of several smaller functions	91 92 93 94
Regular (non-toll) Highways	44	Water Supply	91
Toll Highways	45	Electric Power	92
Housing and Community Development	50	Gas Supply	93
Libraries	52	Public Mass Transit Systems	94
Natural Resources	55 56 59		

The data include a number of different functional separations for expenditures and the table above shows the name of each along with the corresponding census function number or numbers included in that expenditure function. NEC stands for not elsewhere classified. The indentation of the variables in the first column indicates how subcategories of data collapse into larger categories. More detailed descriptions of each expenditure function can be found in the Census' 2006 classification manual (http://www.census.gov/govs/classification/) included with the database download.

Cash and Investment Positions

Several of the cash and investment positions of each government are recorded in the data. Other current and long term assets, such as those recorded on a typical statement of net position are not included by the census. A summary of these variables is shown in table 9.

Table 9. Cash and Investment Security Variables.

Variable Name	Census Code	Description
Total Cash and Securities		The sum of all cash and securities held
Insurance Trust Cash and Securities		The sum of retirement and unemployment investments

Employee Retirement Cash and Sec.		The sum of all employee retirement cash and security amounts
Employee Retirement Cash	X21	Cash held by the employee retirement system
Employee Retirement Securities		The sum of the following two subcategories
Federal Securities	X30	Amount invested in federal government securities
Non-Governmental Securities		The sum of the following five variables
Corporate Bonds	Z77	All forms of corporate debt
Corporate Stock	Z78	All forms of corporate equity investments
Mortgages	X42	Mortgages owed to the retirement system
Other Investments	X44	Mutual funds, international investments, loans to members and several other investments
Miscellaneous Investments	X47	All investments of the retirement system NEC
State and Local Government Sec.	X35	Included in X44 but also reported separately
Unemployment Cash and Securities		The sum of the following two variables
Unemployment in US Treasuries	Y07	The balance held in federal securities
Other Unemployment Balances	Y08	Negative when states borrow from the federal gov.
Non-Insurance Trust Cash and Sec.		The sum of the following three variables
Sinking Fund Cash and Securities	W01	Funds held in order to service debt
Bond Fund Cash and Securities	W31	Proceeds of bond issues awaiting disbursement
Other Non-Insurance Trust C&S	W61	All other non-insurance trust cash and investments

This table describes the coding of the cash and investment security variables and provides a short description of each. NEC stands for not elsewhere classified. The indentation of the variables in the first column indicates how subcategories of data collapse into larger categories. More detailed descriptions of each variable can be found in the Census' 2006 classification manual (http://www.census.gov/govs/classification/) included with the database download.

It may seem like an odd choice to report state and local government securities twice given the explicit division between governmental and non-governmental securities in the data, but given that defaults by state and local governments are more likely than federal defaults ([25] and many others) including state and local government bonds with non-federal securities is reasonable. For situations where this combination is unwanted state and local government securities can be subtracted out of the non-governmental securities variable.

Debt Positions

Debt statistics were significantly simplified following the 2005 redesign of the Census' government finance statistics program. Prior to this simplification data on debt were separated based on whether the debt was issued with the backing of the full faith and credit of the government in question, whether it was not guaranteed, or whether the guarantee was unspecified. Within each of those categories the debt was

broken out by function: debt to be used for each of the four utilities (water, electric, gas, and transit), general use debt, elementary and secondary education debt, or higher education debt. Measures of debt outstanding, debt issued, and debt retired were recorded for each of these guarantees and functions.

Debt outstanding, issued, and retired are still reported variables, but the distinctions between the guarantee levels and functions of debt have been removed. Instead, debt variables are disaggregated into public debt for private purposes, and debt for all other general purposes.

Because the data prior to 2005 have substantial additional detail, the government finance database keeps all of the potential categories of debt, even though many of these values are missing for the most recent years. Any research using the finer-grained debt data should exclude years prior to 2005, but the larger debt categories are comparable over the entire timespan of the data.

Discussion

Implications of Unbalanced Panel Data

The government finance database is an unbalanced panel dataset because the annual samples vary in size, and so any analysis of the data should be informed by traditional approaches to working with unbalanced panels, such as fixed effects models [26]. However, a deeper understanding of how the sample varies over time can provide us with advantages over the simple application of statistical tools, by guiding future research designs and by helping to interpret results.

One particularly striking finding from a high level analysis of the data is that smaller (larger) sample sizes indicate that the sample is skewed towards larger (smaller) governments. The graphs in Figs 3 and 4 show a clear, inverse relationship for counties (r = -0.93) and municipalities (r = -0.78) between the number of governments sampled each year and the median population of the governments in the sample, providing strong evidence that larger municipalities are more likely to be sampled during non-census years. The two government types that are exceptions to this pattern are states and school districts, because of the uniformly high reporting rates for both.

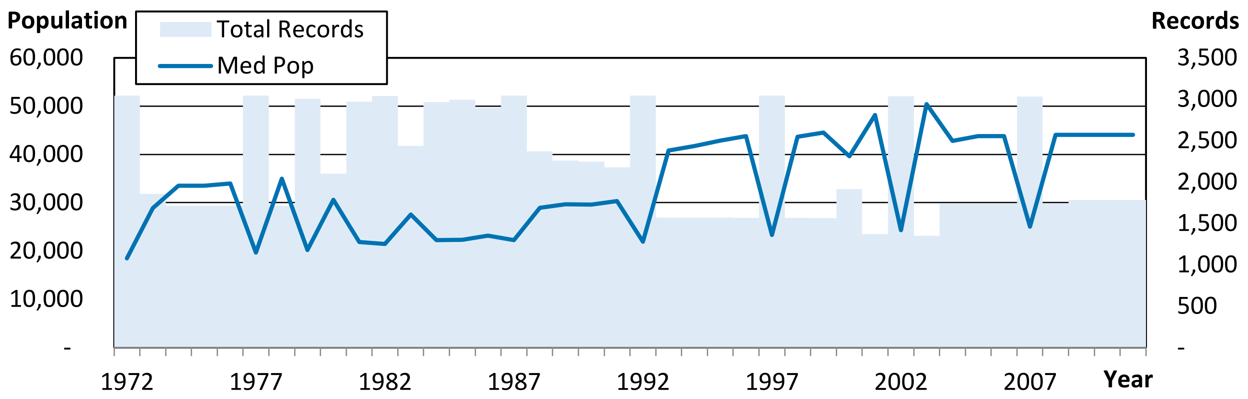

Fig 3. The Relationship between Sample Size and Population for Counties.
This figure shows how the median population reported in the data for counties correlates with the number of total records found in the data for counties. Median population is graphed against the left hand axis and the number of records is graphed against the right hand axis.

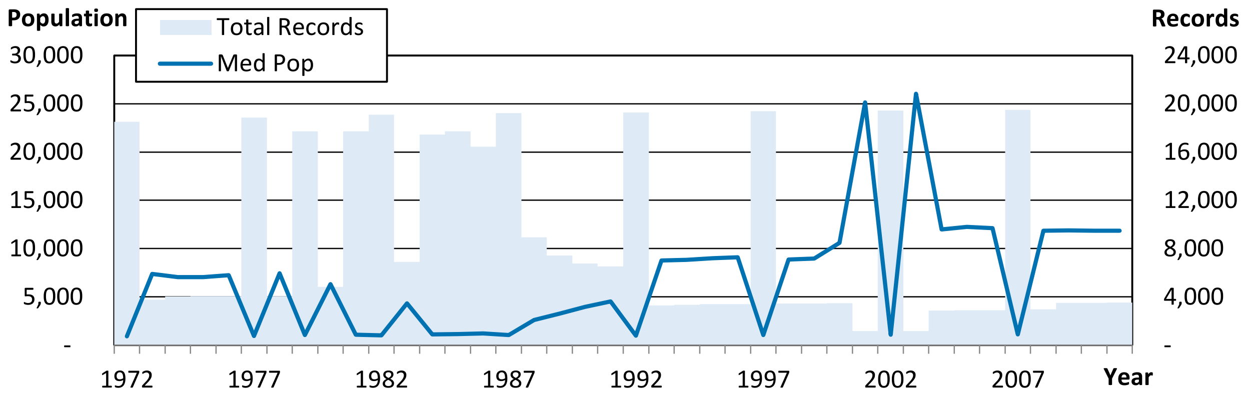

Fig 4. The Relationship between Sample Size and Population for Municipalities.
This figure shows how the median population reported in the data for municipalities correlates with the number of total records found in the data for municipalities. Median population is graphed against the left hand axis and the number of records is graphed against the right hand axis.

This relationship indicates several actionable steps, beyond the straightforward advice to apply year fixed effects, for quantitative research using this data. First, considering government size in your research design will be essential. Directly controlling for size, or being able to make a plausible argument for why size is not important for the question being investigated is an important bar for studies using this database to clear. If such controls or arguments are missing, academics and policy makers should be very wary of generalizing their results.

Second, your research focus may inform the data cleaning and selection process in novel ways. For instance, studies that aim to identify long term financial trends across all governments may want to only use the data from years ending in a 2 or a 7, because that will ensure that every measure they calculate is representative of a population of governments. Some of the time series we graph later in this paper will clearly show the impact that ignoring this advice can have.

On the other hand, studies that include or truncate data based on the population of each observation may claim to be including all of the data, but are actually removing much of what is available and are prejudicing their sample towards including more observations during the most recent years. While this type of data cleaning is often implemented without much thought in other fields, reviewers of work using the government finance database should ask authors to justify (or test to ensure) that the choice to include only governments with a certain population does not bias the results of the study.

While the number of governments sampled in any given year varies considerably, impacting the median population of the sample, it has long been understood that city populations follow a power law, or Pareto distribution ([27] or [28]), and thus it is reasonable to ask whether years with a small number of governments might nonetheless cover a large fraction of the population. Figs 5 and 6 take advantage of the fact that all states report data for every year to calculate the percentage of the total population covered by various government types each year.

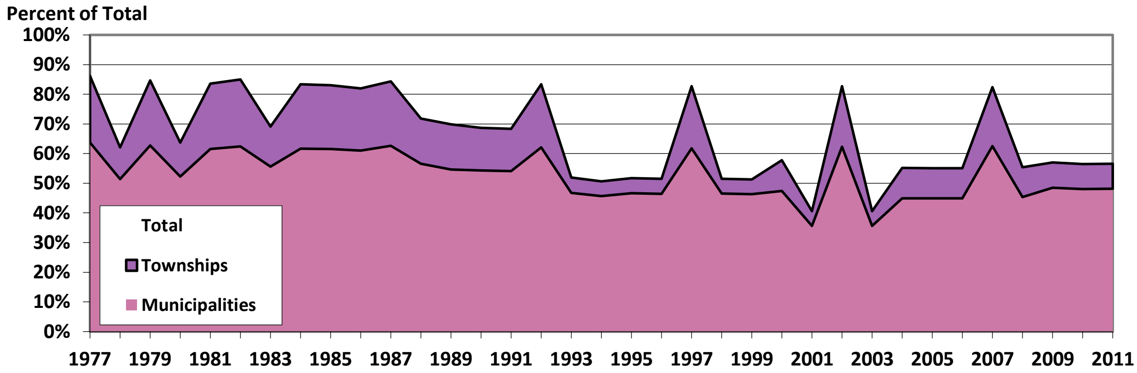

Fig 5. Population Coverage for Sampled Local Governments.
This figure stacks together the population covered by municipalities and townships, and compares it to the population indicated at the state level for every year of the data.

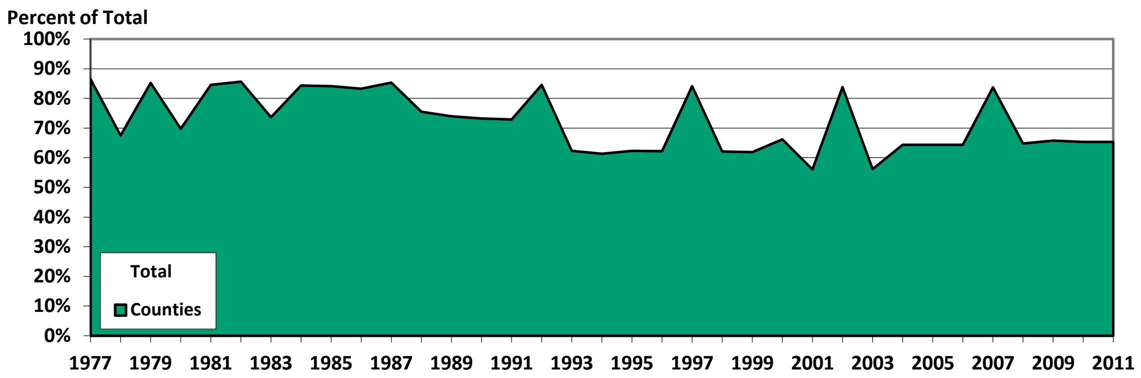

Fig 6. Population Coverage for Sampled County Governments.
This figure graphs the population covered by the counties in the database, and compares it to the population indicated at the state level for every year of the data.

What these figures show is that even though the samples are skewed towards governments with the largest populations, and so are not representative of all cities or all counties, they do capture a sizeable portion of the overall population in both cases.

An important implication of this is that studies which use the government finance database to measure the overall economic force of a particular category of government cash flow are likely to come very close to an accurate estimate, even during small sample years. Per capita numbers, which are easy to compute in the database, will often be a reasonable tool to use given that the data cover so much of the population. These measures will still be weighted towards representing people living in the largest governments during years when the sample is the smallest, but most people live in places with large populations and so per capita measures will be broadly representative.

There are likely several reasons why none of the years reach 100% coverage for population. One is that Connecticut and Rhode Island do not report county data, even though they both have counties. In addition, the District of Columbia is coded as a state and not as a local government. In practice however these reasons do not account for much of the gap. Other sources potentially include systematic non-reporting from less obvious sources, or the possibility that state population estimates are updated more often than other governments and so display growth sooner.

Another important consideration in working with unbalanced panel data is that requiring a long, uninterrupted time series of observations will limit the generality of your results. More specifically in this case, depending on the type of government being researched, requiring consecutive observations is likely to bias your sample towards including larger governments and data measured during the years in the late 1980's when the samples were larger. The size of this effect is controlled by the number of concurrent observations your research design requires however, so even small differences in such requirements have the potential to sizably impact your findings. Table 10 shows the number of observations that have consecutive data of a given length, and Table 11 shows how average population changes in those samples.

Table 10. The Impact of Requiring Consecutive Data on Sample Size.

Consecutive Years Required	All Reports	2-Year	3-Year	4-Year	5-Year
Federal	25	24	23	22	21
State	1,800	1,700	1,650	1,600	1,550
County	93,365	76,582	70,505	65,096	60,109
Municipality	361,300	208,715	170,286	144,915	121,995
Township	273,063	133,936	100,474	80,014	60,311
Special Districts	393,918	159,349	137,548	116,732	98,437
School Districts	488,319	425,588	395,090	367,150	339,564
All Types	1,611,790	1,005,894	875,576	775,529	681,987

This table displays how the sample size will change when researchers require consecutive years of data. The calculations are shown by government type, and each column increases the number of required years by one.

Table 11. The Impact of Requiring Consecutive Data on Average Population.

Consecutive Years Required	All Reports	2-Year	3-Year	4-Year	5-Year
Federal	229,703,936	229,703,936	229,703,936	229,703,936	229,703,936
State	5,148,833	5,148,833	5,148,833	5,148,833	5,148,833
County	95,796	108,182	113,368	118,457	123,753
Municipality	15,568	23,986	28,017	31,423	35,677
Township	4,963	7,521	8,923	10,087	11,967

This table displays how the average population of included governments will change when researchers require consecutive years of data. The calculations are shown by government type, and each column increases the number of required years by one.

Fig 7 graphs these sample sizes as a proportion of all of the available data for each government type.

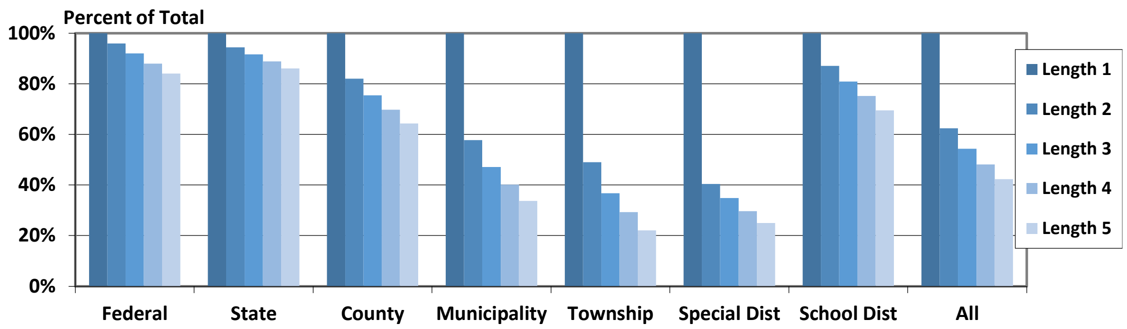

Fig 7. Proportion of Governments with Consecutive Years of Data.
This figure shows how the requirement of consecutive observations will limit the data that is available, and disaggregates the impacts by government type.

These points also suggest some practical considerations that arise when using unbalanced panel data in less academic settings. For instance, if you are interested in discovering something about the revenues or expenses of a particular local government you are not likely to find a complete time pattern of behavior in this database. In fact, unless the government you are interested in serves a particularly large population you may find that data only exist once every five years. Individuals who want to dig deeply into the finances of a particular local government are likely to have much better luck asking for financial records directly from the local government they are interested in.

Results

In the process of organizing and cleaning the data we were struck by its wide applicability to many different areas of public administration. In this section we present a number of simple analyses that illustrate both the flexibility and usability of the government finance database.

Fig 8 is a good example. It shows a time series of the average number of tax revenue sources computed for both municipalities and school districts. These data were constructed by adding an indicator variable to the database for each type of tax revenue. The indicator was coded as a 0 whenever the total amount of that tax was either missing or equal to zero, and was coded as a 1 otherwise. The indicators for property taxes, sales taxes, income taxes, license taxes, and other taxes were then summed and the average was calculated, by year, for each government type.

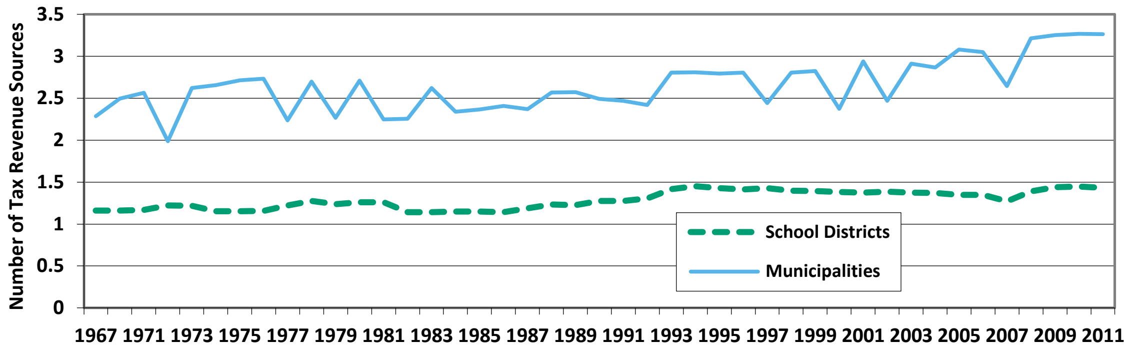

Fig 8. Number of Tax Revenue Sources by Government Type.
This figure shows how the diversity of tax revenue sources increases over time for both municipalities and school districts.

The results show two interesting features. The first is a quantitative confirmation of the often-noted trend towards increasing revenue diversification by municipal governments [29]. This trend is mirrored by school districts, a fact which is far less well known. The second notable feature is that the number of municipal tax revenue sources *looks* much more variable than the number of school district tax revenue sources. In fact, much of that variability is induced by the different sample sizes (and therefore the different average populations) each year.

Fig 9 shows a similar analysis that also highlights several additional considerations for using the data. It graphs average, real, per capita government debt at both the state and municipal levels.

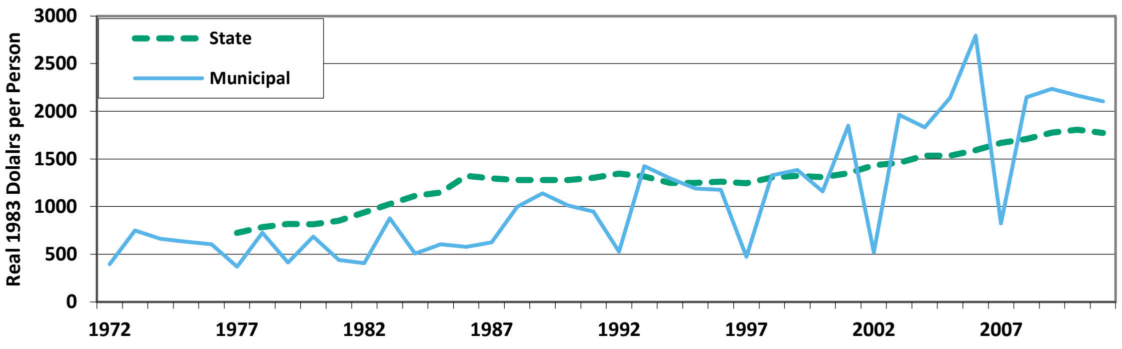

Fig 9. Real per Capita Debt by Government Type.
This figure plots real, per-capita debt per person at both the state and municipal level.

Scaling by population is easy, since population figures are included in the data, but because the database is recorded in nominal thousands of dollars any analysis that wants to control for inflation needs to merge an appropriate scaling factor into the database. In this case we used the annual average CPI levels from the Bureau of Labor Statistics (with 1983 ≅ 1), scaled total debt outstanding by both CPI and population, and multiplied each resulting figure by 1,000 (to correct for the fact that all data in the government finance database is recorded in thousands of dollars). The government level figures were then averaged, by year, for each government type.

On the surface the graph in Fig 9 shows many of the features described by Hildreth and Zorn [30], including a substantial increase in debt levels following the Tax Reform Act of 1986, decreasing new issues in the early 1990's, and a general upward trend in debt outstanding since. Beyond those well-known trends however the real, per capita levels tell an interesting story about how large and small municipalities have used debt markets differently.

Prior to 1986 census years show relatively little difference from annual samples in terms of the average level of real debt per person. Following the 1987 census however those differences dominate the figure, indicating that large municipalities have taken advantage of the Tax Reform Act of 1986 far more than small municipalities, even in inflation adjusted per capita terms. While city size has been studied in relation to its impact on interest rates [31] [32], this previously unnoticed pattern between city size and the level of outstanding municipal debt is a potential area for future research.

There is no reason why the data need to be analyzed from the aggregate perspective our previous two figures used. Breaking the data out and studying one particular government is also an interesting exercise. For instance, Fig 10 shows the state of Oregon's total revenue and total expenditure, in billions of nominal dollars through time.

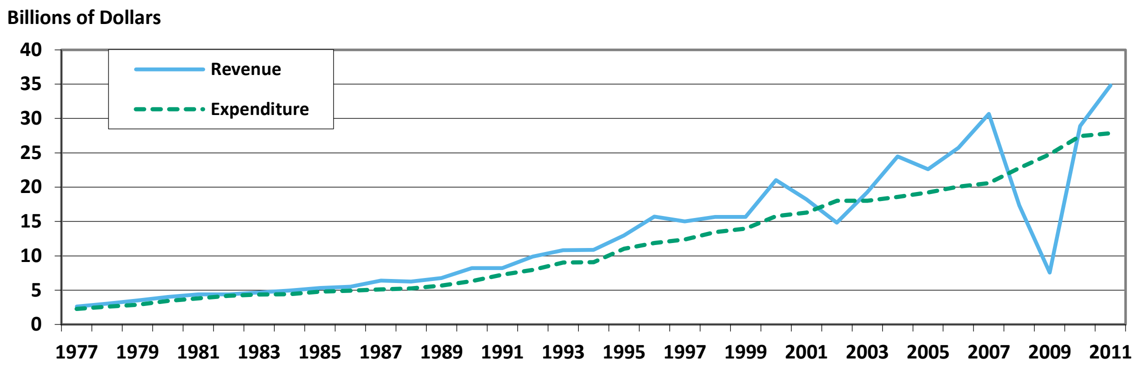

Fig 10. Oregon Total Revenue and Total Expenditure.
This figure shows the total revenue and total expenditure of Oregon State government over time.

The most striking feature of this graph is the sizeable impact of the great recession on total revenue in 2009. Contrary to what you might think, this change is not the result of a large decrease in taxes collected or any other traditional revenue source, instead virtually all of the difference between the 2008 and 2009 numbers comes from the approximately $12 billion dollar loss from public employee retirement system investment revenue.

The visual impact of this loss on the graph is small compared to the actual impact losses like this had on public retirement systems across the nation and the world [32], but it drives home an important point about the flexibility of the government finance database. Isolating more stable government revenues through the use of general revenue, rather than total revenue, is likely to be advisable in many situations, and further isolating your data from the impact of intergovernmental revenue by using the “own source” versions of either revenue number is also possible.

Another option for segmenting the data is to look more closely at patterns within a particular government type. Table 12 shows one such analysis for special districts. While the general pattern of growth in special districts is well known [34] [35], and there are a few isolated studies that attempt to understand what is driving that growth (cf. [36], a study using one year of data, to our table), there are no studies describing which types of special districts have contributed most to that growth.

Table 12. Special District Growth.

Special District Category	Code	1977 Ct.	2007 Ct.	Δ%	1977 %	2007 %
Total	-	25,987	35,574	37%	100.00%	100.00%
Local Fire Protection	24	4,186	5,814	39%	16.11%	16.34%
Water Supply Utility	91	2,481	3,424	38%	9.55%	9.63%
Housing and Community Development	50	2,412	3,391	41%	9.28%	9.53%
Other Multi-function Districts	99	517	2,545	392%	1.99%	7.15%
Soil and Water Conservation	88	2,431	2,531	4%	9.35%	7.11%
Drainage	51	2,254	2,021	-10%	8.67%	5.68%
Sewerage	80	1,608	1,867	16%	6.19%	5.25%
Libraries	52	588	1,663	183%	2.26%	4.67%
Cemeteries	2	1,615	1,588	-2%	6.21%	4.46%
Sewerage and Water Supply	98	1,064	1,359	28%	4.09%	3.82%
Parks and Recreation	61	830	1,320	59%	3.19%	3.71%
Other Single Function Districts	89	312	926	197%	1.20%	2.60%
Irrigation	64	933	827	-11%	3.59%	2.32%
Regular Highways	44	652	813	25%	2.51%	2.29%
Health	32	356	768	116%	1.37%	2.16%
Hospitals	40	717	671	-6%	2.76%	1.89%
Flood Control	63	681	588	-14%	2.62%	1.65%
School Building Authorities	9	1,019	522	-49%	3.92%	1.47%
Air Transportation	1	299	490	64%	1.15%	1.38%
Solid Waste Management	81	71	425	499%	0.27%	1.19%
Public Mass Transit Utility	94	96	356	271%	0.37%	1.00%
Other Natural Resources	59	179	336	88%	0.69%	0.94%
Miscellaneous Commercial Activities	3	0	297	-	0.00%	0.83%
Industrial Development	41	0	168	-	0.00%	0.47%
Sea and Inland Port Facilities	87	166	162	-2%	0.64%	0.46%
Electric Power Utility	92	82	154	88%	0.32%	0.43%
Reclamation	86	114	149	31%	0.44%	0.42%
Natural Resources and Water Supply	97	71	87	23%	0.27%	0.24%
Fire Protection and Water Supply	96	66	59	-11%	0.25%	0.17%
Gas Supply Utility	93	46	57	24%	0.18%	0.16%
Public Welfare Institutions	77	0	51	-	0.00%	0.14%
Mortgage Credit	42	0	39	-	0.00%	0.11%
Parking Facilities	60	122	32	-74%	0.47%	0.09%
Correctional Institutions	4	0	23	-	0.00%	0.06%
Police Protection	62	0	23	-	0.00%	0.06%
Toll Highways	45	0	15	-	0.00%	0.04%
Public Welfare	79	0	12	-	0.00%	0.03%
Other Corrections	5	0	1	-	0.00%	0.00%
Unknown	0	19	0	-100%	0.07%	0.00%

This table displays the growth of special districts by comparing the 1977 data with the 2007 data, and is ordered by the number of districts existing in the 2007 data. The absolute number of special districts of each type is displayed, along with the percentage change between the two years, and the proportion of all special districts that each type comprises. The code column is the special district function code used by the census.

Our findings demonstrate a number of interesting patterns. First, much of the growth seems to be an organic expansion of the most common special districts without much change in their proportion. For instance, even though local fire protection districts added 1,628 to their total and grew almost 40% over the 30 years, they represented a very stable 16% of all special districts at both points in time. Second, some of the most dramatic growth came from the other multi-function district category, which grew from around 2% to over 7% of all districts, indicating that citizens who form special districts are increasingly deciding that the efficiency of combining multiple functions (perhaps from economies of scale, or reductions in administrative costs), outweighs the burden arising from additional complexity.

There are many other interesting storylines that we might draw from this Table 12, including the reduction in school building authorities and cemeteries potentially representing shifts in population demographics, or the strong growth of library, health, and solid waste management districts potentially representing increased demand for those services in areas without the population to support them previously. The diversity of potential insights from this relatively simple analysis highlights the fact that we can only begin to characterize the full extent of the flexibility and utility of the government finance database here.

Conclusion

A trade-off between ease of use and purity exists with any data cleaning effort. On the one hand we would like to present researchers with a database that is free from abnormalities and can be easily used in the widest range of circumstances. On the other hand we also want a database that is as close to the raw data as possible in order to limit statistician induced measurement error.

In this effort we have made several decisions that ensure the purity of the data even when there is some reduction in its usability. We plan to implement fixes for these issues, but have reserved these changes for a later work, since this will give us an opportunity to describe our approach to the data cleaning in a complete way, and because our approaches are potentially controversial. Academics who appreciate the changes we plan to make are free to apply them or use our revised database, and those who

disagree with us or would prefer to use an alternate method can still have access to the government financial data in this form. A brief description of the issues we would like to fix is warranted however, since our choosing to not amend the database now means that the data may have less utility for some studies.

The two primary issues surround the population figures and the fiscal year end dates. The issue with the population numbers is that they do not update annually. Given the fact that per capita levels are a common, useful transformation to apply to government finance statistics, the use of old population figures means that per capita variables are likely to be measured with error in many cases. Short of conducting a retrospective count of populations for every government in the dataset the best solution is to model what the population must have been in every year when the population estimate is not current. This model could take many different forms, so we will reserve a discussion of how to create it for the future.

The issue with the fiscal year end dates is threefold: inconsistent coding of dates, a large number of error codes that we can interpret, and a surprising collection of other strange entries that are harder to interpret. The bluntest illustration of this problem is that fact that there are 520 unique values of fiscal year end dates, and only 365 days in a year. There are several avenues for correcting this problem, none of which is perfect. In the meantime however research that relies on fiscal year end dates should be careful of dropping observations that don't conform to the expected format of this data field.

While the data we provide is far from perfect it still represents a substantial step forward for quantitative research in public financial analysis, and helps to solve a long-standing problem created by the lack of standardized, cross-sectional databases in local government finance.

All of the data was collected by the U.S. Census Bureau's annual surveys and five-year censuses of state and local government finance, but prior to our work the use of government financial data in public accounting and finance research always involved a substantial investment of time into data cleaning and organization. As a result there was very little standardization in the

time periods and government types covered, and the interpretability, accessibility, and replicability of research suffered.

We offer this database in the hopes that it can bring more consistency and transparency to quantitative research in public financial management. In the process it should also make conducting this type of research less costly, and may provide a template for others with access to unique data sources who want to provide them to our field.

References

[1] Gill J, & Meier KJ (2000) Public administration research and practice: A methodological manifesto. *Journal of Public Administration Research and Theory*, *10*(1), 157-199.

[2] Gore AK (2004) The effects of GAAP regulation and bond market interaction on local government disclosure. *Journal of Accounting and Public Policy*, *23*(1), 23-52.

[3] Baber WR. Gore AK (2008) Consequences of GAAP disclosure regulation: Evidence from municipal debt issues. *The Accounting Review*, *83*(3), 565-592.

[4] Kido N Petacchi R Weber J (2012) The influence of elections on the accounting choices of governmental entities. *Journal of Accounting Research*, 50(2), 443-476.

[5] Murray SE Evans WN Schwab RM (1998) Education-finance reform and the distribution of education resources. *American Economic Review*, 789-812.

[6] Carroll DA (2009) Diversifying municipal government revenue structures: Fiscal illusion or instability?. *Public Budgeting & Finance*, 29(1), 27-48.

[7] Mullins DR (2004) Tax and Expenditure Limitations and the Fiscal Response of Local Government: Asymmetric Intra-Local Fiscal Effects. *Public Budgeting & Finance*, 24(4), 111-147.

[8] Fisher RC Papke LE (2000) Local government responses to education grants. *National Tax Journal*, 153-168.

[9] Mikesell JL Ross JM (2012) Fast money? The contribution of state tax amnesties to public revenue systems. *National Tax Journal*, *65*(3), 529-562.

[10] Kurban H Gallagher RM Persky JJ (2012) Estimating local redistribution through property-tax-funded public school systems. *National Tax Journal*, *65*(3), 629-65

[11] Chapman J Gorina E (2012) Effects of the Form of Government and Property Tax Limits on Local Finance in the Context of Revenue and Expenditure Simultaneity. *Public Budgeting & Finance*, *32*(4), 19-45.

[12] Witko C Newmark AJ (2010) The Strange Disappearance of Investment in Human and Physical Capital in the United States. *Journal of Public Administration Research and Theory*, *20*(1), 215-232.

[13] Hou Y (2006) Budgeting for fiscal stability over the business cycle: A countercyclical fiscal policy and the multiyear perspective on budgeting. *Public Administration Review*, *66*(5), 730-741.

[14] Vallés-Giménez J Zárate-Marco A (2011) Disparities in the Spending Needs of Highland Municipalities. *Public Budgeting & Finance*, *31*(3), 26-48.

[15] Breunig C Koski C Mortensen PB (2010) Stability and punctuations in public spending: A comparative study of budget functions. *Journal of Public Administration Research and Theory*, *20*(3), 703-722.

[16] Meier KJ Morton T Molina A (2010) *Texas School District Performance Data 1993-2009*. College Station, TX: Project For Equity, Representation and Governance.

[17] Krause GA Meier KJ (2003) *Politics, policy, and organizations: frontiers in the scientific study of bureaucracy*. University of Michigan Press.

[18] Kelman S (2005) Public management needs help!. *Academy of Management Journal*, 48(6), 967-969.

[19] Bartle JR (2001) *Evolving Theories of Public Budgeting*. Amsterdam, New York: Emerald Group Publishing Limited.

[20] Raadschelders JC (2013) *Public administration: The interdisciplinary study of government*. Oxford University Press.

[21] GASB, *Concepts Statement No.1 of the Governmental Accounting Standards Board: Objectives of Financial Reporting* (Norwalk, CT: GASB, 1987)

[22] GASB, *Statement No. 34* (Norwalk, CT: GASB, 1999).

[23] Voorhees WR Kravchuk RS (2001) The new governmental financial reporting model under GASB statement no. 34: an emphasis on accountability. *Public Budgeting & Finance*, 21(3), 1-30.

[24] Ingram RW (1984) Economic incentives and the choice of state government accounting practices. *Journal of Accounting Research*, 126-144.

[25] Chalmers JM (1998) Default risk cannot explain the muni puzzle: Evidence from municipal bonds that are secured by US Treasury obligations. *Review of Financial Studies*, *11*(2), 281-308.

[26] Baltagi B (2008) *Econometric analysis of panel data* (Vol. 1). John Wiley & Sons.

[27] Beckmann MJ (1958) City hierarchies and the distribution of city size. *Economic Development and Cultural Change*, *6*(3), 243-248.

[28] Blank A Solomon S (2000) Power laws in cities population, financial markets and internet sites (scaling in systems with a variable number of components). *Physica A: Statistical Mechanics and its Applications*, *287*(1), 279-288.

[29] Hendrick R (2002) Revenue diversification: Fiscal illusion or flexible financial management. *Public budgeting & finance*, *22*(4), 52-72.

[30] Hildreth WB Zorn C (2005) The evolution of the state and local government municipal debt market over the past quarter century. *Public Budgeting & Finance*, *25*(4s), 127-153.

[31] Rivers MJ Yates BM (1997) City size and geographic segmentation in the municipal bond market. *The Quarterly Review of Economics and Finance*, *37*(3), 633-645.

[32] Simonsen B Robbins MD Helgerson L (2001) The influence of jurisdiction size and sale type on municipal bond interest rates: An empirical analysis. *Public Administration Review*, *61*(6), 709-717.

[33] Pino A Yermo J (2010) The impact of the 2007-2009 crisis on social security and private pension funds: A threat to their financial soundness? *International Social Security Review*, *63*(2), 5-30.

[34] Carr JB (2006) Local government autonomy and state reliance on special district governments: A reassessment. *Political Research Quarterly*, *59*(3), 481-492.

[35] McCabe BC (2000) Special-district formation among the states. *State & Local Government Review*, 121-131.

[36] Nunn S Schoedel C (1997) Special districts, city governments, and infrastructure spending in 105 US metropolitan areas. *Journal of Urban Affairs*,*19*(1), 59-72.

Supporting Information

S1 File. Appendix for the Government Finance Database. This file contains detailed information showing how to replicate our work creating the database described in the paper. The three appendices include step by step instructions, a mapping of our variable names to the census data codes, and the SAS code we used to consolidate the data files we received from the Census into the government finance database.
